# GAS‐J, a User‐Friendly Browser Application for Genome Assembly, *emm*‐Typing, MLST Typing, and Virulence Factor Gene Detection of *Streptococcus pyogenes*

**DOI:** 10.1111/1348-0421.13223

**Published:** 2025-04-20

**Authors:** Norihiko Takemoto, Kohei Ogura, Yuan Gao, Rumi Okuno, Masaya Yamaguchi, Yujiro Hirose, Masayuki Ono, Shigetada Kawabata, Tadayoshi Ikebe, Takashi Hamabata, Tohru Miyoshi‐Akiyama

**Affiliations:** ^1^ Department of Infectious Diseases Research Institute, National Center for Global Health and Medicine Tokyo Japan; ^2^ Laboratory of Basic and Applied Molecular Biotechnology, Division of Food Science and Biotechnology, Graduate School of Agriculture Kyoto University Kyoto Japan; ^3^ Tokyo College of Medico‐Pharmaco‐Nursing Technology Tokyo Japan; ^4^ Department of Microbiology Tokyo Metropolitan Institute of Public Health Tokyo Japan; ^5^ Laboratory of Microbial Informatics Microbial Research Center for Health and Medicine, National Institutes of Biomedical Innovation, Health and Nutrition Osaka Japan; ^6^ Bioinformatics Research Unit, Graduate School of Dentistry Osaka University Osaka Japan; ^7^ Bioinformatics Center, Research, Institute for Microbial Diseases Osaka University Osaka Japan; ^8^ Department of Microbiology, Graduate, School of Dentistry Osaka University Osaka Japan; ^9^ Center for Infectious Diseases Education and Research Osaka University Osaka Japan; ^10^ Department of Bacteriology I National Institute of Infectious Diseases Tokyo Japan

**Keywords:** *emm* typing, multilocus sequence typing, *Streptococcus pyogenes*, virulence factor, web tool

## Abstract

Clinical isolates of *Streptococcus pyogenes* are usually classified using *emm* and multilocus sequence typing (MLST). Recently, whole genome sequencing (WGS) has been employed for *emm* typing and MLST analysis. WGS data provides additional information on the presence of virulence factor genes. To enable researchers unfamiliar with bioinformatics to analyze WGS data of *S. pyogenes*, we opened an online tool named GAS‐J, which automatically outputs *emm* types, sequence types (STs), carriage of virulence factor genes, and phylogenetic trees. The tool accepts raw short‐read data as inputs, since it includes the velvet assembler. In all isolates, the *emm* typing results from this tool were identical to those obtained by conventional PCR and Sanger sequencing, even in cases where isolates had pseudo‐*emm* (*emm*‐like) genes. STs are determined by performing a BLAST search using seven alleles as references. To detect *S. pyogenes* virulence factor genes, we prepared a new data set containing 620 related proteins. Users may choose which isolates to include in SNP‐based phylogenetic tree from a pool of 406 isolates with epidemiological data. The data set includes isolates whose symptoms (STSS or non‐STSS) were diagnosed based on the STSS criteria of the Japan Communicable Disease Prevention Law. GAS‐J application is available at http://gasj.ncgm.go.jp. The data of isolates are going to be updated in the future.

**Abbreviations**
GAS: group A *Streptococcus*
MLST: multilocus sequence typing
STSS: streptococcal toxic shock syndrome

## 1 Introduction

*Streptococcus pyogenes*, also known as group A *Streptococcus* (GAS), is responsible for a variety of human diseases ranging from relatively mild symptoms such as pharyngitis and impetigo to a severe and life‐threating condition known as streptococcal toxic shock syndrome (STSS). STSS can affect patients of any age, even those without underlying diseases or immunodeficiency. The initial symptoms of STSS include pain, swelling, fever, and low blood pressure in the extremities, followed by rapid and significant deterioration [1]. According to a 2021 report by the Centers for Disease Control and Prevention USA, approximately 1200–1900 people die each year due to invasive GAS disease (https://www.cdc.gov/streplab/groupa-strep/index.html).

In Japan, based on the Communicable Disease Prevention Law, STSS is defined as a shock‐inducing illness with two or more of the following symptoms: liver failure, renal failure, acute respiratory distress syndrome, disseminated intravascular coagulation, soft tissue inflammation (including necrotizing fasciitis), systemic erythematous rash, and central nervous system disease (such as convulsions and loss of consciousness). For treatment of STSS, clindamycin in combination with penicillin has been used [2]. A recent systematic review and meta‐analysis conducted by Bartoszko et al. indicated that clindamycin‐treated patients had low mortality rates, although the confidence in the evidence was deemed low [3].

Conventionally, *S. pyogenes* isolates have been typed using the *emm* gene sequences, which encode the anti‐phagocytic M protein [4] (*emm‐*typing). Previously, *emm‐*typing utilized the first 90 bases that encode the processed (mature) form of the M protein. However, there is a significant risk of mistyping due to the presence of *emm*‐like (pseudo‐*emm*) genes located immediately upstream (*mrp* gene) and downstream (*enn* gene) of the *emm*‐gene in 85% of *S. pyogene* isolates [5]. Furthermore, there is homology between the *emm* gene and the *sph* gene, which is found downstream of the *emm* gene in a limited number of *emm*‐types [5]. To address these challenges, *Streptococcus* Laboratory Home (https://www.cdc.gov/streplab/groupa-strep/emm-background.html) offers an improved *emm*‐subtyping method. The method utilizes a 180 bp subtype‐encoding sequence, consisting of 60 codons that encode the signal sequence (10 codons) plus the mature M protein (50 codons), available online [6]. Additionally, another *emm*‐typing protocol has also been proposed [7]. Multilocus sequence typing (MLST) has also been employed to characterize the genetic traits of *S. pyogenes* [8]. This MLST scheme classifies *S. pyogenes* based on the allele sequences of seven housekeeping genes: glucose kinase (*gki*), glutamine transporter protein (*gtr*), glutamate racemase (*murI*), DNA mismatch repair protein (*mutS*), transketolase (*recP*), xanthine phosphoribosyl transferase (*xpt*), and acetyl coenzyme A acetyltransferase (*yqiL*) genes.

Advancements in bioinformatic tools have made it increasingly convenient to analyze the genomic characteristics of isolates. However, researchers who lack expertise in genomics or informatics may encounter challenges when working with raw read data obtained through whole genome sequencing. These challenges include the need to perform de novo assembly, type isolates using *emm* and MLST, detect virulence factor genes, and conduct comparative genome sequencing through phylogenetic analysis. It would greatly benefit clinical researchers and novice genome analysts alike if there were a tool they could use to analyze the genome from assembly to typing and identifying virulence factors simultaneously. In this report, we introduce GAS‐J, which is our newly developed online tool specifically designed for *S. pyogenes* analysis.

## 2 Materials and Methods

### 2.1 Collection of Genomic Sequences

*S. pyogenes* isolates were obtained from regional public health institutes in Japan. We obtained 406 *S. pyogenes* isolates whose epidemiological data were available including the year of isolation, the prefecture of isolation in Japan, and information on symptoms (STSS or non‐STSS) (Table S1). The classification of symptoms as STSS or non‐STSS was based on the criteria outlined in the Japan Communicable Disease Prevention Law. Genomic DNA was extracted from the isolates using DNeasy blood and tissue kit (QIAGEN) and sequencing was performed using HiSeq X (Illumina). The raw sequencing reads were subjected to de novo assembly using the CLC Genomics Workbench software (QIAGEN). We confirmed that each genome was derived from *S. pyogenes* by its 16S rRNA gene sequence.

## 3 Results and Discussion

### 3.1 Overview of GAS‐J

**Figure 1:**
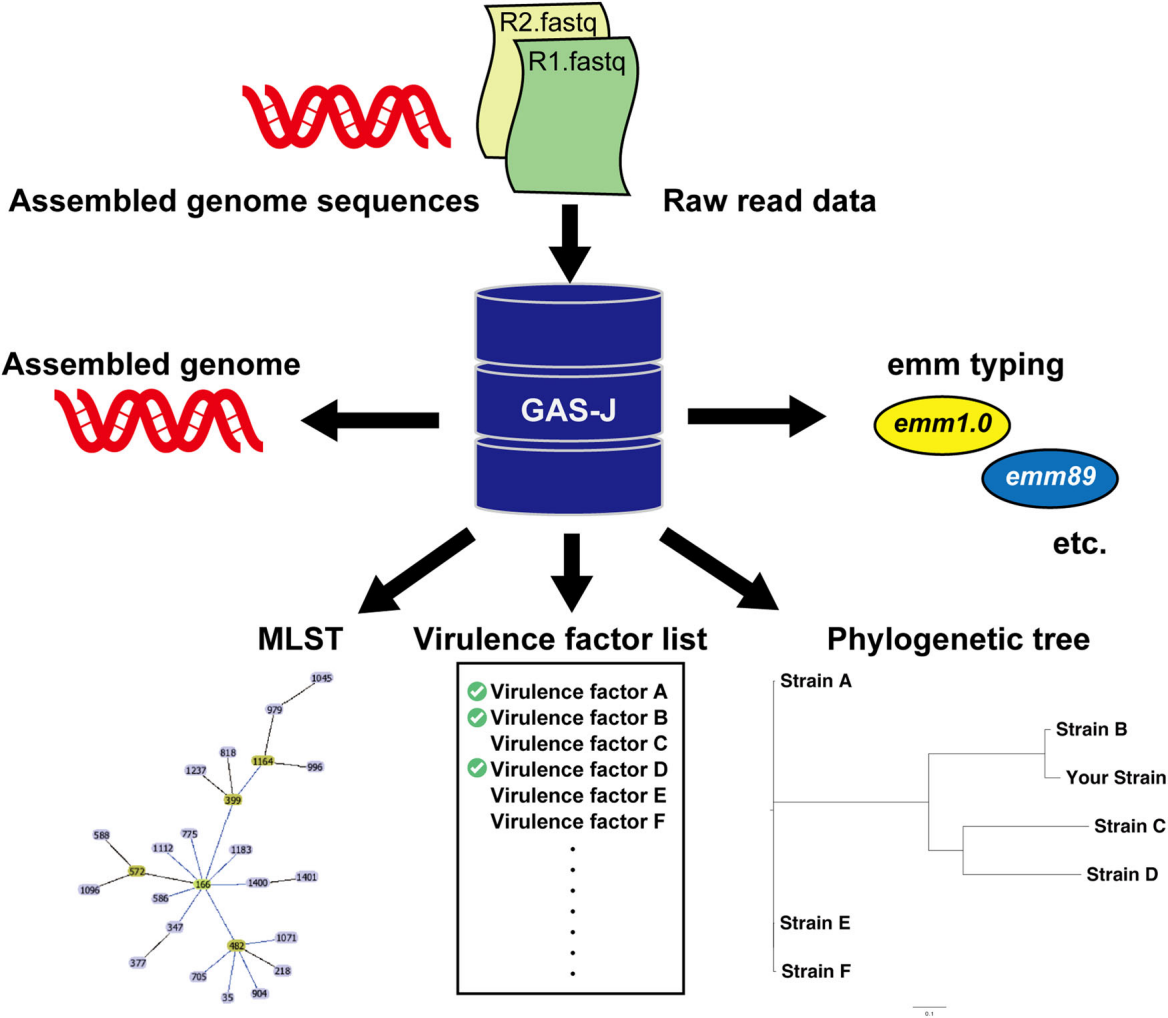
Overview of the online tool. The tool accepts assembled sequences and raw short‐read data of *Streptococcus pyogenes* as inputs, and detects *emm* types, sequence types (STs), carriage of virulence factor genes, and phylogenetic trees.

This online tool is designed to analyze genomic sequences of *S. pyogenes* and provide information on *emm* type, MLST, virulence factors, and phylogenicity (Figure 1). Additionally, users have access to genomic sequences and epidemiological data for 406 strains isolated from patients with STSS and non‐STSS in Japan (Table S1). The data set allows users to compare their own strain data with various combinations of epidemiological information including year of isolation, prefecture in Japan where isolated, and STSS/non‐STSS classification combinations.

### 3.2 Input Sequences

**Figure 2:**
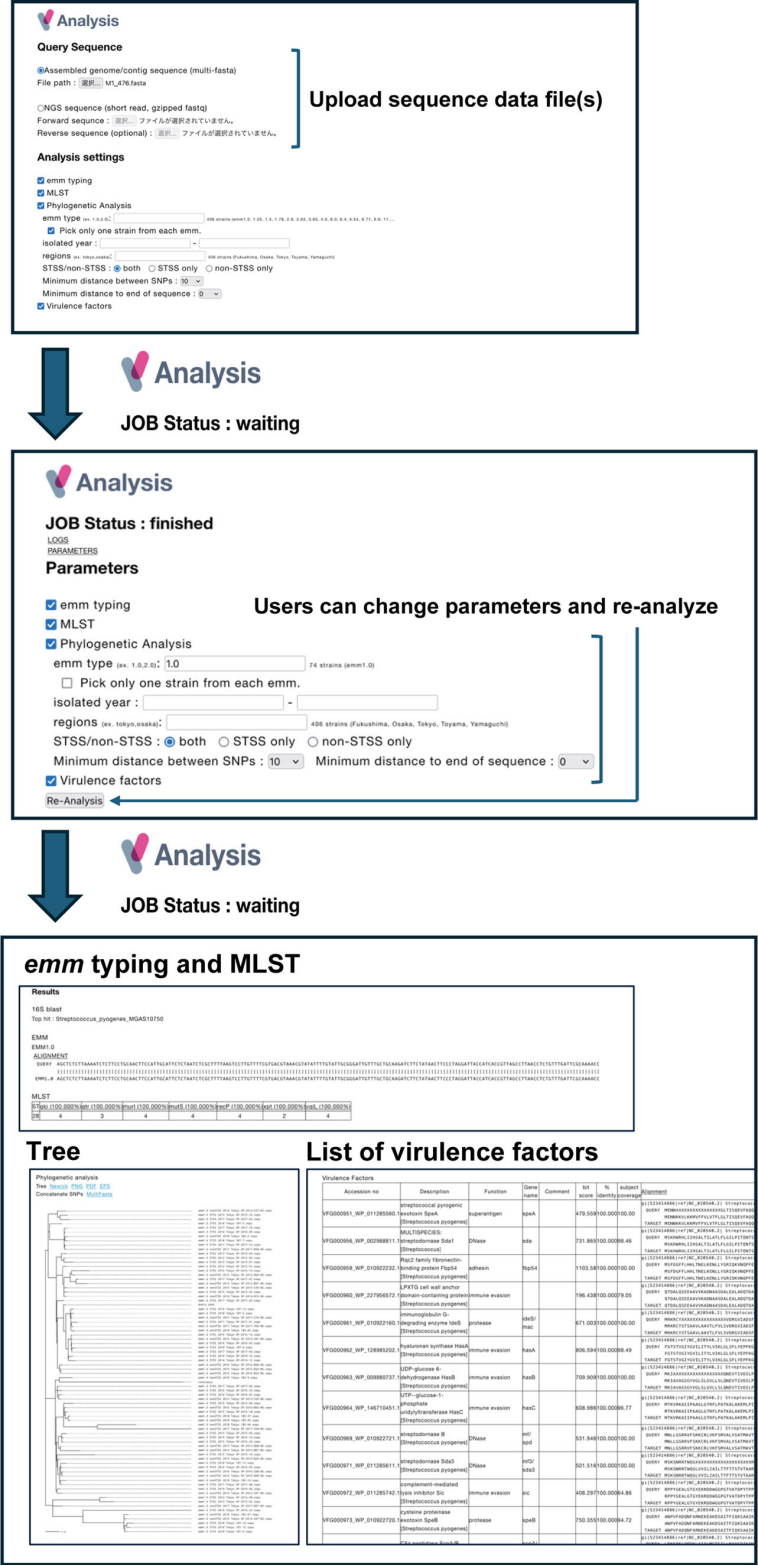
Example screenshots of the analysis. Users can upload assembled genome sequences and raw short‐read data, selecting options such as *emm* typing, MLST, phylogenetic analysis parameters, and virulence factor detection.

This application accepts both assembled sequences (contigs) and raw read data (fastq) as inputs. If raw read data is provided, it is de novo assembled online using the Velvet assembler [9]. We chose the Velvet assembler for this application because it has faster processing times compared to Platanus B, which includes multiple error‐removal algorithms [10]. To ensure that the assembled sequences as queries are derived from *S. pyogenes*, they are searched against a data set of 16S rRNA gene sequences using BLAST as a reference. The application stops at this point if coverages of aligned 16S rRNA gene sequences are lower than 50% of their references and/or their identities to the *S. pyogenes* sequences are lower than 99%. Figure 2 shows an example of the analysis procedure on the website.

### 3.3 *emm* Typing Scheme

The *emm* typing scheme used in this study involves analyzing DNA sequences differently obtained from *emm* databases in CDC *Streptococcus* Laboratory Home (https://www.cdc.gov/streplab/index.html) using BLAST search. In most strains of *S. pyogenes*, the *emm* genes are flanked by two *emm*‐like genes, *mrp* and *enn* [5]. As reported by Frost et al. [11], *emm* and *enn* genes can undergo recombination through homologous recombination [5, 12]. In some cases, the BLAST search resulted in multiple hits for different *emm* types. For example, when we analyzed a specific sequence data (Supporting Information S1: Data set 1), EMM4.0, EMM156.0, and EMM236.3 were all found to have 100% similarity with the analyzed sequence. To determine the *emm* type, the following steps were included in our application. First, the submitted genomic sequence was used as a query, and the *emm* data set served as the subject for a BLAST search. This allowed us to detect *emm* and *emm‐*like genes. Second, a consensus sequence (Supporting Information S2: Data set 2) located immediately downstream of the 180‐bp subtype‐encoding sequence was used as a query for another BLAST search, with the submitted genomic sequence as the subject. Finally, the closest *emm* sequence to the consensus sequence was then chosen for *emm* typing. For example, the sequence from Supporting Information S1: Data set 1 was classified as *EMM*4.0. We confirmed that the results obtained using this scheme were identical with those obtained through PCR and Sanger sequencing.

### 3.4 MLST Typing

The allele sequences for the seven house‐keeping genes were obtained from the *S. pyogenes* section in the pubMLST database [8, 13]. The genomic data (contigs) and the allele sequences were utilized as queries and references, respectively. The purpose of this application is to identify matched sequence types (STs) or the closest STs based on the analysis of these sequences.

### 3.5 Database of Virulence Factor Genes

Approximately 160,000 refseq_protein data were collected from the NCBI website and used to create a virulence factor database. Of the approximately 2000 CDSs possessed by *S. pyogenes*, 620 were listed as candidate virulence factors. To detect the substitution of amino acids in each virulence factor, this database includes 21,936 amino acid sequences without redundancy. In our analysis, we used the genomic data (contigs) and the virulence factor database as queries and references, respectively, for the BLASTX search. This allowed us to compare and identify any matches or similarities between the genomic data and the annotated protein sequences in the database. This database will be updated according to the identification of new virulence factors.

### 3.6 Phylogenetic Analysis

This application generates SNP‐based phylogenetic trees, allowing users to select specific isolates from a pool of 406 isolates to include in the tree. For instance, users have the flexibility to select isolates based on various criteria such as specific *emm* types (such as *emm*1 and *emm*3) or *emm* sub‐types (such as *emm*1.25), year of isolation, and location where the isolate was obtained.

### 3.7 Update of Strains and Typing Database

The current database includes only strains isolated before the coronavirus disease 2019 (COVID‐19) pandemic. However, the epidemiology of GAS infections has changed drastically worldwide during and after the pandemic [14, 15, 16]. An epidemic of new lineages of GAS, including global M1UK, is a concern worldwide [17, 18, 19, 20, 21]. Therefore, strains isolated during and after the pandemic will be added to the reference data for phylogenetic analysis in the future. According to new strains, MLST database will be updated along with the update.

## Ethics Statement

This study was conducted in accordance with the Declaration of Helsinki and approved by the Ethics Committee of the National Center for Global Health and Medicine (NCGM; receipt no. 004867; date of approval: July 4, 2024).

## Conflicts of Interest

The authors declare no conflicts of interest. Kohei Ogura is an Editorial Board member and is also a coauthor of this article. To minimize bias, he was excluded from all editorial decision‐making related to the acceptance of this article for publication. Late Tohru Miyoshi‐Akiyama served on the Editorial Board until 2023, but is no longer a member of the Board and is therefore not involved in any editorial decision‐making.

## Supporting information

Supplemental_Dataset1_contig.

Supplemental_Dataset2_consensus_emm.

Table S1. List of S. pyogenes isolates incuded in the dataset.

## Data Availability

GAS‐J application is available at http://gasj.ncgm.go.jp. The system and data that support the development of this application are available from the corresponding author (K.O.) upon reasonable request.
