# Supplementary material for: GAS‐J, a User‐Friendly Browser Application for Genome Assembly, emm‐Typing, MLST Typing, and Virulence Factor Gene Detection of Streptococcus pyogenes
Source: Microbiol Immunol. 2025 Apr 20;69(7):384–8. doi: 10.1111/1348-0421.13223 (PMC12232109; doi:10.1111/1348-0421.13223)
Supplement: Supplementary file 1 — Supplemental_Dataset1_contig. [file MIM-69-384-s003.docx]

>18S-48_L8_1_1

CAACTACTTTTTCATCTTTTTTCAATAAGTGTGTTACAGGAACCTCTCTGACAGCTTTAT

TAGTATACGATATCTTTACTGTCATTGTCAACTCTTTTATACAAAATGTTTCTGTTTTTC

AATTATATTACTGCTAAATAACAAATTTTATTGTGTAACAAAAATGAACTAATTTTCTTT

TTTGAAATTTATATCTATATATTTAGTATTAAAATCGTTTTAGAGTCTATGTCATTGTGC

CAATCAACCCCTAGAGACTAATCAATTCTCTAGGGGTTGGGTAATTTTAAATATTACAAC

TTTGATAAGTAAGTAGGTATTGATTTTACTACTCATTTACTTCTAAACATTATAGTCTAT

AATGTAATGTCAAATCTTATTATCAAAACATTATTTATTTTCTATTGAATGAGTATAACC

ATAAGCTAAGTAGATTAATGTTCCAATAATTGTGGCAATAGCAAAAGCAATCCAAGTAAA

AGCTTTATATTGGCTCATAAATGATAGACAAATGATAATTGCCAATATTGGTAGGAATGG

TACTAGAGGAGTTTTAAATTCATTTGCTTTTGGTTCTCCTTCTATTCTTCTGAGTTTAAT

AATTGCACCCGACAGAATAATTAAATATGCTAAAGTACAAATATTAACAAACTCTGCTAG

ACTCGATAAAGGAAATATTCCCGCACAAATCATAGCAAGTAGTCCAGTGACAAGAGTAGC

ATTTTGAGGCACTTTATTTTTTTTAGTGAGAGTATAAAGTGATTTAGGTAATAAACCATC

TCTACTAATACTGTAAATTGTTCTAGCTAATGCATAAGTCATAGAAATACATACCGTAAT

AAGAGTCAAAATAGCAACTATCGACACATAGTCAGCTGCCCAATAAAGTCTAATATTTCT

TAAAGCAAAAGCCACCGCGTCTGGAACATTTAATTTAGTGTAGTGTACAATTCCCGTTAA

AATAGTGGTGACAACTATATAGAGTGCTGTTACAATAATCAAAGATAAAATGATACCTTT

AGGAATTGTTTTTTGTGGTTCTTTCACTTCATCAACTGCCATTGAAATAGATTCAAATCC

TAAAAATGCAAAGAACATAACAGAAGCACCAGCAAAGATACCTGTTTTACCTCCAGTAAT

TTGTCCTACACCATATGGAGCAAAATGAGACCAATTATTGTGATCAATAAAAAAAATGCC

TACAAAGATAAATAAAGCCAGTGCTGAAAATTTTAAAATAACTAAAAAACTATTGAATCT

CAGAGCTAATTTCGAATTCATTAGGACAATCCCTGTCACGAAAAACATCACTAAAACAGG

AAGAATATCTATGTAAGTTCCATTCTTAAGATTGAACGTGCCATTTAAAGCGTTTGGTAA

TACTATATTATAATTGGCAAGTAATCCTTTTAAATAACTTCCCCAGCCAACAGCAACACT

AGATATAGCTGTTAAAAATTCCATAATAATATACCAGCCAACAAGCCATGCTGGAAACTC

TCCCAAGGTTGCATAAACATAACTATAAGCACCACCGTTAGAAGGCATACGAGACGCAAA

TTCTGCATAAAATAAGGCTAAAATTCCAATAGTAATCGCGGAAATGATAATCGATATTGT

TAAAGCAGGACCTGCATACTTAGCAGCGCCTATGCCAGTAACTGTAAAGATACCTGTTCC

AACCATTGAACCTAGGCCTAAAAAAACTAAATCAATAACTTTGAAGTGCCTTTGCATTTC

TGTTTTATTGGAATATTTCTTTTTTTTACGAAAAATAGTCATGTTATCACCTTCTATAGT

AAATGATTTTCTACTAAGTGTATCATAGTTAGTAATGATTGTAAATTTCATCTTATTGGT

TGACTGAAACTTTTGCTATCTTCTGTTCTTCTATTATTCTAAGAAAACACTAATAGATTT

TTTCAAATGGCTTTAACAATTTTCTAGTTTTAAATATCAAAGTCTTTCTCACTTTAAGTA

ATAAAGAAAAAAAAGTATGTCATCTTCTCATTTCTAGCAATTAGGCACCTATTACAATTC

ATAACGTTTACAAGGATATTGTGTTGATAAAAAAGCTGAACTTAATCTATAAGTTCAGCT

TTTTTATATCATTCTGCAGATTCAGATTCTGTCATTTTATTTTTAATTTCGTCATAAGAT

AACGCATGAGCTTCATTATCATCAGTTTCTAAATCAACTGGCATTTTACCAGTTTCATAA

ATTGATTTAATTTGTGCAGCGTCTAGAGTTTCATATTTGAGAAGAGCTTCAGCAATCAAT

TTATGAGTATCTCGATTCTCATTGATGATATCAGCTGCTTGATTACGAGCTTGATTTAAG

AGTTCACGAACCTCATCATCAATCATTTGAGCTGTTTGGGCAGAGTAAGCTTTTTCAGGA

GAGATCTGTCCTGGCATCATCGCATGGTTACCTTCATATTGAACTGGTCCAAGCTTTTCA

CTCATACCATATTCTGTAACCATGGCCCTTGCTATCTGAGTAGCTTGCTCAAAGTCATTT

GACGCACCTGATGTTTGAGCATTAAAAACAATTTCTTCAGCAACACGACCACCCATCAAT

CCTGCTAATTGCTCTTTCAGATCTTCTTTAGAAAGAAGCATTTGATCTTCTTTTGGAAGA

GCGATCATATAACCACCTGCTCGTCCACGAGGTACAATAGTTACCTTGTGAACTACACGC

GCATTAGATAAAACTAAACCAACAATAGTATGACCAGCTTCGTGATAAGCAACCATTTCT

CGCTCTTTTTGCGAAATAGTGCGATCTTTCTTTGATGGTCCAGCAATAACACGATCTTCA

GCTTCATCAATATCACTAGCATCAATTTTAATTTTATTTCTGCGAGCAGCAACTAACGCA

GCTTCATTTAAAACATTTTCAAGATCAGCACCTACAAAACCTGGAGTCTGTTGGGCTACT

ACTTTCAAATTAACGTCATTTGCAAGTGGTTTATTTTTAGCATGAACACGTAAAATGGCT

TCACGTCCTTTAACATCAGGGCGACCAACTAAAACCTTACGATCAAAACGACCTGGTCTT

AGTAAAGCTGGATCTAAAACATCACTACGGTTAGTTGCAGCAATAACGATAATGTTCTCA

TTACCCTCAAAACCATCCATTTCAATGAGTAATTGGTTAAGGGTTTGTTCTCGTTCATCG

TTACCGCCTCCCATGCCAGCACCACGACGACGACCTACAGCGTCAATTTCATCGATAAAG

ATGATCGCACGCTCAGCTTTTTTAGCATCCTCAAATAAAGAACGAACACGACTAGCACCT

ACACCGACAAACATTTCGACAAAATCTGAACCTGAAATACTGAAAAATGGCACTCCAGCT

TCACCTGCTACAGCTTTAGCTAGTAATGTTTTACCAGTACCTGGAGGCCCCTCTAGAAGA

ACTCCCGCTGGAATCCGTGCTCCTAGTGATTTATATTTTTTAGGATTTTTCAAAAAATCA

ACAACTTCTACTAATTCTTGTTTTTCTTCTTCGGCACCAGCAACGTCTGTAAATCTCACT

TTAACATCACCTTTTGATTGAGATTTTGCCTTATTTTTGCCGAAACTCATAGCACCACGA

GCTCCTCCACCACCTTGATTCATCATCATCATCATGAAAGCAGCAAAAATAACAATAGGT

AAAAAGCTCATTAAGAAGGTAATCCAAGTACCACTTGAACTTTCCTGCTTAACTGTAAGC

TCTGTTCCATTTTTATCAGCTGCTGCTGTCATTTCTTTCAAGATGGTATCACTAGGTAAT

ACTAAAGATGAGAACTCTGTTACCTGTGTTGAAGCTCTTCCACCTAAAAATGATAGTCCT

GAATTAACTGTCACTTTTTGAGGTTTCTCATATTTCCCTTTAACTTCAATAATACTACCG

CTAGGCTGATAACTTAAAGATTTTATATCTCCAGCCTTAAGATGTTTAATTAATTTGGAA

TAACTAATCTGTTGGCTTTGTGTGCTGGTTCCCTTAAGATAAAATTGAAATCCTGTGATG

ACAACAATAATCATCAATATATATATAAAAGAATTTTTAACAAAACCATTATTTTTATTA

TTTTTCATACAGTAAACATTGACCTTTTTCTATTTTGAATAGACTTCTTCTTTTAATACA

CCTACGTAAGGAAGATTGCGATAATTTTCTGCGTAATCTAAACCAAAACCTACGATAAAT

TCATTTGGAATATTGTAACAAACATAATCTGCTTCAATTTTAACAACACGACCTTCAGGT

TTATCAAACAGTGTTGCAATTTTGATAGTATTAGCTTTACGGTACTTAAACATATCTCGT

AAATATTTTAAGGTGCGACCGGTGTCAATAATATCTTCAACAATTATGATATCTCTTCCT

TCAATATTGGTATCTACATCTTTTAAAATTTTAACTTCACCACTGCTAGATGTACCGCCG

TGATAACTTGATACTACCATAAAATCAATTTCAACATGAGTATCGATGTGTTTCATTAGT

TCTGCCATAAAAGGAACTGATCCTTTTAATACACCAATCATAAGCGGATTTTTTTCTTGA

TAATCTTTTGTTAATTGTTCTCCTAATTTTTTGGTTTTCCGAATAATATCATTTTCAGAG

TAAAGAATTTTTTGAATATCTTGCTCGAGCATGCTGTCACCCTTTTTCTAATTTATCAAT

ATACAGTTTAGCCAACATTATATCATGTTTACAAGCTTTTCTCAAATAAGTTTTATTACC

TATCAATACAAATATAATCTGCTCATCTTGCTCTCCAATAATAGCATTTTGACGTTCAGC

TATTGTGAATTTTTCATCTATAAATAAACGTCTTATTTTTTTTGAAAAGTGACCAAAAGA

AATATAATCTCCAGATTGGCGACTTCTTAATTTAATAGACGATAAACTAAATAAAGGTAT

ACTTACCTGTCCTTGATCTTCGTTACTTTTTGGCATAAATGAAAAATAATAATCTCTGTA

GCATAAGTTACTATCGTATTCTAACATTTTTTCTTCTTTAACTAACTCAGTCTTAGGAAC

GATTTTTGTAATTTTAAAAGAGAATTTATCAATGAATATATAATAGTCTTTTTTGAGATA

ATAATAGCCTTGTTTAGCAGTTTGGATAATTTGCAATAATTGAGTAAATTGAGATTTTTT

TAAATCTAAATCTGGAAAACCTTCTAAATAATCTTGTAACAAAAAGTATTGAATAGACTT

AGATTGCTCATTAAATTCTGTTAAATCAGTGGTAGTAATATGATTTGTTAGCTCCTTAAA

AGCTTGAAAAAGTAAGCTATTTTCAAGTGCTAATTGATTTAATCCCTGAATGAACCTAGG

ATTTTCCTGCTTTAACAATGGTAAATAATTATTTCTAACTCTATTTCTTAAAAATGATAA

TTCCCTATTTGAAGAATCTTCAAAATGAAAAATTTCTGGCAAGTCCTTTTTTGAGAAAGT

TAAAAAAGGTCTTATAAGTTGACCATTGGCAAATGGTTGCACGCTTTTTATACCCGACAA

GTGCCTCAATCGGCTTCCTCTAATTAGCCTCATCAAAATAGTTTCTGCTTGATCATCTGA

ATGGTGAGCTGTTACTAAAGCTGAATAGTTATTTTTTAACATAATACTTTTAAAAAAAGC

ATAGCGCCAATCACGTGCTGCTTTTTCTGAAAATATTCCTTCAAAATTAGAAACATAAAT

AGGTATGTCATGTTTTTTAGCCCAACACTTTAAATAAGCTTCTTCTGAATCTGATTCTGA

TCTTTGTTTGTGATTAACATGAGCAATACCAATACGAATTTTCAATTTGTCTTGAAACAG

GTATAAAAAGTGCAATAAATTCATTGAATCTACTCCACCAGATACGGCTATTAAAACATG

TCTATGATTTTTAAAGTAGGCTTTATTTTTTATTTCATTAAAAATTTCTTGATAGGTCAT

CATTTTAAAATACCATAAACGTCATCTGCAATAGCCGTAATATCTTCATAGGTTGATTTA

TTTGTAAAAATAGACAAAATAAATGGAGTATTACCGTAAACAATAGCAACGTCATGTTTA

TAATCATAAGCATCACCAATTTTATGTGCAACTGGAACAGTAATATTTTTTGTGATACGT

TGTTGATCAAATTCGGTATTTGAAAGGTAAGAAATAATTTGGCCTTTTTGATGATAAATA

GCTTCCATCATATTTGCAGCTGAACGAGAAGTTAATAATCGCTGCTCCATATCCCAATCG

ATACCTGATAAAGCTTTTATCTCTGAGCGGAAAGCTTTATCATACTGATGACATAGATAA

TAACCTAAAATATTAGTTGCTACATTATCCGATTGTTGTGCTACAGCTTTCAGTAGGTCT

TCAACACGATAATCTTTATTATCAGCTATTTTACTAATTTTACCACTTCCCAATGGATCA

TAGTCTCCATAAAAATGATTAACATCTTTAGTATAAGTCAAAGTTTTATTCTCTGCTAAT

TTCTTCTTTTGTAATTGTTTTTGAACAATATAAAGTGGTGCTAACTTCGAGATACTTGCA

GCATACATTTTTTTATCAGCATTAATACCAGCACTTGTTTGTGTGTTGAGTTGCTTTACA

AAAATTGAATAATTTGGGTTATTATATTTTTGTAAGAGCATTTCCTGGACTTTTAACATG

CGGTTATCAAATTGAACTAGATCTTCTTGTGATGCCCAGCCCTTATCATCAATAAGATAA

TAAGTTCCATGGTTTGTTTGTGCCATTTGAGTGGCATGAACTTTTTGAGCAAATGAAAAA

GAAGAAGGAATTGTTTGCGTACCTAAAACATAAGGGGCTGAATAAAGCCTAAGTTTCTTT

TGTGTCCAAAAATAGTTATCTATATCTACTTGATTAAGTACAATATCGTCATAAATCAAT

TGTCGATTAGCCTCAACATAGGTACCATCAGCTAACTCAAAAACCGGTAAAGATTCTTTG

TTAAGCACAAGCGATTTAATAGGTAATGGTTGATTTACACCTAATCTTCCTGCTGGTAGA

GTTAAATCACTGTCTTTATAAGCACACGTTTCTTGATAAAGATTTGGATTAGAGGGTAGT

TGATTATAGAATTGTGTTGATTGAACGACATCTTGTTTCAATTGATAAACAGCATTTTTT

GAAAATATAAGTTTTTTTTCTGTACTAATCACTGGTAAAGGAGTCAGAAAAAAAGTCATT

AACATAGCCACTAATAATTTTCTCATATTGACTCCTTTCTAGTTTTTTTGAATATACTGA

CTTTCTTCTTTAACTTTTTGATTTTCCTGTTGCAGCTCTGCTAATTTTTTATCAGAAAAA

GCAAGAAATGCTTCTATTTTTTTTACAATATTGTCCATCATCATTTTGGTAATAGTCCTG

GAATAGGATAAATCATTTCTCCTTCACGCGATAAATAGTATTTTGCCCTAGCATATTTTT

TGACAAAATTATCATCTTTTAGTCGTTCTGCTAATTGTTTTTCTTTTTTTGTACTCTTTG

ACAATTCATTATACTCTTTTTTTAATTTAACCACCTGTTGATTTTGCTTTTCAAAATCAA

CATAACTTTTGACAAGATTATAAGTTGGTAAAATAAATAAAAACATCATACTTACAAGGA

TCCATCCCATAAAACGATTTCTTTTTTGAGATTCTTCTTCTTCAAATTTTTTTTTGAGAT

TCTCTTTCTTAATATAATGATTATTTAATTGAACAATACTAGGCTTCTTCATTTAATGTT

ATCCTTGTTTCACTGATTATCTCATACATCTTAAGAGCATCTTCTTTTTTTGTACTATCT

TTTATTTCAATGACTCTTACTGTCAGTAATTTATTTCCAAAACTAATTTCAATGTGATCA

TTTAGTTTTACATTCGTTGAACTTTTAGCAAGTATCCCATTAACTTTAATTCGTCCCTTA

TCCGCAACTTCTTTTGCTACTGAACGACGTTTAATAAGGCGCGATACCTTTAGATATTTA

TCTAGTCTCATAATTTCTCCTACAATCCTCTTCATTTTATCATTTTTATTTTTTAAATGG

TAGAAAAGATCCTAATTTCGCAGCAAAGATCTTAGATTTTTGACAAAAGTATAAAGAGCT

AGTTCGTTCCATTGTCATTAATACTATTCAGTCTAAAAGTTGTTTCAAATTATTATTTAA

AAACTGAATTGTTTTTTCTGATTTTTATCTCACTAAGCCTTTCTCCAAATAACATCAATT

CTTCTAAAATTTTATAATCTTTTTGATGGCGAACGTCAAAGACGATATCGATTTTCCCCT

GATGTTCACTAATTTTAGCTTTAAGATGGGTTTTCGATAAGGCTTCGAAATAATCCTGAG

TTAAGAAATAAGTTAAAGAAGTTACTTCAAATCGAACAATGACCTGATTATTTTTGCGTT

CAACTAATTCCGCAAAGGCATTGTCCATATAATGTTTTAGCAGAGCCATCTCTAACAAAT

AGGCAACTTGATCAGGATACTCTCCAAAACGATCTATCAGCTCATCTTGCAAATTGAGAT

AATCTTCTCTTGATTGAATTTCTCGAATACGCTTGTAAATGTCAATTTTTTGGCGCTCAT

CTGCAATATAATCATCTGGTAAATAAGCATCAATCTGAAGATTGATTTCAGTATTACCTT

TTTGGCGAATAGTCGTTTTTCCTTGCTTGCTAGCAATAGCCTGTTCTAATAATTGAGAAT

ACATTTCAAAACCGACCGAATCAATAAAGCCACTTTGAGAAGCTCCTAAAATATTACCTG

CTCCTCGTATAGACAAATCTCGCATAGCAATCTTGAAGCCTGAACCTAATTCAGTAAAGC

CTTTAATAGCCTCTAAACGTTTTTCAGAGACTTCTGTTAGGATCTTATCAGGACGATACA

TCAGGTAAGCATAGGCAATACGATTACTTCTTCCGACGCGCCCCCTCAGTTGATATAAAG

TTGACAATCCCATATGATCAGCATTCTCAATAAACAAAGTATTTACATTAGAAATGTCAA

CTCCTGTTTCGATGATTGTTGTAGCCACAAGGACATCATAATCACCATTTATAAAGTCAA

TCAAGGTATTTTCAAGTTGAATTTCACTCATTTGCCCATGAACAAAACCGATAGAAGCTT

CTGGGACTAATTCTTGTAGCTCTGCAACTTTCTTTTCAATAGTGTCAACTTTATTGTAAA

CGTAAAAAATTTGTCCTCCACGATCCATTTCACGAATGATAGCTTCTCTAACGAGACCTG

GATTATTTTCCAAAACATAGGTTTGAACAGGATAACGATTGGTTGGTGGGGTCTCAATAA

CCGATAAATCTCGGATACCTAACATAGACATGTGTAAGGTTCTAGGAATCGGAGTAGCTG

TTAAGGTTAAGACGTCAACCTTAGTTTTTAATTCCTTTAAAGTTTCTTTATGCTTAACAC

CAAACCGTTGTTCCTCATCAATCACAATTAATCCTAAATCAGAAAAGACCACATCTTTTG

ATAGGAGTCGATGGGTTCCGATAATAATGTCAATTTGACCTTTTCGTACACGTTCTAGTG

TTTCAGCTTGCTCTTTTTTACTACGGAAACGACTTAAGACGTCAACCTCAACAGGGTAAT

TTTCAAAGCGTGCTTTGAAGTTTTCATAATGCTGCTGGGCCAAGACTGTGGTTGGAACTA

AGACAGCTACTTGTTTGTGATCATTCACCGCTTTAAAGGCTGCCCTCATAGCTACTTCTG

TCTTACCAAATCCTACATCGCCTACTAAAAGACGATCCATAGGTTGCATGCTCTCCATAT

CAGCTTTAATTTCCTTTATAGACCTAAGTTGATCTTCTGTTTCTACAAAAGCAAAATCAT

CATCAAAAGCGCGCTGCAAGTCATCATCTGGTGAAAATGAAAATCCTTTTTGCTGACTTC

TTTCAGCATATAATTTTAGAAGGTCATCAGCAATATCCTCTACTTGTCTAGCGACCTTTT

GCTTTGTCTTTTGAAAACGACCATCATTGAGTTTATTAATTTTAGGTTCTTTTCCATCAG

CAGAAACGTACTTTGATAAGCTACCAATTTGGTCAATTGGAAGAGAAATACGGTCTGAAT

TTTGATATTGAATAGTAACGTAATCTCGATGGATTCCCTGAATCTGAATCGTTTCAATTC

CAAGAAAGCGACCGATACCATGGACATTATGAACCACGTAATCACCTACTGCTAACTCAT

TGTAATCTTTCAAACGCTCAGCATTGCTAATATTAGATCGTCTAGCGCGTCGTTTGATCT

TTTTATGATAAATTTCATGCTCTGTGATCAACGCTAACTTTTCATCAGCAAAATAAAAAC

CACTGGAGATAGCTCCAATTACAATTTGTGATTCACGTGAAACAATTTGATTAGCACTCA

CTAAAGGAAGGCGAAATTGGTAATCTTGAAAAGATTTTTCAAGTCGTTCGTAAGCATACT

GAGACTCTACCTGTACAATAACCGTTGTTTGATTTTTTTGATACCGTTTAATCTCATCAA

TCAACAAAGGAAATTGATTAAAAAATTCCTGCATGGCATACTGAGTTAGCTGATGCATCT

GATCAAATTTGATATTTCCAAGTCCCTTATGAAAATTTGAAAAGAAGGTCGCTGGCTTAT

AGTGCCTAAGCTCTCGATAATTATCTGCAAAGTAGTTAAGATTGGAAAGAGCCTTTCCTT

GCTGTAAATCTTCTGTCAAGAGATTAGCAATCTCTAAATCAAATCTTGCATTTTTATCAA

CTAGTTTTTGAAAATCATCAAAAAAGATTGGCGTTCCCTTAGGAATATAATCTAATAATG

ACCACTCTTTTTCATAAAATAATGATTGAAATTTACGGATATCCTTATGTTTAAAACCGT

TTTTTGAAACAGCTAATACATCTTCTAAATAAGATTTTTTATCATCTTGTGCTGTTTGTA

GAGCCTTTTCTAATTGCTCAATGCCACGTTGAAAATCACTAGCCTCAAAAATAAGATCAC

TTGCTGGATTAATAAAAATACCTTCTAGTTGTTCAAAAGATTTTTGAGTTTCTGGATGAA

ATTGCCTAATACTATCAATATCATCGCCAAAAAATTCCAATCGATAAGGCAATTCTTGTG

TAATCTCATAGATATCTAAAATATCCCCTCGACGGCTAAATTCTCCCGGACTAATAACCT

GTGAGACCTTCTGATAGCCAATTGTCATCAGTTGTTTAGTAAAAGTATCACTATCATAAT

CTTCTCCAACTGTTAGTTGAATCTGACTCTTTGTAAAAACATCTGGGTTTGGCAATAAAG

TTCTTAAGCCTGATAAACTAACAATTAAAACGCCCTGAGATTTAGGATTCCTTAAAAATT

GCAGGGTTTCTATTCTTGATAGAGCTTTATCCATTGACGCAAAGATAAATTCCGCTGCAG

CCACATCGTCTGCAAAAAATTGGAAAACAAGTTCTTCATCAAGTAAACTAGATAAATCGC

TGGCTAATTTTTCAACCTCATTTTGAGTTGATGTAACCACAACTATTTTTTTTTGATCAT

CTAAATAAGCGGAAGCTATAGCCAATGCTTTACTTGAACCCGATAACCCCATTACCAGTT

GTCTTCCTAAGGTGGTTAATCCAGAGTGCCAGGATTGGACTTTCTTATTCTGACTAAATA

ATTCTAAAATATCCATGATTATCCATTAAATTTCTGCATTGTTTTTTCAAAATCATTTTC

TTGTAAATAAAACTTGACAGCATTGACAACTCTGTCAAGAGTTAACGAAATAGCAATATT

ATCTTCGGTATTGAATTGGCCCATCACATGGCTAATAACAGTCATACCTTTTAAAGGTCG

TCCAATACCAACTTTGATTCGGTTAAATTCCTGAGTTCCAATGTGGGCAATGATTGACTT

AATGCCATTATGTCCTCCTGCCGAACCCTTACTACGTAAACGTAATTTACTGACTTCCAT

GTCTAAATCATCATAGATAACAATTAAATCTGTTATGTCAATATTATAGTAGGTTAGTAA

TGCTTTTACTGCTATGCCGCTATTATTCATAAAAGTAGTAGGTTTCACAAAGTAAACTTT

TTCATGATTAATAAAAGTACTTCCTATCTGTGCTTTAAAATTTTTATCATCTGTAAAGGT

AACGTCAAGGTTCTTGACAATATTGTCAATAGCCATAAAACCAATATTGTGCTTTGTTTT

TTCATATTTAGAGCCTGGATTTCCCAGACCAACAATCATTTTTACCATATTTTCTCTTTC

TAAAATGCCAAAAGGGCTGGAATGTTCTTCCAACCTTCTGATATTGTTTTAATATAATTA

CACATTAAATCTGAATTCCATGATGTCCCCATCTTGAACAACGTATTCTTTTCCTTCTTC

ACGCAAGCGTCCAGCTTCTTTGACGGCTTTTTCTGAACCGTAGATCATTAGATCATCATA

AGACATGGTTACTGCACGAATAAAACCTCTTTCAAAATCTGAATGGATAATACCAGCAGC

TTGTGGAGCTTTTATACCACGCTTAAACGTCCAAGCACGAACCTCTTTTTCACCTGCTGT

AAAATAGGTTCCAAGGCCTAAGAGATGATAAGCTGCTCTGGTTAATTTATCAACGCCTGA

TTCAGTAAGACCGATAGCTTCCAAAAATTCTTCTTTATCCTCATCGTCAAGCTCTGAAAT

TTCTTCTTCTGCACGCGCTGAGATAACAACTACTTCAGCATTTTCAGTAGCTGCAAAGTC

ACGAATTTGTTTGACATAATCAATACCATCTGGATTAGCAACTTTATCTTCATCGACATT

AGCTACATATAAAACAGGTTTAGTTGTTAATAAAAAGAGACCTTTAACAACTTTTGCTTC

CTCTTCTGTAAACTCAATTGTCCTAGCTGATTTCCCATCTTCCAAAACAGGTTTAATCTT

TTGTAAAACATTGAACTCTGCTACTGATTCTTTATCTTTTTGAGTTCGTGCCATTTTTTC

AACACGCGCATAACGTTTATTGATTGACTCTAAATCAGCTAAGATTAATTCAAGATTAAT

AGTGTCAATATCTGCTATAGGATCAACGAAAGCATCCTCACGACCTTGTTCACGCATGAC

ATTTTCATCATCAAAAGCACGTACCACATGAACAATAGCATCGACTTCACGAATATTAGC

TAAAAATTTATTTCCTAGACCTTCCCCTCTAGAAGCACCTTTAACGATACCTGCAATATC

AGTAAATTCAAAGGTTGTCGGAACTGTTTTTTTAGGTGTAATCAATTCTGTCAATTTTTG

CAGACGCTCATCTGGAACCTCTACCATACCAACATTGGGATCAATAGTCGCAAAAGGATA

ATTAGCAGCTTCTGCTCCTGCTTTTGTAATTGCGTTAAATAAGGTTGATTTACCAACATT

TGGTAAGCCTACAATACCTGCTGTTAAAGCCATTTAGCGAATCTCCATTCAAATTTCAAT

CAAGATTATTATACCAAAAAATAGAAGAAAAAGCGGTATGAATAAGAAGAAAATTTCAGA

TTTGCTTATCATTATGCCACTCTTTCACCTTCATTTAAATTATCAAAGTTTGGTGTATTA

TTCTCATTTTGATATAGTTTGCCGAGAAACTAAGTAACTGTGCTACAACTTTCGTCATTG

TCAATAGCCACCTTTCTTCTTTCGATAACATTAGAATAGCTAATTTTCCAACGGCATACT

AACAATTTTTTAGGAAAGTATTGGTTGCAAATATAGCTAGAATTATTTATTTAAAAGGAC

TTTCATGGTTGCAGTACTTTTTTTAATTTTCGCTCAAAATCATAACGACTCATCATAATC

ACATGCTGACAGTTAGTACACTGAATTTTAATATCAGCTCCTACTCTCAGTACCTTCCAT

TGATTAGCCTTTTTACCAGTTTCTTTAATTACACAAGCATGAGGTTTTTTCATTTCAACA

AATGATCCAATTTGATACATTCCCTTTTCTCCACTTATATTATCGTTAATATTTATAATA

ACAAAAAAACTCCTAAAAGGGAGCTTTTTCTTACTTAGTTTGTTCGTACTGGTGTAATTA

ATTGGATAAAACTTTCTTCCTCATCACCTGGTGTTAGGGTGAATGGTCGAACTGGTGATA

AGAAATGAATTTTTACTGTTTCACTTTTAATAGCTTTTAAAGACTCAATAAGGTAAGTTG

GATTGAAGCTGATAGTTAAATCACTACCAGACTGACTAACAATATCTAAATCCTCGTTTA

CCTTACCAACCTCAGGTGAGTTAACATGAGCTGAAATATGATTTTGAGTAATCTCAAGCT

TAACAGTACCATTTTGAGTAGCATTAGAAATCAAGAAGGCACGTTCCATAGCGTGGCGAA

GGGATTGGGTATTGAAAACAACCTCCGTCTCAAATTCTGTCATTAATAAACGGTCTGTAT

CGGGATAATTTCCTTCTAAGAGACGTGTATAGAAAGAAATATGTTCACTTCTGAACAAGA

TTTGGCTTGGTGAGAAAAATACCTCAACGGTCTCAATATCATCTGTAAATACTGCTGAAA

ATTCTCTCAAAGATTTACTTGGAATAACCACATCAAAATCTGCTGAAGTATTGTCCAAAG

TGATTAAACGTTGGCTCATACGATGAGAGTCAGTCGCTACTGCTTTAAAATCTTTATGAT

TACTTAATACAATATGAACTCCTGTTAAAATAGGACGACTTTCTTGTAAACTGGCTGCAA

AAGCTGTTTCAGCAATAATAGACTTCAATAATTTTGTTTTTAAAATCAAAGGATTTTCTG

TTGATACTTCTTGTAGACGAGGATACTGGTCAACATCTTTTCCTTTTAAGGTAATCTCTG

ATTTACCACTGGTTAAAACAACTTGGTGTTGTTCAATTTCTTTAACATTTATACTAATAT

CTGGCAAACTTGAAATAATATTAATAAAAAAACTAGCTTCTAATAAAATAGCTCCTGGAG

AGGTAATTAGCAAACCAGCATTTTCATTACTTACAGGAATAGTGTTTTCAATTGATATTT

GACCGTTAGACCCTGTTAAAGTTACTCCAGTAGGAGTGACTTCAATTTTTATTGATGAAA

GAATAGGAATGGCATTTTTAGTGCTAATAGCACGTTTAGTTGCATTTAAAGCCTGAATAA

ATAATGTGCGATTAATTGAAAATTGAATCATGAGAACTCCTTTATTTAATAAGTATAATA

ATAAGTAGTAGTAGTAGGTCTTGTGTATATAGTGGATAACTCTAAATAAACGAGAGAATG

CAAGACTAAATGGTTGTTCACAACATGTGGATAACTATTTCATAAAAGATATTCTTTTCC

ACATGTTATTTAATTTTGTTTTTTATGGTTTCAATTTCGATCCTAAGGCTTTCGTCCTGG

CTGATCATGTTTTTGATTTTATTATAGGCATGGAGTACTGTTGAATGGTCTCTGCCACCA

AATTCTTTTCCAATTTTAGGAAGACTGTTATCTGTCATTTCACGTGCTAAAAACATAGCT

ACTTGTCTTGCTAAAACAATATTTTGTGTTCGTTTAGTAGCTTTAATTTCTTTGACGGTA

ACACCGTAAAATTTTCCAACTTGCGCTTGAATTTCTTCGATGGGAATAACTGTCATTTTA

GGTCCATCTTGCTTTCTGGCGCGAATAGCTTCGGCAGCAATGTCAACAGTAATCGTGTCA

ATTTGTTTGAAATTAGCAACCAGACTAATATCTTTTAAGGCACCTTCTAAATCTCTGACA

TTAGAATCAAATTGACCAGCCAAATACTCAATGGTATCTTGAGGAAAAATAAAGTTATAT

TCTTGAATTTTATTTGTCAAAATAGCCACTCGTGTTTCAAAATCAGGAGGTGTGATATTG

ACTGTTAATCCCCATTTAAAACGAGTAACTAATCGATCTTCTAAATCATTGAGATGATCT

GGTGTACGGTCGCTTGTTAGGACAATTTGTTTGTTATTATTATGAAGTGCATTAAAAGTA

TTAAAGAACTCTTCTTGTGTTCCAGAGAGCGTTTTTTTAGCTAAAGATTGGATATCATCA

ATAAGGAGTAAATCTAAATTACGAAATTTTTCTTTCAATTCATCCATGGTATCAAGGCGA

ATATGGATAACAAACTCATTAATAAAGTTTTCAGCTGTGATATATTTAATTCGAGCATTT

GGATTTTCTAATAGTACAGAATTACCAATAGCATTTAATAAATGGGTTTTTCCAAGCCCA

GGGCCACCCCAAATAAACAAAGGATTATAGGTAGTTCCAGGAGTATTAGCTACTGCTATT

GAAGCAGCAACAGCCCAACGATTTTCATCTCCTTGAATAAAGTTTTCAAAACTATATTTC

GGGTTTAAATCTGAAGTAACAGTAGGCAAAGAATTTAAGGCTTGCTGCTTAGGTTTTTGA

TTGATTTTGGTCTGATTTTGCTCAATCATTAGGTCTTCTTCGAAAACATAGTCAACAGAA

ATTTGAGCGTTATAAACTTCAAAACCAGCAGTAAGAATAACATCTTTAAGATTTTTTTCC

CAAAAGAGTTCTTTCATTTGATCTAAGTAAATAGTTGCAATATGCTTATCGACCTTTAAT

AGACGGGCATCATGAACAAAAAATTCATAAGTTGCCTGTTTTAATTGACTCTGAGCTAAT

TCCAAGACCCTGTTCCAAAAAATTTGTTCATTTTCAGTCATACTAGGTTCCTTCTTGTGG

ATAATTCTAGAACTATTCTACCATAAACTATTCTAGTTTTCCACAGAACAATGGATAATA

GTTAGTTTTTCCACAATCTGTGGAAAATAAATTAACACTGTTAACTTGAGTTCTCCACAA

CCTGTGGAAAAGACATTTTAAGGTGACACCGCTATTGTTTTCAACTATTTTTCATGTGGA

AAACTAAAAAAGAAAAAAAAGAATATCAACAAGCTACTTTAGCTTGTTGATAATTCTGTT

TAATTCATCATCATTGGAAAAAGATATCTGAAGGTACCCACTATGGTTTGCTGTCAGCTT

CATATTGACAGAGAGTCCCAATGATTTAGCCAATTGTTTCTCTAAATAAGTGGCAAATAT

ATTTTTAGTTTTTTTAGATAGCTTCGAACTTGGAGTAGAAGTAACCAGTTGCTCAATTTG

CCTAACACTTAGGCCTTCATTTTGTATTTTATGAGTGAGGTACAGTTGTTGCTTATCATC

ACTCAAAGTTAAAAGAGCACGCGCGTGTCCCGCACTAATTTTTCCTTCTTCAATTGCTTT

AATGATAGGTGCTGGGAGTTGTAACAGACGTAAGGTATTGCTAATATAAGGTCTTGATTT

TCCCATATATTTAGCAATCTCATCGTGAGTCATGTGTTTTTTTTCAACCAATAACTGATA

GGCTTTAGCTTCTTCGATAGCGTTAAGGTTAGAACGTTGTAAATTTTCAACTATAGCTTG

TTGCATGCTCTCGAGTGTAGAGATCTTCTTGATGATAGCTGGAACTTTTTTTAGACCAGC

CATTTTTGAAGCTTTAAGTCGTCGTTCTCCAGCAACTAACTCATAGCCAAAAATGTCAGA

TTTTCGAACAATAATAGGTTGAATGAGGCCGTTTGATTTAATAGAAGTTGCTAGGTCTTG

GAGTTCTCTTTGATTAAATTGTATCCTTGGCTGATAGGGATTTGTAACAATATCTTCAAT

TGGTAGGTCTATCAATAATTCTTTTGTCATAAGGGAGTTCCTGGATATAATAAGAAACAT

AAAACAGTCCTTTGGACTGTTTTATGTGATAAAATGTAATAGTATTTAGGTAGCTTGTTT

ACTGCGTTTTAGTCAAATCTTGAGTAGTTTTTGTTAATTTGATATCTGCTTTTTTCTTTG

TAGTTCCTCTATAAAAAGTTACTTTAATAGTATCATTAATATCATGTCCGTATAGACTAC

TTTGTAAATCACTAATTGAATTAACTGTTTTGCCATCAATTTCTGTAATCACATCGTACT

GAGCAAGTTTGCCAGATGCCGGCATTCCTTCCTTAACTTCTGCTACTACAATACCACCAG

TTACACTAGTTGGAATATTAATTTGGCTAAGAGCATTTGTGGATAAGTCATTTAAATTGA

CCATGGATATTCCAAGAGCAGGTCTGATAACTTCACCATTAGTTTCTAGTTGTTTAATAA

TTTTAATAACATCAGTAGATGGGATAGCAAAACCAATTCCTTCAACAGCACCACTATTAC

CGTTGCTACCAGTTGGGGTAGAAGAAATTTTACTTGAATTAATTCCGATTACTTGCCCCT

CAATATTAATTAGTGGTCCACCAGAGTTTCCAGGGTTAATAGCAGCATCTGTCTGAATAG

CATTTGTTGAGACAGTCTCACCATTCTCGTTTTTTAAAGTTACAGTACGACTTAAACTAG

AGACGATTCCTTGAGTAACAGAATTAGCGTATTGTGTTCCTAGTGGGCTGCCGATAGCAA

TAGCAACTTCTCCAACATTTAGTTTTGTAGAATCTGCAAATTCAGCTACTGTTTTTATCT

TATCTGAAGAGATCTTAACAACAGCTAAATCCGAATAAGTATCAGCTCCAACTAATTCAC

CAACAACTTTAGATCCGTCTGCCATAAGAATTTCAATTCGTTTAGCTCCGTCGATAACAT

GGTTATTAGTAACAACGTAAGCGGAGTTGCCATCTTTTCGATAAATGACCCCAGATCCTT

CACTAAAAATAGATAATTCAGCATCCTTATTCTCTTTTGAACGCCCTTCTCCAAAGAGTT

TTGTATAAGGGTTAGAAAGGGATGAAGAAGGGTTATCTTGATAATTAATAACTGATACAA

CTGCATTTTGAACAGCTTTGACAGCTTTAGTAGTATTGGTTGTGTTGTTAAAAACCATAT

TACTAGTTGTTGCCTTACCTGAATTTATTTTTGAAGGAGAATGTGGGTAGAGATTATTGA

ATGTAATAATTGCTATTAATCCTCCTAAAAATCCAATTAGTAAAATACTTAAGGATTTTA

AGATATGTTTCATACTTGGCATGTTTTGTCCTCCGAATTATTTGTCCATAGCTTATAGAA

CTTTGCTTAATTATACCTTAAAAATAAAAAAAAGAAAAGTAATCCACAGGCTGTGGATTA

CTTTTCTTTTTACTAATAAAGACCTTTGTACAAAGGTTTTTATTAGTAGTTGAAGAAAAA

TAACTTGTGGATAAATTGTGTAAAAAGTAATGTTCTTGTGATACAATTAAGCTATGAAAG

TAAAACTAATTTGTGTTGGAAAACTAAAAGAAAGATACCTCAAAGATGGTATTTCAGAAT

ATCAAAAAAGACTTTCCCGTTTTTGTCAATTCGAAATGATTGAGTTAACTGATGAAAGAA

CACCAGATAAAGCAAGTTTTGCTGATAATCAATTGATTATGAGTAAAGAAGCTCAGCGAA

TCCATAAAAAAATTGGGGAACGTGATTTTGTAATTGCATTAGCTATTGAAGGAAAACAGT

TTCCTTCAGAGACATTTAGTGAATTAATATCAGGGGTTACTGTTAAGGGATATTCAACTA

TCACGTTTATTATTGGTGGAAGTCTTGGATTAGATTCAATTATTAAAAAAAGAGCTAATA

TGCTAATGAGTTTTGGATTGTTAACCTTACCTCATCAGTTAATGCGTCTAGTCCTTACTG

AGCAAATTTATCGTGCTTTTATGATTACACAAGGCAGTCCTTATCATAAGTAAGAGAGTG

TGAACTGGAATAACTAACTCTTACCACTTTATTAGATAATAGTTTGGTATAATTAAAAGC

AAATATTAAAAAAAGACAGACTAAATCCTATCTTTTCAATACACTAAGGTAAATACAAAA

AAAGCACTAAAACAGTGCTTTTTTATCTTTGATCCCAGCAGGATTCGAACCTGCGACCGT

TCGCTTAGAAGGCGAATGCTCTATCCAGCTGAGCTATGAGACCTTAACCACATCATTTTA

ACAAAAATTATCTAAAACTGTCAATATTTATTTTAAAAAAACTGGAAAATAACATTATAT

CCCGCGTTGTAAAATGAAGTGCAACAAAAAAGACATGTTCGTCTGATATACTAGAATTCC

CCAATTCAGTATGGAAAGCAGATGAACATGTCTAATATTAATTCTACCAGAAAATCATCC

TATTCTCATCTTTCAGCCACAGAACGGGGTGAAATTGCTGCTTATCTTAAAATGGGAAAG

AAACCCGTTGAGATTGCTCGACTTCTAGGTAGTCACCGTTCTACAATCTGCCGAGAAATA

AAACGTGGTTCAGTAGATCAGGTAAAGGATAAGAATGGAAAACAAACCTTCTTCAACGCC

TATTTCGCTGATAGTGGACAACGTGTCTATGAAACCAATCGTCAAAAGAGTTCTTACCTC

AAATTGAATGGCTGCTCCGCTAGGTTCATTGCACAATTAGAATCTGCCTTGACGGCTAAC

ATTCGTCTCCATAGTGTGGATAGTTTTGTTCAGACTTACAAAGTGAACCATCCTGAAGAA

GTCGTCCCCTCTACCAAAACGATTTATCGTTATATCAAAGAGGGGGTGTTAGCTATTAAA

CCAATCGATTTGCCTAAGATGGTTAGTATTAGAAAACGTTCTAAGAAAGTCATGAAGACG

AATAAGAAGACTTTAGGTAAATCTATCGAAGAACGTCCAGAATATATTAATGATCGTTCT

GAATTTGGGCATTGGGAGATTGATTTGGTTCTCGGCAAGAAAACCAAAGGTGAAGCTGTT

ATGTTGACTTTGGTAGAGCGCCAGACACGCCATGCACTAGGAGTTAAGCTAGAAGACAAG

CAGTCCCAAACCATTAACCGTGCTGTCACGCATCTCATCAGTCAGTATCCTATTGCATCC

ATCACAGCGGATAATGGTTCTGAGTTTAGTTTACTCTCAACCTTAGAAGCTGTTGACGTT

TACTTTGCACATCCCTATTCTTCACATGAGAGAAGAACAAACGAGAACTTCAATGGCCTC

CTCAGAGAATATGTCCCTAAAGGAGTCTCACTTAATCCACTAACCTCTGAAGAACTTGAC

AATGATATTACTGCCATCAATGAGCGCCTAAGACGACTTCTTCAATAGCAATCCTCAAAA

TTCCTGTTTGAGCTATCCCGAACAGCTTAACCTCTGGAACTCTAGTTATCTATGAACTCG

TTGCACTTGACTTGACAACTTGCGGTTTAAAAAAAATCAAATTTTTTTTAAAAAAAGTAT

AGACAAAAAAAATCAATCTGCTATAATAGTAAGAGTGTTAAGGAGATATCCTCCAAAACT

TCTGGTCCGTTGGTCAAGGGGTTAAGACACCGCCTTTTCACGGCGGTAACACGGGTTCGA

ATCCCGTACGGACTATCGCAATTGTCATTTTGGCAATTTTTTTCTTTTTCCGGCATAGCT

CAGTTGGTAGTAGCGCATGACTGTTAATCATGATGTCGTAGGTTCGAGTCCTACTGCCGG

AGTGATAAGATATTTCCTAAGATAATCTTAGGATTTTTTTATTTAGTAAAACAGATACTC

AGGTCAAATACTCAATTTACTAATTATTAAACCACTAAATACAGCATTGTGAACTAACAG

AATTGTATTTTGCGTTAAAATAGAAGTTCTGTTCTTTAATTGTATAGATGATAACATTAA

GTTTAATTACAAACATTAACTGAAGCTTAATTTTTTCCATTTTGAAAGCATGTCTCCCAT

TTAATACTGATGAATATAAGATTATTGTAAGGAGTCAAAAGATTGAAATCGTATCAGCGA

TGATTATTGTCATTTCAATCTTTGAAAGAGAGTAGTTTAACATTAACCCTCACGAAAAAT

CTGACCTTTATATTATTTGAGTTTATTAGTAAAATCGATAAAAGCATCTCAAGTATTAGA

ATATTTGTCTAGCTCTTGAGATGCTTTTAGTATATTGACTCATTTGCCAAATTGATAAAA

AAATCTGATTTTTTTGGACTAAAGTAATTTAGACTTTATATCTATGTACTTATACAAAAG

CATATAAGCGATAATTCCTACACAAGATAGAGATAACCCTAATTTAAAACCATTAGGAAT

AAAGGTAAGGATAACACGTCCCTTTCCTTTAGGAATAGTAACTGATAGAAATCCTCCTTG

TGCTTTTTTGACTGGAAGATTTTTCCCATCTTTTTGTGCTGACCAACCTTTATCGTAAGG

AAGTGTAAAAATAAGAGAACCTTTCGTTTTTGAATTATAATCAGTGATTACCGTATTACT

TTTAGCGTAAGTATGAACATTTTTTTGTTTAATACTATTCATTGCTTCAAGGTAAGATTC

AATAGACAAACTATAAAAATGAGGTTCCTTAAAACTAATTTGTTTATTTTTTGGAAAAAC

AAACGAAAATGTAGCAACTTTGGCATCGGCGAAATATCCTAAATCAAAAAAAGAGTAAGC

GTTATCTGTAGTATATATGAAATTATGATTATCTGTCTGAATACGCACCTCTTTAGCATC

AGGATTTGAAAATATAATATTGGGTATGCTAACATAGAGTTGACTATGTTTAGGGATGTT

AATTTGATAATTAAGGGTAACGGAGTTTTGATAATCAGAAGCTTGTGCAGATATTTGTCC

GTTAAATTGATTAGCACCAGAAATAAGTTGAGCTGGCTGTAAGTTAAAATAGGTTAAAGA

TTTCCCACTTAGTTGGTTAAGTAATTTGGTTTGGTTATCAAGGGTATTGACATTTAGGTT

GACATCTTTGTAAACATTACGTGTTAATATAGCTAAAGGGCTACTATAGTGATTTTGATA

AAGAGTAGTATTCCCGCTAGTTTTTAGTTTTGTAAATCCAAATTTATTTAGAGGTCCTTC

GCTAAGATTATATTTTATACCAAGTAGACTGTCCATAATAATAGTATTGTTTTGGTATCT

AAGGTTTAAATTCGTGCCTTTAGACTGAAATCCCAATCGATCCAATAAAGAACTAGATAG

TCTATTTCTTACAGAGGAAAATTGTGAAATGCCGTAATAATTAAATTTCATGCTATCGTT

CCCTGTTTGGGGAAGTAGTCTTTCCATTCTAAAAAAAGGTTGACTATTTTTTGACACAGA

GTTGACAAGGTTGTTAATATCCTTTAATTGACTATTATATATCTGTCGTGATGGGAATCC

CCACTCCTTATTAATTCCTTGAAGTTGGTAGTAGGTGTTTAACCCTGATTCAAGAAGGCT

AAAGATAAGTATGAAAGCAGAAATAAAAGTAGATGGGATTTGAGAATTTCTAAACGAAAA

TAGTGAAATAGTATAACCTAATAATAAAAAAACACTAAGTAAAAAAAGAGTTAAAGGTAG

AAAATTATATTGTTGAGAAAAGCTATAAGGAAGAGATGTCAGTATAATGAGGAAAATAAA

AGCAAAACCTGCTTTTATTTGAGTCACTTCTGTTAGTCGAGAGAGAGTCTCACATGCGAG

TAATAGGATAACTATGGAAAAAGACCAAGCGTAGCGATGCAAAAACATATTTGGTGAGTG

CATTCCCTGCCAAAAAAGATCAAGTGGCTGTAGGTAAAAACTTATTATAATAAAAGTTAA

CAAGCAGGCATTGGCTAATTTTATTTTTAAAGGGATACTTTCTAAAGTAAAATAAATAAC

ACTAAGCATTAGGAGAAATAATCCTACGTAAATCATAGGCAGAGCATTAAACTTGGTAGT

ATCGTACACTCCTATTGAGAGCTTAGCAGGTATATCCAAAAACCAAGCATTGTTCGTAAC

TAACTGTTTTACCGGGGATAGATTCTCTCCATAAGTTGACAAATCTAGATAGGTAGGAAG

TATTACTAGAGCACTTGTTAAAGCAGCACATATTGACACAGCTGTAAATCTAACAAAAGC

GATAAACATTTTGTTAAAATCATTGAGACTTAAAAGACAAACTAAGGCGTAAAGAATACA

AAAAAGAGCAATCATGTAGCCAAAGTAGTAATTTTGAATGAATAATAATGATATCGAAAG

ATAATAAGTTCTGGTTTTATTTTCTGTGATAAGTTTATTTAATCCAAGTACAACAAGTGG

AAGAAGAATGAAAACATCTAACCAAGAATTTAGTTCCATTTGACTTGTCAAGAAGCTCAT

TAAGCTATAAAAAACTGAGATGGAAATCATCAAGAAAGCACTGATTTTTGGATATAATCT

ATGAAAAGAATAGCATGCAGCTAATCCTATTAACCCAAATTTTATCAAGGTAAACAAATA

GATAGCATCTGGCATAGAGGTTAAATTAAAAAAGAAAAGTAATGGAGAAAAGAAACTGCC

AAGATAATAACACATTAAAGCATAAAAATTTATTCCTAGTCCGCTTGTAAAGGTATAAAA

AAAACTATCAGAACCGTGCATGATGTTACGAAAGTTCTGCGCAAAAATAACATACTGATG

AAAAGCATCACTAGCTAGAATTGTTTTATCACTATTATAATAAATGCCCATCGATAGAAG

GATGATAAATATAATACTAAGAGGGAACAAAAAACTAGCAAGTCCAGCAATTATCCATTT

ATTATTATTTTTCATAAATTATATGGTCTTTCGTTTACAAATTTTAAAAAAGAGGCTAGG

AAGAGATCTTCTTAGCCACCTCATTTATCATAAGGTTTTATTTCCAAAGTTCAGCAACTC

GGGCTTGGACTTCTGGATTGTCAAGGAATTCGTCATAAGTTTCATCTATACGGTCGATAA

CACCATTTTTTGAAATAACAACGATGTGGTTAGCAATCGTTTGAATAAATTCATGGTCAT

GACTGGCAAAGATAACAGATTCTTTGAAGTCTTTGATCCCATCATTAAGACTTGAAATAG

ATTCCAAATCCAAGTGATTTGTTGGATCATCAAGAATAAGAACGTTTGATTTCAATAGCA

TCAACTTAGAAAGCATGACACGCACTTTTTCGCCACCGGAAAGAACGTTAACAGATTTTT

TAACTTCGTCTCCAGAGAAAAGCATCCGTCCTAGGAAACCACGTAAAAAAGTATCATCAT

CTTCACCTTTTGAGGCAAATTGACGTAACCATTCAAGAATAGATTCTTCCGTGGCAAAAT

CTTTTGAATTGTCTTTAGGCAAGTAAGAACGACTTGTTGTTACTCCCCATTTAATGGTTC

CTTCGTAGTCAATATCATCTGCTAAAGCACGCATAAGAGCAGTTGTTTGAATATCATTTT

GACCAATAATAGCAGCTTTGTCACCTGGACGTAAAATGAAACTGATATTATCAATGATTT

TCTCTCCATCGATCGTTACAGAAAGATTTTCAACAGTGAGGAAGTCATTCCCCATTTCAC

GCTCTGCTTTAAAATTAATAAATGGGTATTTACGGCTTGAGGGAACAATTTCTTCTAATT

CAATCTTATCAAGCATTTTTTTACGAGAGGTTGCTTGTTTTGATTTAGATGCGTTTGCTG

AGAAACGGGCAACGAATTCTTGTAATTCTTTGATTTTTTCTTCGGCTTTAGCATTGCGGT

CAGCTTGTAAACGAGCGGCCAGCTCAGAAGATTGTTTCCAGAAATCGTAGTTACCCACAA

AGAGTTTGATTTTACCAAAGTCAAGGTCGGCCATGTGAGTACATACTTTATTCAAGAAGT

GACGGTCATGGGAAACAACGATGACTGTATTTTCAAAATCAATCAAGAAATCTTCCAACC

ATGAAATAGATTGAATATCAAGACCATTGGTTGGCTCGTCAAGTAATAAAACGTCAGGCT

TACCAAAGAGCGCTTTAGCAAGAAGGACTTTCACTTTATCTCCATTAGCCAGTTCACTCA

TATTTTGGTAGTGAAGGTCTTCTGGAATATTTAGATTTTGGAGAAGTTGAGAAGCTTCAC

TTTCTGCCTCCCAGCCACCTAATTCAGCAAAGATACCTTCTAGCTCAGCGGCACGAACCC

CATCTTCTTCTGAAAAATCTGCTTTCATATAGATAGCGTCTTTTTCTTTCATGATATTGT

AAAGTTGCTCATTACCCATGATTACAACATCAATCGCACGTTCTTCTTCATAATCAAAAT

GATTTTGGCGAAGAACAGATAAGCGTTCGTCAGGTCCTAAAGAGATATGACCAGTACTTG

GTTCAATATCTCCTGCAAGGATTTTAAGAAAAGTTGATTTTCCGGCACCATTCGCACCGA

TTAAGCCGTAAGTATTGCCGGCTGTGAATTTGATATTAACATCATCAAATAGTTTTCGAT

CACTAAAGCGTAGTGATACATCAGAAACTGTAAGCAATTTGTCACCTCATTTTATTTATC

TAAGCTTCATTGTACCTAAAATAGTCCCCTTTTTCAATCAGAAAAGGGCTAATCACTACA

TAGGCAATAAAACGTCTTTGTCTTCACGTTTGTATTGTTTAGTCAAACTAAATCCTCTTC

CCATAACTTGAGCTGCTGGCATGACAACAATAAAGGCTGTATCGTCAATTTCGAGGATTT

TTTCTTGGAGAGTAGGTAATTCGTGACTTGATACAATAGCCATTAACATAATTTTATCAG

AAGTAGTATAACCACCGCGAATAGGAAGTTTAGTCACTCCACGATCCATAACAGTTGTGA

TATATTCTCGAATAGCTTGATAATCTTTGGAAATAATCATAACATTTTTGGATGAGTCAA

AGCCATTTTCCATAACACTAATCACATAACCAATTACGAAAAGTCCAATCGTAGAATACA

TCACGTCATCTGCAGAAAGTGCAATAAATCCCATAAGGACACTAATACCATCTACAATTG

TCATTGCAACTCCAAGAGATAAAGGAGAGTATTTATGAAGTATTTGTGTTAGAATTCCAG

TTCCTCCTGTCGAAGAGTTTCCCCAAAAAACCATTCCTAAACCAATGCCACAGATAATAC

CACCAAAAATAGCTGCTAACAACTGATTGTGTGTCAGAGTAGGTAATGAATTGGTTGAAC

GGATGGCAATAGGATATATCCAAGAACCATAAAGTGTTTTAATAAAATTTTGTTTTCCAA

GAAAGAAGTAACACATTAATAATAAGGGAATATTACTAGCCATTAAAAAAAGCGACGGAC

TAATGCCAAATAGAGCTTTTATAACAACTGCAATACCAACCATACCCCCTGATGCAATAT

GATTGTCGACAAACATTGTATTGAAACCAATAGCTGTTATGAATGAGCCAATTGTGACAA

GTAGTAAATCAATCCCTCGTTTTTTCATGCTTTTAATCTCCTTTATAACTGGTTAAAAAT

AGTAGACTCATTATCTCTTAAATGTACTGAGACCGTCAAGTCATATTTTCAAAAAATCTG

GTGAACTTGACAAGAAGTTTAAAATTTCATAGAATAAATCTAAATTAGAAGAGTATTCTT

TTCATGTTTTTAGAGAGTTAGTGGTCGGTGCAAGCTAACAACAGAAAAGGTGAAATGGAC

TAATGATTATTTGAAAGGAAACCATAACTTTCAGGTTGGCACCCCTTACCGTGCAGCTTC

TTTCATTGAGAAGATTGAGATAGTATCTCGTTTTGATAAAGCAGATGCTAATTGAGGTGG

TACCGCGTATTACTTGTAATAACGCCCTCACGTTTTAATAGCGTGGGGACTTTTTGCTAT

ATCAAGAGATAGTTAGGGAGAAAATGATGACAAAACCTATTATTTTAACTGGAGATAGAC

CAACTGGAAAATTACATTTAGGTCATTATGTCGGAAGTCTTAAAAATCGTGTCTTTTTAC

AAAATGAAAACAAGTATAAGATGTTTGTTTTCTTGGCCGATCAACAGGCACTGACGGATC

ATGCTAAAGAATCCGAATTAATTCAAGAATCTATTGGAAATGTTGCCTTGGATTACCTCT

CTGTCGGTTTGGATCCGAGGCAATCAACGATTTTTATTCAAAGTCAGATTCCAGAGCTAG

CTGAATTGAGCATGTATTATATGAATCTGGTATCATTAGCACGTTTGGAAAGAAATCCCA

CTGTTAAAACCGAAATTGCTCAGAAAGGTTTTGGCGAAAGTATTCCATCTGGTTTTTTGG

TTTATCCAGTATCACAGGCCGCTGATATTACAGCATTTAAAGCGAATTTAGTACCTGTAG

GTAACGACCAGAAACCGATGATTGAACAAACACGTGAAATTGTGAGAAGTTTTAATCATA

CTTACCATACAGACTGTTTAGTAGAACCTGAAGGTATTTATCCAGAAAATGAAAAAGCTG

GACGCTTACCTGGTCTTGATGGCAATGCCAAAATGTCTAAATCATTGGGAAATGGAATCT

ATCTTTCAGATGATGCAGATACCGTTCGCAAAAAAGTGATGAGCATGTATACTGATCCAA

ATCATATTAAAATAGAAGATCCTGGTCAAATTGAAGGGAATATGGTCTTTCATTATTTGG

ATATTTTTGCTAGAAAAGAAGATCAAGCTGATATCGAAGCAATGAAAGAGCATTATCAAA

GAGGTGGTTTAGGAGATGTGAAAACGAAACGCTACCTTCTAGATATTTTAGAACGTGAAT

TAGCACCTATTCGTGAAAGACGTTTGGAGTACGCTAAAGATATGGGAGAGGTGTTCCGTA

TGTTACAAGAAGGTAGTCAAAAAGCAAGAACTGTGGCAGCCAAGACTTTATCAGAAGTGA

AGTCAGCAATGGGTATTAATTATTTTTAATGTTTTAGAGAAAAAGCTAAGTTAGTAATGA

TAAGATAATTACTATTATCAAAGCAGGACCTAATTTGTCATTTCTTTACAGGATAGTAGA

GATTATGTTTTATATAGTCTTATTTTTGATTAAAAATAAGACCCAAATGAATTTTTTGAT

TAATAAAAAACGGATAATACTGATATATTATGCTGATAGTGACTTCAGCTGTAGGAATAT

TTTATTGACAAATGATTTTAGCGTGATATGATAGTAACAATAAAAAATAGACCAGGGTAA

TATTCCCTAAAAATAAAAGAAAAGAGGATTATCTGGATGTCAAATTGGGACACTAAATTT

TTGAAAAAAGGCTACACTTTTGACGATGTCTTGCTTATTCCAGCAGAGAGTCATGTTCTT

CCAAATGAAGTTGATTTAAAGACAAAGCTCGCAGACAATTTGACATTAAATATTCCTATT

ATTACAGCAGCTATGGATACGGTTACTGGTAGTAAAATGGCTATTGCAATTGCGCGTGCG

GGTGGTTTAGGTGTTATTCATAAAAACATGTCTATTACTGAGCAAGCAGAAGAAGTTCGC

AAGGTAAAACGTTCTGAAAATGGTGTCATCATTGATCCATTCTTCTTAACACCTGAACAC

AAGGTTTCAGAAGCTGAAGAATTAATGCAGCGCTACCGTATCAGTGGTGTGCCAATTGTT

GAAACTTTAGCAAACCGTAAGTTGGTCGGTATCATTACTAATCGTGATATGCGTTTTATT

TCTGATTACAATGCACCAATCTCTGAACATATGACTAGTGAGCACCTCGTTACGGCTGCT

GTTGGTACAGATCTTGAAACTGCAGAACGTATTCTTCATGAACACCGTATTGAAAAACTT

CCTTTGGTTGATAATAGCGGTCGTCTGTCTGGTTTAATCACTATCAAAGATATTGAAAAA

GTAATCGAATTCCCACATGCTGCTAAAGATGAATTTGGTCGTCTTCTGGTCGCTGCTGCG

GTAGGTGTTACCTCAGATACTTTTGAGCGTGCCGAGGCCTTGTTTGAAGCTGGTGCAGAT

GCGATTGTTATTGACACAGCTCATGGTCATTCGGCTGGTGTTCTTCGTAAAATTGCTGAG

ATTCGTGCTCATTTCCCAAATCGTACTTTGATTGCTGGAAATATTGCCACTGCTGAAGGT

GCGCGTGCTCTTTATGATGCAGGTGTCGATGTTGTTAAAGTGGGTATTGGTCCCGGATCT

ATCTGTACCACTCGTGTGGTTGCAGGTGTCGGTGTTCCTCAAGTGACTGCTATTTACGAT

GCTGCTGCTGTTGCTCGTAAATATGGCAAAACGATTATTGCTGATGGTGGTATCAAGTAT

TCTGGCGATATTGTTAAGGCTCTTGCAGCCGGCGGTAATGCTGTTATGCTTGGATCAATG

TTTGCAGGTACTGATGAAGCTCCAGGTGAAACTGAAATCTACCAAGGCCGTAAGTTCAAA

ACTTATCGTGGAATGGGTTCCATTGCAGCTATGAAGAAAGGATCAAGCGATCGTTACTTC

CAAGGTTCCGTAAATGAAGCTAATAAACTAGTTCCAGAAGGTATTGAAGGCCGTGTTGCT

TATAAAGGAGCTGCTTCAGATATTGTCTTCCAAATGTTAGGTGGTATCCGCTCAGGTATG

GGCTATGTTGGCGCAGGTGACATTCAAGAATTACATGAAAATGCTCAATTTGTTGAAATG

TCAGGTGCAGGCTTGATTGAAAGTCATCCACATGATGTCCAAATCACAAATGAAGCACCA

AACTATTCTGTGCACTGATAATTTAATTTAGTAACTTTAAGGGCTGACATATTGTGCCAG

CTCTTTTTTTATATTAAAAAATGATAAAATATAATAACAATTTTCGGATAACTTTTATTG

TTTTTTCTTAGTAAATAAGCTATAATTAGTCTTTACATGAAACCATTTTATGATAGAATT

ATTATAATCTTTAAAGGTTTACTATAAAGGAGATAACATTTTGGAAGGTATTTTTTATGC

ACTTATTCCCATGTTTACATGGGGAAGCATTGGATTTGTTAGTAACAAAATTGGAGGAAA

ACCTTCGCAACAAACTCTAGGAATGACTTTTGGGGCTTTATTATTTTCATTAGCTGTTTG

GTTAATTGTTAGACCTGAGATGACACTGCAACTTTGGCTTTTTGGAATATTGGGTGGTTT

TATCTGGTCAATTGGTCAAACTGGTCAATTTCATGCCATGCAATACATGGGAGTCTCCGT

TGCCAATCCTTTGTCAAGTGGCTCTCAACTTGTTCTGGGAAGTTTGATTGGTGTGCTCGT

TTTTCATGAATGGACAAGACCTATGCAATTTGTAGTAGGAAGTCTAGCACTATTACTTCT

GATTGTTGGTTTTTACTTTTCTAGTAAACAAGATGATGCTAATGCACAAGTCAATCACCT

TCATAATTTTTCAAAAGGATTTAGGGCGCTCACTTATTCAACAATTGGTTACGTGATGTA

TGCTGTATTATTTAATAACATCATGAAATTTGAAGTTTTGTCAGTCATTTTACCCATGGC

AGTAGGTATGGTTTTAGGAGCTATAACTTTCATGTCATTTAAAATTTCCATTGACCAGTA

TGTGATTAAAAATAGCGTTGTTGGATTACTTTGGGGCATTGGGAATATTTTTATGCTTTT

AGCTGCATCAAAAGCAGGACTTGCTATTGCCTTTAGTTTCTCACAATTAGGTGCTATCAT

TTCGATTGTCGGAGGCATTTTATTCCTAGACGAAACCAAAACCAAAAAAGAAATGCGTTG

GGTTGTCACAGGTATTATCTGTTTTATCATAGGTGCGATTTTATTAGGTGTGGTCAAATC

TTAATCGCCACATAACATCAAAAAAAGGCTCAGTTTAACTGAACCCTTTTTTGATGTTAT

TAGTGAATGTCAGAATCAACTTGAACGGTTCCTTTTGTCACGTGAAAAATACGGATACCT

TCTGGCAACTGTGACAGGTGATCCAAACTTGTTGTTGTGATAAAGGTTTGGACATTTTCT

TTAATTACTGTTTCAAGTAACTTTGTTTGTCGTGTATTGTCTAGCTCGCTCATGACATCG

TCTAGTAAAAGAATGGGATTATCTCCAGTCAAGGCCTTCATTAAACTAACTTCAGCCATT

TTTAAGGAAAGAATAAGGCTGCGGTGCTGACCTTGACTAGCAAAGTTAGCATTCATCCCG

TTAATGTAAAAGGCTAAGTCATCGCGATGTGGACCAACACTAGTGTTTTTACGGAAGAAA

TCTTTCTGATGATTTTTTTCAAGTTGGTGTAAAAATTGTTGGTAAATACTAGTTTTTTTG

TCAAAGACAACTGACGACTGATAGGAGAGAGAGAGACTTTCTAAACCATTTGAGATGGCT

TGGTGATGAGTATTTGCTTCTTTCTCTAACGCATTGATAAAATCAATACGATGTTCCATG

ACACGGGCTCCATAGCTAGCTAATTGCTCATCTAGAACAGCTAGAAAAGCGGCATCAATT

TGTTGGGCACTTTTTAAATAGCTATTACGCTGTTTAAGCACGTGGTTATAATGGGATAAC

TCAAATAAGTAAACAGGCTTAATTTGACCAAGGTCAATGTCGATAAATTTGCGACGAAGG

CTAGGTGCTCCCTTAACTAGTTGCAAATCTTCTGGTGCGAAAAGCACAACCATCATCGTT

CCAATATAATCAGATAATTTTGCTTGCTTTAGCGCATTAATCTTTGTCACACGTCCTTTA

TCTGATAGATTGATTTCTAAATCAACAGTACCACTAATACGCTGAATCTTACCAGTAAGA

GAAACAGTTGAGTGATCAAAATGAATCAATTCTTTATCAGCCCTCGTGCGATGACTTCTA

GTAAGTGATAGAAAATAGATTGCCTCGAGAAAATTGGTTTTTCCTTGAGCATTATTACCG

ATAAAGACATTTAAACCCGACGAAAATGAGGCAAGCAGGTGGTCATAATTGCGATAATGT

TTTAACTCTAACTCTTTAATCCACATACTAGATACCTGGGAAGCGGACAGGTGCAGTTGG

TTTACCTTTAGTTTTCTTAGTTGCTCTTAGTGATTTTTTGGTAGTAGATTGACGATTGTT

ATGTTTAGAAGAAGTCTTTTTGTTAGCTTGGTTCATTTGTTTCACTAGAGCCGCTACACG

TGTCTTTTCAGCCATTTCTTCGGCAAATTGTTCTTTTTCTTCTTGATTAGGCTCAACAAT

AATGATAATTAAGTCTTGGTCAGGTAGAGAAATCTTATCCCCTATTCTGATTTTTTTGCC

TCGACGTTTCTCATCTTCACCATTAAATAGAACGGTTGTTTCAGCGAGAAATCCCTTGAT

AGCGCCACCACTTTGGATAATTCCTAATTCTTTAAGAAGGGCTTGTAGGGTGATAAATTC

TGTAAATAATTTGTATATCATTGTCATCTCCATTTTGTCATTCAAGTCATTATAGCATAA

AACAGAAATAAATAATAATAGCTGAATAAGAGCTATTTGACTATTCCAAAGTGAGACGTT

ATTTGATATAATATAATCTGTATTTTTAAGTATAAAGTGTGGTTGAAACTTTTTAGAGGT

ATTACTGGCTTTATTTTGCAGTAAATCATAGGAGCAAGTCAATTGCTTCTTTTTTTGTTT

AACCGTAAGGAATTTAAAAAGTTATCACTCACCTAACCTGAATAAAAATGGTATAATCGT

AAGGATCACACTATTTATTAAGGAAACATTATGAAAATTGTTCAGGGAGTACAACTTCAT

CTGATTAAGACCAAACAGTTTAAAACCAATCATATTACCTTTCGTTTTTCAGGTGATTTG

AACCAAAAAACAGTAGCAAAAAGAGTCTTAGTGGCACAGATGTTAGCAACAGCTAATGAG

TGTTATCCAACTGTTAGGCAGTTTAGAGAAAAATTAGCACGACTTTATGGAGCTAGCTTG

TCAACTAACGTGTTAACAAAAGGGTTAGTTCATATTGTGGATATCGATATTACTTTTATT

CAAGATAGATATGCTTGCAATGGGGAAAAAATACTAGATGAAATGATTCAGTTTTTAAAA

GATATCTTGTTCTCACCCCTATTATCCATCGCCCAGTATCAGCCTAAAATTTTTGAGACT

GAAAAAAATAATTTGATTAACTACATCGAGTCAGATAGAGAGGATTCTTTTTACTACAGC

TCTCTTAAGGTAAAAGAATTGTTTTATTGTAATAAGAATCTACAAATGTCAGAATATGGT

AGCCCAGAACTAATTGCAAAAGAGACTGCCTATACTAGCTATCAAGAATTTCATAAAATG

CTCAATGAAGATCAGATTGATATTTTCATTTTAGGAGATTTTGATGATTATCGCGTTGTT

CAGCTAATTCATCAATTTCCACTTGATAACCGAAATAAAAACCTAAATTTCTTTCATTTA

CAAACTTCTGTCAATATTATTAAAGAATCTATTGAAAAAAGAGCAGTTCATCAATCTATT

TTACAATTAGCTTATCACTTCCCTTCAGTTTTTGGACAACGTGACTATTATGCTTTAGTT

CTTTTAAATGGGTTGTTAGGCTCTTTTGCACATTCCCGTCTCTTTGTAAAAATTAGAGAA

GAAGAAGGACTTGCTTATAGTATAGGGTGTCGTTTTGATAGTTATACAGGTTTATTTGAA

ATTTATACTGGAATTGACAGTCAACATCGCACAAAAACGTTACAACTAATCATCCAAGAA

TTAAATGACATTAAAATGGGACGATTATCAGAACAATTGATTAAAAAGACCAGGTCGATG

TTGTTGAATAATGCCTTATTATCAGAAGATTATAATAAAAATATAATTGAAAGAATATAC

ACATCTTCTTACATTGATTCTTCTTATTCTATTAAAAATTGGATTAAAGGGGTTAATGAA

GTTAATAAAGCAGATATCATTAAAGTAGCTAATCTTTTAAAACTTCAAACTGTCTATTTT

TTAGAAGGAATATAAAATGACAAAATTAGTAAAGATAAACTATCCTAATATTGACGAAGA

CCTTTACTATGTAAAATTAGAAAATGGTTTAACTGTTTATTTTATAAAAAAAATAGGGTT

TTTAGAAAAAACAGCTATGTTGACAGTAGATTTCGGTTCTCTTGACAATAAGTTGACAGT

CGATGACGAATCACGAGATGCTCCAGCAGGTATTGCACACTTTTTAGAACATAAACTCTT

TGAAGATGAATCAGGTGGAGATATTTCATTAAAGTTTACACAACTAGGAGCAGAGACAAA

TGCCTTTACAACTTTCAACCAAACGAGTTTCTTCTTTTCAACAGCTAGTAAATTTCAGGA

AAATTTGGAACTCCTTCAATATTTTGTTCTTTCTGCTAATATAACAGATGAATCTGTTAG

TAGAGAAAAAAAAATAATCGGGCAAGAAATTGATATGTATCAAGATGATGCTGACTACCG

TGCATATAGTGGAATCCTTCAGAACTTATTTCCAAAAACAAGTTTAGCTAATGATATTGC

TGGCTCAAAAGAATCCATTCAGAAGATCACGAAAATATTATTGGAAACGCATCATACTTA

TTTTTACCAACCTACTAATATGAGTCTATTCATTGTCGGTGATATTGATATTGACGAAAC

TTTTTTAGCTATTCAAAGGTTTCAAACCACATTATCCTATCCAGATAGAAAAAGGGTAAC

TGTTGATCCTTTACACTACTATCCTGTTATTAAATCATCTTCTGTTGACATGGATGTTAC

TACTGCTAAGCTGGTTGTTGGTTTTCGAGGGTATCTGACCTTAACTCAGCATTCACTATT

AACGTATCGTATTGCCTTGAAGCTATTTTTATCAATGCTTATAGGTTGGACTTCTAAAAT

CTATCATACGCTATATGAAGATGGTAAAATAGACGATTCATTTGATGTGGATGTTGAAAT

TCATCATAATTTTCAATTTGTATTAATATCACTTGATACACCAGAGCCTATCGCAATGTC

TAACTATATCCGTCAAAAATTAGCCACTATAAAGATATCAAAAGAGTTTACAAATGAGCA

CTTAAATCTATTAAAAAAAGAAATGTATGGTGACTTTATTCAGAGTTTAGACTCTATAGA

ACATTTAACCCATCAGTTCAGTCTATACCTGTCTGATTCTGATAAAGAAACATATTTTGA

TATTCCAAAAATTATAGAAAGACTCACTCTAAAAGATGTTGTTACAATTGGAAAAGCATT

TTTTGAAAAAGCTGATGCTTCAGACTTCACAGTCTTCCCAAAATAGATGAAAATCTGTTA

TAATAGGGTTACTATATTATTTTTGAGGTACCTGTATGAGTGAAAAAAGTCTTGGTGAAG

TGTTAAGAGAATCGCGTGTTGGAAAAAATATCACACTAGATGATATAGAATTGAAGACAG

GGATTTCTTCCCATTACTTGCTGGCAATGGAATTGGATCAGTTTAAAATTATTCCGGAAG

AAAAATTTGATCAATTTTTAAAAGAATATGCTGACATTGTTGAGTTAGATTTCAATACTT

TAAAAAGACGATACCGTTATCAAGTTAATTCCAAAAAAGACAGTGATATAAAATCTGTAA

CTGAGATTGTTGAAGAGAAGTTAAGTAAGAAACGATTACAAGAGAAGTTGTTATCACATC

AGAGCATGGGGTATGGTCAACCGTTAGAAGTTTCTAAAAACAAAGAAACCTTAGTTATCC

CTGTAAAGACTGTTGGTTTAGATAGCAAATCGCCACTAAAAACGATATCGCCTGTTAGCA

AATTTAAATCAGATAACAGGTTAATTCCTAAAAAAAATGTGTCACGTATGAGGCGAAATA

GCACTGACAGTCGTTCTAAAACGTCACTATTTCCTATAGTTATATTAGGAACGATTGCTT

TTGCAATTATCAGTGTTATCATTTTAGGTGTTTGGCAACAATTCGAAAAGAGTCAAAGAG

CTAAAGAAGCTGAAGTTGCTTTGCTTAAAGCCTCTGAGCAGTCTACTAACGGTTTAAAAA

ATAATTCAAATGATCGCAAAACTCAAGTCACAACTGAGGGGTCGGGGAATTATTTAATAG

CGACTGTTACTAAATCTAAAGAGACCGTTGATATCAGTGTGTCTTTAACCGAGGCACAAA

GTAGTTGGATTTCTCTAACAAATTCAGAAATTGGTGAAGGTGGTATAACATTGACTCAAG

ACTCGCCAACTTATACAGCGACTTTACCTGCAGAAATAACAGAATCATTGTTGACGCTTG

GTGTGACAAATGGTGTATCTGTAACAATTGATGGACAACCTGTTGATCTATCACCTTTAA

CAAGTACTGACTTAAGTTATATCACGTTTAAGATCCAATAATTAAGGTAGACTAATGATA

AAAAAAGAAAATATTCCTAATCTATTAACTCTTGTTCGAATCGCAATGATTCCTTTCTTT

CTTTTTATTACTTCCTCATCTAATAAGGTGGGGTGGCATATTTTTGCAGCTGTGATTTTT

GCAATTGCCAGTTTTACCGATTATCTAGATGGGTATCTTGCGCGTAAGTGGCATGTAGTC

AGTAATTTTGGAAAGTTTGCCGATCCTCTAGCAGATAAGATGCTTGTCATGAGTGCCTTT

ATCATGTTAGTTGGACTTGGCTTAGTTCCTGCGTGGGTATCAGCTGTTATTATTTGCCGA

GAATTGGCAGTAACTGGTCTTCGATTACTACTTGTTGAAACCGGAGGAAAGGTCCTTGCA

GCTGCTATGCTGGGAAAAATCAAAACAGCGACACAAATGTTGTCTATTATTTTATTACTT

TGCCATTGGATATTCCTAGGAAACGTCTTACTCTATATTGCTCTTTTTTTCACTATTTAT

TCTGGATATGATTATTTTAAAGGAGCAAGCTTTCTTTTTAAGGATACGTTTAAATAACAT

GTCAGCTATTATTGAACTTAAAAAAGTTACATTTAATTACCATAAAGACCAAGAAAAACC

AACATTAGATGGCGTATCGTTTCATGTGAAACAAGGTGAGTGGCTGTCTATCATTGGCCA

TAACGGATCTGGTAAATCAACGACTATTCGGTTAATTGATGGATTATTGGAACCAGAATC

AGGGTCTATTATTGTTGATGGAGATCTACTAACCATAACTAATGTTTGGGAAATTCGTCA

TAAAATTGGTATGGTCTTTCAAAACCCTGATAATCAGTTTGTTGGAGCAACTGTTGAAGA

TGATGTTGCTTTTGGGCTTGAAAATAAAGGTATTGCACATGAAGATATAAAAGAGAGGGT

TAATCATGCTTTAGAGTTAGTCGGCATGCAGAACTTTAAAGAAAAAGAACCAGCCCGTTT

ATCTGGTGGCCAAAAACAGCGCGTAGCTATTGCAGGTGCAGTTGCTATGAAGCCTAAAAT

TATTATTTTAGATGAAGCTACTAGTATGCTTGACCCTAAAGGACGATTAGAGTTAATTAA

AACTATAAAAAACATCCGTGACGACTACCAGCTGACTGTTATTTCCATTACTCATGACTT

AGATGAAGTTGCTCTTAGTGATAGAGTTTTAGTGATGAAAGATGGTCAAGTGGAATCAAC

ATCAACACCAGAACAATTATTTGCAAGAGGGGATGAACTACTCCAATTAGGTCTTGATAT

CCCTTTTACAACATCTGTTGTACAGATGCTTCAAGAAGAAGGTTATCCTATTGACTATGG

ATATCTCACAGAAAAGGAATTAGAAAATCAGTTATGTCAATTAATCTCCAAAATGTAAGT

TATACTTATCAAGCAGGGACTCCTTTTGAAGGGCGTGCCCTTTTTAACATTAATTTAGAT

ATTTTAGATGGTTCTTATACGGCTTTTATCGGACATACTGGATCCGGGAAATCGACTATT

ATGCAATTGCTAAATGGTTTACACGTTCCGACAACGGGAATTGTCAGCGTAGATAAACAA

GATATAACGAATCACTCTAAAAATAAAGAGATTAAATCTATTCGAAAACATGTAGGATTA

GTTTTTCAATTTCCAGAAAGTCAACTATTTGAAGAGACAGTTTTGAAAGATGTTGCTTTT

GGCCCTCAAAATTTTGGGGTCTCTCCGGAGGAAGCAGAAGCTTTAGCGCGTGAAAAACTA

GCTCTTGTTGGGATTTCAGAAAATCTTTTTGAAAAGAATCCATTTGAACTCTCAGGGGGT

CAAATGCGTCGTGTTGCTATTGCGGGTATCCTTGCTATGCAGCCAAAGGTTTTAGTTTTA

GATGAACCGACTGCTGGTCTTGATCCTAAAGGGCGTAAAGAATTAATGACTATTTTTAAA

AAGTTACACCAATCTGGCATGACTATTGTTTTAGTAACCCATCTGATGGATGATGTGGCC

AATTACGCAGATTTTGTATATGTTCTTGACAAAGGTAAAATTATTTTATCAGGAAAACCA

AAAACAATTTTTCAACAAGTCAGTTTGCTTGAGAAAAAACAGTTAGGAGTTCCTAAGGTG

ACTAAACTTGCTCAAAGGCTTGTTGATAGAGGAATACCTATCTCTTCTTTGCCTATTACC

TTAGAGGAGCTAAGAGAGGTACTAAAACATGGATAAGCTGATTCTTGGTCGTTATATTCC

CGGAGATTCTCTCATTCATCGTCTTGACCCAAGAAGTAAATTATTAGCGATGATTATTTA

TATTGTCATTATCTTTTGGGCTAACAATGTTGTGACAAATTTGTTGATGTTGACTTTTAC

TTTAGCAGTTGTTTTTTTGTCAAAAATCAAGTTGTCTTTCTTTTTAAATGGGGTTAAACC

AATGATTGGTATTATCCTCTTCACGACTTTATTTCAGATGTTTTTCTCACAAGGAGGAAA

AGTAATTTTTAGTTGGTGGTTCATTAGCATAACTGATTTAGGATTGAGCCAAGCTATTTT

AATTTTTATGCGTTTCGTTCTAATTATTTTCTTTTCAACGCTGTTAACGTTGACGACAAC

ACCTTTAAGCCTATCTGATGCAGTTGAATCATTATTAAAACCTTTAACAAGATTTAAAGT

TCCAGCTCACGAGATCGGACTAATGCTTTCTTTAAGTTTACGTTTTGTGCCTACTTTAAT

GGATGATACCACGCGTATTATGAATGCACAAAGGGCCCGTGGAGTTGATTTTGGAGAGGG

AAATTTAATTCAAAAAGTTAAATCCATTATTCCTATTTTAATCCCTCTTTTCGCTTCAAG

CTTTAAAAGAGCCGACGCATTAGCTATTGCTATGGAAGCTAGAGGTTATCAGGGAGGAGA

AGGGCGAACTAAATATCGTCAATTAGACTGGCAGCTAAAAGATAGTTTAGCTATTGGAAT

AGTTTCTTTATTGGGTTTGCTACTTTTTTTCTTGAAAACCTCTTTGTAATATTTTTTAGC

ACAAAACTTTTCTTTATTATAAGAAGTGAATTGTACCTCAAAAGTTAGATACAACATCTA

ATGATTGAGGTACTTTTCTTCATTTGAGGAATTGAAAACCGCTATCTCAACTATATTAAT

CATCCCAACAATAACAAATCGAACGAAATCTAAAAGGACTGAGTCCTGTTCAATACAGAA

CTAAATTCTTAGTTTAATGATATTATCCCGCTTTTTAATATCGCTAAGAAGAAGAGTTTT

ATGATTTTTTACAACTTCTTATAATATCAATGTAGTTGTAATATTTTTAATATATCTTCA

ATAACGAGGTAATAATTAAAAGATATTATGGATAACGGAGAAAAGTGAGGTATTATTGAA

TGTTTAAGAAAGAAAATTTAAAACAACGTTATTTTAATTTTGGATTAGTAGCGTTAGCTC

TAACAATATTAGCCATCATTTTTGCCTTCTCAAGTAAAAATGATGATACTAAGTCTTATG

CTAAGAAGTCAGAAAGTAAAATGGTAACAATCGACAAGGCTCCAAAAAATAATCATGCTA

TTACTAAAGAAGAAAGCAAAGAAAAAGCAAAGAGCATTGCTTCGGAGCCTATTCCCACAG

TAGAAAACTCTGTAGCTCCGACAGTAACAGAGGAAGCACCGGTTGTTCAGCAAGAAGAGA

CTCAAACTGTTCAGCAGGTATCTTCAGTAGCCTATAATCCAAACAATGTGGTACTTTCTA

ATGGAAATACTGCTGGTATTGTAGGAAGTCAAGCGGCGGCACAGATGGCAGCAGCAACAG

GTGTTCCACAATCAACTTGGGAACATATAATTGCGCATGAATCTAATGGAAATCCTAACG

CAGCTAATGCTTCTGGGGCATCAGGGTTGTTCCAGACAATGCCAGGTTGGGGTTCTACAG

CAACGGTTGAAGATCAAGTCAATGCAGCCTTGAAAGCCTATAGTGCACAAGGTTTATCAG

CTTGGGGTTACTAAATAAACATAGCTGGAAGAAATAAAACCTATTGTTAGTATTTACTAA

CAATAGGTTTTTGGTATTTAATTGTCTTTATGATTTGTAGTGATGAATTAGTCTCAAAAC

AAAACATTGCCTTATCAGGGAATATAGCTCGTATTATTTAGGAAAACTGACCAGCACTTA

AGTGCCTCTGATATTTAAGGCAGTTATACTGATAACAAGCAGGAAAAAAACTCTTAAATG

TTTATAAGGTCATTAGCAATTTTCAGTATTTTACACTACTACACTTTTGAGTTTCAACAA

TTTACTAGGAGTTGTTTCTAAGGGGAAACAACATAGCTTGATAGCTATCTATTAGTCATT

TTAGTGAATATATAGCAGAAAAATAAAACGCTTTCAAATATTTCTAAGAATTTTTCTAAC

TATCTATTTATTCGCCAAATATTTCTACGCTGTAACGTCGTCCAGTTGGAGTAGCGGCTA

AACCACCTTCTGCTGTTTCACGGAAAGCAGTTGGCATGGCAGAGCCGACTTGATACATAG

CATCAATAACTTCGTCAACAGGAATTTGAGATTCAATATCAGCTAAAGCCATATCAGCTG

CGACAAGGGCGAAACTTGCTCCTAGGGCATTGCGTTTGACACAAGGCACTTCTACTAGGC

CGGCAACAGGATCACAGACAAGTCCTAATAAATTTTTGATAACAAACGCAATAGCTTGAC

TAGCTTGATGAGATGTTCCACCAGCTGCCTTAACAAGAGCAGCTGCACTCATAGCAGCTG

CAGAACCAACTTCTGCTTGGCATCCACCTTCTGCACCAGAAATAGAGGCGTTATTTCCAA

TAACAAGACCAAAAGCTCCTGCAGTAAATAAAAACTCTAATTGTTTTTTTTCTGATAAGT

CAAGTTTTTCAATAGCTGTTGCGAGCACTGCTGGTAAGCATCCTGCACTTCCTGCAGTGG

GCGTTGCACAGACAAGTCCCATTTTAGCATTTAATTCGTTTACAGCTATAGCATTTCTAA

CAGCATTAAGAATAGTAGTATCAGAAAGGCTGTTCCCTTTTTTAATGTAGTTATCCATTT

TGACCGCATCGCCCCCTGTCAAACCACTGATAGATTTGGTTGAGGTTAGTCCTTCTGTTA

CAGCTGCTTTCATAACTTGTAAATTGCGTGACATGATTTTGATAATATCTTCTCGATTTC

TTCCAGACATCTCCACTTCCGTGGCAATCATGAGTTCAGCAATATTTCCATTAAATTGTT

GGTCAGCTTGTTTGACAAGTTCTTCAATAGTATAAAACATAGCTTTCCTTTCTAGTCAAA

GAAGTTGACATTATAAATATGAGGAATTTTTGCAATTTGGTTGGCTGCTTCTTGGCACTC

TCTAGAATCGACTTCTATAATCATAGTAGCTTTTTCACCAGCGGATTCACGTGTAACATT

CATTGTGGCGATATTAATATTATTGGAAGATAGGATATCAGTTACCTTTGCGATCATTCC

AGGAATATCTTTGTGTACAGTAACTATAGTAGGAGTATTCATGGATAACGAGACAGAAAA

ACCATTTAGTTCTGTTACTTGAATGTTTCCTCCTCCAATTGAAACACCAGTAACACTAAG

TGTTTTGTCAGCTTTTTTGACACTGATTTTAACAGTATTGGGATGGGGAGCATTACTATC

TTTTAAGATATCCCAATAGATTTTTATCCCTTTTTGATGAGCAATTTCTAGAGAATTTTT

AATATCAGGATTATCAGTATCCATACCCATAATTCCTGCAACAAGAGCCTTATCAGTACC

ATGTCCTCTGTAGGTTTTAGCAAATGAATTATAAAGGTGAAACGTCACTTCATCAGGAAT

ATCTCCAAAGATGGAATGGACTACCTTACCAATTCTGACGGCACCAGCTGTGTGACTACT

TGATGGACCAATCATTACTGGCCCAATGATGTCAAAAACTGATTGAAATTTTTGTGTATT

CATAGGCAACTCCGTTTTTTATTTCTATGTTTATTATAACAAAAAAATATCAAAAAACAG

CAATATAATTATAAAAAATTATTTTTATAATAAAAAATTATTGAGTAATAAAATGATTGG

CATTCTACGCAGTAATTGTTATAATAAGGTTTGCGCTGATGGCGCTTCAAAATCGACCTT

CAAATCGACTTTTGAAAACGACGACCTTCAATCGTACGTTAACTGATAAGAAAAAGAATC

ATAATATTCATAATATGATGATTATCTTTTTCTATCTTATTATTGATGGGAGAATTTTTT

ATGACAGATAACTCAAAGATACGTGTTGTCGTTGGTATGAGTGGTGGCGTTGATTCATCT

GTAACAGCTCTACTTTTAAAGGAGCAAGGTTATGATGTTATTGGTGTCTTCATGAAAAAC

TGGGACGATACAGATGAATTTGGTGTTTGCACAGCTACTGAAGATTACAAAGATGTAGCA

GCCGTTGCTGATCAAATCGGTATTCCTTACTATTCAGTCAATTTTGAAAAAGAATATTGG

GACAGAGTATTTGAATATTTCTTGGCCGAATACCGAGCAGGTCGTACTCCAAATCCCGAT

GTCATGTGTAATAAGGAAATAAAATTTAAAGCTTTCCTTGACTATGCCATGACTTTAGGT

GCAGATTATGTAGCAACGGGACACTATGCTCAAGTCAAGCGTGATGAAAATGGTACGGTT

CACATGTTACGTGGAGCTGATAATGGCAAAGACCAAACCTACTTTTTAAGTCAACTATCA

CAGGAACAACTACAAAAGACTTTATTCCCCCTTGGTCATTTGCAAAAGTCAGAAGTTAGA

GAAATTGCTGAGCGAGCAGGGCTTGCAACGGCTAAAAAGAAAGATTCAACAGGTATTTGT

TTTATTGGTGAAAAGAATTTCAAACAGTTTTTGAGCCAGTACTTACCGGCACAAAAAGGT

CGCATGATGACTATTGATGGTCGCGATATGGGAGAACATGCCGGTCTTATGTATTATACT

ATTGGTCAGCGTGGTGGTCTGGGCATTGGTGGTCAGCATGGTGGGGATAATCAACCTTGG

TTTGTTGTCGGTAAAGATTTGTCGCAAAATATTTTATACGTTGGTCAAGGTTTCTACCAT

GAAGCCCTAATGTCAAATAGTCTTGACGCCTCAGTCATCCATTTTACACGTGAGATGCCA

GAAGAGTTCACATTTGAATGTACTGCTAAATTTCGCTATCGTCAACCCGATTCACAGGTG

ACGGTGCATGTTCGAGGAGATAAGGCAGAAGTTGTTTTTGCAGAGCCACAGCGCGCGATT

ACTCCTGGTCAAGCTGTTGTTTTCTATGACGGAAAAGAATGCCTTGGTGGTGGTATGATT

GACATGGCTTATAAAAATGGGCAGCCTTGTCAGTACATCTAATGAATCTTTTATTAAGAT

AAAAAGAAAGGGACATTCAACAATTCCTTTGACATCATATGATATTATTTATAAAAACCA

ATCATGCGTTAATGGATTAATTTATGATATCTGTATATTTTAAATATAATCACATTATTT

TTAAAAATAGTTCGTAAAAGTTGAGTCATTACTTTATTAGTTACTGTAGAAATCATACGA

TCATGAGACTCACTTTCTCTAGTATTACAACAGATATTTTGATGATTAGGTATTTCGTAA

AACAGATTATTTCATGATTATTAGTACCTATTATCTAATACTAAATGTGTAAAAAATTAT

TGGAAATTCCTAGTTATACTTTTATGGTTTTGTTATCGGCGTTCTTACTTGTAGACTTAG

AAACAATGGTATCTGTATTTTACTACTTTAAGAAGATATCAAAAAATCGTATGATAATAC

TGATGACATTTTTATTTCTCGCCTCGCTAGATTAGACATAGTGATACTTTTGCAATCATT

TTAAAAACATTACAATAGATTGACAGAGCTGTAAAAACATTGGCATTACTGTAAAGGTCT

GTGATGAAATCAATGTTGACAATCACTAATCATTTTTTTAAAATAGTAACAATCGGAATG

ATGGTCAAAGCTCATGAAAGTTGTAGGTTTTTTTGTCCTGCAATCTTCTAGGGTTTTGGC

CCTTTTTATGGTTTAGCTCTAGAAATGAGATGGCTAATAGGACTGGGATTTAGAATCACT

AGAGGTCAGATAACTTTAGTTTACGAGCTAGTGCTTGATTGTTAGAAATGGTAAGAATTT

TTTAACTAGAGATGCAGCTGACCAATATTACACCATTGGTGGTACAAGTTTGGTAGGTGA

AAAAACGCATGAAACTGTTTTACGTGAAACCTTAGAGGAAGTTGGTATTCGAGCTAAGGT

TAATCAACTGGCTTTTGTGGTTGAGAATCATTTTGATATCGATGATGTTTTCTGGCATAA

TATTGAATTTCATTATTTGGCAAGCCCCTTAGAAGACCCTAAACTAGAAATGATAGAGAA

TGCAAGCGACAGGTTTGTGAATGGATTCCAATCAATCAACTGGATCAAATGAATCTTGTT

CCTGAGTTTTTACAAACCGAGTTGGCTAAATGGCCAGGACATATTGTCCGCATTGAAGGC

TAAACTAAAGCGTATGATTTTAAATGAATAGACTGAAATAAAGGATTTTAAAAAAATGAC

ACATGAATTTACAGAAAACTATGACGTCATTGTCATAGGTGCAGGTCATGCTGGTGTTGA

AGCCAGTTTGGCCACTAGTCGTATGGGTTGTAAAACTTTATTGGCAACAATCAACCTTGA

TATGCTTGCTTTTATGCCGTGTAATCCTTCTATTGGAGGATCTGCCAAAGGGATAGTAGT

ACGAGAAATCGATGCTCTTGGCGGCGAAATGGGTAAAAATATTGATAAAACCTATATCCA

GATGAAAATGCTTAATACAGGTAAGGGACCAGCTGTACGCGCTCTCCGAGCTCAAGCAGA

TAAATCTCTGTATGCGCGTGAAATGAAACATACGGTTGAAAAACAGGCTAATTTGACTCT

TCGCCAAACGATGATTGATGATATTCTAGTTGAAGATGGACGAGTTGTAGGTGTTTTAAC

AGCCACAGGACAAAAATTTGCAGCTAAGGCAGTTGTAGTGACAACAGGAACAGCTCTGCG

TGGCGAAATTATTTTAGGAGAACTCAAATATTCTTCTGGGCCTAATAATAGTTTGGCATC

AGTAACCTTAGCAGATAATTTAAAAAAACTCGGCTTAGAAATTGGCAGATTCAAAACAGG

AACGCCTCCTCGTGTCAAAGCATCTTCTATTAATTACGATCAAACGGAAATTCAGCCAGG

TGATGACAAACCTAATCATTTTTCATTTATGTCAAAAGATGCTGATTATCTCAAAGATCA

AATTCCCTGCTGGTTGACTTATACGAATCAAACTAGTCATGATATCATTAATCAGAACCT

TTACAGAGCACCGATGTTTTCCGGCATTGTTAAAGGTGTAGGCCCTCGTTACTGCCCATC

TATTGAAGATAAGATTGTTCGCTTTGCTGATAAAGAACGTCATCAACTTTTCTTAGAGCC

AGAGGGGCGTGATACAGAAGAAGTTTATGTTCAAGGGTTGTCAACGAGTCTTCCAGAAGA

TGTCCAAAAAGATTTGATTCATTCGATCAAAGGACTTGAAAAGGCTGAAATGATGCGCAC

TGGTTATGCTATTGAATATGATATTGTTTTACCTCACCAGCTACGTGCCACACTTGAAAC

CAAGCTAATTTCAGGACTCTTTACGGCTGGGCAAACTAATGGAACTTCTGGGTATGAAGA

GGCTGCTGGCCAAGGACTTATTGCTGGAATCAATGCTGCTTTGAAAGTTCAAGGTAAGCC

AGAACTTATTTTGAAACGGAGCGATGCTTATATTGGCGTTATGATTGACGATTTGGTTAC

CAAAGGAACCTTGGAACCTTATCGATTGTTGACATCGCGAGCTGAGTATCGTTTGATTTT

ACGTCATGACAACGCTGATATGCGTTTGACAGAGATTGGTCGCGACATTGGGTTGGTTGA

TGATGAGCGTTGGAAGGCCTTCGAAATTAAGAAAAACCAATTTGATAACGAATTAAAACG

TCTGAACAGTATCAAATTAAAACCAGTAAAAGCAACCAACGATCGTGTGCAGGAACTTGG

TTTTAAACCATTGACGGATGCTATGACAGCTAAAGAATTTATGCGTCGCCCAGAAATTGA

TTATGCGACTGCAGTCTCTTTTGTTGGTCCTGCGGCTGAAGATTTAGATGCTAAAATCAT

TGAACTTTTAGAAACAGAAATCAAGTACGAAGGCTATATTCGCAAAGCTTTAGATCAGGT

TGCTAAGATGAAACGCATGGAAGAAAAACGTATTCCTGCAAATATTGATTGGGATGCTAT

TGATTCTATTGCAACTGAGGCGCGTCAGAAATTTAAGAAAATTAATCCTGAAACAATTGG

ACAAGCTAGTCGTATTTCAGGAGTCAATCCTGCGGACATCAGTATCTTGATGATTTATTT

AGAAGGAAACGGAAAAGCTCATCGAAAATATTAAATGGAAATGAAATTGTAATCTCAAAG

CAAGTGGAAGCTCTGTATTTCAGGGCTTTTTTATGGTATAATGTGTGCTTAAGAGGTTAT

AAGATGAAAAAATTTCGTTTTGAAACCATTCATTTGATTATGATGGGATTGATTTTATTT

GGACTACTGGCACTTTGTGTCAGTATTATGCAATCAAAAATATTAATATTATTAGCTATT

TTTCTCGTTTTGCTATTTGTTGTTGCTCTTTTATGGTATCAAAAAGAAGCTTATCAATTA

TCAGACTTAGCTCATATTGAGCTCTTAAATGAACAAACAGAAGATAATCTAAAAACGCTA

CTTGATAATATGCCTGTTGGAGTAGTTCAATTTGATCAAGAGACCAACGCTGTAGAATGG

TATAATCCTTATGCAGAATTAATTTTTACAACTGAAGAGGGTTTTATACAAAACAGTTTG

ATTCAGCAAATTATTACGGAAAAACGTCGTGAAGATATTTCTCAAACATTTGAAGTTTCT

GGTAATAAATACACTTCTTATATTGATGTCTCATCAGGAATTTTTTATTTTTTTGATAGC

TTTGTGGGAAACAGGCACTTAGCTGATGCTAGTATGTTAAGACCGGTTGTTGGCATTATT

TCGGTTGATAATTATGATGATATCACTGATGATTTATCAGATGCAGATACCTCAAAAATT

AATTCATTTGTAGCTAACTTCATTGATGAGTTTATGGAGTCAAAACGGATTTTTTACCGT

CGTGTTAATATGGATCGCTACTATTTCTTTACAGACTTTAAAACATTAAATGATTTGATG

GATAATAAATTTTCTGTCTTAGAAGAGTTTCGTAAAGAAGCTCAAGATGCTCAGCGCCCT

TTAACACTCAGCATAGGAATTTCTTTTGGTGAAGAAAATCATAGCCAGATTGGACAGGTA

GCTCTTGAGAATCTTAATATTGCGCTTGTTCGTGGTGGTGATCAGATTGTCATTCGTGAA

AATGCGGATCATACGAATCCAATTTATTTCGGGGGAGGGTCTGTTTCTACAGTTAAACGT

TCAAGAACACGTACCCGTGCTATGATGACAGCTATTTCAGGTCGAATCAAGATGGTGGAC

AATGTTTTTATTGTCGGACATAGGAAACTTGATATGGATGCACTAGGTTCAGCTGTTGGT

ATGCAATTTTTCGCAGGTAACATTATTGAAAATAGTTTTGCCGTTTACAACCCAGATGAG

ATGAGTCCAGATATTGAAAGGGCTATTGAGCGATTGCAGGCTGACGGAAAGACTCGTCTA

ATCAGTGTTTCTCAAGCAATGGGTTTAGTGACTCCAAGATCACTTTTAGTAATGGTTGAT

CATTCTAAGATATCTTTAACACTTTCTAAAGAATTTTATGAACAGTTTCAAAATGTTATT

GTAGTTGATCATCACAGAAGAGATGATGATTTTCCTGATAATGCTATCTTAACTTTTATT

GAAAGTGGAGCAAGTAGTGCTGCGGAGCTCGTCACTGAGTTAATTCAATTTCAAAATGCT

AAAAAACGTTTAAATAAGATCCAAGCCAGTGTTTTAATGGCAGGTATTATGCTTGATACT

AAAAATTTTTCTACGCGAGTGACAAGTCGTACCTTTGATGTCGCTAGTTACCTAAGAAGC

AAAGGAAGTGATAGTGTTGAAATTCAAAATATATCCGCAACAGATTTTGAAGAATATAAG

CAAATAAATGAAATTATTTTACAAGGAGAACGTCTTGGTGACAGTATCATTGTAGCTGCA

GGAGAAAAAAATCATCTTTATAGCAATGTTATTGCTAGTAAAGCTGCAGATACGATACTG

TCAATGGCTCACGTTGAGGCTAGCTTTGTATTAGTTGAAACAGCTTCTCATAAGATTGCT

ATTTCAGCTAGAAGTCGTAGTAAAATCAATGTTCAACGTGTGATGGAAAAATTAGGTGGA

GGAGGCCACTTTAATCTTGCTGCCTGTCAGTTAACGGATATCAGTCTTCCCCAAGCGAAG

CACTTATTATTGAAAACTATTAATATGACAATGAAAGAAACAGGAGAAGTAGAATCATGA

AAGTTATTTTTTTAGCAGATGTAAAAGGAAAAGGTAAAAAAGGAGAAATCAAAGAGGTCC

CAACAGGCTATGCTCAAAATTTCCTTATTAAGAAAAATTTAGCTAAAGAAGCAACTAGTC

AAAGCATCGGTGAATTAAAAGGAAAACAAAAAGCTGAAGAAAAAGCTCAAGCTGAAATAT

TAGCAGAAGCTCAAGCTGTCAAGGCTGTTTTGGATGAGGACAAAACACGTGTTCAATTTC

AAGAAAAAGTTGGTCCAGATGGACGTACATTTGGTTCCATCACAGCTAAAAAGATTTCAG

AAGAATTGCAAAAGCAATTTGGTGTAAAAGTTGATAAACGTCATATTGTTTTAGATCATC

CTATCCGCGCCATTGGTCTAATTGAAGTTCCTGTGAAATTACATAAGGAAGTAACAGCTG

AGATTAAATTGGCTATCACAGAAGCATAATATAAAAAACATCACAAGATTTGGAGGTTAT

GAGGTTGCCTGAAGTAGCTGAATTACGAGTTCAACCCCAAGATTTACTAGCAGAGCAATC

TGTTCTTGGGTCAATCTTTATCTCACCTGATAAGCTGATTGCAGTGAGAGAATTTATCAG

TCCAGACGATTTTTATAAGTACGCTCATAAAATTATCTTTCGGGCAATGATTACTCTCAG

CGATCGTAATGATGCCATTGATGCAACCACTATAAGAACAATCCTAGATGATCAAGATGA

TCTGCAAAGTATTGGTGGCTTATCCTATATTGTTGAACTAGTTAATAGTGTCCCAACTAG

CGCTAATGCAGAATATTATGCTAAAATTGTAGCTGAGAAAGCTATGTTGCGTGATATTAT

TGCTAGGTTGACAGAATCTGTCAACCTAGCTTATGATGAAATTTTAAAACCAGAAGAGGT

TATCGCTGGAGTTGAGAGAGCTTTAATTGAACTCAATGAACATAGTAATCGTAGTGGGTT

TCGCAAAATTTCAGATGTGTTAAAAGTTAATTACGAGGCTTTAGAAGCACGTTCTAAGCA

GACTTCAAATGTTACAGGTTTACCAACTGGTTTTAGAGACCTTGACAAGATTACAACAGG

TTTACACCCAGATCAATTAGTTATTTTAGCTGCTCGGCCAGCAGTGGGGAAGACTGCCTT

TGTTCTTAATATTGCGCAAAATGTGGGGACTAAGCAAAAAAAGACTGTTGCTATTTTTTC

TTTGGAAATGGGTGCTGAAAGTTTAGTAGATCGCATGCTTGCAGCAGAAGGAATGGTTGA

TTCGCACAGTTTAAGAACAGGGCAACTCACAGATCAGGACTGGAATAATGTAACAATTGC

TCAGGGAGCTTTGGCAGAAGCACCGATTTATATTGACGATACGCCCGGGATTAAAATTAC

TGAAATCCGCGCAAGATCACGGAAATTGTCTCAAGAAGTGGATGGTGGTTTAGGTCTCAT

TGTAATTGACTACTTACAGTTGATTACAGGAACTAAACCCGAAAATCGTCAGCAAGAGGT

TTCAGATATTTCAAGACAGCTTAAAATCCTAGCTAAAGAATTGAAAGTACCAGTTATTGC

CCTAAGCCAGCTTTCTCGTGGCGTTGAGCAAAGGCAGGATAAACGACCAGTTTTATCAGA

TATTCGTGAATCAGGATCTATTGAGCAGGATGCCGATATTGTAGCCTTCTTATACCGGGA

CGATTATTACCGTAAAGAAGGTGATGATGCTGAAGAAGCTGTTGAAGATAACACAATTGA

AGTTATCCTCGAGAAAAATAGAGCTGGGGCGCGTGGAACAGTCAAACTGATGTTCCAAAA

AGAATACAACAAATTCTCAAGTATAGCCCAGTTTGAAGAAAGATAAGAAAGAGAGACAAA

AAATGAGCGATGCATTTACAGATGTTGCAAAAATGAAAAAAATTAAAGAGGACATAAGAG

CCCATGAAGGACAGTTAGTAGAATTAACCCTTGAGAATGGACGGAAACGGGAAAAAAATA

AAATTGGTAGGCTTATTGAAGTTTACAGTTCACTCTTTATCATTGAGTATAGTGATAGCT

CTGATACTCCAGGAGCAATTGATAATTCTTATGTCGAATCATACACCTATTCAGATATTT

TGACAGAAAAAACATTAATTCGTTATTTAGATTGATTTTTGCCTCATAGAGGCTTTTTCT

TTACAATAAATTGATATAATTATTAGCGATAGAAGATTATTCTGCCTGTATTCTTTTTAT

TTTGAAAAAATTCCTATAAAAGATAATATTTTTAGGTTAGATTGAGGTAAAAAAGAGGTC

TTCACCATGGTTAAGCAAATGATCTTAGTACTAAGGTTAAAAGATTATCGTGAAATAAAG

TTTTGACACAAGTGTAATTAATAGAGGAACTTGAAGTATCCGATAAAACTATCTTGAAGT

GAAAAAACGAGTAAAACTCCCCTGATAAATACAATATACTAGCCATTTCAGAGCAATTAA

GAGTTTCCAGTGAAGTGCTTTTGGTAGAGAGCTAAGAAAAAACAGTCAAGGTTTAAGAAC

TGCAAATTAGCATTTAAATTATTTTTTGTTAATGACAGTTGTCTTTATTACCATAACTAT

AACTTCTTATAATATAGTCTTCTTATTCATCAGTGGTGGTTCGTCTCATATCTGACCTTA

AAATATTAAACTGATCATCAGATGATAGATAGACTGACTTTACAAAACAACTTTCTTATG

GTAAAATGTTCTTTGTGCGAAATAACAGCAGAAAAATATGAAGCTCGTCAACAGGTGGTT

ATCTATTCAAGTAACCTTTGCTGTTTAGGCGAAAACCCCCTGCACGAATCAGGATTTTTT

AAACCGTTATTTCCTCAAAAAATTTAGAGGAGGACATGTAATATGTCACGTTATACTGGT

CCATCATGGAAACAATCACGTCGCCTTGGTTTATCACTTACAGGCACAGGTAAAGAATTG

GCACGTCGTAACTACGTTCCTGGTCAACACGGTCCAAACAACCGTAGCAAACTCTCAGAA

TATGGTTTGCAGTTAGCTGAGAAACAAAAACTTCGTTTCTCATACGGTTTAGGTGAAAAA

CAATTCCGTAACTTGTTCGTTCAAGCTACAAAAATCAAAGAAGGAACTCTTGGTTTCAAC

TTTATGGTTCTTTTAGAACGTCGTCTTGACAACGTTGTTTACCGTCTAGGTTTAGCAACT

ACTCGTCGTCAAGCACGTCAATTCGTTAACCACGGTCACATCCTTGTTGACGGTAAACGC

GTTGATATCCCTTCATACCGCGTTGATCCAGGTCAAGTGATTTCAGTTCGCGAAAAATCA

ATGAAAGTACCTGCAATCCTTGAAGCTGTTGAAGCTACTCTTGGACGTCCAGCTTTCGTA

TCATTTGATGCTGAAAAACTTGAAGGTTCATTGACTCGCCTTCCAGAACGCGATGAAATT

AACCCAGAAATCAATGAAGCACTTGTCGTTGAATTCTATAACAAAATGCTTTAATTTTAA

GATTAAACTTACAAAAAGCCTATTTAACAGGCTTTTTTTGTATCTCATAACAAATTTATC

CTGATCCCTTCATTTTACTATGGCTTCTAAAAAAAGTTATCACTAAGGCTGATGACTTGT

AAAATGATCCTTTCGTAAGGGTTACTATTTAGGTACTCGTAAGCATTTTGAGAACGAATT

GTGTCATTTCTTGAGGTGATTCTTTTTTACCTTTAGCAATCCAACTTTGACAGACACCAA

AAAAAGCATGGGCTAAGTAGATACTTCGGTATTCTTTTTCCGTTTGAGATAACTCTTCGG

TGCGGAACTTGTCCTGTAAATCAGTAGTGATGAGTAGGCGAACTTTATTGATGATAAAGG

CCTGTATTTCTTTGGTACCGTTAGCAGACAAAAGGGAAGAGAGAAGCTGTTCTCTTTGGA

GAAATTCAAATACTTCAAGGAAGGCTTGTTCTTTATTTTGATATTTTTTCTCAAAAATAT

ATTCTAATTTGTGAAAAAAGGTTTGCTGATAATAGTCAATCATTTCATATTTATCTTTAT

AGTGTGTGTAAAAACTAGAGCGACTAATTCCAGCTCTTTTAGATAATTTAACTGTGGTAA

TATCGTCAAAACTTTCGGTTTTTAGTAACATAACCATTGCTTTTAAAATTGCTTGCTTCG

TATTTTCTTTACGGTTGACCATCTACCATCTCTCCTTTTGAACACTTTTAGACATAATGT

CCAAAAGTGAAATTTTTTCTCTTGTAAATTTAAATTTAAATTGTATAATATATTTTATCA

TAATTTTTGAACAGTATGTCCATTTTAGGAGAAAATATGTTAGAAGAATTGAAAACACTT

ATTAAAAATCCAAAATTAATGATTACAATGATTGGTGTGGCCCTAGTGCCTGCCTTATAT

AATTTATCCTTTCTAGGCTCAATGTGGGATCCTTATGGTCGGGTCAATGACCTTCCCATT

GCTGTTGTTAATCATGATAAGCCTGCAAAGAGAGCTGATAAGTCATTGACAATTGGGAAT

GATATGGTGGACAAGATGTCTAAAAGTAAAGATTTAGAGTATCATTTTGTCTCAGCTAAG

CAAGCTCAAGAGGGACTTAAAGAAGGTGATTATTATATGGTTATTACTTTACCCGAAGAT

CTTTCTCAACGGGCAGCAACCTTATTAAATCCCGAACCCCAAAAATTAACTATCCGTTAC

CAAACGAGTAAAGGACATGGAATGGTCGCTGCTAAGATGGGGGAAACAGCGATGGCTAAG

TTGAAAGAGTCTGTTTCGCAAAACATTACGAAGACTTATACCTCAGCAGTTTTTAGCAGT

ATGACAGACCTCCAATCAGGATTAAAAGAAGCCTCAGCTGGAAGTCAAACACTTGCTTCA

GGAGCGAAGACCGCTCAGGCGGGGAGTCAAACACTTTCGACGAACTTAGCAGCCTTAACG

GGTGCTAGCCAACAGTTTCAACAAGGTACTGGTCGATTGACATCAGGTTTGACTACCTAT

ACAGATGGTGTCAACCAAGTCAAGAATGGGTTAGGAACATTATCAACGGACATCCCCAAT

TATCTGAACGGGGTTTCTAGGTTATCCCAGGGAGCTTCTCAGCTTAATCAGGGTCTTTCA

CAGTTGACACAAGCAACAACACTTTCTGACGAGAAAGCTAAGGGAATTCAATCCTTAATT

GTAGGACTACCAGTCCTAAATCAAGGCATTCAGCAACTAAATACAGAGCTATCAACATTG

CAACCCCCTAACCTTAATGCTGATGAGTTAGGTAATAGCTTAGGAGCTATCGCTCAAGCT

GCCAAACAAGTCATTGCTGAAGAGACTGCCGCTCAGAATGAAGAACTATCGGCTCTCCAA

GCTACTAGCGTTTACCAATCATTAACTGCTGAACAACAAGGAGAGTTAGCTGCGGCCCTC

AGTCAATCTGATAAAAGTCAGACCGTATCTGCAGCCCAAACTATTTTAAGTTCTGTTCAA

ACTTTGTCAACAAGTTTACAGTCTCTCTCTCAAGAAGATCAGTCAAAACAGTTGGAGCAA

CTTAAGGAAGCTGTTGCACAGATTGCTAATCAATCCAATCAAGCTTTGCCGGGAGCAAGT

TCTGCTTTAACTGAATTATCAACGGGATTAGCAAAGGTAAATGGTAGCTTAAATCAACAA

GTTCTACCAGGAAGTAATCAATTGACAACAGGATTAGCACAATTAAACAGGTATAATACT

GCCATTGGTTCTGGGGTAATAAAACTCTCAGAAGGTACCAATGCCTTGTCATCCAAGTCC

GGAGAATTACTAGATGGTAGCCATCAATTATCAGAGGGTGCTACTAAACTAGCTGATAGT

AGTTCTCAATTGAGTCAGGGTGGTCATCAATTAACGAGCGGATTGACTGAATTATCAACA

GGATTATCAACCTTAAATGGTTCCTTAGCCAAAGCCTCTCAGCAGTTATCGCTTGTTTCT

GTGACTGATAAAAATGCTAAAGCTGTCGCAAAACCTCTTGTGTTAAATGAGAAAGACAAA

GATGGTGTTAAGACGAATGGGATCGGGATGGCACCTTATATGATTGCTGTTTCTCTAATG

GTTGTGGCCCTTTCAACCAACGTCATTTTTGCTAATTCTTTATCTGGTCGTCCGGTCAAA

GATAAATGGGATTGGGCTAAACAAAAATTTGTTATTAATGGTTTTATTTCGACTATGGGA

TCCATTGTTCTCTACTTAGCTATTCAATTATTAGGGTTTGAAGCCCGTTATGGTATGGAA

ACCTTAGGATTTATTATGCTAAGTGGTTGGACGTTTATGGCTCTTGTCACAGCTTTGGTC

GGTTGGGATGATCGATATGGCTCTTTTGCTTCTTTGGTCATGTTATTGCTTCAGGTTGGC

TCTTCAGGTGGCTCTTACCCCATTGAGTTAAGTGGAGCATTCTTTCAAAAGTTACATCCT

TTCTTACCAATGACTTATGTGGTATCTGGTTTACGACAAACCATTTCATTATCAGGTCAT

ATTGGAGTAGAAGTGAAAGTCTTAACTGGTTTCTTACTGGCATTTATGGTATTAGCACTA

CTCATTTATCGTCCTAAGAAAACAGTCTAAATAAAATAACCTCAAAAAAAACAGCATCTA

AGGCAGTTACACTAGATGCTGTTTTTTGTCTTATTTTGACAATAACTTGACAATTTCTGT

CAAGTTTTTTATTGTCTTGTTAAGATCATTTGATAGTAATGTCTCCCTTATTGAGATTAA

CCGTTAAATTTCCAGTAGCTGCTTTATTCTCAGCCACATAAGAAATAACATCTGAAGTTG

ATTCCTTACCAATCTTAGCTGGTGTCAGCTGTGAGCCAAGGGAGACTTTACCATTTTCGG

TTCTTGCTCTGTAGGCGATGGCCTTTGCTTTTGTCAAGGTTATGTCAACATCAATATCAC

CACCAGTAATAGTATTAGTCCCTTTAAGGGTAAGGGCATGAGCGGTGAAGATAGCATCAT

TGTCATAGTAGTCGTTCTCCTCTTCTTCTTTGCTTTTAGAAACAGTGAAGGTCACATTTT

CAAGGGTTGTTCTCTCGGCTTCTATACCACCAGAGTGTAATGATTGAATATTGACAGCTT

TTAGAGTGGTATCTTTAGTAGTTAGTTGTCCATTTCCTAAGCTGATGGTCCCATTTTCCA

CACTAGAATCAGTCAGGTTTGAAAAAGAATAGTCAGCTTTTAACTCGAGATTTGTTAACT

GACTTTTAGTGGCTTCCAACATACCAGTGATAGTAGCTTTCTTGACTTTGGTATGTAAAA

GAGTAAGATTGCCATCGAATTGCATATCTTGGACAGTTAGATTTGACAAAGTGGTATGTG

GGACATTACTTCCCTTAACTTCATTGAGGCTAGTTTTTTCAGGAACAGTAATGGTAATAG

AACGGTAGTTCTTCTCTTGACGACTATTATTGAGAGTAAAACCTAAAATTTCAATTCCAC

CAGTGATGACAACGTCTTTAGGTTTTTGCGAAAGAGATAGGGTCTTACCTGTTACTGTTG

TGACGATAGGGTCAATAAATTTAGGATGTGTGTAATAGGTCACTGTTGGTCTTTGAACGG

GTCCTGTCTCTACTGTAATAGTTGAGGCAGAACTGTCAATATCAAGAGCAGTTATTTGGT

CAAATGTTTTGATGACCTTTTTGGGAGCTGTTTGGTGTTTAATGTCCTGAAGTCCTCCAG

TTGCATAGCCAGCCCCTAGAAGAATGCCTCCTAGAATAATACATAACACACTGGTTATAA

AGCATTTGCGTGAGATATTTTTCATGCTTGAGCTCCTTTCTTATTACTGATCCACTTAAT

AAAGGCAACAAAAGCCCGTCCTGAAAAACGAAGGATTGCTGTGGCAATAATCCAAATAAG

AAGACCACCACCAAAACACAACAAGGCGCTGCCGATGCCCATAGCTTGTGCAGGAATAGA

TGAGCCAATCAAGGTCAAGCTTTCAAACAGAGTCACTCCTGCGACAAGAATACCTGCAAG

CCCAAAAATGTAGGCGGCAAAGATAGCTGTGAGCCCGAGCATTAGGCAGGCAATAATAAA

GAGAATCAACGCCAGCAATACGGGTAAAGCAATCGGAGCAGCAAAGAGGGACAAAATGGT

TAATCCAATAATTGTTGCCCGATTTTTGGGAGTTTTCTCGGGAGTTGCCAAGTGTTTTCC

TAGAACATTTGAAATGATTTCACTGGCAGCTTCCTTAGGATTTCCTAAATCGTCGATAAC

CTGGCTTTCTTTGTCAGGACCAGCTTCATCAAAATATTCAATATAATATTCCATCGCTTC

ATGGTAGTCTTCTCTAGGTAGTTTGCGCAAGTATTTATCTAGTTCTGCTAGATACTCAGT

CCTTGTCATGTCTTAAGCTCCCTTCAATAATCCCGTCTAAGGCAAATTTATAGCTCTGCC

ATTCTTTTTTTAGAAAAATCAGCTGTGCTCTCCCCGAAGAAGTGACAGCATAATATTTAC

GCTTTCGTCCCTGATGTTCTTGACTATAAGTTGTCAAAAAACCTGCTTTCTCCAATTTTT

TAAGAATAGGATAGAGAGTGGATTCTTTGATATTAGCCACTAGTTTAATGGTTTGACTAA

TATCGTAACCGTAAGAATCATTTTTTTCGACAATGGCTAAAATCAGAAATTCAATCAAGG

TAGCCGATACTGGAAAATACATTTTAGGCTCCTTTCATATATAAAAATTTTATATATAAT

TATTTTACATATTTAGTATAAGCTTAGTTTTTTGATATGTCAATATTTTATATATAAAAA

AATAACATATATATCAAAAATCACTAACTAGGAGTAATAAGTTGACCAAAAAGCTAGGAT

TGGTTATGATGAAGATAAGATTAACGGAAAGAGAAATTAGGGAAGGCTATGAAGGACTTA

TTAAAACAGTATCAGGCAAAACCACTAGGAGAGGAGAAGCGGTACGCCGTATTTTTACCT

CTTATTCTTGTCAATGATGACTGGCATGTCTTATATGAAGTCAGAAGTCAGCATATTTCA

CAACCGGGGGAAGTTTCTTTCCCAGGGGGACGAGTAGAAAATCAGGAGACACTTCAAGAA

GCTGCTATCCGAGAAACAGTTGAGGAATTGACTGTTGATGCGTCCCAGATCCAGCTATGG

GGTGAAATTGATTATTTAGTACAGTCTTCTAGAACTATTCATTGTTTCGTTGGTCAATTA

GTCGTTGATGACTGGGAAAGCATACAGCCTAACGAAGAAGTTGACAAGATCTTTACAGTA

CCTTTACGTCAGCTACTTGTCACTGATCCTGTCTATTATCATTTAGAAGCCACTCCTATT

GAGACAACGGATTTTCCTTTTGACCGTATTCGTAATGGGAAATACTACCAATTTAGTCAA

GAATACCGTAGTATTCCGTTTTATGAAAATCTAGAAGAAACCATATGGGGAATGACTGCC

CAGTTTACTAAGTGTTTAACCGATATTCTAAATGATGAATCAATTGCAAAGAAAAACCCC

TTAGATATTGAAAAATCTAAGGGGAGATAGAGCACTAGTTTGTTGGCGTCAACTTTTTGT

CAACAACCTGTAAAGGTTATTTGTATTTTTCTTCTAATTGACTCATCCAAAGTCCGAGCC

AAATACCAAGAGCAAATAACACAACGGCCATAAGCCAGGTTAAGATACCTGCATGACTGT

AGGAAATAGATTCGGGACTACTACTATTTGGAATAAGAATTAATAGCGTGCTGGATAATA

CAATACCAATGATAAAGTGGTAAACTTTTGAATGATGGTGCTGTAAAGCATAATCCATAC

TTTTAGAAAAAGCCAAGATGGCTAGGACTCCTCCGATAGCAATAGGTAAGAAAGTCCCTA

ATAAATCCAAAGATTTGAATCCTATTAACATAGGACCATAGAGTCCTAAGATAAGTAAGA

GATTTGACGGGCTTAGTCCAGGGACCAGTACTCCTAAAGCAATTAAGGCACCTGCTAAAA

TAAAGGTTAAAAAGTTAGCTGGTAAGGTACCGATTAAGTCATTTAAGAAGTAAAGACCTA

GTCCTGAAATAACAAAGGTAAGGACTAGCCATAGCCAGTCAACTTTGTCTCTTTGAGACT

GCTTGGTTGACTCCTTAATCAGACTAGGAATAGTGCCGACAATAGCGCCAGCAAATCCCC

ATAAGACGATAACTTGATAGTGCTTAAGCAAAAACTCAACGGGGAAAGAAAAGAGAGCAA

TACCTAAAATACCACCAATTCCTACTGGTAAGAAAAATAAGACATTTTCAATAAAGTTAT

CCCTCATATGAGCTAAAAAGGAAATCATTCGCTCATAGATTCCTAAAATGGCTGCGAGAA

CACCACCGGATACACCAGGTAAAATAAAACCTAGTGCAATAATCATGCCTTTAAAAACTC

TTGAAATAAATGAAACCATTGTTGCCTCCTGAAAAGAATGATTTTATTGTATCATAAATT

TAAAGAATTGCTGACACAAAGCTGCATATCCCTACTAAAATAATTGTTCCTAATAAATAA

TACATTCAATTTTGAAGATATTTTCCTTAAACATCATGTCTTTGTGATCAAGTCAAGTTT

GATTGTCAAAGTTGTAAATAAAATTTACAAAGACTGTAAACTTTACGATAATTATCAAAA

AGATAAACGAAAAAAAGCTTAGGTCAAAGACTTTTGATAGTAAACTTATTGAAGAAGGAT

TATAGTAGCTATTAAAAAGGAGATTTATTATGGGCTTTAAAAACCAGTTACCTTATTATT

GAAAAAAATTACAGATGTCACAAGAAGAGCTTGCTGAAATGATGTATGTTTCAAGAAGAC

TATCTCAAACTGGGAAAACGGTAAAAGCTATCCAGACCTGCAAAATCTTTTGATGTTAAG

TAAGTCCTTACAGGTTCCTTTAGAGCAACTAATAGCAGAAGATATCCCAAAAATGACTTT

AATTATTAAGGATGAGGAGATTAAACAATGTGATAGGAATACGCATATTATGTTATTTGG

GACACTCCTAATTGCTATCAGTATTTATCCTATAATGTCTTGGTTAGGTTGGTGGGGATA

TGGTCCGGTTGCTATCTTATGGTTGATAATCATGCGTAGCGCCTTAAGAATTGAGCGATT

TAAAAAGACATATGATGTTCAGACTTACCGATAACTTGAAGCTATAGCAAAGGGTGAGAG

CTTAGATCAGATTGATAAGATTAAAGAAAAAGCAAAATATCCTTATCAAAAAGCATGGAT

TGTTATTGCGTACACAGGATTGACAGTGTTAATAACCCTACTCTCTATTTTGTTTTTTCA

TTTGTTTTTTTAAACACGAAAAAAGCCTTTGAAACGTTCATTTCATAGGCTTTTCTCATC

TCAATTATTTTACTTCTGTAAAGGTTAAGTATTTTCTATTTTAGCTGTTATAAACCTTAT

TGTATCAATATTTATTGATAGTTGCTTAATAAAAGGTAGAGTGAATGGTAGAGTAGTTAT

TGCGTTAGTTCAGCAATCTTTTCAAGATAAATAAATACCCACAGCTTCTAACTTTCGTTG

TCGTAGTAACCCCAATAGGTCATGATAGATAAGAATATAAAAAAGGGGCTGTCTGGCTTT

TGCTTAACGCAAGCCACAGCCCCAAGGAATTAACGCTTTTGACCAATAAACCACAATTGG

TTACGAACTCGAACAGGAGAAGAGAAATAATAGTCTGTGTGTTGGATGACAATCTCTTCT

TTCAGTTGTACTCCCGTATCCTTTAGGATTTTTTGCCCCTGTTGGTCTCTAGGAACTTTT

AGAAATACAGTAATGGCATCTTTTCGTATATCTACGATACACTTACGTACAGCTCGGTTA

AACCCGTTATGGATTGGATTGATAGTGGTAACGGTCTGGTGTTCAAAGTTGCTTACCTTC

TGTACTCGTTCACGCTGGAAAAGGAAGTGTCTTAATAGAAAGGTGCGATAAATAGATTGG

CAGTAGTTCCCAAGGCTATCTGTATTCAAGCGTTGTATCATCTTTAATCCAGATAATAAG

GCTAGAAGTAAAGAAATAAGAGCGAATCCATAACATACAGAACTAATGGTATCAGTAACG

TTTTTAACCGTATCCAGATTGACAAATGTGACGGTAGATAATTGAACTGAAACAAGATTG

AACAGCCAACCGAACACATAGCAGATTGGAGCAATAAGCAGTAAGTATAATATGAGATTG

AAGCTTCTCATGAAGTAGGTTTTAGTGGTCATAGGATACCTCGCTTAGTATAGTAGGTTG

GACAGAGTAAGGGTAGAACTTGATAGGAGTGTTCATTATCGACAACTTGAATGATACCTG

TTCCGTGTCCTGTTGGAATGACAATCCCCTCTGGATCTAGGTCTGGAAATAAGAATTGAG

TGGTTTTCTGATTGATATTTCCAATTTGCAATAATACGTTTAGCTGTTCCCTCACTGAAA

TGGGAATAGTGTTATGGTCAAAACGCTGGCTTCCCAAGAATAGATGGATTTTAGTAGCAT

GTCCCAACAAGGCAATTTGTGATAGTAGGGAGAAAAAAGCTTCTCTGATATTCTTATTGA

CTCCCTCTGATAGAGCGAGGACTTCATCAATAACAATAGTTAGATGGGTAAATTGATGAT

TGGGATTATCATACAAGATAGCTTGTCGTTTCTGAATGAGAGTTGCACACTGGTTTAATT

GTTCATTGACTTGTGAAACGAAATCAGATTTTGAATGGTTTTCTACTGGGTGGATAACGG

CAATTTTATTTTCTCTTGCCCATTGGCTAGGGGTATCGAATTTTGGGTCAATGATAATTA

GGCCGGACATAGGTTTCAGGACACTCAAGAAGTAAGTGAGAGCGTACGGTTTCCCAGAAC

CTGAATTTCCAGCAATAGCCCAGTGACTCCCCTTATCTAAATTGAGTTCGAAGTGCTTTA

TGACAGGAATTTTCCCCTGTTGTAAGCTAGATGAAAATTTATCTAAATCAGGAATCGCCA

AGCGTTCTGAAATCCCCTTTCTCCAAGGGAAGAATAATCCACGTTGAATGTGTAAGTCAT

CCGTCTCTAAAGGGACAAAATCTGTGCAAATTTTGATTTATTTATTTTTTGACTTCCGTT

TGACTTAATCGATAAAGTTGACCTTGCTTTTTTGGAAATAACTAATGGGACGGACGTAAG

GTAAGATTTTGGGATATTTTCTTTATACATTCGTTCTAGAATATCATCAACCAGGAATCC

GATATTAAATAGTGGATTTTCTTTGAGTTTTTTTATTAGGTTAGGGATAATTTCTGTCGA

AACATTATCATACCACCATTTATTGACGAGGGGATCAATGTTTTCTTGCCTAAATGTTGA

GGGTATGCCATTTCCGTCTAATTCAAATAGGATACCACAGACATTAACTTCAAATGATTT

AATTAAATTATCCCCTAACATCAACCAAATAATATCAAACATTTCTTCAAAATTAAAGAC

AGTCAATTCATTGATGGTACTGGTTCTAAAACTTCTAGCGAGGTTATAACTTTTTTCACC

AACAGTAATGTCAATGTCATCTTTCTTGTGAAAAGTATCCATTTCAGTTTCATCAGAAGC

CAAAAAATTTTTTCTTTGAATATTGAAATTTGCGAATGTCTTGGAAAGTCTAAGACAGCT

GGATTGTATTGAAATTAAGTCATCGTCCGAAAAAGAATACATTCTTTTATCTACAGTTTC

CAAATCTCTAAATAAAATAGAGCTGAAGTAGTGGTAAAACATATTTTCAACAAACTTGGC

AATATCTCCAAAAATAATTTCTTTATAGTCCGAGTCGGTTTGAGTATTCAAATAAAGCAA

TATTGAAGCGGTAACTTGTCGCTTTCCCTTTTCAACTTGGCTAACTTGACCTGCATTACC

TAGGTCACTTTACTTTAAATTATACTTTTTTCTTAAACATTTGATACGAGTAGGTATTTC

TTGTGAGTAATTTTCATCAAAAAATTTCATAGATAAGCCCCCTATAAACATGGAAAAGTG

GTAAAAATAGTTGTTATCCTAGTTGATTAAAAAAATCTCTGATTTCCTCATCTTTTATAA

AATAATATATAATTTTCCCCTCTCTTCTAGTGTCCAAGATGTTTTGATTGGCTAGTTTAC

GAAGATGGTGGGAGGCAGATGCCATACTGAGATTTAGTAAACAGGCTATATCGCAGACAC

AGAGTTCTTCAACAGTAAGGAGATAAAAGATGATATTTATCTGTTTATTATCGGTAAACT

TTGATAAAATACGAAGTGATTTTTGGACTTTTTCCTTTTCAAGGTAGTTCGTTGCGGTTG

TAACATTTTGTTGATTTATAACATTCACTTGGCAGATACTATCTTTTTTCATAATATTTT

CTCCTAGCCTAACACAGCCCATAGCATGTCAAAACTGTTGTTTTCAATCAGGATATATAT

CCCCAATCCTAAATAAACAACGGCAATAAACCATCTGCTATATTTTTCCAAAATTTCTCC

AACAGAAGGGACTTGTGCCAATTTTTGGGCAGAAAAAACCAAGAGATAAATCATGACTAG

AAAGGTAAGTAAAGCCACTATCAAATTCGCTAAATTTAAGGTAGTAAAATACGGGACAAA

GACACCAATATTGTCAGCACCACAACTTGCAAAAGTAATCATAGCGACTAGAAAAATCAG

GTTTTTATTATCTTTGCGCAAACCTTCTTTGGCAATAGCTTCTCCATCAGAATCTCCTAA

AAGCAAAACTTTGAGGCCTAGGAAAATTGGAATCAAACCGAGCAAACCTAAAATCTCTTT

ACTAGGAATATAATCTAAGACAAATGCAAAAAGCAAACTTAGCAATATTAGACTAACAGA

GCCTAGAAATTGTCCTAAATAGATGTTAATGATGTCTTTTCTGCTTTTTCTTTTGGCAAA

AAATAACATTAGGATAATAAGTAAGTCTACGGCTGTCCCAGAATACAGGATTATTGAAGT

AACAACATTTTGAATCATAAAACACCTCATTCAAATATATTTTTGAATGAATTTTAACAT

TAAACTTTGTAGATGTCAATTTCAGCTCCACCAAAATATAGATAAGAAGTTAGTGTACCA

AATATAAAAAAGCCCTGCCATCCAAATGATAACAGGGCTTAACTTCAATATCCAAATGAT

ATATTCATCTAAAAAAGGTAGAGTAAAAGGTAGAGTTCTTATTGATTTATATTACAATGT

AATGAACTTCAAAAAATTCAAAAATCGCTTAAAATCAATGTTTTGACGTCTAATGACGTG

TAGTGAACCCCTTACTTAACTTCTGTAAATGTAACGTGTTTACGTAATTTTGGTGAGTAT

TTTTTCAATTGAAGACGGTCTGGAGTGTTACGTTTGTTTTTTGAAGTAAGGTACAAGCGT

TCACCAGATTCTTTATGTTCAAGTGTAATATTTACGCGCATGGTATCTCCCTTCTATTAT

TTAGCTTCGTTAGCTTTAGCGATTTTACGTCCTTTGTAGTATCCTTTAAGGGATACACGG

TGAGAACGTGAGTAATCTCCAGTAGTTTCGTCGAATTGTACAGATGGAGCTGTCAATTTG

TAGTGTGTACGACGTTTGTTTTTCTTCGCTTTTGACGTGTGACGTGCAGGTACTGCCATT

TTCTTGTTCTTCCTTTCGATGTGTAAGTTTTGATTTTCATCAACTTCACTATGATAACAT

ATCTTTTTTGTAAAGTAAAGTTACTTGACAAAACTTTTTACTTTTACAATTTCATGGTGA

AAATACTATCAAACTAAAGCAAATGATATATGCTTGTTTAAAATATAAGAAAAAATGGTA

GAATGTTTAAAGAGAAAACTAATCAAAGAGGTAAACTTATGAAACTTCAAAAACCAAAAG

GGACTCAGGATATTCTGCCTGGGGATGCTGCGAAATGGCAGTATGTGGAATCTGTTGCGC

GTGATACGTTTAGCCAATACAATTATGGAGAAATTCGTACTCCTATGTTTGAACACTACG

AGGTAATCTCGCGGTCTGTAGGTGATACGACTGATATTGTGACGAAGGAAATGTATGATT

TCTATGATAAAGGAGATCGTCACATTACCTTGCGACCAGAGGGTACAGCTCCAGTGGTGC

GTTCTTATGTTGAAAATAAATTGTTTGCGCCAGAGGTCCAAAAACCTGTCAAACTTTATT

ATATCGGTTCCATGTTCCGCTACGAACGTCCTCAAGCTGGTCGTTTACGTGAATTTCACC

AGATTGGGGTAGAATGTTTCGGCGCTGCTAATCCAGCAACAGATGTTGAAACCATTGCGA

TGGCCTATCACCTCTTTGAAAAATTGGGCATTAAGGATGTCACCCTTCATTTAAATAGTT

TGGGGAGTCCTGAGAGTCGTGCAGCTTATCGTCAAGCCTTGATTGACTACTTAACACCAA

TGCGTGATCAATTATCTAAAGATAGTCAACGTCGCTTGGATGAAAACCCACTGCGCGTGC

TTGATTCTAAAGAAAAAGAAGATAAACTAGCCGTTGAAAAAGCGCCTTCTATTTTGGATT

ACTTAGATGAGGAAAGTCAGGCGCACTTTGAGGCGGTCCAAGATATGCTTGAAGCACTTG

ACATTCCTTATGTGATTGACACCAATATGGTGCGTGGCCTTGATTACTATAACCACACTA

TTTTTGAATTCATCACGTCTGTGGAAGGTTCTGACTTAACTATCTGTGCGGGTGGACGTT

ATGATAGTTTGGTAGGTTATTTTGGTGGGCCTGAGACTCCAGGATTTGGCTTTGGTCTTG

GTTTAGAACGTCTCTTGATGATTATTGAAAAGCAGGGCATTACCTTGCCAATTGAGACAG

AGATGGATATTTACCTTGCTGTGTTAGGTGATGGTGCTAATAGCAAAGCTTTAGAATTGG

TTCAAGCCATTCGCCGTCAAGGGTTTACAGCTGAACGAGATTACCTTGGCCGTAAGATTA

AGGCTCAGTTTAAATCAGCTGATACCTTTAAAGCAAAATTGGTAATGACTTTAGGAGAAA

GTGAAGTTGAAGCAGGCAAAGCTGTGATTAAGAATAATCGTAGCCGTCAAGAAGTGGAAG

TCAGTTTTGAAGATATGATGACCAACTTTGAAAACATCTCAGAGCAATTATTGTCTTAAT

GATTTTATTGTCGTCATAAGCTTGTGTTGATATGATTGTTTAGAAGATAATCACTAAAAC

AGGTGTCACTTCTCTTTTTAGAAGGGAGTTGCACCTGTTTGTTTTCTGTAAGGGGATTGA

TTAGCTTTCTTAGTATTCTGTGAGGACACCTGATATGTCAAAATGCTGACGACTCAGTAT

TCTTGCTTTATCTTTAGAAAAACTCTTTTTAAATGGGTGGGGTTTTTAAGGGCTTTTTGC

TATAATAGTAGGGAATGAATTAATTGATTTATATAGTGAAAGGAATTGGAAACGAGCAGG

TTTCACAGGATTCTGTTTGTTTTAGGGAATTTATTTATGAAACGTAGCATGTATGCAGGG

CGTGTTCGCGAAGAACACATTGGAACGACAATCACATTGAAAGGATGGGTCAGTCGTCGT

CGTGACTTGGGTGGTCTCATCTTTATTGATTTACGTGACCGTGAGGGTGTGATGCAATTA

GTCATTAACCCTGAGGAAGTTTCCAGTGATGTGATGGCAACTGCTGAACGCCTACGTAGT

GAATATGTGATTGAAGTGGAAGGTTTTGTTGAAGCTCGACAACAAGCCAATGATAAGTTG

GCGACAGGAATGGTTGAGTTAAAAGTATCTGCTTTGACCATTTTAAACACAGCTAAAACG

ACGCCTTTTGAAATTAAAGATGACGTTGAAGTAAGTGATGACACGCGTTTACGTTACCGT

TACTTAGATTTACGACGTCCAGAAATGTTAGAAAACTTCAAGCTACGTGCAAAAGTGACT

CATTCTATCCGTAATTACCTTGATGACCTTGAGTTTATTGATGTCGAGACACCTATGTTG

ACAAAATCAACTCCAGAAGGTGCGCGTGACTATCTGGTACCAAGTCGTGTTAGCCAAGGG

CATTTCTATGCTTTGCCACAAAGTCCGCAAATTACCAAACAGTTATTGATGAATGCTGGC

TTTGATCGCTATTATCAAATTGTGAAATGTTTCCGCGATGAAGATCTACGTGGTGACCGT

CAACCTGAGTTTACACAAGTTGACTTGGAAACGTCATTCCTTTCTGAACAAGAGATTCAA

GACATCGTTGAAGGCATGATTGCTAAAGTGATGAAAGAGACAAAAGAAATAGATGTAACC

CTACCATTCCCACGTATGAGCTACGATGTAGCAATGAATAGCTACGGCTCTGATAAACCA

GACACACGTTTTGAGATGCTTTTACAAGACTTGACAGTAACTGTCAAGGGAATTGACTTC

AAGGTTTTCTCAGAAGCACCAGCTGTCAAGGCGATTGTGGTGAAGGGAAATGCAGATCGA

TACTCTCGTAAAGATATTGACAAATTAACTGAGTTTGCCAAACAATTTGGTGCTAAAGGC

CTTGCCTGGGTTAAGGTGACAGATGGTCAATTGGCAGGCCCAGTAGCCAAGTTCTTGATA

GCTATTGAGACAGAGTTGTCAAGCCAATTAAAACTTGCTGAGAATGATTTGGTTTTATTT

GTAGCGGATACTCTTGAGGTTGCTAATAATACATTAGGTGCCCTTCGTAATCGTATTGCC

AAGGACCTTGACATGATTGATCAGTCGCAGTTTAATTTCCTTTGGGTGGTTGATTGGCCA

ATGTTTGAGTGGTCAGAAGAAGAAGGACGTTATATGTCAGCTCATCACCCCTTCACTCTT

CCAACGCCAGAATCTGCTCATGAGTTAGAGGGTGATTTGGCTAAGGTACGGGCAATTGCT

TATGACATCGTTTTAAATGGTTACGAGCTTGGTGGTGGTAGCCTGCGTATCAATCAAAAA

GAGATGCAAGAGCGCATGTTCAAGGCTCTTGGCTTTACCGCTGATGAAGCTAACGACCAA

TTTGGTTTCTTACTAGAAGCGATGGACTACGGCTTTCCTCCTCATGGTGGCTTGGCAATT

GGTCTTGACCGTTTTGTCATGTTGCTTGCTGGAAAAGGCAATATTCGAGAAGTTATTGCC

TTCCCTAAAAATAACAAAGCCTCTGATCCAATGACGCAAGCACCTAGTCTTGTTTCTGAA

AACCAATTAGAAGAACTCAGTCTTCAAATAGAAAGTCATGATTAAGAAAACAACCTATAG

GAAAAAAGTTAAATATGTCATTAGTAGAGGGGCCAAGAAAGTTGGCCTACTCCACGCTCT

AAGAAGTATTTCAAGAGAAAAATATGCAGAGAAGATTTCGGCTTCTCTGCTTTATGGCAT

TCTCTCTAGTATTGCTGTGAATTTTTTCTTCCAGCCTGGGCATGTTTATTCAAGTGGAGC

AACTGGTCTAGCACAGGTTTTTTCAGCTCTTAGTCATCGTCTTTTAGGCTATGATTTTCC

CATCGCCTTTGCGTTTTATTTGATTAATATTCCTTTGCTTATTTTAGCTTGGTATAAAAT

TGGGCATCAATTTACCATTTTTACCTTTATCACAGTCAGCATGAGTTCTTTCTTTATTCA

AATCATGCCTCAAGTGACGCTGACGACTGATCCTCTTATCAATGCTATTTTTGGTGGTTT

GGTTATGGGACTGGGAATTGGTACAGGTCTCAAATCACGTATCTCTAGTGGGGGGACTGA

TATTGTCAGTTTGACCCTTAGAAAACGAACAGGCAAGGATGTGGGCAGTCTCTCATTGAT

GGTTAATGGTGCAATTTTAGCCTTTGCAGGGATTTTATTTGGCTGGCAGTACGCCCTTTA

TTCTATGGTCTCTATCTTTGTATCAAGTCGTGTTACGGATGCCATTTTCACCAAGCAAAA

GAAAATGCAGGCAACTATTGTTACCAGCCATCCAGAGCGTGTGATTCATATGATCCATAA

ACGTCTGCATCGCGGGGTGACCAGTATCAACGACGCAGAAGGGACTTACAAGCATGAACA

AAAAGCAGTTTTGATTACCATTTTGACATGTGAAGAATATCCAGAATTCAAATGGCTGAT

GTTAAAAACAGACCCACAAGCCTTTGTTTCAGTGGCTGAGAATGTTAGAATTATCGGTCG

TTTTGTGGAAGATGATTAAGAAAAACACAGGAGGTGTCATGTCAATTGACCAACGTTTAC

AGTTAAAGAAATTTTGCTTTATTATTTTAGGAGCTGCTATCTACGCCTTTGCTTTTGTTT

ATTTTTACATGGCTAATCGCATTGCAGCCAATGGTTTAGCTGGTCTCACCCTAGTTGGTA

AGGCACTTTTTGAGATTGATCCTTCTTTGATAGGCTATTTGATTAATTTGCCTTTGGTCT

TGTTGGGAGCTAGATGTTTTGGGAAAAGAGCCATGGTTTACACAGTAATGGGTATTTTGT

CACTTTACTTTTTTGTGTGGCTGTTTCAACGCTTTCCTTTGGTCGTTGACTTGGACCATG

ACAATTTAGTCGTTTCCTTGATGGCTGGAATTATTGGTGGTCTAGGTGGCGGTCTTGTGT

TTCGCAATGGTGGAACCATCGGTGGAGCAGATATTATTGCTAAGTTATTAGAAGACAAGT

TAGGACTACAGCTGAATCAAGCTCTTTTAGGCATTGATCTTTTTGTCATGATTGTCTCTT

TGACTTATATTTCCCTGCCGCAAATGATGTATGCTCTGATTGCTAGCTTTATGTATGGTC

AAATTGTTCGCTTGGTACAACAAGGTGGTTATTCAGCCCGTGGCATTTTTATCGTTTCAG

AACAAGCGGAGAAAATTGCCCGGTTCATCATGGACGAATTGGGAAGAGGAGTTACTTATC

TAACAGGTGAAGGAGCCTATTCTGGTCGCCGTAAAAAAGTGATTTATGTGGCTTTGGGGC

GAAGAGATATTAGGGAATTAAAAGCTTTTTTAACTCAGGTTGATCCCAATGCTTTTATCA

CTTATTTTGATGTTAATGAAGTCAATAGCCCAGAATTTTTAACTATTAAAAGCAAATACC

ATAAAAAGAGGAAATAAATGAGCATTAGAGTTGCCTGCGAAGAATAGTGAGGATAAAGGA

TGGCACATCACGATAAATTGACTAAACTACTAAAACTGTTTTTGATTGCCCTAGGTGTAG

CCATTTATACCTTCGGTTTTGTTAATTTTAACATGGCTAATGCTTTGGCTGAAGGTGGTG

TGGCAGGAATAACCTTGATTTTAAACGCGCATTTTGGGATTAATCCTGCTTACTCTTCTC

TCTTATTTAATCTTCCACTTTTTATTTTAGGGGCAAAGATTTTTGGGAAGCGTTCTTTGG

CTCTAACCATCTATGGAACAGTTCTGATGTCCGCTTTTATCTGGATGTGGCAAAAAGTTC

CTATCGAACTTGGCTTGGAAAATGACATGATGTTGGTGGCTGTAGTGGCAGGTCTCTTTT

CAGGGATTGGGAGTGGCATTGTTTTTCGCTATGGTGCAACCACAGGTGGAACGGATATTA

TTGGTCGCATTGCAGAAGAAAAATTTGGAGCTAAGCTAGGTCAAACTTTGCTTTTGGTGG

ATGCTCTTGTATTGACAGCTTCGTTGACTTATGTGGATTTAAAACACATGCTTTATACTT

TGGTGGCAAGCTTTGTTTTTAGCCAAATGATTAGTGTGGTTCAAAATGGTGGTTACACTA

TCCGTGGAATGATTATTATTACCAAGCATTCAGAGGCTGCCGCTCAAGCTATCCTAACCG

AAATCAATCGTGGAGTGACTTACTTGAAAGGTCAAGGAGCTTACTCTGGTAATGATTACA

ATATCATGTATGTGACCTTGAACCCAACTGAAGTTCGGGAAGTCAAACGTATTTTAGCTG

GTTTGGATCCAGATGCCTTTATCTCCATTATTGATGTGGACGAAGTTATTAGCTCTGATT

TTAAAATTCGCCGAAGAAATTATGATAAATAAAAATACAGGTCAAACAGCCTGTATTTTT

ATTTTGGTCCCCCACTTAAGAAACTACCAAGCATAATAGCTGATATAGCAGTTCCCTTGT

AATGGCAAGCTTTTGTTTTTACCAAGCGACTAAGATCAAATATGCTTTTAGGAGCATCAA

ATTGATGTGTTAAGATGTATTGATTGATAAGTGAGTCAATTTTTGTAGCTAGTAGTGAGC

TCATCCCCTCTTTTTCAAGTTGACTGGCGAGCTCGAAGATCGCTTGACGAAAATAATCAT

CTGCTTGTATTACTTGTTCGCTATAAACTTTACTAAGAGCATCTAATATTTCTTTTTCTG

ATGGCATGGCATTCTCCTGTAACATATTGTGCTATTATTATAATATAAAAAACTAAGACT

GTCATAAATGTGACAGCCTTAGTGATGTTATTACTTACATTTCATTTGGTGCGTCAACAC

CGAGTAAGCGAAGCGCTTCTTTAAGAACAGTAGCAGTTGCATAGCAGAGAGCTAAGCGGT

TATCACGTTCTGAGTTGTCGTCAAGAATGCGAGTGTGAGCATAATATTTGTTAAAGCTTT

GGGCTAAGTTAATAGCAAATTTAGCCATAATAGAAGGCTCAAAGTTATCTGATGTTCGCT

TGATAATGCGTGGGAAATCTTGGATAAGTTTGATAATTTCCCAGCTTTCAGCATCAGCTA

AACTGTAAGTGGTGGTTGCAGATGGCGTAAAGTCAGCTTTACGTAGAATAGATTGGATAC

GAGCGTGAGCGTATTGAACATAAGGTCCAGTTTCTCCTTCAAAGGAAACCATGGTTTCAA

GGTCAAAGTCATACCCATTCATACGATCTGTTTTGAGATCGTAAAACTTGATGGCACCGA

CACCAACAGCGTGTGCAACAGCTTCTTTATCAGCAAGATTTGGATTTTTCGCTTCAATTT

GAGAAGCAGCGCGGTTAATGGCTTCAGCAACCGTAGGTTCAAGAAGAATAACATTACCTT

TACGTGTTGATAATTTAGCACCGCCTTTTGTGACAAGTCCAAAAGCAACGTGTGTCATAT

CATCTGACCAATCGTAACCCATTTCTTGTAGCACTGCTTTTAACTGTTTGAAGTGAGCAG

CTTGCTCATTACCAACAACATAAACGGATTTAGCAAAGTCATAAGTGCGTTTACGGTAAA

GTGCGGCAGCTAGGTCACGGGTAATGTAAAGAGTAGCGCCATCTGATTTTTTGATCAAGG

CAGGGTGTTCAATGCCGTATTTTTCAAGGTTAACAACTTGGGCACCTTTGGATTCTACAA

GAAGGTTTTTCGCTTCTAATAGCTCAAGAACCTCATCCATTTTGTCATTATAGAAGGCCT

CACCGTTGTAACTATCGAAGGTGACATGTAATTGGTCATAAAGACGGTTAAACTCAAGCA

AACTCTCATCACGGAACCATTGCCATAATTCGGTAGCTTCTTTGTCGCCATCTTCCAATT

TACGGAACCATTCACGAGCTTCTTCATCGACGGTTGGATCAGTTTCAGCTTCAGCATTGA

TACGAACGTAAAGTTTTAAGAGTTCATCAATAGGATGTGCCTGAACAGCAGCTTCGTCTC

CCCATTTTTTATAGGCAACAATGAGCATACCAAATTGTTTACCCCAGTCACCAAGGTGAT

TGATTTTTACAGGTTGATAACCCATTTTGGCAAAAATGTGAGCGAGACTATCACCAATAA

CTGTTGAGCGAAGGTGACCGATTGAAAATGGTTTAGCAATGTTTGGGCTTGACATATCAA

TAGCAACATTTCGCCCTTGACCTTCGTCTTGCTGAGCATAGTCTGAACCTGCTGTAATCA

CTTGTTCAAGGACCTGTGAGGAAATCTTAGCTTTATCAAGGAAAAAATTGATGTAAGGGC

CGACAGCTACGACTTTTTCAAATTGGCTCTCATCAATCTGTTCAGCTAACTCGCTAGCAA

TCATTTGAGGGGCTTTGCGAAGGACTTTTGCTAAGCTAAAAGCTGGAAAGGCAAGGTCTC

CCATATCAGAATTTTTTGGGGTTTCAAGCAAGTTAAAAATAGCATCTTGTTCTAATTCTG

GAACAACTTTAGCTATTTCACTTGCGATTAGAGTTTTAGTATCCATACAGCGTCTCCTAA

AAATTGTTTGTCACCCTATTCTAACATATTTCTTTAAAATAAACGAGGAGAGGATTGATA

AATTATACTGAAACTTTCAAGGAAATGAATTGCTTGAGGTTGTTATTTTTCTGAAAATGT

TATAAAAAGAGAAATGAGGTTTCTTTTCAACAGTTTTCAAGTGAAATTCTAGTTGGATTT

GATAAAAAATTGCTGAAAATAGCTGGGCGAAGATGAAATAATACACTTTTTTTGTTATAA

TTTGAGAGGATAATTATACAATTGGGGTGATATATCATGAATAAAATGGAGCGTCGACAG

CAGATTAAGCGAATCATTCAAGCAGAGCATATTGGCACACAAGAAGATATTAAAAATCAC

CTTCAAAAAGAAGGCATTGTGGTCACCCAAGCAACTCTATCTCGGGACTTAAGAGAAATT

GGCCTTTTGAAATTGAGAGATGAACAAGGCAAACTCTATTATAGTTTGTCTGAACCCGTT

GCAACACCATTTAGTCCTGAGGTGCGTTTTTATGTTTTGAAAGTTGATCGAGCAGGTTTT

ATGCTGGTTCTCCATACCAATTTAGGAGAGGCTGATGTTCTAGCTAATTTGATTGACAAT

GATGCCATTGAAGATATTCTAGGAACCATTGCTGGTGCGGATACCTTGCTTGTGATTTGC

AGAGACGAAGAGATTGCTAAACGCTTTGAAAAAGATTTAGCTGCAGGCCTATGACTAGCT

ATCAAGAGTCTTTGACACAATTGATTCTTGCTATCCATGAATTCTAGTATCGTTGCTTAT

AAAGAAGTGCAGCAATAGCTGTCTAAGCTCCGTGATTTTAAAAAGTAAGCTTATCAGATG

AAACGCTATCAACAAGATGCCCTGCTTTTCAAAAAAAATAGATAAAGAAAAGGCTGCGAC

AGTATCTGCAAGCAGGACAAAAGAACTAGAAGATAGGCTCAGTCATCAGCCATTAATTGA

TGATTATCGAGAAAAGATGCAAGATGCAAGTGATGTGATTCAGTATATCACCAAACGTAT

AGAAGATCAGTTAAACAAGGAGTTAACAAATGGCAAAAACTAACATTTCTCCTGGAATGC

AACAGTATCTGGACATCAAAAAAGATTATCCAGATGCTTTTTTGCTTTTTAGGATGGGTG

ACTTTTATGAATTATTTTACGAGGACGCTGTCAAAGCAGCACAACTCTTAGAAATTGGTT

TGACCAGTCGCAACAAGAATGCGGAAAATCCAATTCCCATGGCAGGCGTGCCACATCATT

CTGCCCAACAATACATTGATGTGTTAATTGAGTTGGGTTACAAGGTTGCTGTCGCAGAAC

AAATGGAAGACCCAAAGCAAGCTGTTGGGGTGGTGAAGCGTGAGGTCGTTCAAGTCATAA

CTCCTGGAACGGTTGTGGATTCAGCTAAGCCAGATAGCGCCAATAACTTTTTGGTAGCTA

TTGACTTTGATGGTTGCCGTTATGGATTGGCTTATATGGATGTGTCCACAGGTGAATTTT

GCGTGACAGATTTGGCGGACTTTACGAGTGTTCGTAGCGAAATCCAAAACCTCAAGGCAA

AAGAAGTCTTACTAGGTTTTGATTTATCTGAAGAAGAACAGACGATTTTGGTCAAGCAGA

TGAATTTGCTGCTTTCTTATGAAGAAACGGTCTATGAAGATAAATCTTTAATTGACGGTC

AATTGACAACGGTAGAACTGACAGCGGCAGGAAAACTCTTGCAATACGTTCACAAAACAC

AAATGCGAGAACTCAGCCACTTGCAAGCCTTGGTTCACTATGAAATCAAGGATTATTTGC

AGATGTCGTATGCCACTAAGTCAAGTCTAGATTTGGTAGAGAATGCTAGAACGAATAAAA

AACATGGGAGTCTGTATTGGCTGTTAGATGAAACCAAGACAGCTATGGGGATGAGGCTTT

TGCGCTCATGGATTGATCGACCTTTGGTTTCTAAAGAAGCTATTTTAGAGCGTCAAGAAA

TTATTCAAGTTTTTCTGAATGCTTTTATTGAGCGAACAGATTTAAGCAATAGCTTAAAAG

GTGTTTACGATATCGAACGCTTATCTAGCCGTGTGTCTTTTGGCAAGGCAAATCCGAAAG

ATTTACTTCAATTGGGGCATACCTTAGCCCAAGTGCCTTATATCAAAGCCATATTAGAGT

CTTTTAACAGCGCTTATGTTGACAAACTTGTCAATGATATTGACAGTTTGCCTGAGTTGG

AATACTTGATTAGAACAGCCATTGATCCAGATGCACCAGCAACTATTAGTGAAGGAAGTA

TTATCCGCAATGGTTTTGATGAGCGCTTGGACCATTATCGTAAAGTAATGCGAGAGGGGA

CAGGCTGGATTGCGGATATTGAGGCCAAAGAGCGTCAAGCAAGTGGCATTAATAACCTAA

AAATTGATTACAATAAAAAAGATGGTTATTATTTTCACGTTACGAATTCAAATCTTAGCT

TAGTTCCCGACCATTTTTTCAGAAAGGCAACTTTAAAAAATTCTGAGCGTTATGGAACAG

CAGAATTGGCTAAGATTGAAGGTCAGATGTTAGAGGCTAGGGAAGAGTCATCTAGTTTAG

AATACGATATTTTTATGTGTATTCGAGCTCAAGTTGAAACCTATATTAATCGTTTACAGA

AACTGGCTAAAATTTTGGCAACGGTAGATGTTTTGCAAAGTTTAGCAGTCGTTGCTGAAA

CCAATCATTATATCCGGCCGCAGTTCAATGATAATCATGTGATTACAATTCAAGAAGGTC

GTCACGCGGTTGTTGAAAAGGTTATGGGAGTGCAGGAATACATTCCCAATAGTATCTCTT

TTGACCAACAGACCAGTATTCAGCTGATTACAGGTCCAAATATGAGTGGTAAGTCGACTT

ATATGAGACAGCTGGCCTTAACGGTTATCATGGCCCAGATGGGTTCATTTGTGGCTGCTG

ACCATGTTGATTTACCTTTATTTGATGCGATTTTTACGCGTATTGGGGCTGCTGATGATT

TGATTTCTGGGCAATCAACCTTTATGGTGGAGATGATGGAAGCAAACCAAGCAATCAAAC

GCGCAAGTGACAACTCTCTTATTCTATTTGATGAACTGGGACGAGGTACGGCAACTTATG

ATGGTATGGCTTTAGCCCAGGCAATTATTGAATATATCCATGATAGAGTTGGTGCTAAGA

CCATATTTGCAACGCATTATCATGAATTGACAGACTTGTCAACTAAGTTGACAAGTCTAG

TCAATGTTCATGTAGCAACGCTTGAAAAAGATGGCGATGTTACCTTCCTTCATAAGATTG

CTGAGGGACCGGCGGATAAATCTTACGGTATTCATGTTGCAAAAATAGCAGGACTGCCAA

AATCCCTATTAAAGAGAGCAGACGAAGTTCTGACCCGTTTAGAAACACAGTCACGATCTA

CTGAGATAATGTCAGTCCCTCCACAAGTTGAGTCAAGCAGCGCTGTTAGACAGGGGCAAT

TATCCCTTTTTGGTGATGAAGAGAAAGCTCATGAGATTAGGCAAGCACTGGAAGCTATTG

ATGTCATGAATATGACCCCGCTTCAAGCAATGACAACCCTTTACGAATTGAAAAAGTTGT

TATAGTTTTTCAGCTGAAAATGAAAAAGATGCTTTCTATATTGAAGGCATCTTTTTGTTC

TGTCAAAAACGGTCCGAGGCCTTCGGCTATTTATTAAGTGTGTTATAATAGTCCATAAGA

ATGCGAGGAAATTATGACAAACATTATTGGACTTGGATTGGCAGTCTTTACAGGGTATTG

ACAGTCCTTAAGCATTGTTATGACTGTGTTTTTGAAAAAATATAGGTTTTAAGTTTCCTT

AGACTTCCCTCAAAAGTCCACAAAAAGGTGAACAAAAAAAGACCTTTACAGGTCCTTTAC

ACAATGAGTTCAGCAGGCAAGAACTAGCGTGGTTTAAATACTACGCTTTTTAGTTTGCCC

TATGGCTTATTATAGCACGATTAGTGTCATTGAGGAAAAAACTACCCTCTCAACCAGTCT

TTAAAGTTCCACCATTGGTCTTGGATGCTATCGCTCAAGCGTTGATGCCAAGTTTTTTTG

GATTCTTCTTCGATAGAACGCTTGAATAACACCTCCGCATATTGTTGCTCTTCGGCATCT

CTCTCTTTTTGAAGACGTTCATTATGTGTTTTTCCATCCTCAAATACAAGTTGCCCAAAG

TATAGATTCCCGTCATCCGCAAAAGATAAGGTAACTTTGTCTCCTTCAGCAAGTGGTTTT

TCCAATTGAACTGTGTAGTTGCCTCCTCCATCAGCACGATTGCCACTTACTGTCCATTTC

CTTCCTTCGCAGTTTGGATTATCTTGTTTCTCCAAAATAGAGCAAGGAATTTCATAAGTT

TTGTTTTCGGTTTTTTTAATTTTATTACCATTACTTGAAGTTACAAAACCATAAACATAC

CCATAGGGATCTAGATTAACATTAATGGTTGTATCTCCTGTTTTAAATGGTGCTACGGTT

ACTCCAGGTAAGTGCGATTGCCCTATAGTTGTAAACAAATCGCCACTTTCATTAGCTCTG

ACACTTATAGCACATATTGATATTAACAAAGTTAACATCATAACTAATTTCTTTTTCACT

ATATTCTCCTTAAAATTAAATATCGAACTCCACAATACCAGTTAATCAGTCAAAAAATCC

ATTCTACTTTTTGATATTTTATGTCTAATTTGACCCGCTAAGTCAGATTTGCAAACGATA

CCAAGTGATTTTCTTCTAATCATTATTTTTTAGATGGATATGTTAATTGTATTAGAGACG

TATTAATCTTAAACCATTGTCATAAGCGAACATTATTTTTTAGCGCCCCGTCTCTGTAAA

ATTCCACAAACTCAAGCAAGGCTCTTTTCTTAGATTCATAATACCAGCTTTGACTTCTGT

TAAGTTCTTCCATAATGTCTTGTTGAGTTTTTTCCTCACTGATCAAGTAACACTCAATTA

GTATTTGTCTATATTCTACTTTAGACAGTTGATTAATGGCATACCTAATAGCGTCTAGTT

CCTCTAGGGCGCATTCTCGGCTTATTTCAAGGTGTTTTCTGCGCGTGGGATGGTACTCTA

TATCAAACTGGTAAAGCTCGGTATAAGTTAAATCAAGGCTATTAGCGATACGTTGCCATC

TATGAAATTCTTTCAGCTTACGAATAGCGTTCTTCTTGCTCATCTAACACCTCTAAAGCT

TCTCTATGTAGTTTGAACACAATGTTTCTTGAGTAACCTAGTTTATCGAGTATCTCGTCC

CATGATAAGTCATCCACGTATCTAGCCTTAATAACAGCTATCTGTCTCTCATCTTGGAGC

GTGGCGATCATGGCTAGTCTCTTATCACGTTCTTTAGCTAAATATAAGAGTTGTTTAGCT

GTACCTTTTTCGATATGTCTTATTGCTTCAGGATTATGAAAAGCATTTATCAGCTCGATG

TCTTTATCTCGTTGTTCCTCAAATAAGGTTATTAAAGCGAAAAGTGGTTTCAATTCTTTA

AGTTGTTCTTTAGCGCTCATGGTCTTTTCTCCTGGTTATGGTATAATTTATTTAAGCTTA

AATTTAACCAAGGGGGCGTTCGTATGGACGTCTTTTTGTTTTGTTCGTTTTGTAAACTAG

ATATTTTTTGTAGTACTTCGCTTTTCGCTTCTAAAATCGTTTCTAAGCACTTTTTAGAGT

TTATAGTATAAATCATCAACTTAGTAGCTAGAAGCGCTAAAACAATGTTTTATTTTAGTC

TTAGATGGTTATTCTTTGTGGCAGGGTTATGGGCTAAAATGAGATGTCAAATCCTTTCCT

ATCAAGCTTTTTAATCTTCGTCAAAAATTTCAAAAAGGGAATTTTTTGCACGGAAAAGGG

CGCGTTCTTAAGTTTCCGAACAATATAGTCCCGTTTAAAAATGAAGGGGGTAGTTTCCGA

ATATTATAGCCAGTACCTGTCACCCTTATCCATATAGTAATGAAATCTTCTCCAGTGATA

AAGGTATTTGGTTAATCTCATGTACTTTGGACGTTTAGGAAAGTCATCACGACTATAATA

TCCATGAATGTGTCTTGCTTCTGGATTTACTTTCAAACACTCTTTAAAGGCTAATTGCCA

GTAGTAGCAACAGTCTGTCTTGCTTCGATTGAGCGTGGCTTGATGAACCTTCTGACAAGA

ACCACAAGCAAAGGCATGAGAAGCCTTAAATAATTTCCGACAACGTCTCTCACAGTCAGG

ACACACGAAAAAGTAACGTTTACCACCATAAGTTCCTGGAATCGTTTCAAGCAATAGGCC

TTGACCATTGTAGTGAATCACTAGCCCATCTAGGTCTATGCGGATAGCTTGGTCACCTAT

TGATCCTGTAACTCTTGTCTTCCCCTGTGTCTTCATTGGTTTAATGATATTCTCAATAGA

TAGTTCTAACATTATTTCTCCTTAAAACTCAAACAACCCAAAACTATTGATAAAACGACA

AAAAGAGGAAAAACCCTCTGAATGTCTATTTAACGAGTAACTGACCTTCAACAACCATAT

CATACAAATGGTTAAAGGCTTGACTGATAGACTCAAGGATGGCCCCTAAGTCTTCTGGCG

TCATCTCTTTATAATTCATAGAGAGGTGTTCAGCCAGTTGGTTGTGATCGGAGATGAAGG

CTATAAGTGTGTCTCGATTATTAACTTTCCCTTGAGTACTTTTAGATAAAGAAACCACGC

GCTGATGATCAAGTTCTTCATCCGTCATATCCTCAGGTTGGTTGTAATAGTCCTTGAAGC

TGTCACAGATACGCTTGAAGACTTTGTTTAGTTTTCTGTCTTCAACATATTTTAAGACTA

ACTGATTAGCATGACCACCTTGGTCATCATTGTGATAAGTTGCGTCAATCACTGGTTGCT

CATAAGTACCAGTCATATAGCCTAAAATAGCATGACAAGCCACTTGTGCGGTGTCAAAGT

CTTTAAACGTGTAGTGGAATGTGAATGTCTTTGGTGTGTCTGAAAATGTTCTCATGCTAT

TTCTCCTTTGTGATTGCTATAATGTCTGATAAATTGATAATGGCAGAAGGAGATGCTACC

CAGTTTGGTTGTTCTCCAGATAAAAGATACTTGACCAACTCATTATAAAGGGTGCGGTCT

CCTTGTATGGTGATGGTGTTACCACCTCGTGTGTGTAATTTTAGTTTCATATCAGTTACC

TGTACAAGACCAGTAGTCCTGTTCTTGAATCCATGTCTTCATAATTGCCATAAGTGGCTT

CTTGGAATTTAATGTCTATCACAGATACCGATAGGGTAAAGAGATTGACCCGATGTTCAA

AATCATCTAGTGATTCATTGTGTTTTTGATAAAATAGTTTGATTTTCATGTTTTAGCTCC

TTTCTCAACAGGTATCGCAAAGCCACAGTCAAAGACCCATTGGTAACCACGTCGTGTTAG

TTCTGGTCTGGTAAAATCACAATCACCCGGTCTAGACTTTATGTTGAAATTAATTTTGGT

GATAAAGTCATAACCGCATTGACCATTAGGAAGTTCCAATCGGTATAGCTGGTCGTTTGG

CAAATCATCATAATTGTAACGCATTTTCTTTACGCCATGATTTGTATAGTTAGGATTCAT

ATGTCCCTCTTTTCTCTCTAAAATGTGTGCTTTTTGTTTTTTTAAAAATCAAATAGCACA

GCAAAAAGCACAGGGATAAAGCCAGTGGTATCAAGGGGGTTGGGCACTTTGTGCTTTTAG

TAGCATTAATTTGAGCAAAAGACTTTCTTTTTTTGCACGCGCACTATTAGTTATAAATAT

TATTATCTATAATATTAAATACTACTAATACTACAATAGAGTATAAAGCCTATAATACCA

AGGTTTTAGCTTGTATCATTACGTGTAGTTTTTATGTAGCATTAGTGTAGTTTTTAAACT

CGTTCATAGTAAGGTACGGGAGTTCCACCTTTAAATAGTCTTTTTCTTGCGGGATTCTCT

CCTTTTTTCCAGCCGTCATCATTATCTAAGTAATCACGTATTTTTTGAGAAATAAGGGCC

TTACCACCTTGCGTTGGTTTTTGGTTAAATCCTAGATAAGCGATATGGTTAGGACTTGTC

ACTTGGAGTAAGCAATCTGTCTGTGCGGAAGGGTAGTCACTATAGCTTTGAGCATTATCT

AACGGCTCTCCTAGCTGTTTGAGGATGTATTGCCGTTGTTCATACTGTGATAAGCTATCC

CAACCTTCAACAATTTGAAACTCATTCAGTAATTGATCGATAATTTCTTTGTCGACGTCT

TCAACCTTATAATCTTCTTGAATGTCTGCTAACTGATTCATTAACTCTTTAGATGGCGTT

AGTGGTTCATAATTGTTAAACCATACTTTGGCTTCAGCGAGTACCTGTAAGAAATAATCT

TCTTCCACCTCCATAGGATGTTTTTTCACGTCATTGATACCACATTCAATAGGGAAAAAG

CGTCTTTCTGTTCCACTATCCTTAAGAAAAGATTTTTTATTAGCTGTTCCGATAAAGACA

CAGTGCCTTGGATGAGGAGTGGCTTTACGTTCATAAGGTTCACGATAAGTATCACTATCT

GAGGAAATGAAGCTTTTAACTGTTTCAATTTCTGCCTTTGACATGCCTTTTAGCTCCCCT

AGCTCAATAATGGCATTGGCTTGTATCTTCTGATAATCACTATCATTTTTACCAAACTTG

ATTTCTGAATCAGTGTGGTAGCTAGGGAGTAGTCGCTTAGTAACGGTGCTTTTCCCAGTT

CCTTGTCTTTTATCAATGAGAATAGGAACGACTTCAAACTTTACTTTACGGAGATAAATT

CTAGCCATGAGACCTGTTAGCCATACTTTGGCAATTTCTCTATTATAGGAATTATCAGCA

CAGCCTAATAGATCAATAAAGTAGCGTTCTCCTCTAGCTTTACCATCCCATTTTTGACTT

TCAATACGCTGTTTAATGGGGTGATAAGTGTTCTTTTTAGCTAAAGCGGTAATAGCTACC

TCTATATGTTCTTTACGAGGGGTAAACCGATATTTTTCATCAATGAATGCAATACAAAGG

CTGGTCTGCTCGTTCGTCCATAGCCCTTTTTCTTTAGACCAAGGAACTGCTTTAGTGATT

TCAATAGTTTTTTCAAATTCGTTGTATTTAATACCTGTATAGATATTTTCGTAAAATTCA

AAAACCTTACCGACATTGTAGGGGCTACTAATGACATATTCTTTGTCTCCTCTACCTTTT

CGTGTCCTGAAAGCAGGGGCAAAAGCAGGTTGAGTGGCTTGCGATAGTTTATTTTGATAG

TCTTTCAATTCTTCTTTGTCTATGGCTTGATTCCTCTCTTTCTTAATTCTTTATCAAGTA

TGCTTCTAAAGGTTGTATCTATCTCATCAATGGGTAGTGGCTTAGTTGTCACGCTGTTAG

CTATTTGTACCAGCTCATAAGCCGTCTCTAAATCACAATCCACCCATTTATTAAATAGCA

AGCCAACAAACTTAGTTAAGGCCACGTTGCGCCCGCCTTCGTCTCCAAAACCATTAAACA

AGGTATCTATGACCCTCATGGTAATAGAACGCTGACTTCTAGGGCGTGGCGTGTAAGTAG

TAACAACTTGTCTGTTTGGCGTGCTACCATTTTTAGGAACAGGATAATCAAGACCATGGT

TCACATAGCGCTGATAGTCCTCTGGGTCGCCTGTTGTAACGGGTAAGCCTTGTAATTGCG

ACCAGGTAAGACTAGCTAAATCAAACGGCAGTCCAATCTTATCGGCTATCTCCTTGACCA

CTTGTTTATAAGTTGCTTCAGTCATCACGTCACTAGGCTTCATGACAAGGCGATAACGGG

GCTTCTCGGGGGTGTGTTTAATCGTTGGATAAATAATATAACTATACTCCCAAAGCGTCT

GAGAAACGATTTTAGGTAGGTTGACGCCTGTTTCTATCTCGTCATAGTCAAGAAAAATCA

AATCGCGATAAACTAAACTAGCATTATTGCGCTTATAGCTACCGTTTTTCTCTGCTGTGA

CCTTGCCACTTAGGCAGTAGGGGGCTTGTGTTCGCTTGTATTCTTCAATATCAATATCCT

CAGGCGGTTTCAAAGGTCTAAACTGAGCAACATAGTCAAATGGTTCTAAAGGTCCTTTGT

AGGGGTACAAATAAGAGCTAAAGCCTCTTGCTTCATAAATAGCCATCTACACATTTACCC

CCAAAAAGATAAGAATATCACTGACCTTGTAATAATGTTTCCTGGTGTCTTCTAGTGGTG

GTTGGTATCGTCTTAACCCAGCATTTTCCCACCGTTTTAGGGTTTTACCTTTGATATTTA

ATTCCTCTTTGACTTGTTCGGCCGTGATCAACCCTAAAACTCTTGGTTTAGGTTTCTGGT

AGGCTTCCAAAAAGTGATTAAAAGCGGTCAGGTTTTGTTCTAAGAGTTTGGCTTCATAAT

CTTGACTAAATACGTTCATGCCTAACCTCCTTTGAGTAATTCCTTATAACTGGTTAAATC

GGCATTCAATAACACACTTAGGCGTTCCTGTTCCTTTTGTACTTGATTATAAAAGGCTTT

AGCACCATCTAGTAATTCTTCTTTGTTAGCTGGGATAAAGTACCCACGATTGAATCCGTG

TCTAATGCCGATAATAGGGACGTTATAGCGCGTGATTAAGCTACTGATGATACTTTGGAC

GGAGCGTTCTTCAAGTTTCAGTATTAAGCTAATCTCTGCCCCTGTAATGGGGTTGTCTGC

TCCAACCTTGATCAGATTAAGGACACGTCTATAATTCTCTGGTAGTGTCATTCAGTTCCT

CCCTAATTGTAATAATGGTTCTGTGCTTGAATATAAGCCCCATAGTTTGCGTTCTGACGT

GGTTTAGGTGCGTGGGTATCTTCTGGTAAGTCAATCTCTATTAACGGCTTAGAACGGCTA

AGAAGAAGCCCTAAGAGACCTAAAACAAAGAATAGAATAAGCGTCTGTGTTGGTGTGAGG

TTAAGTTCTTGCATCATGCCGATACCTCACTTAAATAAGTTTCTAGTTCCCCTGAGTCTT

TCTCTGAACAAGGTAAACCGTTAACGGCTCTAAAGACAATCTCTGTGGTTCGTTGATAGT

CTAAAGCGTCCCATGCTTCTTCAAAGCTGGTGGCACTTTTTCTGAATTTAATGACGTACT

CTGTCATAACGTTAGCAATAATTACCCAAGCAATATGTTGGTTATATAGTCGAGTGAAAT

AGACTTCAGCTTTATCTTTGCTGAGTTGGCGATTTTTGAACATTTCTAGCTGTTCAGGAG

TGTATCTATCTTTTGAAAAAGGATTTGTTTCTACTCTATATCTCATTATGTTTTTTCTCG

CTTAATTATTATTTTCTGTGTAGTGTTTTTATTGATTGCTTGTTTCTTATACTAGATTCA

TGCTAGTTTTAAGGGGTAGCTCCCTGATTAGTTCATGTTAGTGTATAATTCTGCGAATAA

CTCGCTAGGGATACGCTCTAGCGCTTTTTGTTGTAAGTGGATGGCTTTAATTCTATCTTG

TGTTTTGGTTTTAATGTCTTCTATAATTTCAGATGTTGAGACCACTTGTTCATAGTAAAT

GTTAGCTTTATAAATGAGTCCTTGTTCTTTTAATTCCTTATTAGCCATTTTTTCAAGCGT

AACTTCATGTAATACTTCAACATTACGATAATGACCGTTTTTGAGGTCGAATTTTAGCCA

TTTTTTACGCTTCCATTTATATAGGGTGCTTCTGCAAACTTCTGAATTCCCAAAACCAAG

AAAAGAAGCGATTTCTGTTAATGTTTTTCCTTCAATTTCAGATAGTTTATAAGCGACGTT

TCTAAATAACACTCTCGGATTTCTTTTTTTCTTAATCATATTGTCTTGATTTTTGAGCAC

ACAAAAAGCGCACTCCCTTTCTATGAATTTTAGGTTCACAAAATAGAGTACGCATGATAT

ACTATTTACGTACCTACTTTGTGGGTGCTGGGAGTTCCTAACGTGTACGGTCGCCAAACT

ATACCACGTTAGGAACTTTTTTATTTTTCAGACTCATAAGAGTTTACAGCTTTAACGATT

AAATCCGCTTTAGATAATCCATTTTTATCCGCTGTTTCTTGTATTGTATTATACTCGTCA

GCGGTCAAACGAACTTCCAGCCGTTTATCACGTTTAGCTGTACCTTTTACAGGTCTGCCC

ATTTTTGGACTCATAGGAGTTCCTCCTTTCATTTAAGCCCGTGCTTATATCATATGATAA

GCACGTACATAAGTCAACCCCTAAATTAAAAAAACATGCGTATTCTATTTTCAATGTGCA

AATGGTTCACAAAATAGAGTACGCATGATATACTATTTACGTACCTACTTTGTGGGTGCT

GGCTCTATCACGTTGACGGTTTGGCGATTGTAAACGTGGTAGAGTACTTTTTTATTTTGT

AAGATTTTCAAAGGCTTTTCTCAAAGTCTCGGGTTTAGTCAAGTTATTCTCTTTAGCGTA

CTTATTTAGTTTGCTATCTAACTCTTCACTAACCCTTACCGTTAATTTAACAGTATTTGA

GTTTTCTCCCTTTGGTCGTCCTACTCGTTTTTTGGTGTCGGTCATTGAGTTTATCACTCC

TTTCATGTCGACAAAAACTATTATAGTTTATGTCGTCAATAATTGTCAACCCCTAAACCA

AACTTTTTTTGCGTACTCTATTTTATTTTCAATGATCAGTATGATACAATGGAAGTATCA

AATATTTACTAAAACCCCTTTAATAATAGCTTGCCTGCTTTATTAATTGAGTTTAGTTAT

ACTAGTTGAAGGCTTGGAAGTTTGGTCGCTGTCAAAGCCTTTTTTGTTGTTTTCACGCGC

ATTGTAGCGTGTTTTTTAATGCCATTGTCTTAATATCTTGATAGGTAAAATTAAGATTGA

TAAGAGCGATTGCCATATCTTCTAAAGCAGTGTACTGTGCTAGTTCAATTGAGTTTAAGC

AGTCAATATCAGAATAACCACCTCTGGTTACTTTTAATTGCTTGCTATTCTTACCAGTAA

CAGCTTTAAGCAATAGATTGTACACAGTCGGATAAGCCATTTTAGGCGCGTGTTCCCATG

TCTTGATAGCTTCATTAAGTGTTTTTCGTTTAGGAGCTTCAAGAGAACGCTGTAACCTAA

TTTGAGTGAGTTCTTCTCGCATTTCAAAGAATGCCTTGACTAGGTTCTTTTTAAACTCTC

TGACTGGTTCGGTATTTCCTAGATAGGTAACTAATAAAGTCGCCTGCTGTTCGTTTAAGT

GATAAATTCTTCTAGGTCGTCCGCCTAGTGAACCTTTTTCAGGTTTATGGATTTCAAATG

ACAAAACCCCAAACGCTTCTAAAAGGTAACGCATGGTCACGCAAAGTTACGCAGAATTTT

CCATGTTGCGTAACCTACTCAATCTCTTTAGTACCAATCGATTTCAGCATTTATTTAAGA

AAGGTTACGCAGTAACAAACAATAAGTCTAATTAATGATTTATTAATTATTTATTTATAT

ATAGGGGTAGGGTAGGGTGATTTTGCGTTACTGCGTAACCATCCTTCTCTAACGCTATCT

ATATCAACGTTTTAGAGGTTACGTAGTAAAAAAGGGGGTGCGTTACCTTATGTACCACAC

AACAGCACTTTCGTTCTTAATCCTTTTTATGCTTATCAATCAGCTTTCTAACTGAAATAA

TGTCAATTTCAGCATGTTCAGCTATGATGTCGTGTGTGGTGTAAGGCTCTTTCTTACCAT

CCATGTAAACTAGGTTCATGGTAATCCTCCTTTATCCATAGAGTTCCGTTAGTTCTTTAA

AATACTGATCAGGAATTTCATCCATAGCCACTTGTTGTAATTGAATGGCCTTTAAACGAT

TGGTGTCGCTAGCAGTCGGTTTATTAATAATTTCAGCCGTTGCCTGTACTTGCTTGAAAT

ACTCTTCAAGCTTAAGTTGCCTTCCTGCGGAAACTTCTTCGGATATGGCTTTTCTGTTAT

TGATAATTTCAAAGGTATCAATTTCACCGTTTGCCATGGTGTAATCAATGTTATTTCGAT

ACCGCCAAGCTGCCAGCCTAAGCTTGATGTCTTTTTCAGACCAATCGGGAAGCCGCTCAG

CAATCAGCTCTAGGCTCATTGTTCCTGTATCGTCAAATATCTGATGTAATAATTCTTGTG

TAAATGGTGTTCTAGCCATTTGTTATTTACCTGCCTTTCTTAATCGGTGATAGCTAAGAA

GTCGCTAACAGTTTGATTAGCGAGCTGTGCTATTCTTAATAGACTGTTTCTATTCGGGAG

GTTTCTTCCTTTCTCCCAATTATTAACAGTACCTTTAGAAGTGCCAAAGCGTTTTCCGAA

TTCCTCCATAGTTTCTCCTAAGTTAAGGCGAATAGTTTTGATTTTGTCACCCACTGTCAT

TTCTTGCCTGCCTTTCTAATTAATAAAGTACTATTATTTTCGTACTTTTTTACCTAAAAA

AAGGTTATCAATAGTGACACCGTACAAAGATGATAATTTTTGCAATAGTCCTAAAGAAAT

ATCGGAGCTATCTTTTTCGTACTTTGAAATAGTTTGGGGATTTTTCCCAACCGCTCCAGC

TACTTGTTTTAAAGTGTAACCAGCGTTGATTCGGGCAGCTTTTAGTGTAATTTGCGTCAT

GTTTTCAACTCCTTTCTAAAAAGCTATGGCTTAATAGTACTATTATTTTCGTACCAAGTC

AATGATTTTTGTAGAAAAATATTAAAAAAATAGTACTTTTAGGTTTATTTGTGTTAGAAT

TAATTCAGATACAAAAAGAAAGGGGTAAGAGAAATGGCTAAAAATAGTCCCCAAGATTTA

ATAAATAGAGAAATTTTCTCAACAAATCTCAACATGCTTATGGCTAAAAAGAATATCAAA

CAGATAGATATTCACAACAAACTAGGAATACCTAAGAGTACGATAACTGGCTATGTTAAA

GGTCGTTCACTCCCAACTGCTGGAAACGTTCAAAAGCTGGCGGACTTCTTCGGAGTTCTA

AAATCAGACATTGACCCCCGTTTTGATTCTAATAATATTGAAACAAATAGTAATATTATC

CCATCAACCCTACAAAAAGTAACATCTACTTTATCTCAGCTAGAACACAAGCGACAATTA

AACGTCCTTGATTATGCTGAAACACAATTAGAACAACAAAACACAGTAGAAGAACCACAA

GCCACCTACTACACTTACAACTACTACGACCACGCAGCTTCAGCTGGTACAGGTCAGTAT

CTAAATGATGTACAAGTAGAAACAATTGAATTACCAGTCGATTACGACGCTGATTTTGTC

ATACCGGTTTATGGCGATTCTATGGAACCCGAATACCATTCTGGGGACTATGTATTTATC

AAACTATCTATTAACCTGTCAGATGGTGATATAGGAGTTTTTGAGTATTACGGTGATGCT

TATATCAAACAACTTGTTATAAACGATTCTGGAGCGTTTCTGCATAGTCTGAACGACAAG

TATGACGATATACTCATAGATAGAGATAGTGATTTCCGTATTATCGGAGAAGTTATTGGG

AGTTTTACATCTAAATCATGACTATCTGATACCCACGCGCCGAATTCCACTATTTTCCAC

TAATTTTGGTTGCTTTACCTTTAAGGACTCGCATTTTGCTCTTTAAGACAGCCGAAAAGT

CCGATTTTCTGAATACTATACGCTAAAAATGCCAACTGATTTTAGAAGCTGTCACAACGG

AAAAAGTAAATTAATAAACGACCGATATATCAAGTTCTTTAAGTGAATTTACCGAGCGTT

TTACAACTATTGAAATAGGTTGACGTATTATGTCAGTACGTGCCAACATTTTCCAGCATT

CCGAAATGCGACCATGTGTTCGTGTTTGGGAGTAGTAACCTTCTATTCTCTAAGTTCGCT

CGAAGTTCAGCATTATGCGCGTGGAATAAAATTAGCTACCTTACTGTAACCTTACCGTTA

CCGCTCATTTTATGACCTGTTCAATTTTCATGTCTAAATGCCAAATTTATCATCTTTTAC

CTACGCGCAAATACCTTGATACGCCTTTAATTTTCTTTAATTACAAGACCCTCAAAACTT

GGCAAAAATTGAGGTATAATCTGAACTTTTCTGAACTTTTTGTCGGCGGTAATTAAAAAA

ACTATCTGCGCGTGATCAGTGGTTGCATAAAGTATTATAAATTCTTGCATAAAATTATTT

TTATTCATTTTGGTAATACTAAGGAATGTTAAGAGCGCTAAAGACTGTACTTTCTAACTA

CACCCTTTTGACTTCTTTATCAAACAAAGCTATAATGGACATAGAAAAAGGAGATTGCGC

AAACAATCTCCTGTGGTAGCACCGTTTAAGACGGCAGCCTTACCGTATTTGTTTATATTT

TCTATAAACCGTCCACGATTGGCTAAAGTGTGGGACGGTTTTTCTATTTGTTCTTGTTAT

TCATGATAGCTACTATCAGAGTACCAAAGGCAATCATCAAAGTAAGCGTTTCATAAACTG

ACAAACCTTGTCGTCTCCTTTCTTTTGGTTTCTGTGACTTACATACATAAGCACCACCTC

CAGACATAAGGCTACGACTACCTTGACCTAGTTTTTAGACATGCAAAACTTCTTCTGAAA

CTCTCTGTACCTTACAAAACCAAACAAAAAAGACCACGCAAGTTTTCCACGCTCGCAAGG

TCTTAAAAAGACTAATATTATACCATGATTTTCTTTTATAATATTTCGGATATTTACCCG

ATACCATTATTATACCATGATATGAACTAATCTAAAACCCTTTTAATAATAGCTTGCCTG

CTGATGGAAAGGTTTATGATCATGAAAATAACAGAACATAAGAAGAAAAACGGTACAATT

GTTTATCGTGCTAGTATTTATCTAGGCATTGACCAAATGACAGGTAAGAGAGTAAAAACA

AGCATCACAGGAAGAACAAGAAAAGAAGTTAATCAAAAAGCCAAGCACGCGCAGTTTGAC

TTCCTATCTAATGGATCTACAATTAAAAGAAAAGTTGTGATTAAAACATTTAAAGAACTT

AGTCATTTATGGCTTGAAACCTATAAGTTAACAGTAAAGCCTCAAACTTATGATGCTACT

GTTACTAGACTTAATCGACATATTATGCCAACTCTGGGCAATATGAAGGTTGATAAGATA

ACCGCTAGTGATATTCAAATGCTGATTAATAGATTATCTAAATATTACGTCAATTATACT

GCGGTACGTTCAGTCATCCGAAAAGTTCTCCAACAAGGAGTATTGCTAGGGCTAATAGAT

TATAACTCAGCAAGAGATATTATCCTTCCAAGGAAGCAGCCAAACGCTAAGAAAAAAGTT

AAGTTTATTGATCCGTCTGATTTGAAATCTTTTTTAGAACATTTAGAAACTAGTCAACAC

AAACGCTATAACCTTTACTTTGATGCAGTTCTCTACCAACTTTTATTATCCACTGGCTTG

AGGATAGGCGAAGCCTGTGCATTAGAGTGGGGAGATATTGACCTAGAAAATGGTACAATA

GCCATTAATAAGACTTACAATAAAAATTTGAAGTTTTTGAGTACAGCTAAAACCCAGTCA

GGCAATAGAGTGATTAGTGTTGATAAAAAGACCCTTAGAAGCCTAAAGCTCTATCAAATG

AGACAGCGACAATTATTTAATGAGGTTGGTGCGCGTGTGTCGGAGGTAGTGTTTGCCACA

CCAACACGAAAGTATTTTAATGCTTCGGTTAGACAAAGCGCTTTAGATACTAGGTGTAAG

GAAGCAGGGATTGAACGCTTTACCTTTCACGCTTTTAGACACACTCACGCTAGTTTATTG

CTGAACGCAGGTATTAGTTATAAGGAACTTCAGTACCGTCTAGGACATGCGAATATCAGC

ATGACTTTGGATACCTATGGCCATCTTTCTAAGGACAAAGAAAAAGAAGCTGTTTTATAT

TATGAAAAGGCTATGAATAATTTATAAGTCCACAAAAAAGTCCACAAATTAATATTTTGA

GAGGTGTAAACCTAATGAAACCTTGTTATATCAACGTTTAGAAAGTGTTAAAAACAAAAT

TATGACAAACATTATTGAATTACCGGAAGTTCTCGCCAACCAAATTGCAGCTGGTGAAGT

TGTAGAAAGGCCAGCGAGTGTTGTCAAGGAATTGGTTGAGAATGCTATTGATGCTAAAAG

TAGCCAGATTACCGTTGAAATTGAAGAGTCTGGCCTTAAGATGATACAGGTTACAGACAA

CGGTGAGGGAATGTCTCATGAAGATTTACCTTTAAGTCTGCGTCGCCACGCTACTAGTAA

AATTAAGAGTCAGAGTGATTTGTTTAGAATTAGAACACTTGGCTTCCGTGGAGAGGCTTT

ACCGTCTGTTGCCTCTATCAGTAAAATCACGATAAAAACAGCAACAAAAGAAGTCACTCA

CGGTTCTCTTCTTATAGCTACTGGTGGGGAAATTGAGACACTTGAAGCGATCTCAACTCC

TACTGGAACCAAAATTAAGGTTGAAAACCTTTTTTACAACACGCCTGCTCGTCTCAAATA

CATGAAAAGTTTACAGGCAGAATTAGCTCACATTGTAGATGTGGTCAACCGGTTGAGTTT

GGCACATCCAGAAGTTGCTTTCACACTGATTAGTGATGGTCGCCAATTAACTCAGACATC

AGGAACTGGCGATTTACGCCAAGCAATTGCAGGGATTTATGGTTTAAATACTACCAAAAA

AATGCTGGCTATCTCTAATGCTGATTTGGATTTTGAAGTTTCCGGCTATGTTAGCTTGCC

AGAGTTGACACGCGCCAACCGTAATTACATGACGATTTTGGTTAATGGGCGTTACATCAA

GAATTTCTTGCTTAATCGAGCAATTCTTGATGGTTACGGTTCTAAGCTCATGGTTGGACG

TTTCCCAATTGTTGTGATTGATATTCAGATTGATCCCTATTTGGCCGATGTCAATGTTCA

TCCCACAAAACAAGAGGTTCGTATTTCAAAAGAGCGTGAGTTGATGGCTTTAATTAGTAC

GGCGATTTCTGAAAGTCTTAAGGAACAAGATTTGATTCCAGATGCTCTGGAAAACTTAGC

CAAATCAAGCACACGACATTTCTCTAAACCAGAGCAAACACAACTTCCTTTACAGTCTAG

GGGACTTTATTATGATCCTCAAAAGAATGACTTTTTTGTCAAAGAGTCGGCTGTCTCGGA

AAAAATACCTGAAACTGATTTTTATTCCGGCGCTGTTGACAACAGTGTAAAGGTTGAAAA

AGCAGAGCTGTTACCCCACTCAGAAGAAGTTATAGGACCTTCTTCGGTTAAACACGCAAG

TCGTCCCCAGAATATCTTTACCGAGACTGATCATCCTAATCTTGACTTAAAAAACAGACA

AAAGTTATCTCAAATGCTAACCCGTTTGGAAAATGAAGAAAAATCAGTATTTCCTGAATT

GGATTATTTTGGTCAGATGCACGGAACTTATCTCTTTGCTCAGGGAAAAGATGGTTTATT

TATAATTGACCAACACGCTGCCCAGGAGCGGGTTAAATACGAATATTATCGTGATAAGAT

AGGTGAGGTTGACAGTAGTTTACAGCAATTGTTGGTGCCCTATTTGTTTGAGTTTTCCGG

TTCTGATTTCATTAACTTACAGGAGAAAATGGCACTCTTAAATGAAGTTGGTATCTTCTT

AGAAGTTTATGGGCACAATACTTTCATTTTGAGGGAGCATCCTATCTGGATGAAAGAAGA

AGAGATTGCATCTGGTGTCTATGAAATGTGTGACATGTTACTTCTAACCAATGAGGTATC

TATTAAAACTTACCGAGCAGAGTTAGCTATTATGATGAGTTGTAAACGATCTATCAAGGC

AAATCATAGCTTGGATGATTATTCAGCAAGAAATCTGCTACTGCAATTGGCCCAATGTCA

AAATCCTTATAACTGCCCTCATGGTAGACCCGTATTGATTAATTTTAGTAAGGCAGATAT

GGAAAAAATGTTCCGTCGAATTCAAGAAAATCACACTAGCCTGCGAGAGCTAGGAAAATA

TTAGAGAGGATGAGATGCAAGAGTTTTTAAACCTTCCTAAGCAGATTCAGCTGAGGCAAC

TGGTACGCTTTGTGACCATTACCTTAGGCAGTAGTATCTTTCCCTTTATGGCCATGTATT

ATACGACTTACTTTGGTACGTTTTGGACAGGCCTCTTAATGATGATTACCAGTTTGATGG

GATTTGTTGGAACTTTATACGGTGGGCATTTGTCAGATGCTCTTGGTCGCAAAAAAGTCA

TTATGATTGGGTCAGTAGGAACAACGCTAGGCTGGTTTTCGACTATTTTAGCTAATTTGC

CTAATGCGGCTATTCCTTGGTTAACCTTTGCGGGTATTTTATTGGTAGAGATTGCTTCTA

GTTTTTATGGTCCTGCCTATGAAGCTATGTTGATTGATTTGACTGATGAGAGTAATCGTC

GATTTGTTTACACCATCAATTATTGGTTTATCAATATTGCCGTCATGTTTGGTGCAGGGC

TATCTGGGCTTTTTTATGATCATCATTTTTTAGCCTTGTTAGTAGCTTTATTACTCGTCA

ATGTACTTTGTTTTGGCGTTGCTTACTACTATTTTGATGAGACTAGACCAGAGACACATG

CTTTTGATCATGGAAAAGGCTTGCTGGATAGTTTTCGAAATTATCGTCAGGTGTTTCATG

ATCGTGCCTTTGTCTTGTTTACCTTAGGTGCCATCTTTTCTGGTAGTATCTGGATGCAGA

TGGATAACTATGTGCCAGTCCATTTGAAACTGTATTTTCAGCCAACGGCTGTGTTAGGTT

TCCAAGTAACTAGTTCTAAAATGTTATCATTAATGGTTTTAACTAATACATTGCTGATTG

TCCTTTTCATGACAGTAGTAAATAAATTAACGGAAAAATGGAAACTATTACCTCAGCTTG

TGGTTGGTTCTTTACTATTTACTCTAGGGATGCTCTTGGCATTTACCTTTACGCAGTTCT

ATGCTATCTGGTTATCAGTTGTTTTGTTAACTTTTGGGGAAATGATAAATGTTCCTGCTA

GTCAAGTCCTACGTGCTGATATGATGGATCATTCCCAAATAGGATCTTATACAGGTTTTG

TGTCAATGGCACAACCCCTAGGTGCTATTTTGGCTAGTCTCCTAGTATCTGTCAGCCATT

TTACAGGTCCTTTAGGCGTGCAATGCTTATTTGTAGTCATTGCTTTGCTAGGGATTTATT

TTACGGTTGTTTCTGCAAAAATGAAAAAGGTGTAATATGTACGATTATATTAAAGGTCAA

TTGACCAAAATTACGGCAAAATACATTGTCGTTGAAGCTAATGGATTGGGCTACATGATT

AATGTTGCCAATCCTTATAGCTTTACAGATAGTGTCAACCAATTGGTAACCATTTATCTG

CATCAAGTGATTCGTGAGGATGCTCACCTGTTGTTTGGTTTTCACACGGAAGATGAAAAA

GATGTTTTTCTGAAATTAATTTCTGTATCAGGTATTGGTCCGACAACAGCTCTTGCTATT

GTGGCAGTTGATGATAATGAGGGACTTGTGAATGCCATTGATAACAGTGACATTAAGTAC

CTAATGAAATTTCCTAAAATTGGTAAAAAAACAGCGCAGCAAATGGTTCTTGACTTAGCT

GGCAAATTTGTGGAGGCTCCACAAGAGACTGGTCATACCAAAGCGCGCAGCAATAAAGCA

GGCAATACTCAACTGGACGAAGCGATTGAAGCCCTCCTTGCCCTCGGTTATAAAGCAAAA

GAGCTGAAAAAAATTCGTGCCTTCTTTGAGGGGACCTCTGAGACGGCAGAGCAATACATC

AAATCAGCATTGAAACTGTTAATGAAAGGGTAGGTGTTTCACATGAAACGTTGCTCATGG

GTTCCTAAGGATAACCAGCTTTACTGTGATTACCACGATTTAGAATGGGGTCAACCCCTA

CATGATGATAGAGATTTTTTTGAATTACTCTGCCTTGAAAGTTACCAGTCAGGCTTATCC

TGGCTGACTGTTTTGAAAAAACGCCAAGCTTTCAGAACTGTTTTTCATCATTACGACATT

GCATCGGTTGCAACCTTTACCTCGGAGGAACTGGCTGACGCTTTAGAGAATCCATCCATC

ATTCGCCACAAGCTAAAGCTAGCAGCAACTGTCAACAATGCTATCGCAGTACAGAAAATT

CAAGAAGAGTTTGGTAGTTTTTCAACTTATCTCTGGAATTTTGTTGGGGGAAAGCCAATT

AACAACCTTGTCAATCAAGAGAATCTTGTTCCTGCTCAAACAGAATTATCTATTCGCTTA

GCTAAGGATTTAAAGAAAAGAGGATTTAAATTTTTAGGGCCTACAACGGTTTATTCTTTT

ATGCAAGCATCGGGTCTGGTAAATGACCATGAAGAGGCTTGTGTTTTCAATAGAATCTTC

TAATTCTTCGTGAATTGGAAGATTTTTTGATAAAATGAGATCAGATTACGAATAGATAAG

TAGGTGGGGAAATGAAAGCTGAACTGATTGCAGTAGGTACCGAAATTTTGACTGGTCAAA

TTGTGAATACCAATGCTCAATTTCTGTCGGAAAAAATGGCAGAGCTAGGTATTGATGTCT

ATTTTCAAACGGCTGTTGGGGACAACGAGGAGCGTTTACTTTCAGTGATTACAACTGCTA

GTCAGCGGAGTGATTTGGTCATTTTATGCGGTGGCCTTGGTCCAACGAAAGATGATTTAA

CCAAACAAACTTTAGCAAAGTACCTTAGGAGAGACTTGGTTTATGATGAGCAAGCTTGTC

AGAAACTAGATGACTTTTTTGCTAAGCGCAAGCCTTCATCACGGACACCAAATAATGAGC

GACAGGCACAAGTGATTGAAGGATCAATCCCTTTGCCAAATAAAACTGGTCTTGCGGTTG

GTGGGTTCATTACAGTCGATGGTATTAGTTATGTTGTCTTACCGGGTCCTCCAAGTGAAT

TGAAGCCGATAGTAAATGAAGAATTGGTACCACTTCTGTCAAAACAATACAGTACATTGT

ATTCAAAGGTACTACGCTTTTTTGGTATTGGGGAAAGTCAGTTGGTAACAGTCTTGTCAG

ATTTTATTGAGAATCAAACTGATCCAACCATTGCTCCGTATGCTAAGACTGGCGAAGTGA

CTCTTCGCTTATCAACAAAAACTGAAAACCAAGCTCTGGCAGATAAAAAGTTAGGTCAGC

TAGAAGCGCAGCTACTATCCCGAAAAACTCTTGAAGGTCAACCCTTAGCTGATGTCTTTT

ATGGCTATGGGGAGGATAATTCCTTAGCGCGTGAGACATTTGAGCTCTTAGTAAAATATG

ATAAGTCAATTACAGCAGCAGAAAGTCTAACCGCGGGATTATTTCAGTCAACTTTGGCGA

GTTTTCCAGGAGCTTCTCAAGTATTCAATGGAGGCTTTGTGACTTATAGCATGGAAGAAA

AAGCGAAAATGCTAGGCCTTCCTTTAGAGGAGTTGAAATCGCATGGCGTTGTTAGTGCTT

ATACGGCCGAGGGGATGGCGGAGCAAGCAAGGTTATTGACTGGTGCTGATATTGGGGTAA

GTTTAACAGGTGTTGCCGGACCAGATATGTTGGAGGAACAGCCTGCAGGTACAGTTTTCA

TTGGTCTTGCCACTCAAAATAAGGTAGAATCAATAAAGGTTTTGATTAGCGGGCGAAGTC

GTTTGGATGTGTGCTATATCGCTACTTTACATGCCTTTAATATGGTCCGTAAAACTTTAT

TAAAACTTGAGAATTTGCTATAATGTTATTTAGTAGCTATAGTAGGATATGGTTTACAAT

TATGTAGATTAATCCTGCTGACGATGGTTTGTTAAAAAAGAGGAGAAAGTATTGGCAAAA

AAATTAAAAAAGAATGAAGAAATCACTAAGAAGTTTGGTGACGAACGTCGCAAAGCTCTT

GATGATGCTTTGAAAAATATTGAAAAAGATTTCGGTAAGGGCGCAGTGATGCGATTAGGA

GAACGTGCAGAGCAAAAAGTTCAGGTTATGAGTTCAGGAAGTCTAGCTCTTGATATTGCG

CTTGGAGCTGGTGGTTATCCTAAAGGACGTATCATCGAAATCTATGGTCCAGAGTCTTCC

GGTAAAACGACTGTGGCTTTACATGCTGTAGCACAAGCTCAAAAAGAAGGTGGAATCGCA

GCCTTTATCGATGCCGAGCATGCGCTTGATCCAGCTTATGCTGCTGCGCTTGGGGTTAAT

ATTGATGAACTCCTCTTGTCTCAACCAGATTCTGGAGAACAAGGACTTGAAATTGCAGGT

AAATTGATTGATTCTGGTGCGGTTGATCTGGTTGTTGTCGATTCAGTAGCAGCTTTAGTG

CCACGTGCTGAAATTGATGGTGATATTGGCGATAGCCATGTCGGATTGCAAGCACGTATG

ATGAGTCAGGCCATGCGTAAATTATCAGCTTCTATTAATAAAACAAAAACTATCGCTATT

TTTATTAACCAATTGCGTGAAAAAGTTGGTGTGATGTTTGGAAATCCTGAAACAACACCA

GGTGGTCGAGCTTTGAAATTCTATGCTTCTGTTCGGCTGGATGTGCGTGGAACAACTCAA

ATTAAAGGAACTGGTGACCAAAAGGATAGCAGCATTGGTAAGGAGACCAAAATCAAGGTT

GTTAAAAACAAGGTCGCTCCGCCATTTAAGGTAGCAGAAGTTGAAATCATGTATGGGGAA

GGTATTTCTCGTACAGGGGAGCTTGTGAAAATTGCTTCTGATTTGGACATTATCCAAAAA

GCAGGTGCTTGGTTCTCTTATAATGGTGAGAAGATTGGCCAAGGTTCTGAAAATGCTAAG

CGTTATTTGGCCGATCATCCACAATTGTTTGATGAAATCGACCGTAAAGTACGTGTTAAA

TTTGGTTTGCTTGAAGAAAGCGAAGAAGAATCTGCTATGGCAGTAGCATCAGAAGAAACC

GATGATCTTGCTTTAGATTTAGATAATGGTATTGAAATTGAAGATTAAAAAGAGCCCTTC

AGGGCTTTTTAAAATGTATTTTTAAAGGTTAGTAGGACTACTGTTCATAGGTGTGTGGGT

GAAAAGAAACTATCATTTAAAAAGATGTGAGAGAGCAGAACTTAGGTTAACTGATATAAA

ATTAAAATAACTAATAATTCAAGGGAATACAGGTGAAAAACCTTATTTTACCAAGATAAT

ATCTTTAAAATTAAAAAAATAGGTCCAAAGACCTATATTTATAGATAAGAAACTGTGCTA

CAATAAAGCTATCACGAAATAGAAAGTGAGTCAGCAACATGATTAAAATTTACACGATTT

CAAGTTGTACGAGCTGCAAGAAAGCCAAAACTTGGCTAAATGCCCATAAGCTTGCTTATA

AAGAACAGAACTTAGGAAAAGAACCGCTAACTAAAGAAGAAATTTTAGCAATTTTATCAA

AAACCGAAAATGGAGTGGAGAGCATTGTTTCATCTAAAAATCGTTATGCCAAAGCTCTCG

ATTGCGATATTGAAGAATTAAGTGTTAGTGAGGTTATTGATTTGATTCAAGATAACCCGC

GCATCCTTAAAAGTCCCATTTTGATTGATGATAAACGTCTGCAAGTAGGCTACAAAGAAG

ACGATATTCGTGCTTTTTTACCTCGCTCTATTCGTAATATTGAAAATACTGAAGCACGAT

TACGTGCTGCACTCTAAAAGCTCTTGTAGAGCTTTTTTTTAAATACCTTCTTCGCTTGAA

ATTCTTGTTTATTTAATAGAAATAAGCTATAATAAAGCATAGCACTTAATTTTAGAAAGA

AGGTGTAAGTATGGGATTTACAGATGAAACAGTCCGCTTTAAGTTGGATGATGGTGACAA

ACGACAAATTAGTGAAACATTAACAGCAGTCTATCATTCACTTGATGAAAAAGGTTATAA

TCCAATTAATCAAATTGTTGGTTATGTCTTGAGTGGGGACCCAGCTTATGTTCCTCGTTA

TAATGATGCTCGAAATCAGATTCGTAAATATGAACGTGATGAAATTGTAGAAGAACTTGT

TCGCTACTATTTACAAGGAAATGGAATTGATGTTAAATGAGAATAATGGGACTTGATGTT

GGCTCGAAAACGGTTGGTGTAGCTATTAGTGATCCTCTTGGCTTTACAGCCCAAGGACTT

GAAATTATCAAGATTGATGAGGAAAAAGCAGAATTTGGCTTTACACGTTTAGAAGAGCTT

GTTAAGCAGTACCAAGTTGAACAGTTTGTTATTGGATTGCCAAAAAATATGAATAACACC

AATGGTCCACGTGTTGATGCAAGTATAACTTATGGAAACCACATAGAGCACCTTTTTGGA

CTTCCAGTCCATTATCAAGATGAACGATTGACGACTGTTGAGGCAGAGCGTATGCTTATT

GAGCAAGCTGACATTAGTCGTGGTAAGCGAAAAAAAGTCATTGATAAATTAGCTGCACAA

CTCATTTTACAGAACTATTTAAATCGGAATTTTTAAGGAGATAAAAAATGACACATAATC

ATGAAAATGACCACCAACACGAAGTTATCACACTTGTGGATGAACAAGGAAATGAAACCT

TGTTTGAAATTTTATTAACAATTGATGGTCGTGAAGAGTTTGGTAAAAATTATGTTCTCT

TGGTTCCAGCGGGATCTGAAGAAGATGAGTCTGGTGAAATCGAAATCCAAGCCTATTCAT

TTACTGAAAATGAAGATGGTACTGAAGGGGATTTACAACCGATTCCTGAGGATTCAGATG

CTGAATGGGATATGATTGAAGAGGTATTTAATAGTTTTCTAGATGAGAACTAAATTGTTA

GTGACACTATGTTAAAAAGTAGCCGTGAGGGCTATTTTTTTTAATGTTATTTTCGAATAA

ATAAGCAGGAGGTGATAATGATAAAAGAATTAAATGATAAATTTATTAATAATTATAAGG

CTAAACCTTTGCATAGATTTAGAGAATTATTACAGTATGTGAAAATATATCAGAAATTGA

CTAAATAAAAGATTCTGTTATACTTGTTTTATAATATTCTTTGACGATAATATCATTTTT

CAACTTACTTTGTTTATGCACTGTCACTTTGCTATGTGCAATACAAAGTCATGTTATTTA

TCTCATACTAAAAAATAATGGATAATAGCTATGTTAGACTAGTGATATGAAGAAATATTG

CTTTTATTGAATCGTTAGAGAATATGATATTTCAAGGAGAAAAATAGTGATCGAGTTAAA

CGATGAATTCATCCGAAAAGAGACGATAGAACTGGCCAACGATGGCCCACGAGTACACAC

CACTCAATATGAAACTAAAGTACCTCGGCTTCATAAATGCTATCTGCTTTTCTTTAGCAT

TATTATCAGTTCTTTAACAATTGCAGTACCTTTTTTAACAGATGCAGCCAATGGTCTTCA

GTCCCAAAACTTATATATAGGAATGATGTTAACGAAGGGGCAACTTCCCTATAGTGATGC

TTTTACAACAGGAGGGCTCTTTTACTTTGTTATCATTGCATTAAGTTATTATTTGGGATC

GACACTTTGGCTAGTCTTTGTTCAGGTGTTTTGTTTTTACTTATCTGGTTTATATCTTTA

TAAACTCATTAATTATATGACAGGCTTTCAAAAAGTGGCTTTAACTTTTTCAATTAGCTA

CTATTTGTTATCTGTTAGTCTTGGTTTTGGGGGATTGTATCCTACTCAACTGGCTATGCC

ATTTATATTAATATCGGCTTGGTTTTTAACTAAGTATTTTGCCTGTTTAGTGAAAGATGA

GGCATTTATTCTTTTTGGCTTTGTAGGTGCTCTTGCAATGCTAATTGACCCGAGTACCCT

TATCTTTTGGTCTTTTGCTTGTGTGACAGTTTTTTCTTATAATATAAGCCAAAAGCATCT

TGCAAGAGGTTTTTATCAACTGCTAGCTTCGATTTTTGGAATGATTTTAGTTTTTTACAC

AGCAGGATATTTCATTTTGAACTTACAAGTGCTAAATCCTTATTTATCACAAACGATGAT

TTATCCTTTTACTTTTTTTAAATCAGGAAACTTATCGTTGCTTTTTGGACTGGCTATTCA

GTTGTTCTTCGCTTTGGGGCTTGGTCTTTTGACGGGAATGGAGAATGTCATTAGGCGATT

TAAAAACAATTCTGATAGGGTCGTCAAGTGGCTATTTGTCATGGTCATTCTAGAATCTAT

ACTTGTGGCTATATTTTCACAAGACTATCGCCCCTATCATCTTTTACCTCTTTTACCTTT

TGGATTAATTTTGACTGCTATTCCTGTTGGCTATCAGTATGGTATAGGATTAGGTCAGAG

TAGTCATCGCAGACGTCATGGTAAAAATGGTGTTGGTCGAGTAATGATGATTTATCTTAA

GAGACACTTTTATTTGCCAATTTTAATTGTAGGGACAATACTAATCTGTTCTACTTATTG

TTTCATTAGTAGTATTCCTCTTAATCAGGAGCGTGATCATATTGCTAGTTATTTAGAACA

GAAACTAAATAAAACTCAATCTATTTATGTTTGGGATGATACTTCTAAAATTTATTTGGA

CAGTAAAGCTAAATCTGTTTCTCAATTTAGTTCTCCTGACATCAATACGCAAAAAGAGAG

TCATCGAAAAATATTAGAAGATGAACTATTAGAAAATAAGGCTGCTTATATCGTTGTTAA

TCGCTATAAAAACCTGCCTAAAATCATTCAAAAAGTATTATCTACTAATTACAAAGTAGA

TAAACAGATAACGACAAAAAGTTTTATTGTTTATCAGAAAAAGTAATCTAATATCAATAT

ATTGTGTTCGAGCAAAAAACTTGACACAATATATTGATTTTTTTAGTTGTCGTGATATAA

TAGTTCCGTAAGGAGATTTGATATGGTAAGTTTAGAAGAAGACAAGGTGACTGTTCAACC

TGATATTAAAGTGATTAAACGAGATGGTCGCCTTGTTAATTTTGATAGTACAAAAATCTA

TAGTGCTTTATTAAAAGCAAGCATGAAAGTAACTCGGATGTCGCCACTTGTTGAGGCTAA

ATTAGAGGCTATTTCTGATCGCATTATAGCAGAAATTATTGAGCGTTTTCCAACTAATAT

CAAAATTTATGAAATCCAAAATATTGTAGAGCATAAGCTTCTTGCAGCTAATGAATATGC

TATTGCAAAAGAATACATTAATTATCGTACTCAGCGTGACTTTGCACGTTCACAAGCAAC

AGATATCAATTTTTCTATTGATAAATTAATTAATAAAGATCAAACAGTTGTTAATGAAAA

TGCTAACAAAGATAGCGATGTTTTTAATACTCAACGAGATTTAACTGCTGGAATCGTAGG

GAAATCGATTGGTTTAAAAATGTTACCTTCGCATGTTGCTAATGCTCATCAAAAAGGAGA

TATCCATTACCATGATTTGGATTACAGTCCTTATACACCGATGACGAACTGCTGTTTAAT

TGACTTTAAGGGCATGTTAGCCAATGGCTTTAAAATTGGTAATGCTGAAGTGGAAAGTCC

CAAGTCTATTCAAACTGCAACAGCTCAGATCTCACAGATTATTGCGAATGTAGCATCAAG

TCAGTACGGCGGATGCACAGCTGATCGCATTGACGAGTTTTTAGCCCCATATGCGGAGCT

TAACTTTAAAAAACATATGGCTGATGCTAAGAAATGGATCGTTGAGACTAAGAGAGAAAG

CTATGCTTTTGAAAAGACTCAAAAAGATATTTATGATGCGATGCAGTCTTTGGAGTATGA

AATTAATACGCTCTTTACGTCTAATGGTCAAACACCATTTACTTCTTTAGGATTTGGTTT

GGGGACGTCTTGGTTTGAACGTGAGATTCAAAAAGCTATTTTGACCATTCGGATTAATGG

TCTTGGTAGTGAACATCGCACGGCTATTTTCCCTAAATTAATTTTCACGGTTAAACGTGG

CTTGAATTTAGAACCAGATTCACCAAACTATGATATTAAGACTTTGGCTTTAGAATGTGC

GACTAAGCGGATGTACCCGGATATGTTATCTTATGATAAAATTATTGATTTGACAGGATC

TTTCAAATCTCCAATGGGATGCCGCTCTTTCCTTCAAGGCTGGAAAGATGAAAATGGGCA

AGATGTGACCTCAGGCCGTATGAATCTTGGGGTTGTCACCCTCAATTTACCTCGCATTGC

CATGGAATCAAATGGCGATATGGATAAGTTTTGGGAGCTGTTTAATGAGAGGATGCTAAT

TAGTAAGGATGCTTTAATTTATCGTGTCGAACGTGTCACAGAAGCAAAACCAGCAAATGC

CCCTATTCTTTATCAATATGGTGCTTTTGGAAAGCGTTTGGAGAAGACAGGGAATGTAAA

TGATCTCTTTAAGAATCGTCGTGCAACAGTCTCTCTTGGCTATATTGGTCTTTATGAAGT

GGCGTCTGTTTTTTATGGTGGTCAATGGGAAGGTAATCCAGATGCTAAAGCTTTTACCTT

GTCAATTGTCAAGGCAATGAAACAGGCCTGTGAGGATTGGTCAGATGGATATGGTTATCA

TTTCTCTGTTTATTCGACTCCATCAGAAAGTTTGACAGATCGCTTTTGTCGTTTAGATAC

GGAAAAATTTGGCATTGTGACAGATATTACGGATAAAGAATACTATACAAATTCTTTTCA

CTATGATGTGCGTAAGAGTCCGACACCTTTTGAAAAATTAGATTTTGAAAAAGATTATCC

AGAAGCAGGTGCTTCAGGTGGTTTTATCCACTACTGTGAGTATCCTGTTTTGCAACAAAA

TCCAAAGGCCTTGGAAGCGGTTTGGGACTATGCTTATGATCGTGTGGGGTATTTGGGAAC

CAATACGCCTATTGATAAATGCTATAATTGCCAATTTGAAGGCGATTTTACCCCAACAGA

ACGTGGTTTTACTTGCCCAAACTGTGGCAATAATGACCCTAAAACAGTTGATGTGGTCAA

ACGTACATGTGGTTACTTGGGGAATCCTCAGGCCCGCCCAATGGTTAACGGTCGCCATAA

GGAAATCTCTGCGCGTGTAAAACATATGAATGGTTCTACTATAAAATACCCAGGCCTGTA

AACTCAAGCTGGGGGAGAGCCCTTTTGATGGAAATATGATATGCTTAAAAGGTTGCTTCA

TGCAAGCAATCTTTTATTGTTTAATGGTTTCTTAGCAATTGTGAGATAAGAATAACTAAT

CCAAAACGACTAATTAGCTGTTAGAGCAATCGGACTATAAGAAGAAAGAGATAAGGAAGA

AATAATGGGAAAATATCAATTAGACTATAAAGGAATGCAGCAGGTAGAACGCTTTCATGA

GAAGCATTCTAAAAAAAAGACGGATAAAAAATCCCGCCTTCAAGAACTTAAGGCTCGGTT

TTTAGAGAAGTCAAAAAAACAGGATAACTAAAGTATCCAGTAAAGAAAGAAGGTTGAGTA

TGCTTAATATTGGGATTGTGGGACTAGGTGCTATTTCACAAAAAGCCTATTTACCATATA

TGAGACAGCTGAGTGACATTACTTGGCATCTATCAACGCGGAATGCAGCAGTACGTCAGC

AGGTCGGTCAGTTATTTGGTCACGCTATTCTTTACAGTGATGTCAAGGAGTTGTCAAAAA

CAAACTTAGATGGGATCTTTATTCATGCGGCCACATCGGCCCACGCAGAGTTGGCTAGTT

TATTTTTAAATCAGGGGATCCCAGTCTTTATGGATAAACCTATAGCTGACAATTACCTGA

TGACAAAGAACCTCTATGACTTAGCCAAAGAAAATCAGACTTTTCTGATGGCAGGATTTA

ACAGGCGTTTTACACCTCGTGTCAAGAAGTTGTCAAGTTTGTCAACTAAGCGTAAAGTGG

CTGTTGAAAAAAATGACTTGAACCGGCCAGGAGATATGGCTTTTAAGCTCTTTGATTTCT

TTATCCATCCTTTAGATACAGCCTTATTCTTGACAGAAGGGACCTTACTAAAAGGGCATT

TTCAATATCATCTGGAAGCAGGCTTACTGAGTCAAGTTATGGTAACCCTGATGACCGAAA

GCATGACGACTACAGCTTCTATGAATTTACAATCAGGGAGTCGCCGTGAGGTAATGGAAG

TTCAACGGGCTGAAGAAACTTATCATTTAGAGAACTTAGACGAGTTATCTATTTATAAAG

GTACTGAAAAAAGGGTGCTTGGCTTTGCTTCTTGGGACACTACCTTGCACAAAAGAGGTT

TTGAAACAATGATCGATGCCTTTTTAGAAGCTATTAGCACAGGCGTCAATCCTGTCAGCC

CTGAGTCTAGTCTTTTAAGCCACTGGATTTGTCAACAGATTGCTGACTCTCAGCTTTCTT

ATGGAGAGTTAACGGTAGAGTTGCCTAAGGATTAGGAATTGATATGGAAATAAGACGACC

GACTTTAAAAGATAAAGATGCTGTATTGTCAATGATTAACGAATTTTTAGAGCAGAAAAG

TGCTACAGATGGGTTATGGCATTTTAACGTCAATGACTTCAACTATGAAACATGGTTGGA

AGATTCCTTGCGACAGGAAATGGGTTTGTCCAGTCAAGGAGTGCCTGCTATTCAATACGT

CGCATTTGATGAGAGAAGTCAGGCTATCGGGTTCTTAAATCTTCGTTTACGGTTAAATGA

GAGATTACTTGAAAAAGGTGGGCATATTGGTTACTCTGTCCGTCCCAGCCAGCGTGGGAA

AGGTTATGCCAAGGAGATGCTTAAACAAGCTGTCAGCTGTGCGATTTCCAAGAATATCAC

GACAATTTTGGTAACTTGTGATGAGACTAATGTTGCAAGCCGAGCAGTTATTGTGGCTAA

CGGCGGTGTCTTGGAAGATAGTCGAGGTGGAACGGAACGTTATTGGATTGAAGAATAAAG

AGGCGATAAGTGATGGCAGAAAAGTGTTGGAATAATCCTAAGCCAAAGCAATGGCAGGCA

GAGGAGTTAAGTCAAGGCCGGATTATTGACTATAAGGCTTTTAACTTTGTTGATGGTGAA

GGGGTTCGTAATTCCCTTTATGTGTCTGGTTGTTTATTCCATTGCAAGGGGTGTTATAAT

GCTGCGACTTGGTCTTTTAAGGCGGGAATGCCTTATACGCAAGAACTTGAAGAGCAGATA

ATGACGGATTTGGCACAGCCTTATGTTCAGGGACTGACACTTTTAGGTGGAGAACCCTTT

TTGAATACGGGTATCTTAATTCCTCTGATTAAGCGTATTCGGTGTGAATTGCCAGAAAAA

GATATTTGGTCTTGGACGGGTTATACTTGGGAAGAAATGATGCTTGAAACACCAGACAAA

CTGGAAATGCTGTCCTTAATTGATATTCTGGTAGATGGTCGTTTTGATATCACAAAAAAG

AACCTCATGTTGCAATTTAGAGGCTCTTCTAACCAGCGAATTATCGATGTTCAAAAGTCT

TTGGCTGCTAAAGAAGTGATTATCTGGGATAAGTTAAACGATGGGGACCAAACCTTTGAA

CAAATTAGTCGGGAGGATTTGCTTTAAGAAGCTGACTTATCCACATGATTTTTTTGAAAA

GGTCATGTGAAAACAGGTCCCTTAAGGGTGAATTGGGGAGAAAGTAATTGTTATTCCCTG

GTTTTCCCCACATTATCCTCATAGTTATCCACAGGTTGTGTATAAGTTGTAGAATTGTGA

ATAAAGACTAAAAAAGAGGCCTAGGCCTCTTTTTTGATATAGACAAATTCATGATCTGTT

GATAGGTCTGGATGGTAGTAAAAACCAGCAAAGACAAGGCTTTTAAGGCTGTCAACAGTT

TGGCAATTATTTTCCATACAGGCAGTAAGAAATGCACCTCGTGCCTTTTTTGAAATGGTG

GAATGGGTTTTGAATTGGCCCTCTTTTTCTGCCATAAACTTGGGACCTATCCACAATTGT

TTACAGTCTTTGGAAAAGACATCGTCAAATTCACTGGATAAAAGGGAAATCACTTGGGGA

TGCTCTTTGGCAAACTGATTATAACAGGGCCGCCAGTAAGACTTGAGGCTTTGTCCTTCA

ATTTTTATTCTGGTGTGAAAATCATGGCGGTGTTCTGCGATAGGATGATTAGCGGGAATG

ATACCGTAAAAGGATGAGGTGATGTAAACCTGCTGGGTGAGGTAAGCCTGTTCTTGGGTA

GTGAGCTTATCGCGCTTGATATGGCGGTACATAAGGCCATTGAACAGTTGGTAGGCAGGG

TAAGCCAAGCTTTGTTGAGAGGTCATGTCTTGCCAGCGTTGCTGTTCTTTTTTAGCTGCC

TCTTCTTTGATACGATAGGCTTTGGCCAAGTCCTCTGTGGTCATAGCTGCCATTGCCTTT

AAAATGGCCTGGCTTGGTTGGGGCAGTAGGTGTGGATAAGATTCTTTAGGAATCACCATT

TCTTTTGCGGTTGGAATTAAAAAGGTTAGCATAGTGTTATTGTAGTAAAGTTACTTGCTT

TCTGTCAAGAAAACTAGTCTTGTCCAAGGATGACGTTCAAACGGCTACCTTCCTCGTCTT

CTGTCGTGAAAGAAGAAGAATCTTCTTGTAAAATAGCTAGGCCATATCATCTGGCTTTTT

TGAGAGTGGCTGAAAAAAGCAGGGGCGTCTCAATGTGAATGGTTAAGAAGGCTAGACCTG

GAGTGCCTTCTTGGTGTTTTTTGAGGTAAGGGGCTGACCAGTGATTGAATGCAAGGTGAT

GATGGTAGTTCCCTGAAGCAATCCAGCTAGCACTTGGAATGGTCATTTTATCGCCTAAAT

CAAAAACCTTTTGATAGAGAAGTGAAGAATCCAGAGCATTTTTGACACTCAGATGGACAT

GACTAATGCGTGTGTCTTGAGCTAGTAAAAAGTGCTTGGGGATATCTGTCAACTGCTCCA

ATATGCTTTTGGTATCCGTGGGCTCAGTCACACCAATAATTTGACCATTGTCGCGGATAT

CCCAATGCTCAACAGCTTTGTCATGATAGATTTCAATCCCATTACCCTCAGGATCGCTGA

GGTAGATAGCTTCGCTATAGCCGTGATCAGCTGCTCCTTCTAAAGAGATACCTCTGGTTA

AGAAATGAAGTCCCAAGCTATGACGATCAGGAACTAAAAAGGCTGTGTGATAAAGACCAT

AAGCCTTATTTGCTGGTAAAGGTGTTTGTCGTAACTCTAAGATAACGGTTTTACCGTCTG

TAGTCAATTGACGAGATGTCGTGTCTTGTGAGAGAACCTGTAAACCGATAATACTTGTGT

AAAAGGTTGTCATCTTTGCTAGATCGGTAACATTGAGGGAAACCTTTCCTAGAGAGATGG

TTGAGTTGTAAGGGTATGTCATCCTATTGTCTCCAAAAGTGATTAAGCCTATGATAACTT

ATTAGTTTCAAAAAGAAAAGTAGGCACAAATAAGTGGTATAGGCACTAAAAAGTAACTCA

TTTGTCAGCCCTATCTTGTTTCTGTTCTCTCTAATAGTCTATAATGGGTTATAAGTTACA

AAAAGATACTAAGAGGAGCTGCTGCCTATGCTGAAAAAAAGACCGGCTTGTCCCGTAGAA

ACAACCTTATCGGTAATCGGGAATAAATGGGAATTATTAATATTAAGGGACTTGCTAAAG

GGGACCTTGCGTTTTGGGCAGTTGAAGTCCTCAATAGGTTCGGTCAGTCAAAAGGTACTT

ACTGCCCAATTAAGGGCTATGGAAGCAGACGGATTGGTTCATCGGGAATTGTATGCAGAA

GTGCCACCTCGGGTGGAGTATTCCTTAACAGAAACTGGGTTGAGCTTAGCTCCTGTTATT

GAAGCGATGTCGGATTGGGACCAAACTTACCAAGAAAAACATGCCGATTTATTTGAAGAA

GGCTAGAGGAAAGAGTTGTCCGAGCATACTGAGACTTAGAGAAGCTGAGAGTTTTTTTAA

AATGATATTATCAAATTCTTTACAGTTTGATAAAATTACTTTTTTTCTACAAAAAGACTT

GCGCTCATCTCACGAATATGATAATATAATATCAAGTCTGTTTGAGAAAACAGAAGCTCA

GGTCAAGTAAGTGCACACTTACAATAACACAGTTGATAATGGGGAAACTCAGAGTCAAGA

AAAAGAAAAGCAAGCCTTGCATTTTGCAAGGCTTTTTTCTGTCTTTATTTTGGTCTGCGC

CGAGCAAAATCTAAAAATTTAAATTTTTCCAGTTTATGGCGACTCTCGGTGAATTGGAAT

TGGTTATTATTGGAAAGGTAAACTTTGGATTTGACGGAAACAACATGTTGGTCTGATCCA

AGGTCAAGTAAAATTTTATCACGATCGGTTATTTGGTCAATGGTAACCTCTTTGAGGGCA

AAGTCAATGGCGAGATGGAGTTCCTTTTCCAAATATTGATAAATAGAATGCTCTGCGATT

TCCCTTGTCATTATTGGCACCAAAGTTTTACTAAGGTAATCAATGTCTAAGACGGAAGCC

ACACCCTCAACAACGCGTTGGCGCGTAACACGCCAGACCAAGCTGTTTTTGCTAAAACCG

GTCAATTTTGAAAGTGTCTCATCCACAATCAGTTTATCAATGGCAATGACGTTGGTCTTA

GAATCTAGATTTGAGTAAGAGACGAGTTCCTGATAACTAGTTAGTTCTGAGATAGGGAAC

ATGATCTGATGATGTTTAATCACTTGGGTGCCACGTCCTTGTTTTTTGAGGATAAGACCT

GCCTTTGTGAGAAGTGAGAGAGCCTTGCGGACGGTATCTCGGCTAGCTTGGTATTGTTGA

CTCAATTCTATTTCAGTAGGTAAAAAATCCCCTTCTTTGTAAAAGTCTTTGTTTATTTTT

GTTTCGAGGTCTTTATAAATACGTTCATACTTTGTCATAGGGTAAATTACCTTTCCACTT

GTCTATTTTTTTACAGACAACACCTTTTATTTTACCAAAATAATGGCTTTTTTGGCACAA

ACAACTTGCAGACAAATTTGCAATCGATTACAATAAAGGTGTCAATCAGGTTCTATCTAA

GAATTTGAATATTTCATTTTGACGTTGCTTGTTAAAAGCAACTAGAACAAAGGACTAGGG

GATGTCACATGGGAAAATTTGAACAGGATGCTAAAAGTCTTCTAACTGCTATTGGCGGTA

AAGAAAACATCAAGGCTGTCACACACTGTGCAACGCGTATGCGTTTTGTTTTGAATGATA

ATAATAAGGCAAATGTCAAAGAGATTGAAAAAATCTCTGTAGTTAAAGGGACATTTACCA

ATGCTGGGCAGTTTCAGGTAATCATTGGTAATGATGTTCCAGTTTTTTATAATGACTTTA

CAGCTGTTTCTGGTATTGAAGGGGTGTCTAAAGAAGCTGCCAAATCAGCAGCTAAAAGTA

ATCAAAATGCCTTACAACGGGTGATGACCATGTTGGCTGAGATTTTCACACCTATTATTC

CGGCGATTATTGTTGGGGGGCTTATTTTAGGTTTCCGTAATATTTTGGAGAGTGTGCCTT

TTGAATTTCTTGGGCAGCAGGTCGAAAAAGGGAAATTAGTTTTTGATGCAGCTGGGGATC

CTGTTTGGAATACGATTGTGAGGGTATCTCCTTTCTGGTCAGGGGTTAACCATTTCTTGT

GGTTACCAGGGGAAGCTATTTTCCACTTCTTACCAGTTGGGATTACTTGGTCTGTGACGC

GTAAGATGGGAACCACTCAAATTTTAGGGATTGTCCTTGGTATCTGTTTGGTGTCACCAC

AATTATTGAATGCCTATGCGGTAGCAGGAACGCCTGCTGCTGAGATTGCCAAAAACTGGG

TTTGGGATTTTGGTTTCTTTACCATTAATCGTATTGGGTATCAGGCACAGGTTATTCCAG

CCCTTTTAGCTGGTCTGTCCCTTGCTTATCTTGAAATTTTCTGGCGTAAACGGATTCCAG

AAGTGGTTTCAATGATTTTTGTGCCATTCCTTTCTTTGATTCCAGCTTTGATTTTAGCGC

ATACGGTATTGGGGCCAATCGGTTGGATTATTGGTAAAGGGATTTCCTTTGTTGTGTTAG

CTGGATTGACTGGTCCTGTTAAATGGCTATTCGGTGCTATCTTTGGTGCCTTGTATGCTC

CGCTAGTTATTACTGGTTTACATCACATGACAAATGCCATTGATACCCAATTAATTGCTG

ATACTGCAACTCGTACAACTGGTTTGTGGCCAATGATTGCTCTTTCAAATATCGCTCAAG

GGTCAGCCGTTTTTGCTTACTATTTAATGAATCGTCATGAAGAACGTGAGGCTGAAATAT

CGCTTCCTGCAGCAATTTCTGCTTACCTTGGGGTAACTGAGCCTGCCTTATTTGGGGTTA

ATGTTAAATACGTTTATCCCTTTGTAGCCGGAATGATTGGCTCAGGTATTGCGGGTCTCT

TATCAACAACCTTTAATGTTCAGGCAAATTCTATTGGTGTTGGTGGCTTACCAGGTTTCA

TGGCTATCAATGTGAAGTACATGATTCCATTCTTCATCTGTATGGCAGTAGCCATTGTGG

TACCGATGTTTTTAACCTTCTTTTTCCGTAAATCACATATCATGACTAAGACAGAAGATG

AGGCTAAACTACCTGAGACACCCGTTTCGGAGGCTCCTGCAGCAACTGCTCCACATAAGA

CTATGCAAGGGACAGTTATCACTTTAACAAGCCCTTTAACGGGTGAAGTTAAAGCGTTGT

CTGAAGCTGTTGATCCTGTCTTTGCACAGGGAGTTATGGGTCAAGGTGCTCTTCTTCAAC

CGACAGAAGGGGTGTTAGTAGCGCCTTGTGATGCTGAAGTATCGGTCTTGTTCCCAACTA

AACACGCTATTTGTTTGGTGACGACTGAAGGTTTGGAATTATTGATGCATATTGGCATGG

ATACGGTTAACTTAGCTGGTCAAGGATTTGAAGCTTTGGTGAAGCAAGGTGATCAGGTTA

AGGCTGGACAAACATTTATTCAATTTGATATAGCAGCTATTTCTGAAGCTGGATACGCCA

CTGAAACGCCTCTTGTGGTGACTAATCAAGATGCTTTTACGGTAACTGTTGAAGGTAACT

TACCGCATCAGATTAAGGCTAATGATAAGTTAGTAGTAGCGATGAAAAAGTAGGTTTACT

AGGGAGAAAACGTTCAGGAACTGAGATCAGTTCCTGAAACTATTTTAGGGAGGAAAACTC

ATGACAATTGATAAAAAGAAAGTCGTCTATCAAATTTACCCAAAATCCTATAAGGACACT

ACTGGAAATGGTGTGGGAGACTTGCGAGGGATCATTGATAAATTGCCTTACTTACAAGAA

CTAGGAATAGATATGATTTGGTTGAACCCTTTCTACCCTAGTCCACAACGAGACAATGGC

TATGATGTTTCAGATTATACGACGGTCAACCCTGATTTTGGGACGATGGCTGATTTTGAA

GATTTGGTAAAAGCTGCTAAGGAGCATCAGATTGAGTTAATGTTGGATATGGTTTTGAAT

CACTGTTCCACAGACCACGAGTGGTTCCAAAAAGCTTTAGCAGGAGACCCTTATTATCAG

GATTTCTTTATCTTGAGAGATCAGCCGACTGATTGGGTTTCCAAATTTGGTGGGAATGCT

TGGGCGCCTTTTGGAGATACAGGCAAGTACTACTTACACTTGTTTGATGTGACACAGGCT

GACTTGAATTGGCGGAACCCACATGTTCGTGAGGAATTGGCTAAAGTGGTTAATTTTTGG

CGAGATAAAGGAGTGAAGGGGTTCCGGTTTGATGTGATTAATCTGATTGGGAAAGATGAA

GAGCTGGTGGATTGTCCGGTCAATGATGGTAAGCCAGCCTATACGGATCGTCCTATTACT

CACACTTATCTTCATGATCTCAATCAAGCCAGTTTTGGTCAGGATGATTCGTTTATGACA

GTAGGGGAAATGTCTGCCACGACTATTGACAACTGTCTTTTATACACGGCTCCCGAACGT

GAGGAGCTATCCATGGCTTTTAATTTCCACCATCTAAAAGTTGATTATGAGAACGGTCAG

AAATGGACTATTATGGCTTTTGATTTTGCAGCGCTGCGAGACTTATTCCATGCTTGGGGT

GAAGGCATGAGTCAAGGCAATGGGTGGAATGCCTTGTTCTACAATAACCATGATCAACCA

CGTGCCCTGAATCGTTTTGTTGATGTAACACATTTCCGAAACGAAGGTGCGACGATGTTA

GCGGCTTCCATCCATTTGTCACGAGGAACGCCTTACATTTATATGGGTGAGGAGATTGGC

ATGCTTGATCCAGACTTTGATAGTATGGATGATTATGTGGATGTGGAAAGTCTCAATGCT

TACTCAAGCTTATTAGTCTCAGGTAAAAGTGCGAAAGAAGCCTTTGCCATTATCAAGGCT

AAGTCAAGGGACAATGCCAGAACACCAATGCAATGGGATGCTAGTGAACATGCTGGCTTT

ACGACTGGTAAGCCTTGGTTAGAGGTTAGCAAATCTTATCGAGACATCAATGTCGAAACA

GAAAAAGAGGGACGTATTTTTCCTTTCTACCAACGCTTGATTGCTTTGCGGAAGGAACTG

CCTATTATTGCTGAAGGGGACTATCGGGCTGCTTTTAAAGATAGTCAGGCTGTCTATGCC

TTTGAACGCCATTTAGGTGACCAGTGTTTGCTTGTTCTCAATCATTTCTATGCTGATGAG

GTCGAACTGGAATTACCTCCACGTTATCAACATGGACAGGTCTTAATCAGCAACTATGAG

AAAGTCTCTATTTGTGAAAAAGTGATACTGAAACCTTATCAGACACTTGCTATCTTAGCT

GATAACTAACTAAAAGCCTGAGGTTAGGCCTCAGGCTTTTGGTGATTGTCACGAAAAAAG

AAGTTTTCTCATATATTTATTTTTAAACTCTTATTTTTTCTAAATAAAAACAATTGAGAT

AAGTTATTTTAACCAAATTTTGGTACAATGGAAAAGATTATAAGTAGAGTTTAAGATAAA

GATTCTACAAATTTAAAATGATATAAGGAGATACGAATGACAACTTATCAAGATGATTTT

TACCAGGCTGTGAATGGGAAGTGGGCTGAAACAGCAGTTATTCCCGATGACAAGCCTCGA

ACAGGTGGATTTTCAGACTTAGCAGATGAAATTGAGGCATTAATGCTAGACACTACAGAT

GCTTGGTTAGCAGGGGATCATGTCCCTGATGATGCAATTTTAGCAAACTTTGTTAAATTT

CACCGTTTGGTGGCTGACTACGCTAAACGTGATGAAGTTGGAGTAAGTCCAATTCTCCCT

TTAATTGAAGAATATCAGTCCTTAAAATCTTTTTCAGAATTTGTTGCTAATATAGCGAAA

TATGAATTGGCAGGTTTACCAAATGAATTTCCCTTTAGTGTGGCACCAGATTTTATGAAT

GCTCAGCTGAATGTGCTTTGGGCAGAAGCACCAAGCATTCTTTTGCCAGATACAACATAC

TACGAAGAAGGCAACGAAAAAGCTGAGGAATTGCGTGGTATTTGGCGTCAGTCGCAAGAA

AAGCTTTTACCACAATTTGGTTTTTCAACAGAAGAAATCAAGGATCTTTTGGATAAGGTA

ATTGAACTAGATAAGCAATTGGCCAAGTATGTCTTGTCTCGTGAGGAAGGCTCAGAATAT

GCCAAATTATACCATCCTTATGTGTGGGCTGATTTTAAAAAACTAGCTCCAGAGCTTCCT

TTGGATAGCATTTTTGAAAAGATTTTAGGCCAAGTACCTGACAAGGTTATTGTTCCTGAG

GAACGTTTTTGGACAGAATTTGCAGCCACTTATTACTCTGAAGCCAATTGGGATTTACTC

AAGGCTAATTTGATTGTGGACGCGGCTAATGCTTACAATGCTTACTTGACGGATGATATT

CGTGTGGAATCAGGCGCTTATTCGCGCGCTCTTTCAGGAACACCCCAAGCTATGGACAAA

CAAAAAGCAGCCTTTTATTTGGCGCAAGGACCGTTTAGCCAAGCGCTTGGCTTGTGGTAT

ACTGGTCAAAAATTCTCCCCAGAAGCTAAGGCTGATGTGGAAAGCAAGGTAGCACGCATG

ATTGAGGTTTATAAGTCTCGTCTAGAAACGGCTGATTGGTTAGCACCAGCCACACGTGAA

AAAGCCATTACTAAATTAAATGTTATCACGCCACATATTGGTTACCCAGAAAAATTACCA

GAAACCTATGCTAAAAAAGTGATTGATGAGAGCTTGTCATTGGTTGAAAATGCACAGAAC

TTGGCTAAAATCACGATTGCTCACACTTGGAGTAAATGGAACAAGCCAGTTGACCGTAGT

GAGTGGCATATGCCAGCCCACTTAGTCAATGCTTATTATGATCCTCAGCAAAATCAGATT

GTCTTTCCTGCGGCAATCTTACAAGAGCCATTTTATTCTTTAGATCAGAGTTCTTCAGCC

AATTATGGAGGAATTGGTGCAGTCATTGCGCATGAGATTTCACATGCCTTTGACACCAAT

GGTGCTTCTTTTGATGAACATGGTAGCCTAAATGATTGGTGGACACAAGAAGACTATGCT

GCCTTTAAAGAGCGTACAGATAAGATTGTGGCGCAGTTTGACGGCTTAGAGTCACATGGT

GCTAAGGTCAATGGTAAATTGACAGTTTCAGAAAACGTCGCTGATCTAGGTGGGGTTGCT

TGTGCTTTAGAAGCGGCACAATCAGAAGAAGACTTCTCAGCCCGTGACTTCTTTATTAAC

TTTGCGACGATTTGGCGTATGAAGGCCCGTGAGGAGTACATGCAAATGCTAGCTAGCATT

GATGTTCATGCCCCGGGTGAACTGCGAACAAATGTCACCTTAACCAACTTTGATGCTTTC

CATGAGACCTTTGATATTAAAGAAGGCGATGCTATGTGGAGAGCTCCGAAAGACCGTGTG

ATTATTTGGTAATTAGGTAGTTGATAAACCTTCAAATGCCTGTGATTATAAAAATGACTG

ATTCTGTGAAACACAGGATCAGTTTTTTTCTTCCTTATTGGTGTCCGTTTTGACACAACA

AAAAGACAACCCTCAGGAAAGAAGGTTGCCTTTTTTAGACTTATCAGAAATAAGTGATTA

GTTGTTAAGGGCAGCAGCCATAGTAGCTGCAACTTCTGATTCAAAGTCGTTAGCTTTTTT

CTCGATACCTTCACCAACTTCAAAACGTGCAAAAGCAATCGCTTTAGCGTTTACTGAGTC

AAGGTAGGCTTCAACAGTTTTGCTGTCGTCCATGATGTAAACTTGTGCAAGAAGAGTGTA

AGCTTGGTCAACTTTAGTGTTGTCAAGCATGAAGCGGTCCATTTTACCTGGGATGATTTT

GTCCCAGATCTTTTCTGGTTTGCCTTCAGCAGCAAGTTCAGCTTTGATGTCTGCTTCAGC

AGCAGTAATCACGTCGTCTGATAATTGAGCTTTTGAACCGTATTTCAAGAATGGAAGTGC

TGGTTTGTCAACCATTGCACGCGATTCGTTGTCAAGTTCGATAGCGTGGTTCAATTGAGC

AAGTTCATCTTTGATGAATTGTGCATCAAGCTCAGTGTATGAAAGAACAGTTGGTTTCAT

TGCAGCGATATGCATTGATACTTGTTTAGCAAGAGCATCGTCGCCGCCTTCGACAACTGA

GATAACCCCGATACGGCCACCATTATGTTGGTAAGCACCGAAGTGTTGCTCATCTGTTTT

TTCAATCAAAGCAAAACGACGGAATGAGATTTTTTCTCCGATGGTAGCAGTTGCATTAAC

GTAAGCTTCAGCAAGAGTTTCACCTGAAGGCATAACAAGTGCAAGTGCTTCATCGTTGTT

AGCTGGTTTGCCTTCAGCGATAACTTTAGCAGTTGCGTTTACCAATTCAACGAATTGAGC

ATTTTTAGCAACAAAGTCTGTTTCAGCGTTAACTTCAACAACAGCAGCAACGTTACCATG

AACGTAAACGCCTGTTAAACCTTCAGCGGCAACACGGTCAGCTTTTTTAGCCGCTTTAGC

CATTCCTTTTTCACGAAGAAGTTCAACGGCTTTGTCCATGTCACCATCTGTTTCAACAAG

TGCTTTTTTAGCGTCCATAACGCCAGCACCAGATTTTTCACGTAATTCTTTTACAAGCTT

AGCTGTAATTTCTGCCATGTGTAGTATCCTCCAAATGATATTTGTTTTGGTTTCAAAAAA

CGAGACAGAGCGGTTTGCTTTCTGCCCCGTTTGGTTTTCGATTGTTAAAGACAAACGAAG

TTTGCCATAAGAGTTAGGAACTCTGTGCTAATTAAGCGTTGTCGCCTTCGACAACTTCAA

CGATTTCTTCGATCGAATCTGCTTGAGTGTCAGCTTCAAAAGCAACGTCTGCATCTTCAC

CTTGACGGCCTTCGATAATAGCGTCAGCTAATTTAGCAGTGATTAATTTAACGGCGCGGA

TAGCGTCATCGTTAGCTGGAATGATGATATCGATATCATCTGGATCAGCGTTTGTATCAA

CCATCGCTACGACTGGGATACCAAGTTTTTTAGCTTCTTTAACAGCAATTTGTTCTTTAT

GTGGGTCAACAACATACATCACGTCTGGGATACGAGGCATATCTTCGATACCGCCCAAGA

ATTTTTCAAGACGAGCGCGTTGTTTGTTAAGAAGTGCAACTTCTTTCTTAGGAAGAACGT

CAAAGGTTCCTTCTTCTTCCATACGTTTGATTTCTTTCAAACGAGCGATACGTTTTTGGA

TAGTTCCCCAGTTGGTAAGCGTTCCACCCAACCAACGGTGGTTGATGAAGTATTGGCCTG

CACGCGTTGCTTCGTCAGCAACTGCTTCAGCAGCTTGTTTTTTAGTACCAACAAACAAGA

TGACAGCATCGTTTGCTGCAGCATCACGAACAAATTCGTAAGCTTGGTCAGCTAATTTTA

CAGTTTGTTGAAGATCGATAACGTGGATACCGTTACGCTCTGTGAAGATGTATTTAGCCA

TCTTAGGGTTCCAGCGACGAGTTTGGTGACCAAAGTGAACACCAGCCTCAAGAAGTTGTT

TCATTGAAATTACTGCCATGAGTAGTTTCTCCTTTATAAAATGTTTTTTCCTCTTCCAGA

CTTCGACTTGCAGGCTAACCTAGCGGCAACAAGCCCACAATTGGTCTAGAATGAGTATTT

GCCGTCTACACGACCCTACTATATTATCAAAAAAAGAGGTTAAAAGCAAGGAGAAATGCT

ATGGATTCCCTTATTTTTGAGATGACTAGCTTCTTTGTTTATTGGTAAGTGTTAAGACAA

GCTTTTGCTCATTTTGGGTGACACTTGCCAAATCGTTTTATTTAAGATATAGTGAATTTG

ACTAATTTTTGGAGGCGTTTTCATGACGAAGGGTATTCGATTTCAATTGCTTGGCAGTCC

CCACATTTTTCTTGATAACAAAGAACAATTTTTTGCCTTTGCCAAAGCAAATGCTCTCCT

TTATTACTTAGTGGTTAATGGATCTGTTAGCCGCGAGGTGGCGGCGAGTCTACTTTGGGA

AAATAAGAACACGCAAACAGCGAAAAAGAATTTACGAAACGCTATCTACCAGGTCAATAA

AGTCTTGCAAGCTGATGTGATTATTTGTCCCAATCGCAACCTGCTGGTTTTGAATAAAAC

ACTTGATATCAAAACAGATATTAACCTTTTTCTGGCTGATCCGCTAGCTCACTTAGAACT

CTACCAAGGGGAGTTTTTACAGGGCTTTTACCTTAAAAGTGGTGAGGAATTTGATCTTTG

GGTTTCAAAAATGCGTATGCAGTATGAACAGGTATACCTAAAAGCGTGTTACCAAAAAAT

TGAAGAAAAGCTCTCTTTAGATGCCATTGAAGATGTCGAGGAACATTTAAAACAGCTTAT

TGAACGTGATGAATTTGAAGAGAAAAATTATCAGCTCTTGATGAGGCTTTATCAGCAGGG

GAATTGTCCTGGTAAGGTCATCGAAACTTATTATCAGTTGGCTAATGTGTTGGATAAGGA

GCTAGGGATTCAGCCAAGTTTGCAAAGCCAACAGATTTATCAGGAAGTGGTCGCTAAAGA

TCGTAATGAACGCAAAATCAAGCATTTTTTGCGCAACAGTAATCATTTTTTGGGGCGGAT

TGATGAGATTAAGCAGCTGGAAAACTATTTTGCCAACTGTTTGGCCTGCCAAGAGGTAGG

AGCTCTGCTCTTAATTGGTGATACAGGGATTGGGAAACGAACCCTAGCCAGACAGGTCTT

AGCCAATCAAACCCAAACGTTTCAAATTGTCACAGCTAAATGTTTTCGTGAAGAGGCTAT

GGATTCCTTATTGCCTTGGCGTAATATCTTAGATGGCCTGGGAGATTTGGTGATTCAAAA

CCGCTTATTGACCACCAAAGCTTGGAAGGCTGCTCTTAAACGCTGTTTTCCTGTGGCAAC

CATTTTTCAAGAAGATAACAACCAACCCTTTATCAAGGACCACACCAGCTTGTTGGTTTC

CTTTATTGTTGATATTTTACAACATTTGGCAGAAATCAAGGCGCTGGTAATCCTTATTGA

GGATTGTCATTGGATGGATGAGGATAGTTTGACCTTGTTGCAACGGGTCATGAATCAATT

GGTCCACTATCCGATTGCTTTTGTCTTGACCAAGCATCTAGGAACGACTCCTGAGCTCGG

TCTTTGTTTGAATGCTTTGATGAGTCAGGGGCGTTTAGAAAGCATTTGTTTGGAGCCTTT

TAATCGGCAAGAAAGTTTGGCTTATATTAACAGTCAATTGGGTAGTCAGCCAGTGACGGA

AGAAGAAATGGAGCACCTCTATCAAGCCAGCCAAGGCAACCCTTTCTTTTTGTCAGAATA

CACTCATGCCCTACTGCGTCACGAAAAATTTGTGCCTTTAACGCCTGCCATTAAGGCTAA

GTTGGGTCTTAAACTAGCTAATCTAAGTAGTCGTGATGACGCATTGTTAAACTATTTGTC

TTGTTGTCGGAGGCCTATCCCTCTAAATACCCTCGCTCAATTGATGTTACTGCCTTTAGA

AGAAGTGATCGAGATGGTGGATAATCTGGGGCACTACTATATTTTGGTAGAAGAAAGCGT

AGGAGAGGAGGTGTTGATTTCATTTCGTCAGCGAATTATTCAACTCTATAGTTATGACCG

TTTATCTTTGTCAAAAAGGCGTTTATTGCACGGACAAATTGCCAAACGGTTAGAAGATTT

ACTGCCTATTTTGACACCAAGCCCTCACTTATTAGATGACATTGCTTACCATTATCAGGA

ATCACGGCAGGTGATTAAGGCTTTGGAATACAACCTTAACTATCTAGATGCTACCTTGCC

ATTTCAGCATGAGCTGTTTCCCATTTATTCAAAGAGTATTGGCTCCTTGGAAAAGTCAGA

TCGTGACCATCAACGCTTAATGGAAGAGCAGTTTGATAAGATTCGGCAAAGCATTGCGGA

TTTGGAACTGACCTATGATAATAACCGTGATTTCCAGCAGTTGCTTATTCGTTTTTCTTA

CCTTGAAGGCCGTTATGATATTCGAACAGGGAGGTATCAAGAAGGGATTAAACATATTCA

AAAGGTCATCGCTTTGGCAACCGAGCTAAAACAGCCCTCCTTCTTGCTAGAAGGCTATCG

CCAGTTAATTCATTACTGTATCCAAGTGGAAAACAAGCCAGAAATGCGTTATTACACTAG

TCTTTCATTGGAGGCAGCGGTTGCTGCTAATCATTTTGAGGCCATTGCGATTAGTTTGCG

GTTAAACGGTCTTTATCATTTGATTATTGGGGAGTTAAATGAGGCAGAGCGTCTGCTTCA

ACAGTCGATTGACTTCTTTAAGGTGACGCCAGGCTTGCAAGCTAACTATGCGATTCAGAT

TGCTGCTGCCTTAGATTATTTGGGGGAGATCGCCCAGATTCGCTATCAGTTTGAAAAGGC

CGTTGCTTACCAAAAACAAGCCATTGCCTTGACCGAAAACAAGCCAGCTGAGTTATCAGT

GAGTATTTTTTATATTGGATTAGGGATTTCCTACTTTTATTTAGCTGATTTTGAACAAGC

TGAGCAAATCTTGAGTTTGGCTAAGGAAGCTTTGGTCAATCATAGTTATCCTTGGAAAGA

AACGCAACTTGAAATTTACCTGGCCATGATTCAATGGAAAAAGGGTAATTATCAGCCAGC

CTTGACTCTACTTGATTACAGAGAAACCTTGATGTCTAGGTATCGCAATCCGCGAGATAA

GGGCTTGGTTTTCTATTTGATGGCTGTGGTCAAGTACCAATTGATACGTCAAGGAGCTAC

CCTAAGTCAGCAGGAAAAGGAAATGGCAGACCACCTGTTGTCAGAATCCTTTGAGTATTA

TTATGAGATTGCTAGTACTAACCTCAACCCTTACCGAGATTGTCATTTGGTGAGTGAGTT

GAACGATTTGCGTCAACAATTATCTGCTAAAAGCTAAGGAAAACCCTCTGATTCACATCA

GAGGGTTTTAGTTATGTTGTGCCATAATCTGTACGAGGTAAAAGATAAAGGTGGCTGCTA

AATTGGCGGTGTGGTTATCAATATCGTGTGGTGGTGAAACCTCAACCACATCAAAGCCAA

CTAATTTTCCGCTAGCTGCAATGTGTTGCAAAACTAAGACGGCAAGATTTGGGTCAACAC

CGAGGGACTGGATAGCACTAACCCCAGGTGCAGCACCAACTGAGAAACAATCCATGTCGA

TAGTCAGGTAGACCCTTTCTTGCCCTTCTAAGAAACGATCAATGGCTCGGCAGACCTTTT

GATGGCCCATTTGGTAGATGTCTTGACCTGTTAAAAATTGAATGCCCTTGGATTTTGCCA

CAAAGTCAAATAGGAAAAGGTTGTTATTATGTTCTTGAATGCCTAAAACGAAGTATTTAA

AGAGGCGTTTGTCAGCAACCGCATCGTCAAACATTTGTCGAAAGCCTGTCCCTGAATTAG

GGCCTGTTTGGTCGTAAGGCCTTAAATCAAAGTGGGCGTCCATATTGATGACAGCTAGGT

CATCACTTGAGGATAAGCTTTGGCGTAGGCCTAAGTAGTGGCCGTAGGCGGTTTCATGGC

CACCTCCTAAGACGATGGGTTTGAGATTGAGGTCACACATTCGTTTGATGGCTTTGCTTA

AACTGTTTTGCAGTTGTTCCAAAGAGCGATTGGGGCCGTCAATGTTACCCACATCGTAAA

CCATAACTTGGTTTCCTAAATGCCAAGGGAATTTGGCTAGCTGGGTTCGTATGGCAGCTG

GACTTTCGACCGCTCCGACACGACCATTATTGATATAAACGCCCTTATCACTTTTAAAGC

CAATCAAGGCAAAATGTGTCCCTTCAAATGGGGTTAGTGAGCTGTCGTTAAGGTCAAGGA

AAGTCATGACCATGCCCCATTTGGCAGTATATAAGTCATCGTCGATCCCACTATGATAAT

AGCTAGTGGTGGATGGGTAGTAATCTTCTAGCATGCGCGTCATCTCCTTTAGGATTATGA

TAGCAAAATAAAACTTACAGTCAACTCTCTATAGGCTGTCATCACTGGTTTTTAGGAATA

GCAGAGCAATAAAATCGTCTGCTTTTGGTCCCAGTTAGTTAGTCTAAAGAGGCCATGACC

TCTGCCACAGCCCCTCTCAGTTAAAGAAGTACCTTATTCACTAAAATTGGATACTCAACT

CAACTGCCTGCTCAACCGCTTCGAGGAAGCTTGGGTCTTCAATGACTGTTGATGCCTTGT

TTAGTTCATCGTAGATTTCGATGTGTTTATCGTGTTCGATAAAGTTGACTTCCTTGCGGA

AGAGATCGTAGGCAACCTTAGTCCCTTTACCTAGTTCATGATTTTCAGATTTGAGATCAA

GAGCTTGGCAGGCTGCCATGATTTCAGTAGCCACGATGCGGCGAGAATTTTTAAGGATTT

CAAAGGCCTTGCGAGCAGCGGTGGTGCCCATGCTGACAAAGTCTTCTTGGTTCTCACAAG

ATGGGATAGAATCCACACTGGCTGGATGTGCCAAAACCTTGTTTTCAGAAGCAAGAGAGG

CACAGGCGTACTGGGTAATCATAAAGCCTGAATTGAGACCTGGGTATTTGACAAGAAAGG

ATGGCAATTTGCTGAGCTGGCTATTGACCAAACGTTCCACGCGGCGTTCAGAAACATTTC

CAATTTCAGAAATGGCGATGCCTAAGAAGTCAAATGGCTGTGCCATTGGTTCTCCGTGGA

AGTTACCTCCTGAAATAACGTGGCCATCTTTACAGATAATTGGATTATCTGTGACAGAAT

TGATTTCAATGTCAACTTTGGATTTGACATAGGCGATACTGTCTTTGCTAGCCCCATGAA

TTTGAGGCATGCAGCGCAAGGTGTAAGGGTCTTGCACACGAGATTGAGTAGCTACGGTTG

TGTTTTGGCTGCCTTCAATGAGGTTTCGGATATTGCGGGCTGTTGCCAACTGGCCGCTTT

GTGGGCGAATGGTGTGAAGGTTTTCTTCAAATGGACTAGTAATCCCGTTATGCACTTCAA

GGGTTAGGGCACCAGCTAGGTCTGACAGTTTCAATAATTGAATGGCGTCATAGGTTGCAA

GGGCACCGATTGCAGTGAGAACAGTTGTTCCGTTGATAAGGGCGAGTCCTTCTTTAGCGG

CTAAAGAAATCTTGTCAATACCAGCTTTATCAAGAGCTTCTTGACCAGAAAGAAGCTCTC

CTTTGTAGTAAGCTTTCCCTAGACCAAGCATAGGAAGCACCATGTGAGCAAGCGGCGCCA

AATCTCCTGAAGCCCCTAGAGACCCTTTTTCAGGAATGTAAGGATGAACCCCTTTATTGA

GCAGTTCCAACAGCTTTTCAATGGTGGATAGACGAATACCAGAATACCCTTTGACGAGAG

AGTTGATACGGATCAGCATGATAGCGCGCACGGCATCTTCTGGCAATGGGTCACCAAATC

CACTAGCATGGGTACGAATCAAATTTTCTTGGAGTTGAACTGTATCTTCTGGTGAGATGC

TCACGTTACAAAGGGATCCAAAACCGGTGGTCACCCCGTAAACCACCCGTTTTTCACTGA

CAATGTCATCAACAATTTTACGAGAGGCATTCACAGCTTCAATAGCACTATCATCAATGC

GACAGGCTATGCCTTGGCGAGCAATCGCAATCACGTCTTCGATAGTAAGGCTTTCGCCAT

CTAAATTAATCACTCTTGTCATAGTTTTTCTCCTAGATTTGTTCCTTAGGGGGCATTTGG

TCCATTTGGTGGTTACGTCCGTGCATAAGGTAGTACACACTACTTGCAATGATGACACCA

ACTGCACCATAAACAAAGTTCATTGGGTTGTCACCCCAAATCATAACTAAACTAACGATA

ACTGCAAGAATTGGGATAATAGGACCAAATGGTACTCGGAAGATCACATTAGCATCGGGA

TCGTCCTTCCGTAATTTCATAACGGCTAGTGCTGTTGGAATGTATTGGAAGAAGCGGAAA

ATAACACTGAGTTTTGCTAAGTTTTCAAATGAGCCAGTGAGTAGGAGTACAATAGCAATT

GCTCCTGAAACCAAGATAGCAACGAGTGGTGCGCCATTTTGATTTTGCTTGGCAATAGCA

GCTGGTAAGAGCCCTTCATCAGCAATGGCAGCACCGTAACGTGGCACCATGATGGATTCT

CCTATGTTAAGACCTGTAATGGAAATTAAAGCACCGATTGAGACCATCCAAGCGCCAGCA

GGGCCTATCATTTTAACAAAGGCATCTTGAACAGGTGCATTGGTCATCATGATTTGAGAG

CCAAGCATGGCAATGGTTCCACCGATAATCAGCATATATAAGACAGAGACGATACTGATT

GATCCTAAAAGAGCACGCGGTACATTTTTTTCAGGGTCACGCATTTCCCCAGCGACAATA

GAGAGGGTTTCAAAACCGATAAAGCCATAGAAGATATAAACAGCAGTATTGGAGATAGCC

CCTAAGAGGTTAGTCCCTGGTTCCAACTGAACAAATGGGGTAAAGTTAGGAAGCCCGTTT

TTGATGAAGAAAAGGGTACAGGCACAAAAGGCAACGATTGGAATTAACTTAGCAATGGTG

GCTGTGATGGTGACAATTTTAGAGGTTTTCAAACCAGCGATATTCATCAAAGATAGTAAG

ATGATTAACCCAATGCTAAGTGGAATGTGCCAACCTTCAAAGGCAGGGAAAGTAATGATA

AACATTCTGGCAAACCCAGCTGCCATGGCTGCCCAAGCGAAGATGGTAACGGTCCAACCA

AGGAAGCCAACGTTAAAGCCAATAAAATCACCAAAAGCTCGTTTGGAATATTGGAAGGCC

CCGCCGTTTTTCCCAAAATAACCAGAGACTTCTGCAAAACAAACTGCCAACATGATGGTC

AAAATAGCTGTTCCAAACATAACAGCGATGGATGCAGGGCCAAGGCCCTTGTAAATGGCC

CTAGGTAAGAGAAAAATACCAGAGCCGATAACGGCATTGATCCCGTAAAGAGTGGCACCG

CTCAAGCTAAACTTTGCCTGTTCTCTTTCTTGTTCGTTCATGTGATTTGACGCCATAATA

ATGCTCTCCTTTTTGTTTGCAAATTGCGTTCAAATATAATGGATAAGGTCGCTAGGAAAT

GAGGAGATAGTCGGTTTAGGAGGGTCCTTCTTGCACCTAACGATAGTGATGAGCTATTGG

TTAGCGATGAGAAATAGGTAGCCTTTTGTGATGATGTGTAGTGATTTCATTGGAGAAGAA

TAGGCATGAGTAGTGTGATGCCTATCTTTATCCCACTAAGTCTTATACCATAAGCCTGCC

CAAACAGCTACTGGAGTTTGGGCGGGGCTTGTTTGATTGAGGCAAACTTTTAGATCTGAA

AGGCTGGCCTCCTGCTCGATTACCAACAAGTGATTGGCTTCAAGCTCTTGATTCGCCTGA

TTGGTCACAGAATAAGTCAGTTTGCTTAGGGCATAAAGCAGTTGACAGTCCGCTTTAGGT

GCCTCAAATAGTGTGTCAGACCAGACGGGTAATAATTGCCCTTGGTATTGGTCATCATAC

ATTAAATTCATGTCTTGGCAGATTCCACGACTGACGATTCTGGTATCGCCTTCAAACGCA

TAAGGTTCAAAAGGAGTTAGGGAAATCTCTTTAGCATGACTCAAATCACTCAATGTGATG

GGCTTGTCTAGGGTCATTAGAATCCTGTGATAGCCATCAAGTAAGGTAAAGGTTGTCTGT

CGTAGGGCCACAGTTGACTTTGAGAGGCGATAAGAGAATTCTCTCTTTTTGTAATCTCCA

TCTTCGGGATAAAGGAACAATTGGTTGGTTTCACCGCCAGACCAGTCAGTTTGGAGGTAA

TCAGAGGGCGCTTTGATAGTTACCTTTGTCATTTGTTCTCTCCTTGTTTCCAATTAGAAT

AGTCCTGTGATGTTGCCCTCCTCGTCAATATCAATGGTTTCGCTGGCAGGTACTTTTGGA

AGGCCAGGCATGGTCATGATAGCACCCGTCAGAGCAACGATAAATCCTGCTCCTGCAGAC

ACTTTGAGGTTGCTAATAGTCACTGTAAAGTCAGTTGGTGCGCCAAGTGTTTTGGCATCG

TCTGAGAAGGAATATTGGGTTTTGGCCATGCAAATTGGGTAGTTGCCAAAGCCTAGGCGT

TCTAAGTCAGCCAGTTCACGTTTGGCTGCTGGGCTCAAGGTGATGCCTTTACCACCGTAA

ACTTTGGTAACGATTTTAGTCAGTTTGGTTTCAATGCTATCGTCTTCCTCATAAACAAAG

CGGAATTGATTGTCTTGCTCGGCTAGTGTCACGACTTTTTCAGCCAGTTCACGGCCACCA

GCTCCGCCATTTGCCCAAACATCAGAAATCACAACGTCAACGCCACGTTTGTTGCAGGCA

TCATAGACAGCTTGTAATTCAGCATCGGTATCAAGCGGGAATTTATTGATTGCAACGACA

ACTGGAAGGCCATAGACGTCTTGGATATTAGCCAAATGTTTGTCAAGGTTAGGCAAACCA

TCCACAACGGCTTGAACATTTTCAGTAGCCAAGTCAGTTTTTTGAACGCCACCGTGCATT

TTAAGGGCACGAATAGTGGCTACTAGGACAACGGCTGCTGGACGAAGGCCTGACATGCGG

CATTTGATGTCAATGAATTTTTCAGCTCCAAGGTCAGCACCAAATCCAGCTTCGGTAACG

GCATAATCACCATATTTCAAGGCTAGTTTGGTGGCGAGGACACTATTACAGCCATGGGCG

ATATTAGCAAATGGGCCACCGTGAATGAGGGCTGGTGTGTGTTCCAGGGTTTGCACGAGA

TTAGGATGAATCGCGTCCTTGAGCAGAGCGGCTAGGGCACCACCAGCTTTCAAGTCTTTA

GCTGTTACAGGTTCGCCTTGATAGTTGTAACCGATGATGATTTTTTCAAGGCGGGCTTTG

AGGTCAGAGATATTTTCGGATAGGCACAGGATTGCCATGATTTCAGAGGCGACGGTAATG

TCGTAACCATCCTCACGAGGGACCCCGTTGACCTTGCCTTGCAAGCCGTCAACAATATGA

CGAAGCTGGCGGTCATTCATATCCACCACACGTTTCCAAGTAATACGTCTGGAATCAATG

CCTAGGCTATTGCCATGGTGAATGTGGTTATCAATCAAGGCAGCTAATAAGTTGTTGGCG

ACACCGATGGCATGAAAGTCCCCTGTAAAGTGAAGGTTGATGTCTTCCATTGGGACAACT

TGCGCGTGGCCACCGCCAGCAGCTCCTCCCTTAACCCCAAAAACAGGACCAAGAGAAGGT

TCACGAAGGGCAATAACTGCTTTTTTTCCAATGGCAGAAAGAGCATCTACCAATCCAACG

GAAGTGGTGGTTTTGCCTTCACCAGCTGGTGTTGGTGAGATAGCAGTGACAAGAATGAGT

TTGCCATCAGGTTTGTCTTTTAAGGCGACCAGTTGACGGGCATTAATTTTTGCCTTGTAC

TTACCATAGAGACAGAGCGCTTCCTTGTCGATCCCTAATTGATCGGCAACCTTACTGATA

GGTTCCATAGAGACAGAGTTGGCTATTTCAATATCTGATAAAACCATAGTTGCTCCTTTA

CTTGTTAAACAATATCGAGAATTTTCGTGTAGATGTCATCTGCCAAGTGACAGCCCTTGT

CTAATAAGGCTTGTCCTTTTTGGCGATAGTCGGTAACAAAGTCTTCATCTTTGATGCCAG

ACAGATTAATCAAAACATTCAACCAAGCGCCTTGTAAACCTGCTTTTAAGTTTAAAGCAG

CAACTCCTAAGTCGCTAGCAGCATTTGTATTAGACTTACCGACAGCTGTTGCAGTGATCT

CAAGTGCTTCGACCATGAGGGTCATCATCTCAAAAGGTGATTGTGCAGCTGTTTTCAAGG

CCTTTTGCATGGCAGTTCGTCTAGCAGCCTTGTCTTCGTCAGTCTCTTTTGGCATATCAA

AAACGGCAGAAACCAGGTTGAAGGCTTCGGTATCTTTGTCAATGGCTGCAAGCAAGCTAG

CTTGTAGAGCGGTGCTTTTAGCATGGACCTCTGTAATAATATCTTGGTAGTCAGCGTATT

TTTTCTTACCGAGGGTCAATTCACAGACCATCTTAGTCAAGGAAATGCCATTAGCGCCAG

AGAGAGCAGCAGCAGAACCACCACCTGGAGCAGGGGCATCTGAGCCAAGGACTTTAGCGA

AATCTGTTAAGCTTAAATCAACTAAACCCATAGAGAACCTCCTAACCTAGCAAATGGTTT

TCAAGAACCTGTTTATGGTAGTCAAAGTCCTCAACTTGTAAGTAGTATTCAGCCACATCG

ATTAAGGCCTTGGCTGGCGCTAAGCCGATGACTTCAGAACCAATAACATTCACACCGTAG

CGACGCGCTTCAAATTTGATGGTTTCAAAAGTACGGTAAAGAGAACATTTTTCAAAGTTA

ACCATGTTCATGGAGACTTGGGCAATATGGCGGTCTTCTAACATGACACCGATAGCCTTA

CAATATTTGTAGCCACCACCTGAACCGCGGATAATTTTCGCAATTTTATGAGCAATGTCG

ATATTGTCAGTATCCAAATTAACATTGAAGGCAACCAATGGCATGCGAGCACCAACGGCA

GTTACCCCAGCAGTTGGGTGAATCTTACGATCTCCGTAGTCAGGTGCCCAATCCTCTTCC

AACAGTTTTTCTGGCATGCCTTCGAATTGTCCTTTACGGACTTTGGCCAGGTTTTGACGT

TCTGGGCGTGTGGCAGAATCTTCATAAAGGAAGATTGGGATTCCAAGTTCACGGTTAATC

CGTTCTGCGACTTGTTTGGAAATCTCAACACATTCTTGTGTAGTGATGTCTTTGATCGGA

ACGAATGGGCAGACATCGGTTGCTCCCATACGAGGGTGTTCGCCATGATGTTTGGTCATA

TCAATGTTTTCAGAAGCGTATTTCACCAGTTGGAAAGCTGCTTCTTGAATAGACTGGTCA

TCTCCAACCAAGGTAAACACGCTCCGGTTGTGGCTCGCATCAGAAGAGTAGTCGAGAAGG

GTCACTCCAGGAATACTTTTGGCCGTCGCTACCAAACCATCGATAACAGCCTGATTTTGG

CCTTCTGAGAAGTTAGGAATACATTCAACAATTTTTGCCATGTTAATCTCCTTTTATGTC

ATTGAGTTACTCATTAAGGGAATAGGTTACCTACTTTAGATAACGCTTACATTTTTAGCT

AGCAGTTATTAGTGAAATAATTGATCTACCGCTGAAGTCACAAGGTCATCGTCAGCCAAA

TATGGGATAGTGATATGATCAGTACCTGCATGGAGACGGTTGTATTCAATAGCGGTTTCA

ATAGCGTGCTCATTGCGAGCCCAATTGCGACGAGCAACACCACCCATGGTGTCCCAAGCA

ATGGCAGATTTAATAATCTCATCAATGCGTTCGCTGCCATCTAAAACAAGACCGAAACCA

CCATTGATGGCTTTACCAATACCAGTTCCGCCACCGTTATGAAGAGCCACTAAACTCATA

CCACGCGCAGCGTTACCAGCGTAACATTGAACCGCCATATCACAGGTCACATTGGAACCG

TCTTTAATGTTTGAGGTTTCACGAAATGGTGAGTCGGTACCAGACACGTCATGGTGGTCA

CGACCAATCATGACAGGACCAATCTTGCCTTTGCGGACCAATTCATTAAATTTAAGGGCG

ATAGTCACACGACCGATACAGTCTTGGTAAAGGATCCGGGCTTGGGTACCAACTACGAGT

TGATTTTTCTCAGCATCACGAATCCAGTTGTAGTTATCACGGTCTTGATAACGGCGGTCA

GGATCGATAGCTTCCATGGCAGCTTTGTCAGTGGCCACCAAGTCTTCGTGTTTACCACTC

AAGCAGACCCAACGGAAAGGTCCATAGCCATAGTCAAAAAGCATAGGTCCCATGATGTCT

TCAACATAAGAAGGCCAGATAAAGCCGTCTTTGTCGTTACGACCATTCTTAGAAATTTCT

GTAATACCAGAGTCGTAAACAGACTTCATAAAGGCATTACCGTAGTCAAAGAAATAGGTG

CCATTTTCAGTTAACGTCTTAATAGCTTCAAAATGCCGTGCCAAGGTATCATCAACCATT

TGGTGGAAGGTATCCTTGTCTTCTGCCAAGAGCCGGGTTCTTTCGTCAAAGCTAATGCCA

GCTGGGCAGTAGCCACCGTCATAAATGTTGTGACAAGAGGTTTGGTCAGACAAAAGATCA

ACATGAATCTGCTTGTCATTGACGTACTCTAAAAGATCAACGATATTACCATGGTAAGCA

ATCGAAGTGGACTCCTTAGCATCTATGGCTTTTTGGGCTAATTGCAGAGCTTCTTCAGGG

CTCTCAGCAATTTGGCTAACCCAACCTTGCGAATGACGGGTTTCAATCCGAGATTGGTCA

ACTTCAGCAATAATGGCAACTGCTTTAGCAATTTCAGCAGCCTTACCTTGTGCCCCACTC

ATGCCTCCTAAACCAGATGAGATAAAGAGTTTACCGGTCAAATCACCATCGTCAGCCACT

CCAAGTTTGAGACGACCAGCATTTAAGAGAGTATTAAATGTTCCATGAACGATTCCTTGT

GGGCCAATATACATCCAACCACCTGCGGTCATTTGACCATAGTTGGTGACACCCATCTCT

TCAGCGATTTCCCAGTCTTTCATGTTGTCGTATTCACCCACCAAAAGGCCATTAGTAATA

ATCACACGAGGCGCTTCAGGTTTGGATTTGAAAAGACCAACTGGATGACCAGACTCAACC

ACCAAGGTTTGTTCATCAGTCATGACTTCTAGGTATTTTTTAATCAAACAATATTGCATC

CAGTTGGCACAAACAGAACCAGTTTCTCCATATGTGACTAGTTCGTAAGGGTACAAGGCA

ATTTCAAAACTCAAGTTATTGTCAATCATGACCTGCATGGCCTTGGCAGCTGTACAATTT

CCCTTGTACTCGTCAATGGGTTTGCCGTAAATGCGGTCTTTTGGTCTAAAGCGGTAGCCA

TAAATACGGCCACGGGTTTTGAGTTCTTCCAAAAATTCTGGAATGACTTCTTCGTGGAAC

TTCGTAGGTACATAGCGTAGGGCATTTTTCAAGGCGATTTCGGTCTGAGCCTGGGTCAAT

CGAAAACCTCTGTCTGGTGCTCTTCGGATACCTTCTTCAAACACAGTCTTTTCCGGTAAA

ACATCGTCTAACTTAACAGTCATAGCGGCAGCTATGTCAGTTTCACTGTAAAATGACATA

GAAAACTCCTCCTCAACTCCTTTTTATTGTCGAGATTAGTCTAACTTAGTCTAGTCTATA

AAAAGTCAACCACGCTTTGAAATGCGAAAAAAGTCTAAAAAAAAATCAAAACCTCATTTT

TTGACTAAAATTTGACGCTTAATCAGTACACTTGAGGTATGACAGATAAAGTCAGTCAGA

GGTTAATTGTTGTTACCAAAGTTGCAAAGATCGCAACTATTGAGTGGGTGAGGGGGGACT

AGTAATGGCTATTAATCAGTTGACAAACGATGTTAAAAAAGGAGGTTTTTATGGTTGCTG

ATGTCTTATTAACACATTTTAATCAACTATTTTGTCTAAATGATCCAGGTCATCCTTTAA

CTGGTCAAGAAATGAAAAAAGCCACTATCGTTGAAGATGGCTATATAGCCATCAAAGACA

GCCTTATTGTGGCTCTTGGTTCTGGTGAACCAGATGCTGAGCTTGTCGGTCCACAGACTA

TTATGCGTTCTTATAAAGGTAAAATTGCAACGCCTGGTATTATTGACTGTCATACCCATT

TGGTCTATGGTGGCAGTCGTGAACATGAATTTGCCAAGAAATTAGCAGGGGTGTCTTACT

TGGATATTTTAGCTCAAGGTGGTGGTATTTTAAGCACGGTGAGGGCCACTCGGTCAGCCA

GCTTTGACAACCTTTATCAAAAATCCAAGAGATTGCTAGACTACATGTTGCTTCATGGAG

TGACTACTGTCGAAGCGAAAAGTGGCTACGGCCTTGATTGGGAAACAGAAAAACGCCAAC

TGGATGTTGTTGCTGCTTTAGAAAAAGATCATCCCATTGATTTGGTGTCTACGTTTATGG

CAGCCCATGCGATTCCTGAAGAATACAAAGGGAATCCTAAGGCTTATCTCGATGTGATTA

TCAAGGATATGCTTCCTGTTGTTAAAGAGGAAAATCTGGCAGAATTTTGTGATATTTTCT

GTGAAAAAAATGTTTTTACAGCTGATGAGTCTCGCTATCTGTTAAGCAAAGCCAAAGAAA

TGGGCTTCAAGCTTCGTATTCACGCAGATGAAATTGCGTCTATAGGCGGAGTTGATGTCG

CAGCAGAGCTCAGCGCAGTTAGTGCAGAGCATTTGATGATGATAACAGATGATGGTATCG

CTAAGTTGATTGGTGCTGGTGTTATTGGTAATTTGCTTCCAGCCACCACCTTTAGTTTAA

TGGAAGACACTTATGCCCCCGCTCGCAAAATGATCGATGCCGGCATGGCTATCACTCTCT

CAACAGATAGTAATCCAGGCAGTTGCCCGACTGCTAACATGCAGTTTGTGATGCAATTAG

GTTGTTTTATGTTACGTTTAACACCGATTGAAGTCTTAAATGCTGTTACCATTAACGCGG

CCTACTCTGTCAATCGTCAGGAGAGAGTTGGTAGTTTGACAGTTGGTAAAGAAGCAGATA

TTGCCATTTTTGATGCCCCAAATATTGATTACCCATTTTATTTCTTTGCCACCAACTTGA

TTCACCAAGTCTATAAAAAAGGACAACTCACAGTTGACCGAGGTCGTATTCTTTAAAAAA

TTTAAAAAAGAAGCTTTGAGCGTTAGCTCAAAACTTCTTTTAAATATGCTGTTTTATTGA

CGAATCAAGTAGTCAAAGGCACCGATGGCAGCAGTAGCACCAGACCCCATGGAAATAATG

ATTTGTTTATAAGCTGAATCAGTACAGTCCCCAGCAGCAAAGATACCAGGAATATTGGTT

GACCCATGTTTATCCACAATGATTTCTCCGCGGTCGGTTAGGTTGACACCACTATCTTTG

AGCCAAGCTGTGTTTGGAACAAGACCAATTTGAACAAAGACCCCTTCAAGGTCAAGGTGT

TTGTCTTCACCGCTATCACGCTCAGTGTAGTTAAGACCTGTAACATGGTCCTCTCCAACA

ATGTCCTTAGTAGAAACATTTTTGATGATGGTCATATTGTTAGTCTTAGCTGCGCGGTCT

TGAAGCACTTTGTCAGCTTTAAGTTCAGGCAAGAATTCTAAAACGTAAACGTGTTTAGCA

AGACCAGCCAAGTCAAGGGCCGCTTCTAATCCAGAGTTCCCACCACCGATAACAGCAACA

TCTTTGCCTTCAAAGAGAGGACCGTCACAGTGAGGGCAGTAAGTCACCCCTTTATTACGG

AATTCATCTTCACCAGGAACATTAATGTTACGCCATTTGGCCCCAAGAGCTAAGATAGCA

GTTTTGGCTTGTAGAACCGCACCATTAGCTAGAGTAACCTCAATGTTTTCTTTTTTCTCA

ATAGAAGTAGCTAGTTGTGCTTTGATAATATCAACATCATAAGACTTAGTATGGGCCTCC

ACTTCGGCCATTAATTTTGGTCCTTCAGTGTAAAGGGTACCAATCATGTTTTCGATGCCG

ACCGTTTCCATGACCTGACCACCAAAGGTTTCAGCAAGTAAACCTGTTTTTAGTCCTTTA

CGAGCTGCATAGATAGCAGCGCTATTGCCCGCAGGGCCACCACCAATAACAAGGACATCA

TAGAGGCCTTTATCAGCAAAGGCTTCCTCAGAAAGTGGGCCAGCGATTTGTTCTAAGAGT

TGCTCAATGGTAGCGCGACCAGAAGTGAATTCTTCCCCGTCTAAAAAGACAGTAGGGACA

GACATAATGCCTTTTGCTTTCACTTCATCTTGGAACATGCCGCCTTCTACCATAGTGTGT

GAAATCTTGTCGTTCAAGACGGACATAATGTTTAAAGCTTGAACAACATCTGGGCAGTTA

TGGCAGGTTAAGCTGACATAAGTTTCAAAATGCAACGGACGGTCAATGGCCTTGATGCGA

TCAATAACATCTTGATCCACTTTTGGAGCACGTCCAGATACTTGCAAGAGAGCAAGAATA

AAGGACGTCAATTCGTGGCCTAAAGGAAGACCAGCAAAGACAACCCCACTACCGTGACCT

TTTTTCGCAACCTTAAAGCTTGGTTGTCGGTCTAAAGTGATGTTTTCAATAGAAATACGC

TCAGACATAGCAGCTATTTCTTCCACAAAGTCTTTGACTTTTTGAGACTGCTCATTGTCA

CCAAGGGAAACTTGCAAAACAAGATCAGCTTCTAACAGGGTGAGGTATTGGGCAAGTTGT

TCTTTAATATCAGGACTTAATGCCATAGCTTTCCTCCTAAAATTTATTAAATTTTACCAA

CTAAATCAAGACTTGGTGTCAAAGTTTCAGCGCCTTCTTTCCATTTAGCTGGACAAACTT

CACCTGGATGTTTACGGACGTATTGGGCAGCGTGAATTTTATCAATCAAGGTGCTAGCGT

CACGTCCAATACCATCAGCATTAATTTCCATCATTTGGATAATACCATCTGGATCAACGA

TAAATGTTCCACGTTGAGCAAGTCCGTCTTCGCCAAGCACTTCAAAGGCTTGTGAAATAA

GGTGTGAAGGGTCACCAATCATAGGGTATGTGATAGTGCCAACCACATCTGAATCATCAT

GCCAAGCTTTATGAACAAAATGAGTATCAGTAGAGACAGAATAAACTTCTACACCAAGAG

ATTTCAGTGTTTCGTATTGCTTTTGAAGGTCACCGAGTTCAGTTGGGCAAACAAATGAAA

AGTCTGCTGGGTAGAAACAAAAAACTGCCCATTTTCCTTTAACGTCTTCATTTGTAACAG

TGATGAATTTTCCATCGTGATAAGCTTGAGCTGAAAATTCAGCAATTTCTTTTCCAATTA

GAGACATATAGTTGTCCTCCTTTTTTATAGTTTTATACTTGTATTATAAAAGAACAACAA

GATTATGTCTACTCAGAAACCCTTGATTTTACTGGGAAATCAGCGATTGTTTTCTGACAA

TTCAGTTGTGAAACAAATCACATTAATTAAAATGATTCTAAATAAGCTGAAGATGCTGTT

AAATCAAAGGTTAATTTAATTATAATCTACAGAAAATATAATTATTATTAAAGCGTTTAC

AAAAAACTTTTTTGATTCCGTTAAGGCAACTTAATATTATGGTTACCTAATCACTAATAT

TGTAACCTATTTTGGCATGAGATCAGGGAATGGCTGTTATATTAATTGGATATGTCTTGT

GCAAATAGTGCTTGACGGATTTGTGGAATTAGGGTAAAATAATCTTTGTTGCGGCGGTAT

AGCCAAGTGGTAAGGCACGGCTCTGCAAAAGCTTGATCGTCGGTTCAAATCCGTCTACCG

CCTTTTATATACAAAACAAGCCTTGATTAACTTCGTGAAAACGTTGTTAAATCAAGGTTT

TTATTTTTTGCGACAGGATAAAAAAAGAGAAAAAAAGAAATAAAGTCGGTCAAATTTCCC

GAAGATGTCTTTTATAGCCTGATTATTAGGTATGAAAAATACTATGGCCAAAATTGGTAA

ACGATTGCAAATAAAGGTCAGAGTTCTTACCCCCTTAAACGATATGCGTTAAAAGTTAGT

TGAACTGCCGTGCACCTAACGATAGACACGGTGATGTGAGAGCGAGTAGAGTTGAATCTC

TACTTGTTTATCTTACCATAGTCAGCTTTATAAAGTAACCACCCATTACAATGCAACTAT

ATTCTTAATGTTTTTAAGCATGTTATTGACAAGTGTTATAAAATGTGTTACTCTTAGTGA

GTAAATTAGTTTTTGAGTTAGTATTTGTGTTTCAACATCTTTATTTCTCTCTATTTTTAC

AAATGATTTTTAGAAAGGAAGTGGATGTATATGGCACAAGGTACAGTAAAATGGTTTAAC

GCTGAAAAAGGTTTTGGTTTTATTTCAACTGAAAACGGTCAAGATGTCTTTGCACATTTT

TCAGCCATCCAAACTAATGGTTTTAAAACGTTAGAAGAAGGACAAAAAGTGGCATTTGAC

GTCGAAGAAGGTCAACGTGGACCTCAAGCAGTCAATATCACTAAATTGGCCTAATCTGGT

TAAAGTAACTAAAGAGAAGCAGATGTTGGAAACAGCAACTGCTTTTTTGATTTCTACCAG

TTACCTTTAATGATTAAGAGTTGCGTTTAAGGTCTTGTATTTGTTATAATAATAGTCAGT

TTTAGTCAAAATGTATTGACGTGGTGCTACTAAGTTTTTAGAGTAGCCCGCAAGCAAAGG

AGTGACAGCATGCCAACCAAAAATACTTCAGACAGCATTGAAGAGTATATTAAAGAATTA

TTAGCTAAGTCAGGAATAGCAGAAATTAAGCGGTCCATGCTAGCAGATTCGTTTCAAGTT

GTCCCAAGTCAGATCAACTATGTCATTAAAACCCGTTTTACGGAGAGTCGAGGCTATGAA

GTAGAGAGTAAACGAGGTGGTGGAGGCTATATTCGTATTGCCAAGGTTCATTTTTCAGAC

AAACACCATCTGATTGGCAATTTAATGGCAACTATTGAAGACTGTATCAGTGAGCAAGTT

TTCACGGACTCGATACAATTGCTTTTTGACGAACACTTATTGACAGAGCGTGAGGGAAAT

ATCATTTTGGCTGTGGCGTCTGATGATGTTTTAGGAACTGATGGTTCAACTATTCGTGCA

CGAATGCTCTATCGCTTATTGCAACGAATTGACAGAAAGGGAAGCAATTAATGATTATGT

ATTCAACGAAGATGCAAGACATTTTTAGACAGGCGCAGTTCCAAGCTGCTCGCTTTGATA

GCCACTGCCTGGAAACTTGGCATGTTTTGTTAGCTATGGTAGCTGTAGACAATTCTTTAG

CAAATATGATTTTAAGTGAATATGATGCCCAAGTCGCCATAGAAGAATATGAAGCTGCAG

CTATTTTAGCCATGGGCAAAACCCCTAAGGAACAGTTGTCTCGTGTAGACTTCAGACCTC

AATCTAAAACTTTGACTAACTTGTTAGCTTTTGCGCAGGCTATTAGCCAAATCACTAGGG

ATCAAGAAGTCGGCTCTGAGCATGTCTTATTTGCTATTTTATTGAATCCAGATATTATGG

CGAGTCGCTTGTTAGAAATAGCAGGCTATCAGATAAAAGATAGCGGCAATGGGCAGCCGC

GATTAGCTGACTTACGAAAAGCAATAGAACGTCATGCAGGTTACAGTAAGGAAATGATCA

AGGCTATTCACGAACTACGTAAGCCTAAAAAAACGAAAACACAAGGGACCTTTTCAGATA

TGATGAAGCCACCAAGTACAGCTGGTGAGTTGAGTGATTTCACAAGAGATTTGACTGAAA

TGGCAAGACAAGGTTTGTTAGAATCGGTGATTGGACGTGACCAAGAAGTATCTCGTATGA

TTCAGGTATTAAGTCGTAAAACGAAAAACAATCCTGTCTTGGTAGGTGATGCAGGTGTTG

GTAAAACGGCGCTTGCTTATGGCCTTGCTCAACGGATTGCAAATGGCGCTATTCCTTATG

AACTTAAGGAGATGCGTGTCCTAGAATTAGACATGATGAGTGTGGTAGCAGGAACCCGTT

TTCGTGGGGATTTTGAAGAGCGCATGAATCAAATCATTGATGATATTGAAGCTGATGGTC

AGATTATTCTTTTTGTTGATGAACTACATACTATTATGGGTTCTGGCAGTGGTATTGACA

GTACACTTGATGCGGCTAACATTTTAAAACCAGCATTATCGCGCGGCACTCTTCATATGG

TTGGAGCAACAACTCAAGAAGAATATCAAAAACATATTGAAAAAGATGCAGCTCTTTCGC

GTCGTTTTGCTAAAATATTAATTGAAGAACCTAATACAGAAGATGCTTATCAGATTTTGA

TGGGCCTAAAATTATCTTATGAGACCTACCATAATGTCTCGATATCAAATGAAGCAGTTA

AAACAGCTGTAAAAATGGCACACCGTTATTTAACCAGTAAAAATCTCCCTGATTCAGCTA

TCGATTTATTAGATGAAGCTAGTGCTGCTGTGCAAAACATGGTGAAAAAATTAGCACCTG

AGACTTTAACACCAATAGACCAAGCTCTTATCAATGGTGATATGAAAAAAGTATCTCGCC

TCTTAGCTAAAGAAGCAAAAGGTCAGATGAGAAAACCAACACCAGTGACAGAAGATGATA

TTTTGGCAACCTTGAGTAAGTTATCGGGAATTCCACTTGAAAAACTGACGCAAGCTGATA

GTAAAAAATACCTCAATTTAGAAAAAGAACTGCATAAGCGTGTGATTGGTCAGGATGCTG

CTGTTACGGCTATTTCAAGAGCCATTCGTCGCAATCAGTCAGGTATTCGAACAGGAAAAC

GTCCTATTGGATCATTTATGTTTCTTGGCCCAACAGGAGTAGGTAAGACAGAACTAGCAA

AGGCCCTTGCAGAAGTTCTCTTTGATGATGAAGCAGCGCTTATTCGTTTTGATATGTCTG

AGTACATGGAAAAATTCGCAGCGTCTAGGCTTAATGGAGCACCTCCTGGTTATGTCGGCT

ATGATGAAGGAGGTGAACTGACACAGAAAGTTAGAAATAAACCTTATTCAGTCTTGCTTT

TTGATGAAGTGGAAAAAGCACATCCTGATATTTTTAACGTTCTCCTTCAAGTATTAGATG

ATGGTATATTGACTGATAGTCGTGGGCGTAAGGTCGATTTTTCAAATACTATTATTATCA

TGACCAGCAATCTTGGCGCAACAGCCCTGCGCGATGATAAAACGGTCGGTTTTGGGGTCA

AAGACATTCACCAAGACCATCAAGCTATGGAGAAACGTATTTTAGAAGAATTAAGAAAAA

CTTACCGCCCAGAATTTATCAATCGTATTGATGAAAAAGTGGTCTTTCATAGTCTGACCC

AAGATAACATGCGCGATGTGGTTAAAATCATGGTACGGCCCCTGATTACTACATTGGCAG

AAAAAGGTATTACCCTTAAAATTCAGCCTTTGGCCTTGAAACATTTGTCCGAGGTCGGCT

ATGATGAGCATATGGGGGCAAGACCATTACGTCGAACGCTGCAAACTGAGATAGAAGATA

AGCTATCAGAGCTTATCCTTTCTCGAGAATTGACAAGTGGGCATACGCTAAAAATTGGAT

TATCACATGGCAAATTAACGTTTCACATAGCTTAATTTCCATATAAAAGGTGGATCATCC

TTGATGTCACCAAAAAAAGAGGTTATTCTTACAAAAATAACTTCTTTTTTCTTGACTATT

TTTGACCAAGTGATAAAATAGAATATAAAGTTAGCACTCGATGAACATGAGTGCTAACTT

TACTAAAGATTCTTTTATTGGAGGAAAATTTCATGTTAAAACCATTAGGTGACCGTGTGG

TCGTAAGATTTGATGATGAAAAAGAGCAGACAGTCGGTGGTTTTGTCCTTGCAGGCACTC

ATAAAGAGTCAACACGAAAAGCGACAGTCCTCGCTGTCAGTGAAACGGGTGTTCGCACTA

TTACAGGTGATAGCGTGCTACCTTCAGTTTCAGTGGGTCAAGAGGTTTTAGTTGAAAATG

GGCATGATTTAGAAGTAACCGTAGATGATGAGAAAGTTTCTATCATCCGTGAATCTGACA

TCATTGCTATTGTTACAAAATAAAACGACAAATCTCTAAATAGAAAGAAGGATTGAATAT

GGCAAAAGATATTAAATTTTCAGCAGATGCGCGTGCTGCCATGGTGCGCGGAGTTGATAT

GTTAGCAGATACCGTCAAAGTAACGCTTGGTCCTAAAGGGCGCAATGTTGTTCTTGAAAA

AGCTTTTGGTTCTCCCTTAATTACTAATGACGGGGTAACCATTGCTAAAGAGATCGAATT

AGAAGATCATTTTGAAAACATGGGAGCAAAATTGGTGTCTGAAGTGGCTTCTAAAACCAA

TGATATTGCTGGTGATGGGACGACTACTGCAACAGTTTTGACACAAGCCATTGTTCATGA

AGGACTAAAAAATGTGACAGCAGGTGCTAATCCAATTGGTATCCGTCGAGGCATTGAAAC

AGCAACAGCAACAGCCGTTGAAGCCTTGAAAGCCATTGCTCAACCTGTATCTGGCAAGGA

AGCTATTGCTCAGGTCGCTGCAGTATCATCACGCTCTGAAAAAGTTGGAGAGTATATCTC

AGAAGCTATGGAGCGTGTGGGCAACGATGGTGTGATTACCATCGAAGAATCTCGAGGTAT

GGAAACAGAACTTGAAGTGGTTGAAGGCATGCAATTTGACCGTGGTTACCTGTCTCAATA

CATGGTCACAGACAATGAAAAAATGGTTGCAGACCTTGAAAACCCATTTATCTTAATCAC

GGATAAAAAAGTGTCAAACATCCAAGACATTTTGCCACTACTTGAGGAAGTTCTTAAAAC

CAACCGTCCATTACTCATTATTGCAGATGATGTGGATGGTGAAGCCCTTCCAACCCTTGT

CTTGAACAAGATTCGTGGTACTTTCAATGTGGTTGCTGTCAAAGCGCCAGGATTTGGTGA

TCGTCGTAAAGCTATGCTTGAAGACATTGCTATCTTGACAGGTGGTACAGTGATTACAGA

GGATCTAGGACTTGAATTAAAAGATGCTACAATGACAGCCCTTGGACAGGCTGCTAAGAT

TACAGTTGATAAAGATAGCACAGTAATTGTTGAAGGTTCAGGAAGTTCAGAAGCTATTGC

TAACCGTATTGCACTGATTAAATCGCAATTAGAGACAACAACTTCTGACTTTGACCGTGA

AAAACTACAAGAACGTTTGGCGAAATTAGCTGGTGGTGTAGCTGTTATCAAAGTAGGAGC

TCCAACAGAGACAGCTTTAAAAGAAATGAAACTTCGCATTGAGGACGCTCTAAATGCTAC

ACGTGCAGCCGTTGAAGAAGGTATCGTTGCTGGTGGTGGAACAGCACTTATTACGGTTAT

TGAAAAAGTAGCAGCTCTTGAACTTGAGGGCGATGATGCTACTGGACGTAACATTGTGCT

TCGTGCTCTAGAAGAGCCTGTACGTCAAATTGCTTTAAATGCTGGGTACGAAGGCTCCGT

AGTTATTGACAAGTTGAAAAACAGCCCTGCAGGAACAGGATTTAATGCTGCAACAGGTGA

GTGGGTTGATATGATTAAAACAGGAATCATTGACCCTGTCAAAGTAACACGATCAGCGCT

TCAAAATGCAGCTTCTGTAGCTAGTCTGATTTTGACAACAGAAGCAGTTGTTGCTAATAA

GCCTGAACCAGCTGCGCCAGCGCCAGCAATGCCAGCAGGTATGGATCCAGGAATGATGGG

CGGCTTCTAAGCTCTCTATAGCATTAAGGATTATGAAAAAGAAGTTGAGACTAAAGGTCT

GCTTCTTACTATGAAAAAAGAACAAAGATTGGATTTCGACTCATGATATCCCATCTTTGT

TTTTTTAGTTGTGATATAAAATGCTAATCTTTGTGGTTACAGACTTTTAATGTATTGGAA

AATATATTGTTTTTCTTTGTCATGTGCTATCACGGAGCCAATGAAAAGTTATTCCCATAT

ATGGGTGTTGGAGGTCTGTGATGAATCATTTTGGAGAGATTTTTAAAACCTTTAGAGAAT

CAAAGGGATCACGATTAAAAGATGTCGCAAAGGCTGGTATATCAACCTCTCAGCTGTCGC

GTTTCGAAAAAGGGGAGACGGACTTAACCATATCGACATTTATGCTGATTTTGGATGAAA

GTAACATGCCCATTGATGAGTTTATGTATGCTGTCCATGATTTTCATCGTGACGACTTAA

ATGAACTCTTATCAAAAAGTGAGGCATTTCGTAACAATTCAAGATAGTGATGGCTTGAAG

CAGTTACTTCATGCTCAACTAGCATCAACTGATAAGAAGGAGATGTTCACGGATATCTTT

TTGGCATATCCAGCATGTCTAAGTCTGCTATGGCATGCCATTTTGGGAGTCCCTCACGCG

ACTCTGTCAGTAGCTCTCCTTCAAAGAAAGATTCACAGGGCTCGACTTTACCTTCTGGCT

GGACCCATCCTGGGAAGTTATCGTGTTGTCTATGGAGTCATAAAATCATATCGTTATGTT

TGACACAAATATTCTCCCAGTCTTTAACCGTTGCTATCACCAGATTCACCACCTTTTAGT

ATAGTATAAGAAAAAAGGGGTAAAAGAGGTTTCTTGTTGCTTTTTTGGCTTAAGGTTTCT

TTAAGTCTTATCTAGTATACTAATGTCATTCCTTAATAGGATAAGTATTTGGTTGTAGAA

GTATCGTCTTAAACTAACGCTATCTCAACCGATTCCCATCTTATTCACCCCTCCTATACC

GATAAGGTCTCACAAAGTGTGAGACCTTATTTTTTGACTTCAGCTCTAGTTATGGAGAAA

CTTGTTGATACAAGCTTTTTTGGGTTATGGTATTATGATCTATTCAAGCCTTTCTTGTTT

TTGGGTTTTTCTTTAACCAACTTGGCTTCAATAGCTTTTAAGCGTTTGAAGGTTTTTTCA

GCGCGATCTAGTGAAATCTTATCAGCTTTTTCTTGAGCAGCTTTGGGGTCACTGACGACT

AAGTCACTGATTTCTTTGGTGCTTTTTTCTTGTTCGGCTATCCAGGTTTTTTCCAAGCCT

TTCATCTCATCAATCACTTTAGTTCCAAATGGTTTTGGATGAGCAGCCACGAGATCATTA

ATGTGTGACACCGTCCAATACCATGACTTATCGTTATATTGGGTGCTTTTTTCTTGATAG

GCTTCATAGGTTCTACTGATATTGCCAAGGTATGGCAAGTAAGGGGCATTACGAGGACTG

CCAATAGATAACCACATGACACCACCACCCAATTCAGCAGGGATGTCTTTTTTCAGTTGG

AAAATATGAGCTTCCATGACATTAGGATTTGAGATGGGATAAGCATAGCCCTTAACAGCT

TTTTTGGATTTTGGTTTACCCTTACCGTCTAATGCCATCTGATCAAGTGGTTTAAGATCC

AGCCCCTCAAAACGATTACGTTGCAGTTTCATTGCATCTTCTAGACTAAAGGTCTTATCA

GTGCTTTGGAGCAACTCATAATTGCTGTCTTTATAAGTTACTTTTGAATCAGGGTCAAGT

GATTTAATCCCTGAGAAAGAGCGAGAGCGGTTGGCATCGTTTAGTGGCGGATTGTAAGAT

TTGGCAATATGAAATTTACCATCAACTTCGGTATAAGATTTGGCTTTTTTAGCGACTTTT

TCCACATCTTCTGAGGCAATCGTATTTTCCTTATCGTTGAAATCGACATGTCCGAGATAG

AAGGTATTTGGAAAAACAGCGTATTTATCATCTGGGAATTTGATGGCAACATATTGGTGA

CCAGAGAGAATTTCCATGTACCAGATGCCGTCTTTGTCAGCCAAGGTAACAATATTTCCT

TCAGCAGCTCCTTTTTCAGTGACTATTTTAGCAATCAATGCGACACCTTCACGGGCTGTT

TTGACACTTGGTAAAATGACACTGGCCATAGATGATTCCGCTAAGCCATTTTTGACGTAA

GGATCAATTTTTTGGATGGCATCATTAGCTGAAGCTGATACTGTTGCAGACATAGAGACG

CCAAACTCATTAAAACCAGCTTCGTCATAAACGCCTTTATTAGGTGTTACATCAGGAATC

GCACTATAGCGATAAGAATGCTCAGGCAATGGGTACTCAAAACCATTAGAGGGGTCTTTC

CATTTTTCGCCAGCAGGATTGTCCTTTGCTAGACGTACAATGAAATTTTTATTATGGTGT

GGCTCAAGATCTTCTGTTCGACCATAAAGAAGAGAACCGTCTTTGGTTAAATCTTTGCCA

ATAATAAAACCAGTACAAGCATAACTAACACTTTGAAGCGAAAAGGCTGTCAGAATACTT

AAAACTCCAAGTGATATTTTTTTGTTTATCATGCGAATCCTCCTAGTAAACGATAACGCT

TTCACAGACATTATAAAATGCTCTTTTTAAAAATTCAAGTCTTTTATGAAGTGTTTTGAA

ATTGTTAGCTATCTAAATATTATGAGAATTCTCTCTTGAATTTTGGCTTAACGGCCCCTA

AAAACGTATAGGCAGTGAACTATCTAGGTTTCTTCGAAAATCCATTAGCCATTTATGATA

TAATAGTAAAGAAAAACATAGAAAGTTGGGGTCTGTTGATGACCAAACCATTTCATCATA

AGAAACTAAAACAAATAACTATAATAGCTGCAACTAGCCTTTTTTTATTCCTGATTGGTG

GTGCCTTTTACTACTCTAAAAATCATTGTATTAATGCCTATCTTAAAGCTCGATCAGCCC

AATCAGGACCAGTTTTTGAGAATATCAAAGCATATCTAGTCTGGGATGATACTAATGAGC

AGATCACAAATGACGAGGCGATGTATACTAAGTTTAGAAGGTATAGTCAAAAAGAATTGA

GGCAAAAAAAGCAGGATTTAAAAGCCGCTAGTCAAGATAGTGCCGTTCAAGTAAAGTCTG

TTGGTCGTCGTTTTTGGATTTTTCCTGATTATCGGATTGCCATAAAACCCATGGATTTAA

CGATAAAAACGAATGTGCCTCAAGCAGACGTTCTTTTAAATCAAAAAAAAGTTGCTGTTT

CTGATTCAGAACAGTTCTCAGTCAAGCTTGATCGGCTACCAACGGCAGAATATACCGCAA

GTATCAGAGGCAAACACAACGGGCGAAACATTAAAGTCAATAAATCATATGATGGTGATA

ATCCCGTGCTAGATTTGAGTGTGTCTTTTAGAACTTTTTTGGTAACAAGCAATGCTAAGC

AAGGAGATCTTTACTTCGATGATAACCATATTGGCACATTAAAAGATGGTCAATTACAAG

TAGAAGATTACCCTGTTACAGAAAACGCACAAGCTTATATGAAAACAACATTCCCAGATG

GTGAGTTAAGATCACAAAAATATGCTCTAGCTGATGTTGAAGAAGGAGCAACCCTGGAGA

TTTTAGTTACAGATCTTTTAGAAGAGGACATAGCAGGGGAGCTATTAGTATCAGCTTTTG

ATCAGCTGATGCACTACCTCAGTACAGGTCAAGACTCATCTAACTTACGTAGCGTCTTTG

AGGCAGGGTCTAGCAACGCATTTTATAGGGGATTGAAAGAGTCCATAAAAGCGAAATTTC

AGACAGATACAAGAAAAGCCAGCCGTCTCAATATTCCATCTATCCTTTTGACAACAATGA

CTCAAGTGGGCAAAACAACTTACGTGCTTGATTTCACAGCTACCTACGAATTTTTGTATG

ACAACTCAACAGATCCTGAGCAGCATACCTCTGGACATATTAATCAAGACTTGACTGGAA

AAGTGACTGTAAAAAAAGTTGGACAGCATTACCTTATCAGCCAGTCCGGCTCTAAAAATA

TTACTGTTGTTAAAGAGGACAATCAACTCAAAGCGCCATCTGTCTTTCCTGAGTCTATTT

TGGGAACGTGGACAGGCCAAGCCAATGGTTTAAGCATCCATATGTCTCTAGCATCAGATG

GAACAATTACGACTAAAGTTGAAGATCAAAAAGGCAACCGTTCTAAAGAAACTCGGACAG

CTAAAATTAGTAAAGTTGAAGACAAAGGCAATGGTTTTTATCTTTATACGCCAGATCCTG

GAAGTGACATAAGCGCCTTAGCTCCAGAAGGAGGATTGGGGGGTGCAAATGTCAAATATG

CTTATGGTTTCAAAATATCTGGTAAAACAGCCTCTCCAGTGGTGTGGCAGGCAGCATTAA

CACATGAATTTGATTATACCAAGCCACTTTCGGGAGTAACTTTGCAAAAGCAACCATAAA

GGATGCAAAGCCACAAAGAACAGTAGAAACAATGTTATTACTCAAATACAGTGCTATGAA

AACAGCTAAACTCAAAAGGCTAGGAGATTATTTCCTAGCCTTTTGAGCGTTTAAGACTGC

GTCAAATGTAGCTGATTTAGCTTGCACGCAAATGTCTTCTTGGGTTAGAGGGTGTTTCAA

ACGTAATTGATAAGCATGGAGCATCAGTCGAGCACAATCTTTTTTTCCATTAGAATACAA

AGGATCACCAAATAAAACATGACCTTGATGGGCTAAATGGACTCGAATTTGATGAGTCCG

TCCAGTTTGTAATTGACAATTGAGTAAGCTTGCCGTTTTGTGGAAGTTTTTGACTAGAGT

TACCTCAGTAATAGCTTTCTTGCCATTTATCGGATCGACAACACGTTTTCTGCGGTCATG

TCGATGACGACCAATTGGATGATGGTAAGTTACCCTAGGACTGTCTAAGGATCCGTGAAC

TAGTGCCAAATATTCTCGATGGATGTCTCGTTTTTCTAAAAGACGATTAAGGATTGGTAA

GATAAAAGGTGTTTTAGCAAATAAAATGGCCCCACTTGTTTCCTTATCCAGACGATGAAC

CACATAGCAGGTCTGTCCCGTGTAAGCAGACACGTGGTTGAGCAGCGCCAACTCTGTTGG

ATCATTGCCGTGTGTTTTCATCCCTTCAGGTTTGTTAACAATAATGATGTGCTCATCTTC

ATAAAGGCAGGTTACTTTTTCGGCCTGCCCCATCACGATAATCTTTTCAGGATAATCTTC

GTGATCGAAAAAAAGTTTGACCTGATCACCGTATTTAACACAACTTTGCCAGTTAACAGG

ATGACCATTAATAAGAACATGTTTTTTAGTTCTCAGAAAATGCCTAATTTTACGGGGAAT

TAACAATTGTTCCTCTAATAATGCTTTGACTGTCAATTCTTCAAAGGGATTTTTCAGTGT

GACAGTAAACCCCTTTGATGGTTTATTTGTTGTCTTCATTGAGCCAGTTATGGTATTTTT

CAACAAGGTTGATAGCCATTTGGGTGGCAAGAGCAGCAGAGAAAGCTTCAGTACCTTCAC

GCTCGTCATTAGCTACCTCCAAGCGAGTCTCATCGTAAATGGCTTTTAGACTTTCTGCGG

ATAAGGTTTCTTTAAAAGCTTGGAATTTATTCATTGGTTAAACCCGTCCTTCTATGTTTA

ATGTGAGCTCCTGATTAATGCAAGGAATTCAATGCTATTATAGCATGTTTCTAATCAAAA

CGTGCCACTCAAAAAGGTAAAAACGTTATTTTGCTAATCCATCTAATAAGAAAGTGTCTT

AAATAAGACCTCTTGATAGAGTTTGTAACAAAAATGATATGAAACTAGGTGCTAACGCAG

AAATAGAAACCAACTAACGTGTTGCGAAGTTGGAACAGTCATAAAAAATAGCAAGATAAA

ACTTATCAACACACAGTTACCTAAGACGATGAGTCAAACAGGTATGTTCTTTCAAAACAA

CCCCATGAGGTTGAACGACGTATCGGAAAAATACGGATTAGTTTGGCCTTATGATAGGTA

TTTCTCTATCGTAAAAGAAGTCAACCAAAGGTTTTTAATATAAAATATTAAGAAAAATTG

TTATAATTATGATAAAAACATAATCGTAAAATAGAGGAAAAACAATGAAAACAATTAGAC

GTTATGATGTTAACGAAGATAGAGGGCATACAGGCCTTGTAGAAGCAGGAGACTTCTATT

ATTTAAACTATTGTGTCGGCAATGTCGGACAAGACATTGAAAGTCAGATTAATGGAGCCT

TTGACGAGATGGAGCGCCGATTAGCATTGGTGGGGCTCACATTAGATGCAGTTGTTCAAA

TGGATTGTCTATTTCGAGACGTTTGGAACATACCTGTGATGGAAAAAATGATTAAAGAGC

GCTTTAATGGCAGATATCCTGCTCGAAAATCGATTCAAACGGAATTTGCACATCACGGTG

GACCACAAGGACTACTCTTTCAAGTGGATGGTGTGGCTTATTCAAAACATATTTCGATGA

CCTAATCAAGGTTACTTTAAGATCATAGTGGTATAATAACGTTATGAGATTTCTAGAACT

TTTACAAAAGAAATTTTTTCCTAAAGCATATCAGGAAAAACAATTCTTAATGCATCAAAA

AACGCGTTTAACGCCACAACACAATCAAAAGCAGTATTCGCCAAATGCCAATCATTTGGA

CTCATCAGCTACCAAAAACTCAGAACAAGACCCTGCAATAGCTCTGCAACGCAGTAGAGC

CTATGAAGGAAGCCCTAAAAGTCGGCCCGCTTGGTTGCAAAAGCTGGAAGCTGTTTTGCC

GTCTCCTCAACATCCAATTCGGCGTTTTTGGCGCCGCTATCACATCGGAAAACTGCTAAT

GATTCTGATTGGAACTCTTGTCTTACTCTTAGGATCATACTTGTTTTACTTATCAAAAAC

AGCTAAAGTATCTGATTTACAAGATGCCTTGAAGGCTACAACTGTTATTTATGATCACAA

AGGAGAGTATGCAGGCAGTTTATCTGGTCAAAAAGGGAGTTATGTTGAGCTCAACGCTAT

TTCAGATGATCTTGAGAATGCTGTTATTGCCACTGAGGATAGGACTTTTTACAGTAATAG

CGGTATTAATCTTAAACGCTTCTTATTGGCAGTAGTTACGGCGGGCCGCTTTGGAGGTGG

CTCAACGATTACACAGCAACTGGCTAAAAATGCTTATCTCTCACAAGATCAGACAATTAA

ACGAAAGGCCAGAGAGTTTTTTTTGGCGTTAGAGTTGACCAAAAAATACAGTAAAAAAGA

TATTCTTACTATGTACCTTAACAACTCCTATTTTGGAAATGGAGTTTGGGGAGTTGAAGA

TGCCAGTCAAAAATATTTTGGAACCACAGCTGCTAACTTAACATTGGATGAAGCTGCCAC

ATTAGCAGGTATGCTCAAAGGACCTGAAATATATAACCCTTACCATTCTCTAAAGAATGC

TACTCACCGTAGAGATACTGTTTTAGGAGCGATGGTTGATGCCAAAAAGATTACCCAAAC

AAAAGCTCAACAAGCTAGAGCAGTAGGGCTAAAAAATCGCTTAGCTGATACTTATGTTGG

TAAGACAGATGACTACAAATACCCATCCTACTTTGATGCTGTTATTAGTGAAGCAATAGC

AACTTATGGTCTTTCAGAAAAAGACATTGTTAATAATGGATACAAAGTTTACACCGAGCT

AGATCAAAATTACCAAACTGGCATGCAGACGACTTTTAACAACGATGAACTATTTCCTGT

TTCAGCTTATGACGGTAGCTCTGCTCAAGCAGCTAGTGTTGCTTTAGATCCTAAAACAGG

AGGTGTTAGAGGTCTGATTGGTCGTGTGAATAGTAGTGAAAATCCGACTTTCAGAAGTTT

TAACTATGCGACTCAAGCAAAACGTAGTCCCGCATCAACAATCAAACCACTCGTGGTTTA

CGCGCCAGCCGTTGCTTCAGGATGGTCAATTGAAAAAGAACTACCAAATACCGTTCAAGA

TTTCGATGGCTATCAGCCACATAATTATGGAAATTATGAATCAGAAGATGTTCCTATGTA

TCAAGCATTAGCAAACTCTTATAATATTCCAGCAGTTTCTACATTGAACGATATCGGAAT

CGATAAAGCCTTTACCTATGGTAAAACATTTGGGTTAGATATGAGCTCTGCCAAAAAAGA

GTTGGGGGTAGCTTTAGGTGGCAGCGTGACAACCAATCCATTGGAGATGGCTCAGGCATA

TGCTGCCTTTGCCAATAATGGAGTAATCCATCCTGCGCACTTGATTAACCGGATTGAAAA

TGCCAGGGGTGAAGTGCTTAAAACCTTTACTGATAAGGCTAAACGTGTTGTCAGCCAGTC

TGTTGCAGATAAGATGACAGCCATGATGCTAGGTACCTTTTCAAATGGAACAGCAGTCAA

TGCTAACGTATATGGCTATACACTAGCTGGTAAAACAGGGACGACAGAAACCAACTTCAA

TCCCGACTTAGCAGGCGATCAGTGGGTTATTGGTTATACGCCAGATGTTGTTATTAGTCA

ATGGGTAGGATTTAATCAGACCGATGAAAATCATTATCTAACGGATTCAAGTGCAGGCAC

GGCTTCAGCTATTTTTAGCACTCAGGCATCTTACATTTTGCCTTATACCAAGGGCAGCCA

ATTTCATGTAGATAATGCCTACGCTCAAAATGGTATTTCAGCTGTTTATGGAGTCAATGA

AACAGGTAATCAATCAGGAGTTGACACTCAATCTATTATTGATGGTTTAAGAAAATCAGC

ACAAGAAGCTTCGCAATCACTATCAAAAGCAGTCGATCAGTCAGGGTTACGTGATAAGGC

CCAATCTATTTGGAAAGAGATTGTTGACTATTTTAGATAGCTTGCAGTATTTTAGAGATC

GTGTTAAAATAATAGTTACGGAGGCGTTATGGCACAGAAAAAAGCGAGTCTAGCGTGTGT

CGAATGTGGCAGTCGTAACTACTCTATCGGAGTGAGTAGCACGCCAAAACCAACACGTCT

AGAAGTAAATAAATTTTGCAAATACTGCAAAACATATACCTTACACAAAGAAACACGTTA

AAAAGGAGAGCCTGATGGGATTCATTAGCGGGACTTTTAAAGTCTTAAAAGACACAACAT

GGCCAAATCGTAAACAACGTTGGAAAGATTTTATCTCAGTTCTTGAGTACACAGCTTTCT

TTACGGTGATTATCTACATCTTTGACCAGCTCTTAGCAAAGAGTGTATTAGCACTGATTA

ATTTATTCTAGGTCAAGATTGGTAGAAATGATAAGCTTGCTTGGAAAAGCCTAGGCTGGG

AGTATTCCCAGCCTTTTTTGTAGTATCAATAATCTGATTAGTAAGCTTTTTTAAAGCCCA

TACTGGTTTGAGCTTTAAAAAAGCATAAAGGTAAAGGCAATGTAGCAACGATCTCCAAAC

CAAGTGCACTAAATTAGCTTGTAACAGAGTCAACGCATAAACAGCCTGGCCTCACTTGCT

TAGCACATTAAAAATAGCAATGAATATTATGGTTGAGGAAAACAGCCTGATAGTCTGCTA

AATCTTCGGGATGATAAGCCTTGACCTCAGCCATCTCATACTCCCTACCTAGAAGTTTGT

ACTTGTTTTCTCCAAATTGATGGAAAGGTAACAGTTGCACTTGGTCAATCTCAAGCTGAT

TAAAGAGCTCTGAGAAGGCTTTAGCATCGTCTAGGGAATCATTAAATTGGGGAATAACAG

GGATGCGCAAGACGATTTCTTTTCCAGCTTGAAAGGCATAATGAATATTTTTGATAATCA

AGTCATTACGAACCCCAGTCACTTTTTGGTGTCTGAGCTGATTATAATGTTTCAAATCGG

TATAGATAAAGTCCACGTAGTCAATAAGGGTGACAAATTGCTCGTGTTTGGCAAAGGCGG

TGGTTTCAATTGCGGTGTGAAGACCTGCTGCTTTGGCTGCTTTTAACAGGGCTAAGGCAA

AGTCAAACTGAGCAAAGATTTCCCCCCCTGACAGGGTCATGCCCCCACCTGACTCTTCGT

AGAAATCAAGGTCTTTAAGGACTTCTTCGATCACCTCGTCCACGGTTTTTTCCTCTCCAA

CTATTTTAGTATTAAGCCCATCACTTGTTAACATCTGCTCAGGCGCTTTTTGTTGGGATT

CGGGATTGGCACACCATGGACATCTCAAGGGGCAACCTTTTAAAAAAACGGTTGTCCGAA

TACCAGGACCGTCATGAATACTAAAATGTTGGATGTTGAAAACAATACCGCGATCAGTCA

TAAGTGTGAATTCCTTTCGTTGTAAAACCATCATACCAGATAAACGTAAATAAATCAATT

ACGAATGAAAGTAAATATCCCTTTTCTTTTAAGAAAATAACTGGTACAATGAAAGGGAGG

TGAATGATGAATCGATTAGAACGTATTATTCAACTGGTTTCCCAGAAGAAAAAAATAGAT

GTTAACAGTTTATCAGAGCAATTAGACGTTTCGAAAGTAACCATTCGAAAAGACCTTGAT

AAGCTTGAAAGTAAGGGCTTGTTGCGACGGGAACATGGCTATGCGGTGCTAAACAGTGGC

GATGACCTCAATGTACGCTTGTCCTACAATTACAACATTAAGCGACGGATTGCAGAAAAA

GCAGCTGAATTGGTGCAAGACAACGATACCATTATGATTGAATCTGGCTCAACCTGTGCC

CTTTTGGCAGAAGTACTTTGCCAGACCAAACGAAATATTAAAATCATTACCAATTCGTGC

TTCATTGCCAACTACATTCGTCAATATAGTAGCTGTCAAATCATTTTACTGGGCGGTTAT

TACCAGCCCAATTCAGAGGTGACTGTTGGTCCTTTATTAAAAGAAATGATATCCCTTTTT

CATGTCAATCGTGTCTTTGTTGGAACAGACGGTTTCAACAAAGATCTTGGCTTTATGGGC

AAGGACATGATGCGCTCAGAAGGGGTGCGTTACATGGCAGATGCTGCTGAGGAAGTGGTG

ATTCTGACCGATTCCAGCAAGTTTTCAAAAACCAGTCTGGTACACCAGTTGTCATTAGCT

GATGTCAATCGCGTCATTACAGACCAAGCACTAGACAAGCAAACACAAGAGTTATTAAGT

GCTAGTGGTCTTGTTCTTGATTTTGTTTCTTAGAAAATAGGTGGCTAGATGAAAGAAGAA

CGCAGACGGCTCCTTGCCAAGGTGGCTTACTTGCATTATGTTCAAGGCAAGAGCCAAACC

TTGATTTCAAAGGAAATGAATATCTACCGAACAACGGTTTGTCGGATGTTAGCAAAAGCC

AAGGAAGAAGGCATTGTTCGGATTGAAATTGCCGACTATGATGCTGATTTATTTGCTTTA

GAAGAATACGTGCGAAAGCAGTATGGCTTGGAAAAACTAGACCTTGTGCCCAACCAGGTT

GAAGATACCCCAATGGATACCTTGACAAATGTTGCTAAAACAGCAGCTGAGGTTTTTAGA

CATGTTGTTAAAGATGGCGATAAGATCGGTCTGTCCTGGGGAGCGACTCTTTCTTGCTTG

ATGGATGAGTTAAATCCAAAAGCGATGAAAGATGTGTTTATTTATCCTTTGGCAGGAGGA

CCTAGCCATATTAATGCCAAATACCATGTCAATACCCTGGTTTACCGCTTGGCTCGCATC

TTTCATGGTAATAGCGCTTTTATGAACGCTATGGTCATTCAAGAAGACAAACACTTGGCT

AAGGGAATCTTACAATCTAAATACTTTAACGACATTTTAACAAGCTGGGACCAGCTAGAT

CTTGCCTTAGTAGGAATTGGTGGGGAGCCTAATAGCCTTGAGCAGAGCCAATGGCGTGAT

TTATTAACCAGCTCAGATCATGACCAACTCAAATATGAAAAAGCCGTTGGTGAAGTTTGT

TGTCGTTTCTTTGATCAGGCAGGTCAGCCAGTTTACACTGGCCTACAGGATCGAACTATT

GGTATTTCGCTAGAACAGTTGAGGCGTGTCCCCAAAACAATGGCTGTTGCAACAGGTAAA

CACAAGGCTAAGGCAATATTGGCAGCCTTAAAAGCAGGTTTTATCAATTATCTAGTGACG

GATAAAGAGACCATGCTAGCTGTTTTAGCGTTAGATGAAGATATTGATCTTAATAACGTT

CTTCTTTAATAGAAATTCAAAGATACTATGATATTTATAAAGGCCCAAGACCAAACGTCA

GGGCTTTTTTTGTTTGATGTGTTGATGAAACAGAGATTGCTCAAATGTGCAGTTAATATA

TTGGCACAAAAGTGCATTAACCAAAATATAGCACAAAATATATTGACCCTAAAAACAAAA

ATGATACAATAAACTTACCAACGAAATGTAAACGATTACAAACACAATAACATCACCTTC

GAAAAAAAGACTCATTGCCGTTACATCACATAAAAAAGAAAAAAAGGAGACACCAATGCA

AGTTATTGTACCAGACCAAATCATTATGGGCTTAATTTTAAATGCAGGCGATGCCAAACA

ACATATCTACCAAGCTCTGAAATGTGCTAAAGAGGACGATTATGCCACTAGTGAAAAAGA

GATGGCCTTAGCAGATGATGCCTTATTAGAAGCCCACAATTTACAGACCCAGTTTCTTGC

CCAAGAAGCTAGTGGTAATAAGTCAGAGATTACAGCTCTTTTTGTTCACTCACAAGATCA

CTTGATGACGACCATTACAGAAATTAATCTCATCAAGGAGATTATTGACCTTCGTAAAGA

ATTGGCAACCAAATAATATTTCATTCATAAAAAGGAGATTGACATGATTAAGATTGGATT

GTTTTGCGCAGCAGGGTTCTCAACAGGCATGTTGGTGAATAATATGAAAGTAGCTGCTGA

AAAGAAAGGCATTGACTGCCAGATTGATGCTTATTCCCAAGGAAAGTTGGCTGAGTATGC

TCCATTGATTGATGTGGCACTTTTAGGCCCACAGGTTGCTTACACTCTGGATAAATCAGA

AACTATCTGTAAAGAGAATGGCATCCCGATTGCAGTTATCCCAATGGCTGATTATGGAAT

GTTAGACGGTAACAAGGTGCTTGATTTAGCCCTTAGCCTTGTTAAAGAATAGGAGTCAAT

CATGGCAAAAATGAATATGCAAAACATCATCATGCCAATCATGAAATTTGTGAATATGCG

CGGCATCATTGCCTTAAAAGATGGTATGTTAGCCATTTTACCTTTGACTGTTGTGGGGAG

TCTTTTCCTGATTGCTGGGCAGATTCCTTTCCAAGGGGTTAATGATGCTATTGCTAGTGT

GTTTGGTGCTGACTGGACAGAACCCTTTATGCAGGTTTACCATGGAACATTTGCTATTAT

GGGGTTGATTTCCTGTTTTGCAATTGGCTATTCTTATGCTAAAAACTCAGGTGTTGAACC

TCTCCCTTCAGGGGTTCTATCCTTGTCAGCCTTCTTTATTTTATTGAGATCATCTTATGT

TCCAGCAGAAGGCGAACCTATCGGTGATGCTATTAGCAAGGCTTGGTTTGGTGGGCAAGG

GATTATTGGTGCTATTGTGATTGGTTTAACGGTAGGTGCTATTTATACAGCATTTATCCG

TCGCCACATTGTCATCAAAATGCCAGAGCAAGTGCCACAAGCCATTGCCAAACAGTTTGA

AGCTATGATTCCAGCCTTTGTGATTTTTACCTTGTCAATGCTTGTGTACATTATTGCCAA

GTCAGTGACAGGTGGTGGCACCTTTATTGAAATGATTTATGATGTCATTCAGGTACCGCT

GCAAGGGTTAACGGGTTCTCTTTATGGCGCACTAGGTATTGCCTTCTTTATTTCCTTTCT

TTGGTGGTTTGGCGTGCATGGGCAATCTGTGGTCAATGGAATTGTCACTGCTCTTCTCTT

ATCAAATTTAGATGCCAATAAGGCCTTGATGGCAGCAGGTGAGTTATCCCTAGACAAGGG

TGCCCATATTGTAACCCAACAATTTTTAGACTCTTTCTTGATTCTATCAGGTTCTGGCAT

TACCTTTGGTTTAGTGGTAGCTATGATCTTTGCGGCTAAATCCAAACAATACAAGGCTTT

AGGTAAGGTTGCAGCCTTCCCAGCCCTCTTTAATGTTAATGAACCTGTCGTCTTTGGTTT

TCCAATTGTGATGAATCCAGTCATGTTCTTGCCATTTATCTTGGTTCCTGTATTGGCAGC

TCTCACAGTTTATGGCGCCATTGCTATTGGTTTCATGCAGCCCTTTGCAGGAGTGACCCT

TCCGTGGTCAACACCAGCCATTATCTCAGGGTTCATGGTTGGTGGCTGGCAAGGAGCTAT

TGTGCAAATTCTTATACTCATCATGTCAACGTTGGTGTACTTCCCATTCTTTAAAATTCA

AGATAATATGGCTTATCAAAATGAACAAGCTAGTGAAGAGTCATGATTTAGCGTCCACAC

AAATTCTTGAGACCATCTCCAGTTAGAGGTGGTTTTTTAGAAAAAGAAGAGCGACTTATC

AGGCCTATATTGCACAATAGTGCAAAATAAAGTGACTATCTCTTCTTATGGTTAAGGATA

CGCAAAAGTCGCAATATATGATAAAATAGTTACGAAAGAAACATTTATAATTTCGTAAGA

AAGAAGGAGACCCCGCATGACTGAAACAAAAAGCCCTTATTTTGGACATTTAACAGACAG

AATGACTCACTATCGTGAAGCTGTGTTAGACAAGAAACCTTATATTGATGCTGAGCGTGC

TATTTTAGCCACTGAAGCCTATCAAAAACACCAAAATAAGCCAGCAAACCTCAAACGCGC

TTACATGCTTCAAACTATTTTGGAAAACATGACCATCTATATTGAGGATGAAAGCCTCAT

TGCTGGTAACCAAGCCTCTTCAAACAAGGATGCACCGATTTTCCCAGAATACACTCTGGA

ATTTGTCCTCAACGAACTTGATCTTTTTGAAAAACGAGATGGGGATGTCTTCTACATTAC

CGAAGAAACCAAGCAACAGCTCCGAGACATTGCCCCATTCTGGGAAAACAATAACTTACG

TGCTCGCTGCGGGGTCTTGCTACCAGAAGAAGTGCAAGTGTACATGGAAACAGGTTTCTT

TGGCATGGAAGGCAAGATGAACTCTGGGGATGCCCATTTAGCGGTTAATTACCAAAAACT

CTTGGAGCACGGCTTAAAGGGATTTGAAGAACGGGCACGAGCTGCCAAAGCTGCGCTTGA

TTTGACCATTCCTGAAAACATTGACAAATACCATTTTTACGACTCTGTTTTTATTGTCAT

TGATGCAGTCAAAACTTATGCCAAACGCTATGCCAAGTTGGCTAGAGAACTAGCAAAAAC

GGCTAAACCAGAACGTCAAGCAGAATTGTTAGACATCGCGCGTATTTGTGACAAAGTGCC

TTACGAACCAGCGAAAACCTTTGCAGAAGCTGTGCAATCTGTCTGGTTCATTCAGTGTAT

CTTGCAAATTGAATCAAACGGGCATTCCCTATCTTATGGCCGCTTTGACCAGTACATGTA

TCCTTATGTCAAAGCTGATTTGGAGGCTGGCCGTGAAACAGAAGACTCAATCGTTGAACG

TTTGACTAACCTCTGGATTAAGACCTTGACCATTAACAAAGTGCGGAGCCAAGCCCACAC

CTTCTCATCAGCAGGTAGTCCACTTTACCAAAACGTGACGATCGGTGGTCAAACACGCGA

TAAAAAAGATGCGGTCAACCCACTGTCTTACCTTGTCCTACGGAGTGTGGCTCAAACCAA

ATTGCCACAACCTAACTTGACTGTTCGTTACCACAAAGGCCTAGACAATACCTTTATGAA

CGAGTGTATCGAGGTCATGAAACTTGGTTTTGGCATGCCAGCCATGAACAACGATGAAAT

CATCATTCCATCTTTCATCAAAAAAGATGTCTCAGAAGAAGATGCTTATGATTACTCAGC

CATTGGCTGTGTGGAAACAGCGGTTCCAGGCAAATGGGGCTACCGTTGCACGGGGATGAG

TTACATCAACTTCCCTAAAATCTTGCTGATTACCATGAATGACGGGATTGACCCAGCATC

AGGCAAGCGCTTTGCAAAAGGATACGGTCACTTCAAAGACATGACCTCTTATGAAGAATT

AAAAGCAGCATGGGATGCCACCTTGCGCGAAATTACCCGCATGAGCGTCATTGTTGAAAA

TGCCATTGACCTAGGTCTTGAACGTGAAGTGCCAGATATTCTTTGCTCAGCCTTGACAGA

TGATTGTATCGGTCGTGGCAAGACCTTAAAAGAAGGCGGAGCTGTTTACGACTATATCTC

AGGCCTTCAAGTTGGAATTGCCAACCTATCAGACTCCCTAGCAGCGCTGAAAAAATTAGT

GTTTGAAGAAGGCCGCTTGACCCCAGAAGAACTCTGGCAGTCCCTTGAAAGTGACTTTGC

TGGAGAACGCGGAGAAGAAATCCGCCAAATGCTCATCAATGACGCACCAAAATATGGTAA

CGATGATGACTACGCAGATAGTTTAGTTGTCGAAGCTTACGACACCTACATTGATGAAAT

TGCCAAATACCCAAATACCCGGTATGGCCGTGGCCCAATCGGAGGTATCCGCTATTCTGG

GACATCATCAATCTCAGCCAATGTCGGACAAGGAAAAGGAACCTTAGCGACACCAGACGG

TCGCCATGCAGGGACACCCCTAGCAGAAGGCTGTTCACCAGAACACAGCATGGACAAAAA

AGGACCTACTTCAGTATTGAAATCTGTTGCTAAATTACCAACAGATGAGATTGTCGGAGG

AGTACTACTCAACCAAAAAGTTAACCCACAAACCCTGGCCAAAGAAGAAGACAAACTGAA

ATTAATGGCCTTACTTCGTACCTTCTTTAATCGCCTACACGGTTATCATATCCAGTACAA

TGTCGTGTCACGTGAAACCTTGATTGATGCCCAAAAACATCCTGAAAAGCACCGCGACCT

CATTGTCCGTGTTGCCGGCTACTCAGCTTTCTTCAATGTGCTCTCAAAAGCAACCCAAGA

TGACATTATTGAACGTACAGAACACACACTTTAGGAGAAAATAAGTATGGAATACATGTT

AGACACTTTAGATTTAGAAGCTATTAAAAAATGGCATCACATTTTGCCTCTCGCAGGTGT

GACCTCAAACCCTTCTATTGCCAAAAAAGAAGGTGACATTGATTTCTTTGAACGGATTCG

AGAAGTCCGTGCTATTATTGGTGACAAGGCTTCTATTCATGTTCAAGTGATCGCCCAAGA

TTACGAAGGCATCTTAAAAGATGCTGCTGAAATCCGCAGGCAATGTGGAGATAGTGTGTA

TGTCAAAGTACCTGTAACCACAGAAGGCCTTGCAGCCATTAAAACCTTGAAAGCCGAAGG

CTATCATATCACAGCAACCGCTATTTATACGACCTTCCAAGGCCTATTAGCCATTGAAGC

TGGTGCTGATTATTTGGCTCCTTACTATAACCGTATGGAAAATCTCAATATTGATCCAGA

AGCTGTCATAGGACAATTAGCAGAAGCTATTAACCGCGAGAATGCTGATAGTAAGATTTT

GGCAGCAAGCTTTAAAAATGTGGCTCAGGTTAATAAATCCTTTGCTCTAGGAGCACAAGC

CATCACTGCTGGCCCAGATGTGTTTGAAGCAGGATTTGCCATGCCATCTATTCAAAAAGC

GGTTGACGATTTTGGCAAGGATTGGGAAGCCATTCATCACCGCAAGAGCATTTAAGAAAT

AGCATCAGTAGTAATCACATCATTAGGAATCACAGATAAAGGAGTTCAACGATGAAAGTA

TTTGCAAGTCCATCTCGTTACATTCAAGGTAAAAATGCCTTGTTTACCAATGCTAAAACC

CTAAAACAACTAGGGGATAGCCCCATTTTACTGTGTGATGACGTGGTGTATGGCATCGTC

GGAGAAAGATTTGAAAGCTATTTGATAGATAATGGCATGACTCCTGTTCACGTAGCCTTT

AATGGCGAAGCCTCAGATAATGAAATCAGTCGCGTGGTTGCCATTGCCAAGGAAAATGGC

AATGATGTTATTATTGGACTTGGTGGTGGAAAAACCATTGACAGTGCTAAGGCTATCGCT

GACTTACTTGCTGTTCCGGTGATTATTGCCCCAACCATTGCTTCAACAGATGCCCCAACC

TCAGCCTTATCGGTTATTTACACCGATGAAGGAGCCTTTGAAAAATACATTTTCTATTCA

AAAAATCCAGATCTTGTTTTGGTTGACACACAAGTGATCTGTCAGGCACCAAAACGATTA

CTGGCCTCTGGGATTGCAGATGGATTAGCAACATGGGTAGAGGCGCGTGCTGTTATGCAA

AAAAATGGAGACACCATGGCAGGTGGCAATCAAACCTTGGCAGGAGTTGCCATTGCCAAA

GCCTGTGAGCAGACCTTGTTTGCAGATGGCCTAAAAGCGATGGCTAGCTGTGACAGACAA

GTCGTGACCCCAGCCTTGGAAAATGTCATTGAAGCCAATACACTCCTTAGCGGGCTTGGT

TTTGAAAGTGCGGGTCTGGCTGCTGCACATGCCATTCACAATGGCTTTACAGCGCTGACA

GGTGCTATTCATCACCTTACCCATGGTGAAAAAGTCGCCTATGGCACGTTGACCCAACTC

TTCTTGGAAAATCGTTCGCGTGAAGAAATTGACCGCTACATCGACTTTTACCAAGCTATT

GGCATGCCAACAACCCTCAAAGAAATGCATTTAGACACGGCTACTCAAGAGGATTTCTTA

AAAATTGGCCGTCAAGCAACCATGGCAGGCGAAACCATCCATCAAATGCCATTTGTTATC

AGTCCAGAAGATGTAGCAGCGGCTTTGGTTGCTGTTGATGCCTATGTTACGAGTCGATAG

GCTAACCAAAAAGAGATTTCTTATTTGCCAGATAACCTCAAAAAAACAAGTCTAGGGAGT

CACTTCCTAGACTTGTTTTGCTTACTGTTGGTTGGTAATAGCACTTTTAGCGGGGTCATT

GTGGGCAATACGACTGGCAGCTGCAGCAGCAATGGCGCTAACAATATCATCTAAAAAGGT

ATGGCAGGAGTTACCATCTTTATGGTTGAGTTTGTCAACAATTCCTGGTTTTGTCTTGTC

CAGATACCCATAATTGGTAAAACCGATAGACCCATAGAGGTTAACAATGGATAAGGCCAG

GATTTCATCAATCCCATACAGCCCTTGGTCGGTTTTCAAGATACTAAGGAGTGGCTCAGA

CAGCTGGTTGGCTTCAGCCAATTTATCCAATTCTACCCCAGTGATAATAGCGTTTTGCAC

TTCTCGTTTTGCAAGCACCGCCTCAACGCTCTCTAAACATTCTGCCATGGTCAAATTGGG

AATATAATCATTCTGCAAAAAAAGCACCAGCTCTGCAATAGCTTCTAAAGAACCCCCTCG

CTCAGCCAGTAATTGATAAGACACTTCTCGTAGGTGATGGTCAGTTGACATGTGAGACCT

CCTTTTTTTCTTTATTGTAACATAAAAGATTTCGAGAAACCTCTACAACAGCTAAAGAGG

GGATAGTTCTTTTTTAAGAGATGTTGACTACTGAGATCCCCTACTTGGTAAAAGTCAAAG

AAACGATAGTAAAGTAACCATCTTACCTAGGCCTAAATACAATCACCACCCCTAGGTGAC

ACACAGCACCTATTTCAAAAATGATATTAGTATGGCAAAACAAAAAGACAACGCCTTCTT

TTTTCTCCTTACTATCTCCTTTAATTTTCATATTTTTTAAAAAAACTATTGATAAACTAG

TTAAGTAAGCGTATACTATGGTTAGTGAGCGAAATTAGAAAAGAGGACAAGCATATGAAT

CTACTTGGATCAAGACGGGTTTTTTCTAAAAAATGTCGGCTAGTAAAATTTTCAATGGTA

GCTCTTGTATCAGCCACAATGGCTGTAACAACAGTCACACTTGAAAATACTGCACTGGCA

CGACAAACACAGGTCTCAAATGATGTTGTTCTAAATGATGGCGCAAGCAAGTACCTAAAC

GAAGCATTAGCTTGGACATTCAATGACAGTCCCAACTATTACAAAACCTTAGGTACTAGT

CAGATCACTCCAGCACTCTTTCCTAAAGCAGGAGATATTCTCTATAGCAAATTAGATGAG

TTAGGAAGGACGCGTACTGCTAGAGGTACATTGACTTATGCCAATGTTGAAGGTAGCTAC

GGTGTTAGACAATCTTTCGGTAAAAATCAAAACCCCGCAGGCTGGACTGGAAACCCTAAT

CATGTCAAATATAAAATTGAATGGTTAAATGGTCTGTCTTATGTCGGAGATTTCTGGAAT

AGAAGTCATCTCATTGCAGATAGTCTCGGTGGAGATGCACTCAGAGTCAATGCCGTTACA

GGAACACGTACCCAAAATGTAGGAGGTCGTGACCAAAAAGGCGGCATGCGCTATACCGAA

CAAAGAGCTCAAGAATGGTTAGAAGCAAATCGTGATGGCTATCTTTATTATGAAGCCGCT

CCAATCTACAACGCAGACGAGTTGATTCCAAGAGCTGTCGTGGTATCAATGCAATCTTCT

GATAATACCATCAACGAGAAAGTATTAGTTTACAACACAGCTAATGGCTACACCATTAAC

TACCATAACGGTACACCTACTCAGAAATAATACCAAAAGGCTAGACCTCTGCTCACTAGG

CCTAGCTTTTTACATCAAAAAAAGCAATGACTATAGAAAGTAAAAATACTAGAAAAAGCA

ATGATTGCCGTCATTGCTTTTTATGATTTGTCAAAAAGTAAAAAGCAAATTGAAAAAATC

GCCCTGGAGCTGTTGAGATAAACTACACCATTTATTAAGATACCAAATCAAAAAGCTAAC

ACCATAAGAGCGTTAGCTTTTTAAAGAGCCTCAGGACAGTTTATGTTTAATGGCTTCTAG

GTAGGTTTGAAACATATGATGGATCGTTTTGCAATTAAGTAATTGGAAGATCTCCAAGAT

CTGCACACACTTTTGCTCAGTCCCCTCTTTACCATTTATGAGGCCAATAATGCCATCAAA

AAAAGACACACAACACCGTTCATATAAGCAATCATCATTTAGCTGATAATCTTCAATTTT

TGCCAAAATCTCAGAAGCTTTATCATACTCTTGTCGCTGAATAAACAAAATCAACATATT

GACAAACATCCGAATCGATTCATTCCCATAATACCTTAGGGTATTGTATTTATCGAGGTT

CAAAATAACTTTTGAAAACACCATCTCAATAAAGCAAGACTCAAAAATAAACATACAATT

ATTAAAAAGCACAGTCTCATAGTGACTCCAAGTTTCAATATTAATAAGATAGTTTGACAA

ATAAGTCCGCTCTGTCAGACAGTCTTCCTCAGTAAGAGTTGCTAATAACACCTTGACCAA

GGCAAAAAGGTTCTTTTCCTTTGTTGACTTACTATCTTTATAAGAATCAAGGATATGATT

TAAACCTTCTATGTTTCGGCATTCAAAATAATGCTTTGCCGTATCCATATCAATAAATTC

TTTGTATTGTTTAAAGTTATTACTAATAAAGAGAAACTCATCAACATTGACATTCAAGTT

GTCTAAGATATAAAGTAACTTTTTCGAAGAAATGTCAAGATTATTTTTAATAAAACGATA

ATAGGTTTGCCTAGTGAGATAATCACCACAAACTTGTTTAATCGAAATGTTTTTTGAATG

CCTAATGAATTCAACGGTTTCACCAATTTCCATATGTCAAGCCTTCCTAGTTGATGTCAA

AAATACGTTACGCATGTACCTAATACGTAACAAGTTGAATGTTTCGGATGATAGTCGCTT

ATGATAGGTGCATAAGGTCAATAGCCAGATGCGATAGTTTTATCAACTGTCATATGTTAA

ACCTTTCTAATCTAATAGATGTTAAAAATACGTTACGTGTGTACCTAATACGTAACAAAA

TAATGGGTTAGCAAAATAAGCAGCTATGATATAGCCATAAGGTTAAAAGGAGGCGCCTAC

TATGTGGTTATTGTTACTATTTTTGTAGTTCCTTTTGCAAAATGGTAGTAGATAAAAATG

AGAAGTCAATAAAAGCATTGACAAACAAAAGAATCATTTTGTTTATATTATCAGTATTTT

TAAGTTTCTTGTCAACTGAAATGAGCATCTACTAGCCACAATAGTAACTAAAAGGCTAAT

TGTCAGTAAAAATGAGATAAATCAGCCAGGAAAAAGCCTACGAGTCAACTAATGGACAAC

CCAAAAAAAGACAAAGGACTTTCACTTGGTGTTGTGGACCCTATCACGATCCTTATCACA

GAATCAGTTATTAACAAAATGAGAACGGTCCCATATCATCTAAAAAAACTGTCAAGCACA

ACCTATTGTTTGCTAGCTGATCTTGCCTATCAGTCTGCCTAGTAGATACGGCAGAAGATA

AGAGATCAACCAAAAGATGATAAGTGCAACGACTCATAGCGTCTTTTGACCAAAACCAAT

TAGGCTCACATGAGATGAGAATAATTGGGTTGGGTTGTCAGTGTCAACTAACCGTGTTAT

TGTCTATTACCATTCATGGTATCAGCGACATCGTATGATAACCATACGATTCAGCTAAGT

AAGGAGGTGTGTCCAATGTACCGTTAAAAGCAAATGCAGTAGATTAACTTATTTTGAAAG

AGGTATAAAAAAAATGAATAAAAAGAAATTAGGTATCAGATTATTAAGTCTTTTAGCATT

AGGTGGATTTGTTCTTGCTAACCCAGTATTTGCCGATCAAAACTTTGCTCGTAACGAAAA

AGAAGCAAAAGATAGCGCTATCACATTTATCCAAAAATCAGCAGCTATCAAAGCAGGTGC

ACGAAGCGCAGAAGATATTAAGCTTGACAAAGTTAACTTAGGTGGAGAACTTTCTGGCTC

TAATATGTATGTTTACAATATTTCTACTGGAGGATTTGTTATCGTTTCAGGAGATAAACG

TTCTCCAGAAATTCTAGGATACTCTACCAGCGGATCATTTGACGCTAACGGTAAAGAAAA

CATTGCTTCCTTCATGGAAAGTTATGTCGAACAAATCAAAGAAAACAAAAAATTAGACAC

TACTTATGCTGGTACCGCTGAGATTAAACAACCAGTTGTTAAATCTCTCCTTGATTCAAA

AGGCATTCATTACAATCAAGGTAACCCTTACAACCTATTGACACCTGTTATTGAAAAAGT

AAAACCAGGTGAACAATCTTTTGTAGGTCAACATGCAGCTACAGGATGTGTTGCTACTGC

AACTGCTCAAATTATGAAATATCATAATTACCCTAACAAAGGGTTGAAAGACTACACTTA

CACACTAAGCTCAAATAACCCATATTTCAACCATCCTAAGAACTTGTTTGCAGCTATCTC

TACTAGACAATACAACTGGAACAACATCCTACCTACTTATAGCGGAAGAGAATCTAACGT

TCAAAAAATGGCGATTTCAGAATTGATGGCTGATGTTGGTATTTCAGTAGACATGGATTA

TGGTCCATCTAGTGGTTCTGCAGGTAGCTCTCGTGTTCAAAGAGCCTTGAAAGAAAACTT

TGGCTACAACCAATCTGTTCACCAAATTAACCGTAGCGACTTTAGCAAACAAGATTGGGA

AGCACAAATTGACAAAGAATTATCTCAAAACCAACCAGTATACTACCAAGGTGTCGGTAA

AGTAGGCGGACATGCCTTTGTTATCGATGGTGCTGACGGACGTAACTTCTACCATGTTAA

CTGGGGTTGGGGTGGAGTCTCTGACGGCTTCTTCCGTCTTGACGCACTAAACCCTTCAGC

TCTTGGTACTGGTGGCGGCGCAGGCGGCTTCAACGGTTACCAAAGTGCTGTTGTAGGCAT

CAAACCTTAGTATGGAAATGCATTTCGTTAGAACAGAACCTGAGGCACGCCGCATAGCTG

AAACCTTTTGTGCCGAAAACACACAAACAAAAACCCCTATGCGCGTGCAGCAGTTAAGCT

ATCCATCAGACACAGATCACTCAGGTGGTGAGCTATACATCTATGCTTTGTCTCCTGCTG

GATTTATCATCGTATCAGGAGATACCAGAGCGCACACCATTTTAGGCTATTCTTTTGATA

ATAACCTGGACCTCAACCATGATAATGTCAGAAGTATGGTAGAAGCTTACCAAAAACAAA

TCAACTCTTTGGATTGATCGGTCAAAGTAGCTTTTGCCCCCTCACCACTCGTTTAAGACG

ACCGTGTTTCTAAAATACCACTACCTTTTAAGTTTCACGCCGGCCTAAGCAAGTGTTTGT

CTCATGGAGCAGACTGTTTCCATCGAAAAGTCACCCATTGATAAAGGAGGTCATCAACGC

CTACTAATGATGACTTCCCTAAGATGCCAAGGTAAAGGACTGAGTTCTGTAGAGATGGGA

CTCAGTCCTTTTACGATTTTTGGGAGTTATTCTAGCCATACCGTGGCAAGAAAACAACAC

TCAAAAATAGCCAACGTCAGTTTCTGTTCATACATGACTACAGCCAAACACCACAACCTA

ACCTATCCTAAACACAGTGGCTTTCTAACGAACAGTCACAACGAGCAGCAACACCAAAAA

GCAAGATTAAGCTACGTTTAAGACTAATGATATAAGATAACAGTAATTATTAATGAGAAA

AGAGGCTCAAAAGGACATGAAACAAATGAATAAACTCATTACAGGAGTGGTAACGCTGGC

GACAGTCGTGACCTTATCAGCTTGTCAATCATCACACAACAACACCAAACTCGTCTCGAT

GAAAGGAGACACCATCACTGTCAGTGACTTCTACAATGAGACCAAAAACACAGAACTCGC

ACAAAAAGCCATGTTAAGCTTGGTGATTAGCCGCGTTTTTGAGACACAATATGCCAACAA

AGTCTCTGACAAAGAGGTTGAAAAAGCCTATAAACAAACCGCAGACCAATACGGTACATC

CTTTAAGACAGTCCTAGCACAATCAGGCTTAACGCCAGAAACCTATAAAAAACAAATTCG

CCTCACAAAATTAGTCGAATATGCCGTCAAAGAACAAGCCAAAAACGAAACCATCTCAAA

AAAAGACTACCGTCAGGCCTATGACGCTTATACCCCAACCATGACCGCAGAAATCATGCA

GTTTGAAAAAGAAGAGGATGCCAAAGCAGCGCTTGAAGCCGTCAAAGCTGAAGGGGCAGA

CTTTGCAGCTATTGCCAAAGAAAAAACCATTGCAGCCGATAAAAAAACAACCTATACGTT

TGACTCAGGCGAAACAACCCTACCAGCAGAAGTAGTTAGAGCTGCATCAGGCCTCAAAGA

AGGGAACAGATCAGAAATCATCACAGCGCTTGATCCAGCCATCTCAAAACGCACCTACCA

TATCATCAAAGTCACCAAAAAAGCAACTAAAAAAGCAGACTGGAAAGCGTACCAAAAACG

CTTGAAAGACATCATTGTGACTGGCAAATTAAAAGACCCTGACTTCCAAAACAAAGTCAT

CGCTAAAGCTCTTGATAAAGCAAATGTCAAAATCAAAGACAAAGCATTTGCCAATATCTT

AGCCCAGTTTGCAAAACCTAACCAAAAACAACCTGCCCAAAAATAGAGGGTTGCCAGTTT

ATTTCCCTAAGGTTGCTTAAAAAGACAGCCCTAGCCATCACTCCCCTTTTCAAAACACCG

ATAATGGAAACGTGTCCTATACTGATGGTCTAGGGCAATGTCATAAAGCTAAAGACTCTA

TCCAAATAAGGATAGAGTCTTTAATATTTAAAAACCAGTCAGCAAAAAAATTCACGCTAA

GCCTAGAATGAGGCTTAGCGTGAATGGTATCAGTGATAGTCCGTTAACATAGCTGATTGG

GAGTCAGAAACTCTAACTACCCAATCTGACTATACCCCCAGTACCAGAGGTGTCTCATCG

CAGAAGTAGCAATCAGATAATCGTCTGATAAGCTCTTGTCCTCAAGCAACTGATGAGTGT

CGTCGCACGGGCTATGAGCTGCCGGTGGCTGATAGCTAGTAGGCTCATCAGCCAAAACTT

TTTTGGAGTAACGCTTTGGTCTCAGACGGCGCCAGAGGGTTGAGAGAGGGGTGAGAAAAA

AACGATGGGTCATCTTGGCCTCCTTGTCTAATGATAGTCACGTTAACAGACAAAAGTGTC

AATAATGTCATTTTAAGGAGAAAAATATTAAAAATCAAGGTCACAAACTTTCCTTTGTTA

AAAAAACTCGACCCTAACAAAGAACCTAAATACCCCTCGCACTTGAGCTTCTCCTTGCGC

AGCTATCTTTTGTACTCAGTTGCATACAAGCTAATGATAGCGATACTATTATTGTCCTAT

AGGCTATCGCGACAACCCCTTTTTTTCGCCTCAATGAGGCTGAAACAGCTGTCATGACAG

AGAAAAGACCCTTGTCTATCTAAGAGACCATTAATTTTTATCTCACCAAAAAACTGATTT

TAGAAACAAAAAATCATGGTGTATAATAAAGTTCGGAACAATTATGACATTATAATGAAA

GTAAGGTTAACGAAACATTGACAAATTGTAAGTATAAACTTAGAAAGTTATCTGTAGGGC

TCGTCTCCGTCGGAACGATGCTGATAGCCCCGACAGTTTTAGGACAGGAGGTTAGTACTA

GTGCTAGTAGTACTAAGACGAGTGCTAGTACTAATATTAATACTAATACTAGTACCGCTA

GCGCTGGTACCGGTACGAGTGGGACGGCTTCCACTACTCCTAGTGTTGGTACAAGTACTG

GTGGAGCAGCTGGGGGTGAAGCAGCTGTAGCATCTAGTGGAGGAAGTCAGAGTTCAGAAT

CTGCCCAAGCCTCAACACAACCTCAAGCACAGACAGCTGTAGCAGCATCTGCTTCCACTA

CTGCGTCCCCTTCTAGTAGTGAAGAGAAAACTCCTAAGACAGTAACTTCATCTACCTCAT

CGACTCCAGCAGCCAGTAGCAGTAGTAATGGCAACCAAGTAACTGGTACTGAAGTTGAAC

CACAGATGATGGACGTGGAACAGTATAAGGTTAATAAGGAAAAAACAGAGCTTACTGTTA

AAGATGACAAACAGCAGCTCAAGATCCGTAAAGATGTTGATGAATTAAAAAATAAAGATC

TTTTTGATGTCAAACGTGAAGTAAAAGATAATGGCGACGGAACCTTAGATGTAACCTTAA

AAGTAATGCCTAAACAAATTGACGAAGGTGCCGATGTTATGGCCCTTTTAGATGTCTCTC

AAAAGATGACACAGCAGAACTTTAATAAGGCTAAAGAACAGATTAAAAGATTAGTCACAA

CTTTAACAGGCAAATCATCTGATGGAAAGGAAAATCATAATAGGCGTAATTCTGTACGTT

TAATGACCTTCTACCGTAAAATCAGTGAACCAATTGATTTATCAGGAAAAACTAGCGACG

AAGTCGAAAAAGAGTTAAATAAGATATGGGATAAAGTCAAAAAAGAAGATTGGGATTGGG

GCGTTGATTTACAGGGTGCTATCCATAAGGCTCGAGAAATTTTTAGATCTTCTTACGAAA

AAAAGTCGGGCAAACGCCAACATATCGTCCTGTTCTCTCAAGGCGAGTCAACCTTTAGTT

ATGATATTAAAAATAAAAATGATAATAAACTCACAAAAGCGAGAATAGAAGAGGAGGTCA

CGTCCTCTAATCCTCTTTTATCCTGGCCACCAATTTTTAATCATACGAATCGAAAAGCAG

ATATGCTTAATGATATAGAGTATCTTATAAAACTAGGTGAAAGATTGGGAATTACGGGTC

TAGATAGTTTAAAGAATACACTGAAGTTAGCGTCGACAGGAAGTTCTATAGCAGGCTCAC

TTTTAGGCAGTGGTAGTCTGTCAGAGTACCTTACCTTGAAGGAGTATGAGTCTAGAACAT

TGAAGGAAAGTAATTTTGATTATACTAAACGTGTGGGCGAAGGGTATTATTATCATAGCT

TTTCTGAAAGGATACAAAACGAGTTACCACTTAAGTCAATTATAGAGCCTCAACTAAAAG

GATTATTTAAAACAGAGGATAGTAGTTGGTTCGGTAGGTTTCTCAATAAGTTTTCATTAG

CGAAGGGTTATCAAGAATTTAAAGAAACTGCCCTTTTAAAAGTTCTCGAATATTTGTTCT

ATAAACGTGAATATATTTACTACAATCACAATCTCTCAGCAATAGCTGAAGCCAAAATGG

CTCAACAAGAAGGCATCACCTTCTACTCCGTTGATGTCACGTCTCCAAATCAGTCGGCTA

ATAAAAGGACTAGGAGGTCAGCTGATACACCGGAGGAAAAACGTAACAAAAAGTTTGATA

ATTACCTAAAAGAGATGTCCGAAGGAAGAAAATTCTTAGAAGGTCAGGATGTCACAAATA

AAGATAAATTTAAAGATACTTTAACAGAACTAACGATTAAAGATGAATTCACAGAAAAGG

TTACAGTTCAGAAAGATTCAAAAGATAAAGAATATTATAAAACTAGTTTAATAGATGACA

GACCTAAAGTAACACATCAAGCCCCTTATAGCGGTTGGTTAAACAGTACCAAAGAAAGCC

TCACTTGGACCATTTCCAAAGACCAGTTGAAAAAAGCTTTTGAAAGTGGTCAACCATTAA

CCTTAACTTATAAGCTAAAAGTCGAGAAGGAAAAATTCAAAGAAGCACTAAAAAAACAAC

AAGAACGAAAAAAACGAGCAGCATCTCCGGAAAGTGAGAATACTGTCACAGACACAATTA

TTTCAAATAAGATTTCTTACAAGATTAATAATGGTACGGATATAAATAGCAATAACAATA

AGTTGGAAGATGTTAAAATGTCTTACAGCAAGTTCAAGATGCCTATACCAGAACTTGATA

TAGAAGTTGTACCAATACCAGAAAAACCACTGGTAGAACCAATGACGCCTCTATATCCTG

CAATTCCTAATTACCCAACACCAGATATCCCTACCCCTCAACTTCCAAAAGATGAAGACC

TGGAGATTAGTGGAGGTCATGGACCGATTGTCGATATCGTCGAAGATACTGGTACAGGTG

TTGAGGGCGGCGCTCAAAACGGCGTGGTTTCAACTCAGGAAAATAAAGATCCAATCGTTG

ACATCACCGAAGATACCCAACCAGGTATGTCAGGCTCTAATGACGCAACAGTTGTCGAGG

AAGACACAACACCTAAACGTCCAGATGTTCTTGTTGGTGGTCAAAGTGATCCAATCGACA

TCACCGAAGACACCCAACCAAGTGTATCAGGCTCAAATGACGCGACAGTTGTCGAGGAAG

ACACAGCACCTAAACGTCCAGATATCCTTGTTGGCGGTCAAAGTGATCCAATCGACATCA

CCGAAGATACCCAACCAGGTATGTCAGGCTCTAATGACGCTACTGTTATCGAAGAAGATA

CGAAACCAAAACGCTTCTTCCACTTTGATAACGAGCCACAAGCACCAGAAAAACCTAAAG

AGCAACCATCTCTACAAGATAGTAACAGCTTACCACAAGCTCCAGCCTATAAGGCAGCTC

ATCACTTGCCTGCATCTGGAGACAAACGTGAAGTATACTTTACAATTGCTGCTCTAACAA

TTATTGGAGCTGCAGGTTTGCTCAGCAAAAAACGTCGCGACACCGAAGAAAACTAACCCT

CGTTAGTCTAGCGACCCTAAGCCTTTGGCTTTAAAAATTTCTCATTATCCCCCGTCAAAC

ACCGGTACCTCACTGAGATGGCGCTTAAAAAAGCCGCTAGTCTCAAATCAGGTATCCCCT

ACGGTTGAGGGGGATTAATGGGTATCACAACAAGAAAAAAGGTATAATGATGAAATATGA

CTAAAAAATTTATTAAATCAGCAGTGATTCTAGGCTTAGCAAGCATAACCATCAGCATGA

CGCAACCCGTTGAAGCCATTGCAAATCGCTTAGAATATCAAGAACAATACGAACGTGTCA

AAAAAGAACTAGACGCATTAAAAGAAAAGGGCCCAAAATTAGGAGAACGTTCCCTTTATT

TTAGAGTGGTGAATAAATTTCTAAGTTGGTTTGGCCAAGTCTGGGACCCACTCGCAATCG

AAGAAACCGATCACCTGTTGGACCTCATCGGACAAGTCAATTACTTGGTGAGTAACACCG

AGTTTCAAGCCCCTGAGTACCAAGAGGCTAAAAACAAACTAGATTTAGCTGTAGCAGGCT

ATGACCTCTACCTATCTAGAGAAAAAGGACGTGGTGATGCTCCTGATTACCAAGTGACCC

AGCTAACGCTAGAACAGATCGCAACCATCAAAACGAAGATACAAGAGGCCCTGAAAACAT

TTGCACAAGAGGTCAAAACAATCCAACAAACACACCCAGACCTATTTCCCCATGCTTATG

TTTTAAACGCTAAGACCAGTGCTATCGATGGTAAAGCGCAACCCTTTTTAGTGAATGGTT

ACTACGATGTCTTTTATAAGCAGTTTTTATTCACTAATACAAAGGACGGGTATAACTATA

ATAAAGAAGCAGACAATCTGACCTCGTATCAAAATCTGATTAAAGAACTATTAGAGTTAA

AAACTAAGATCGATCAGTTAACAACAGACAAGCCTAAAAACGATCTTTTAGAGACGTTAA

TGTGGCAATCAAAGTTCACCTTATCTGACTTTCGGAGAACTTCACGCCTTAGCGCCTACT

ATACACAAGAAGATTATGTCAAAAAGGTGGTCCTATCTGAAGAAGCTCCCAACTTGAGAA

CAGCAAAAGAAAAGGTCAAGACCTTAAAAGGGATCTTGCATGATTACTATGTTGAAACTA

ATGATTTAGAAAAAGCTAAAGAGTATCATGTCGAGGACGAAACCTCACCGTCTCAACCTG

AGGCCCCTGCTAAACCTGAAGCTCCTAGTCCTAGTCCTGCTCCTGGTCAAAAACCAGCAG

AAGATGAGAGATCATCACAAGCTACGGAACCAGCTAACCCGTCAAAAGAGGATTCCTCAA

CAGACGCATCTCAGGGATCTCACGATAGTGAAAACCCAGCGACAGACAGTCCTTCTCAAC

CTCAGGCACCAGACCAAGGCAATCACCAAAGCCAAGTGCCTAACGACAAGCCTCAAACGG

ACAAGACGGACACTCCTAACGTCCCAGCCCCTCCACAGGACACCCCAAAAGTGCCTGAGG

CTGGTGGTCAGAGTGGCCCCGCTGGGAACGCTGAGGAGAAAGCTCCTGACTCTGGACCTA

AAGAGTCTGATCAAAGCTCATCTAAAGAGTCCCCATCCGTTGGCGAAGACACGACCCCTA

ATCAGCCAGACGTCCTTGTAGGTGGTCAAAGTGAGCCAATTGATATCACCGAAGACACAA

TCACTGACGCTCCTCCGACAGTATCTGGGCACAACGCATCAACACAGCCCCAATCTGTGG

TCGAAGACACAGCACCTCAGCGCCCAGATGTCCTTGTAGGTGGTCAAAGCGAGCCAATCG

ATATCACCCAAGATACCCAACAGGGCATGTCAGGCTCTAATGACGCTACCGTTATCAATG

AAGATACAAAACCAAAACGCGTCTTCCACTTTGATAACAAAGAGTCACAAGCATCAGAAA

AAGCGGCAGAGCAAAAACTTGCTCCTCATGATAGCCACACAACCCCTCAAGCTTCAGATG

ATACAGCAGCCCCTCACTTGCCTGCATCTGGAGACAAGCCTGAAAAAGTGTTTGCACTTG

CTGCCTTGACAATGGTCGCAGCTGCAGGGCTACTCAGCAAAAAACGTCGCAACACCGACA

AAAACTAGTCGCTGTTAGGCACAACGCATCACTAAGGCCAGTGCAAATGCACTGGCCTTA

GTTAGGTTTTTATCAAATAGAGGGTTTTTCATGACAATTGCTAGCATTTCGGTGTAAAAT

AAAGGTAATAACCAGCAAAGGAGCAACCCATGACAAAGCAAGATCAGTTAATCGTTGAAA

AAATGGAACAAACCTATGAGACCTTTAGCCCAAAGCTAGCAAATCTCATAGAAGCCCTAG

ATGCCTTTAAAGAGCATTATGAGGAATATGCCACACTTAGAAATTTTTACAGTAGTGACG

AATGGTTTCGGCTTGCAAACCAACCTTGGGATGATATCCCGTGCGGTGTCCTATCTGAAG

ACCTCCTCTTTGATATGATCGGTGACCATAACCAACTACTAGCTGATATCTTAGACTTGG

CCCCTATCATGTACAAGCATATGTAACCATCGCTAACGTCACAGATACTACGATGACAGG

ACAAAAAAAGCTTGAAAATCTCAAAAAAAACAGATAAAAAACAGATAATCGTTAGAAAAA

CAGTAGATGCGTCTGTTAAACTAAAGGTATCAAAACAAAAGGAGCATTTCATCATGAAAT

TCAAAAAAGTATTAGTCATTCCTGCATTAGCATTAGCAGCAACATGCTTTTTAACAGCTT

GTGGCACAAAAAAAGATTCTAAAAAAGAAGAAGTTAAAGAAATCAAAATGTCTGACATCA

AAGACGACGCAGTTAGCAAAAAAACAAAAGTGGTTGATGGCGAAGAAGTTACAGAATACA

CCACAAAAGATGGAAACGTGATTCAGATTCCTGCAGGAAACGAAGAAGGCATGGAATCAA

AAGATGCTGGTGGATCAGGCGCTCCTGCTAAAAACTAAGGGTTACAACAAAGAGCATAAG

GTGACTCGCAAACGTTACGCATGACATGAGGCAGTCTGCCTAAGATGGTAACTCACTTAT

CATAATGCCTAAATAGCCAAACACACTAACTCGCAAATGTACAAAATCATATACCCGTGC

TTATGTGAGCGAACGGTTATATGATTTTTGTTTTCCAAAAACAAGAAAAGAACGCTAAAT

TAGCCAGTTTGTCGTGTTTCAAGCTACCAAAAAGGAAGTGCTAAAATGAAAAAAAGATCC

ATTATACCTGTCATCTTACTGTTGGTCATCGGATTAATCGTTGGTGTTAAGCGTCATCAT

GACTAGTAGGTCTTGCTAGTGACCGTTCAAAAGGGATAACGTTGGTATGGTATAACCATA

AAAATCGTATAAAAAGAAAAAATAGCTATTGAGGAGAAAGTATGTTTCAGTTAAGAAAAA

AAATGACGCGCAAACAATTAGCCTTGTTGAGTGCTGGAGTGTTGACTTGTGTGGTTGGTG

GTAGCTACTTGATAATGAACCATCAACAACAAGAAATTGTTTCTAGTGTCAACAAAGTAA

AAGCCTTAACCATAAAAGAAGCCATGGAACAAGGAAAAGATATCAGCTTGACCTTAGCTG

GCGAAGTAACAGCTAACAACAGCAGCAAAGTCAAAATCGACTCAAGTAAAGGAGAAGTCA

AAGATGTCTTTGTCAAAAAAGGCGATGTTGTCAAAGTAGGACAACCCTTGTTTAGCTATG

AAACGTCACAGCGGTTAACGGCTCAAAGTTCAGAATTTGATGTTCAAACCAAAGCCAATC

AACTCCAAGTTGCTAAAACCAATGCAGCATTGAAGTGGGAAACCTACAATCGCAAGGTCA

ATGAAATCAATACCCTAAAATCTCGCTACAACACTGCACCAGATGAGAGCTTACTAGAGC

AGATTCGCAGCGCAGAAGACAGTGTATCCCAAGCACTAAGCGATGCCAAAACAGCAGATA

GCGATGTCAAAACTGCTCAAATCGAACTCGATAAAGCTAATGCTACTGCCACAACGGAAA

AAGGTAAACTAGAGTATGACACCGTTAAGTCAGACACCGCAGGAACCATTGTTAGCCTAA

ATACTGATTTGCCAAATCAATCAAAATCCAAAAAAGAAAATGAAACTTTTATGGAAATTA

TCGACAAATCAAAAATGTTAGTCAAAGGTAACATCAGTGAATTTGACCGTGACAAGTTAA

AAATCGGTCAAAAAGTCGAAGTGATTGACCGCAAAGACAACTCTAAAAAATGGACTGGAA

AAGTAACCCAAGTTGGCAACCTCAAAGCAGAGGAAAAAGGCCAAGGTCAAGGCCAAGGTG

GCAATGACCAACAAGACAATCCAAACCAAGCAAAATTCCCTTATGTTATCGAACTTGACC

AATCAGACAAGCAGCCACTCATTGGCTCACACACCTATGTTAATGTGCTCAACAATGTTC

CAGAAGCTGGCAAGATCGTATTGAAAGAAACCTTTACAATGGCAGAAAATGGAAAAACCT

ATGTGTGGAAAGTTGATAAAAACAAGGTCAAAAAACAAGAAATCAAGACTAAGCCCTTCT

CAAAAGGTTATGTTGAGGTAACAAGTGGCTTGACCATGCAAGATAAGATTGCTCAGCCGC

TTCCTGGCATGAAAGACGGTATGGAGGTAGGAAGTATTGTTAAACCTTAAAGATATTCGA

AAAAGCTATCATCTTGGAACTGAAGAATTTGCGATTTTAAAAGGAATCGATTTAGAAGTT

AACGAGGGTGACTTTTTAGCCATCATGGGACCATCAGGTTCGGGAAAGTCAACATTGATG

AATATCATTGGGTGTTTAGATAAGCCTGGCTCTGGCTCATATGCCATTGAAGGCAGAGAC

GTGTCATCCTTATCTGATAATGAACTTGCTGATTTGCGTAATCAAAAAATCGGCTTTGTT

TTTCAAAATTTTAACCTGATGCCCAAGCTAACAGCTTGTCAAAATGTCGAATTGCCCTTG

ACTTATATGAATGTTCCTAAAAAAGAACGTCGCAAACGAGCCCTAGAGATGTTAAAGCTC

GTAGGATTAGAAGAACGTAGTGAATTTAAACCGATGGAGCTATCTGGTGGGCAAAAACAG

CGTGTAGCGATTGCAAGAGCTTTAGTCACTAACCCGAGTTTTATCCTTGGTGATGAGCCA

ACAGGTGCACTAGACACAAAAACCAGCGTCCAAATCATGGACCTATTTAAACAATTCAAT

GATAACGGCAAAACGATTATTATCATCACACACGAGCCTGAAGTAGCTGCCTTATGCAAA

AAGACGGTGATCCTAAGAGATGGTAATATAGAACATTCCGATATAGAGTAAAGGGGGAAA

ATAATGGAAGATATTATTGCAGCTTTAGGCTCTATATTATCACACAAGATGAGGTCAATT

TTGACAATGCTAGGGATTATTATCGGTATTGGTGCCATTATCGCAATCTTTTCAATCATC

GAAGGTAATACCGAAAATACCAAAAGACAGTTGATTGGTGGCAGCAATAACACTATTAAC

ATTGTGTTTAACAAAAAAAGTAGTATTGACCCTAAATTTCCAGACAAGTCAAATGCTAAA

AAACCAGACTATCTTCCTTTTATGGCTGAAGAAGAGCTTAGCAAAATACAGCAAGTCAAA

GGCGTCAAAAACGCCCTGATCTCATATGGTATTGATGACAAAGTCTACCATCTAGGCCAA

AAATCATCTGCAAAGATTTCTGCCATCACAAAAAACGTAGCTGAAGTGAGGCGCATGACA

TTTATTAAAGGCAGTGACTTTTCAGACAAAGACTTTATTGACCAAAAACAGGTGATTTAC

CTTGAAAAAAGCTTGTATGAGTCACTATTTCCAAAAGACGATGGCCTTGGAAAATTTGTC

GAAGTGATGGGAAATCCCTTCAGGGTCATTGGGGTGTTTGAATCAAAAGAACAAAGTGGC

CTCACAAGTGGGACTGAGAAAATTGCTTATATCCCGCTACATCAGTGGTATAATATTAAT

GGTGTAGTGGATGCGACACCTGAGATTACGATCCAGACCTATCGCGCAGATGACCTCAAA

CCTGTAGCAAAGCGTGTCAGTGATATGCTCAATCAAACGATCCCTAAATCAGATTATATG

TTTGGTGTCATGAACCTTAAAGAATTTGAACGCCAACTAGACAACCTAAACAAATCTAAT

TTTGTGTTATTAGCTGGTATTGCTAGCATCTCTCTTATTGTAGGTGGTATTGGTGTGATG

AACATTATGCTAGTATCAGTTACCGAAAGAACGAGAGAAATTGGGATCAAAAAAGCGCTT

GGCGCTAGACGTAAGCTTATTTTAAAACAATTTTTAATTGAAGCGGTCATTCTAACCTTG

CTAGGTGGTGTGATAGGGGTTATTTCAGGGATGGTATCAGGATTGATCATCACAAGATCA

CTCGAGTATCCCTATATCCTATCCTTGTTCTCAGTTGTCTTGAGTTTAGCGTTTTGTTGT

ATTATAGGGATTGTCTTTGGACTATTACCAGCCATTAAAGCGTCTAAGCTAGACCCAATT

GAAGCATTGCGATTTGAATAATCAACCAAACAAGCATAAAAGTCAAAAAGGATGACCCTT

TGATGTCATCCTTAGCTGCTTGATACACTAAACGCCAATCTAGAAAGGGAAGTTATGTTT

AAAATATTAGTAGTTGAAGATGATGATACCATCAGTCAAGTGATTTGTGAGTTTTTAAAA

GCGAATAACTATGATCCAGATTGTGTATTTGATGGCCAAGCTGCCTTAGACAAGTGGCAG

ACAACGTCTTATGATCTGATTATTTTAGACATCATGCTCCCTTCCTTAAGTGGCTTAGAA

GTGCTAAAAACAATTCGAAAGACATCAGATGTCCCTATTATTATGCTAACGGCATTAGAT

GACGAGTACACACAGCTTGTAAGCTTCAACCATTTGATTAGTGATTATGTGACCAAACCA

TTTTCACCACTCATTTTAATCAAACGTATCGAAAATGTATTACGCGTCTCTACTCCAGAT

GAAAAGCGCCAAATCGGAGACTTACTTGTTGATGAGACCGAACATAGCGTCTATTGGCAA

GGAACATTGGTCAAACTAACCAAAAAAGAATATGACATTATTGATTATTTAGCAAAACGT

CACCAAAAAATTGTCACCAGAGACCAATTGATGGATGACATATGGGGCTATTCTGAACTG

GACACGCGTGTCCTTGACAACCACATCAAGAACCTTCGTAAAAAAATGACGGGTATTCCC

CTCAAGACAATTACAGGCATGGGCTATTTATTGGGAGAGCGTGAGTGAGACTAATCAAAA

AAACCTTTTTGGTCATCAATGGCTTAATTATTGTAGTGGTTACCTCTATTTTGTTAGTGC

TTTATTTTGCAATGCCAATTTATTACACCAAAGTCAAAGACAAAGAAGTTAAGCGTGAAT

TTGACCAAACAAGCAAGCAAATCAAAGGCAAAACGGTAACCGAGATTAGAGATATCCTAA

CAAAAAAAATCAACAAAGATAATATTTGGTACAGCTTAGTAGACAGTGATAACCAACTGC

TCTATCCCTCCTTGCAGCTCCTAGACGGCGTCAGTGAAAGCAAAGACAGTCAAAATGTTA

ATATCGTCACCACCTTTGACAACTCTTATTCTAATGTTAAGGTGATGAGTCAAAAAGTAA

CCCTACGCGATGGAAAAAAGATGACATTGCTTGGGCAATCTTCACTTCAACCCGTAACAG

ATGCTAGCAAAGTTCTCCTTGACCTCTACCCGTCACTATTGATGTTTTCAGTGACCGTAG

GGAGTATTGTCGCCTACTTATATAGTAGGACTTCTAGCCGGCGTATTCTTAGCATGTCAC

AAACCGCCAAAAAAATGGTCAACCTAGAACCAAACTTAACGTGTACCATTCACGGCAAAG

ATGAAATAGCAATGTTGGCTAGTGATATTAACCGTTTGTATGCGAGTCTTTCAACGAGCA

TCAAATCCTTGCAAAAAGAGTATGAAAAAGCTTCTGATTCAGAAAGAGAGAAATCTGAGT

TTTTACGCATGACATCGCACGAGCTAAAGACACCAATCACCAGTGTTATCGGAATGATTG

ATGGAATGCTCTATAATGTTGGTGACTTTGCAGATCGTGACAAATATTTGCGAAAATGCC

GAGACGTCCTTGAAGGGCAAGCCCAACTGGTTCAATCCATTTTATCTTTATCTAAGATTG

AAACCCTAGCTTCTCAAAATCAAGAACTGTTTTCACTAAAAAGCAGTCTAGAAGAAGAAA

TGGAAGTCTTTCTCGTCTTATCAGAACTAAAACACCTCAAAGTGACTATCAATCTCGAAG

AACAATTCGTCAAAGCCAATAAAGTATACCTACTAAAGGCGATTAAAAATATTATTGACA

ATGCCTTTCACTATACCAAACCAGGCGGCCAAGTGATGATTCAACTAAAAGACAACCAAC

TAGTGATTAAAAATGAAGCAGAGACATTATTGACACAACAGCAGATGAAACAGTTATTCC

AACCGTTTTATCGACCAGATTATAGTCGTAACAGGAAAGACGGTGGTACAGGATTGGGGC

TGTTTATCACCCACCAGATTCTTGATCAGCATCATCTGGCTTATCGCTTTGTTGTTCTTG

ATCAAAGATGGATGGTATTTACGATTGATTTTCCATCCCATCATGACGATTGATCCACAC

TAGTAGGACGACAAAAGTCATCAATCACTAACCAAGTCTTGACCTTTACCCATCATTGTT

TAGGCAAGTATAAAAAAGAGGAGAGAAATCATATATGAAGAAAAGGAAATTGTTAGCAGT

AACACTATTAAGTACCATACTCTTAAACAGTGCAGTGCCATTAGTTGTTGCTGACACCTC

CTTGCGTAATAACACATCATCCACTGATCAGCCTACTACAGCAGATAATGATACGGATGA

CGAGAGTGAAACACCACAAAAAGATAAAAAAAGCAAGGAAACAGCGTCGCAGCACGACAC

CCAAAAAGACCATAAGTCATCACACACTCACCCAACCCCCCCTTCAAATGATACTAAGCA

GACCGATCAGGCATCATCTGAAGCTACTGACAAACCAAATAAAGACAAAAACAACACCAA

GCAACCAAACAGCAGTGATCAGTCTACCCCCTCTCCCAAAGACCAGTCGTCTCAAAAAGA

GTCACAAAACAAAGACGGCCGACCTACCCCATCACCTGATCAACAAAAAGATCAGACACC

TGATAAAACACCAGAAAAATCAGCTGATAAAGCCCCTGAAAAAGGACCAGAAAAAGCAGT

TGAAAAAACACCAGAGCCAAATCGTGACACTCCAAAACCCATCCAACCTCCTTTAGCAGC

AGCAGCTCCTGTCTTTGCACCTTGGAGAGAAAGTGACAAAGACCTGAGCAAGCTAAAACC

AAGCAGTCGCTCATCAGCGGCTTACGTGAGACACTGGACAGGTGACTCTGCCTACACTCA

CAACCTGTTGTCACGCCGTTATGGGATTACTGCTGAACAGCTAGATGGTTTTTTGAACAG

TCTAGGTATTCACTATGATAAAGAACGCTTAAACGGGAAGCGTTTATTAGAATGGGAAAA

ACTAACAGGACTAGACGTTCGAGCTATCGTAGCTATTGCAATGGCAGAAAGCTCACTAGG

TACTCAGGGAGTTGCTAAAGAAAAAGGAGCCAATATGTTTGGTTATGGTGCCTTTGACTT

CAACCCAAACAATGCCAAAAAATACAGCGATGAGGTTGCTATTCGTCACATGGTAGAAGA

CACCATCATTGCCAACAAAAACCAAACCTTTGAAAGACAAGACCTCAAAGCAAAAAAATG

GTCACTAGGCCAGTTGGACACCTTGATTGATGGTGGGGTTTACTTTACAGATACAAGTGG

CAGTGGGCAAAGACGAGCAGATATCATGACCAAACTAGACCAATGGATAGATGATCATGG

AAGCACACCTGAGATTCCAGAACATCTCAAGATAACTTCCGGGACACAATTTAGCGAAGT

GCCCATAGGTTATAAAAGAAGTCAGCCACAAAACGTTTTAACCTACAAGTCAGAGACCTA

CAGCTTTGGCCAATGCACCTGGTACGCCTATAATCGTGTCAAAGAGCTAGGTTATCAAGT

CGATAGGTACATGGGTAACGGTGGCGACTGGCAGCGCAAGCCAGGTTTTGTGACCACCCA

TAAACCTAAAGTGGGCTATGTCGTCTCATTTGCACCAGGCCAAGCAGGAGCAGATGCAAC

CTATGGTCACGTTGCTGTTGTAGAGCAAATCAAAGAAGATGGTTCTATCTTGATTTCAGA

GTCAAATGTTATGGGACTAGGCACCATTTCCTATCGGACGTTCACAGCTGAGCAGGCTAG

TTTGTTGACCTATGTCGTAGGGGACAAACTCCCAAGACCATAACCCCACAACCAAGAAGC

TACCTGCCTGTTGACCAATCTCAAAAAGAGACAGCGTCAACTTAGAATGAGGTTCATCAG

ATGATAAAGAAAGTGACCACCCCGAGTCAAAAAACAAAAAAACGTGTTCGTAATGGCTAT

CTCCTAAAACTAGGTACAGCCTGTTTACTCTTAAGTATACTCAGTTATGGCATTGGTCTT

TTGGGCCAGCCTAGCATGGAAAATACGTTTGTGGGAATAGCGAGTGTTGCCATGTTAGGA

AGTGTCTGTTTCTTTATTATCTTTGCTCTAAACCGTATCTTTGACGCACTAGAGGACAAT

TTGCGAGATTAGAGTAATAGGTCAAATAATCTTCCAATAATTTAGAGCACAAGCTAAGTT

ATTGGAAGTTTTTTTGTCTTTTGAGAGCCATGATGCCATCAAAAAGTCAAACACTCCTTA

TGATGTGAGAGTCACATGGCTAAAATTAAAAGCAAAATAGACCCCAAAGCCCTGTTTTCT

CAAAACAGTCAACGAAAAAAGACAAGTTAACCAGTTCATGAAAAACATCGTAGAAACATT

TTCAAGAGCTAATGTTGGTGTAAATTGACTGAAGTATGATAGAATTTTTAATGAATGTGA

CAATAATGTCACAGATAAAAACCGAAAACAGCAAAAATAATTAAATTGAATATAAAAAAG

TTAATTAAGTGAGTGATAATAAAAAAATGAAGTTCAAAAAATATAAATAAAGTAAAACAC

CAGAACTACTAAAAGCAGTGAATGACATAAATGTCGCTAAAAAAATAGATAGAAGAGTCT

AAAATTTCAGTTTTAGGTCACGTTTTATGAATGGAAATCAGGATGATGACCTTAAGTTGT

GATTTGCGCGTCGTTGACCGCTTTCCTCGTTTTAATGAAAGAATTTGAAAGATATTTTAT

GGTATACTTTTCCTTAATATGGTTCATACGGACTTGATAAATCTATTTACAAAAATAATA

AAAAATTAAGATAAGGACATGAAGTTAATGCATGTAAGTAAATTGTTTACTAGCCAACAA

TGGAGAGAATTGAAACTGATTTCATATTTAACAGAAAACTCTAATGCTATCGGAGTTAAA

GATAAGGAGCTTAGCAAGGCTCTCAATATTTCCATGTTAACATTACAGTCATGCCTCACT

AATATGCAGTTCATGAAAGAAGTAGGTGGAATTACCTACAAAGACGGCTATATTAATATT

TGGTATCACCAGTGTTGTGGGCTACAAGAGGTCTATCAAAAAGCTCTTCGAGAATCGCCT

TCTTTGAAATTACTAGAGCTTTTGTTTTTCAGAGACTTTAGTTCCTTAGAAGAGTTAGCT

GAAGAGCTGTTTGTCAGCTTATCTACCCTCAAACGCCTCATCAAAAAAACCAACACTTAC

TTGTCTCATACCTTTGCTATTAGTATCGTGACAAGTCCAGTGCAGGTGTCAGGAGATGAG

AGGCAAATCCGCCTGTTTTACCTCAAGTATTTTTCAGAAGCCTATAAAATCTCAGAATGG

CCATTTGGGGACATTTTAAACCTGAAAAACTGTGAACGCCTATTGAGCTTATTGATCAAA

GAAGTAGATGTCAAAGTTCATTTTACACTATTTCAGCACTTAAAAATCTTAAGTGGTGTC

AACTTGATCCGTTACTACAAGGGGTATTCTTGCAGTTATAACAATAAAAAAACCAGTCAC

CGTTTTTCTCAACTTATCCAACATTCCTCAGAAATCCAGGATCTTTCTCGCTTGTTCTAC

CTCAAATTTGGGTTACATCTCGACGAGTATACCATAGCTGAAATGCTTTCTAACCATCTT

AACGACAAGCTAGAAATTGGCTGTGCTTTTGAGATTATCAATCAAGACCCGACATCAGGA

GGCAGACAAGTAACCAACTGGATCCATCTATTAGATGAGATGGAAATCAAGCTCAATCTC

AGCATCACCAATAAATACGAAGTTGCCGTGACCCTCCATAACGCTTCGGTGTTAAACGAA

GAAGACATTACAGCCAATTATTTGCTGTTTGATTATAAAAAGAGTTACCTGAACTTTTAC

CAAAAAGAACACCCTCGCATTTATGAGGCCTTTGTGACAAGTGTCGAGAAGCTGATGCAG

GCAGATAATGCTCAAGTTAGCAAAGAACTGATCAATCAGCTCACTTATTGTTTCTTTATT

ACCTGGGAAAATAGTTTCTTAAAAGTAAATCAAAAAGATGAAAAAGTACGACTCCTAGTG

ATAGAGAGAAGTTACAATAGTGTCGGTAATTTTTTGAAAAAGTACATCGGTGAGTTTTTT

AGCATCACGAACTTTGATGAGTTAGATTGTTTGACAATTGATCTAGTAGAGATTGAAAAA

CAGTACGATGTTATCGTGACAGATGTTATGGTGGGTAAAAGCGAAGAGTTAGAGATTTTC

TTTTTCTACAAGATGATTCCAGAAGCTATTATTGATAGATTAAACGAATTTTTGAACGTT

AGTTTCACAGATAACAACGTTATGGTCAAAAACCTCGAAGCCCCTTCCTCTTCAAAATCT

CATAGTGACAAAGAGGTACAAAAGCCAGAAAAGCCAGACAATTCAGTTAAACAAGCAACA

TCATCATAGGATTTCAGACGTCATGGTAAGAAAAAAAGAATCATTTACAGCCCATAGAGG

TAAGGTCAAAAGCTGAAAACAGCTCAAAAAAACTGACCTTTACCTTTTGGGTTTTTTTAT

TTAGAATAATTTTATTGGAGAGATGCTTAATAATTTAAGCACAATTCTTAGAAATTGAGA

AATAAGGAGTAAACAATGTCTAAAAGAAATCCAAACAAACTCTATTCACTGAGAAAGTTA

AAAACAGGTACTGCATCAGTAGCAGTAGCTTTGACAGTTTTGGGGACTGGATTAGCAAAC

ACAACTGATGTAAAGGCTGAGAGTCGTCGTTATCAGGCACCTCCTCGTGTGTTACTGCAA

GGCAAAGAAGCTAACAAAGTATTCGAAGAGCGCAAAGCCTTGGAAAAACAAGCACGTGAT

TTGGGTGACACTATTAACCACATGTCACAAACCATTAGCGAGCAAAGCCGCAAGATTGCA

GCACTAAAGTCTGAAGCAGAACTTAAAAACCAACAAGCTCTTGAAGCTTTAAACAATAAA

AACAAGCAAATCTCAGATTTAACCAACGAAAACGCACAGTTAAAAGAAGCCATTGAAGGT

TATGTGCAAACTATCCAAAACGCTAGTCGTGAAATCGCAGCAAAACAACAAGAACTTGCA

GCTGCAAAAAGCCAGTTAGAGGCAAAAAATGCTGAGATTGAGGCATTGAAACAACAAGAT

GCCTCTAAGACTGAGGAAATTGCTAAATTGCAATCAGAAGCAGCAACTTTAGAAAACCTC

CTAGGTTCAGCTAAGCGTGAGTTGACTGAATTGCAAGCTAAGCTAGATACAGCAACTGCT

GAAAAAGCAAAACTAGAATCACAAGTAACAACCTTAGAAAACCTCCTAGGTTCAGCTAAG

CGTGAATTGACTGATCTGCAAGCTAAGCTAGATGCAGCTAACGCTGAAAAAGAAAAGCTC

CAATCACAAGCAGCAACCCTAGAAAAACAACTAGAAGCAACTAAAAAAGAGTTAGCTGAT

TTACAGGCTAAATTAGCAGCAACCAACCAAGAAAAAGAAAAGTTAGAAGCTGAAGCAAAA

GCTCTTAAAGAGCAATTGGCTAAACAAGCTGAAGAGCTTGCTAAGCTAAAAGCAGATAAA

GCTTCAGGAGCTCAAAAACCAGATACTAAACCTGGCAATAAAGAGGTTCCAACAAGACCG

TCACAAACAAGAACAAACACTAATAAAGCTCCTATGGCTCAAACAAAGAGACAATTACCG

TCAACAGGCGAAGAAACAACCAACCCATTCTTCACTTCAGCAGCATTGACAGTGATCGCA

TCTGCAGGCGTACTTGCCCTAAAACGCAAAGAAGAAAACTAAGTCCAACCCACATTATCT

TTTCTAGCCCAAGAAAAAAACAAAAAAAGAGGAAGCCCCTTCCTCTTTTTTTGAACGGTT

AAACAGCAAAAAGGTCAAAAAGGCACTAAAGTCTCAAAAACCTGGTCTTTACCTTTTACC

GCTCATTCTTTAGAATAGAATTATTAGAGAGAAGTCTTAGAAAAATGAGGCTAATTCCCT

AAAAGATGAAAAAAATAAGGAGCAAATAATGGCTAGAAAAGATACGAATAAACAGTATTC

GCTTAGAAAATTAAAAACAGGTACAGCATCAGTAGCGGTCGCTGTGGCTGTTTTAGGAGC

AGGCTTTGCAAACCAAACAGAAGTTAAGGCTGCGGAGATTAAAAAGCCTCAGGCTGATTC

AGCGTGGAACTGGCCTAAAGAATATAACGCGTTACTTAAGGAAAATGAGGAGCTCAAGGT

AGAACGTGAAAAATATCTATCTTATGCTGACGATAAAGAAAAAGATCCTCAATATAGAGC

ATTAATGGGTGAAAATCAAGATCTTCGAAAAAGAGAGGGACAATATCAGGACAAAATAGA

AGAACTTGAAAAAGAAAGAAAAGAAAAACAAGAAAGACAAGAACAATTAGAACGTCAATA

TCAAATAGAAGCAGATAAGCATTATCAAGAACAACAAAAGAAACATCAGCAAGAACAACA

ACAATTAGAAGCAGAAAAACAAAAATTAGCTAAAGACAAACAAATCTCAGACGCAAGCCG

TCAAGGCCTAAGCCGTGACCTTGAAGCGTCTCGTGCAGCTAAAAAAGAGCTTGAAGCTGA

GCACCAAAAACTCAAAGAGGAAAAACAAATCTCAGACGCAAGCCGTCAAGGTCTAAGCCG

TGACCTTGAAGCGTCTCGCGAAGCTAAGAAAAAAGTAGAAGCAGACTTAGCAGAAGCAAA

TAGCAAACTTCAAGCCCTTGAAAAACTAAACAAAGAGCTTGAAGAAGGTAAGAAATTATC

AGAAAAAGAAAAAGCTGAGTTACAAGCAAGACTAGAAGCTGAAGCAAAAGCTCTTAAAGA

GCAATTGGCTAAACAAGCGGAAGAACTTGCTAAACTAAAAGGCAACCAAACACCAAACGC

TAAAGTAGCCCCACAAGCTAACCGTTCTAGATCAGCAATGACACAACAAAAGAGAACGTT

ACCGTCAACAGGCGAAACAGCTAACCCATTCTTTACAGCAGCAGCTGCAACAGTGATGGT

ATCTGCAGGTATGCTTGCTCTAAAACGCAAAGAAGAAAACTAAGCTATTAGACTGATGCT

AAAGCTAAGAGAGAATCAAATGATTCTCTCTTTTTGAGTGGCTAAGTAACTAACAATCTC

AGTTAGACCAAAAAATGGGAATGGTTCAAAATGCTGGCCTTTACTCCTTTTGATTAACCA

TATATAACAAAAACATTAGGAAAATAATAGTAATATTAAGTTTATTTCCTCAATAAAATC

AAGGAGTAGATAACGGCTAGACAACAAACCAAGAAAAATTATTCACTACGGAAACTAAAA

ACCGGTACGGCTTCAGTAGCCGTTGCTTTGACCGTTTTGGGTGCAGGTTTTGCAAACCAA

ACAGAAGTAAGAGCTGAAGGGGTAAAAGCGACTACGAACTTGCCAGAGAAGGCTAAATAT

GATGCATTGAGAGACGAGAATACTGGTTTACGTGGTGATCGGACAAAATTATTAAAAAAA

CTTGAAGAAGAACAAGAGAAGAGCAAAAATCTAGAAAAGCAAAAACAGGAGTTAGAAAAC

CAAGCCCTTAACTTTCATGATGTAATTGAAACTCAGGAAAAAGAAAAAGAAGATCTCAAA

ACAACTTTAGCTAAGACTACTAAAAGCCCTCAAAGAACAATTAGCAAAACAAACTGAAGA

GCTTGCAAAACTAAGAGCTGAAAAAGCAGCAGGTTCAAAAACACCTGCTACCAAACCAGC

TAATAAAGAAAGATCAGGTAGAGCTGCTCAAACAGCTACAAGACCTAGCCAAAATAAAGG

AATGAGGTCACAATTACCGTCAACAGGCGAAGCAGCCAACCCATTCTTTACAGCAGCAGC

TGCAACAGTGATGGTATCTGCAGGTATGCTTGCTCTAAAACGCAAAGAAGAAAACTAAGC

CTTTAGAACTTGATTTTTGTAACGGTGCAATAGACAAAAGCAAGCAAGGCCAAAAACTGA

GAAAGTCCTAAAAAGCTGGCCTTTACCCCTCAAAATTAATGTTTTATAATAAAGATGTTA

GTAATATAATTGATAAATGAGATACATTTAATCATTATGGCAAAAGCAAGAAAAATAGCT

GTATCATATGCAAATAACCCCTGTTTGCTCTTTAAAAAAAGACGTTATCCTTATTTCTCT

ACGCACAGATGGACAGCTAGGAGAGAATCGTTTGATTCTCTCTTTTCTTAATGGTCATAA

AGACAAAGTCTCTCATCAGGAAAGGACGACACATTGCGTAAAAAACAAAAACTACCATTT

GATAAACTTGCCATTGCGCTCATGTCTACGAGCATCTTGCTCAATGCACAATCAGACATT

AAAGCAAATACTGTGACAGAAGACACTCCTGCTACCGAGCAAGCCGTAGAAGCCCCACAA

CAAACAGCGGTTTCTGAGGAAGCACCATCATCATCAAAGGAAACTAACCCCCCACAAACT

CCTGATGACGCAGAAGAAACAGTAGCAGATAAGGCTAATGATCTAGCCCCTCAAGCTCCT

GCTAAAACTGCTGATATACCAGCAACCTCAAAAGAGACTATTAGGGATTTGAACGACCCT

TCTCATGTCAAAACCCTGCAGGAAAAAGCAGGCAAGGGAGCTGGGACTGTTGTTGCAGTG

ATTGATGCTGGTTTTGATAAAAATCATGAAGCGTGGCGCTTAACAGACAAATCTAAAGCA

CGTTACCAATCAAAAGAAGATCTTGAAAAAGCCAAAAAAGATCACGGTATTACCTATGGT

GAGTGGGTCAATGATAAGGTTGCTTATTACCACGACTATAGTAAAGATGGTAAAACCGCT

GTCGATCAAGAGCACGGCACACACGTGTCAGGGATCTTGTCAGGAAATGCTCCATCTGAA

ACGAAAGAACCTTACCGCCTAGAAGGTGCGATGCCTGAGGCTCAATTGCTTTTGATGCGT

GTCGAAATTGTAAATGGACTAGCAGACTATGCTCGTAACTACGCTCAAGCTATCAGAGAT

GCTGTCAACTTGGGAGCTAAGGTGATTAATATGAGCTTTGGTAATGCTGCACTAGCTTAC

GCCAACCTTCCAGACGAAACTAAAAAAGCCTTTGATTATGCCAAATCAAAAGGTGTTAGC

ATTGTGACCTCAGCTGGTAATGATAGTAGCTTTGGGGGCAAGACCCGTCTACCTCTAGCA

GATCATCCTGATTATGGGGTGGTTGGGACACCTGCAGCGGCAGACTCAACATTGACAGTT

GCTTCTTACAGCCCAGATAAACAGCTCACTGAAACTGCTACGGTCAAAACAGCCGATAAG

CAAGATAAAGAAATGCCTGTTCTTTCAACAAACCGTTTTGAGCCAAACAAGGCTTACGAC

TATGCTTATGCTAATCGTGGGATGAAAGAAGATGATTTTAAGGATGTCAAAGGTAAGATT

GCCCTTATTGAACGTGGCGATATTGATTTCAAAGATAAGATTGCAAACGCTAAAAAAGCT

GGTGCTGTAGGAGTCTTGATCTATGACAATCAGGACAAGGGCTTCCCGATTGAATTGCCG

AATGTTGACCAGATGCCTGCGGCCTTTATCAGTCGAAAAGACGGTCTCTTATTAAAAGAC

AATTCTAAAAAAACCATCACCTTCAATGCGACACCTAAGGTATTGCCAACAGCAAGTGGC

ACCAAACTAAGCCGCTTCTCAAGCTGGGGTCTGACAGCTGACGGCAATATTAAGCCAGAT

ATTGCAGCACCCGGCCAAGATATTTTGTCATCAGTGGCTAACAACAAGTATGCCAAACTT

TCTGGAACTAGTATGTCTGCGCCATTGGTAGCGGGTATCATGGGACTGTTGCAAAAGCAA

TATGAGACACAGTATCCTGATATGACACCATCAGAGCGTCTTGATTTAGCTAAAAAAGTA

TTGATGAGCTCAGCAACTGCCTTATATGATGAAGATGAAAAAGCTTATTTTTCTCCTCGC

CAACAAGGAGCAGGAGCAGTCGATGCTAAAAAAGCTTCAGCAGCAACGATGTATGTGACA

GATAAGGACAATACCTCAAGCAAGGTTCACCTGAACAATGTTTCTGATAAATTTGAAGTA

ACAGTAACAGTTCACAACAAATCTGATAAACCTCAAGAGTTGTATTACCAAGCAACTGTT

CAAACAGATAAAGTAGATGGAAAACACTTTGCCTTGGCTCCTAAAGCATTGTATGAGACA

TCATGGCAAAAAATCACAATTCCAGCCAATAGCAGCAAACAAGTCACCGTTCCAATCGAT

GCTAGTCGATTTAGCAAGGACTTGCTTGCCCAAATGAAAAATGGCTATTTCTTAGAAGGT

TTTGTTCGTTTCAAACAAGATCCTAAAAAAGAAGAGCTTATGAGCATTCCATATATTGGT

TTCCGAGGTGATTTTGGCAATCTGTCAACCTTAGAAAAACCAATCTATGATAGCAAAGAC

GGTAGCAGCTACTATCATGAAGCAAATAGTGATGCCAAAGACCAATTAGATGGTGACGGA

TTACAGTTTTACGCTCTGAAAAATAACTTTACAGCACTTACCACAGAGTCTAACCCATGG

ACGATTATTAAAGCTGTCAAAGAAGGGGTTGAAAACATAGAGGATATCGAATCTTCAGAG

ATCACAGAAACCATTTTTGCAGGTACTTTTGCAAAACAAGACGATGATAGTCACTACTAT

ATCCACCGTCACGCTAATGGCAAGCCATATGCTGCGATCTCTCCAAATGGGGACGGTAAC

AGAGATTATGTCCAATTCCAAGGTACTTTCTTGCGTAATGCTAAAAACCTTGTGGCTGAA

GTCTTGGACAAAGAAGGAAATGTTGTTTGGACAAGTGAGGTAACCGAGCAAGTTGTTAAA

AACTACAACAATGACTTGGCAAGCACACTTGGTTCAACCCGTTTTGAAAAAACGCGTTGG

GACGGTAAAGATAAAGACGGCAAAGTTGTTGCTAACGGAACATACACCTATCGTGTTCGC

TACACTCCGATTAGCTCAGGTGCAAAAGAACAACACACTGATTTTGATGTGATTGTAGAC

AATACGACACCTGAAGTCGCAACATCGGCAACATTCTCAACAGAAGATCGTCGTTTGACA

CTTGCATCTAAACCAAAAACCAGCCAACCGATTTACCGTGAGCGTATTGCTTACACTTAT

ATGGATGAGGATCTGCCAACAACAGAGTATATTTCTCCAAATGAAGATGGTACCTTTACT

CTTCCTGAAGAGGCTGAAACAATGGAAGGTGCTACTGTTCCGTTGAAAATGTCAGACTTT

ACTTATGTTGTTGAAGATATGGCTGGTAACATCACTTATACACCAGTGACTAAGCTATTG

GAGGGCCACTCTAATAAGCCAGAACAAGACGGTTCAGATCAAGCACCAGACAAAAAACCA

GAAACTAAACCAGAACAAGACGGTTCAGGTCAAACACCAGACAAAAAACCAGAAGCTAAA

CCAGAACAAGACGGTTCAGGTCAAACACCAGATAAAAAACCAGAAACTAAACCAGAAAAA

GATAGTTCAGGTCAAACACCAGGTAAAACTCCTCAAAAAGGTCAACCTTCTCGTACTCTA

GAGAAACGATCTTCTAAGCGTGCTTTAGCTACAAAAGCATCAACAAAAGATCAGTTACCA

ACGACTAATGACAAGGATACAAATCGTTTACATCTCCTTAAGTTAGTTATGACCACTTTC

TTCTTGGGATTAGTAGCTCATATCTTTAAAACAAAACGCACTGAAGATTAGTACAACTGA

TCAGATGGCTACTATTGATGATGAGTTAGATCAGTCTTCTTTTCAAAAGAAGGCATAGCA

GTATAAAAATAGATATGTTAGGACAATATGTTTAGAGTAGAAAATAACAAACACAGCCGC

TATTCCATTCGCAAACTGAGCGTTGGGGTAACGAGTATAGCAATTGCGAGTCTCTTTTTA

GGAAAGGTTGCCTATGCCGTAGATAGCATCCCTCCAATCTCTCTTACTCAAAGGACTCCA

GCCACTACATCAGAAAAATGGCATCATATTGATGATAAGGGCTCTATTCCTTTAGGTATA

AGCTTAGAAGCTGCCAAAAAAGATTTTAAAGAAGAAGTAGAAAAATCACGTTTGTCTGAA

GAACAAAAACAAACGTATAATCAAAAAATTGATGCTGAAACAGACAAAGACGAACTATTG

TCAACGTATCATAGAGACTATCTGACAGCCGTTAAGAATCTTCCAACGTCTACTGAGCCA

GTTGAGGCATCCGTGCAGGAGACACAGGCATCAGTTTCAGATTCGATGGTGACAGGTGAT

TCAACATCAGTTACGGCTGATTCTCCTAAGGAAAGTCCAGTAGCCCCAGCTTCATCACCT

GAGAGTGAGGATTCTTCAGTAGCATCTTCTGAGGAAACCTCATCTCCAGAAACTCCTGAA

GAGCCAGCAGCTCCATCTCCATCACCTGAGAGTGAAGAACCTTCAGTAGCAGCTTCTTCT

GAGGAAACCCCAACTCCATCAACTCCAGAAGAGCCAGCAGCTCCATCTCCATCACCTGAG

AGTGAGGAACCTTCAGTAGCAGCTTCTTCTGAGGAAACCCCAACTCCATCAACTCCAGAA

GAGCCAGCAGCTCCATCTCCATCTCCTGAGAGTGAGGAACCTTCAGTAGCAGCTTCTTCT

GAGGAAACCCCAACTCCATCAACTCCAGAAGAGCCAGCAGCTCCATCTCCATCTCCTGAG

AGTGAGGATTCTTCAGTAGCAGCTACGACAAGCCCGTCTCCATCAACTCCAGCTGAATCA

GAGACTCAGACGCCACCAGCTGTTACTAAAGACTCTGATAAGCCATCTTCAGCAGCTGAA

AAACCAGCAGCCTCTTCACTTGTTTCAGAACAAACCGTTCAACAACCAACTTCAAAGAGA

TCTTCTGATAAAAAAGAAGAGCAAGAACAGTCTTACTCTCCAAATCGCTCATTGTCAAGA

CAGGTTATGGCCCATGAGTCAGGTAAGTACTTGCCTTCAACAGGTGAAAAAGCACAGCCA

CTCTTTATAGCTACTATGACTTTGATGTCTCTACTTGGCAGTCTTTTAGTCACAAAACGC

CAAAAAGAAACTAAAAAATAGTATTACTTAAACTGATAAGAGCTTATTTAATAGAGAGAA

CCAATTTGGTTCTCTCTATTTTTTTATATGTGCTGCCTCATCAAAAAGAAGTTAGGACAA

ATAGCTGTCAACAATTACCTTTTGCTTATCCCTTCAGTTGCTGTGATTTTATCATACAGT

GATAAAAGATGGTAGTTGCCATTGACAAAGCATTGATAAAGGAGTAAAATTAACTGGTTA

ATAACTGGTTAAATTATAATTGAGGAGGTACTATGAAAAAAGGTTTTTTTCTCATGGCTA

TGGCTGTGAGTTTAGTAATGATAGCAGGGTGTGATAAGTCAGCAAACCCCAAACAGCCTA

CGCAAGGCATGTCAGTTGTAACCAGCTTTTACCCAATGTATGCGATGACAAAAGAAGTAT

CTGGAGACCTCAATGATGTGAGGATGATCCAATCAGGTGCAGGCATTCATTCCTTTGAAC

CGTCTGTAAATGATGTGGCAGCTATTTATGACGCGGATTTGTTTGTTTACCATTCACATA

CTTTAGAAGCTTGGGCAAGGGATCTAGACCCTAATTTAAAAAAATCAAAGGTTGATGTGT

TTGAAGCGTCAAAACCTCTGACACTAGATAGAGTCAAAGGGCTAGAAGATATGGAAGTCA

CACAAGGCATTGACCCTGCGACACTTTATGACCCACATACCTGGACAGATCCCGTTTTAG

CTGGTGAGGAAGCTGTTAATATCGCTAAAGAGCTAGGACGTTTGGATCCTAAACACAAAG

ACAGTTACACTAAAAAGGCTAAGGCTTTCAAAAAAGAAGCAGAGCAACTAACTGAAGAAT

ACACTCAAAAATTTAAAAAGGTGCGCTCAAAAACATTCGTGACGCAGCACACGGCATTTT

CTTATCTGGCTAAACGATTCGGCTTGAAACAACTTGGTATCTCGGGTATTTCTCCAGAGC

AAGAGCCCTCTCCTCGCCAATTGAAAGAAATTCAAGACTTTGTCAAAGAATACAACGTCA

AGACTATTTTTGCAGAAGACAATGTCAATCCCAAAATTGCTCATGCTATTGCGAAATCAA

CAGGAGCTAAAGTAAAGACATTAAGTCCACTTGAAGCTGCTCCAAGCGGAAATAAGACAT

ATCTAGAAAATCTTAGAGCAAATTTGGAAGTGCTCTATCAACAGTTGAAGTAAAGGAGAT

TATTAGTGAAGAAAACATATGGTTATATCGGCTCAGTTGCTGCCATTTTACTAGCTACTC

ATATTGGAAGTTACCAACTTGGTAAGCATCATATGGGTTCAGCAACAAAGGACAATCAAA

TTGCCTATATTGATGATAGCAAAGGTAAGGCAAAAGCCCCTAAAACAAACAAAACGATGG

ATCAAATCAGTGCTGAAGAAGGCATCTCTGCTGAACAGATCGTGGTCAAAATTACTGACC

AAGGCTATGTGACCTCACACGGTGACCATTATCATTTTTACAATGGGAAAGTTCCTTATG

ATGCGATTATTAGTGAAGAGTTGTTGATGACGGATCCTAATTACCATTTTAAACAATCAG

ACGTTATCAATGAAATCTTAGACGGTTACGTTATTAAAGTCAATGGCAACTATTATGTTT

ACCTCAAGCCAGGTAGCAAGCGCAAAAACATTCGAACCAAACAACAAATTGCTGAGCAAG

TAGCCAAAGGAACTAAAGAAGCTAAAGAAAAAGGTTTAGCTCAAGTGGCCCATCTCAGTA

AAGAAGAAGTTGCGGCAGTCAATGAAGCAAAAAGACAAGGACGCTATACTACAGACGATG

GCTATATTTTTAGTCCGACAGATATCATTGATGATTTAGGAGATGCTTATTTAGTACCTC

ATGGTAATCACTATCATTATATTCCTAAAAAAGATTTGTCTCCAAGTGAGCTAGCTGCTG

CACAAGCTTACTGGAGTCAAAAACAAGGTCGAGGTGCTAGACCGTCTGATTACCGCCCGA

CACCAGCCCCAGGTCGTAGGAAAGCCCCAATTCCTGATGTGACGCCTAACCCTGGACAAG

GTCATCAGCCAGATAACGGTGGCTATCATCCAGCGCCTCCTAGGCCAAATGATGCGTCAC

AAAACAAACACCAAAGAGATGAGTTTAAAGGAAAAACCTTTAAGGAACTTTTAGATCAAC

TACACCGTCTTGATTTGAAATACCGTCATGTGGAAGAAGATGGGTTGATTTTTGAACCGA

CTCAAGTGATCAAATCAAACGCTTTTGGGTATGTGGTGCCTCATGGAGATCATTATCATA

TTATCCCAAGAAGTCAGTTATCACCTCTTGAAATGGAATTAGCAGATCGATACTTAGCCG

GCCAAACTGAGGACAATGACTCAGGTTCAGATCACTCAAAACCATCAGATAAAGAAGTGA

CACATACCTTTCTTGGTCATCGCATCAAAGCTTACGGAAAAGGCTTAGATGGTAAACCAT

ATGATACGAGTGATGCTTATGTTTTTAGTAAAGAATCCATTCATTCAGTGGATAAATCAG

GAGTTACAGCTAAACACGGAGATCATTTCCACTATATAGGATTTGGAGAACTTGAACAAT

ATGAGTTGGATGAGGTCGCTAACTGGGTGAAAGCAAAAGGTCAAGCTGATGAGCTTGCTG

CTGCTTTGGATCAGGAACAAGGCAAAGAAAAACCACTCTTTGACACTAAAAAAGTGAGTC

GCAAAGTAACAAAAGATGGTAAAGTGGGCTATATGATGCCAAAAGATGGCAAGAACTATT

TCTATGCTCGTGATCAACTTGATTTGACTCAGATTGCCTTTGCCGAACAAGAACTAATGC

TTAAAGATAAGAAGCATTACCGTTATGACATTGTTGACACAGGTATTGAGCCACGACTTG

CTGTAGATGTGTCAAGTCTGCCGATGCATGCTGGTAATGCTACTTACGATACTGGAAGTT

CGTTTGTTATCCCACATATTGATCATATCCATGTCGTTCCGTATTCATGGTTGACGCGCG

ATCAGATTGCAACAATCAAGTATGTGATGCAACACCCCGAAGTTCGTCCGGATGTATGGT

CTAAGCCAGGGCATGAAGAGTCAGGTTCGGTCATTCCAAATGTTACGCCTCTTGATAAAC

GTGCTGGTATGCCAAACTGGCAAATTATCCACTCTGCTGAAGAAGTTCAAAAAGCCCTAG

CAGAAGGTCGTTTTGCAACACCAGACGGCTATATTTTCGATCCACGAGATGTTTTGGCCA

AAGAAACTTTTGTATGGAAAGATGGCTCCTTTAGCATCCCAAGAGCAGATGGCAGTTCAT

TGAGAACCATTAATAAATCTGATCTATCCCAAGCTGAGTGGCAACAAGCTCAAGAGTTAT

TGGCAAAGAAAAACGCTGGTGATGCTACTGATACGGATAAACCCAAAGAAAAGCAACAGG

CAGATAAGAGCAATGAAAACCAACAGCCAAGTGAAGCCAGTAAAGAAGAAGAAAAAGAAA

AAGAATCAGATAACTTTATAGACAGTTTACCAGACTATGGTCTAGATAGAGCAACCCTAG

AAGATCATATCAATCAATTAGCACAAAAAGCTAATATTGATCCTAAGTATCTCATTTTCC

AACCCGAAGGTGTCCAATTTTATAATAAAAATGGTGAATTGGTAACTTATGATATCAAGA

CACTTCAACAAATAAACCCTTAACCAAAAGAAGATCTCATTGTTAAAGCACTGCTTTGTC

AAAGTAAGTTACGGTGATTTTGAAGTCATTCTATGTAACGAGTAGTGATAAAAGTTGGAT

AATAGTGGTTTTCTTTTGCAAAGAAATGGTATCCATGTTAGAATAATAAAAAAAAAGAGG

AGGATTCTAATCATGTCAGAAGAAAAATTAAAATCAAAAATTGAGCAAGCATCTGGTGGC

CTAAAAGAAGGTGCTGGTAAGTTGACAGGTGATAAAGAGCTAGAAGCAAAAGGATTTGTT

GAAAAAACAATTGCTAAAGGCAAAGAACTTGCAGACGATGCAAAAGAAGCGGTTGAAGGT

GCTGTAGATGCCGTTAAGGAAAAATTAAAATAATTAAAAAACCAGCTGTAAGCTGGTTTT

TTTAATGGTGTGATTTTGTTAGAAAAAGACTCAATAGGTAATGTTAGGTATGCTTGAACA

GTTGAGTACGATTCTCTTCAATCATATAGCACGCGTCACAAAGTTTTTCTAACATATCAA

AATCATGACTAATAATGATGAGACCTAAATTTCTATCCTTTACAATCTCCAACAGGCTTT

TCCATACACTAGCTTGTGTAATGCTATCCAACATAGTAGTCATTTCATCTGCAATAAGGT

ATTTCGTCTCTGGATGTAATGAACGCACAATCGAAAAGCGTTGCAATTCCCCTCCCGAGA

GTTCACTAGGACGACGCTTTAGCCATTTTTCTTGAATGCCAAAAGCATCTCGCAAATCTT

GACTTGGATAGTAGGCTTCTTCCAAACTTTTTTTCATAGGCCATAAAGGATTCATGGTTT

GTTCAGGATGTTGTTGGATAAGTTGTACAGGTCTAAATGCTTTGTTAGGCAAATGACTGC

CATCAACTAATACTTCACCAGATTTAGGCTGCAAAAAACCGGCAAGCACTCTGGATAAAC

TCGTTTTTCCACACCCACTTTGTCCAAATATCCCTAAGATTTGACCAGGTGCTACCTCTA

AATCAATCTCCTTAAAAAGCCATTGATCTTTTTTATGATAAAAGCCAAGCTTTTTAGCCT

CTAAGGTCATGAGTAACTCCTTTCAAAAAGTCTTGCTGAGGGAGTGTGCGCCATAAACGT

CTCGCAAATTCCGTTTGTAATTGCTCTCCACCTCCGCTAAAAAAGCTAGCTGGGGCTGTT

TCAATAGCTTTTCCCTCTTTAAAAATAGTTATACGATCCGCAATTTGACTAGCTGCTACA

ATATCATGAGTGATAAAGATGACGCTTATTCCTTTATCTGCAAAAGAGCGTAGTTGATCT

AAAACCATTTGCAGAGCATCTGGATGTAATCCAGGGGTGGGCTCATCCGCAATAATCAAA

GAAACCGTATCACTAATACACGTTGTAAACAAAACACGTCGGAGCATTCCGCCAGAAAGT

TGGAAAGGATACAAGTCACCATCACTTTCTTTTAAACCAAACTGATGAAATAATCCTTCT

TGAGTAGCCTTAGCATTTTCTGAGATACCCAAGCGCACCTGATGCTTGACTTTCATAGAT

GGATCTAAATAATTAACGGATTGTGGAATCAACGTCATTTCTTTTCCTCGCAACTGTTTA

ATGCGTTTAGAAGTTAGTGATTGACCACGATAAATCATATCTCCTGTTACAGCTGCATTT

TTAGGAAGAATATCCATAATAGCATGTGCTAATAAACTCTTGCCTGAACCACTAGCACCT

ATAATAGCTAATAACTCACCTTTTTTAACTTCTAAATTCAGCGCTTGGATCGGTGTGGAT

TGAAATGGTTTTAAAAATCTTCCGTATTGAGTGAAGGTGATGGAGAGGTCTTTAATGCTT

AATAATGTTTCTGTCATATCTCTACCTCCTAAAAATGATCCGTTTGAGGGTAAAAGAGTT

TCTTTAAAGATTCTCCGATAGTATCAAAGGCATTGACAACCAAAATAAGATAAAGGCCTG

GAAAAATCACCAACCACCAATTGCCAAGAGAGATATGCTTAGCTGCCTCTGACAAAATGA

TACCAACCGAAGGTTGTTCGGCAGAAAGGCCAAATCCTAAGAAAGTCATGGATGCTTCAT

GCAAGATGACGTGCGGAAATAAGAGGATAAACCCAATGAAAATTTGAGAAGCAATCAAAG

GCAGGATATGATGCCTCACAATATAATAAGGCGTTTTTCCCATGCTTTTAGAGAGCTGGA

CAAAGGCTTTATTCTTTAGATCATAGACTTCATTGCGGATAAGCCTTGCTAGAGAGGGCC

AATGGGTAACAGCTGTTGCAATGATAACCCCTTGAGCCCCTTTCCCAACAACAAAAGAAA

TGAGAATCATAAAAATCAAATGAGGCATACCAATAAACAAATCAACCAACCAGGCTATTA

TTTTATCAATAAAGCTATTTCCTAGACCTGCAAGCACTCCAAAAACGGTCGCAAGAAAGA

CTCCCATAAGGGCACCTAATAAGCCGACTTGTAAAGAGAAATAAAGCCCTTTAATCGTTC

TGACAAACATATCCCTACCTAAACCATCTGTTCCAAAAAGATGGTTTAATGAAGGAGCGA

GGTTGCGTAAAGCTGCATTGGTTTCCAAAGGCGTCCTATAGAAATAAAGATTTAAGGCTA

GAATACTAAGAATGAGAGAAATGGCGATACCTAGTTGCCATAAAACCATCGTTCGACGTT

TCAATATCATACTTTTCTCCTTAACTGTGGATTGATGATGCTATTTAAAATGTCCGCAAT

AAGATTGCCCGCAAAAACAAATAATGTTCCTATCATCACGATAGCTAGAAGTAGCGGTGT

ATCACTTTTAAGTCCTGCTTCAGTGAGGGTAGACCCTAAGCCAGGATATGAGAAAACTTG

CTCAGCAAGAACAGATCCTCCAAACAATTCTCCAAAATAGGAAAAATGCAGTGTAATAGC

TGGTACGATAGCATTTCTAAGGCAATGATGTTTAAAAATTTGCCACTGTGTTTCCCCACG

CGCTCTGGCAAATAAGACATATTCACTAGAAAGCACCGACATCATTTTAGTTCTCGTATG

AAGAGTGACATTGGCAATGCCTAGAATACTTAGCGTGAAAACAGGTAACATAAGGTGCTT

AATCCGATCAGCTAACGTAATATCTTGACTCAAAGTGCCTATCGGGGAAGAAATACCAAT

CGGGAACCACCCCAGCTGGACAGAAAAGATTAATAAAAAAATGAGGCCAATCCAAAACGT

TGGTACTGATATCTGAAGGTAAGAAAACCACCTGACAACTCGGTCAAGTAATTTCCCTTG

ATGGAAAGCTGATAACGTTCCTAAGATAAATCCAATAAGACCCGATAAGATCCAAGAGAG

TCCCATAAGTATGAAAGAAGCACCTGCCCGTGATCTAATAATATCACTAACAGGTTGCCG

ATAAACGAGCGAGGTCCCTAAATGTCCCTGTATCACATTTTTCAACCAAATAAAATATTG

GACTAGAGCTGGCTTATCCAAGCCATAGTGGTGAGCAATCGCTTTGTACTGAGCAGGGGT

TAGTGATGTGTCATAATTGACACTTGCCATGACGGGATCTACTGGAGATTGTTTTAACAA

AACGAAGGTCAAAACAGATACCCCAAAAATAAGTGTGATACATCTGATGATTTTCCAGAT

GATAATAATGGTAGTACGTTTCACAATGAAATCTCCTTAATCGATAAGAAAAGGAGCCGA

GGAAACGATCAGCACAGTTTTGTGTATCTTGTTTTCTCAGACTCCAAACTTAAAGTCGCT

GTATTAACTAAACGCGTTACTTAGTTGATTCATCCCAAGTCCACTCGGCAATGTTAGTCA

ATAATGACCAATCATGACCATGACTGTGGACGCCTTGTTTACCTACATTGATACGTTTAT

CACCAATATAAGTATGGTTAAGGCTCACCAACCATACATTTGGCAAATCTCCAAGAGTAG

AAGCACCTGTTTTGCCATCCCACTGCGCTAACTTCCAATATTCGTTAGCTTTGTCAAGGT

CAGAAGATGTCATTGCTTTGTCAAGGTACTTAGTCACGGTAGGATTGTTATAAAACGTAA

TATTGGTCCAACCTTTACCTGCTAGGCTTGGATGATGTGATTCATAAAATTGCTGCGCGT

GATGACGTCCTCCGGCATAAAGTAAGGCTGAGTCATGTGACTTCGTTGCCATTTCATCCC

AGTTACTAGCTTTGAGTTTAATAGTAATCCCTAGGGTTTTGGCTTGCTCTGCTACTTCAA

CGGCTAAGTTCGCTCGCAATTGATCATTAGTAGGGTAGTACAGATCAAACGCTGCATCAA

GGTCACCTTTTTTACGGCTACCGTCTGCTTGTTCTTTCCATCCCGCTTTTGTCAACAATT

GCTTAGCTTTAGCTACTTTATTATCTTTAATGGCTGTTTTTGGATTCCAAAATGGTGTTT

TATCAATAATTGAATAAGCTGGTTTACCATAACCATTTAAAACCGTATCGAGAACTTTTT

GTCTATTTAAACCAATAGTCAAGGCTTTGCGGATTGCTGGATCACTAGTGACATCATTTC

CTACAGGATAACCATCAGGAGAATCAGTGATGACGCCTTTTTTCACATAAGGTAATGATA

AGCCGCGCACATCATTTGATGGAATGTCAAGGAGGCGAGTGCCTTTGACTTTCTTACTAG

CAAGTTCTGGTGTCGCGTAGATCATATCAACATCACCAGATTCTAAAGCAGCTAGTGCTG

TGTTTTCATCAAGTAAGACCCAAGTCCACTTTTTAAAGTATGGTTTTTTCCCATGCCAAT

AAGGGTTACGAACAAAAATAGCTTGTTCTCCAGCCTTATATTCTTTTACCATGTAAGGTC

CTGAACCGATAGGATTGCTCTTATACTTATCATTGTAATGTTTTTTAGGGACGATTGGGA

TTTCAGTCAACTGTGCTGTAAATGTCGAATGCGCCTCAGTCAAATGGATATTGACCTGAT

TTTTCCCAACTACTTCAACGTTCTTGATGAAGGTTAGATCCCAAGCTTTTCCATCTGCTT

TCAACATATCATAAGTAAACTTAACATCATCAGCAGTAACAGGCTCACCATTTGAGAATT

TAAAATCATCATGCAAATCAAACGACCAAGTCAGCCCATCTTCAGAGAGATGGTATGTTT

TAGCAAGCTCTCCTTTTATATCTAGTTCAGGAGAACGTTTCAATAGAGTGCTATGAGTGA

TATTCCCTTCATTGTGGACTCCATAACGGTCCTTTGGATCGAATTCATGAGGGAGCTTTG

CCCCCATAGAAACGACAATTTCGTCTTTTGGACGTTGTTTGCGCTGACGTTCTTTTGTTT

GAGGCTTTTGTTGTTGACATGCAACTAAAATAAGCCCAGTCAAAAATAACGTGATAATAG

AGAAGTATTTTAGGTATTTTGACACAATCAAATCTCCTTATTTCTAATAATAAATAATAA

TAGTAACGACTCCATCTCATTATAGCAATAAACAAATAGGAGGGCAATTGTTTAACTGGT

TAATTAGAATTTTTGTTATTTTCAGGAAGATACTCAGGTTTTTTAATAAGTTGATGTATT

AACAAAAGATAAAGAGGTTTCATGACTTATTTTAGATTAAAAAAAGAATTAAACTGGTCA

CTAAGAAGTAAAGGAATTGTAGTTTACGAAGGGAATCAAAAACTTCAGATAGCCATTAAT

ACAGGTAAAAATAACAGTGAAAAAGTCTTTTTTGATACTTTTTTTGAGGTGGTTCGGGCG

GCAGCGACTTTCTCCACAGCAAAAAACATCTGTTTTGGTCTTGGCTTACTTCGTTCGCAG

AAATAGGAAATCGGGTATCACATTAGCAGGGTAGTTCATGCTTTTTGAGCATCACACTGG

GTCTGTCTACGTTCTAAAAGCTTTCTTCATTTTTGTATCATACCCCCCTACAAGTAGAAA

AAGCATAATAAAGAAAATTTATAAACAAAAATGGCTAGTATTATCAAGGAATTTATCAAT

TTTTGTTTTTTAAAATTAATATAGAAAGTATAGCATTCTAGAACAAATTTAGAACAAAAC

CTAAGTGATATAAAAAAAGACATACTTACTTTTTAGAGGATTCAATCTATTTGGAATAGC

TTATTGTATAAAAGTTATATTGAAAAATTGAATTAGGAGTCAATTTCTATATCTAAATGC

CCAATTTTATCTATATTGAAAGATTTATTGTCTTTGTATACTTTAAAAATACTTTCTTTA

TCTCTATATCCTACATAAAAAAGATCGAGAGAATATTTATCTGAATTATCATTTGTATGA

AAAACTAATTTACCACTTTTGTAGCTAGAACCAGAAGAATATAATTGATGTTGTGCTATT

AAAAATTTTCTAACCTTTAGATCAATTTCTTGAGCTGTAACTGTTGTTTTGTTAGTTGAT

ATTTCGTTGTAAGTAACAGAGATCTGCTTTCCATTAATCCATAAATTAACAGGGATATTT

TTAGATATTGGTTCTTCTTGATAAGGCGTCATTCCACCATAGGTATATGCTATGACTTTT

CCTTCTGACAAGTAGTTCCAATCAAATGGGACGGAGAACACAGCTATTTTATCTCCAGCT

TTAAAATCATTGGCTGCATATTCGTCCATTTCGGAGTTAATAAAAAAATCTCTAGCATCT

CTAACATCAAGTTTCTTAGTCACTAAGTTATGACTGGTTTTAAAATCAATTACTGTATCT

GAATATTCATATACAATCGTACTATAGATATTCCTTAGAAGGGAATTATTATCTACTTCT

AATCCAAACACAGGACGAGAAATTATTGCAATGAATATTGAAGTAAAAGAAAAAATAAGT

TTTGTTTTTTTCATAAATAGCCTCTTTTCAGGAGTTATTATTTTATTATGTTTTAATATA

ACACTTTCAAAAGAAATAATCATTAACAATTTTGTAGATATACTTGCTGACTATCAAATG

AAAATTGAGACATGATACTAAAAGTGGATTGACGCTTTTAATTAAGCCGATTAGTTATGA

AACAATCAAAAAAGCCTTTAAACAAAGGCTTTCAGTCTGAATAGCCCGGATTACCCGTGG

TTATTTTAGTTTTGCACTCTAATTAGTTGATCGTATTCATATAATGACTTAGCTGCTTTT

GATTTGTTAAGACAATGGTTTTATGAGAGTATTTTTGGACAACGGATTGGTATTTTTTTA

ATTGGTTTCTTGAACGTCCATCTAGTAGAATCCATTTCACAAACGCAACGTCAAATTTTT

CCTGACAGTTGTCAGCCATATCAGGACGCGTTTTTCCCCTATAGTTTAAATAACGCTTAA

ATGCTCGATAGACACAATGAAACCGACTGAAATTAACATAGATAATGTAATCAGCTTCAC

TCATGCGCTCTTCATAGAGACAATTGGCATAATTCCCCTCAATAATCCAATCTTGTTTGA

GCAGGCATGTTGATAAATCAGCTATCATGTCATGATCTGACCGTTCTTGCCAGTTCGATG

AGAAGTGTAGTTGGTCTAAATGGAATACCTCACAATGGTAATGTTGGCCTAAAAATCGTG

CTAAGGTTGACTTTCCAGAGCCACTATGTCCAATGATGGCAATTTTCAATGGATACCTCC

TAAAAATGACGTATGAAATGACTATAAAGGGTATGAAATGTGTTTAGATTAGTTTCTTGA

TGAGATGCGTGTCATCATAAAATTGATAACCCTGTTTAGGATAAAACCGATAAGACTCAA

ACCATTGTTCTTTAGGTTGACCTAAGTGGATACGAACATGACTCTTGCCGATTTTCCTTA

GATAATCTTCTGCAAACGTCAGAAGTTGACTGCCAATGCCTTGATGTTTTAAGCTTGGTT

TGATGAAGAGACGGTGCAAGAAGACCTCATTTGTTTTGGGAATAGAAGAATAAGCGATTG

AGCCAATAACACGACTTTCTTCATTGACCGCTAGCCAAAAACAATCCCCTTTTTCCAAAT

AACTTGTTTCAATTGTCAAAAGATCCGGGTTAATGCTGGGAATTCTCCTTAAGGCATTTT

TTGCTTCCAAGACCATAAAAATGAGGTCATCTCTGTAATTTCCTTTGTACTCAATTATTT

TCATAACAGTCTCCACCTTAATCTTAAGCGCTTTAATTGGTATCATACCATAAAAAAATT

ACTGCGGGGATAAATGATGGAACAAATCCTCTTCTGACAAGCAAAAAAAGCCTTTAAACA

AAGGTTTTTTCATGTATAGTTGTATACCCCCTACAGGGCTCGAACCTGTGACCCATAGAT

TAAGAGTCTACTGCTCTACCAACTGAGCTAAGGAGGCAAGAATGCTGTATTGGCACCGGT

GATCCACGTTTTTGTATTGAGCCCGCGCAGTATAAGCAGGTGGGTAACGCGCTCTTTCTT

GGAAGTTGCGTCCGCGTGAAACGGCTTGCAGACTAGAAAGAGTCTTTGTTTCCCGAAAAC

ACAAAGAAATAGTCGGTCAACACTTAGATGTGGATTCGTATGCCACAGCAATGTTTCTGT

ACTTTTATAATACCACTTTTTAAAATTTTTTCAAGCAAAATTATCGATTTTTGAAATCGG

TTGCGATTTTTAAAACTGGCAGTCAGTCTAAGAGCTTATCAATACGCTCTTTATTTGAAC

CAAGTGCGCGTTCGTATTTACCGGTTTCATTGGCTTCAAAATAATGATGTCCTACTAATT

TATCTGGTAGGTATTGTTGTTTGACCCATTTTTCAGGGTAGGCGTGTGGGTAGAGGTAAT

CTTTGGCATTGCCTAGGTCCTTGCTGCCAGCGTAATGCCCGTCTCTAAGATGCCTTGGAA

TGGGGAGGTTACCTGATGTTCTCAAATCAGCTAAAGCTGCATCCATAGCTAAATAAGCTG

AATTTGATTTAGGAGATAGAGCCAAATCAATGACCACGTTAGCGATAAGAATACGAGCTT

CTGGAAAACCAATGCGTTGAGCGGCATCTAGTGCGGTGACGGTATGAACTTGGGCGTCAG

GGTTGGCTAAGCCAATATCTTCGTAAGCAATAATGGTCAATCGTCTTGCTAAACTAGGCA

AGTCTCCAGCTTCTACTAAACGAGCGGCGTAGTGCAGGCTAGCATTGACATCAGAACCTC

TAATGGATTTTTGTAAGGCAGATAAAACATCATAATGCCCGTCCCCGTTTTTATCCATGG

TAATATAACTACGCTGCAGACTATTTTCCATTGTCTCTAGACTAATATGACGGCTACTAT

CTTCATTAGGAGAAGTAGACATGACTGCTAGATCCAAGGAATTATAAGCAGATCGTAGGT

CTCCATTTGTGGCGGTAACAATGAAATCTAGCGCCTCATCATCAATTGTCACTAAAAAAG

GAAATCCCCGTTCTTTGTCGGAAATTGCTAGCTGAATGGCTGTTTTAATATCCTCGTTAG

AAAGTGGTTCCAATTCAAAAATCTGGACCCTGCTTCTGATAGCGGGTGTCACTGAAAAGA

AGGGATTCTCAGTGGTTGCGCCAATCATGATAATGGTGCCGTTCTCAAGTAAGGGTAAAA

GAAAATCTTGTTTTGTCTTGTCAAGGCGATGAATCTCGTCTAGTAATAATACCAAGCCAC

CAGAAAATTTAGCTTCTTCCGCAATTTCTTGGAGACGTTTTTTGCTGTCAATGGTGGCAT

TAAAGGTCCGAAAGGCATATCTAGTAGTACCAGCAATAGCAGAAGCAATGGAAGTTTTAC

CGATTCCTGGAGGGCCATAGAGGATCATGGACGATAGTCTATTTGCTTCCACCATGCGAC

GGATGATTTTGCCTTCGCCAACAAGGTGTTTTTGTCCGATGACCTCTGAAATGGTTTTGG

GGCGCATTCTGAGCGCTAAATGATCAGGCATGTAACTGTCCTCCTTTCTTTTGGGGGATG

AGATTAACGGGGAATGGCTGCGATGAAGTTGGCGATCATGGTCATGCCATCAGGAGTTCC

GATGCTTTCTGGGTGAAATTGTAGCCCAAAAAGTGGCAGGGTGTGGTGTTCAAAGGCCAT

GATTTCTTGATCGTCACAGTCTCTAGCAGTTACGCTAAAACCTTTTGGTAACTGATCCAC

AACGATAGAATGGTAACGCATGACGGTGATCTCTTGTGGCAGGGAGCGAAAAAGACTAGC

AGGGCCTTGCGTTTCAATGGTGCTTTGTCTCCCATGCATGACGCGTTTTGCCAAGCGTAA

GGTTCCTCCTAGAGTTTCAGCGATAGCTTGGTGTCCCAGACACACTCCTAAGATAGGTTT

TGTTTGGTAAAAGTCTTGAATGAGTTTTGGCATTTGGTTGGCTTCCTTGGGCCAACCAGG

ACCAGGTGAAAAGACTAGAGCGTTAGCTTTTTTGGCCATGTCATATAAGTTTGGGTCTTG

GTTATACAAGACAATCGTCTCGTCAAATTCACTTAGATATTGGGAGAGGTTGTAGGTAAA

TGAATCGTAATTATCAATTAAGAGTATCATAGGTTCCTTAATAACAAAAGATGAGTGGCA

GTTTAGGCTTGACTCTTAGCTAAGAGGGCATCAAGGTGAGTAAGCTCAAGGTTGAGAGGA

TAGAGGCCACGGACAGCATTTCCTCCAAAAATAGCGCTAGCCTCTTTTAAATCAGCTAGT

GTCACCTCTTTTTCCTGAGCTTGACCAGTAGCTAGCAGCTCTTGGCGAAAAAGTCCTGGT

AAAACACCCACCGCCACTGGTGGTGTGTAGAGGGTCCGACCCAGCTGGATAAAAAGATTG

CCGATGGACGTTTCTAATAATTGCCCCGCTTGGTTATAGAATAGCTGTTCATAAGATTTT

TGCTCAATATGAGGCCGGTAGCTGGTCTTAAAGTAGGTAAAAGGCGAAGCTGTCACGTCC

TTTTTCTGTAAAGAGAGTTGAGCTGTCAGAAAATCGGCTGACAAAGGTTCCAAGGGTTGA

TCAGAGAGGCTAATCTTTCCATCTTTGGATAAACGAATCATCAAACGGTAGGCAGCATTA

TTCTTATTTTCCAAATAGGTTGACAGTTGTTTCTGCAAAGCTTTCTCATCGTAAGGGTAA

GCAAAATAAGTGGCTGCTTCTTTAAGGCGATTGAGGTGTTGCTCAAGGAAGGCTATTTTT

TGGTGTTCCACCTTGGCGGTTGTTTTGAGGTCAAAGATTTGTTTGTGCCGATAGAGAAAA

GTTGTTTTTTGATGGACTTCTTCGTATTCGTCTTCCCACTTGCTTTGCCAAGTGATACCG

CCTCCGACACCATAAGTAGCTTGATTATGACTGAGCTGGATGGTTCTAATGGGAACATTG

AAGAAACGGCGACCGTCAGGTAAACAAATGCCAATACTTCCACAATAAATCCCCCTTGGG

TTTGGCTCCAGAGATGTGATAATCGCCATGGTGGAGACTTTAGGAGCCCCTGTGATAGAG

CCACAAGGAAAGAGAGCTGTGAGAATGTCTATCAGGTCACAGTCCGCTTTGAGGTCACCA

ACAATGGTTGAGGTCATTTGCCAGACGGTAGAGTATTGTTCCACCTCACAGAGCCTATCG

ACACAAACACTGCCTGTTTGGCAAATTTTTCCCATGTCATTGCGGAGCAAGTCAACAATC

ATCATGTTTTCAGAGCGATTTTTCCTGTCAGTTTGGAGCCAATCATGTTCTTGTTGATCC

AGCCAGGTGTTAACCCCGCGCTTAGTGGTACCCTTCATAGGTCTGGTGATGAGTTGATTG

CCTTCCTGTTTAAAGAACAATTCAGGACTAGCTGAAATAACAGCAAAGTCATCATGGGCG

ATATAGGCATTGTAGCCAGCAGCCTGTTCAACGACGAGTTTATTGTAAATGGCTAAGCTG

TCAGCTGCATTGAGTTCCTGAGTTAATTGAAGGGTGTAGTTAACCTGATAAGTATTGCCC

TGTCGCATCTCATGGTGAATGGTTTCAATGGCTTTTTGATAGGCTTCTTTTTGAGTGGCG

CTGACCCATTGGTTAGGGATAGTAATGCTGTCATAATCAAGAGGTAGGTCTTCTTTCTGA

CAGGTTTTGTGCACCGTAAAATAGGCAAGATACTCGTTGCCTAATCGGTCATTATGGGTT

TGTAAGGCGTTGTCAAAAAAAGCAGCTGCTTCGTAACTGAGGTAGCCCACCACGTAGTAC

CCCAGTTCTTGGTAGTGCTGAACCTGTTGAATGACTGGGCCGACCTGATCTAAGGACTTG

GCTACCAACTCTAGTAAGGTTTTTTCAAACAAGAAGCGGTGCCCAAGCTCCTTGAAATCA

ATAATGGTTTTTCTATGCATACCCAAATTATATCATAAAAGCGTTTGCCAAGCCTAATGA

GAACAGGCAACAACTGTCTTTTGACCACGTAAAAAGACTGAACTTTCTTAGTGAAAGTTC

AGTCTTGATTGACTACAATATACTCTTATCGTAAGGCAAGCAGCAGTGAATGTTTAGGTA

GGTTAAATCTCCGACGACAATAGCCTAGTCTTTTACTTTATTGCTCCAGAAGCTTCCTGC

TAAGAAGAGGGTTCCAAGGAAAAGGCTGATGTAGCTAGTTAACAACACACCTTTTGACTG

ACCATCACCAGTTTGAGGGAGGGTTACTAAGCCGTTCTGGCCCTTGGTTGGTCCAGTAGT

AAATCCCTTGGTTTGATAAGTCACTGGAATGGCAACGCCTTGTTTGTTTTCAGATTGAGT

TGAGAGGTTATGCTCATGCGCAGGAGTTTCTGGCAATCCTTTTTTGCCAGCCGTCAGGAT

ATGGAAACGATTTGCTTTTTCAAAGAGCTGTCCCAGTTGTAACAGGAGACTATCGTTATG

AGCTCCGCTATTCACCAAAATGCCAAGAGGTAGACCGCTTTTCGTCACATGGGTTGGTAA

GGACAAGGATGGTGTTCCCGTCAGGTTAGCAAGCTGGGTAAATGGTGTTAGCGTCCATGC

AGGTAACCATTGCCGGTAAATAAGGTCCAATTTTTCTTCTTTGCTCAAGCCTGACATGTC

ACTGAGCTGTGCTACTAGGTCTTTCGGAATGTGGTGGTAATCGGCAGCAGGGGCGGGATA

GGCTGTTGTCGGTGTTAAGAAGATGGGGTATTTTTGGTAGAACTGGTTTAATTGCTCGGT

CATGGCTGCGATACCCTCCCAGGCTTTGTTGACATCCTCTTTGGTCAAGTCTTTCCCAGT

TTGGTAGAGCGCCCATGATAGGAGTTCGACATCCTCTTTTTGAAGGGGGCGTTTCAAGGT

TTGTTGTGCCATAAAATTGATAGATGGCGCTGCAGAAGCAGCAATAGTGTAATAGTATTG

CATCATGAGCTTACCATCAACGGGGTAAGGCACTTCTACGGTTTGATAACCTTGCTCCTG

TAAAAAGGCTACAGCTTCTTTGATCGCAGCAATAGCTTCCTCACTAATTGGTGTTCCAGC

TGGTGTCTGGGTGCTGTAAGCAATGGGGATACTTGTGTCTAAACGCTGTGGGTTTTGTTG

CGCTTTAGCCTTGTCTTTTAAGAGGAATTGGAAGAGCTTTTCAGTGTCTTCCATGCTCTT

AGTTAGAGCAAAATGGCTAACATTGCTACGTTCAGAAGTCGGATTTCCCTCTAAAATGCC

ACGTGTTGGGTGTAGGCCAATCAAGCCACTCCAAGAAGCTGGAATGCGGGTTGAGCCCCC

ACCATCAGAAGCACTGGCTAGACTGACTTGTCCTGAAGCCACAGCAGCTGCAGAGCCACC

AGAGGACCCGCCAGGATTTTGGTCAAGCTGCCATGGGTTATGTGTATTTCCGTAAAGATT

GGAATTCGTCACATTAATCCATCCCATTTCAGGGAAAGAGGACTGCCCTACCACGATAAA

ACCAGCCTTTTGGAGTTGTTTGACAAAGGCGCTGGTAGATGAGCTGGTTTTGTCTTTCAA

AAAGGCAAGCCCGTTGGTGTTGCTACTTCCAGCAACGGTGTGTCCTAACCCTTTAACCAG

AATAGGAACCTTGTAAAAGGGTTGCCCCTCATCTGTCATTTGCTCGCTCTCTTGTCGAGC

CAATGGTTCACGCAGAGAAATGACATTATTGAGCTCAGGGTTAGTTTCTTTGATGGTTTC

CAAGGCAAAATCTAGCAGCTGTTGGCCTGTGACCCGTTGCTGGCGAGCCCACTCAGCTAA

TTGGCTCGCACTGGCTGCCTTATAGTCTTCTAAGGTAATCGGAGATTCTTGGGCGGCAGG

AGTGGCCTCTGTTGTTAAAGGGTGTAAAGGAGACGAAGCCTCTTGCGTTGAGATTGGATG

GGTCTGGTCTGCAGGTGAAGTGACAGATTCCATGGCTGTCGGCTTACTAGCTTCCACTTG

GGCCGTTAGTGGTTGATGGTTGGTATTAACCTCATCCGCATAAGTAGTCATAGCTCCAGC

TTGTAGGCTGGCAGTCATTAATAACAAGGCAAGAAGCATCTGTCTTGTCTGAGAAGGGTA

GCGTCTAGAATATCTCATTCTTTTTCTCCTAACATCATAATCGCTTATTAGTTTAACATA

ATGGGAGGAAAGTAAGGGGTAAAACGCTTACTAAAATACAGAAGGCCCATCAGTCAGCAT

TTGCCAGTGAAAGTTAGCTCAAATTATGCTACAATGGAAGCAAATAATCGTTAGGAAAAA

GGACAATTAGATGGCAAAATACGGCTTTTTATCAATATTAGAAGAAGAAATGGATAAGCA

TTTTCAGTATGATTATGCTATGGATTGGGACAAGAAAAATCATGCGGTTGAAGTGACTTT

TGTGTTAGAAGCTCAAAATAAAGAGGCCATAAAAACCATTGACGATAGTGGCGAAGTGAC

CCAAGATGATATTGTCTTTGAGGACTATGTGCTCTTTTACAACCCTGCCAAGTCTCAATT

TGATGCAGCAGATTATTTGGTTACTATTCCTTTTGACGCTAAAAAGGGCTTTTCTCGTGA

ATTCTTAGCTTACTTTGCGCAATTTCTCAACGATGTTGCTATTGAAGGGCACAGCGATTT

GATGGATTTCTTAGCAGATGACAGCAAGGTTGATTTCGGCTTGGAATGGAATGCGCAAGC

TTTTGAAGAAGGTCAACAAGGACTAGAAGAAGCAGCAAGTTATCCTTACCCAAGGTACTA

AGGTGACACACGAGATGGTGCTAACACCATTAGGACAATAACCAAAAGGTCAAAATAGAA

AGGACATACTTATGGAAACGTGGCAAGAAGTGACGGTTCATGTGCATCGTGATGCGCAAG

AGGCTGTTTCACATGTGTTGATTGAGACAGGCAGTCAAGGGGTTGCTATCGCGGATAGCG

CAGACTATATTGGGCAAAAGGACCGCTTTGGGGAGTTATACCCAGATGTGGAGCAGTCTG

ACATGATAGCTATTACAGCCTATTACCCAAGTAGCACTAATTTGGCGGATGTGACCGTGA

CCATTAATGAACAGTTAGCGGAGTTAGCTAGTTTTGGGTTACAGGTTGGTCAGGTTACTG

TGGATAGTCAGGAATTGGCTGAGGAAGACTGGGCAGACAATTGGAAAAAATACTATGAAC

CAGCTCGTATCACGCATGATTTAACCATTGTCCCTAGTTGGACAGATTATGACGCTTCTG

CTGGGGAAAAAGTGATTAAATTAGATCCTGGAATGGCATTTGGGACAGGGACACATCCCA

CAACTAAGATGAGCCTTTTTGCTCTAGAACAAATCCTTCGTGGTGGTGAGACAGTTATCG

ATGTAGGGACCGGATCAGGTGTCCTCTCGATTGCCAGTTCCTTACTAGGTGCTAAAACCA

TCTACGCTTATGATTTAGATGATGTGGCTGTTCGAGTGGCTCAAGAAAACATTGATTTAA

ACCAAGGAACAGACAACATTCATGTGGCTGCCGGCGACCTTTTAAAAGGGGTTAGCCAAG

AAGCGGATGTGATTGTAGCCAATATCTTGGCGGATATCTTAGTCTTATTGACTGATGATG

CTTACCGTTTGGTCAAGGACCAAGGCTATCTCATTTTATCTGGTATCATTTCTGAGAAAT

TGGATATGGTTCTGGAAGCTGCCTTTAGCGCAGGCTTCTTCCTCGAGACACACATGATTC

AAGGTGAGTGGAATGCCCTTGTCTTTAAAAAAACAGATGATATCTCAGGAGTGATTGGCG

GCTAATGCAACAGTATTTTATTAAAGGCAAAGCAGAAAAAAAGGTAACAATTACCGATAA

AGATACCATCAAGCACATGTTTCAAGTGATGCGCTTGGCTGATGAGGCTGAAGTTATCTT

GGTCTTTGATGATGGGGTGAAGTATTTAGCCAAGGTGACCAATAGTATGGCTCATGAGTT

AGAGATTATTGAGGCTTTGCCAGATCAGGTGGAATTGCCTGTTGAGGTGACTATTGCTTC

TGGATTTCCTAAAGGAGATAAGTTAGACACGATTGCCCAGAAAGTGACTGAATTGGGAGC

CAGTGCGCTATGGGGCTACCCAGCAGACTGGTCAGTGGTCAAATGGGATGGCAAAAAATT

AGCTAAAAAAGAAGACAAGTTGGCTAAAATCGTTTTAGGAGCGGCTGAGCAGAGCAAACG

AAATAGGGTGCCTGAAGTTCACCTTTTTGAACATAAAGCAGAGTTTCTAAAAAGCCTTTC

TAGCTTTGATCATATCTTCATTGGTTATGAGGAAACAGCTAAAGCAGGTCAGCTAGCGAC

CTTGGCGCGTGAAGTAAAAGAAGTTAAGCCAGGGGCAAAAATCCTCTTTATCTTTGGACC

AGAAGGGGGAATTTCCCCAGCTGAAATCACTCAGTTTGAGGCAGCAGGTGTCATCAAGGT

GGGCTTAGGCCCTCGCATTATGCGAGCAGAGACAGCGCCGCTATATGCCTTAAGTGCTCT

TAGTTATGCTTTGGAATTGCAGGCATCAAAATAAGTAAGCTGTTGCTTACTTTAGAAAGA

AGAGGCTAAAGAAACGAACAGGCAGTTCACGATTGTGTCAACGGCCTTTTTTTATTGCTT

CTTGTTTTTTTGAAGAGCGTTGATGGGCTCATCACCAAAAAAGGAAATGGGCTCTTGTCC

TCTTTTTGGAGAGTTACACTACTATATTAATGAAGAAAAATGACTAACATCTGTCTAAGA

AAACGCTTTACAAATTGTTGCGAGGCTAGTATAATAAAGGTATCAGATCAATGCAAACGA

TTGCATTGCTGAGTTGTAGTCTATATCATTGTGTCACAAAAGCGCCTTAAGGGCAGCTAT

GGCTAAAAGGGGATAATAAAGGAGAGATAGAATGAAAACATCATTCAAACAACTGTTTCG

TTTCGAATTTTGGCAAAAATTTGGGAAATGTCTTATGGTCGTTATTGCTGTCATGCCAGC

GGCTGGTTTAATGATCAGTATCGGAAACTCTATTCCAATGATTAACCATGACTCAGCATT

TTTGGCATCTCTAGGCAACATTATCGCTCAGATTGGTTGGGCTGTTATTGTTAACCTTCA

CTTGCTATTTGCCTTGGCCATTGGTGGTAGTTGGGCTAAAGAAAGAGCTGGAGGGGCCTT

TGCCTCAGGTCTTGCTTTTGTCTTGATCAATCGGATAACAGGCGCTTTTTATGGCGTGTC

TAGTGCTATGCTAGCTGATCCAGAAGCAAAAATCACAAGCCTCCTTGGCACCCAAATGAT

CGTTAAAGATTATTTCACCAGTGTCTTGGAATCTCCAGCTTTAAACACAGGGGTTTTTGT

TGGGATTATTGCAGGGTTCGTAGGAGCAACGGCCTATAACAAATACTATAATTACCGTAA

ACTTCCTGAAGTTTTGACCTTCTTTAATGGGAAGCGTTTTGTACCGTTTGTCGTTATTTT

ACGTTCTATTTTTGTAGCCCTTATTTTGGTTGTTGTTTGGCCGGTGATTCAGTCTGGGAT

TAACAGTTTTGGGATGTGGATTGCCTCTTCACAAGATTCAGCTCCAATCCTAGCCCCATT

CCTATATGGTACCTTGGAACGTCTCTTGTTACCATTTGGTCTTCACCATATGTTGACGAT

CCCAATGAACTATACAGCTCTTGGTGGAACTTATGAGGTCATGACAGGTGCAGCAGCGGG

AACAAAAGTATTTGGTCAAGACCCTTTGTGGCTTGCTTGGGTAACTGACCTTGTTCACCT

TAAAGGATCAGACGCTTCAGCCTATAGTCACTTAATGGATAGTGTGACTCCAGCTCGCTT

TAAGGTAGGACAAATGATTGGAGCTACCGGAACATTGATGGGGGTTGCCTTAGCCATGTA

CCGTAATGTGGATGCTGATAAAAAACACACATACAAAATGATGTTCATCTCAGCAGCGGC

AGCGGTCTTTTTGACAGGGGTAACTGAGCCACTTGAGTACCTATTTATGTTTGCGGCGAT

GCCACTTTACCTTGTTTATGCCCTTGTGCAGGGAGCTTCATTTGCCATGGCAGACCTTGT

GAATCTCCGTGTTCACTCATTTGGAAACATTGAATTATTGACCCGTACCCCTATGGCCCT

TAAAGCAGGTCTAGGCATGGATGTGATTAACTTTGTTTGGGTCTCTATCCTATTTGCAGT

CATCATGTACTTTATTGCAGATATGATGATCAAGAAAATGCATCTCGCAACAGCTGGTCG

TTTAGGCAACTACGATGCCGATATACTGGGTGACCATAACACTCAAGCAAGACCTACCCA

AGTAGCAGATAGCAACTCTCAAGTTGTACAAATCATTAATCTCCTTGGTGGGGCAGGCAA

TATTGATGATGTTGATGCTTGTATGACGCGCTTACGAGTGACGGTCAAAGACCCCGCTAA

GATTGGCGCTGAGGACGATTGGAAAAAAGCTGGCGCTATGGGCTTGATCCAAAAAGGCAA

CGGTGTTCAAGCGGTCTATGGGCCTAAAGCAGATATTTTGAAATCAGATATTCAAGATTT

GCTGGACTCAGGGGCTGTTATTCCAGAAGTAAATATGTCACAGCTCACTAGCAAACCGAC

TCCCGCAAAAGATTTTAAACACGTGACAGAAAAAGTGCTATCAGTGGCAGACGGGATGGT

TCTCCCAATCACAGGTGTGAAAGACCAGGTGTTTGCGGCTAAGATGATGGGAGATGGGTT

TGCGGTTGAACCAACAAATGGCAATATCTATGCACCCGTAGCTGGCCTTGTGACCAGTGT

CTTTCCGACCAAGCATGCCTTTGGTTTACTGACAGATAATGGTCTTGAAGTGCTGGTGCA

CGTGGGACTTGATACCGTCGCCTTAAATGGTGTGCCTTTTTCAGTCAAAGTCTCAGAAGG

ACAACGGGTTCATGCAGGTGACCTATTAGTCGTAGCAGACCTTGCTGCTATTAAATCAGC

AGAGCGTGAAACAACCATTGTTGTTGCTTTTACCAACACAGCAGAGATCCAAGATGTGAC

CTTGACATCTTTAGGAGCTCAGCCTGCTAAGACTAAAGTAGCTACCGTAGAACTTTAACA

AAACATCACGAGATAGTGATAGGTAATAGCCATAGCTGGTTTAGGCCCTATGGCTCTTCT

TGTCTTATAAAGGAGGATTAATGACCAAGGTATTAACACTGAATACCCATTCGTGGATGC

AAGCCAACACTCTTAAAAAATTAGTTGCTTTGGCTGAGCATATTTTAGCTGAAAAATACG

ATATCATTTGCTTGCAAGAAATCAATCAGTTAATCGAGAGCGAGCTGGCCACAGATTTAC

CAAGATATCAAGCTCTTTCAGGGACACCAAGCATTCATAAAGACCATTTTGCTCTTCTTT

TGATCCATTATTTACAAAAACGAGGGCAGCACTATTATTGGTCTTGGGCTTATAACCATA

TTGGCTATGACATTTACCAGGAAGGCGTGGCAATCTTATCAAAACAACCTATTCATGTTT

CAGATATTCTCGTGTCAGCGATGGCTGATGAAACAAACTACCACACCAGACGCTCACTCA

TAGCCAAAACCACGTTAGATGGCAAAGAAGTCGCTGTTGTCAATGTGCACTTGTCATGGT

TTGACAAAGGGTTTTTAGGGGAGTGGGAGAAATTAGAAAAAGAGCTGCTCACACTAAACT

GTCCCTTGCTACTTATGGGAGATTTTAACAATCCAACGGATCAAGACGGTTATCAAGTGA

TGATGGGTAGCCCCCTAGACCTACAAGATAGCCATAAGGTTGCTGACCATGTGTTTGGTG

ATCATAGCATCGTGGCTGATATTGATGGCTGGCAAGGTAACAAAGAAGCCCTCAAGGTGG

ACCATGTCTTTACCAGCAAGGACTTCATCATCCGATCTTCTAAGATTACCTTTGAAGGCG

GAGATGCGCCAGTCGTCAGTGACCACTATGGTCTAGAAGTGACACTAGACTGGCGTTAGA

GCATGTGCTAACAAGCACACCACATAAGAGAAGCCAAGACACATAAGAAACTGCGATCGT

CTAGGGGGATTAGGAGAACACAAAGAGGTCTAACCAGCTCAAGGTAGAGCTGGTTAGACC

TCTTTAGTCATGTCAGTAACCCTAGAAAACCAGGGCTTTTTTTGTCACCAACCATCTGAG

CTTCACAGAAAGTACCACCAAGCGTATAGTTGCCTTGCTAAAGTCTGCATGGGGGTATTT

CTAGGAGTGCTTGGTGTGATCCCAAGCCCCACGAAGCACCATGGACCACCTCTTCAAAGG

CTGAGAAATCATAAGGCGTGGGGTAAGAGACCTCTGTAGTTATTGTCCTAAAAGCGCTCT

TTTTTAAAACAGCTATGATCATCTATAGACGGTCTAGCTGTTAGGCAGTGTCTGTGTGTC

GTTAGACGAGTGTCTGGAGCTGGCCTCCATGATGACCTGGGCTAACCGCGAAATGTCATC

AGGAGTCCCTCGCAGCTCAAAATCACCTAGCAGTGGAAAGCCAAAGCGTTTGGCATACTG

TTTGGCTGTCAGGCAATACTGATGGTTAAAGTTTTTGTTGCCTGAACCAATAATCCCAAG

ACAACGCTGGGCGTTGCCATGGGCAGCAATAAACTCTCCCAAAGGAGTGGTGAGGATCTC

CATCTCACCAGAGTCAACGCCATTTCCGCCTTCTAGATAAGTGGGTAGAATCGCCACAAA

CTCTTCTTTGACGGGAAAAGTCTCGTGCTTCAAATCTTTAATGTTAATCTGTTTGACATG

GTAATCATAATTGTCTGCTAAATAAAGGGATAGCCGCTTGACAAAGCTAAGGGTGTTGCC

ACTGAGGCTAATAAAAACAAGGGTAATCTGAGGCATAAGTGTCTCCTTTTTTTATCCTAC

AAGGTTTCTTCATAAGCTTACCACAATCAAGACCAACAAACAAGTAAAGAAGAAACCTAG

CGGTATCCAACCAGACAAGAAGGTACAAGCAGTCAAAAAAAGTAACATTTCAAAAAAGAG

TGACCTTAAAGAAAGGATCCGTATGCCCTCGACTTAAAAACGAGGCAGGTAAGTGCCAAA

GTGGCCCTCTGATCTCCACAAAAGAGCGATCAGTCAAAATCTAAAAGATGAACAACAAAA

GAGAACTTTACAATCGTTTTCACACTCTATTATAATACTATTATAAATAATCAGGTCTAG

CTACCATGTTATTTTATCTAAAACACACCATCTAATAAAGAAAGAGAGAACAACATATGT

TGACATCAAAGCACCATAATCTCAACAAACTGGTCTGGCGCTACGGGCTAACCTCAGCTG

CAGCCGTCCTTCTAGCCTTTGGAGGCGGGGCAAGCAGCGTTAAGGCTGAAGGTATGACGT

CTGGAGAAAGAACACAAAAAATCAATGAGATTAAAGAATTTATAAAAAAAATCCCAAAAA

CAGATCAGGAGCAATTTCGGCACCGACCATGGTATGGTGGCGACTACTTTAAAGACGAAG

ACTATCAGAAGGAACTGAATAAATTTACGGAAGATGCACTTAAAAAAATTGTAGACTTGA

TTGAAAAAGGTGCAACAAAAGGTGAAAAAGGGGATGCTGGTCCTAGAGGCGAGCGCGGCC

CACAAGGTCCAGTAGGCCCAGCAGGTAAAGCTGGTGAAAAAGGTGCTAAAGGTGATAAGG

GTGAAGCCGGTGCCCCAGGTAAAGACGGCGCCCAAGGACCTAAAGGCGACAAAGGTGAAA

CCGGTGAACGCGGTGAAAAAGGCGAAGCTGGAATCCAAGGCCCACAAGGTGAAAAAGGTG

AAACGGGCGCACAAGGCCCAGCAGGCCCACAAGGTGAGGCAGGCAAACCAGGTGAGCAAG

GCCCAGCAGGCCCACAAGGTGAAGCAGGCAAACCAGGTGAAAAAGCTCCAGAAAAGAGCC

CAGAAGTGACACCAACACCAGAAACGCCAGAGCAACCAGGCGAAAAAGCTCCAGAAAAGA

GCAAAGAGGTAACTCCAGCTCCAGAAAAACCTGCTGACAAAGAAGCTAACCAAACGCCAG

AACGCCGCAATGGCAATATGGCTAAGACACCTGTAGCCAACAACCACAGACGTCTACCAG

CAACTGGTGAGCAAGCCAACCCATTCTTTACAGCAGCAGCAGTAGCAGTGATGACAACAG

CTGGTGTCCTAGCCGTTACAAAACGCAAAGAAAACAACTAATCCTCTGAATTGAGAGACC

TTACTGATAAGCAAACATAATGGAGAGAGCCCCTTGGTTCTCTCTATTTTTGGTCGGGAG

CAGGCCTTTGGTATCATGCTCACGCCTGAGAGATCCCAAAAGATATCAAAAGCAGCTCAC

TAACCGTTTTCTAAGAAAAGGTCACACTAAGCGCTTTCTTAGCAGATTCTGCCTTTTGCT

TTTGTGTGTAAAGTGTTAAAATAGGGAAAAGAAATGCTAGATGAGGAACGAAATGGCAAA

AATAATGAACGTAACAGGAGAAGAAGTCATTGCCTTAGCGGCCACCTATATGACCAAGGC

TGATGTGGCTTTTGTGGCAAAGGCTTTAGCATATGCAACAGCGGCCCATTTCTACCAAGT

GAGAAAGTCAGGCGAACCCTATATCGTCCATCCGATTCAGGTGGCGGGGATTCTGGCTGA

TTTGCATCTGGATGCTGTGACAGTTGCCTGTGGCTTTTTACATGATGTCGTAGAAGATAC

GGATATTACCTTAGATGAGATCGAAGCAGACTTTGGCCAAGATGCTCGTGATATCGTTGA

TGGTGTCACCAAGTTAGGTAAAGTTGAGTACAAATCTCATGAGGAGCAACTCGCCGAAAA

CCATCGCAAAATGCTGATGGCTATGTCCAAAGATATTCGCGTGATTTTGGTGAAATTGGC

TGACCGCCTGCATAATATGCGCACCCTCAAACATTTGCGCAAGGACAAACAAGAGCGTAT

TTCGCGCGAAACCATGGAAATCTATGCCCCCTTGGCGCATCGTTTGGGGATTAGTCGCAT

CAAATGGGAACTAGAAGATTTGGCTTTTCGTTACCTCAATGAAACCGAATTTTACAAAAT

TTCCCATATGATGAAAGAAAAACGTCGCGAGCGTGAAGCTTTGGTAGAGGCTATTGTCAG

TAAGGTCAAAACCTATACGACACAACAAGGGTTGTTTGGAGATGTGTATGGCCGACCAAA

ACACATTTATTCGATTTATCGGAAAATGCGGGACAAAAAGAAACGATTCGATCAGATTTT

TGATCTGATTGCCATTCGTTGTGTCATGGAAACGCAGAGCGATGTCTATGCTATGGTTGG

CTATATTCACGAGCTTTGGCGTCCCATGCCAGGCCGCTTCAAGGATTATATTGCAGCTCC

TAAAGCTAATGGCTACCAGTCGATTCATACCACTGTGTATGGGCCAAAAGGACCTATTGA

GATTCAAATCAGAACTAAGGACATGCATCAAGTGGCTGAGTACGGGGTTGCTGCTCACTG

GGCTTATAAAAAAGGCGTGCGTGGTAAGGTCAATCAAGCTGAGCAAGCCGTTGGCATGAA

CTGGATCAAAGAGCTGGTAGAATTGCAAGATGCCTCAAATGGTGATGCAGTGGACTTTGT

GGATTCGGTCAAAGAAGACATTTTTTCTGAGCGGATTTATGTCTTTACACCGACAGGGGC

TGTTCAGGAGTTGCCCAAAGAATCAGGTCCTATTGATTTTGCTTATGCGATCCATACGCA

AATCGGTGAAAAAGCAACAGGTGCCAAGGTTAATGGACGTATGGTTCCTCTCACTGCCAA

GTTAAAAACAGGAGATGTGGTTGAAATCATCACCAATGCCAATTCCTTTGGCCCTAGTCG

AGACTGGGTAAAACTGGTCAAAACCAATAAGGCTCGCAACAAAATTCGTCAGTTCTTTAA

AAATCAAGATAAGGAATTGTCAGTGAATAAAGGCCGTGATTTGTTGGTGTCTTATTTTCA

AGAGCAGGGCTACGTTGCCAATAAATACCTTGACAAAAAACGCATTGAAGCGATCCTTCC

AAAAGTCAGTGTGAAGAGCGAAGAATCGCTCTATGCAGCCGTTGGGTTTGGTGACATTAG

TCCTATCAGTGTCTTTAACAAGTTGACCGAAAAAGAGCGCCGTGAAGAAGAAAGGGCCAA

GGCTAAAGCAGAAGCTGAAGAATTGGTTAAGGGCGGTGAGGTCAAACACGAAAACAAAGA

TGTGCTCAAGGTTCGCAGTGAAAATGGGGTCATTATCCAAGGAGCATCAGGTCTCTTGAT

GCGGATTGCCAAGTGTTGTAATCCCGTACCTGGTGATCCTATTGACGGCTACATTACCAA

AGGGCGTGGCATTGCGATTCACAGATCGGACTGTCATAACATTAAGAGTCAAGATGGCTA

CCAAGAACGCTTGATTGAGGTCGAGTGGGACTTGGACAATTCGAGTAAAGATTATCAGGC

TGAAATTGATATCTATGGGCTCAATCGTAGTGGCCTGCTTAATGATGTGCTCCAAATTTT

ATCAAACTCAACCAAGAGCATATCGACAGTCAATGCTCAGCCGACCAAGGACATGAAGTT

TGCTAATATTCACGTGAGCTTTGGCATTCCAAATCTGACGCATCTGACCACTGTTGTCGA

AAAAATCAAGGCAGTTCCAGATGTTTATAGCGTGAAGCGGACCAATGGCTAAGAAAGGAT

AAAACCATGAAACTTGTCTTGCAACGGGTCAAAGAGGCCTCTGTTTCTATTGATGGGAAA

ATTGCTGGCGCTATTAACCAAGGGTTGTTGTTGTTAGTAGGTGTGGGACCAGATGATAAT

GCAGAAGATTTGGCTTACGCTGTGCGAAAAATAGTCAACATGCGCATTTTTTCAGATGCT

GATGGCAAGATGAATCAGTCTATCCAAGACATCAAGGGCAGTATCTTATCTGTGTCGCAA

TTTACCCTTTATGCTGACACCAAAAAGGGAAACCGTCCTGCCTTTACTGGTGCTGCTAAG

CCAGATTTGGCTAGCCAGTTGTATGATAGCTTTAATGAACAATTGGCAGAGTTTGTGCCA

GTTGAGCGTGGCGTTTTTGGGGCAGATATGCAGGTTAGTCTCATCAATGATGGACCAGTT

ACGATTATTTTAGACACGAAATGTCACTAGTCTCTACAACTAAGAGGTTAGAAAAAAGCA

AGCAACTGTTGATAGCGTGTTGCTTGCTTTTTTTGTCAGTTAATCTCATCGTTTTAGAAG

ATCGTGGTTATTTGTCGTTAGGGTTATCAGGTATAGGTGTCCCTGTATAACGCAGGTAGC

TGTAAACTTCTCGTTCTTCTTCGGTATAACGATCTTTATCATAAGCTAAATGATAGCTAG

CATTCTCTCCTTCGGGTCGCTTGCCCATATAAACGGTTATGATACGGTTGGTGTCATTGT

GATTATCCTCTACTTTTCCAGTTAAGGTATAGTCCATAATACCAAAAGCATCGAGATTGT

TGTAGAGTAGTTTAGCCTTATCACGAGGATCGTATAAATCTCTGAAGTCTAAGTTACGTT

CGCTAGCTGTTAAGAGCTGCTCGCTTTTTAGCAATTCGTTGGTGTTGACATCAACGTAAT

TGATGGTGAACAGTTTCAAGTGACTGCGATCAAAGGGATCATACGGCTTTTCCCCTTTTT

TAAGGACGTAATATTTCTCAGAGATAAGGTCAGTGTTGTTCGTTTTTTCTACAATACCTG

TTTTAGAATTGGCCTTATAAGCTTGTTCCCGGTCTTTGATATGGTAAGTAAACTCTTGAT

CCATTGGTAAAATCGTACGGAAAATGTCATTGTCATGAGTGACGATTGAGGAGTCACGTT

TTGTAATGATATAATCTGGATATTTTTTTGATAGGATAAATTGGGCAATCGCTGCTAACT

CTTGTGATGAAAGAGAGTCACCAACTGCCAGTGTGGTCAAATGGTATTGGTTTCTTAACA

ACGGTGTAAAGTCTAAATCTCCTGTTTCGGAGACAAAGCTCACTTCATAGTTGACGTTAA

CGCGTTCAGCTGAGTTGTGAACGGCTTTAGGTTGATACGGTCTAACGCGCACATGCCCGC

TTAGCAAAAATTCTTGGACAGGTTGGGTCGGCAAGGTTACCGAATCATCTCGGTCAGCAA

AGTAGACCTTGCCGTTTCGATCAGTAATGGTTGCATCGCTTGCAAAATCAATGACCTCAA

AGTAGCCGTCGTTACTGTGAACGTTAGCGATCAATTGTTCTTGAATAGCCTTTAATAAGT

CAGCTTTTTCAAGTTTATGTGGCATTGCGCCTTTATTTGTAGCAAATGGTTTTGATTTTG

GACTTAAGCCTTGCTCTGTCTTTCCTCCCTGAGCAGGTCCTGATGTTAGGTCAATTTCAA

AAAATTTAAGGCTAATTTCTTGATTCGTCCCCTCAACAGTACCGGCAACGCTAACAACTA

ATTGGATGTTGTTGACAGATGGACGGTCTAGCAGCCATTCAGGTCCAGCAATAGCTTGGA

CAGGCTTGACTGTTCCAAATGTTAATGCAAACAGCAGTGCAAACATCCCAAAAGATAAGT

AATTTTTCATAGAAACCTCCTAAAAGTTAAGTTTCAATCCCCATTATTAGAATAACATAA

TAATTTTATAGATACAAACAAAAACCTAATAATTGTTTTTAAATGATATCTTAATGATAA

TGTCATGATAATGAAAAAAGAGAAACAATACCTGTTTTAGTATAAACATAAATTAATCTT

CCGATAAATAGCTATTTGATTGAATGTTATTGCTAAAAATGAATAACCATTATTTAGTTT

TAATGATTAAAAAGTCAAAAAATACAAAATCGTGCTGACAACAAACCGAACTGTGTGATA

AAGAGTCATGTGTTATAATGGTTTAAAGACCGTCATGAAAGGAATCGCTATGGAGCTAAA

AGACTATTTTCCAGAGATGCAAGTAGGCCCTCATCCGTTAGGAGATAAAGAGTGGGTGAG

TGTCAAAGAAGGGGACCAATATGTGCATTTTCCCAAATCATGTTTATCCGAAAAAGAGCG

CTTATTACTTGAGGTGGGGCTGGGTCAGTGTGAGGTCTTGCAGCCGCTAGGGTCACCTTG

GCAGCGGTATTTGCTTGATCATCAGGGCAATCCCCCTCAGCTGTTTGAGACTTCTCAGTT

TATTTACCTCAATCATCAGCAGGTTTTACCAGCTGACTTAGTTGAGTTGCTCCAGCAGAT

GATTGCAGGGCTTGAGGTCATTTTGCCTATCAGCACAACCCAAACAGCATTTTTATGTCG

ACAAGCAACTTCAATAAAGGTCTTGCGCTCGCTTGAAGGTCTCTTGCCAACGCTAGAAAG

TGATTTTGGTTTGGCCTTAACCATGTTTGTTGGAAATGCCTGGTATCAAGTAGCTGCGGG

TACATTGAGAGAGTGTTTTGAGGAGGAATGTCAGCTGTTAACGGCCTATTTAAGGCAACA

ATCAGGCAGAAAATTATTAACATTTTCAGGACTGATGCTGTGGTCTTTGCTGTCGCACCA

CACTTTTTTGGCATTGACGCGTCAGTTTCACCAATTCCTAAGTCCTCAAAGTGATATGGC

CGATGTGGTTCACGCGCTATGGTCAGAACACGGCAATCTGGTGCAGACAGCGCAGCGCTT

GTATATTCACCGTAACTCCCTGCAATACAAACTTGATAAATTTGCTCAGCAATCAGGACT

ACACTTGAAGCAATTGGATGATTTAGCCTTTGCTCATCTCTTTTTGTTGAAATATTAGGA

AAGCCACAGGGCTTTCTTTTTTGCTGCCTGGACTGTAGCTGATAAGGAGAATGATAGATC

GTTCAGCTTGTTTTGGCTCCTGTGACTGGCCTGTGTTTACATGGTTTTCTTGGTTTATGG

TCCCTTACGACTTGACTAGCCCTGCTATTGTGCAAGTTGCCCAAAGAAGTTTGGTCGCTT

TGTCTCTGTTTTGAAAGCGCTATTTTTGTTATAATAAAAGCATCATATAAAAGATATCAG

CGGTTTCTAGCTTTGCAGTCAGCTAGAACGTTTGCTGGCTAAATAGGAGTTATACATGGT

TGAATTAAATCTAAATCACATCTATAAAAAATACCCAAATACTACTCATTATGCCGTTGA

AGATTTTGACTTGGACATCAAAGATAAAGAATTTATCGTCTTTGTTGGACCTTCAGGATG

TGGTAAATCAACCACTCTTCGTATGATTGCAGGTCTTGAGGACATTTCTGAAGGAGAGTT

AAAAATCGGTGGCGAAGTGGTGAATGACAAGTCTCCTAAAGACCGTGATATTGCCATGGT

CTTTCAAAACTACGCCCTTTACCCACACATGACCGTGTATGATAACATGGCCTTTGGTTT

GAAATTACGTAAGTACAAAAAAGATGATATTGACCGTCGTGTCAAGGAAGCTGCGCAGAT

CCTTGGTTTGACCGAATTTCTAGAGCGTAAACCGGCTGACCTGTCTGGTGGTCAACGTCA

GCGTGTGGCTATGGGACGTGCTATTGTCCGTGATGCTAAGGTATTCTTGATGGACGAGCC

TTTGTCAAACTTGGATGCGAAGCTTCGTGTCTCCATGCGTGCTGAAATTGCTAAAATTCA

CCGTCGTATCGGCTCAACAACTATTTACGTTACCCATGACCAGACTGAAGCCATGACTTT

GGCAGATCGTATCGTTATCATGAGTGCCACTAAAAACCCTCAAGGTAATGGAACGATTGG

TAAAATTGAGCAAGTAGGTTCACCACAAGAATTGTACAACCTTCCAGCTAATAAATTCGT

TGCCGGCTTTATCGGAAGCCCTGCCATGAACTTCTTTGAAGTGGAAGTTAAAGATGGCCG

CATTGTGAGTGAAGATGGCCTTGCTATTGCGATTCCTGAAGGTCAAGCAAAAATGCTTGA

AGCTGCTGGTTACAAGGGGAAAAAAGTCACTTTTGGTATTCGTCCAGAAGACATTTCTAG

CAGACAAATCGTGCATGATACTTACCCAAGTGCAACTGTCACAGCAGAAGTTTTGGTTTC

TGAGTTACTGGGCTCTGAGACGATGCTCTATGTCAAGCTTGGACAAACTGAGTTTGCTTC

TCGTGTAGATGCGCGTGATTTCCATAGTCCAGGGGAGCAAGTTAGCTTGACCTTTAATGT

GGCTAAAGGGCATTTCTTTGACAGAGATACCGAACAAGCTATTCGTTAAGGTATAAGCGA

TATTCATAAAAAGGTTGGGTTCCAGCCTTTTTATCATGTCATTTTGCGCAAACGATTGCA

TAAAAAGAGAAAAAACGTTAAAATAAAAGATGAATGAATGATTAATAGGAGAAGAATAAT

GAAGAAACACTGGTGGCATAAAGCTACGATTTATCAGATTTACCCAAGGTCCTTTAAAGA

CACCAGCGGAAATGGTATTGGTGATTTAAAAGGCATTACCAGTCAACTGGACTACTTGCA

AAAACTTGGCATTACAGCCATTTGGTTATCTCCTGTTTACCAAAGTCCAATGGATGACAA

TGGTTATGACATCTCTGATTATGAAGCGATTGCTGATGTTTTTGGAGACATGGCAGATAT

GGATGAGCTCTTAGCAGCAGCTAATGAGCGCGGCATTAAGATTATCATGGACTTGGTGGT

CAACCATACGTCTGATGAGCATGCTTGGTTTGTTGAAGCTAGGGAAAACCCTAATAGTCC

TGAGCGTGATTTTTATATTTGGCGAGATGAACCTAACGATTTGACTTCTATTTTTAGTGG

CTCAGCTTGGGAATATGATAATGCATCAGGTCAATATTATTTGCACCTTTTTAGCAAACG

TCAGCCAGATCTCAACTGGGAGAATGAAGCTTTACGTCATAAAATTTACGATATGATGAA

TTTCTGGATTGCCAAAGGCATCGGTGGTTTTCGTATGGACGTGATTGACCTGATTGGGAA

GGTTCCTGACTTGGAAATCACGGGTAACGGGCCGCGCTTACACGATTACCTCAAAGAAAT

GAATCAAGTAACCTTTGGCAACCATGACGTGATGACTGTCGGTGAGACTTGGGGAGCAAC

GCCTGAAATTGCTCGCCAGTATTCTCGCCCTGAAAACAAAGAGTTATCCATGGTCTTCCA

ATTTGAGCATGTGGGTCTTCAACATAAACCTGACGCTCCCAAGTGGGATTATGCCAAAGA

ATTAGATGTCCCTGCTCTTAAAACCATTTTCAGCAAGTGGCAGACAGAATTAAAGTTAGG

AGAAGGCTGGAATTCGCTTTTTTGGAATAACCATGACTTGCCACGGGTCTTATCCATTTG

GGGCAATGACAGCACTTACCGAGAAAAATCAGCCAAAGCTTTAGCTATTTTACTGCATTT

GATGCGAGGAACCCCTTACATTTACCAAGGAGAAGAGATTGGCATGACCAATTATCCTTT

TAAAGACTTAACCGAGGTTAATGATATTGAGTCCCTTAACTATGCTAAGGAAGCTATGGG

AAATGGAGTGTCAGCTGCGCGTGTGATGGATAGCATCCGTAAGGTAGGACGAGACAATGC

TAGAACGCCGATGCAGTGGTCTAAGGACACACATGCTGGCTTTTCTGAAGCAAAAGAGAC

TTGGTTGCCGGTCAACCCCAACTACCAAGACATCAATGTGGCCGATGCCCTTGCTGATCC

AGACTCTATTTTTTACACCTACCAAAAATTGATTGCCTTGCGTAAGGAGCAAGACTGGCT

AGTTGAAGCGGATTATCACCTCTTGCCAACAGCTGATAAAGTGTTTGCTTATCAGCGTCA

GCTTGGAGAAGAGACGTATGTGATTGTTGTCAATGTGTCTGATGAGGAGCAAGTCTTTGC

CACAGACTTAGCAGGTGCACAAGTGATCATCGCCAATACAGATGTGGACACGGTATTAAA

GACCAAGCACTTACAACCTTGGGATGCCTTTTGTCTCAAACTGAAGGCCTAGCAGTTGTA

TGTGATTTGAGTTTACCCAACAGCCAAGCGAGCGGAAACTCGTTTTGGCTGTTTAATTTT

TGAGATCCTATTAAGGGGTGATAGTCATTGTTTGAGCTGTGTTCTAAGTTGAAGTGCGGC

ATGTCTTGCAAGCGCTTGCGCAATGTGATAAGATAGTCTTGTTTGAATGTTATACTAAAT

GGAGGTCTTATGAAAAAGAAAGTCAACCAAGGATCAAAGCGCTATCAACATCTGTTAAAA

AAGTGGGGGATAGGTTTTGTAATCGCTGCAACTGGGACTGTCATGTTAGGGTGCACCCCT

AGTATCTTAACACATCAAGTTGCTGCTAAAACCATTGTTGGACTAGCCCGCGATGAAGCT

CAACAAGGAGATGGCAATGCTAAATCTGGTGATGGTTTTCAATCGTCTAGTAAGGAGGCT

AAACCAGTTTTAGACAGCTCGTCAGCTAATCCTGCTAGTATTGCTGAGCATCATTTGCGT

ATGCATTTTAAAACATTGCCAGCTGGTGAGTCGCTAGGAAGCTTGGGACTTTGGGTGTGG

GGAGATGTGGATCAACCTTCAAAGGATTGGCCAAATGGTGCTATCACCATGACAAAAGCG

AAAAAAGATGACTATGGCTATTATCTAGATGTGCCACTAGCAGCTAAACACCGCCAGCAA

GTGTCTTATCTCATTAATAATAAAGCTGGAGAGAATCTTTCAAAGGACCAGCACATCTCG

CTTCTTACGCCAAAAATGAATGAAGTTTGGACAGACGAGAATTACCATGCGCACGCTTAT

CGACCTTTGAAAAAAGGTTACCTTCGAATCAACTACCACAATCAATCGGGACACTACGAT

AACTTAGCTGTCTGGACCTTTAAAGATGTCAAAAACCCAACGACCGACTGGCCAAATGGA

CTTGACTTGTCACATAAAGGGCCTTATGGAGCTTATGTTGATGTCCCCTTAAAAGAAGGA

GCTAACGAAATCGGATTTTTAATCCTTGATAAAAGTAAGACAGGAGATGCTATTAAAGTG

CAACCAAAAGATTATCTGTTTAAAGAGTTAGACAATCATACTCAAGTTTTTGTCAAAGAC

ACTGACCCAAAAGTCTACAACAATCCTTATTATATTGATCAGGTTAGTCTCAAAGGAGCT

GAACAAACCACGCCAAATGAGATTAAAGCCATTTTTACGACCTTAGATGGGCTTGATGAA

GATGCGGTGAAACAAAACATCAAGATCACTGACAAAGCAGGGAAAACTGTTGCAATTGAT

GAGTTGACACTTGACAAGGATAAGTCTGTAATGACATTAAAGGGCGATTTTAAGGCGCAA

GGTGCAGTCTACACGGTTACATTTGGAGAAGTTAGCCAAGTCGCTCGCCAATCCTGGCAA

TTAAAAGATAAACTCTATGCTTACGATGGTGAACTTGGAGCTACCCTAGCTAAGGATGGT

TCTGTTGATTTAGCGCTATGGTCTCCAAGTGCTGATACTGTTAAGGTTGTCGTTTACGAT

AAACAAGATCAGACAAAGGTGGTTGGTCAAGCTGATTTGACCAAGTCGGACAAGGGTGTT

TGGAGAGCTCATCTGACTTCTGACAGTGTCAAGGGAATTAGTGATTACACAGGCTACTAT

TACCTTTATGAGATCACGCGCGGTCAGGAAAAAGTCATGGTTTTGGATCCTTACGCCAAA

TCTCTCGCTGCCTGGAATGATGCGACTGCTACTGATGACATCAAAACAGCAAAAGCTGCC

TTTATTGATCCAAGCAAACTAGGACCAACAGGCCTTGATTTTGCCAAAATTAACAACTTT

AAAAAACGTGAAGACGCTATTATCTATGAAGCACATGTGCGAGATTTTACGTCGGATAAG

GCTCTAGAAGGCAAGTTAACACACCCTTTTGGGACTTTTTCAGCTTTTGTTGAACAGCTA

GACTATCTCAAAGACTTGGGGGTTACCCACGTTCAATTGCTACCGGTTTTGAGTTATTTT

TATGCCAATGAGCTGGACAAGAGCCGCTCAACAGCCTACACGTCTTCAGACAATAATTAC

AACTGGGGTTATGACCCACAACACTACTTTGCCCTTTCTGGCATGTATTCGGTAAATCCT

AACGACCCTGCTTTACGTATCGCAGAGCTTAAAAACCTTGTCAATGAGATTCACAAACGT

GGTATGGGTGTTATTTTTGATGTGGTTTATAACCACACGGCTAGAACCTATCTCTTTGAA

GATTTGGAACCCAACTACTATCATTTTATGAATGCTGATGGCACAGCTAGAGAGAGTTTT

GGCGGAGGTCGTCTAGGAACGACACATGCCATGAGTCGTCGTATCTTGGTGGATTCGATT

GCTTATCTGACTCGTGAATTCAAGGTAGATGGTTTTCGTTTCGACATGATGGGTGACCAT

GATGCGGCAGCTATTGAGCAAGCCTTTAAGGCAGCCAAAGCCATTAATCCAAATACCATT

ATGATTGGCGAAGGCTGGCGTACCTACCAAGGTGATGAGGGGAAAAAAGAAATTGCGGCA

GATCAAGATTGGATGAAAGCAACCAATACGGTCGGTGTTTTCTCTGATGATATCAGAAAT

ACCCTCAAGTCAGGTTTCCCAAATGAAGGCACAGCAGCCTTTATTACTGGTGGCGCAAAA

AATCTAGAAGGACTGTTCAAAACGATCAAAGCACAGCCTAGTAACTTTGAAGCAGATGCC

CCAGGAGATGTAGTGCAGTATATTGCAGCCCATGACAACCTGACCTTACATGATGTCATT

GCCAAATCCATCAATAAGGATCCTAAAGTGGCTGAAGAAGAGATTCATAAGCGTATTCGT

CTAGGAAATACCATGATTTTAACTGCTCAAGGGACTGCCTTTATCCATTCTGGTCAGGAA

TATGGACGAACCAAGCAGCTTCTAAATCCCGACTACAGGACAAAGGTGTCTGATGACAAG

GTGCCAAATAAGGCGACTCTGATTGATGCTGTAGCGAAATACCCTTACTTCATCCACGAT

TCTTATGATTCGTCTGATGCGGTCAATCATTTTGACTGGGCAAAGGCAACAGATTCCATA

GCTCACCCGATTAGCAACCAAACAAAAGCCTATACACAGGGACTAATTGCGTTGCGTCGC

TCAACAGATGCCTTTACAAAAGCAACCAAAGCTGAGGTAGATCGGGATGTGACCTTGATC

ACCCAAGCAGGACAAGATGTTATTCAACAAGAGGACCTTATCATGGGTTACCAAACGGTG

GCATCAAATGGAGATCGCTATGCTGTCTTTGTCAATGCAGACAACAAGACCCGCAAGGTA

GTTTTACCTCAAGCCTACCGCTATTTGCTAGGAGCCCAAGTGCTTGCTGATGCTGAGCAA

GCTGGTGTTACTGCCATTGCTAAGCCTAAGGGAGTCCAGTTTACCAAAGAAGGCTTGACT

ATTGATAGCCTAACTGCCCTGGTCCTCAAAGTATCTTCAAAACCGGCTGATCCCTCTCAG

AAAAAGAGTCAGACAGGCAATCATCAAACCAAAACACCAGATGGCTCAAAAGACCTAGAC

AAATCATTAATGACTAGGCCAAAAAGAGCTCAAACAAACCAAAAGCTCCCAAAAACGGGT

GAAGCCTCCTCAAAAGGCTTATTAGCAGCTGGAGTAGCTCTGCTTTTATTGGCTATTAGC

CTGTTGATGAAGCGCCCAAAAGATTAGGGTGGTGTTAATAGAGACAAAAAGATTGCTGTT

TGCGGATTCTTTTTGACGGCTATAAAAGCTCTGAGCTGTGAAAAGACCTTGTGATTGAGA

AAATTGCTACTTTAGTGTATCATAAGACCAAAAGAGAAGGAGACATCCATGACATTTGAA

GAAATCGTTGCGAATTTTATACCGTCGAGTGTTGCAGAAGTCACATCAGCTATTGCTAGT

GGCAAAGACATGATTGTCTTTTTAGGACGTTCGAGCTGTCCTTATTGTCGCCGCTTTGCT

CCTAAGTTGGCACAAGTGGCAACAGATAATCAAAAAGACGTCTATTTTGTGGATAGCGAA

AACGCTGCTGACGCTGCTGAGCTTGCTGCCTTTCGTGAGAACTACCAATTAGTGACAGTT

CCAGCTTTGCTGGTTAGTTACGATCAGCACCAAAGGGCTGTATGTGATTCAAGTTTGACA

CCAGATGACATCTTAGCTTTTTTGACTCGTGAGTAAAGCACTGCGCTTAGCGTCTCGTTT

AAAGAAGAAATCAGGTAACGTCTAGTCTTGCTATACCGCTTGATTTTTGATATAGTAAGA

CATGACTTTTATAGAATAAAAAGGAGACATTATGGGATTTCCAACAATTTTAATGTTTGT

AGTGATGCTTGGATTGATTTGGTTTATGCAACGTCAACAAAAAAAACAAGCGCAAGAACG

CCAAAATCAGTTAAACGCTATTGAAAAAGGAGACGAAGTCGTAACGATTGGTGGCATGTT

TGCGATTGTTGACGAAGTCGATACAACAGCTAAAAAGATTGTGTTAGATGTGGATGGCGT

CTTTTTGACATTTGAGCTATCAGCTATTAAGCGTATTGTAACAAAAGCTACCACAGAAAC

GACACTGGTTGAAACAGAAGAAACCAGTGTTGTTAATGAGCCTGCTGTTGAGACAGTAGA

TAGCACTGACAGTGCCATTGAAAGCCATTAAGGCGGGGAAAGTTTGGATGAAATATGCCA

AACTTTTTTGTTTTGATCCCTGGACTGAAACAGTTATTCTGATGCTAACAGGTTCATGAA

AAGCTGACACATCTATCAAAGGATAGGTGAACATTTTGTGAAGATAAGATCTAATTGATA

AAAAACAAGCCAAAAGGCGTGAAATATGTTAAAATATGAGATAAGTATGCTTATCAATAA

GGAAAAAACATGTTTGGATTAAAAGCGAAATCAACAAAAAAAGTTTTGGGGAGTATCCCA

AAGCATATTGGAATTATCATGGATGGTAATGGACGTTGGGCCAAAAAAAGACTAAAACCT

AGAGTGTTTGGGCATAAAGCTGGGATGGATGCTTTACAAGAAGTAACGATTACTGCATCT

GAGTTGGGGGTTAAAGTTCTGACAGTCTATGCCTTTTCGACAGAAAACTGGTCCAGACCC

CAAGATGAGGTGTCTTTTATCATGAATTTACCAGTAACCTTCTTTGACAAATATGTGCCT

GTGCTTCATGAGAACAATGTAAAGATTCAAATGATTGGAGAGACTAGTCGTTTACCTGAA

GATACCTTGGCAGCGCTAAATGCAGCCATCGACAAAACAAAACGTAATACGGGTTTAATT

TTGAATTTTGCTCTCAATTATGGAGGACGTGCTGAGATTACTAGTGCTGTTCGCTTTATT

GCTCAAGATGTCTTAGATGCCAAATTAAATCCAGGAGATATTACGGAGGATTTGATTGCT

AATTATTTGATGACAGACCATCTGCCATATCTCTACCGAGACCCCGATTTGATTATTCGC

ACCAGTGGTGAGCTGCGTTTGAGCAATTTCTTGCCTTGGCAATCAGCTTACAGTGAATTT

TATTTCACACCAGTCTTGTGGCCTGATTTTAAAAAGGCAGAGCTACTGAAGGCCATTGCG

GATTATAACCGCCGCCAACGTCGTTTTGGCAAGGTCTAAAAGGAGATAGTTATGAAAGAA

CGTGTTGTTTGGGGGGGAGTAGCAGTAGCTATTTTCCTACCTTTTCTCATAATAGGAAAC

CTACCATTCCAATTATTTGTAGGCGTTTTGGCCATGATTGGGGTATCAGAATTACTGAAA

ATGAAAAGATTAGAAGTCTTTTCTTTTGAAGGTGTTTTTGCCATGTTAGCCGCCTTTGTA

TTGGCAGTTCCGATGGACCATTACCTCACTTTTTTACCAATTGATGCGAATGTTGCTTTT

TATAGCTTAATGGTTTTTTTCATTTTGGCAGGTACTGTTTTAAATAGCAGAGCTTATTCA

TTTGATGATGCGGCCTTTCCGATTGCAACTAGTTTTTATGTTGGGATTGGTTTTCAACAT

TTGATCAATGCTCGCCTGTCAGGAATTGACAAGGTCTTTTTGGCCTTATTTATCGTTTGG

GCCACTGATATTGGAGCATATCTGATTGGGCGTCAGTTTGGCAGGCGCAAACTATTGCCT

ACAGTTTCTCCAAACAAAACCATTGAAGGTAGTTTAGGAGGGATCGCTTGTGCAGTCCTT

GTGTCTTTTATCTTTATGATGATCGATAGGTCTGTTTATGCACCACATCACTTTTTGACC

ATGTTGGTTTTGGTCGCTCTTTTTTCCATTTTTGCCCAATTTGGTGATTTGGTTGAAAGT

GCTCTCAAACGTCACTTTGGTGTCAAAGATTCAGGTAAGCTTATCCCGGGACATGGTGGT

ATTTTAGACCGCTTTGATTCCATGATTTTTGTTTTTCCCATCATGCATCTCTTTGGTTTA

TTTTAATCTTAAGAGGTAATGTTTGGAGTCGCAACAACGCGACAGAACTCAAAAAAGCAA

TAGACTGAAAGGAAATCTATGTTAGGAATAATAACCTTTATTATTATTTTTGGTATTTTA

GTGATTGTCCATGAATTTGGACATTTCTATTTTGCTAAAAAATCAGGCATTCTAGTAAGA

GAATTTGCCATTGGAATGGGCCCTAAAATTTTTTCTCATGTTGACCAAGGAGGAACTCTT

TATACCTTGAGGATGTTACCCTTGGGCGGTTATGTGCGAATGGCTGGTTGGGGTGATGAC

AAAACTGAGATCAAAACAGGTACTCCAGCAAGTTTAACCCTTAATGAGCAAGGTTTTGTT

AAGCGCATCAACTTGTCTCAAAGTAAGTTAGACCCAACGAGTCTCCCAATGCATGTTACA

GGCTATGACTTAGAAGATCAGCTGAGTATTACTGGCTTGGTTTTAGAAGAAACCAAGACA

TATAAGGTCGCTCACGATGCTACTATTGTTGAAGAAGACGGTACTGAGATAAGAATTGCT

CCGCTTGATGTTCAATATCAAAATGCTAGTATTGGCGGACGTTTAATCACCAATTTTGCA

GGTCCCATGAATAATTTTATTTTAGGGATTGTGGTTTTTATCCTCTTGGTCTTTTTACAA

GGTGGGATGCCAGATTTTAGTAGCAATCATGTCCGTGTTCAAGAAAATGGAGCAGCAGCT

AAGGCTGGTCTTCGAGATAATGACCAAATTGTCGCAATTAATGGTTACAAGGTGACTAGC

TGGAATGATCTCACTGAGGCTGTAGACCTTGCAACACGGGATCTAGGCCCGTCACAGACC

ATTAAGGTCACCTACAAGTCACATCAGCGCTTAAAAACGGTGGCTGTGAAACCACAAAAG

CATGCAAAGACATACACGATAGGAGTTAAGGCGAGTCTGAAAACAGGATTTAAGGATAAG

CTCTTAGGCGGTCTAGAATTAGCTTGGAGTGGGGCGTTTACTATTTTGAATACTTTGAAA

GGATTGATCACTGGCTTTAGTCTCAATAAATTAGGTGGACCTGTTGCCATGTATGACATG

TCCAATCAGGCTGCTCAAAACGGCTTAGAGTCAGTCTTATCTCTTATGGCAATGCTTTCG

ATCAATTTAGGGATCTTTAACCTGATTCCGATTCCTGCACTTGATGGGGGAAAAATCTTG

ATGAATATCATTGAAGCCATTCGTCGCAAGCCTATCAAGCAAGAAACAGAGGCCTATATC

ACCCTAGCTGGGGTTGCTATCATGGTTGTATTGATGATTGCTGTGACATGGAATGATATC

ATGCGCGTCTTTTTCTAATCAAGATAGGTCATCTTAAGCTGACCTTATCATGTTAAAGAT

CGAAAAAGACCTCAACCCTAAGGTTTGCAAGGGTTGAAGTCTTACTCAAAATGAAAGGAA

TACACATTCAAATTATTGAATAATCCCGCTTCACTCTTACTTGATGTTTCCAGAAGGGTT

TGAGGATAAGGGGATGTATTAAAACGGAATAATCTATGAAACAAAGTAAACTGCTTATCC

CAACCTTGCGCGAAATGCCAAGTGATGCCCAGGTTATCAGCCACGCGCTTATGGTGCGTG

CCGGTTATGTGCGCCAAGTTTCTGCTGGTATCTATGCTTATTTACCACTGGCAAATCGTA

CCATTGAGAAATTCAAGACCATCATGCGTGAAGAGTTTGAAAAGATCGGTGCTGTTGAAA

TGTTGGCACCAGCTCTTTTGACAGCTGATCTCTGGCGTGAATCAGGCCGTTATGAGACCT

ATGGAGAGGACCTCTATAAGCTTAAAAACCGTGATAACTCAGACTTTATCTTGGGTCCGA

CCCACGAAGAAACCTTTACGACTTTGGTGCGTGATGCGGTCAAATCTTACAAGCAATTGC

CCTTAAACCTTTATCAAATTCAGTCTAAGTATCGTGATGAAAAACGTCCGCGTAACGGCT

TGCTTCGTACCCGTGAGTTTATCATGAAAGACGGCTATAGTTTCCATCACAACTATGAAG

ATTTAGATGTGACCTACGAAGATTATCGTCAAGCTTACGAAGCTATTTTTACCAGAGCTG

GTCTAGATTTCAAAGGGATTATTGGAGATGGCGGTGCCATGGGGGGGAAAGATTCCCAAG

AATTTATGGCCATTACACCAGCTCGTACGGACCTTGATCGCTGGGTGGTTCTTGACAAGT

CTATTGCGTCAATGGATGATATTCCAAAAGAGGTCTTAGAAGAGATTAAGGCAGAATTGG

CTGCTTGGATGATTTCAGGTGAAGATACTATTGCTTATTCAACAGAATCAAGCTACGCTG

CCAACCTTGAGATGGCAACTAACGAATACAAACCGTCCTCAAAAGTAGCTGCTGAAGATG

CCTTGGCAGAAGTTGAGACACCACATTGCAAAACGATTGATGAAGTGGCTGCTTTTCTTT

CAGTAGATGAAACACAAACCATCAAAACCTTGCTTTTTGTGGCAGATAATGAACCTGTTG

TTGCTTTGCTAGTTGGAAATGACCATATCAATACCGTTAAATTAAAAAACTATCTAGCTG

CTGATTTTTTAGAGCCAGCTAGTGAAGAAGAAGCCCGTGCTTTCTTTGGTGCAGGTTTTG

GCTCACTTGGGCCTGTCAACTTGGCACAAGGTAGTCGCATTGTGGCTGACCGCAAAGTGC

AAAACCTTACCAATGCCGTTGCAGGAGCTAACAAGGATGGTTTCCATGTGACAGGAGTCA

ATCCAGGACGTGACTTCCAGGCTGAATATGTAGATATTCGTGAAGTCAAAGAAGGGGAAA

TGTCTCCGGATGGTCATGGTGTTCTCCAGTTTGCGCGTGGTATCGAAGTCGGTCATATCT

TCAAGTTGGGCACTCGTTATTCAGACAGCATGGGAGCAACGATTCTTGATGAAAATGGCA

GAACAGTTCCAATTGTGATGGGTTGTTATGGTATCGGGGTTAGCCGCATTTTGTCTGCTG

TCATTGAACAGCATGCCCGTCTCTTTGTGAACAAGACACCAAAAGGCGATTACCGTTATG

CTTGGGGTATTAACTTCCCTAAAGAATTAGCCCCATTTGACGTGCATTTGATTACAGTTA

ATGTCAAAGATCAAGTGGCCCAAGACTTGACGGCGAAGTTAGAAGCTGACTTGATGGCTA

AAGGGTATGATGTCTTGACAGATGACCGTAATGAACGCGTCGGTTCGAAATTCTCTGATA

GCGATTTGATTGGTTTGCCAATTCGTGTCACTGTTGGTAAAAAAGCCGCTGAAGGTATCG

TGGAAATTAAAATCAAGGCAACAGGTGACAGCATTGAAGTTAATGCAGAAAACCTCATCG

AAACCCTTGAAATTTTAACAAAAGAACACTAAAAAAGAAATGAAGAGGCTGCCTGTTTAG

AGTAGCCTCTTTTTGCTGTGAACTTGTCTGATACAGAGTGGCTTTTGCTTTACCAAAAGC

GCTTGGCGACAAAAGATTGGCAAAAAAATGACCTCAGCTGAGCTATCACTTGGAATAAAT

TAGCCAAAGAACGTCAGAAGAAAGCCCTGCTTTTCTTTCCATCTAAAACTCGAAAATCAT

CTTGAATTGTGATAAAATAAAGCCTAGTAAAATCAGGAGAAAATTATGTCAGATTTATTC

GCTAAATTGATGGACCAGATAGAAATGCCACTTGACATGAGACGTTCAAGTGCCTTTTCA

TCTGCTGATATTATCGAGGTAAAGGTGCATTCGGTGTCACGCTTGTGGGAATTTCATTTT

GCCTTTGCGGCGGTTTTACCGATTGCAACTTATCGTGAATTACATGATCGTTTGATAAGA

ACTTTTGAGGCGGCTGACATTAAGGTAACCTTTGATATCCAAGCTGCTCAGGTGGATTAT

TCAGATGATCTGCTTCAAGCTTATTACCAAGAAGCTTTTGAGCATGCACCGTGTAATAGT

GCTAGTTTTAAATCTTCTTTCTCAAAGCTCAAAGTGACTTATGAGGATGACAAACTCATT

ATTGCAGCGCCAGGTTTTGTGAATAACGATCATTTTAGAAACAATCATCTGCCTAATCTG

GTCAAGCAATTTGAAGCCTTTGGCTTTGGCACCTTGACCATAGATATGGTGTCAGATCAG

GAGATGACTGAGCATTTGACCAAGGATTTTGTTTCCAGTCGTCAGGCTCTTGTGAAAAAG

GCTGTGCAGGATAATTTGGAAGCCCAAAAATCCCTTGAAGCCATGATGCCACCAGTTGAG

GAAGCCACACCTGCTCCTAAGTTTGACTACAAGGAACGAGCAGCTAAGCGTCAGGCAGGG

TTTGAAAAAGCGACCATCACACCAATGATTGAGATTGAGACCGAAGAAAACCGGATTGTC

TTTGAGGGGATGGTTTTTGATGTGGAGCGTAAAACGACTAGAACAGGTCGCCATATCATC

AACTTTAAAATGACAGACTATACCTCCTCGTTTGCTCTCCAAAAATGGGCTAAAGACGAT

GAGGAGCTCCGTAAATTTGATATGATTGCTAAGGGGGCTTGGTTACGGGTACAAGGTAAT

ATTGAGACCAATCCTTTTACGAAGAGTCTCACCATGAATGTTCAGCAGGTCAAAGAAATT

GTCCATCATGAGCGCAAAGACCTGATGCCAGAAGGACAAAAACGGGTCGAACTTCATGCC

CACACCAATATGTCTACCATGGATGCCTTACCGACAGTAGAAAGCTTGATTGATACGGCA

GCCAAGTGGGGACACAAGGCGGTTGCTATTACCGATCATGCTAATGTGCAAAGTTTTCCT

CATGGCTACCATAGGGCTCGCAAAGCTGGAATTAAGGCTATTTTTGGTCTAGAAGCCAAT

ATTGTTGAGGACAAGGTGCCTATTTCTTATGATCCTGTTGATATGGATTTGCACGAAGCT

ACCTATGTGGTCTTTGACGTGGAAACCACAGGTCTATCTGCTATGAATAATGACCTGATT

CAGATTGCGGCTTCCAAAATGTTTAAAGGAAATATTGTAGAGCAGTTTGATGAATTCATT

GATCCTGGGCATCCTCTTTCTGCCTTTACCACCGAATTGACAGGGATTACCGATAAGCAT

TTGCAGGGCGCCAAGCCATTGGTTACCGTCCTAAAAGCTTTTCAGGACTTTTGCAAAGAT

AGTATTTTAGTTGCTCACAACGCCAGTTTTGACGTGGGCTTTATGAACGCCAATTATGAA

CGCCATGACTTGCCCAAAATCACACAGCCTGTGATTGATACCTTAGAATTCGCAAGAAAT

TTATATCCTGAATACAAACGTCACGGTTTGGGACCACTCACCAAGCGTTTCCAAGTGAGT

CTAGACCACCATCATATGGCCAATTACGACGCGGAAGCCACAGGACGTCTTTTGTTTATT

TTTCTAAAAGATGCCAGAGAAAAGCATGGCATCAAAAACCTTTTGCAACTCAATACAGAT

TTGGTAGCTGAGGACTCTTACAAAAAAGCGCGGATTAAGCATGCGACTATCTATGTGCAA

AATCAGGTTGGTCTTAAAAATATGTTTAAGTTGGTCAGCCTTTCCAATATCAAATATTTT

GAAGGGGTGCCGCGTATTCCAAGAACCGTCTTAGATGCTCACAGAGAGGGTTTGTTACTA

GGAACAGCTTGTTCGGATGGTGAAGTTTTCGATGCCGTTCTGACTAAAGGAATTGATGCG

GCGGTTGATTTGGCTAAGTATTATGATTTTATCGAAATCATGCCACCAGCCATTTACCAG

CCATTGGTTGTCCGTGAATTAATCAAAGACCAAGCAGGTATTGAGCAGGTGATTCGTGAC

CTCATTGAAGTAGGGAAACGAGCTAATAAACTTGTACTTGCCACTGGAAATGTGCATTAC

CTAGAGCCTGAAGAAGAGATTTACCGTGAAATTATTGTGCGTAGTCTCGGTCAGGGTGCC

ATGATTAATAGAACAATCGGCCGTGGGGAAGGGGCCCAACCTGCTCCCCTACCTAAAGCG

CACTTTAGAACAACCAATGAAATGCTGGATGAGTTTGCCTTTCTTGGAAAAGACCTCGCT

TATCAAGTGGTTGTAGAAAACACTCAGGATTTTGCGGACCGTATTGAGGAAGTGGAAGTG

GTTAAGGGCGATCTTTACACCCCGTATATTGACAAGGCCGAAGAGACGGTTGCCGAATTA

ACCTATCAAAAAGCCTTTGAAATTTATGGTAATCCTCTCCCAGATATTATTGATTTACGC

ATTGAAAAAGAGTTAACCTCTATCTTGGGGAACGGTTTTGCCGTGATTTACCTAGCGTCA

CAAATGCTTGTTAACCGGTCAAATGAGCGAGGCTACCTAGTTGGTTCTAGGGGATCTGTA

GGGTCTAGTTTTGTGGCCACCATGATTGGGATTACCGAGGTTAATCCTATGCCGCCTCAC

TACGTTTGCCCGTCTTGCCAACATTCTGAATTTATCACAGATGGGTCAGTTGGATCGGGC

TATGATTTGCCTAATAAACCTTGTCCAAAATGTGGCACCCCTTATCAAAAAGACGGCCAA

GATATTCCCTTTGAGACCTTCCTTGGATTTGACGGGGATAAGGTGCCCGATATTGATTTG

AACTTCTCTGGTGATGACCAACCCAGTGCCCATTTGGATGTCCGAGATATTTTTGGTGCT

GAGTATGCTTTTCGTGCTGGAACCGTAGGTACCGTAGCAGAAAAAACAGCCTATGGATTT

GTCAAAGGCTATGAACGCGACTATGGCAAGTTCTATCGTGATGCTGAGGTGGATCGTCTA

GCAGCAGGTGCTGCTGGTGTGAAACGAACGACTGGGCAGCACCCTGGGGGGATTGTTGTT

ATTCCTAATTACATGGATGTTTATGATTTTACCCCCGTGCAATATCCAGCCGATGATGTA

ACGGCTTCTTGGCAGACAACTCACTTTAACTTCCATGATATTGATGAAAACGTCTTGAAA

CTTGATATCCTAGGGCATGATGATCCGACCATGATTCGTAAACTTCAGGATTTATCGGGC

ATTGATCCTATTACTATTCCTGCTGATGATCCGGGAGTTATGGCTCTCTTTTCTGGGACA

GAGGTTTTGGGCGTTACCCCGGAACAAATTGGGACACCGACTGGTATGCTAGGCATTCCA

GAATTTGGAACCAACTTTGTTCGCGGCATGGTTAATGAGACGCATCCGACCACTTTTGCG

GAGCTTTTGCAGTTGTCTGGACTATCTCATGGAACCGATGTTTGGCTTGGTAATGCACAA

GATTTGATTAAAGAAGGCATTGCGACCCTAAAAACCGTTATCGGTTGTCGTGACGACATC

ATGGTTTACCTCATGCACGCTGGCTTAGAACCGAAAATGGCCTTTACCATTATGGAGCGT

GTGCGTAAGGGCCTCTGGCTAAAAATTTCTGAGGAAGAACGTAATGGCTATATTGATGCC

ATGCGAGAAAACAATGTGCCCGACTGGTACATTGAATCGTGTGGAAAAATCAAGTACATG

TTCCCTAAAGCCCATGCGGCGGCCTATGTCTTGATGGCCCTTCGGGTAGCTTATTTTAAG

GTGCACCACCCCATTATGTATTATTGTGCTTATTTCTCTATTCGTGCGAAGGCTTTTGAA

TTAAAAACCATGAGTGGTGGCTTAGATGCTGTTAAAGCAAGAATGGAAGATATTACTATA

AAACGTAAAAATAATGAAGCCACCAATGTGGAAAATGACCTCTTTACAACCTTGGAGATT

GTCAACGAAATGTTAGAACGCGGCTTTAAGTTTGGCAAATTAGACCTTTACAAAAGTGAT

GCTATAGAATTCCAAATCAAAGGAGATACCCTTATCCCTCCATTTATAGCGTTAGAAGGT

CTGGGTGAAAACGTGGCCAAGCAAATCGTTAAAGCTCGTCAAGAAGGCGAATTCCTCTCT

AAAATGGAATTGCGTAAACGAGGCGGGGCATCGTCAACGCTCGTTGAGAAAATGGATGAG

ATGGGTATTTTAGGAAATATGCCAGAAGATAATCAATTAAGTCTTTTTGATGACTTTTTC

TAGATTATCATGATGGTAGTCATATGCAAAGTCATAAAAGAAGAGGGACAAGATCACTTT

CTAATCTCTCTGATACTAAACTGTGACATACCGTATAAAGCGAGGAAATCAAATGAGTCA

TTTAGATAAGAACACCGCTTTGAAAGCCATGGTGGTGTTTCGTAAAGCTCAGCGAACGCT

AGATGCTTTTGGGGCTGATATTTTTAAAAAGGCAGATTTAACAGCCACGCAGTTCAGTGT

GCTTGAAGTACTATATACTAAAGGCTGTATGCGAATCAATCACTTGATTGATTCTTTGTT

AGCGACTTCGGGGAATATGACTGTTGTACTAAATAATATGGAACGTAATGGCTGGATTAG

CAAGTGCAAAGACAAAACTGATAAAAGAGCCTATGTGGTTACCTTAACAGATAAGGGTAC

TCGCTTGATTGAAGCTGTTTTGCCTAAACACGTCGCACGGGTGGAAGAAGCCTTTGCTGT

TTTGACTGAAAAAGAACAACTTTGTCTGATTGAACTTCTCAAAAAGTTCAAACAATTATA

AACAAATCTCTTTACAGCTCTTCTTAGAGAAGATATAATTACTACATAGTGATAAATTTC

AAAAAGAAGTATCAATGAAATACCACTTTTAGAAAGCTTTAAAACGATCTTTAAAAAAGA

GGACAAAGAGATGAAACATATTTTATTTATTGTTGGCTCGCTTCGTGAAGGGTCTTTTAA

CCATCAATTAGCGGCTCAAGCACAAAAAGCTCTGGAACATCAAGCAGTTGTAGCTTACTT

AAATTGGAAAGACGTTCCTGTTTTGAATCAAGATATCGAAGCTAATGCACCTTTACCAGT

TGTTGACGCTCGTCAAGCTGTTCAGTCAGCGGATGCTATCTGGATTTTTACACCAGTTTA

CAACTTCTCTATTCCAGGTTCTGTTAAAAACCTGCTAGACTGGTTGTCTCGTGCTCTTGA

TTTGTCTGATCCGACGGGCCCATCTGCTATTGGCGGTAAGGTGGTTACGGTCTCTTCAGT

TGCAAATGGCGGGCATGATCAAGTATTTGATCAGTTTAAAGCACTATTGCCGTTTATCCG

AACTTCAGTAGCAGGAGAGTTTACAAAAGCAACTGTGAATCCTGATGCCTGGGGAACAGG

AAGGCTTGAGATTTCAAAAGAGACAAAAGCAAACTTGCTATCTCAGGCAGAGGCTCTTTT

AGCGGCTATTTAGACTAACAAAAAAAGGCTAAAGGGCCTTTTTTTGTTAGTCTAAAATCA

AGAGCTCTTCTTTGGTTTCAAAGGGGTTTTTAGCATTGATCCGATCGTAGAACAAAACAC

CATTGATATGGTCAATTTCATGTTGAACCACAATAGCGTTGTATCCCTTTAATTTGATGC

GATGCTGTTGTCCTTCTTTATCATAGTAGTCTACTGTAACACGAGCATGGCGAACCACAT

ACCCTTCAACAACACGATCGACAGATAAACAACCTTCACCATCAGAAAGGGCTGCATCTT

GAACAGAATGAGAAACGATTTTAGGATTATACAGGACTTCTTGCCAGCTATAGGCTTCTT

TAGGAGGATTTCCTTCTTTATCAGGAAGATTAGGAACGAGCACAGCGATGATACGTTTTG

ACACATCAATCTGAGGAGCTGCTAGACCAACACCGGCCCTAAGACCAAGTTTTTCCGCCA

TTACAGGATCTTGAGAATGCTTCAAAAATTGCATCATCTTTTCTCCTAATAGGATATCCT

CATCACACAAGGGTAAGCTCACTTCTTTGGCGACAGCTCTTAAAGTTGGATTCCCTTCAC

GAATAATGTCATCCATGGTAATCAGATGACTAGGTTTAATTAATTTATCTTGTGCAGACA

TGTCTACTCCTTTACTTTTTTTTTAACTGGATTTACTATATCACAAATCATTTCTTTTTT

AAAGAAAAGTCAGGTAGTGGTCAGAGTGATTGCTATTATAAGTTAATGGGCTGCATACCT

CAGAGGTCTAGTTATTAACACTTAATTGAGTTAGAGCGTCTAAATCGGCAATACGACAGG

TTTTGAAGCAGATGCGTTCGATAATCCTCTGAAAAATGAGCTGCTTAAGTACGTGCTTAA

GATGGCGATCACTTATTCCAAAACGGTTTGCTAATAATGTAAAGTTCAGATGAACCTCTT

GGTTGGCTTGCGCTTCAAGGATATAGGAAGCAAAGCGCTCTTTGACGGTATAGGTAATGT

TTTGTTGGCAAACATTTTGTGTCAACTCTTGACCTATGTCATAAAGGCAGGTTGCATCGG

TTAATAAGCTGTGTTCATGGATTAGGGATTGGGTTTGTTAATACTAGAGCAATTAATATT

ATCAGTGAACACTTTGTTGGAAAGAAATGAATGACTATGCTAGGTTACCGCTTATCAGCT

GAAGTGCGGGGATCAGCCATCTTACCTCTTTTAGCTGGTTATCTTATTAGGCAAGCAATC

GCACAGGTTTATTCTGATTTAAAAACAGCCTCTTGGCTATTTGGCCTTGCTAGTTCTGTT

GCTTAAGGTGGTTTGGATGTGAGAGCTTAGAATAATAGGATAACGGTTGACCTAATATTA

TGAATCTGCTAGAATAGAGTGGTCGAAAGACAAACCAACTTTTTCTTGGTTTTAGGAGTG

CCAATCCTAAGACTCGGATTAAGCAAAAAAAAGGAGAATATATTATGGCAATCTCAAAAG

AGAAAAAAAATGAAATCATTGCTCAATATGCACGTCACGAAGGTGATACTGGTTCAGTAG

AAGTTCAAGTTGCAGTTCTTACATGGGAAATCAACCACTTGAACAACCACATCAAAGAAC

ACAAAAAAGACCACGCTACTTACCGTGGATTGATGAAGAAAATCGGTCACCGTCGTAACT

TGTTGGCATACCTACGTCGTACAGACGTTAACCGTTACCGTGAGTTGATCCAATCTCTTG

GACTTCGTCGTTAATTTCAAGTTTAAAAAGTCCTCTGCTTATGCAGTGGGCTTTTTCATT

TTTGACATGTTGTTTGTTAACTTACACATTTGGCTAGAATGGTCTAAGGTTGTTTTTTTA

GCAATTGATGATTTTATCTAATAACAAAAGGTATCTCAGTGATGAGGTATCTTTTGTTGT

ATCATGAAGAAAAAGGGAATCAATTCCCAGATTTGTCTATTTTTTGCCACCTTTTGAGGA

ACTTGTTACAATGATATCACCTGTTGAGTCCAATTGCGGTCAAAAATGACGAGAGACAAT

TGGACTCAATTTGTCTTGAAAATGAAAACGCTTCATTTTATACTGACATTATAGGAGGAG

ATGACATGCTATCTCATGAGCTCATCAGAAACTATCAACTCTTTTCTAAATATAAAGGAC

ATTCACTGGAGGCATTTGAATCCATGTTAAAGACAAGCAAACGTCATATACTGGCAGATA

TTGCTAAAATCAATGACACGTTGTCACTCTATCAGTTACCCCTTATTGCTCTGGACAGGC

AGCTGGTTTATCCGCCAGACCTTACTGAAAAGGACTTGTTGAATCGCATGCTACCTACCT

TAGACGACTATCTCTTTCAAGATGAGCGTCTGGACATGATTATCATTTACATCATGATGG

CTAAAGAATTTATCTCCATTAACCACTTGGAAAGCTTGTTACGGCTCAGCAGAAATTCCG

TTATTGCTGATTTAAACTTGGTGCGTGATCAGGTACAAGCTTTTCAGGTAACTTTAGCTT

ACAATCGCCAGGATGGTTATTTTTTTGAAGGAGAACCCTTAGCCTTGCGGCGTCTCTTAG

AATCAGCGGTCAGTTCTCTTTTGCAAGTTACATCAGGACCTTGGGTGTTCAGTTACTTAT

TGCATGAACTTGGTTTGCCCGACCAGACAAAGGTGATGGCAGCAACGTTGGAGGAACTGA

GCAGGGAGAACCACTTAACTTTTATTTCGGAGAAATTGAGGGATTTGATTTATTTTTTCT

GCCTTCTCGCTCATCGACCATTTTCACGGAATGTAAGGGCTGAAGCAGTGGATACTTTTC

CTTTAGCTTCGCCAGCTGTTGAAACGATGGTGGACCAGTTATTGGTCAACTTCCCCAGTC

TAACCGAAGAAAAATATTTGGTTCAATCTAGGTTACTTGGTTGTATCCAAGGTGACTTAG

AGTTAGTCTTTCAGCAACCTATTTATGACATTATGGAAGAAATCATCAATTCGGTGGCGG

TTAACACGGGATTGTCTATCACCGATACCCCAGAACTTCGTCAGAACCTATACAGTCACC

TTTTGCCAGCTTATTACCGACTTTACTACGACATTAACTTGACCAATCCTTTGAAGGAAC

AAATCAAACAAGATTATGAATCTCTCTTTTACTTGGTCAAGCGCAGCCTTTCTCCTCTAG

AAAAACAATTGGGGAAACTAGTTAATGAAGATGAGGTTGCTTATTTTACCATTCATTTTG

GGAGATGGTTGCAGGCCCCTAAGAAGAGGCCGAGCAATCAGCTGGTAGCCTTATCTGTTT

GCCCTAATGGCATTAGTTCGTCTCTGATGTTGGAGGCAACCTTGAAGGAACTTTTTCCAC

AGCTACAGTTTATTAGGATTCACCAGCTGGACAAAATAAAGTTGTTGGATCCAGCTTCCT

TTGATTTGATTTTTTCAACAGTAGCTTTTGACTGTGCTAAACCTGTTTATGTGACGCAAG

CTTTGATGGGGCCTGTTGAGAAAATGATGTTGAAAAAGATGGTCTGTGATGACTTTCATC

TTCCCTTGTCAGAGCAATTCGCTTTGGATGACCTGTTGAGTATTATTCATAAACACACCA

CGATTACTAATAAAGAAGGACTTGTTAGTGATTTATCCCGTTACCTGATTGGTAACCATT

TAACGATTGAAAAAGGAGGTCTAGGACTATTGGACTTGTTAACAGCAGATTTTATTAGGC

AGGCTGATGCTGTTTCGGATTGGCAGGAAGCGATTCGTTTGGCTGCTCAGCCTTTACTAG

AACACCAGATGATTGAAACATCTTATATCGATGGCATGATTGATTCAGTCAATGAGCTTG

GGGCCTATATTGTTTTAACCCCTAAGGTGGCTGTTCCTCATGCGGCGCCTGAAAAAGGAA

CGCGGCAGTTAGGCATGTCTCTCTTACAACTAAAAGAACCTGTCAGCTTTGATTTGAAGC

AAGAAGGTGATCCAGACAAGCAAGTGCAATTGATTTTTGTTTTGTCTGCTGTGGATTCTA

GTTCACATTTGAAGGCTTTGCAGGAACTGTCGTTGATTTTAGATGATGATGAACATATTG

AGCAATTAATTGAGGCTAAGAATACTGAGGAAATAATGAGTCTGATTAGCCATATGATTG

AAAAAGGAGATGAATCACATGATTAAAATTGTAACGGTTTGTGGAAACGGTATTGGTAGT

AGCTTGTTACTTCGCATGAAAGTAGAAGCTATCGCATCTAGTTTGGGTATTGATGTGGAT

GCAGAATCCTGTGATTCCAATGCGGCTGTTGGAAAGGGTGCAGATTTGTTTGTTACCGTT

AAAGAATTTAAAGATATTTTTCCAGAGGATGCCAAGGTTTGTATCGTTAAAAGCTATACC

AATCGTAAAAAAATTGAAGAAGATTTGGTTCCGGTTCTCAAGGAAATGAGTGGCAAGGAA

TAAGCCTTTAAAGGAGATAGGAAGATACTATGGAAGCATTATTATCATTTATTCGAGATA

TTTTAAAAGAACCTGCATTTTTAATGGGCTTGATTGCCTTTGCAGGGTTGGTGGCTTTGA

AAACACCTGCTCATAAGGTGTTGACAGGAACCTTGGGGCCGATTTTGGGATACCTTATGC

TTGCTGCAGGGGCGGGTGTTATTGTGGCCAATCTGGACCCTCTTGCCAAACTCATTGAGC

ACGGTTTTAGCATCACTGGCGTAGTGCCAAATAATGAAGCCGTCACTTCTGTCGCTCAAA

AGATTCTGGGTGTGGAAACCATGTCCATCTTGGTGGTTGGGTTATTGCTCAATTTGGCTT

TTGCTCGTTTTACCCGCTTCAAATACATTTTCTTAACAGGACATCACAGCTTCTTTATGG

CTTGTCTCTTGTCCGCCGTGCTTGGAGCTGTTGGTTTCAAAGGAAGTCTTTTGATTATCT

TAGGTGGGTTCCTCTTGGGAGCTTGGTCAGCTATTTCGCCAGCCATTGGTCGACAGTATA

CGTTGAAAGTGACTGACGGAGATGAAATCGCCATGGGACACTTTGGTAGTTTGGGCTATT

ACCTTTCTGCCTGGTTTGGTAGCAAGGTTGGCAAAGACAGTAAAGATACCGAGGACCTTC

AGATTTCTGAAAAATGGAGTTTCTTACGCAACACCACCATTTCAACAGGACTTATCATGG

TGATTTTCTACTTGGTGGCAACAGTGGCTTCTGTTTTGAGGAATGCTTCAGTAACAGAAG

AATTAGCAGCAGGTCAAAACCCATTTATCTTTGCTATTAAGAGTGGTCTTACCTTTGCGG

TTGGTGTGGCAATTGTCTACGCCGGTGTTCGCATGATTTTGGCTGACTTGATTCCAGCCT

TCCAAGGTATTGCTAACAAATTAATTCCAAATGCTATTCCAGCTGTAGACTGTGCGGTAT

TCTTCCCTTATGCGCCAACCGCTGTTATCATCGGTTTTGCGTCAAGTTTCGTTGGTGGCT

TACTTGGGATGTTGATTTTAGGAGTTGCAGGTGGTGTCCTGATTATCCCAGGTATGGTGC

CTCATTTCTTCTGCGGTGCAACAGCAGGGATTTTTGGAAATTCAACAGGTGGTCGTCGCG

GTGCAATGATTGGTGCTTTCGCTAATGGCTTATTACTCGCCTTCTTGCCAGCCATGCTTC

TACCTGTACTTGGTAAACTTGGTTTTTCAAACACGACCTTTGGAGATGTGGATTTCGGTG

TTTTAGGGATTTTACTGGGCCGCTTAGGAACGTCGATTGGTCAAATCGGTATTTACTTGG

TGGTTGCTGTGCTTGCTATTGTATTGGTGCTTCCAAACTTTCTGTCAAAATCTCAAGAAG

CCATCAATAATGTTTCAGAAGAAATCTAAAAGGAGGATATGATGACAAAAAAACTAAACG

TTAAAGTCTTTTCAGACGGTGCGGTCTTAGAGACCATGTTAAAGGATTTACAAACAGGTT

TGGTCACAGGCTTTACAACCAATCCAAGTTTGATGAAAAAAGCAGGCATCAGTTCTTATA

TTGGCTTTGCCAAAGAAGTGCTTGCCAAAATTACAGACTACCCAGTGTCCTTTGAAGTCT

TTGCGGATGATCTCGCAAGCATGGAAAAGGAAGCTGAGAAAATTGCCAGTCTAGGTGACA

ATGTCTATGTGAAAATTCCAGTCACCACTTCAACAGGAGAATCAACCTGTCCCCTCATTC

AAAAGTTGTCAGCCAAAGGCATTAAGTTAAACGTCACAGCCATTTTTACAATTGAGCAGA

CTCAAGCTGTGGTTGATCATTTAACCGCTGGAGTTCCAGCTATTGTGTCTGTTTTTGCTG

GTCGTATTGCAGATACCGGCGTTGACCCAATGCCAATCATGGAAGAAGCTCTACGCATTT

GTCGTCAAAAAGAAGAGGTCGAATTGTTGTGGGCTAGTCCTCGTGAAACTTATAATATCT

ATCAAGCTGATCAGCTAGGCGTGGACATTATCACTTGTACCACTGATTTGATTGCCAAGT

TGCCTCTTCAAGGAAAAGATTTAGAAGACTATTCCCTTGAAGCCGTGCAAATGTTTCTGA

AAGACAGTACCAGCCTCGGCTTTAAAATTTTAGAGGATGCCAAACATTAATATTAGGAGA

CGAAAGCAGGTATACCAACCTGCTTTTTTTGCTTTGTTAAAGAGTATAAGTTTCGTTTAA

TATTTTTCAAGGACATTTCAAGAGATTGCGATCTGACAGTGAAATTTCTGCTATTTTATG

GTAAAATGTAGCAGATACGTCGTTTGATACAAAGAGGAGCGTGTGACAAGCGTGGCAGGT

TGCTTTTTGTAGCAAAACAACGTATAACAATTGAATCTAAGCAGAGAACAGGTACCTCTT

TACCCCTCTGAAAATTAAAAGGAGAATATATGTCAAAACAAACCTTTACAACAACATTTG

CAGGGAAACCCCTTGTTGTTGAAGTTGGTCAAGTCGCTAAGCAAGCCAATGGGGCAACCG

TTGTTCGTTATGGCGACTCAACTGTCCTGACCGCAGCTGTCATGTCTAAGAAAATGGCAA

CGGGTGATTTCTTCCCTCTTCAAGTTAATTACGAAGAAAAAATGTATGCCGCTGGTAAAT

TCCCAGGAGGTTTCATGAAACGTGAGGGACGCCCTTCAACAGATGCGACCTTGACAGCTC

GCTTGATTGACCGCCCGATTCGTCCCATGTTTGCCGAAGGTTTCCGTAACGAAGTACAAG

TGATTAACACTGTCCTTTCTTATGATGAAAATGCTAGCGCTCCAGTGGCAGCGATGTTTG

GCTCATCTCTGGCCTTGTCCATCTCAGATATTCCATTTAATGGACCGATTGCAGGTGTTC

AAGTTGGCTATATTGATGGCGAGTTCATCATCAACCCAGATAAAGAACAAATGGAAGCGT

CGCTCTTAGAATTAACCGTTGCTGGTAGCAAAGAAGCCATCAACATGGTTGAATCTGGTG

CCAAAGAATTATCAGAAGACATCATGCTTGAGGCTCTTTTAAAAGGTCACCAAGCTATTC

AAGAATTAATTGCCTTCCAAGAACAAATCGTAGCAGTTGTTGGTAAAGAAAAAGCTGAGG

TTGAACTGTTACAGGTTGATGCAGACTTACAAGCTGATATTGTGGCTAAGTACAATGCTC

AACTGCAAAAAGCTGTTCAAGTTGAAGAGAAAAAAGCGCGTGAAGCAGCCACAGAAGCCG

TTAAAGAAATGGTTAAGGCTGAGTACGAAGAGCGTTACGCAGAAGACGAAAACCTAGCAA

CCATTATGCGTGACGTGGCAGAAATCCTTGAACAAATGGAGCATGCTGAAGTGCGCCGCC

TCATTACAGAGGACAAGATTCGTCCTGATGGTCGTAAGATTGACGAAATTCGTCCGCTTG

ATGCTGTGGTTGACTTCTTGCCAAAAGTACATGGATCAGGTCTCTTTACACGTGGTCAAA

CTCAGGCGCTATCAGTTTTGACCTTGGCACCAATGGGAGAAACTCAAATCATTGATGGTC

TGGCTCCAGAGTACAAAAAACGCTTTTTACACCACTACAATTTCCCGCAATATTCAGTTG

GTGAAACGGGTCGTTACGGAGCTGCTGGTCGTCGTGAAATTGGACACGGTGCCTTAGGTG

AACGCGCTCTTGAACAAGTCTTGCCAAGCTTAGAAGAATTTCCTTATGCCATTCGTTTAG

TTGCCGAAGTTCTAGAATCTAACGGTTCGTCTTCTCAGGCTTCTATCTGTGCTGGTACCC

TTGCTCTGATGGCTGGTGGTGTACCAATCAAGGCTCCTGTGGCAGGGATTGCCATGGGTC

TCATTTCAGACGGGACGAATTATACAGTCTTGACCGATATCCAAGGTCTTGAAGACCACT

TTGGAGATATGGACTTTAAAGTGGCAGGAACTCGTGAAGGGATTACAGCCCTTCAAATGG

ATATTAAGATTGCAGGAATTACCCCTCAAATCCTAGAAGAAGCTCTTGCACAAGCTAAAA

AAGCTCGCTTTGAAATTCTTGATGTGATTGAAGCAACTATCGCTGAACCACGTCCTGAGT

TAGCCCCAACTGCACCAAAAATTGACACCATCAAGATTGACGTAGACAAAATCAAAGTGG

TTATCGGTAAAGGTGGCGAAACTATTGATAAGATTATTGCCGAAACGGGTGTCAAAATTG

ATATTGACGACGAAGGAAATGTCTCTATCTACTCAAGTGACCAAGCTGCCATTGACCGCA

CTAAAGAAATTATTGCAGGTCTTGTTCGTGAAGCCAAGGTAGGCGAAGTTTACCATGCCA

AGGTTATCCGTATCGAGAAATTTGGTGCCTTTGTCAACCTCTTTGACAAGACAGATGCAC

TTGTTCATATTTCTGAAATTGCCTGGACGAGAACAGCTAACGTGTCAGATGTCCTTGAAG

TGGGTGAAGACGTTGATGTTAAGGTCATTAAGATTGATGAGAAGGGTCGTGTGGATGCCT

CAATGAAAGCTTTGATTCCACGCCCACCAAAACCAGAGAAAAAAGAAGAAAAACATGACT

AAATCAAATGAATTAGATATTCGCTTAAGAGCTTTTATCAATGCGCCTGATAACTTTTTG

GATAGCCTTGCCCTTGTCAACGCTTTCCATAATTTTCCTGTCTGGGCTGCTAAAGAACCC

TACGTGATAGAAGTCGAAGGTGTCAAGGTTACCCCTGTTTTTACCGATAAGGAAGATATG

GCTCGCTTTAAAGAAGAACAAAAGAGTGCTCAAAGTCAGTATTGGCTGGAGCGTTCAGCT

CTTGCAGTGCTAGAAGAGGTCATTACATCTGGTGCAGCAGGTCTTGTCTTTAACCTTAAG

AAAAAGGGAGATTTTGGGAATTCTACCATTTTCAAAAGCAGTGACATGATTCAATTTATG

AACCATTACACCACTGTGTTAAATACGCTTATGAGTGATGACAATGTGGCGGCAGATACG

ATGGAAAAGGTTTACCTTGTTCCCGCCTTTGTTTATCCTAAAGATGATAACCATTACGAC

CGCCTTTTTCCTACCATGTCAACGCCTGAAGGAAAAAGCTATGTTCCCGCCTTTTCAAAT

CTTCAAAGCTTTGCGAAATGGTACAACCAAGACGATTTTGGAGGCCTTTTCAGGAAAGCT

GAAGGAGTGATTCTGACTTGGACGATTGATGATATTTATCAACCAAGAAATGGTGAAAAC

GAGCTTGATGAAACGTTCGGTGTCGCCATCAATCCTTTTGATGACCAACAAATTCTTGTT

GATTGGTCAGAATTAGATAAGTCGTAGGAGTTAGTATGGGTTGGTGGAAAGAAAGTATAG

CTATTGTTAAAGCTCTAGACCCAGCTGCTCGTAATAGTCTTGAGGTTATCCTAACTTATC

CAGGCATCAAAGCCCTAGCTGCTCATCGTTTGTCTCATTTTCTTTGGCGACATCATTTTA

AATTACTAGCCAGAATGCATAGCCAATTTTGGCGATTTTGGACACAAATTGAAATCCATC

CTGGAGCACAAATCGCTCCTGGAGTCTTCATTGACCATGGTGCTGGTCTTGTTATTGGAG

AGACAGCAATTGTTGAAAAAGGTGTGATGCTTTATCATGGGGTGACCCTAGGTGGAACCG

GAAAAGATTGTGGCAAGCGTCATCCAACGATTCGACAAGGTGCCTTAATTTCGGCACATG

CCCAAGTGATTGGACCTATTGACATCGGAGCAAATGCTAAAGTAGGGGCAGCAGCTGTTG

TGTTATCAGATGTTCCTGAAGACGTGACAGTTGTAGGTGTGCCAGCTAAGATAGTACGAG

TGCATGGGCAAAAAGATAATCGTCAAATTCAAAGTTTACAAAAACAACGAGAGGTCTCTT

ATCAGTTGTCAAAATGATCGGAAAATAACAAAAAGAAGCACTGATGACTTCCAATAAAAT

ATTTTGGGACTTTTGTGATACTTTTTCTCAGGTGGTGCTTACGGCAGCGACTTCCTTTGG

AATTCCATGCCTTAGTTTTGAGCCTAAGATCTCAAAAATCCGTATGGGCATCACTGTCAG

TATTGATGCCACTTTTACCACAACAAAAAGCAGCTGTTTTGGCTTACCCCACATGCAGAA

ATAGGAAACCTGATGTCAAATCAGCAGATCTATTCATGCTTTTTGAGCATAAAAGTGGGT

TTGTCTACTTTCTGAAGCCTTCACAGGTTGTGAAGGTTTTTCGTTAATAAAGGAGCCTCA

TGAATAAAATAAGAGTTGGGCTGTATGTCATTATGAGTTTGTCTATCCTTGTTTCTAGTA

TTACACTGGTGGCTAGTTTAATCTTTTTACTGAAACAAGACATGGTTAAAGCGATTATAT

ACTTCATTTTGACCCTATTTTGGTTAATAGGTTTGGTTATTAATATCAAATTGTTGAAAA

GGTGGAACAGCATTTCAGAAAAAGACTTAGGATATCAAGATGACAACTAATGCCAGGCTA

TTGAATAAGGAGTTTACATGATTAAAATTTATGATACCATGACCCGTTCGCTCCACAAGT

TTGTACCTTTGACTGAAAATACAGTCAATATGTACGTTTGTGGACCGACGGTCTATAATT

ATATTCATATTGGAAATGCTAGATCAGCGGTCGCTTTTGACACCATTCGGCGTTATTTTG

AGTATACTGGCTACCAGGTCAATTACATTTCCAATTTTACCGATGTCGATGATAAAATTA

TCAAGGCTGCTACTCAAGCAGGTGTTTCTCCCAAAGAATTGTCAGATCGCTTTATTGCAG

CTTTTATAGAAGATACCAAGGCACTTGGTGTTAAGCCAGCCACACAAAATCCTCGTGTTA

TGGATTATATAGCAGAAATCATTTCATTTGTTGAAAGTCTCATTGAAAAAGATTTTGCTT

ACGAAGCAGATGGAGATGTGTATTTCCGCGTGGAAAAGTCAGAGCATTATGCCAAGCTAG

CTAATAAAACCCTGTCAGAACTTGAAGTTGGAGCCAGTGGTCGAACAGATGCTGAGACAG

CTTTAAAAGAAAATCCACTGGACTTTGCTCTTTGGAAATCAGCTAAGGCAGGTGAGGTTT

CTTGGGATAGCCCCTGGGGGTTTGGTCGTCCAGGCTGGCACATTGAATGTTCTGTGATGG

CTACTGAAATTCTTGGTGATACCATTGATATTCATGGTGGCGGAGCTGATTTGGAATTTC

CTCATCATACCAATGAAATTGCCCAATCTGAGGCCAAAACAGGCAAGACTTTTGCTAACT

ATTGGATGCACAATGGGTTTGTCACTGTTGATAACGAAAAAATGTCCAAGTCACTAGGTA

ATTTTGTGACCGTTCATGACATGTTACAAACCGTTGATGGTCAGGTTTTGCGATTCTTTC

TTGCGACACAACAGTACCGAAAACCAATTAACTTCACTGAAAAGGCTATTCATGATGCGG

AAATCAATCTGAAGTATCTTAAAAACACCTTGCAACAACCGCTGACAGAAACTGCAGATG

AGCAAGAGCTAAAACAGTTTGTAATAGCTTTCCAAGACGCTATGGACGATGATTTCAATA

CAGCAAATGGGATTACCGTCGTATTTGACATGGCCAAGTGGATTAATTCTGGCTCCTATA

CAGAACCTGTTAAAAGCGCTTTTGAGAAAATGTTAGCTGTTTTTGGCATTATCTTTGAAG

AAGAGGTGCTTGAGGTTGACATTGAAGCCTTGATTGCTAAGCGACAAGAAGCACGCGCGA

ATCGTGATTTTGCCACAGCTGATGCCATTCGAGATCAGTTAGCAGCTCAAGGCATCAAGC

TGCTAGATACCAAAGATGGTGTGAGGTGGCTGCGTGACTAATCCAGTTGATGTGAATTTG

ATTAATGGTATTGCCCTAGCCTTTGAAGGGGATGCGGTTTATTCCTGCTATGTTCGTCGT

CATCTCATTTTTCAAGGTAAAACGAAACCTAGCCAGCTACACCGTTTAGCAACGAGGTAT

GTTTCTGCTAAGGCACAAGCCAACTTGATTCAGGCTATGTTAGAAGCGCAGCTATTGACC

GAAAAAGAAGAAGACATCTATAAGCGTGGTCGCAATACCAATAGCCATACTAAAGCTAAG

AATGCCGATATTATTACCTATCGTATGTCGACAGGTTTTGAAGCCATTATGGGTTATCTG

GATATGATGGGCCAAAAAGAGCGGTTAGAAGAATTGATCAGATGGTGTATTGAGTATGTA

GAAAAGCAACAATTGATATCCTCATAAGAGTCCCCTAGGAGACTCTTATTTTCATAGATC

TGAAGTAAAGGTAGAGAGCCATTAAGTCTTTTTATGACATCAAAAAGACATAATGATATA

AAAAAGATACATTTGTAACAAAAAAGACATAAAAATTATGAAATCATTTTCGAATAGTTT

ATAATGAGAGGGTAAGACTAATTTAAAGAGGAGATATTTATTATGACAACTATGCAAAAA

ACAGTTAGCTTATTATCACTAGCTTTACTTATTGGTTTGCTGGGGACTTCTGGCAAAGCT

ATATCTGTGTATGCACAAGATCAGCACACTGATAATGTTATAGCTGAATCAACTATTAGT

CAGGTCAGTGTTGAAGCCAGTATGCGTGGAACAGAACCTTATATTGATGCTACAGTCACC

ACAGATCAACCTGTCAGACAACCAACTCAGGCAACGATAACACTTAAAGACGCTAGTGAT

AATACTATTAATAGTTGGGTATATACTATGGCAGCGCAACAGCGTCGTTTTACAGCTTGG

TTTGATTTAACTGGACAAAAGAGTGGTGACTATCATGTAACTGTCACCGTTCATACTCAA

GAAAAGGCAGTAACTGGTCAATCAGGAACTGTTCATTTTGATCAAAACAAAGCTAGAAAA

ACACCAACTAATATGCAACAAAAGGATACTTCTAAAGCAATGACGAATTCGGTCGATGTA

GACACAAAAGCTCAAACAAATCAATCAGCTAACCAAGAAATAGATTCTACTTCAAATCCT

TTCAAATCAGCTACTAATCATCGATCAACTTCCTTAAAGCGATCTACTAAAAATGAGAAA

CTTACACCAACTGCTAGTAATAGCCAAAAAAACGGTAGCAACAAGACAAAAATGCTAGTG

GACAAAGAGGAAGTAAAACCTACTTCAAAAAGAGGATTCCCTTGGGTCTTATCAGGTCTA

GTAGTCAGTTTAGCTGCAGGTTTATTTATAGCTATTCAAAAAGTATCTAGACGAAAATAA

AAGAGTCTATGCATTTATTTTGAAGCATTCATGGTATAATAAACTCATGGAAGATAAAGA

TACTATTGAAACAAACGATATCGTCTATGGTGTTCATGCCGTTACAGAAAGCCTTCAAGC

AAATACAGGAAATAAGCTTTATATCCAAGAGGATTTAAGAGGAAAGAAAGTGGATAACAT

CAAAAGCTTAGCGACACAAAAAAAGGTCGCTATTTCATGGACGCCTAAAAAAACCTTGTC

ACAAATGACTGATGGAGCTGTGCATCAAGGTTTTGTTCTGAGAGTATCAGCTTTTGCCTA

TACTGATGTTGATGAGATCCTCGAAATAGCAGAGCAAGAAGCAAATCCTTTGATTCTTAT

TTTAGATGGTCTGACAGATCCTCATAATTTAGGATCGATTTTACGGACAGCTGATGCTAC

AAATGTATGTGGAGTGATTATTCCTAAACATCGTTCTGTTGGTGTGACTCCAGTGGTCTC

AAAGACGTCTACAGGTGCTGTTGAACATATTCCGATAGCGAGAGTAACTAACCTTAGTCA

AACTCTAGATAAATTGAAAGCAAGAGGATTCTGGATTTTTGGCACAGACATGAATGGAAC

ACCGTCTGATTGCTGGAACACTAATGGTAAACTTGCTTTAGTCATTGGTAACGAAGGCAA

AGGCATCTCTACCAATATCAAAAAGCAAGTTGATGAAATGATTACGATTCCTATGAATGG

TCATGTACAGAGCTTAAATGCTAGCGTAGCGGCAGCTATTCTCATGTATGAAGTTTTCCG

AAATAGGCGCTAAATTATGAAAAAACGGATATTATTAGTAGATGGGTATAATATGATTGC

CTTTTGGCAATCAACCCGTCAGTTGTTTAAGACAAATCAGCTTGATCAGGCACGTAACAC

ACTTTTAACAAAACTTAATCATTATGCCCATTTTGAGAATATTAATATTATTTGTGTTTT

TGATGCCCAATATGTACCGGGGTTAAGGCAACGATATGACCAGTATTATATCTCAGTGGT

ATTTACAGAGGAAGACGAAACAGCAGATAGCTACATTGAGCGCATGGCAGCAGAGTTAAA

TACGGCTATACATATGGTAGAAGTAGCTACGAGTGATTTAAATGAACAATGGACGATTTT

TTCTCAAGGAGCTCTTCGTGTCACTGCAAGAGAATTAGAGCAAAGAGTTCATACTGTCAA

AGCTGATTTAGATAAAATGTCTAGAGATATTGATCTCAAAACGCCTAAACTGCGGCCTTT

TGACCAAGGTCAGCTTAGTCAACTGAAGGATTTTATGTCTCAGTTAGATCGATAATGAGA

ATTACGACTCGCCAAGTGCGGGTTTTTTTGATAAAATAAAACAATAATTATTTGATTGTC

AAACTAAAAAACTGGATGAGGAGAAGTAAGAATGACCTTTACAATAATGACAGATTCAAC

CGCTGATTTGAATCAAACCTGGGCAGAAGATCATGATATTGTCCTTATAGGATTAACGAT

TTTGTGCGATGGAGAAGTTTATGAAACGGTCGGTCCTAACCGTATTAGTAGTGATTACCT

TTTGAAAAAGATGAAAGCTGGCAGTCATCCTCAAACCAGTCAGATTAATGTGGGAGAGTT

TGAGAAGGTTTTTCGTGAACATGCTAAGAACAACAAAGCACTGCTTTATCTTGCTTTTTC

TTCGGTTTTATCGGGTACCTATCAAAGCGCTCTAATGGCACGCGATCTTGTTCGAGAAGA

TTATCCCGATGCAGTGATTGAAATTGTTGATACCTTGGCTGCTGCAGGAGGAGAAGGCTA

TCTAACCATTTTAGCAGCAGAAGCCAGAGATAGTGGCAAAAATTTACTGGAGACTAAAGA

TATTGTTGAAGCAGTGATTCCTCGACTGCGCACCTATTTCTTAGTAGATGATCTTTTTCA

TTTGATGCGTGGTGGGCGTCTGTCAAAAGGTTCAGCTTTTCTAGGCAGTTTAGCCAGTAT

CAAACCTCTTTTGTGGATTGATGAGGAAGGGAAATTGGTGCCCATTGCAAAAATTCGGGG

ACGCCAAAAAGCCATCAAAGAAATGGTGGCTCAAGTGGAAAAAGATATTGCGGATTCGAC

GGTTATTGTCTCTTATACAAGCGATCAGGGTAGTGCCGAAAAGTTGCGAGAAGAGCTGCT

GGCGCATGAGAATATTAGCGATGTTCTTATGATGCCACTAGGACCAGTTATCTCAGCTCA

CGTTGGTCCTAACACCCTGGCAGTTTTTGTGATTGGGCAGAATTCCCGTTAATCCCTTAT

AAGGATTGAAGAGAAATCCTCAATCCTTTTTTGCTGCTTAAATCAATCAAGAATGTAAAC

TATATTTGACAAAATATCTAAAATAATTTATATTATGTCATATAAAGATTTAAAGGAGAA

ACGAATCATGTTTGATAGCAAACAAAATCTCGCTAAGTCACAATGGATGTTTATAATCAT

AACAGCAGTGATGGATTTGGTTGTGTTAGTAGCGACACTGCTGGCTAAAACCAGTTTTCA

ACGTTACTTAGTTGGAGGTGTAGCGGTTATATTGACTAGCTTTCTCATATTGTTGGTATG

GGGGCTAAAGACAGCAAAAAAGCTCCAAACGAGACTAGATAGCAAAGAAATACTAGATTC

TAAGGTAAGGGACGATCAGAAGCTGCCCAGATATGACGAACGCCAAAAACAGATTCTTCT

CAAAGGATACACTATTGGGTTTTGGTTTATGATTGTGGCTGTTTGGCTGTCAATCTTTCT

TCATCGCTTCTCAGAGAGACTGGTATCTGCAAGCTTCCTCTTTACATTGGCTCTTTGGGG

AGGGTTGGCAGTTCAAACGACATATTGTAACCTCAACGGGGTATCGCCATTTGTGGATAG

GCGCTTTGGAAAACAAGGAATACTGGTGGGGATGATAGCAACAGGAATGAGTTTAGTCAC

TATAGGTTTGGCGGTAAGAGAGATGATGGCAGGGCAACTTAAAATGGGAAGCTTTTTCAA

GGCTGGCGGCAGTGGATCTTTAATGGTGTTGGGCATAACGCTCCTTAGTATGGGAGCTAG

TATTTTCTATCGTCATTATCTGGATGCTAAAGAGGCAGATGAATGAAGAACCTCAAATTA

AAAGCAGCGCGTGCAGGCAAAGATTTGTCTCAACAAGCTCTAGCAGATTTGGTAGGCGTC

TCCAGACAAACCATTGCGGCTGTTGAAAAAGGTGATTACAACCCAACGATTAATCTTTGC

ATTGCCATTTGTCGAGTGCTTGATAAGACCTTAGACGACCTTTTTTGGGAAGCTGATGAT

TCCCATTAACATCAGCAGAGCAGCTCTGCCATTCCTCAAAAACGAAAGAAAAAATAATCC

AATAAAATCAGCTAAAGCACTTGACTGGAGCCTCTTAAGTTTGATATCATAGTAGGCGGT

ATTGTTTACCCCATTTGAAAGGCCCCGGAACCTTCCAAATACCTTCGATGGGACGGAACA

CCCATCACCTTGTAAACAAATATTACGAATTCGTATAGGAGAAATCATGAACAAAACAAC

TTTCATGGCTAAACCAGGCCAAGTTGAACGCAAATGGTACGTTGTTGACGCAACTGATGT

GCCACTTGGACGTCTTTCTGCAGTAGTTGCTAGCGTACTTCGCGGAAAAAACAAACCAAC

TTTCACACCACACACTGATACAGGTGATTTTGTTATCGTTATCAACGCTGAAAAAGTTAA

ATTAACTGGTAAAAAAGCGACTGATAAAGTTTACTACACTCACTCAATGTACCCAGGTGG

TTTGAAATCAATCACTGCTGGTGAACTTCGCTCTAAAAACGCAGTTCGCTTGATTGAAAA

ATCAGTTAAAGGCATGCTTCCACATAACACTCTTGGACGTGCACAAGGTATGAAATTGAA

AGTCTTCGTTGGCGGTGAGCACACTCACGCAGCACAACAACCAGAAGTACTTGACATCTC

AGGACTTATCTAAGAGGAAAGGAGCATTAAATAATGGCACAAGCACAATATGCAGGTACT

GGTCGTCGTAAAAACGCTGTTGCACGCGTTCGTTTGGTTCCAGGTACTGGTAAAATCACT

GTTAACAAAAAAGATGTAGAAGAATACATCCCACATGCTGACCTACGTTTGATCATCAAC

CAACCTTTTGCAGTTACATCAACTGAAGGTTCATACGACGTTTTCGTTAACGTTGTTGGT

GGTGGTTACGGTGGACAATCAGGTGCGATCCGTCATGGTATCGCTCGTGCGCTTCTTCAA

GTAGACCCAGACTTCCGCGATTCATTGAAACGCGCTGGCCTTCTTACACGTGACGCACGT

ATGGTTGAACGTAAAAAACCAGGTCTTAAGAAAGCTCGTAAAGCTTCACAATTCTCAAAA

CGTTAAGAACAGCTTTACATCAACGATTACAAGCATTTTGCAAGTCTCTTGCAAGGTGCT

TTTTTGTCGTTTTTTGCTTTTGATACCTTTTTTGATACCGTTTCGGAAAACTTTTGACTT

GGTTTAATACGTTTATTTAGTTATTTTTGAGATAGTTAGCAAATTGTTTGATTGATTTGT

CCTCTCCGACTGCAGTAGCATGACTGTATGTATCAAGAGTCATTTGACTACTAGCATGTC

CTAGTCTTTTGGCTGTATTTACTGGGTCAACTCCAATCTCAATCATCAGGGTTGCATGGG

TATGCCTGAAGCCTTAAGGTAGATTAGCCTAGCTTCGTAGTCTTTGGCTTCTTTGTGGAA

TATCTATTAACCAGCCCGAACTCATTTTGCTCCTAGTTAATTGGTAATCTACCAAAAAAG

CCCTAGCAAGACTGCTAAGGCTTTTAACGTGTTATTTGAATAACTTTATATAGTCGCTTT

GAGTAGGAAGCAAGTAATCAATAGCGACCCTATTTTCTTCCCCCTCGATATGGTATAGGA

CAATATAATCTTTACTTAAGGTGTAACCTTTAGTGCTGTGATAATGGCTGATTTTTGAAC

CATATTTTTCATCGGCATCAAAACCAACTTCTGGGAAAACCTCGAGTTTTTCTATGTCGC

TAATGATTTGCTCCATTTTACGCTGACCTGATGTTGAGGAGTAGTTTTGAGAAATATAAT

CACGAATTTCTTTTAGCTTCTCTAAAACATCAGGAGCATAGATAATCTGATATTTCTTAT

AGTCCAAGTTCAGCCCTCACTTCTTCAGAGGTATAAAACTTCCCTTGACGAACGTCCTCA

ATATTTTTGTTGATTTCAGCTTGAAATTGCTTGAAAAGTTTTTCTTTCTCTAACTCCTCT

TCCGTCAATAAGTCAACCTCGTTTGTGACAACGACATTTTGTAGGAATAATCTTAAAGCA

GATGATAAGGTTAAATTTTTTTTGTTTAATACTGTCATTGCTTCGCTTACCAATTCTTTA

TTAGCTTGAAAGGTAACCGCTCTGTTTTTTTTTACTGTAGTCATTTTATGTAACCCCTTT

ATGTAATTTATTTAATTATATCATATATACAAAAAAGTCAATAAAAAAGACTCGGTAATT

GAGACAGATTTTCATTATTTGATAAGCTCCGAAACTTATTTTTGATACCGTTTAGGTTTA

AGTATTTGTCTATAAATAAGATTTTTAGTGATTTTTCATTTTAGAAACATTGATTTTAAA

GGGATTTGACTTTAAATAATGGAGTGAACCAAAAACCAGGTCTTAAAAAAGCTCGTAAAG

CTTCAGAATTCTCAAAACGTTAATATACTGTTACATCAACGTTTTAAAGCGCTTTGAAGA

CACCTCTTAAGTAGACATGGTAACGAACCAAAATCTACTTAAGAGGTATGTTGATGGCTA

AAAGAAAGCTTTACTCCATACGATAAAGAACAAGCATGTCTTGATGACAGTAATGGCAAC

CATTCTAGAGGTGAAATATATAGCTGTCTGCCTATTTCCACGTTATTCATCAGAAAAAGG

AACAGTACAAGACTGATAAGTCTGAAAAAAACCAGAAAATAAGTTAGCAAGAAACTTCTT

TATAATACAAACACTACTTTCAGATATGCTCCCTGTTTACTTGACAGGGGATATATCTAA

ACCCTGAAGTGCTTTTTAATATGAGTAGGTCCTTTACTTGTCAGCGATTATGATTTTTGA

GAATTTCTTGTACTTTTCTATGATACCAGTTGTCACATTTTTATCAGTCACAATTAAGTC

CACTTGATCTAGTTGGTAGAAATTATAGAAGTCATAACTTTCAAACTTGGTACTATCAAC

TAGAAGGATTTTCTCAACAGCATTATTCAATGCAACCCTTTGAACTTCTCCTTCGAGATC

ACTGTACGTCGCAATAGAATTATTGTAAACTGCATTTGCGCTAACAAAAGCCTTTGAAAA

AGTTAATGATTCTAGATTATTAATGGCAATAGCACCAACAAAAGCTCCAGTAATTTCACG

ACATTCACCACCAATTAAAATCAAATCAATAGTAGTGCTCGGGTGCAAAATTAAAAAAAC

AGGAAGGCTATTTGTAATGACCCTGATATTTCTATTTTTCAATTCGACAGCTAGATGCTC

TAATGTTGTCCCTGGACCTATAAAGACAGTTTCGCCATCTTTAATAATGTTTTTAGCTCT

TTGTGCAATTTGTTTTTTTTCGGTAGTCTGTAAGACCTTCTTTTCAGTGTGTGTTTTCTC

GTGACCTTCGTCCTTTGCTGAAGAATAATCCAAATATTGAGCACCACCATGGGTCCTGAT

AAGTAATCCTTTATCTGCCAATGTATCGAGATCTCGTCTAGCGGTCATATCTGAGATGTT

CATTTCATCGATAATATCTTTTACTTTTATAAAACCGTCAACTTTTAAGATATCGAGAAT

TTTTTCATGACGCTCTTTCTTTTTCATTCTTATCTCCTATCAGTGTCTTAGGGTATAACT

ATTAAAGGTATCATAACATAATATCAATAAAAAAATCAAACAAAACAAACATAAATGTTG

GAAAAAACAAAAATTAACAAAATAGAAAACGTTTTAAATAAAACGTTTACAATAAATAGA

GATAAGGGTTTCGTAATATAGGATAGGTTACGAGTGGCTGTCAAAGGGATTTATGAAATG

AAAAAGAAACACAAGAAATATTAGGATAATAACGTAAAAATGAAAAAAATAAACATATTT

TGTTTATTTTTTATAAGAATAAACAAAAAAATGTTGACTTTTATGTTTGGTGGGTGTATG

ATTAGGTCATAGAAATAATGAAAGCGTTATTTCAAAAGATTTTGCAAATCGTTAATTAAC

TATCTACGTAAGTTTTCCACATTTACTTATTCTAAATTATAGGAGAAATAAAATGGCAAT

TATTATCGGTGCTGACAAAGCAGGTCAAGAACTTAAAGAAGTTATCAAAGATTACCTTAA

AGAAGGTAAATACGAAGTCGTTGATGTTAGTGAAAACGAAGTACGTGACTTTGTAGATAC

AACTTTAGCCGTTGCAAAAGAAGTTAATGCATCTGAAGATAACTTAGGTATTGTCATTGA

TGCATATGGTGTTGGTTCTTTCATGGTAGCTACTAAAATCAAAGGAATGGTGGCAGCTGA

AGTATCAGATGAGCGCTCAGCATACATGACAAGAGGTCACAATAACTCTCGAATCATCAC

GCTTGGTTCTGAAATTAGTGCTCCAGGAATTGCAAAGAACATTATCAAAGGCTTTGTCGA

AGGTAAATATGATGGTGGACGTCATCAAGTTCGTGTCGACATGCTTAATAAAATGTGCTA

ATTGATTGAAATAGGTTAAAACAAGGAGAACAAAAATGAAAATTGCTGTAGGTTGTGACC

ATATTGTTACTTATGAAAAAATCGCTGTTGTTGACTATTTGAAAACACAAGGTCATGAAA

TTATTGATTGCGGGACATACGATAATGTCCGCACACACTATCCAATCTTTGGTAAAAAAG

TTGGTGAAGCAGTAGCAAGTGGTGAAGCAGAATTAGGTGTTGTTATCTGTGGTACTGGTG

TTGGTATCACAAATGCTGTAAACAAAGTTCCCGGTATTCGTGCAGCACTCGTTAGAGATA

TGACATCTGCTATCTACTCAAAAGAAGAGCTAAACGCAAACGTTATTGGTTTTGGTGGAA

AAATTATCGGTGGTTTATTGATGAATGATATTATCGATGCTTTCTTAGCCGCGGAATACA

AACCAACTGAAGAAAATAAAAAATGGATTGAAAAAATGGATAGCTTGCAACATGCTAGTC

AAGATCAAAACAACCCACACTTCTTTGATGAATTTCTAGAAAAATGGGTCCGTGGTGAAT

ATCACGACTAATATTTGAATAAATTACTATAGGGATATATAGAGAAAAAGGAGCTATTGT

GATTTTAACAGTTACTTTAAATCCAGCTATTGACGTCTCCTACCCTTTGGATGAGCTAAA

ATGCGATACTGTAAACCGAGTTGTAGACGTTACAAAGACACCCGGTGATAAAGGCTTGAA

TGTTTGCAGAGTTCTAAATGATTTTGGTGAGACAGTCAAAGCTACTGGTTGTATTGGCGG

AGAAAGCGGTGATTTTATTATTAATCATCTTCCTGATTCGATTTTAAGTCGCTTCTATAA

AATTTCAGGTGATACTAGAACTTGTATAGCCATTTTACACGAAGGTAATCAAACGGAGAT

TTTAGAAAAAGGACCGCTGTTAAGTGTAGAGGAAATTGATGGATTTACTCATCATTTTAA

ATATTTACTGAATGATGTTGACGTAGTGACGCTCTCAGGCAGTTTGCCTGCTGGTATGCC

AGATGATTACTATCAGAAGTTAATCGGTATTGCCAATATTAATGGTAAGAAAACGGTTCT

GGATTGTTCTGGAAATGCTCTAGAAGCAGTACTAAAAGGTGATAGTAAACCAACAGTTAT

CAAACCTAATCTTGAAGAACTTTCTCAACTTCTAGGTAAAGAAATGACAAAAGATTTTGA

AGCTTTAAAAGAAGTCTTACAAGATGAACTATTTGAAGGAATTGAATGGATAATCGTTTC

TCTTGGTGCTGATGGTGTATTTGCAAAGCACAATGATACGTTTTATAACGTTGACATCCC

TAAAATCGAAATTGTAAGCGCTGTAGGTTCTGGTGATTCTACAGTAGCAGGTATTGCTTC

AGGTTTAGCGAATGATGAAGATGATCGAGCTTTATTAACAAAAGCAAATGTCTTAGGGAT

GTTAAATGCGCAAGAAAAAACAACTGGGCATGTTAACATGGCAAATTACGATAAGCTATA

TCAATCTATTAAAGTCAAAGAGGTATAAAAAATGACAATTACTTTAACTGAAAACAAACG

TAAAAGCATGGAAAAACTAAGTATAGATGGTGTTATTTCTGCTTTAGCTTTTGACCAACG

TGGAGCTCTCAAACGTATGATGGCTCAACATCAAACAAAAGAGCCAACAGTAGAACAAAT

TGAAGAGCTTAAAAGTTTAGTTTCTGAAGAACTGACACCATTTGCATCATCAATTTTACT

TGATCCTGAATATGGCTTGCCAGCAAGTCGTGTTCGTTCAGAAGAAGCAGGCTTGTTGTT

GGCATATGAGAAAACAGGATACGATGCAACAACAACTAGTCGCTTACCTGATTGCTTAGA

TGTTTGGTCTGCAAAACGTATTAAAGAAGCTGGAGCAGAAGCAGTTAAATTCCTTCTTTA

CTATGATATTGATGGAGATCAAGATGTTAACGAGCAGAAAAAAGCTTACATTGAACGTAT

TGGTTCTGAATGCCGTGCAGAAGATATTCCATTCTACTTAGAAATTCTAACTTATGATGA

GAAAATTGCTGACAATGCAAGCCCTGAGTTTGCAAAAGTTAAAGCTCATAAAGTTAACGA

AGCAATGAAAGTTTTCTCAAAAGAGCGCTTTGGTGTTGACGTGTTGAAAGTTGAAGTTCC

AGTAAATATGAAATTTGTAGAAGGCTTTGCAGATGGTGAGGTTCTCTTTACAAAAGAAGA

AGCAGCTCAAGCTTTCCGTGATCAAGAAGCTTCTACTGATTTACCATATATTTACTTGAG

TGCAGGTGTTTCAGCCAAGTTGTTCCAAGATACTTTGGAATTTGCAGCAGAATCAGGTGC

TAAATTCAACGGTGTCTTATGTGGACGTGCTACCTGGGCAGGATCTGTAAAAGTCTATAT

CGAAGAAGGACCACAAGCAGCTCGTGAATGGTTGCGTACAGAAGGTTTCAAAAATATTGA

CGAATTAAACAAAGTACTAGATAAAACTGCAAGTCCTTGGACTGAAAAAATGTAACATTA

TTTTCGGAGGAAACTACAATGAATAGAGAAGAAACTACTCTTTTAGGTTTTGAAATCGTA

GCCTATGCTGGTGATGCCCGTTCAAAATTTTTGGAAGCGCTTAAAGCAGCGCAAGAAGGA

GATTATGCTAAAGCAGAAGAATTAATCGCTGCAGGTAGTGACTGCTTGAATGATGCTCAC

AATGCTCAGACAAGTCTACTACAAAAAGAAGCAGCTGGAGATGACTTAGCTTATAGTGTC

ACTTTAATGCATGGTCAAGATCATTTAATGACGACCATTTTATTACAAGATTTAATGAAA

CACATGATTGAACTTTATAAGAGAGGAGCAAAATAATGAATGGGTTAATTGCTCAGATTG

AGAAAGGGAAACCTTTCTTCGAGAAAATTTCTCGAAACATCTATCTTCGCGCTATTCGTG

ATGGTTTTATCACTGGTATGCCAGTTATCTTGTTTTCAAGTATCTTTATCTTGATTGCCT

ATGTTCCAAATGCATGGGGCTTCCATTGGAGTAAGGATATTGAGAATCTCTTGATGACGC

CATACAACTACTCAATGGGTATTTTAGGTTTGTTTGTGGCTGGTACGACAGCCAAAGCTT

TAACTGACTCAATGAATCGATCATTACCAAGCACAAACCAAATCAACTTCATGTCTACCA

TGCTCGCTGCTATTGTAGGTTTTTTACTTATGGCAGCCAATCCAGCAAAAGACGGTGGTT

TCTTAACTGGATTTATGGGAACAAAAGGTTTGTTGACAGCCTTCATCGCAGCTTTTATTA

CCGTAAATGTCTATAAAACCTGTGTGAAAAACAATGTTACTATTCGCATGCCAGAAGAGG

TTCCACCAAATATATCTCAAGTTTTTAAAGATTTGATTCCATTTACGTTATCAGTTGTAC

TGCTTTATGTGATTGAATTGATTGTTCATACTACTTTAGGCGTTAACGTTGCCGAGTCAA

TTGGTAAACTTCTTGCACCATTGTTCCAAGCAGCAGATGGTTATGTTGGAATCACTATTA

TCTTCGGTGCATTTGCTTTCTTCTGGTTTGTTGGTATTCATGGACCATCTATTGTTGAAC

CAGCCATTGCAGCGATTACTTACGCAAATGCTGAAACCAACCTTCAATTACTTCAAGCTG

GTGAACATGCTGACAAGATTTTGACTTCAGGGACTCAAATGTTCATCGTTACCATGGGTG

GTACTGGTGCAACTCTCGTCGTTCCATTCATGTTCATGTGGTTAACAAAATCAAAACGTA

ACAAAGCCATTGGACGTGCATCTGTTGTTCCAACATTCTTTGGTGTTAACGAACCTATCT

TGTTCGGTGCCCCACTTGTTTTGAACCCAATTTTCTTTATTCCATTTATCTTGGCACCGA

TTGTCAACGTATGGATTTTCAAATTCTTCATTGACACACTTGGAATGAACAGTTTCACTG

CAAACTTACCTTGGACAACACCAGCTCCATTGGGTCTTATCCTTGGGACAAACTTCCAAG

TGCTAGCATTCATTTTGGCAGCTTTGTTAATTGTCGTTGATGTGATCATCTACTACCCAT

TCTTAAAAGTATATGATGAGCAAATTCTTACTGAAGAAGCAGCAGGTACGAATTCATCAG

ATGCCCTCAAAGAAAAAGTAGCAGCAAACTTTGATACTAAAAAAGCTGATGCTATTCTTG

AAAAATCAGCAGCTAAAGAAACCAAAGAAATCACTGATCAAACTAATGTTCTCGTTCTTT

GTGCCGGTGGTGGTACAAGTGGACTACTAGCAAATGCTTTGAATAAAGCAGCTAAAGAAT

ATGGTGCACCTGTCACTGCAGCAGCAGGAAGTTATGGTGCTCACCGTGAGATTTTACCAC

AATATCAACTTGTTATCCTTGCACCTCAAGTAGCTTCTAACTATGAAGATATGAAAGTGG

AAACTGATAAACTTGATATTAAATTAGCTAAAACAGAAGGGGCTCAATACATCAAATTGA

CCCGTGATGGTCAAGGAGCTCTTGATTTTGTAAAAGCACAGTTTGAAAATTAAGAAAACA

GTTTAAATGTCGTAAAGTTCTCAAACACAAAAATTGTTTTGGGAACTTTATTTTTGAAAG

ATATTCAAGGAGAAACTGAGATGACAAAAACATTACCTAAAGATTTTATTTTTGGTGGTG

CTACAGCTGCTTACCAGGCTGAAGGTGCTACCCATACAGATGGCAAAGGACCAGTAGCTT

GGGATAAATACTTAGAAGACAACTATTGGTACACTGCTGAACCAGCAAGTGATTTTTACA

ATCGTTATCCAGTCGATTTGAAACTTAGTGAAGAATTTGGTGTCAACGGCATCCGTATCT

CTATTGCTTGGTCTCGTATTTTTCCAACAGGAAAAGGAGACGTTAACCCTAAAGGAGTTG

AATACTACCACAATCTCTTTGCAGAGTGTCATAAGCGTCATGTTGAGCCTTTTGTTACAC

TTCACCATTTTGATACACCAGAAGCTCTCCACTCGGATGGTGATTTCCTCAATCGTGAGA

ACATTGAACATTTTGTAAATTATGCAGAATTCTGTTTTAAAGAATTCTCAGAAGTTAACT

ATTGGACAACATTTAACGAAATTGGACCAATTGGTGATGGCCAATACTTAGTTGGTAAAT

TCCCTCCAGGTATTCAATATGATCTTGCTAAAGTTTTCCAATCACACCATAACATGATGG

TTTCTCATGCTCGCGCTGTGAAACTCTTTAAAGATGGTGGCTATTCAGGTGAAATTGGTG

TTGTCCATGCTCTTCCAACTAAGTATCCATTTGACGCTAACAATCCTGATGATGTTAGAG

CAGCTGAACTGGAAGATATCATCCATAATAAATTTATTCTTGATGCAACTTATCTAGGTA

AGTATTCAGATAAAACAATGGAAGGTGTTAACCATATCCTTGAGGTGAATGGTGGTGAAC

TTGATCTTCGTGAAGAAGACTTTGTAGCACTGGATGCTGCAAAAGATTTGAATGACTTCC

TTGGTATTAACTACTATATGAGTGATTGGATGCAAGCTTTTGATGGTGAGACTGAAATCA

TTCACAATGGTAAGGGTGAAAAAGGCAGCTCTAAATACCAAATCAAGGGTGTTGGTCGAA

GAAAAGCACCCGTTGATGTTCCAAAAACGGACTGGGACTGGATTATCTTCCCACAAGGCT

TATATGATCAAATCATGCGTGTCAAAGCCGATTATCCTAATTACAAGAAAATTTACATTA

CAGAGAATGGTCTTGGCTACAAAGATGAGTTTGTAGATAATACTGTCTATGATGATGGAC

GTATCGATTATGTGAAAAAACACTTAGAAGTTATTTCTGATGCTATTTCTGATGGTGCAA

ATGTTAAAGGATACTTTATGTGGTCACTGATGGATGTCTTTTCATGGTCAAATGGCTATG

AAAAACTTTACGGTCTCTTCTATGTTGATTTTGAAACTCAAGAACGTTATCCTAAGAAGA

GTGCCTACTGGTATAAAAAAGTAGCAGAAACTCAAGTGATTGAATGATTTTGAGATACTG

CTTTAATACATTTTTACACGTCATGTTCGTTTACTGAATTATAGTCACAAAAAGTAGATG

ATGTTGACATTTTTCAAGCATAGTATTATTTGCTGTTGATGGTAATCCATTAGTTGTAAG

ATCGGGTTATAAGTGTTTTTAGTTGATGAAAATGGGTTTTGAAAGTTTTATTTAACATCC

TTTTTCAGATATTTGACTTCTGTCATTCAAACTCCTGACCTGATTAAATAAGTAAAGTAA

CCATTTTCTGATTAGAGATTGATGTTATAACGAGAAGCGATTAGATTGTAAAATAGCTGT

AAAGAACGAGTTTTGTTTTTTATATCAGAGGGCTATTTGTTATAAAGTGACCTCTTATGT

GACATCTCAAAAATCTTACTAAAAAATATGAAAAAAGTCATTTTTGCACTTTTTACTCAT

TGAAAATGAAGACGTATTATTTACAATAAAAGTGTAGAAAATAGTCACTACATACTGATA

GGAAGGAGAATGAGTTTTATGAAAAATTCAAAGGATATTTTGACTAATGCTATCGAAGAA

GTTTCTGAAAAAGAACTTATGGAAGTAGCTGGTGGTAAAAAAGGTTCAGGTTGGTTTGCA

ACTATTACTGATGACTGTCCGAACTCAGTATTTGTTTGTTGTTAATTAAAACAATCTGGA

TAAGCAGACTAAAATTTCCTGTTTAGACTTAGTCTAGGCAGGATTTTTAAGTAGTGGTAG

GAAAAAATGATAAAAAGAAATGAATTAAAGTTAGAGTATCAGCAACATCAGTTTTACGAA

TACTTTAACTCAATTTTTGATTACGACATATTAGATACTTCTATTAGTGAAAATATTTAT

TTGTTAAGGGAAAAGACACATAAGTTATTTTATAGTGAGTTTGAAAATCAGTTATTTGAA

ACTATAATGTTTCTTTCAATGAAAACACTAGTTCTAGATATAAATCATTTTTCCAAGGAA

ATTGAAAACAAGTCAAAGGCATATGAACAATATATACAGCAGATAAGGGAAGAAAATGGG

ATAAGTAATTTTTTTGACAGATATCCATATTTGCTAAAACAAATCAATAGAGAAGTTGGA

TTGGTAGTGGAATCATACTCTTTGCTATTTGATAGGTTTTTAAAAGATTTGTCTGAAATA

AGGTCATGCTTTAATATTACTGAGTCCTTAAGTAATGTTGAATTTTCACTTGGAGACAGT

CATTCCCAAAAACAAACAGTCGTTAAGATTGAATTTAAAGGGAAATCCGTTTACTACAAG

CCTAAAAGCTATGATTCATACAACATCTTGTTGGAACTTATTAGTCTATTGAAATCTAAT

AATATACCTTCGTTCAGTCTTCCTGAAAGCTTAATTAAAGCGGATTATTGTTGGCAACTT

GGTGTAGCTTATACAAGTTCCAATAAAGATGAAGTAGCGAAAATTTACTTTAAGTATGGT

GTTTTAGCAGCATTTTCTGAAATATTTTCTATTACGGACTTACATATGGAAAATGTGATT

GTTTCAGGCGGAGATTTATATTTAATTGACGTTGAAACCTTTTTTCAAAGAAAGTTGAAT

GTACAAAATCAGAATTTTGAAGGGATTACTGTAGACACCTATCAGAGAATTTATAAGACA

TCACTTTCAAATGGTTTATTTCCTGTACAATTTGAGAAAAACAGCGCTCCTAACGTTTCT

GGGATAAGTGGAAAAGGTGGGAAAAGAAAAAAGGGAAAGTATGAACTGATAAATAAAAAT

CGTGGAGATATGAAGTTAGTGAAAACTGACTATTTTCAGGAAGATGGTTATAATATTCTG

ACATTAAATGGGAAAGTGGTGGAACCTCTCGACTATGCTAATGAGGTAATTGCAGGTTTT

AGAGAATGCTATACATTTCTTATGTCGCAACGTGCAAAGGTTAAAAAAATTTTAGAAGAT

TTCCCAAAATTGAAAACTAGAGCCATATTCAGAAATACTTCCGACTATGGAAAATTTTTA

CAAGCCTCTACGAATCCAAAATATCTTTTCTCAGAAAAAAAGAGGGAGAATTTATTTTCG

ATACTACATGAGTCAAAACATATTGAACAATTTATTGTTGTTAGTGAAATTAAAGATTTG

ATGAATGGTGATATTCCTTACTTTTCAATGGATACCAGTGGAAATGTATATAATTCTTTA

GGAACAGTAATTGGAAATCTAGGAGAGACAACCTCATTATTTGATAATATAGTTACCCTG

AATGATGAAAGAATGAAGTTCACTTGCGAATTGCTTGGAATAGTCTTGAAAAAACCGATT

AAGCATTGGGAGAGGGAAAAGGGCAAATCTTATCAGTTTTTATCAATCTCCTCAGAACAC

AATTTTAGTGAGGAAATCTTAGACAGCATTAGACGAATATTTATTGATGCAGATAAGAAT

AGCTTTTCTTCAGAGGAGGAGATAACATGGCTCAATATTGATATTACAGAGACTGAGCAA

TGGGTAATATCTCCTCAGAACATTACCTTGTATAATGGATTGATTGGGAATGCCTTAGGT

TATTTATATGCTTATCAAATTCTTGGTGAAGAGCAATATCTAGTATCATTAAATAAGATA

TTGAAAACATTGGAAACCACAAAGAATCTTATAGAAACTAGTGATATGTCAGTTTTTTTG

GGAAAAGGAGGTTTGATTTATCTATACTTTAGTTTGTGGAAAAGACTTAAATTACCTCAA

TACCAAAAATTATATTTAGACATAATCAAAGAATTTTCCAGCCAGTCACTAGAGGAGCAA

AATATAGATTATATTTCTGGAGTGTCTGGTTTATTAGTTGTTCTGTGCAACATATATAAT

GTAGAGCAAAATAAAACAGTATATCATTTAATTAATAGGATATCTGAATTTATTATTGAT

AATGTGAAAAAAGAAGATGATAAGGTTTATTGGGTATCGGATTTTTCAGATTCAGAAATT

CTAAATGGACTATCTCACGGACAATCTGGGATTGCATATGCATTACTTTTATCATGGAAA

ATCAATAAAAATTATAATTATTTTAAAATAGCGAAGTCAGCTATTGATTTTGAAAATACC

CGTATTTCTGATGGAAATTGGATTGATTTTAGAAATAAAGGAAAACGTTCAGAGTTAGGA

ATGCCAGAACCTATTTATTGGTGTCATGGTGCAACAGGTATCGGATTAACGAGATATAGA

GAATCAAAATGGTTAAACGATAAAGAGTTGAAAAATAATTATGAAATGGCTAAACAAACG

GTATTAAATAATGGTTATTTGAATTCGGATTGCCTTTGTCACGGGAAAATGGGCAACATG

GAATTATTTATGAATTTAGATGACTCCTTGAAAAATGAAGTAGATATAGAAGGTATCATA

CTTAATATTGTTCGTAATTCTCAAAAATTTGGTTGGGAATCTGGCTTGCCACAACACACA

AGAGTGTTTAATATGATGGTTGGTGAAATAGGGATAGCATACCAGTTACTTAGATATATA

TCCAATTATGAAGTACCATCATTATTGTTATTAGATGTTCCAAAAGGGAGTATTGAAAAT

GAAAAAGATTACACCGATTGAACAAACAACTCCTACTGAATGTGGATTATGTTGTTTATA

TATGATGTTAGATTATTTTGATATTTCAGAAACATATTTTAAATTGAAACAACAAGTCAA

TTTAGGGAGAAATGGACTGAGTATAAAGAATATCTCAGATATTGCTTCTATCTACGGAGT

TACTTGTAAGACTTATCGTTTTTCCAAATATCCCGAAAACTTACCTGTCATGGTATTTGT

TTCAGATTCGCATTTTGTAATTTTAGAGGATATTCGTGATGATGCCTTTACTATTGTTGA

CCCTGCTGTTGGGAAATATGTCTTAGCAAAGAATGAATTTTTTGAATTATCTCCTAAATT

TTATACCGAATTTTTTTATGATAAGGCGACTAATTCTTCAAAAAAAATAGCCAAACGAGG

ATTAGTTGGCAGAAACGTAAAGGAAATGATATTTGTAAATAGGAAGGATACTTTTTTAAC

CATTCTATATACTCTGATTTTTCAGATTATCACAGTGTCAATTCCATTTTTTATAAGGGG

AATTATTGATGGAAATTGGGTTTTCCTTAAAAAGTTTGGCTATTTAGAAAGTGCAGTCCT

ACTTTCTATAATAATTTTTTTTCAGGGAGGATTTTATTTTCTAAAGAATATCTCTTTAGT

GAAATTGCAAAATAAATTTCACAGCACTATTTCAGAAAAGTTTGTGACTAAGTTATTGAA

ACTTCCCATAGAATATATTGGAAAAATAGATAAAACAGACATCATTCATAGATATAATGG

TTTGATGATTGTGAGGGAGTTACTATCAGAAAGAATTATTTCTATCTGGTTGGATATTAT

CTTAATGTTTGCGAGTATTAGTTATATCACATATGTTTCTACACCTTTGGGAGTTATATT

GGGTATAGTTTTTATTGTAGAAATGATGATTTTTTTCCTATCACTAAGCATTAAACAAGA

AAAGTTAGGTAAAGAAGTTTTAAGACAAAAGAACTCTTTACAAACATTTTTTTCACTGAT

GGACGGGCTATTTCTTTTTAAAGCTAAAAATTCTGAAAAAGAACTTTTGTCAAAATGGGA

TAAAAGTTTTAAGGACTATATCAATTCAACATATGATAGAAATAGATATTTCAATCTTTT

AGGAGCAGTAAATTATGTGATAACTTTTTCCATACCTATTTTGTTAATTCTGTTATTTCT

ATATTATTCACCAAATAGTAGCAGTGGTGAGCTTATTCTTCTTTATATGATGGTTTTAAA

TTTTATTAATCCCATTAATAATATTTTGAATTCGATTGATGAGATTCTATATGGTGTTAA

GCATTACGAAAGGGTATTAGAGATATCAACTCTTGATGATGAGGCGAATGGCACTTATAA

GCTATCAGAGGATACAGATATAGAGATAAATTTAGAAAATATAAACTATCACTATGAGTT

AAATGGGGCAGAGGTGTTAAATGGAATAAACCTAAAAGTCCATTCAGGAGAATTTGTTGC

AATTATAGGGAAAAGTGGAAGTGGAAAGACAACTCTAGCTAAGATGTTGTTAGGCTATGT

AAGCCCTAGTAGTGGAGACATCACATACAACAATATATCCTACTCTAAAATTGATAAAAC

TGATTTTAGGAATATATCGGCTTTTGTATCTCAAGATTCTCCCATATTTGATGGGGATGT

GATGTATAACATTTCGCTAGGGAGAGAATCTGTTTCAGGAGAACAGGTTATTGAAACTTG

TAAAAGGGTATCACTATATGAGGATATCAGGAGTATGCCAATGAAGTTTCATACTCCACT

TTTTCGAGATAATCCATCACTATCTGGGGGGCAAAAACAACGAATTTCTTTAGCAAGAGA

GTTAGTAACTACCCCTAGAATCTTAGTTCTTGATGAACCTACATCAGCTTTAGATGTAAA

AACTGAAAGAATAATCCAAAAAAATGTTGAGGCTTTACATTGTACGAGGGTTTTGGTTAC

CCATAGACTTAATACAGTTGAAAAAGCTGATAAGATTTTAATAATGGATAATGGCAAAAT

TATTGACTATGGAAACCATCATTATTTGTACAAAAATAATAAGGATTATTGTGACTTATA

TGACTCGTATATGAATAAATATCAGGAGGAAGAGGCAAAATGAAACAATTAGTATTAAAA

GATGTTTGCAAAAAATATCCCAATCAACTGAATTATGCTCTAGACCATATCAATTTGACT

GTTAGAAAAGGAGAATTTGTAGCTGTCATGGGGCGTAGCGGTAGTGGGAAAACGACGCTT

TTAAATGTTACTTCAATTATTGATAAAATTGATAGTGGGAATATTTATTGTGCGGATAAA

GAGATTAGTGTATTTTCTGATAACGAGGCAACTAGTTTTAGAAAAAATGATATTGGTTTT

GTTTTTCAAGATTATATGCTGTTAGACAGTCTAACCATTAGGGAGAATATTTCTGTTGCT

TTGTCTCTAAAAAATATTGATTCTTCAAAGATTGATGATTTGATAAATAGCTATGCAAAA

AGATTTAATTTATATGAACAGTTGAAGAAATATCCGTATCAACTTTCTGGAGGTCAAAGA

CAGAGGGTCAGTATTATCAGAGCAATCATTAAAGAACCAGAGATTATTTTTGCTGACGAA

CCAACTGGAGCACTTGACTTGAAATCTTCTGAGGAAACAATGATGATTTTGAGTGAAATC

AATAAAACAGAAAAGGTAACTATTTTAATGGTTACTCATGACGTTTTATCAGCTTCTTAT

GCCGACAGAGTTGTACTTCTCAAAGATGGGAAACTTCACATGGAGATTGATAAAAAAGAT

TGTGGAGAGTCGTTTTACGATGTAATTAGTCAAGCTTTATCCGATAGAGGAGAGTAATAT

GATTTGGTCCATTACAAAATCTAACATTAAAAAAAATTTTTCGTTATATCGTATCTATTT

TCTAGCTACGATTGGTTTATTAAGTATTTTTATAGCTTTTCTAAATTTTATCTCAGATAA

AATCATTACAGAAAAAATTGGGGATAGTGGTCAAGCTCTAGTTATCGCTAATGGGTCATT

AATTTTTTTGATTGTATTTTTGGTGGTATTCTTAATTTACTTCAATAATTTCTTTGTAAA

AAAACGTAGTCAAGAGCTTGGAGTCTTAGCAATACTAGGGTTTTCAAAAAGAGAATTAAC

AAAATTACTAACTTTAGAAAATCTTGTTATTCTAGTTCTGAGTTACTTGGTAAGTTTATT

GCTGGGACCGACTTTATATTTTTTAGCTGTACTGGCAATTACTCATCTATTGGAATTAAC

ATTGGAAGTTCAGTGGTTTATTACAGCTAATGAGATTATAGAGTCTTTAGGAATATTAGT

TGTAGTTTTTCTGATTAATGTCATCACAAATGGACTTATCATTAGTAAACAGTCTTTGAT

TGAATTTGTTAATTTCTCAAGAAAGGCTGAGAAAAAAATTAAGATAAGAAAAGTCAGAGC

TATTACTGCTATTACTGCATTGCTATTATCATATATTTTATGTTTGGCGACAGTATTTTC

ACCCACACGAAATATGCTATTAGGCATAGGGATGGTACCGGTTTCTCTATTGATAATTGT

TTTAGTTGTTTTGGGAACAGTGTTTACCATCAGATATGGATTGGCTTTTGTAGTTTCGTT

ATTAAAAGGAAATAAAAAAAGGTTATACCGTCCTCTGTCTAATATCATCTATCCCAAATT

TAACTATCGTATTGCAACAAAAAATAAATTATTAACAGTCTTGGGAGGTCTTTTAACAGT

AACCGTTTCAGTTGCCGGAATGATGGTAATGCTCTATGCTTATTCTCTTAATGGGATAGA

GAGGTTGACTCCATCTGCCATAGAATATAATGTTGAATCAGAAAACGGTCAAGTCAATGT

TACAACTATTTTAGAGAACGACCAAGTGAGCTTGGTTGATGTTGACCTATTGAGATTGAA

CACTAACCCAGAAGTAACTATCACAGAATCTGGGCAAACAATTCCTTATTTTGATATAAT

TAATTACAGTGATTATAAAGAGTTAATGAAAGCTCAAGGGAGAATAAATTCTATTGAAGG

TAGTAAGTCACTCCCATTGTTAATAAATTATTATCCAACAGAAATTAGCCTTGGAAAAAC

CTTTAACTTAGGAAATGCATATGATGTTACTGTAAAACAAGTATCAACGCATAATGTTTT

TAGTTTTTCTACAAGTGTCACGACCTTGGTTGTTTCTGATAAATTATATGCTAAACTTAG

TTCTTGTTTCCCAGAGAAAGAAATGACAATTAGGACTTTTAATGGAACTTCGATTAGGTC

AAGTGAAGCATTTTACAATCAGTTTAGTATGGTTCCTGATGTTATCAGTAGTTATAGTAA

GGAATACACAGTAAAGACTGCTAATATTGCGACTTATATCTTTATAACTTTCCTATCCAT

ACTCTTTATTATTTGTACAGGTAGTATTCTGTACTTTACAAGCCTCATCGAAATCATGGA

AAATAAAGAAGAATATGGCTATCTAAGTAAGCTAGGTTATAGTAAAAAAATGATTCATCG

GATTCTTCGATATGAAACAGGTATACTTTTCCTTATTCCTGTATTCATTGGGATTGTAAA

TGGTGGTATGTTGCTTATTTACTATAAATATTTATTCATGGATACATTGGTAGCAGGCAA

TATCATAATGTTATCTTTATTGCTTTGTCTGCTTTTCTTCTTGATAATATATGGCACATT

TTATGTATTGACATTGCGGTCAGTGACATCCATAATCAAAAATTAATTTTAAAAATCTTT

TAACTTTAACGATTAAGTAAATAATATGATAAGAATAAAAAACATAACAAAGAGTAAGTT

CTTTGGGACTGCAATAATCTTGTTGCAACAATTAATAGCCTTGTTAATTTTGGTCTATAA

TCGAGAGAACCTGTCTCTTCTTTTCTCTGAAAAAGTAGCTCTTGTAATGACCCTTATTGA

TACAGCTTTTATTTGGCTGGCAACAATATTACGCCAAAAACAAGGTGATATTTTTAAAAG

AATTATTTCTATCATCTCCTTAACAATTTGGCAATACTTAGTATCTGTGCTGACAAGAGG

AACTCCTCTCTTTTTAAGCAGTGGACTACAGATTATATTGCTCTATTGTTATACCGTTGA

GATTACTAATTTAATATTATATGGGCACAAACAATTTAAAGATAAGTTAGATAAGGGCTT

GTTAATAATTATTTTTGTTTCTATAACTAGCCTTTTTGTTAATAGAATTTTATTTAATTT

TTTATTCCTGATGGTTTTTACTATTTTACACCTGTATCCGTTACTAGTTATAGTGCTATA

TTATAGGTCCTTTAGGCAACAAATATCAGTTGTTAGACGTTCATTGATACTATTTTCTTT

ACTTTTGCTGGTAATACTTGGTAGTGAACTTTATGGAGAAATGCTAGATGTTAATCAAGC

CTTTAATAATTTGGGATGGTATCTGTTTCCCCTTATAATGAGTGTAATATACTATTTTAA

GACCATACATGATAAATTGAGTTTTGTTATCCAAAGATGGTTAGGTGATTACAAAGCTCG

TATAGAACTATTATTTCTACTTCTTATCCTTTGTTGGGTTGTACTCATAAAGGTACTAGT

TAAAGAATTTTTATTATTTTTTATAATTGTTGACGCAAGCACGTTATTTTGTCTAGTGGT

AATTTCTTGCATCTTTTATTATTTAGAAAACTCCAAACAAAATTTTGACTATGAAAATAG

AAGACTGAACTATTTTATGAAATCAGAGGAAAACATGAGAGTGGAGTTCTCAAATTATTT

GCATGATGATGTTTTACAAAATATCATAGCGATAAAAAACTTGCTTTCTCTAGAAAATAG

TAATATAACACATGGTTTTATTGTTAATGAGTTGAATGATTTAGTTTCTGGTATCAGAGA

AGAAATTGATACTTACCACCCAATAGTTCCCGCTAATCAAACAATGAAAGAAAACATACA

GTCTCTTTTTGATGATATTGTTAAAAGTAGAAAAAGTAATACCTTATTGTATTTTAACTG

TTCAGATAATATGGTAGTGCCATCCCCATATGGAGATATTGTTTATAGGTTTATAAAAGA

GCTAATAAACAATGCTATAAAATATGGAGATGGAAAAGACATTCGTTTATCTTTAACGAT

TCAATCTGATATTATTATCATTGAGGAGAGCAATCAAGTAGTGGAAAAAGTACACTCTAT

AAGTTATGGCAGAGGATTGAAATCATTTCAAGAAACACTAGCAGCTTTTGATGGAGATCT

AGAACTACAAATGGATACTAAACAATTTACTATCAGAATATTACTTCCCATAGATTGGAA

ATTGTGTTATGAAGATTTTATTAATTGATGACCACAGACTATTTGCAAAGAGTATCCAAC

TGTTATTCCAACAGTATGATGAAGTAGATGTCATAGATACGATTACTTCTCATTTTAACG

ATGTGACGATTGATTTGTCTAAGTACGATATTATATTGCTTGATATTAATTTAACAAATA

TTTCTAAAGAAAATGGATTGGAGATAGCAAAAGAACTCATACAGTCTACTCCTCATTTGA

AAGTTGTTATGTTGACAGGATATGTTAAATCAATCTATAGAGAGAGGGCAAAAAAAGTAG

GAGCATACGGTTTTGTAGATAAAAATATAGACCCTAAACAACTCATTTCTATCTTAAAAA

AAGTGGATTCTGGAAAAAAGTATTTTGAACAAATCGAATCTCAGGATTATGTTGAATCTT

TAACTGACCAGGAAATAGCTATCTTAAATTTGAGTAAGAAAGGATTCTCTATAAAAGAAA

TAGAGGAAACGTTACAAATTAGTAGGAGAACAGTATTTAATCATTTGACCCACATCTACT

CAAAGCTTTTGGTAAATAATAAGCAAGAGGCTATCTACAAAGCCGAACAATTAGGGTATT

TTATGGATTTCTGAGTTGCAGATAATCAAACGACATAAGTTTTACTTGTGTCGTTGCTAG

GGATTTCCTGTTAAATACCCAGATTTAAACTCATTGTTTTTGAGTTCTTTTTGATTGAAA

TTGATTGAGATAACGCTTTTATAAAAAGTAGGGCAATGCCTGATATGAAAAGCTTTTTAA

ATTTAGTCAAACAAAAGTTGTTTAAACCAGGTCTAAAAAAACTCGTAAAGCTTCACAACT

CCCAGAACGTTAATATATGCTTATATATCAACGATTGGAACTAATTTATGGTTCGCACCA

TGGTTTTTGTGGAAGGATCAAAAGTTGTCCTGAAAATTTCTCTTAACCGTGTTTAAGCTT

TCTAAACGTTTGAGAAATCATTGTAACTTCAAATAACTATTTTAAATAAAACAAAGAAAC

GGACAAAGTAAAGTTAGATGAGATGATTCTATTTTGATTTATAGGTGAAGAAGGAGAATC

TTGTTTGATAAGTACTCAATAAAGGTTCACTACTCAATTTTAAATAAAACGCCACAGTAG

TAATCATCTACTGTGGTGTTTTTTTGTCTTTTAGTTTCTAAAAGTATCCAATTCGTCTTG

TGCTTCTTTGGTTGTTCCGACTAGTTGTTTCATGAGTTGATCTAGTTTGGCATTTGTTTT

GGCAATTTCTTGATCAATGTCACTGAGCTGTTTAGCTAATTCTGGCAGTGGTTTAACTGG

GACTTCCTCAAAGGTATCAACATAGCGTGGGATATTTAAGTTATAGTCATTTTCAATGAT

TTCATCAAATGAGGCTAAATATGAAAACTTATCAGAATTATCACGACTCTTATAAGCATC

AAGGATCTTTTTGATATGGCTATCTGTCATTGTGTTTTGATTTTTGCCTTTATCAAACTC

TTTTGAAGCATCGATAAAGAAGACATCTTTGTTGGTTCTGTTCTTCTTTAATATGATAAT

AGTCGTAGGGATGCTTGTATTATAAAAGATATTTGATGGGAGTCCGATAATGGTATCGAT

AGCCCCTTGTTCGAGTAGTTTTTGGCGAATCTTGCCTTCTGCAGCTCCACGGAAAAGAAC

ACCATGTGGTAAGACAATTGCCATAGTTCCTGTATTTTTTAAATGATAGAAACCATGAAG

TAAAAAGGCAAAGTCAGCTTTTGATTTTGGTGCTAATACACCATAACTTGAAAAACGTGG

ATCCGTTAAGAAACCAGCAGTCGCTGACCATTTCAAAGAATAAGGTGGGTTCATCAACAC

CCCATCAAAGTTGGTTGGCTCATCTGTTGGCCAGTCTGCATCCAAGGTATCAGCGTTACT

TAAGTGTTGGTTTTCGATGGCAACGCCATGAAGCATCATGTTCATACGAGCCAAGTTATA

AGTAGAAGTATTGATTTCTTGACCATAGTAAGAAACGGTATCGGATTGGTTACTATATTT

CTTAGCATTTAAAAGGAGTGATCCAGATCCCATAGCAGGGTCATAGAGGGTCATTCCTTT

TTGATCTTCACGACCTAAAAAGACAATCTGAGTCATCAAGTGAGAGACAGCTTGTGGGGT

ATAGAACTCACCTGCTTTTTTTCCTGACTCACTGGCAAATTCACCAATCAAGTACTCATA

AGCATCGCCTAAAGTATCCCCATCTACTGCCTCAAAGTCAATTTCATTTAATGTTTTCAT

GACATTAGAAATGGTTTGATTTTGTTTTTGAGGAGTAGAGCCTAATTTTTTAGAGTAGAG

GTCAATATCTTCAAATAAGTTTTCAAAGTCTTCACCACTTTGTTCAATATCGCGAAAACC

TTGAGCTAATGATTCTAATTGGAAAGTATTGTGATAAACATCTTGGATGAGTTTTTCAAA

GGTCAGTGTTGGTTCGATAAAGTAACCTAAATCACCAGTAACAACAGAAATGAGGTCATC

TTTTAGGCCTTCATCTTGATAAGCATCTTCAAAGATTTTTTGAGCCTCTGTAAAGGTATT

GAAGTGTTTTTCTAGATTATCACAAACAGCCAAGAGTAATTTATCAGATAAGTGTTTGTA

AAAAATAAGTCCAAGAAGATAGTTTTTATAGTCATTGGCATCCATTTGGCCACGTAATTG

GTCGGCCGAATGCCATAGTGCTTGACGGAGTGAAGTTGTTTTCTCTGCCATGTGAGTCCT

TTCTTATACGAATAAACGATTGAGTAATGTTTGTTTGAGTGCTTTAAGTTCTGTTAATTT

ATCTTCGATTTGACTCATTTGTTGGTCTAGGGTTTGGAAGAAGTCACCGATGGCTTCTTG

TTCGGGGAGGGAGGGTAGATAAAGCTCTAATCCTTTCACAATGGAACCAGATAAATTTCC

TTGACCACCCTGTAAATATTTCTCAATAATTGAAGATTTTTGTTTATATAACCAATTTTT

TATAAAAAGAGAAGAATATTTTTTCTCAGGAATAATGGCTAAAATTGCTTGATTAATAGC

TCCACTTATTCTTGAAAGTCCAACTTCTCCACTAGTTGCACCGTAGAGAGCGTATAATAA

TGTATTTTTTTCCACTATTTTTGCTGATGAATTTGATAGACCTTTGTTTGTGATAAATAA

CTCTGTTTGATCACTATTAATCTCAGAAGATCTAATGAAAGGAATATTACCGTTATAGTA

TTCAGAAATACCTACGCTAGGAGTACCACCTGAAAACATCCGAGATATCTCTCCCAATTT

CTTCTCTTCCCACTCACCATCAAAACCTTGTAAACGGATTTCAGGGACTTTTTGTCCTTG

AGCTGGGAACATCTTTCTGAGGAAGGTTTGCTTTTGTTCTTTTAGAGTTGCTAGCTTTTG

ATCCTGCAACTGAATCAATTGATCAACCGTTTGGAAAAGTTCGCCAATAGCTTCTTGTTC

GGGGAGGGAGGGAATTTGAATTTTAGCATATTTGATATTACTAGAGCTAATGCTATCAAA

AGTTGAACCTGTACTAATTCTTTTCCAATAACCAATTTCTTTCAAGTATTTTAATATTTG

AAAGATAAATTCATTACCTTTTATAGCAGCAACTCCACGCCCAATAATAACATGATAATT

TGTCTTTCCAACATCACCAACTGGAGCCCTAACACTAAGAATAATATCACCTTTGTCAGC

TTGTTTTGTAATTTGTGTTGTCCAGACACGCGGAAAAACATATCCATTTTTTATATCGGC

ATTTCCTTGTACTAGAATATAATCAGAAGGGTTTGTAGTATAATTTTGACTTGATGGAGA

TTGGCCCATTGTAATTTGAACAATATCCCCTAGTTCTTTTTCTTCCCACTCCCCTTCAAA

ACCGTCAAAGCGATATTGAGGCTGTTTAGATTTTGTCATCTCTTTCCTTTCCTAGTGCCC

TTTTAAGGGTTGGATTTGATCTCTAATGAATTGAACTAACTCCCGTTCAAATTGGCTCTT

GTATTTGAGTTTATTCATGGCAGAATCACCTTGTTTTTCTTTATAGGCTTGGTAGTCTAG

GGATTCTTTTAAGGTTGACATGCCCACTTGTTTGGTAGAGTTAGCACTATGATAATTAGT

CGCCACAAAGGCTAATTTGTCAGGATCTGCCTTCCATTGGTCCGCAAAGTGTTTGATGGC

CCTTTGGATCTTATCACCAATGAGGTTATCCAACTCATAAGCAATGCTTTTCCCTTCATA

GGCTTTGGATTCTTTTTGGATATCTTGCCATAACTCAGCTAGTGAGTCAGCCATAGCAGG

GTTTGATTTTGCCAAATCTTGGATGTATTGATCCATGAGATTGTCATTGAGCCTTTCTTG

AAGAGCTTCTTGTTCTTGATCGACAAAGGCTTGAATTAAGGTCAAGATATAGTGATAGTT

GATATCATCCATCTGAACACTTTCTAGCTCATAGTCGATATTGATCTCTGGGATGGCTTC

GTCATCTTCTCTTCGTTTTCGTATTTCTGCAATGACATTTTGGTAAGTGCCTTTATAAGT

ATCAATCACTTCATCTGATAACCCAACCTCACTCAACAGTGTTTCATTAAAGTCACTATA

GACTTGTACAGATGCAAAGAGTTTATCAAATGCTTGAAAGGCTTTGGCGTATTGTTTGAG

AAAAGCTGTCTGTGCGCTCTCAATAGTTGGGAAAGCGTCTGGATCAGGAACTATATTTTT

TAATACTGTTACTTTTTCAAAGAATCTTGCTTTTTCTTCTTCCCAAGAAGGGGCCAAAAC

GTCGTTTTCACCCCCATTAGAGTAGAGGCTAAGCGCTTTGTCAACAGCTTCTTTAAAACG

AAGTGGTGTTTGGAAAGTCACAACCTGACCGTAATGTTTTCTACTTTCAAAGATACGGTT

GGTTCGTGAGAAAGCTTGAATAATGTGCTGAGGTTTCATAGGCTGGCGATCAATAAAGAT

CGTTGATAAACAAGGCGCATCAAAACCTGTTAAGAGACGGTCAACCACAATCACGAGGTC

TAATTGTTCATGACGGTCTTTGAATTTATCTTTTTTACGGGCTAAGCGATCATTTAAATC

TCTGTTATAGCCTTGTAAGTTATCGATGGTAAAGTTAGTGCCAAAGAGGTGATTATAGTC

TTCTAGATTTTTGGTCATTTTATCTTGTCGGCTGATAGACGCATTGTCATTTTCAGTGAT

AGAATAAGTAATAGCTACCTTTGGAAAGTCTGGTAGTTTTTCTTTGACCTTCTTAGAAAT

GACTAAATCCGTTTCACCAGCTTTAACTTTCTGAATCAGATCATAATAGGCTTGGGCACG

AGCAATACTCTTAACGGTTAATAAGCCTTCGAATGTCTGACCAATCCCATTATTAAAGCC

AAGCTTCTTACGTGATTGATTAATGATACTATTTAAGACAGCTAACATATGTTCTTCATG

ATCATAAGCTTCTTCAGGAATACTGTCTTCAGGCATATCAGGAATGGTTGTCTTATACTC

AACTTGGAAGCCTAGAACCGCTTTATCGTGAATGGCTTCTTTGACAGTGTATTGATGGAG

ACATTTGCCGTATTGCTGTTCCGTTGTTTGGGCTAAATCACCTAATTGAGCACGCTTATT

TTCCATAAAGATAGGAGTACCTGTAAAGCCATACCAGCGACTGTTTCGAAAAGTATTCGT

GAGATGACGTTGACGTTCTGGCGTGACAGCACGGTGGCATTCGTCTACAACAAAGACGAC

ATTTAGGTGTGCAAGACGCTCTTTTAGTTTCTTGAATTTTGGAGTGTCATAACTTTCCAT

CTGACTGATCATAGCATTGAGTTTTTGAATGGTGGTGACCACAACACGACGATCACTGGA

TTCTAAATTTTTGATGAGTTGTCTCGTATCTTCTGTCTCATCGACATCAAAGATATCATT

TTGCGCATAAGATTGAAAAGCAGAAGTGGTCTGATTATCTAAATCCTTTCGGTCAATGAC

AAAGATGGATTTTTCAACGGCCGGTATTTGAAGGATATTGCGAGCCACTTTATAGGAAGT

TAAGGTTTTTCCAGAACCTGTTGTGTGCCAGATATAGCCAGACTTGCGGTGACGACTCGC

TTCAGCGACGGCTTCGATTGCATGAATCTGATAAGGACGAAGGAGAATTAGTGCTTTTTT

ATCATCATCAATGACAGAGTAAGTCATGACCATTTGGTGAGCACGAGGGATAGATAGGAC

TTCTTTGGCAAAGGCAAAGAGATCTTTTTGTGGTTTGTTGTTTTGATCTACCCATTGGGT

CAAGAAGTTAGGGTTTAATTTATTTTCTTTAGCTGCAGCGATATACCTTGTATCTGTTTT

ATTTGAGACCACAAACATTTGTAAGGTTGAAAAGATACCTCTAAACTGGCCTTCTTTATC

ATACTTTTTGACTTGGTTAAAAGCTTCAATGCATTGATGATTCTGAGATTTTAATTCAAT

TTGGATCATAGGTAAGCCATTGATGAGAAGTGTCACATCACCTCGTCTATCACGAGAACC

AGAGAAAGCAACTTGATTGGCAATCTCATAAACAGAAGTTCCACCAGCCACATTGTCTGC

TTTAACGACTTCTAAACGAATCGTTCCTAGTTTTGCATCTTCGCGTTGCACTTGTACTTT

AGCAATGCCGTTTTCACCAGCTAACCATTTAGCCGCTTCATAGTAATTGACAAAGTTAAG

TTGATTCTTAATCTGCTCTTTTTCACTAGCTGTTAAAGGCTCCTCGTTCAGATATTGCGT

ATTATTTTGCGCTAAGATCTTAAAGAAATTATCCCATAAAGCATCTTCAGTTTTTAATTC

TTTACGATATGTCCATTGACTTTCACCTGTTTCTAAGAGATGGATCAACTCTTTTTCAAG

TTCAAGTTCCGTTTTAGTTTGATTTCCCATTTTTAATCCCCATTCGACATGATATAGTAA

TATTATATCATTGGCGGATCCTTTTTAGAATGGCAAAGCTATTGAAACCTTCCAAACAGT

CTTTTTTTGATTAGTTGAATGCTTAAGATCCTAGCAACTAAACAAAGCTATGACTATGTA

AATGCAATCGAGAAAGTTCTTGTATTTTTGAGTTTGCTTGACAATAATTTAAGGTGAAGA

AAGTAATAAGACAACATTAGTAAAAAGGTCGAGTTCTCAAAATAGATAAAAAACTTAAGA

CAGATCTAGCTTGACTATTAATAGGGTTTTAAAGGACGATAATCTGCTACAATATACAGA

AAAGGAATTTATTGAAGATATGAAAATTTTGGTAGCCATAGATTCTTTTAAGGGATCGGT

AACGTCTCCAGAACTTAACACTAGTGTAGCTCAAGCCCTTCTTTCTGTCGATAAGCAGTT

AGTTATTGAAACAAGAGCTATCGCTGATGGAGGAGAAGGAAGTTTAGCAGCCCTCTCACA

AACCGTGGCGGGGAGGTGGCATCAAGTAAAAACCATTGATCTATTAAGACGCCCGATTAA

AGTCGCTTATTATCGTCACGCAAAGCAAGCTTTTATTGAATCTGCGAGTATTATTGGTAT

TGATAAGATCACCCCCAATTCAGTAAGCTATGCACAAGCCACTTCCTATGGTTTGGGTTT

GGCAGTTAAAGATGCCATTCAAAAAGGAGCTACTCAAATCGAAATCATGCTAGGAGGAAC

AGGCACTTCTGATGGTGGCAAAGGGTTTTTGGAAAGTTTAAACTATGATTTCATGACAGG

AAGATCTTATCTTGATACTTTAGCATCACCTGTTACGCTATTAGGGCCGACTGATGTGAC

CAATCCATATCATGGCCCTCAAGGATTTGCTGCGGTCTTTGGCCCTCAAAAAGGTGGGAG

TCTTAGTCAAATTGAAGAAACTGATCAAATTGCAAGTAACTTTGCTAAAAAAGTTTTTTG

CCAAACAACCATTGATTTACAGACAATACCAGGAAGTGGAGCAGCTGGAGGATTAGGAGG

TGCCATTGTCCTATTAGGAGGCACCTTAACTAGTGGTTTTAGCAGGATTGCAGAGCTACT

AAATCTGGATAATTCCCTTCAGTCTTGTGATTTAGTTATTACTGGAGAAGGATGCCTGGA

TACACAATCACAATCTGGCAAGGTACCTGTTGCGATTGCAAGAATGGCTAAAAAATATCA

AGTTCCAACGATTGCTCTATGTGGTTCTGTAAAGATTGAGACAGGACTAGCAGCAGAAGA

CTTTTTGGCTGTTTTTTCAATACAACAGCAGCCAATTTCCTTAGAAGCAGCGATAGATAA

AACAACAACACTTTCTAATATCAAAATATTAGCAGCTAACCTTATGCTATTGATAGCTCA

GTTTAATAAGTAAACTCATCTATTTTCGATTGTGTAGCATTCATTTGGCGGTTAAAAGTA

ACCTATGAAGAAGGAGACGCACGAGCTAACTCATTAGCATAGTATGAAAAACAGAAACAG

TTTGTAAAAAAGAGTTGACGATGACAGTAAAGATATCGTATACTAATAAAGCTTGCATAG

AGGTTCCTGTAACACACT

>18S-48_L8_1_2

TTCTTCATGCGTTTGGCGTGGTAAAGTTATCAGAGAAACAGAGAGTCATAATTTTATCCT

TTTTTCTCATATTGATACCATGGTATTAAACGAGTTGATTGGGGTGACTCACAATCATTT

CTAATTGACCATCAAATGACCACGATTTAACATCGTTAGGCAAGATAAAGTGCATGCCTT

TTTCTAGCTCATAAGCCTTCTGGTCCACATAGAGTTTTCCTTGACCTTTTAAAACACTCA

CTAAAAGATAAGGCGCAGCTTGTTTCATGTCAACCATTTGACTAGTTACCCATTTGTAAA

CAGTGAAAAATGGTGTTGAGACTAAAGTTGTTGCTACCATATTATCCAAAACCATAGTGG

CTGGCACGCTATTTTCTGGCTTACCAATCGTTAAGACATCAATAGACTTTTCAATATGAA

GGTCACGAAGATTACCATTAACGTCCTTACGGTCAAAATCATAAACCCGATAAGTGGTAT

CAGATGACTGCTGGGTTTCTAAAATAAGAATTCCCTTGCCGATCGCATGCATGGTGCCAC

TTGGAACATAGAAAAAATCGCCTGCTTTGACTGGGACACGAGTCAGCAAATCATCCCAAG

CGCCTGCTTCAATCATGGCACGAAGGTCTTCTTTGGACTTGGCCTGATGGCCGTATACAA

TTTCAGAACCCTCTTCGGCAGAAATAATATACCAGCATTCTGTCTTGCCTAATTCTCCTT

CATGTTCACGTCCATAAGCATCATCAGGATGAACTTGGACACTTAGCCAGTCATTAGCAT

CTAAAATCTTAGTTAACAGTGGAAAAACTTCTTCCTTTGGATTCCCAAACAAGGCCGGCT

CTTGCGCATATAATGTGTTTAAAGGTTGTCCTTGATAACGTCCATTAGTAACTGTCGAAA

CACCATTAGGATGAGCAGAAATAGCCCAATACTCACCTGTGGTGTCACTTGGGATGTTGT

AAGCAAAGACATCTCTCAACTTGGTGCCACCCCAAATCCTGTCGTGCATAGTTGATTTTA

GAAATAATGGTTCTGACATAATATTCCTCGCTTCTGATGTTAATCTCTGTTTATTATAGC

ATGTTCTAGCTGACTGCGCTTTCTTTTACTAGACAAGCAGGCAAAAACAGATCCTATTTT

CCTTGTGCAGCCAACTTGGCCAAAGCAAAATTTCCCAGAGTAGCGGAGCCATTATCAGCA

ACAGCTGGAGTAACAATGTAGTCTGTCAAATCAGGGACTGGCAAATAACCGCTTAAAAGG

GTAGTGAATTTATCATGCACGCGCAACACCATATGTTCTTGCGCCATGACACCTCCGCCA

AAAACAATCACTTGAGGACGGTAAAGCATAGTTGCTTGCAGCGCTGCTTGGGCAATGTAG

AAGGCTTGAATGTCCCAAACATCAGCTTCCTGATCAAGCCGTTCACCTCTCACTCCCGTT

CTGGCCTCAATACTTGGACCTGCTGCCATCCCTTCCAGACACCCTTTATGAAAAGGGCAA

ACACCTAAAAATCCCTTGGCCATATCATCAGGATGGGGCATGACATAGGTATGACCCGCT

TCTGTATGACCTAAACCCCCAATAAAGTGGCCATGTTGAATAGCTCCCGCCCCAATACCA

GTACCTATGGTGTAATAGACCAAACTTTCCACACCAGGACGAGCAAGCACCTCACCATAA

GCAGAGCTATTAACATCTGTTGTCACATCAAAAGGAATCTTAAAAGCAGCCGAAAGTTGT

CCTAACAAATCCACATTAGCCCAGCCTGACTTAGGAGTGGTTGTGATGTAACCGTAAGTC

TCTGACGACGGATCAATATCAATTGGTCCAAAAGAACCAATAGCAATACCAGCCAAATCT

GCCTCAAAAGCTTTAAAATAAGCGATGGTTCGCGCGATGGTTTCTTCTGGTGTGGTGGTC

GGAAATTGTGTTTTATCCACCACTGTAAACTCCTCATCACCCACGGCACAGACAAACTTT

GTCCCACCAGCTTCAATGCTTCCATATAATTTTCCCATGTTTTCTCCTTTTTCTTGTTCT

TTTTTATCTTTATGATGACACAGGATCTTATCAAGACAGCCATGTCTCAAGGACAAGCGC

CACTTTCTGGTAGTCTAACGATTATTTAGCTTTATGTCATCAAAACCTATAAAAGACTAA

AAATCTGGGACTATTCATCTCAGATTTTTAGTCTTTTGGTTAATCAATTCCAGCAAACCT

TTATCATAGTCACATTTTGTTAATTTAATCTGAATGTTGCTATCTAAGAAAGTTACTTTG

TTTATTTTTTTAGCAGCTGCCTTTTCTCAATACAATCTTTCAAAACTCCAGCATTAGCAT

TAATAGCAGTTACATCAAAAATCTTACTGTTCAAAGAAGTTTCTAAAGCCATCTCTTTTA

TTTTTAACTCATCAAGCATCTTTTGAGAAAACTTATCTTTTTGTTGAGATAACTCTTTAG

CAGTAGTTACTGTTTTCATGATAGTGTCGGCGTTAGGTAACGGATAACCTAAAATTTGGA

GTTCAGGGACTACTGGCGAACTATATCTATCTCCTTTAGTATCAAAGACAACTCGCCAAT

ATTTACTAGTAATATTATTTAATGTATTACTGAATGCAGTAGCTCTCTCTCCTTGTGCAC

TGTAAGTATCTACAGTAATCCAATATTTTTCATCGTCAAATTTATTGGGATTTTCCAACA

AATTATCTAGATTATAATCTTTGATATTAAAAATTTGTAGACTTGCTTCCTGAATAGGTT

TATTGGTTGTCTCAGGATTTCGGGCTGAATCATTGAAGAAACGCCAATGCTTTATTAATC

CATCTTCTTTCAATTTAAATATAATACTTTGCTTAGAATCCCATCCTAAAGAGATATTAT

CAGTCTCACTGCCCAGTTGCCCATCAAATACCTTTCTTGCATTTACAGGATCAGCACTTC

CTCCAATAACTGTTGCGCCTTCTGCCAAATTAACCATCATGGTTTTTTCGTCACCAACAA

TCACTTTAGCAGTATGAACAGCTTTTGTCTTATCTGCTGGGCTAAAGAAGTCAACCTTAT

AGGTCTCTTCTTTATCAGTTGCTAGCGTTTTGTCAGTAGTGGTTCCCAATGTGGAATCAG

TTACTTTAACGGTATAGTTCTCATAAGAAACTTTAAAGTTATTGTAATGATAGTTTGAAT

CAACAAAGCTACGTCCAGCAATTTTATGATTTTGGTAAGCCTTATAGTCTGCTTCGCTAT

TGATTAGGGTTCCTTGATTCGTCACAGTCCCAAACAAAAGCTGGCTTTGCAAATCAACTT

TTTCATTTGCCACTGGTAAGCGCAGACTAGTTTTCCCATAGGTATCTGGGTAATGTCCAG

TTGGTTTTTGCTTGTCAAATTTCACTGTTTGTTCATTGCTTCCAACATGATTGCTGATAA

TTGATAGCATAGTATCAAAAATTTGTCGATTTTCTGTTCCTGGAGCCAAATCAAGTTTGT

TGCCAGAAATATCAACTTTTTCTAAAGACGTTAGAGTAGCGGCATCAAGACCAGCCAAGG

TTTCACGGTCAAAACCTGACAAATCTAATTCTTTCAGACCAGTTAAACCAGAAACCTTCA

AAGATACTGGTGGGATAGTAGCAGGTTCTTCTTTGTTATCCTTTTTATAGGTTTCAAGAA

CTGTTTCCAAGGTATCTTTGCCTGGCTTCATATTAGCGGGTAAAACAGATTGGTCAAGCT

TTGTAATGCAAGATAAGCCAATCAAGTCTAATTGAGCTAATTTTTTAAATTTATTTAGAC

CTTCTAAACTTTGAATCGCTGGATTATCCAATCGCAATGTGCCATTGAAACGTTCCAAAT

CACCTTTTCTAGTTCCAACCTGCGCCATCACAGCTTCTCGCAAAGCCTTATCTGGGAAAT

CTTTCTCATCAATCAGATCATACGACTTATCTTTTAGCATAACTGTCTTTAATGCCTTGG

AGACACTATAATCTGAGTGGAAAATGTTATCAGTAATTTCTGGAATCTGAGGCTGATTAT

TTTTTACATTCTGTTTATCTTTTTCAGCTATTTGTTTGGGTTGATGAGCTACACCATCAC

GGTCAATAGCGTAGGAGAAGATACCTCCCTTAACCCCACCTGTCTTAGGTTGCCACCTTG

CATACCGTTCGGCACGCGTTCCAGTTATGTCAGTGTTAATTCCATTTGCTTTGTCATCGT

CCTTTCGAGAATTAATATCATACCAAAGATTGCCGCTGCCTGCTCTTTCCTCATAGAAAG

AAAAACCAATCATGTATTGTTCAGGACGAATATACTTGCTATAACCTTGCCATCGTTCTT

CAGGAGTCTCGGTAACTAACTTGGTATCGTTTTGAAACTCACCTTGTTCTCCTCTTGCGC

CATAGACTTGTACCAATAACAAATCAATATAAGGAGCTCCTCGCTCAATCAATGGGTTTT

TATCAGCCATATAGGTGCTATCCATAATAAATAACCGCGATTTATCAACACCTTTTGGTC

CAATTAATTTCCCAATTTCTTCAAAAACATCAATAGATCGCTTGAGATTTTCGTCACTAG

CTTCTCCATTTACTTTTGGAATACTATCATGTTCAACATCCACATCTAAGCCATCAAGGT

TGTACTTATAAACATATTCATCAACAATAGCTTTGGCTAAAGCTTTATTTCCCTCTGGTG

TATTTGGGTATTTACTGGTATCTTCTGCAATACCACTGTTATCACCTCCAGCTAGGAAAC

GCCATGGAATGGTACGAATGACACGTGTCCCTTGCTTGTTTAACTTTGGCACATGTTTGG

TGGCCAATTCTTTCCAAAAAAGGCTATAATCTTTTGTCCAATCGTGGAAAATAAAGGCTA

GATCTACTTCTTTAGGAAGCTCTCCCATCGAGTTAACTTTGTCTTTTTCTGTTGGATCTG

ATGTTTTGTCATGCCAAGTTCTAAAGTAACCACCGTAGAGAGGACCATGTAACGGTTTCA

TCAGTATTTTCTCAGGAATTTTCATTTTGGCAAGTTCTTGAGCTTGTTTATCTGCCTGCT

GAGCTTTTGCTAATATCTCTTTGACCTTTTGAGATTCTTGCCCCGCTTTTGAGAGTTCTT

CTTTAAATTCTTTTTTGCTATTCTCTGACAGATAATGCAAGCTATCTATAGAAGATAATT

CTTTCTGAACCTGAACAGTCTTCTCCTCCGCTTTTACAGTATTGAGTGAATCATGGTGGG

TCGCTAAGGCAGCTCCCATCAACGTTGCAGCACAAACACACCCTAGTGTTCTTTTTACCA

ACAAATGTTTATCCATTTGGACACTCCTTATTTTTGGTACTAAGTCATGGCTTCCCAGTT

GATATACCATATATGTAAGCGCATTCCTATAGTTCGCCTTAAGTATAACAACACTTTATT

AAAAAAACAAGGTACAAAGTATAGGTAAAATAACACAAATTTAAGGTTTTTAGGCTTTTC

TCATTTTTTTGCCAACCTGCCATTCATGGCAGGACAAAAGAGCTGCTTAGTTAGCTCTTT

TGTCCTTATTTGATAAGTGCCACTTGATCACCAATCAAAATATGTCCTTGAGCAAGAATG

TCCACGCTTTGATAGTCAGCGCTGTTGGTCACAATCATCATGGTGGTGTCATCTAGTCCA

GCTTCTGCAATCTTGCTAGGATCAAAGTGTCCAAGAAGATCACCTTTTTTAACCGCCTGA

CCTACTGCTACAAGAGATTCAAAGCCATCTCCTGCCATCGACACGGTATCAATACCAATA

TGAAGTAACACTTCTGCTCCTTGACTTGACGTTATAGCATAGGCATGACCTGTTTCAAAG

ACAATTTCAACTTTACCATCAACTGGTGAGTATAGGGTATTATCTTCTGGCTTAATTGCT

AAGCCTTGACCCATAGCACCTGATGAAAAAACAGGGTCTGAAACAGCTGCTAAATCTACA

ACTGTTCCATTCAAAGGACTATATAAAGTTTCCTCTGCTAAGGCTGGCTGATCAGCCGTT

TGATCAACTTCGACAGCTGGTAACGGAAGGGTTTCTCTATCTTGATAACCCCAAGTATAA

GCAATTGCAAAGGCTACCCCAAGACCGACAAGCATAGTCACAAGGTATTGTAATAATTGG

CCATTCAAGTAAAGGAGAGTTCCTGGTAAGACCGTAATCCCAAAACCAGTTCCTGCAATG

CCAAAGAGTCCAGCGACCCAACCACCTAAGGCACCACCAATAAGACCTGAAACAAAGACT

TTTGGATAACGGAGATTGACCCCAAAAATAGCTGGTTCAGTAATCCCTAAAAGAGCTGAT

AAGGTCGATGGAAAAGCCAAACCTTTTAATTTTGGTGATTTGGTTTTAACAGCAACAGCT

AAGGTAGCTCCAGCTTGAGCAGCTATTGCTGCTGTTAGGTAAGCATTGAAAGGATCTTTT

CCGGTATTGGCAATCAGCTGCGCTTCTAGGAAGTTAAAGATATGGTGAATACCAGTAACG

ACGATCAACTGTTGGATACCACCAACAATAAGACCAGCAATGCCAAACGGCAAATGTAAG

ACTGCCTGTGTCCCAGCCAGAACAAGGTTTTCAAGAGAATGGAAGACTGGTCCAATCACA

AATAGTCCCAAGATACTCATAATAGCAAACGTTAAAAACGGTGTGACCAATAAGTCCAAA

GCTTCTGGAACCTTTTTGTGCAACCATTTCTCCAACTTAGCTCCTACCAGACCAACAAAG

AAGGCAGGTAAAACGGTACCTTGATAACCAACAACAGGAACAAATCCAAAGAAGGTTAGC

GGCTTAACATCTCCACCAGAGGCAACTACCCAGGCGTTTGGCAATTCATTGGACACCAAC

ATCAAGCCTAAAACAATACCGATAATAGGATTACCCCCAAAGACCCTAAAGGCTGACCAA

GCCACCAAAGCTGGCAAGTAGACAAAGGCTGTATCCGTTAGAATACGAGTGTACATGAGA

AAATTTTCCCCGTGCTCATGCACCCCAAATAAATCCATAATAGCTGGCTGGGTCACCAAA

CCACGAACCCCCATAAAGAGACCCGTTGCTACAATAGCTGGAATAATGGGAACAAAGACA

TCTCCAAACGTACGAATCGCCCGTTGGAAGATATTGCCTTGTTTGCCTGCTTCTGCTTTT

TGCTCACTGGTTGATGATGTTGGTAAACCAAGAGCAACGACTTCGTCATAAATGTTATTG

ACGGTACCAGTTCCAAAAATCATTTGATATTGACCAGAGTTAAAGAAAGCTCCTTTAACC

TTGTCAATAGCTTCTGCTTTTTCCTTATCAATCTTTCCTTCATCATAAACCATCACGCGA

AGGCGAGTTGCACAGTGGGCAACACTTCTCACATTTTCTCGGCCACCTAAAGCCTCAATC

ACTTCAGTTGCAATCTGACGATTATCCATGCCTAAAAGTCTCCTTTTTCATTTGGTGTTA

GTCCCTCATCCATTCTGCCAGCTTTGTGGCTAAGAATGTGTAGGACCAACGCTAGTGAAT

CCGCTTCACTTCTTGACTATCATTTTATCACTAATATTTTTTATGTCAAGCGTTTAACAT

ATTTTTATTTTATATGCTTTAAATTCTTGATTTTTAAACAGAAAACGTTTAGTATATTCT

TGAAGACGACTATAAAACAAGGAGATTTTGATGGATCTACCACAAGCTATTCGCTACCGC

CCTTACAAAGAGTGGAGCTCAAAAGACTACCAAGCTATTACTGAAAAAATGGCCCAATCC

CCATGGCACAGCCAATTTCATGTCGAACCTAAGACAGGATTACTCAATGACCCCAATGGG

TTTTCTTATTTCAACGGTCGCTACCATCTTTTTTATCAAAACTGGCCTTATGGGGCTGCG

CATGGTCTGAAACAATGGGTACACATGACATCCACAGACCTCGTCCATTTTACGGAAACA

AGGAGCCGCCTTCTGCCAGACCACGCTCACGATAGTCATGGAGCTTACTCAGGCTCTGCC

TATGCTATTGATGACAAGCTCTTCCTCTTTTACACTGGTAATGTTCGCGATGCCAACTGG

GTCAGAACACCATTACAGGTTGGCGCATGGATGGATAAACAAGGAAATATTTCCAAAATC

CCGCAGGTTTTGATTGAACAGCCCGATGATGTTACAGAACATTTTCGGGATCCTCAGCTT

TTTTCTTATCAAGGTCAATTCTATGCTATTATTGGCGCCCAAGGTTTAGATGGCAAAGGA

AAAATCAAACTTTACAAAGCTGTGGACAATCATGTGGACAACTGGCGATTTATAGACGAT

TTGGACTTTGACGATTCTGGTACGGAATACATGATTGAATGCCCCAACCTTGTGTTTGTC

GATGACAAGCCTGTTTTGATTTTCAGCCCACAAGGACTGGCTAAAGCTGATTTAGATTAT

CAAAATATCTACCCCAATACCTATAAAATTTTTGAAAGCTTCAATCCAGAAACTGGCCAA

TTGCTGGGTGGGGGGGCCCTTCAAAATCTTGACTTTGGTTTTGAAGCTTATGCCACTCAA

GCCTTTAGTAGTCCAGATGGCAGGGTTCTAGCCGTTTCTTGGATTGGGCTACCAGATATT

GATTATCCGACAGATCGTTATGACTATCAAGGAGCTCTTAGTTTAGTGAAAGAATTAAGG

ATTAAAGACGGAATTCTCTATCAAACACCTGTTTCAGCCCTACAAAATCTTCGAGGGCCA

GCAGAATTATTTCATAATAAAATAGACAGTTCTAACTGTTATGAATTGGAATTGACGATT

CCAGGCCAAAAAAAGCTTGACCTGCTCCTATTTGCCGACCAAAAAGGGAATGGCCTAAGA

TTGAAAGTGGATACAACAAAAGGACAGTTAAGCATTGACCGAAGTCGGGCAGGGGTTCAA

TATGCCCAAGACTATGGTACGGTGCGCTCTTGCCAAATCCCACAAAGCCATGTTACACTT

AATGTCTATGTGGACAATTCCATTCTTGAAATCTTTATTAACCAAGGGCAAAAAGTCCTA

ACTAGTCGTGTTTTCCCAACCCAAGGACAAACAGGTATCCAAGTAGTAGAAGGACAAGCT

TTTGGACATTATTATGAAATGAGGTATTAGGCAAGTGGTCGCAAAATTAACAGACGTGGC

AGCCTTGGCGGGTGTCAGTCCAACAACTGTTTCGCGAGTAATCAACAAAAAAGGCTACCT

TTCCCAAAAAACAGTGAATAAGGTGAATAAGGCCATGCGTGAATTGGGCTACAAGCCTAA

CAATCTCGCTAGAAGTTTGCAAGGTAAATCAACCCAACTCATTGGGCTTATCTTCCCCAA

CATTAGCAATATCTTTTACGCTGAATTAATTGAACATTTGGAAATTGAATTGTTTAAACA

AGGCTACAAAACTATTATTTGCAATAGTGAACATAATCCCGTCAAAGAACGCGAATACTT

AGAAATGCTTGCGGCCAATCAAGTGGACGGCATTATTTCTTCCAGTCATAATTTGGGAAT

TGAGGATTATGAGCGTGTCGAAGCTCCTATTGTAGCTTTTGACCGTAATCTGGCGCCAAA

TATCCCGGTTATTTCTTCAGATAATTTTGAAGGTGGTAAATTAGCCGCACAAACTTTGCA

AAAACACGGTTGTCAAAACATTGTCATGATTACAGGTAATGACAATTCTGATTCGCCCAC

AGGGTTGCGCCAGCTAGGGTTTAACTACCAGCTCAAACGAAGTGCAGAAATCATCAAATT

ACCAAATAACCTATCTCCTGTTAGACGCGAAATGGAAATTAAATCCATTTTGGCAACCCG

AAAACCTGATGGCCTTTTCGTTTCTGATGATTTAACTGCTATCCTCATTATGAAAGTAGC

CAAACAACTTCACATTACTATTCCAGAGGATATGAAAGTCATAGGTTATGATGGAACGAC

ATTTATCCAGCAATATGTTCCTCAATTAGCGACCATTCGTCAACCTATTGATGAGATTGC

TAAATTGAGTGTCGAAATACTCATTAAAAAAATTAAAAAAGAAAAAACCAGTAAAGACTA

TATTTTACCGATTACGCTACTTCCAGGAGCAAGTATTTAAACAAAACATAACCAATCACT

AATTACTCTAAAAGACAAACAAGCATACTNNTATGCTTGTTTGTCTTTTAATATTCGTAC

ATAAATTGAAAAAATCGGATACAATCCTAAGATTGCTCCGACTTATTATGATTTATTTGC

TGAAGGAGCTTCCGAGACATATTGGCTAAGTAAGCCATTAATAAATTTAGCAGAGGTTTC

ATCAGAATATTTTTTAGCTACTTCAATGATTTCGTTTAGTGCAACACGATCTGGTGTTTC

GTCAAAATACTTGATTTCAAAAAGACCTAGCCGTAATAACGTTTTGTCTGTTAGTGTCAA

GCGTTCTAACGACCAACCTTTTTTCAAATGAGTTGATATCAAGTTATCCAACTCTTCTTT

ATGATTATTGACACCCATCACAAGACTTAATAAAAAAATCGGCAGCTCCAATACTTGAGC

GTCCTCCCCTGTTACTTTATCATAACCATAGGCAAACTGAGAAGCTGCCAATAACTCAGC

TCCCATCTCTATGTTAAATAAAGCCTGAAAGGCACGTTCACGCAAATCTCTTCTTGAATT

TTGAAAGCTGTTAGTCATCCAAGAAATCCTCATCAAAAAGTGACTTCAAATCTGGTTTTG

GTGTCTTTTCAGCAACAATACCTTCAACATGGATATTAACTGCAGAAATAGGCACTTCTG

CCATATCGTACACAGCAGATTTGACTGTTTTTTGAATATTCATAGATACAGTCGGGACCT

TAACTCCATATTGCAAGTAAACATAAATATCTGCTGTAACACTTCCATCTTCTTCTGTCT

GGAGATAAACCCCTTTACCAAGACTTGCTTTGTTAAAGCTATCTGCCATTTTCTTGTTAT

GGAGTGAGTGGACGCCCTCAACTTGTGTCGTAGCAATCCCCGTAATAACCTCAAGCACTC

GTGGTGAAATGACGATTTCACCGATATATTCAGTTGTCATAGCATTTCCTTTCTCTTTTT

TAGACTAATCTGTTTTGACAAAGGATTAAGCACGAGAAACGTAAGTACCTTCTGCAGTGT

TAATGATTAGTTTTTGGCCAGCTTCGATAAAGTCTGGAACGTTAACAACAAGTCCTGTCT

CAAGAGTTGCAGGTTTCCCTGAACCCGTCACTGTCGCTCCTTTAATAGATGGTTGTGTTT

CCGCAACGGTCAATTCAACAGTTGTTGGAACCGTTACCCCAATCACTTCACTTCCATAAA

ATTGGATTTTCACGTCTGAGTTTTCAAGAATGTAAAGCAATTCTTGCTCAACGTTAGCAA

CTGGAATTTCGTACTGATCATAAGTATCAGTGTTCATGAAGTAAGCAGTGTCATCCATTT

TGTATAGGTATTGTGCTGGGACAGTTTCAATGATGGCTTGCTCAAATTTTTCATCTGGGC

GGTAAGTTGTGTCAAAAGTAGAACCTGTACGCACATCACGTAGTTTCATACGCATGATAG

TGTTTCCTTTACCTGGTTTGTGGTGGCTAGCTTCAAGGACACGGATTAATTTTCCTTCTG

CTTCAAATGTCATACCTGCTTTAAGCTTACTTGCTTCAATCATTATTATTACCTCTTACT

AAAATATTTATCACTTCATTTTACCACAAAAGTGGCCTTGGCTCAAATCTTTTATGACCG

TCCTTTTAGGTACACTTTCTATTGTTCCTTATCGCCTCCTAAAACAATTTGAGGCACATC

ATGTTGCACATAGGCAACCCCTTTTTTCTCCATCAGTTCAATGGCAAAGGGGTGCGGACG

GTAATGTGCTTTATAGGTAATCTTTGTAATCCCCGCCTGCAAAAGCGCCTTAGTGCAGTT

AATACAAGGAAAATGAGTCACGTAAATTTCAGTTCCGTCCGTTGAAATTCCTTCTTTCGC

ACATTGAATCAAAGCATTCATTTCAGCATGAACAGTGCGAATACAATGACCGTCTTCCAT

ATAATGACCCGCTTCGTTGCAGTTATCCGTAGCAGAAACACCGCCATTGTAACCTGTGGC

AATAATGCGGTTATCCTTTACCAAAACAGCTCCTACAAAAGCGCGATCACAGGTAGAGCG

CTTGGAAATCAGCTCTGCATTGGCCATAAAATAGTCTTGCCATGATAAACGATTTGTCAT

TGCTTCTCCCCTAATCCTTTCCTGAACCATTCTTTTACAATACAATTAATTCTTTGGGTG

CCAAGGTCAAGACTTGACAACCAGTTTTTGTGATAACCAAGTCGTCTTCGATACGGACAC

CATATTTGTTATCCAAATAGATACCTGGCTCATCTGTTACCACCATTCCAGCTTGGAGAA

GTTGCTCAGATTTCCCAAAAAATGGATTCTCATGGATGTCAAGCCCGATACCATGACCAA

TGCCATGTGTGAAGCGACTGCCATAACCCGCCTCAGCGATGAGTTGGCGCGGAATACCGT

CAAAGTCACTATAAGTCATGCCAGCGCTAGCTTTAGCAATTAAAGCCTTATTAGCAGCAA

GAACAAGAGCATAAATCTCACGTTCTTCATCAGTAACTTGGCCAATATGAATGGTCCTCG

TCATATCACTAACATAGTGATTGTAGTAACACCCAAAGTCCATGGTCAAGCTCTCTTTAT

TCTGGATAACCTTGTCACTGGCGCGTCCATGAGGCATGGCAGAGCGATAGCCTGAAGCTA

CAATGATATCAAATGATGTGCCGCTGGCACCATACTGACGCATACGAAAATCTAAAAAAT

TAGCCAGGTCACGTTCAGTGGTTGTCCCTGGTTTAATAAAATCAAGAGCATCTTCAAATG

CTTTGTCTGAGATCGAGCACGCTTTAGCAATGGTATCAATTTCAGAAGTATCCTTAATAA

GACGTAAATGCTCCACAAAACCTGACTGAGCAAGCAAGGTTATTCCTGACAGTTCTGCTT

GCATGGCCTGATAAAAAGAAAACGATACCTGGTCCTCAAAACCAAGGCAATCTATTTGAT

CAGCCTCTAACAATTCTGCCACAACCTTAAGCGGCGTGCGGCTTTCGATAATATCAAATC

CCTCAACACTAGCTTTAGCAAGCAAGGTATAACGTGAATCTGTGATCAAAACATGACGTT

TGGCCGTTATCAAAACAGTTGCTGCAGTTCCAGAAAAACCTGTCAAGTAATAACTATTGG

TTAAATGGGTGACCAGAAGAGCCTCTAGCCCCTTCTCTGCCATCTGCCTTAGGCAGTGAC

CTAATCGTTGCTCTAAAAATCCTGACATCGCGAACTCCTTTGGTAACTTTTGAGAATATT

GTAGCATGAAAAAACTACTTTAAGTTTACCATGATTTGAGAGCATTTAAAAGATTAAAAA

GGAGCTCAAACGAACTCCTAATCCTATTGTTGTAGTTTTACTTTCAAATAATGACCAGTA

TAGCTTTCTTTAACTTGAGCTACCTCTTCTGGTGTCCCCGTGGCAACAATCTGACCACCT

CCGACACCACCTTCTGGTCCTAAGTCAATAATATGGTCAGCAGATTTGATAACGTCCAAA

TTATGCTCAATCACAAGAACCGTATTACCATCGTCTACAAAGCGTTCCAAAACTTTTAAT

AGCCTTGCAATATCATCTGTGTGTAAACCAGTCGTTGGCTCATCTAGGATATACAAGCTC

TTACCTGTCGAGCGTTTGTGCAATTCACTAGCTAATTTCATCCGCTGCGCTTCTCCTCCA

GATAGAGTAGTAGCTGGTTGACCCAAGGTCACATAACCCAAACCAACATCCTTAATGGTT

TGAATTTTTCGGGCAATTTTAGGGATAGCAGAGAAAAATACCAAGGCATCATCGACAGTC

ATGTCAAGCACTTCAGCAATATTTTTTTCTTTATAATGCACCTCAAGCGTCTCAGAATTA

TACCGTCTGCCGTGGCAAACCTCACAAGGCACATAAACATCTGGCAAAAAGTGCATTTCG

ATTTTGATAATCCCATCACCTGAACAAGCCTCACAACGCCCACCTTTGACATTAAAAGAG

AAACGGCCTTTTTTGTAGCCATGAATCTTAGCTTCGTTGGTTTGGGCAAACAAGTCTCGA

ATATCATCAAAGACACCCGTATAGGTGGCTGGATTTGACCGTGGCGTCCTTCCAATCGGA

CTTTGGTCAATGTCAATCAAGCGTTCAATATGTTCAATACCAGAAATAGAGTGATATTTA

CCAGGCTTATCAGCATTACGGTTTAATTTTTGGGCGACAGCCTTTTTCAAAATACTATTA

ACCAAGGTTGATTTCCCTGAGCCTGATACCCCTGTGACCGCAATAAATTTACCCAGTGGG

AATCTCACGTCGAGGCTTTGAAGATTATTCTGCGCTGCTCCTTTTATTTCAATGAATCGG

CCATTACCAGAACGACGCTCTAATGGCACTGGAATGAATTTTTTACCAGACAAATATTGA

CCCGTGATGGATTTTTTATTCTTAGCCACTTGTTTCGGCGTTCCAGAGGCAATAATTTCT

CCACCGAACTCACCTGCCCCAGGCCCCACATCAATCAGCCAGTCTGCCTGCATCATGGTA

TCTTCATCGTGTTCTACCACAATTAACGTATTACCCAAATCACGCATTTTTTTCAGGCTT

TCGATCAAACGGTCGTTGTCTCTTTGGTGCAATCCAATAGAAGGCTCATCCAAGATATAG

AGCACCCCTGATAAGTTAGAACCAATTTGTGTTGCTAACCTGATTCGTTGACTTTCCCCA

CCTGATAAAGTCCCAGCAGCACGTGATAAGGTCAGATAATTCAGTCCCACATTATTTAAG

AAGGTCAGGCGGTCATGAATTTCTTTGACAATCGGTTTAGCAATGGTACTTTCATTTTCT

GTGAGTTCCAGTTCTTCCAACAACTGCAAGTGGTCGGCAATCGATAATTCTGAAATTTGA

CCAATATGAGGTCCTTCTTCGCCACCCACATGCACACACAAGGCTTGGTCATTCAAACGG

TAACCATGACAAGTCGCACAAGTCAATTCATTCATGTAGCCACGCATCACATTGCGCGTA

TAATCACTATTAGTTTCGTGATAACGGCGATTAACATTAGTCACAACACCTTCAAAAGGA

ATGTCAATGTTACGCTCACCACCAAAATCATTCACATAGTGAAAATGAAATTCTCTATCA

CCTGAACCATAAAGCACTAAATCCCTTTCTTCCTCAGTCAAGGCCTCAAAAGGTGTATCC

ATATCCACCCCAAAACTTGCCATAGCTTGCTCAAGCATGGTTGGGTAATAATTGGAGGAA

ATAGGATTCCAAGGCGCCAAAGCTCCCTCTTTCAAAGACTTACTTGGGTCAGGAACAACC

AAGTCCAAATCCACTTCCAACTTAATGCCTAGCCCATCACAGGTCGGACAAGAGCCGAAC

GGAGCATTAAAAGAGAAGAGTCTTGGTTCTAATTCAGGAACCGTAAACCCACAGACAGGA

CAGGAATAATGCTCAGAAAAGAGCAGTTCATTGCCATCCATAGTGTCAATCATAAGGTAG

CCATCTCCCAGCCTTAAAGCTGCTTCAACCGAATCAAATAAACGGCTACGAATACCATCC

TTATTAACCAGACGATCAATAACCACCTCGATGTTGTGCATCTTACTTTTTGACAGCTCC

GGCACTTCTGTCACGTCAAAAATATCCCCATCAACCCGCACACGGACATAACCATCTTTT

TGAATTTTCTCAAAAATGGTTTTGTGTTGACCTTTTTTCCGGCGAACAATAGGCGCTAAA

ATCTGCATGCGTGTTCGTTCAGGGAGGGCTAAGACCTGCTCTACAATTTGTTCTGCAGAA

GATGCTGTAATGGCCCCATGCCCATTAATACAGTAAGGTGTGCCCACACGCGCATATAGT

AAACGTAAATAATCATTGATTTCAGTCACCGTACCAACCGTTGAGCGGGGGTTCTTACTG

GTAGTTTTTTGATCAATGGAAATAGCAGGGCTAAGACCGTCAATGGAATCCACATCTGGT

TTTTCCATATTACCCAAGAACTGTCTAGCATAAGCTGATAAACTTTCTACGTAACGACGT

TGCCCTTCCGCATAAATAGTATCAAAGGCTAGACTAGATTTTCCTGACCCTGATAACCCT

GTCACAACAACTAATTTGTCTCGTGGAATTTCCACGTCAATATTTTTTAAATTGTGGGCT

CTTGCCCCATGAATTATTATTTTATTTTGCATCTTGAATGTGCCTAAAGGCACCCTCCTC

ATCGAGCTTTCTTATATTATAACAAAAATTTCTGATAACCTATTCCCCAAACTTTGCGAA

TTTTGGTATAATAGATAAGTATTGAGAATTTTTCAGAAAATCCTTGAGAAGGCTTTATTA

TCAAGGTTTGATGTCAATCTCTACCAGTCAATCATCAAAAAGGAGGGCTTGTCATGAAAC

AAATGTTTCTTTCATCGGCAATTGAATTCAAAGAAATAGAAACATTCGAGCCAGGAGCTT

GGATTAAACTGGTTAACCCTTCCCAAGAAGAGTCTATGAAGATCGCCGATCAATTTAACA

TTGATATTTCTGACTTGCGCGCCCCTTTGGACGTGGAAGAAACCTCACGTATTGCCGTAG

AAGATGATTATACCCTAATTATCGTAGACGTCCCGATATACGAAGAACGTAACAATAAAA

GTTACTATATTACTATGCCTTTAGGCATTATTGTAACGGAAAATGCTGTTATCACAACTT

GTCTGCATGACATGACCCTTTTCGATCATTTTCATAACCGCCGGGTCAAAAATTTCTATA

CCTTCATGAAGACACGTTTTGTTTTTCAGATTCTATATCGCAACGCCGAGCTCTTTCTGA

CTGCCTTGAGGACCATTGATCGCCAAAGCGAACGTTTGGAAGCTCAGCTTGAAGCAGCTA

CTCGAAATGAAGAACTTATCGACATGATGGAACTTGAAAAATCCATTGTCTACTTAAAGG

CTTCCTTGAAATTCAACGAACGCATCGTCAAGAAATTATCCAGCTCAACAAGCTCTCTCA

AGAAATACATTGAGGACGAGGATTTGCTAGAAGATACCTTGATTGAAACCCAACAGGCCA

TTGAGATGGCTGGCATTTATGAAAATGTCTTGAATGCCATGACTGAAACCACTGCTTCTA

TTATCAACAACAACCAGAACACCATTATGAAAACACTGGCCTTGATGACCATGGCTTTAG

ATATACCAACTGTGATTTTCTCCGCCTATGGTATGAATTTCCAAAACAACTGGTTACCAC

TAAATGGCCTAGAACATGCTTTTTGGTATATTACCTTGATTGCTATGCTACTTAGTTCCT

TTGTTGTGATTTACTTTATTAGAAAAAAATGGTTCTAGAGTGGAGCAAGCTGCAGAATGC

GGTTAAGACCTGACAAAACTCTGTGTCAAACGGCTAGCTCCAAAGTTTCATTACAACTAG

CTAATACCATTGACTTACTTTAACTAACCCATATAAAGAAGGGATTCCCATGGAATTACA

AGAACTTACCAAAAAAAATCAAGAATTTATCCACACCGCTACGAACAAATTAATCCAAGA

CGGCAAGTCTGATGAGGATATTAAGCTGATTCTCGAAGAAGCCATTCCAGCTATTCTAGA

AAACCAGAAAAAAGGTGTCACCGCTCGAAATCTATTAGGCACTCCGACTGCTTGGGCAGC

TTCTTTTAGCCAAGACCCTAGTCAAAGAGCAGCAGAAACTGACAAAAACACCAATCCTTG

GTTGATGTGGCTAGATACCTCTTTGCTATTCATCGGAATTGTTGCCCTTTTAAATGGCAT

CATGACCTTCTTCAACACAAACGCTACAGTTACAGGATTGATTTCCTTACTGGCACTTGG

TTTTGGTGGAGGAGCATCCATGTACGCTACTTATTACTTTATTTACCGTCATCTCGGAAA

AGATAAGAGCCTTCGTCCAAGTTGGTTCAAAATCATCGCAGCCTTATCATTAGCTATGTT

GATTTGGATTGCCTTGTACTCTGCAACTGCCTTTTTACCAACCTCTCTTAATCCACAGTT

GCCTCCTCTTGTCTTGCTCATTATCGGTGGTGTTTCCCTTGCTTTACGCTATTACCTACA

ACGCAAATACAACATTCAAAACACTATGTCACCTGTTAACAAATAACAATCATTTCATCC

TTGCTGTCTACGCAAGGATTTTCGTTTACTCTCATCTTTATTTTAGACAAAATTCATCTA

CATGCATAGACTATTCTAATCAACTAAAAAACAGTCCGAAATGGACTGTTGGTTTATTAG

TCTTCGTTTACGTAAGGCATAAGAGCCATAACGCGAGCACGTTTGATTGCTGTTGTTACT

TTACGTTGGTTTTTAGCTGAAGTTCCTGTTACACGACGAGGAAGAATTTTCCCACGTTCT

GAAACGAAACGGCTAAGAAGCTCAGTATCTTTGTAATCAACATATTCAATTTTATTAGCT

GCGATGAAATCAACTTTTTTACGGCGTTTGAATCCGCCACGACGTTGTTGAGCCATGTAT

TTTCTCCTTTATAAATTATGATGTTAAGGGCTCTTAAAAAAGACTGAATTTTAGAAAACT

GGCAAGAAATCTTGATTTCCTAGGGAGTTGATCTAAATTCTAAGTTCTAAATTTTTAGCC

CGAACTCAAATAATTTCGTATTCGTTCAATCCATTCTGACTAGAATGGAAGATCGTCATC

TGAGATATCCATCGGGTTTGAGTTCCCAAATGGGCTATCATCTCTTCCAAAGTTAGGCGT

TTGTTGTGCAGGCGCTGAGTAACTGTTTGATGATGAAGTGTTATTGTTAAAACCACCATT

AAATGAGCCAGTTGAGCCACCTTCACGTGTAGCACGACTTTCCAACATTTGGAAATTATC

TGCAACAACTTCTGTTACATAGACACGTTGTCCTTGTTGGTTTTCGTAGTTACGTGTCTG

AATACGACCCGTAACTCCGATCAAGGCACCTTTTTTAGCCCAGTTCGCTAAATTTTCAGC

CGGTTGACGCCAGATCACACAGTTAATGAAATCTGCCTCGCGTTCACCATTTTGGCTTTT

AAAGGTACGGTTAACAGCAAGTGTGAAGGTAGCCACAGCTACCTGACTTGGTGTGTAACG

AAGTTCTGCATCCTTGGTCATACGACCAACTAGTACTACATTATTAATCATAAACTACCC

TCTTAGTTGGAATTAAGCGTCAAGTTTAACGATCATGTGACGAAGAATGTCACCATTGAT

TTTTGAAAGACGGTCAAACTCGTTAAGAGCTGCTGCGTCAGTCGCTTCAAGGTTAACGAT

GTGGTAAAGCCCTTCACGGAAATCGTTGATTTCGTATGCAAGACGACGTTTTTCCCAATC

TTTTGATTCAACAACAGTTGCACCGTTGTCAGTCAAGATAGAGTCAAAGCGTGCTACCAA

AGCGTTTTTAGCTTCTTCTTCAATGTTTGGACGAATGATATAAAGAATTTCGTATTTAGC

CATTGATATTTTCCTCCTTTTGGTCTAATGACTCACTACTTTTGGAGTGAGTAAGTGAGG

TATACTCACAGAATCTTATTGTACACTAATAGTCAATTAAAAGCAAGGAACTTTTTCTAC

TTGACTAAGTTTTGTCAAACATTTCCTTTCACGAGTTTTAAAAATTGGCACTTAGTCAGA

CTCCTAAAAAATAGGTGAAAAAAGAAAAAGCAAGGAAAAGTTTTAACCAAAGTGCAAAAT

ATAAAATTAGGGCCAAAACAAAAAGGTTAAACTTTTTAAAAATAATAGCAACCATGGCAC

ATACGATACCTAATCCAGGAAAAACTTGCGTCATGATCAGCCATTCCAACTGTAGTCCGC

TCAAAGAAGGATCAAATGGATAAATATAGTTAAGAAATAACACAAGAATCATCATTATAA

AAACGAACCACGAATACCGATCAATAAGATTCTTTAATTTCTGGAACATGTCCCCTCCAA

ATTAAAACCTGTCTGCTTCTATCGTTAATTTCAGTTATTCTAAATCAACATTTAGATGAG

GTAAACCTAATATTATTATAATATTTCCTTTTCTTTTTCGCAAATACCTAAAATCTTTTT

TTACTCTTTTTAAGACTCCTGTGTTACAATAACAACATGATTGAATTAAAAGATTATGGT

ATTAATATGTGGGATAATGAGACAATCGCTTCTTTTCGTCGCACACTTTTAGGGTGGTAT

GACCAAGAAAAACGTGATCTCCCTTGGCGAAGAACTACTAATCCATACTATATTTGGGTT

TCTGAAATCATGTTGCAACAAACACAAGTTAATACGGTAATCCCTTATTATAAACGTTTT

CTAGAGTGGTTTCCTCAAATAAAAGATTTGGCAGACGCTCCTGAAGAACAATTACTAAAG

GCATGGGAGGGACTAGGCTATTATTCTCGGGTGCGTAATATGCAAAAGGCTGCACAGCAA

GTGATGGTTGATTTTGGTGGCATATTTCCTCACACTTATGATGATATTGCTTCATTAAAA

GGGATTGGTCCTTATACTGCAGGTGCTATCGCCAGCATCTCATTCAACTTACCAGAGCCT

GCTGTTGATGGCAATGTGATGCGTGTCATGGCTCGTTTGTTTGAAGTTAATTATGATATC

GGTGACCCTAAAAATCGTAAAATTTTTCAAGCTATTATGGAAATACTAATTGACCCTGAC

AGACCCGGTGATTTTAACCAAGCACTTATGGATCTAGGTACAGACATCGAATCCGCTAAG

ACACCTAGACCTGATGAAAGCCCTATTCGTTTCTTTAACGCTGCTTATTTGAATGGTACT

TATGGTAAATACCCTATAAAAAATCCAAAGAAAAAGCCTAAACCAATGCGAATTCAGGCT

TTTGTCATTCGTAACCAAAATGGCCAATATCTACTGGAGAAAAATACTAAAGGGAGACTT

CTAGGAGGTTTTTGGTCATTTCCTATTATCGAAACGTCACCTCTTTCTCAACAATTAGAT

TTATTTGATGACAATCAATCCAACCCCATAATCTGGCAAACCCAAAATGAAACTTTCGAA

AGAGAATATCAATTAAAGCCCCAATGGACTGATAATCACTTTCCAAATATTAAGCACACT

TTTAGTCACCAGAAGTGGACAATAGAACTCATAGAGGGGGTGGTCAAAGCAACAGATTTA

CCTAATGCTCCTCACCTAAAGTGGGTAGCAATTGAGGACTTTTCTCTGTATCCCTTTGCA

ACACCACAAAAGAAAATGCTAGAGACCTACTTAAAACAGAAAAATGCTTAATCACAAGTG

AACTATGACGGTTCACTTTTTGTTTACAATTCCTTTACGCCGCTGTAAAGTTTTCCTCAG

ATTGTCTTTTAATACCTTATAAATAGTGATCTATCCCTAATGTCAAATACATTTGATAGA

CCCGTTAGCTTATCATTCTTATACTAATTTTATGAAAGGAGATATTTATGGAAATTGGTC

AGCAAATGATTCGCTACCGTAAACAACAAACGCTCTCGCAAGAAGAATTAGTCGAGAAAG

TCTATGTAAGCCGTCAGAGCATATCTAACTGGGAAAATGACAAAACTTACCCAGACATCC

ATAGCCTCTTACTCCTAAGTCAAATTTTTCAAGTGAGCTTAGACCAACTCATTAAAGGAG

ACATTGAGAAAATGAAATATACCATCACTCAAGTTGATAAAAAGAATTTTGAACGAGATA

CCAAAGTAATGGTAACATTGATGATACTATTAATGATTTCAAGTTATCCATTAGTCTATT

TCTTAGAGTGGCTAGGACTTGGCATTTTTGTTTTATTATCGATAATTACTATGACTTATG

CTAATCGTGTGGAACGTTTTAAGAAAAAATATGATGTGCAACCTTATAAGGAAATCCTAG

CTGTTTCAAATGGAAAATTATTAGACGAAATCGAAAAACGAGAAGAAAGAGCAAAACTTC

CTTATCAGAAACCCCTTATTGTCACTGTATTTTTCCTCATTACTGTTGTTATTGTTTTTG

CAAGTCGTTTTATGTTCACATGGTTATTCCATTGAAGTTAGTAGGTCATAAAATAAAGCT

CAGCGTGACGCTGGGCTTTATTTTAGTTAACTCAATTCAGCAATAATAGCTTTCAGTTGG

TCTTTAGTATGAACACCTGCAACTTGTTTAACCACTTCTCCGTCTTTTTTTAACATCAAT

GTTGGAATTGACATAATACCAAACTGACGAGCAGTTTCCGGATTTTCATCAACATCCATT

TTCAAAATTTTGAGTTCATCTTCATCGATTTCTTGAGATAACTGTTCTAAGATTGGTGCT

TGCATACGACATGGACCACACCAAGTTGCCCAAAAATCAATGAGTACTAAACCTTCTTTA

GTTTCTTCTACAAATGTAGCATCTGTTACTTCTAATGCCATCTTTTTTCTCCATTTCATC

TTAATGATAAGCCTATTGTACAAGAATTTAGTTGTTTTTGAAACTATTTGCTACGAACTC

GCTAAATCAATAAACAAAGCCCTAACATCAAGCCAATAAGGTAACCTACAATAACATCCT

TAGGATAGTGAATACCTGCTATCACACGGCAAATTGCCAATAAAACCGATAATATTAAAC

AAACTATTCCAAAGTAAACATAATATCGTAACAAACACATACTAATCATCGTAGCTGAAA

AAACATGTCTGCTAGGCATTGACCTTCCCTTTGTATCCTTATCAATTAAAGGTTTAATAT

TCCATTTTTCATACGGTCGTGGAAAATCAAAGCGTTTTCTAATGTAAGACAAAGCTATAA

AGCCAACAGCAGGAATAATTAATAGCCTTAACCATAATTGAAATGTCATCCCTTGCCAAA

GCGTAAATATCAGAAAAGATGGATAAAGTATGTACATCACTCCTGTGATTATTTTTAGTA

AAACATTTAAAAGAATTATTAACTGTGGGGTTTTTCTAAAAGGTTGAGATAGTTTGGCAT

AAAAGTGTTCATATGATTCCATCATTTACCCTAACGTTACAATTGTGGCACCAGATCCCC

CTGCATTTTGTGGGGCATAAGCAAAATGCTTAACATGCTTATTACGACGAAGATATTTTG

TCACTCCCTCACGAATAACGCCTGTACCAATACCATGAATGATATCAACTTGTCCCATAT

TGTTAAGCAATGCTTGATCAATAAAATGATCTAACTCTTGCATTGCTGCTTCGTATCTTT

TACCTCTAAGATCAAGTCGAGCTCTTGGTCCAGAACTATCAGCCTTTTTAACCACATTAA

TCTGTTTATTTTTGACTTTCTGTTCTTCTTGAACTCTAACGAGGCTAAATTCATCTTGTG

TTAATGTCATTTTGATAATTCCCACTTGTGCTTCCCAACGGCCATCTTTTAATTGACTAG

TTAAGGTACCTCGCTGTCCATAACTAGTCACTATAATATCATCACCAATTCTAGGGGCAC

GAGCTGCTTTGATTTTTTTAGCCTTATTTAAGACTTTATTTTTTGATAAATCAACTTGAG

GTGCTAATTTTTTTATTTGTGCCTTAGCATCTATAATTTCGTGAGGTTTTAATTGGCTCT

TATCATTGAGTTTTTTTAAGATAGTATCACTCTCATTCAAAGCCATATCTACAATTTCTT

GAGCTTCTTGATAGATTTTTTCTAACTCTTTATCGCGTTCATGTGAAAATTCATTATAGA

GTTTCTTAACCGCACGATTGAATTTGAGATTTTCTTGTTCAACTTCTTTAATATGATCCA

GTCTTCTACGTGTCTCAAGTGTCTGTGCCTCTAACTGTTCAATAATACGGTTAACATCTG

AGTCAGAATCTGTCATCTGCTTAGCTTGTTTAACAATAAATGGAGCTAAACCAAGGCGAG

AAGCAATTTCAAATGCATTTGAGCGTCCAGGAACTCCTTGCATAAAGCGATACGTAGGAC

TAAGCGTTTCTGCATCAAATTCCATGCTCGCATTCTCTACAAAATTTGTTTCAATCCCAT

AAGCTTTTAATTCTGGATAGTGCGTGGTCGCCATCGTTTTGATATGACTTAACCTAAGGT

GTTCTAAAATAGCCATAGCCAAACTAGCACCTTCTTGAGGATCCGTTCCTGCTCCTAGTT

CATCAAAGAGAACTAAACTATTGCGGTCAGCCTCGTTTAAAATACTGACTATGTGCGTCA

TATGACTAGAAAAAGTTGATAGACTTTGTTCAATAGATTGCTCATCGCCAATATCTGCAA

AGATATTGTTAAATACTGCAATTTTACTACCTTTATCCGCTAATACTGGCAAACCAGACT

GTCCCATTAATTGTGCTAAACCGAGTGTTTTTAGCATAATCGTCTTACCACCAGTATTGG

GACCAGTGATGACAATTGCAGTTAAATCGTGATCAAAATGTAAGTCATTAGCCACAGGAT

TACTTAAAAGAGGATGACGAACATTGATTAATGCTAACGTGCTGTCATCAGAGATCTTAG

GTATCGTTGCCTTATTATCAGACATAAAAAGATATTTAGCCCTTACAAAATCAAGATGCC

CAAGAATCCATGCATTATTTCTAATAGTGGCGACATGAGGTCTTAACAAGTCTGAAAATG

TGTGTAAAATACGACCTTCTTCGTGGCGTTCGTCAGCTCTAAGCTGCGTTATCTCTTCGT

TTAGTGTAACTACAGCACGAGGCTCAATATAAACAGTACTTCCTGAAGAAGAGATGTCAT

GAACCACACCAGAAATACGATTCCGATAAGTATTTTTTACTGGTAGGACACTTCGTCCAC

TACGACTAGCGATTAGATTCTCTGATAAAAGCTCTGCTTTTTCCTTAAGCATATCCTGTA

AAATCTGACGAACCCGTCGTTCACTATTTGTTAATTGACGACGGATACGCTCTAATTTTG

GACTCGCAAAATGTTCTAAAAAACCACCATCATTGATAGCTTGAAAAGACCCTTGCAGAT

TAGGGAATTGTTCCAAATTTTCAAACAAACGATCCAAAGATTGGAAAGAAACATTATCCA

AATCAGAATAAAAGTGAATCATATCCGAAGAACTCTGTAAAACTTTTTTGATAGCCAAAA

GTTCTTGAATATTAAGATCAGCTGAAAGCTCTAAGCGTTTTAAACTCTCAGAGATTGAAC

TTAGGCTGACTATGCCAAATGAGTGATGTTCAACAAAAATCTGTTCCATGTCAGAAATTT

CATTAAAACTTTTTTCTATTTTAGGAGCCTCCGTCATCGGCTCCAGCTCTAATAATTCTT

CTTGTGATTGTTCTGTCTTGAGATAAGGTAATAGCAATTCCTTAACTTTGTTAAATTCTA

ACTGCTCTAAAATCTTGTTATTCATACTCTTCATTATACCTAAAATGATGGAAAATGTGG

CAAGAAAGTCACTAAGTTCCCATGCGTTCTCTTTATTTCTAAACAGTTTCTCGTAAATAA

AGAACCTAAAGGTGAAACTCACTCCAGGTTCTTATATTAGTCACAAAACGATATCAAATA

ATGGCTTGAATCCACAATTTTTGAATAATTATTGTCAATGGTGGAAGGTGTTCAATCACC

AAACGAGCAAGAAAACTAGAGTGCAAATAGTGCTGTAAAAAAGGCATAGGGACCGTTGCA

AAAATAGACAGTAACATGTTCAAAAAAAGAAGCGATACTAGAAGCGCTAGTCCGCCACTC

AAACATTTTGTCCACTGGTTATCAAAATAATCTAGCAAAAGGAAATGAACAAAAATCCCA

AGAAAGCGACTCAGTGCATACCCCAATAAGAAAATGATAAAAAAGGCAAGGCCTGCATAA

TAAACCTTATCTAAAACAAAGATATCAACTGATTTAAAGAAAAAGACTGATGTTCCTTCT

ACCGGGTTAGAATAAGGAATCCAAAGCGTTAGCTTATGGGCTAATCCAATATAAAATCGA

TTAGCTACCAAAAGAGAAAGTAGGGCTCCTAAGACATAAAACGATTGAAGAATAATACCT

CTACTATAACCAATATAAAAATTCCAAGTTAAAACAAGCACTATCAATAAAGATAACATG

ATTAAACCTCATTCACATCTATTTGATTAGCTTTTTCTTGAAGACCAACCAAGGTTTTTT

GACGTAAATCTAAAATTTCAGCTTCCATTTTTTCAATGGCAATCTCTCTGCTTAATTGTG

TTGACAAGGTATTGATAGCCATTAAAATAGCGATTGTCTCGTCATCAGCTTCTGGTAAAT

GATTTTTAAGAGCCTGATACTTTTCTTTAGCTACCCGCTCAACTTCTTCCATAAAGAGAT

TGTCTTTGTCAGTTGTCAATGTTAATGTTTTTTCACCAAAGGTGAATTTGTAGCGATTAA

TACTTTTCATAGGGTCACCTCACTAGCATTATACCGTAAAATGAAACTTTCGTAAAATAG

TCAACACCATTGCCCTAACCTGTCTAAAAATTCGAGTCTCTGTTTTCTTTGTTAAATAAA

CTTATGAATTAACCATAGTCAACTATTTTATGTTAAAATAATTATTATGAATACCTTAGT

TTTGAAAATAGATGCTATACTGAGTAAACATTTAAAAAAACAGCTAGCCCCCTACACCAT

CAATAGCCAAAATACCTATGTAGCCTTTGCTGCTAAAAAAAATGGGGTTACTGTTTTGCT

GTACAAATCCGGAAAATTAGTTCTCCAGGGAAACGGTGCAAATGCTTTAGCTCAAGAGCT

TAATCTTCCAGTAGCTAAGACAGTTTTTGAAGCAAGCAACAATAGCCAAGATATACCTAT

AATCGGCAGTGATGAGGTGGGTAATGGCTCTTATTTTGGTGGGATTGCTGTTGTGGCAAG

TTTTGTCGATCCTAAAGATCATTCCTTCCTGAAAAAGCTTGGGGTTGATGATTCTAAAAA

ATTATCGGATAAAACCATTCAACAGATTGCGCCTCTGTTAGAGAAACAAATACCTCATCA

ATCACTTCTCCTATCACCTAAAAAATATAACGAATTGGTTGGTAAAAGTAAACCATATAA

TGCCATTTCTATCAAAGTTGCTCTTCATAACCAAGCTATTTTTTTGTTGTTACAAAAAGG

CATTCAACCTAAACAAATTGTTATTGATGCTTTCACTAGCCAATCAAATTATGAAAAGCA

TCTAAAAAAGGAAAAAAGCCATTTCCCAAATCCTCTAACCTTTCAAGAAAAGGCAGAAAG

TCACTATTTGGCTGTGGCTGTTAGCTCTATCATTGCTAGAAATCTATTTTTAGACAATCT

CGATCAATTAGGTCAAGATTTAGGTTACCAACTCCCAAGCGGTGCTGGTTCAGCTTCAGA

TAAAGTTGCTAGCCAACTCTTAGCCGCTTATGGTATGTCTAGCTTAGAATATAGTGCTAA

ACTTCACTTCGCCAACACTCACAAAGCGCAAGCGCTTCTCACTAAATAAAAGGAATCATA

TGAAACAGTTTATTAAAGAATGGGGCCCATTCACTCTCTTTTTAATTCTCTTTGGTCTAT

CTCGTCTTTTTTTGTGGCAGGCTGTTAAAGTAGACGGCCATTCTATGGACCCAACTCTAG

CTCATGGCGAACGCCTTATCGTTTTTAATCAAGCTAGGATTGATCGCTTTGATATTGTAG

TTGCTCAGGAAGAAGAAAACGGACAAAAGAAAGAAATCGTAAAAAGAGTTGTTGGATTGC

CAGGCGATACCATTTCTTATAATGATGACACACTTTATATTAATGGTAAAAAAACAGTTG

AGCCGTATTTGGCTGAGTATCTAAAACAATTTAAAAACGATAAACTCCAAAAAACTTACG

CCTATAATACCCTATTCCAACAGTTAGCAGAAACATCTGATGCTTTTACAACTAATTCTG

AGGGACAAACACGCTTTGAGATGAGTGTTCCAAAAGGAGAATACCTTCTTCTTGGTGATG

ATCGTATTGTTTCCAGGGATAGTCGCGAAGTTGGTAGTTTCAAAAAAGAAAACCTTATCG

GTGAAGTGAAAGCTCGTTTTTGGCCACTCAATAAAATGACCGTCTTTAATTAGAAAAAAC

CTGCCTTTGGGCAGGTTTTCGACTATCTTGTATAGACAGAAAGGTGCCTTATGGAACACT

TTTTTACTGGTACTGTTGACCGCATTATCTTTGAAAATGCAGCTAATTTTTTTAAAATTC

TCCTCCTTGCCATCGAAGACACAGATAGTGATATTGACGACTTTGAAATCATTATCACAG

GGACGATGGCTGATATTATTGAAGGAGATGACTACACCTTTTGGGGGGAATTGACCCAGC

ACCCTAAATATGGACAGCAACTCAAACTAAGCCGTTACCAAAAAATCAAACCTAGTTCAT

CTGGTTTGGTTAATTATTTCTCTAGCGACCATTTTAAGGGGATTGGTAAAAAAACAGCGG

AGAAAATCATTGCGCTATATGGTCATAATACCATTGACCATATTTTAGAAGACCCAAGCA

AATTAGAAACTATCTCTGGCCTATCTAAGGCTAATCGTCAAGCCTTTGTCGCTAAACTAA

AATTGAATTACGGCACAGAGCAACTGATTGCTGGTCTCGTCGAACTTGGCCTTAGCAACC

GTTTTGCCCTTCAAGCGTTTGAAAAGTATAAAGAAGAGGCTCTTGACCTTGTTAAAGAAA

ATCCCTATCAGTTAGTCGAAGATCTACAAGGTTTTGGGTTTAAAATGGCAGATGCTCTCG

CTGAAAACTTAGGGATTGAAAGCGACTCTCCGAAACGTTTTCGTGCAGCCCTCCTTCACT

GTCTCTTGGAAGAATCCATCAATCGAGGAGATACTTATGTCCAAGCACGACAATTATTAG

ACTTTGCCATCACACTCCTTGAAGATGCACGTCAAGTAGAATGTGATCCTGCTGCTGTGG

CTGAACAACTAAGCGAGTTGATCATAGAGGGCAAAATCAAAAACAGTGACACCAAACTGT

TTGATGCTAGCCTTTATTTTGCTGAAGAAGGGATTGCTAACAACATCTCTCGTCTCTTAG

ATACTCCTTTAAGTCAGTCATTTAGTCATGATACCATTCAAACAACCATCCAAGCCGTTC

AAAAAGACTTTGCCATCACCTATGACCAAGTGCAGCAAGAAGCCATTACTAAGGCCTTAA

CCAGCAAGGTCTTTCTCCTAACAGGTGGTCCCGGAACAGGAAAAACAACTGTTATTCGAG

GTATTTTGCAGGCTTACGCGAACCTGCATCAGATTGATTTGGATAAAAAAGACCTTCCTA

TCTTGTTAGCAGCTCCAACAGGTCGAGCTGCACGTCGCATGAATGAGTTGACTGGACTTC

CTAGCGCAACCATCCACAGGCACTTAGGCCTCAATGGCGACAACGATTACCAAGCCATGG

AGGATTATCTAGACTGCGACTTGCTGATTGTCGATGAATTTTCAATGGTGGATACCTGGC

TTGCCAACCAGTTGTTAGGAGCGATTAACTCAACAACTCAAGTGATTATTGTCGGAGATA

GTGACCAGCTTCCTTCGGTTGGACCCGGTCAAGTCCTGTCAGATCTTTTAAAAGTCAATA

GCCTGCCCCAAATCGCCTTGCAAAAGATATTTCGTCAATCCCAAGAATCTACTATTGTCA

ATTTGGCAGACCAGATGCGTCGAGGAATCTTAGCTGCTGACTTTCGTGATAAAAAAGCTG

ACCGTTCTTATTTTGAAGCCCAAGCAGCCTTTATCCCTGACATGATTCAAAAAATTGTCC

TATCTGCTATTAAAAGTGGCATCCCTGCTGAGGAAATTCAAATTTTAGCACCTATGTACA

AAGGACAAGCTGGCATCAATCATCTTAATCAACTCATGCAAGAACTTCTCAATCCCTTGC

AAGGACAAACAGAATTTCTGTTCAATGATACGCATTTTCGTAAAGGTGATAAAGTCCTGC

ACTTAGTCAACGATGCTCAGTTGAATGTCTTTAACGGGGATATTGGTTATATTACAGATT

TGATTCCTGCTAAATACACCGAATCTAAGCAAGACGAATTAATCTTAGATTTTGACGGTA

GCGAAGTCACGTACCCTAGAAATGAATGGCTAAAATTAACCCTGGCCTATGCCATGAGCA

TTCATAAATCGCAAGGGAGTGAGTTTCAAGTGGTGATTTTACCTATCACACGCCAAAGCG

GCCGACTCTTGCAACGAAATGTGATTTACACGGCCATTACTCGGTCTAAAAGTAAGTTAA

TTCTGTTGGGAGAATATACTGCCTTTGAGTATGCTATTAAACACGAAGGCGATAAACGCC

AAACCTACTTGATCGAACGCTTCCAAGAACAATCCGACTTAGCCTCCTCTCAACCTAACC

AAGAGCTAAAATCAAAAGAGCAGACCTCCCTTTTTTCTAATACGGCAACCCTTGAGGACG

ACTCTCAAAAATCTTCCTCTCAATCAACAAACTCTAACCCCACTGAGAACTCTCAGTCAG

ATAATGATGATTTTAGGTTAACACCTGAGAATTATTCGACTATCGATTCGATGATAGGGC

TTACAGAGTCAGATATTGCCCTCTTTTTCCAGAAAAAATCCTAGAAAAACGGACATTTTT

GATTAATCTATGGTATAATATTTTTGTATTAAACACATTATACAATTTCGGATTAAGGAC

TCTAGGAGGATTATATGATTTCTTATGAAAAAGTGCGTCAGGCACTCAAAACGTCTACTA

TTGCCATTATTATTCTAAACGGGCTTGGAGTTGTACTGTCATTAATGGGATTTGCAGGGA

TTTTCTACTTACAAAGCCAACTCAAGAATGAAGCATTTCGGGCCCAATTAACAACTGAGC

AATTGGCACAGCTACAAAGCAGCATGACACCATTTATGATTTTCTTATCTGTCTTAAATG

TCCTTGCTATTATTGCAATTATCATCTTCTGTACTCAGAACTTATCCAAACTAAAACAAG

GATTAACGGTTAGTTATATTCCTTACATATTAGGACTTATCCTTTCTGTTATCGGCTTGG

TTAACCAGTTCACAACAACCTTGTCAATGGTTGGAACTATCCTTATCCTTATACAAGCTG

CTCTTTATGGCTTTGCTTTTTACAAAGCAAAAAACCTTAACGAAAAAGGTGATGATACAG

ACCAAGCTATGCTGTAATAAAATAATGGACCAAAGAAGCTGATTAGGGAAATCCCTAATC

AGCTTCTTTTTTCTCCAAAATGAGGCGGACGGATAGAGCACTCTTTCTCCTATAGGTCCA

AGGAAATATCAGCTACCTTATCCTCCAAATTGGTCATGGTTACCCCTAACAGGCGGATAC

CAGCAGGATTTTCGCTTAAGCTGTCAAAAATATCCCCAGCTACTTGCTCAATTTGTGCGG

CATTTCTGGTTAATTCTGGCAAGGTGACACGTTTTGTCAAGGTGGTAAAATCAGCATAAC

GCACTTTGAGCACAATGGTCTTGCCTAACTTTTTATGGTCTTGTAAGAGAGCGGCCACGC

GCTTAGCATTTTTACTGATCTCTGCCTTGATGTCTGTTTCTTGATAAAGCAGTTTAGCGT

AGGTTCTCTCACTGCCAATTGACTTGCGTATCCGATCAGACTTGACAGGAGAATTGCTGA

TGCCTCTCGCTTTACGGTAAAGGTCAAAACCAAACCGACCAAAATGATCAATCAAGGTCA

TTTCAGGAACTGCCAACAAATCCTGTCCTGTATAAATCCCCATGTCATGCAGTTTTTCAA

CTGATTTTTTACCAACACCATGAAACTTTTCAATGGGGAGTTTGGCTAAAAAGCACAGGG

CATCTTCTTTCAAGACTAGGGTGAGGCCATGGGGTTTTTCAAAATCACTAGCCAATTTAG

CCAAAAATTTGTTATAAGACACACCTGCTGAACAGGTCAATCCTACTTCTTTCCAGATAT

CATGCTGAATCAGCTTGGCTATTTTGACGGCTGATTTTATACCCAACTTATTGTTGGTGA

CATCTAGATAGGCCTCATCAATGGACATGGGCTCTACCACATCAGTATAACGCTTAAAAA

TACGGCGGATCTGGTCTCCAACTGTTCGATACTTTTCATAATTTCCTGAAATAAAAATGG

CTTTGGGACAACGCTCATAAGCTTCCTTAGAGCTCATGGCCGAATGAATGCCATATTTTC

TCGCTTCGTAATTACAAGTGGAAACAACTCCGCGACCACCTGTTTCTCTTGGATCTTTCC

CAATCACAACAGGCTTTCCTTTTAAAGCAGGGTTATCCCTTTCCTCAACTGCAGCAAAAA

AGGCATCCATGTCAATATGGATGATTTTTCGTGACGTGTCATTAATCAGTGGAAAAATAA

GCATAAGTCCCTCCTTTCTCTATTATACCTTTTAGGCTATCTAAAGCCAAAAAAATACAT

TCTAATACAGTTTGTGACTGTTTCAACAATCTGTATTATAAAAGAATCTTAATTTTGTCA

AGAATTTTGCTTGGGAAAACGTTTCCTATGTGTTAAACTTAGAATTGTAAATGTAACAGA

TCGCTTTGTTACTAGAATTTAAGGAGAAATTTATGGCAACTGTTAAAACCAATACAGATG

TTTTCGAAAAAGCTTGGGAAGGCTTTAAAGGAACTGACTGGAAAGAAAAAGCAAGTGTCT

CACGTTTTGTACAAGCTAACTACACCCCATACGATGGCGATGAATCTTTCTTGGCTGGTG

CTACTGAACGCTCACTTCACATCAAAAAAGTTATCGAAGAAACAAAAGCACACTATGAAG

CTACACGTTTCCCATACGATACTCGTCCAACTTCAATTGCTGACATTCCTGCTGGTTTCA

TCGATAAAGAAAATGAATTAATTTATGGTATTCAAAACGATGAATTGTTCAAATTGAACT

TCATGCCAAAAGGTGGTATCCGTATGGCGGAAACGACCCTTAAAGAAAACGGCTATGAAC

CAGACCCAGCAGTACATGAAATCTTTACTAAGTATGTGACAACTGTTAACGACGGTATCT

TCCGCGCTTATACTTCAAATATTCGTCGCGCTCGTCATGCCCACACTGTAACTGGTCTTC

CAGATGCTTACTCACGTGGACGTATCATCGGTGTTTACGCTCGTCTTGCTCTTTACGGTG

CTGACTACTTGATGCAAGAAAAAGTCAATGACTGGAATGCTATCACTGAAATCGATGAAG

AATCCATTCGTCTTCGCGAAGAAGTTAACCTTCAATACCAAGCTCTTGGTGAAGTGGTTA

AACTTGGTGACCTTTACGGAGTTGATGTTCGTCGCCCAGCACAAAACGTTAAAGAAGCTA

TCCAATGGGTAAACATCGCTTTCATGGCTGTATGTCGTGTGATCAACGGTGCTGCTACTT

CTCTTGGACGTGTGCCAATCGTTCTTGATATCTTTGCAGAACGTGACCTTGCTCGCGGTA

CCTTTACAGAATCAGAAATCCAAGAATTTGTTGATGATTTTGTCCTTAAACTCCGTACCG

TAAAATTTGGTCGTACTAAAGCTTACGATGCCCTTTACTCAGGTGACCCAACATTCATCA

CAACTTCTATGGCTGGTATGGGTAACGACGGTCGTCACCGTGTGACTAAGATGGACTACC

GTTTCTTAAACACTCTTGATAATATCGGTAACTCTCCAGAACCAAACTTAACAGTTCTTT

GGACTGACCAACTGCCAGAAACATTCCGTCGCTACTGTATGAAAATGAGCCACAAACACT

CTTCTATCCAATATGAAGGTGTGACAACAATGGCTAAAGAAGGTTATGGTGAGATGTCAT

GTATTTCATGTTGTGTATCACCACTTGACCCAGAAAATGAAGAACAACGTCATAACATCC

AATACTTCGGTGCTCGCGTAAACGTTCTTAAAGCCCTTCTTACTGGTCTTAACGGTGGTT

ACGATGATGTTCACAGAGACTACAAAGTGTTTAACGTTGTTGAACCAATCACATCAGAAG

TTCTTGAGTACGACGAAGTTATGGCTAACTTTGAGAAATCTCTTGACTGGTTGACAGATA

CTTATGTAGATGCGCTTAACATCATCCACTACATGACTGATAAATACAACTACGAAGCAG

TTCAAATGGCCTTCTTGCCAACACACCAACGTGCTAACATGGGATTCGGTATCTGTGGTT

TCGCTAACACAGTTGATACCTTGTCAGCTATTAAATACGCAACTGTTAAAACTATCCGCG

ATGAAAATGGCTACATCTATGACTACGAAGTAACAGGTGACTTCCCTCGTTACGGTGAAG

ATGACGACCGTGTTGATGACATCGCGAAATGGTTGATGGAAGCTTACCACACACGTCTTG

CAAGCCACAAACTTTACAAGAATGCTGAGGCTTCAGTATCACTTCTTACCATCACTTCAA

ACGTTGCTTACTCTAAACAAACTGGTAACTCTCCAGTCCACCGCGGCGTCTTCTTGAACG

AAGATGGTACAGTTAACACTAGCCAAGTGGAATTCTTCTCACCAGGTGCTAACCCATCTA

ACAAAGCAAAAGGTGGCTGGTTACAAAACCTTAACTCACTTGCTAAACTGGAATTCTCAC

ATGCTAACGATGGTATCTCATTGACTACTCAAGTATCACCTCGTGCTCTTGGTAAAACAT

TTGACGAACAAGTTGACAACTTGGTAACAGTGCTTGACGGTTACTTTGAAAATGGTGGTC

AGCACGTTAACTTGAACGTTATGGACCTTAACGATGTTTATGACAAAATCATGAACGGCG

AAGACGTTATCGTACGTATCTCTGGTTACTGTGTCAACACCAAATACCTTACACCAGAAC

AAAAAACTGAGTTGACACAACGTGTCTTCCACGAAGTGCTTTCCATGGACGATGCTGCTG

AAGCTATTTCAAGTAAATAGTAGTATCTAAATGATCAATAACTTATAAAAAAGAACAGCC

ATCTCATTTGATTCAAAATGAGATGGCTATTCTTTTTTCACTTATAATCACTCTAATCGC

ATCAGACAAATGAAATGGCTATTTTTTAGAAGAAACTTTAAAAACAGTAATAAGATCACT

AAAACTGCCCCTTTAGACGTTACGATACGGGGGTTGTCAGATCATGCTTTAGTGCATTGT

AAAGCAACTGACGTTCTTTTAACCATTTACTTTTATCATCCTCGTCGTAAGTACGGTTGG

TCAAAAAAATAGCTGCCGTTTGCTCTTTTTTATTAAGCATTAGAAATGGCCCTGTGTAAC

CTGTATGACTAATCCAATCGCCATCTAAATTCCACCCTAGTGACCGCTCAATTGTTTGTT

GGCTGTAGTTGCGCCACAGACAATCTGAAAATGGATCATTAAGATAATGATTGCTAAAAC

TTTCTAAATCTGCAAGAGTTGAAAATAGCCCCGCAGATCCAGAATGTTTTTTTAAGATTT

TTGCTTTGGGATCATGAACTTCTCCATCACTCACACCCTTTAATGTTGGTACAGCTTCTG

GACGAGGGCCAAAAGACGTATGATACATCCCAAAAGGAGTAAAGATAGTCTTATCAAATA

TCTGGTCTAAAGATTCACTAAAGAGCTCTTCTAACATAAAGCCTAACAAGAGGAAATTGA

CATCTGTGTAATAAAAGTTTTTATTTTCTTTTTGAGTAAGATGATTAAGTGCTTTTCTTA

ATTGTTGTGCATTTAAAACATCTCTGTTAGGAATATAAGGATCAAGACCACTGGTATGAG

TCAATAACTGTCGAATAGTGACAGTCGCATCAGCAATACTAGGATAATATGCTTTTAAGG

GATCATCTAATGCTAACGTACCATTATTCAATAAAATATTGCATATTGTGGCCACACCAA

CAACTTTTGAAACACTAGCCAAGTCATAAACTAAGTTAGCATCAACTGGTCGTCTTCCAT

CAATTGTTCCGATATGGTATTCTTGCCATCGACCAGATTGAAAGAGAGCCAGACTGGCCC

CTTTGTATACTTTTTTGTGAAGATGGTTTTCAATACATTTAATGACTGCCAAAGTCATTT

CGTAAACCATACTTCGATCTGACTTGGATCAGAAAGGACCAATACTTTGTGTTTTTTATC

AATGTAGACCTTGTGCCCGTCTTCTTCTAAAGTTGCTTTTAGGACCTTCATATCATAGTC

CTTTGAGACTTGAAATTCTAAAATTTCTAAATCCCAAGCAATATGGGGATCAATTGCCAA

ATCGGGACCTTCTTCTTGAATAAAATCCATAGTAATAGGTAACTGATCACTAAACAGATC

CCGATAAAAAGCTTTTGAGCGTTCTTCACTAATAACATTTAAAACAATAATATCAAACTT

AAATTGAGTTAAACCTTTAAAAGTTGCATCTTTTTCAAGCGATGGTAAATCAGTACCTTG

GAGATGTTTAATATCTTGTTCAGCATGCAACAAGAATCGGTCTCCTTCTGGGGAAATAGT

CTCAAAGGCATAGCCATTTTGACCTTTAAAAAGAGTATCATAATATGCTCCATGAGCTAA

TAATTGTTCGATTTCTTTAGGTTGATTTGTTTTGATGACAATCGTATTAACCTTTTTAGG

GCCTTCTACCGCACGCGTTCTAACTGAAGGTGACTCTTCAATTACAAAACATTCCTGCCC

TTCTCCCCAAGATGAAAAGATAGCAATCGCATTTTCTTCAGATACTAAACGAAGCCCTAA

ATTATTTTGATAAAATGCAATATTAAGGTCGCGATCATTAACGCGTAATACTGGAGTTTT

AAAGGTAATATTTTCCATTAAAGTCATAATTTCCTCCTAATCCTCTGCTATTATAGACAA

AATTCCTCTATTTGACAAGAAATTATACGTATTCATCCTACTAGATTTTTGATAAAGATC

GACAAACTAGCCTTTTAAACATTCACTACAATAACAGCTAAGCTCAGTAAGGGAAAAAGG

AAGGCGTCCCCTTTCATGCAATATTAATATTTTAGCTTAATTTTAACAAAATACATCGAA

CCAAAGCTCTTCCAAGCATAGTGGCATGTTTTAATGAAGTAAATCACGTTGACGGCTAAG

GTTTTTTTTTGTAAACTAGTCTTTATGAACGAAATGCTTATCCTTAGACTGATACAAGCT

TTGCTAGTTTCGGCAATGCTTTTTATTTTCTTTATGTTAGTAAAACATCTTAAAAAAAAT

AAGATCAATCCTTTTAAACGCTTTTGGACGGGGTTTTGGATTGGCCTGCTAACAGATGCC

CTTGACACCCTTGGGATAGGGTCTTTTGCTACTACCACAACGTGCTTTAAGTTAACTAAA

TTAGTCACTGACGATAGACAATTGCCTGGCACTATGACCGTAGCTCATGTGCTTCCGGTG

CTCATTCAATCTCTTTGCTTTATTTTTGTCGTAAAAGTTGAGGTTCTAACTTTACTTGCT

ATGGCTGCAGCAGCTTTTATTGGAGCTTATTTGGGGACTCATATTACAAAAAACTGGCAT

GCTCCAACTGTCCAACGAATCCTTGGTAGTCTTTTAATCATAGCAGCTATAATCATGATC

ATTAGAATAATCTACCATCCAGGAGAACACCTTTCAGACACCATTCATGGCCTCCATGGC

ATCTGGCTTTTTGTTGGAATTGGTTTTAATTTTATCGTTGGAGTCTTGATGACTATGGGG

TTAGGCAATTATGCTCCAGAATTGATTTTCTTTTCATTAATGGGTCTTAGCCCAACAGTT

GCTATGCCTGTTATGATGCTAGATGCAGCTATGATTATGACAGCTTCTAGTAGCCAGTTT

ATCAAAGCAAACCGTGTCAGTTGGGATGGTTTTGCTGGAATTGTTTCTGGTGGTATTATT

GGCGTTTTACTAGCTGTGTTTTTCTTAACGAATTTAGATATTAATAGCTTGAAACTTCTG

GTCATCGCTATCGTTTTCTTCACTGGTGGAATGCTGATTCGTTCCTCCTTTACCTCTCAT

AAGCAACAAACAAATAGATAAGATAACGTTTCTGGGAATCACTAACCAGAGACGTTTTTA

ATATTTTACAACAAAAATGAAAAATCGTTTTTTACTCTATCAGTGATTTAGTCCGTGCAA

AATAGTTTCAACACCAGATAAAGGAAGTTAGGGATGCCTCGTTTATTTGTAACACCGTTT

AGAGGCATTAGTCATCTTATCTGAGGAGAGGCTTTTTTACAAGAAGTCTAGTAAAATTTA

CAAAATACGCTATACAAGACAGTCAAGTTTGCATTAGATTTCGCTTAACTCATATAAAAA

AGACAAGTTCCTTAAGCGAGCTTGTCTTGAGTCTAAAAGAAGCTTTAGTTCTTTTTTTAT

CTGATATAAAGGTATTTAAACGCTGCTACGGCTACTATCGCCGCTAAGATAGGTGCAACT

ACAGGAACCCATGCATACCACCATTTTGAATCACCTTTGGCTTGACCAAGAACTGATTTT

GGTAAGAAGTGGTGTAACAAACGAGGCCCAAAATCACGCGCTGGGTTTAGTGCTGGCCCA

GTTGGTCCACCTAATGAAGTAACCAAGACCATAACTAAGAAACCAATACCAATGTGAGCA

ACCGCCAATGAGCCTGTCACGTATGGTGAAATTTGTGTTGCTGCAGTTGTTTGGTCGTAA

CCTGCTTCAATCAATTTACCAACTAACTCTACCCCAAAGTAGTTTTTAGTCAAAGCCAAA

GCACCAAAGAAAAGAACAAATGAACCTACAAATTCGTTTAAGAAACCATTGATATAAGAG

ACTTTATGGCTGTCTTTTTGACCATTATCAAGAGATGAAATCGTTGAGAATGAGCCTAAA

ACATGATTTGGATTTTCAGTTTTCATAAAGTAAGGTTTATAAACCATTACCACTACCAAC

TGACCAAAGATAGCTCCAAGTAATTGTGCCACAACGTATTGTAAGACGTGAGCCCATGGG

AACAAGCCTGATACTGCTAGTCCAACTGTGAAAGCAGGATTAATATGGTTTCCAGATACA

TTACCAAACATCAAAGCTGGCATCATAACTCCTAAGCCATAGCCAAAAGCAATCACTAAC

CAACCTGAGTTATGTCCTTTTGTTCCTTTTAAGTCGACGTTAGCAACAGCTCCATTCCCC

AAAATAATCAAAAAAGCCGTTGCGATAAATTCTGTGATGTATTTCACAGTCCATGTCATT

TCCATGTTTTCTGGTTTCCTCCAAATAATAAAATATAACAAATACCATTTTAACAGGCTT

ATTGATGAATGTAAAGGTAATAAAATAATAAATAAAATGATCATTAAGTGTTATTTTCAC

ACAATAATAAAAGCGGTAAAGAAAAGGACCATAAAAGACCTTATCTTTATCACTTTATTT

CATAGTATTATAACATTTTTTATTAGCTCTCACTATCAACAGATCAATCTTAACATGACA

AATCCTATATTCTGATGTTAATCCAATTGCACCTACTAAAAATAAGGCCACATTTTCTCT

TTCACGAATGGCTTTTGCGAGTCATTTTTATTGACATGTACCAATAGCAAACTAGGATTA

TTTCAAAGCGCTACAGTAATGGCTTTACCCACAGCTAATCCCGGCTCATAGGAACTATCT

ACAGCTGTTAAGATACGTTTGTATTTAAGTATAAGACTAAACTCCTTTTCTTGTCCTTAA

GACACAACCTGTCTTGTCTTTGTTTTTAACAAGTAAAGCAAGGTACAAATCAGTGGAATC

ACAGCAGCTAACCAGAATAAGTGCCTAAAGGAAGCCACACCACTACCAAGTGCCAAATCT

TTAATCAAGCCCAAGAGGTAAGGCCCAGCACCTAAACCTAAATCAAGACCAATCATATAA

GTAGACATAGCCGTATTAAAGCGATGCTCATCCACTCCTTGTATGGAGGCGGCTTGACCA

CAAGACATAAAAGTGCCATAACCAAAGCCAATCAAGGCTCCTGAAAGCAAATAGCTTCCT

CCAGACGATACACTACCTAGCAAGAAGAGTCCCATTGCTAAAAAAAGATAACTTGGGTAC

AAGACCCATTTATCGCCTTTAGCATCCATTAAACGTCCCATTGCTGGACGAGTGATGGTA

ATGATTAAGGCATAGACCACAAAGAAATAAGCACCTACTGCTGTCAAATGAATTTCAGAC

GTGTATAATTTTTGAAATCCTAAAACTGAAGCATAGGCAATCCCCATTAAAAAGGCGATT

GCAGTGATAAACAAGGCTTTTTTCTCAATGAAACTATCCACAGTCCACGATTTGGTTTTA

GCCAATTGCTCCGCATTTAAAGACATGTTTTTGACTGGAAAAGCAAAAGCACCAACAACC

ACACACCCAATCAAGACGCTACATAATACAATAATCATGCGAAAATCAATATGAAGATTA

TCCAACATAAAGGTTCCTACAAAAGGACCAATAGCTGCTGCTAGACTGGTTGACAAGCCA

TAAAAATTAATTCCTTCTCCTCTTTTACAAGCTGGAATATAGGCTGTTACAATGGTATTT

GTCGCAGTTGATACCACACCATAACCAAATCCGTTTAAGAAACGAACCAAGTACATCATG

CTAATCGTTGGCATATAAAAGTAGGCTAAGGTCGTTAAGAGGTAAAAAATAGCACCTCCT

CTTAAAACCAAACGACGACCAAACACCTCCAACTGCTTTCCAAAAATCAAACGGGCTAAT

AAAGTCCCTAGAATATAAATCCCTGTTGCCAATCCTGCCTGGCTAGTCTGAGCTCCTAGC

TCTCTAGTCGCCACAAAGGCAATAATAACCGTAAAAAGATAGTAGACCATATAGACTATA

AAGTTTATTACTGTGATGGCCACAAAATGTTTGTTAAACAATTTTTCTTCCATTGAAAAT

GAACTCCTCTCTATTTTCATAAACATTTTACAAAAAAAGCAGAATATAATATATTTCAAT

GGTATAATATAAGTGTTACTATCAAAGGGCATTAGCAAATGTTAATTTCTCTTGAAGATA

CAAATTATACATTGGTAAAAGAATATTTGGAGAAAAGCGTGAATCATCACATTTTGCAAC

GGTATATTGATAATCATAACTTTCCTATCATTGAAAAAAGTTATCACAAGTACTTGACTT

TTGAAAGTCTCGAAGAGGACTTCACTTATATTTTAAAAGACGGCATTGTGAAACAGAGTG

TTCTTTCAAAATATGGAATGGAATTTAATTTGAGATATGTTACAGGACTTGAAATTACTT

CTGTTCTCAACACTGGTTATTCAAAAGATATGGGAGAGCCTTATAATGTTCGCATCGAGT

CAGAGAAAGCTAGTTTTTACAAGGTTCGCCGCTCAGCATTTTTAAAAGATATAAACGAAG

ATATTGAACTACAAGGATATGTGAAGGACTTTTACCATAATCGTCTTCAAAAATCCATGA

AAAAAATGCAATGTATGTTAACTAACGGCCGTATCGGTGCCATTTCTACTCAAATATATG

ACTTAATGACCCTATTTGGCGAAGAACTACCAAATGGTCAAATTTTAATTAATTTTGTCA

TTACTAATGAGGAACTCGGTAAATTCTGTGGCATTTCTACCGCTAGCAGCGTCAGCCGTA

TTCTAAAACAGTTGAAAGAAAAAAACATCATTCGCATTGATAAACAACATATTATCATTA

CCAATCTTGATAAATTAAAAGATAATATCGTTTTCTAAGCCACTAGAAAGGAAACTCCTA

TGCGCTATAATCAATTTTCCTATATTCCAACTAGTCTAGAAAGAGCGGCTGAAGAATTAA

AAGAATTGGGCTTTGATCTGGATCTGCAAAAAACTGCCAAAGCTAACCTAGAAAGCTTTT

TACGTAAACTCTTCTTCCACTACCCTGATAGTGATTATCCTTTAAGTCATCTAATCGCCA

AAAATGACATGGACGCATTGAGCTTTTTTCAATCAGAGCAAGAGTTATCCAAAGAGGTTT

TTGATTTACTGGCCCTGCAAGTCCTTGGCTTTATTCCTGGAGTTGATTTTACAGAGGCCG

ATGCTTTCCTTGATAAACTGGCCTTTCCTATCCACTTTGACGAAACAGAAATTATCAAGC

ATATCCACCACTTATTAGCGACCCGTTGTAAGTCTGGCATGACCCTGATTGATGATTTGG

TCAGCCAAGGAATGCTTACTATGGACAATGATTACCACTTCTTCAACGGTAAATCATTAG

CTACCTTTGACACGTCACAGCTGATTCGAGAAGTTGTTTACGTTGAAGCACCTCTTGATA

CCGATCAAGATGGCCAACTTGATCTTATTAAGGTTAATATCATTCGTCCTCAATCTCAAA

AACCATTACCAACTTTGATGACACCTAGTCCATACCATCAAGGTATCAATGAAGTAGCCA

ACGACAAAAAACTTTATCGTATGGAAAAAGAGTTAGTCGTTAAAAAAAGGAGACAAATCA

CTGTCGAAGACAGGGATTTTATTCCTCTTGAGACACAGCCTTGCAAACTTCCTATAGGTC

AAAATCTAGAAAGCTTTAGCTACATTAACTCCTATAGCCTCAACGATTATTTCCTCGCAC

GTGGGTTTGCTAATATCTATGTTTCAGGTGTGGGAACTGCTGGTTCCACTGGCTTTATGA

CGAGTGGAGACTATGCTCAAATTGAAAGCTTTAAAGCCGTTATTGATTGGCTAAATGGAA

GAGCCACAGCCTACACCAGCCACTCAAAAACCCACCAAGTGAGAGCTGATTGGGCAAATG

GTCTAGTTTGTACGACTGGAAAGTCCTACTTGGGAACTATGTCTACTGGTTTAGCCACTA

CAGGCGTAGACGGATTAGCCATGATTATTGCAGAATCAGCTATTTCTTCTTGGTATAACT

ACTACCGCGAAAATGGCTTAGTTTGTAGTCCTGGTGGCTATCCTGGAGAGGATCTAGATG

TCCTAACTGAGCTCACCTACTCGCGTAATTTATTGGCTGGCGATTATCTTCGCCATAATG

ATCGTTATCAAGAATTGCTAAACCAACAGTCTCAGGCGTTAGACCGACAATCTGGTGATT

ATAATCAATTTTGGCACGACCGCAATTACTTAAAAAATGCTCATCAAATAAAGTGTGATG

TCGTTTATACTCATGGCTTACAAGACTGGAATGTCAAACCAAGACAGGTCTATGAGATTT

TCAATGCGCTTCCTTCCACAATCAACAAGCACCTCTTTTTACATCAAGGTGAGCATGTTT

ACATGCATAATCGGCAATCCATTGATTTTCGCGAAAGCATGAATGCCCTTTTATGTCAAA

AACTCTTAGGCCTAGCTAATGATTTTAGCCTTCCTGAAATGATCTGGCAGGATAACACCT

GCCCACAAAACTGGCAGGAGCGTAAAGTCTTTGGAACCTCAACCATCAAAGAGCTTGACC

TCGGTCAGGAACTGCTTTTAATTGATAATCACTATGGTGAAGATGAATTTAAAGCTTACG

GTAAAGATTTCCGTGCGTTCAAAGCAGCTCTCTTTGAAGGCAAGGCCAATCAAGCCCTGG

TTGATATTTTACTGGAAGAGGACTTACCTATTAACGGTGAAATTGTCCTACAGTTAAAAG

TGAAATCAAGTGAAAATAAAGGACTTCTATCTGCACAAATCCTTGACTATGGTAAGAAAA

AACGCTTGGGCGATCTTCCTATTGCTCTCACCCAATCAAGTATTGACAATGGGCAAAATT

TCTCTAGAGAATCCCTTAAAGAGCTACCATTTAGAGAAGATTCTTACCGTGTTATTTCGA

AAGGTTTCATGAATTTACAAAATCGCAATAATTTGTCGTCAATTGAAACTATTCCTAATA

ATAAATGGATGACTGTTAGACTTCCTTTGCAACCAACCATCTATCATCTAGAAAAAGGAG

ATACCCTTCGAGTCATCTTATACACAACCGATTTTGAACATACTGTCCGTGACAACAGCA

ACTATGCTTTAACTATCGATCTCAGTCAGTCTCAATTGATTGTGCCAATAGCATCAAATT

AACAAAGAAAAGTGCTTAGCAATATAACTAAGCACTTTTTTGTTAATTGGTCTTACTAAT

AGCAAACAGAAAACGATCGTCTTAGTTCATAAATTCCGAATCTATTCCTAAAAAAATATC

ATCAACGGAAACCGCTAATGCTTGTGCAATACCTTTTAGCTTCTCATATTTTGCTCGCCT

TAGCTTTTTAGTATCTTTTTCATACCTAGCAATCGTTCTGACAGAAATACCTGTTAAGTG

AGACAATTCCTGCTGTGTCATTTTCTTTGTTGTTCTTAACTCTTTTAAAGTTGATTTGGT

CACAATTTTCCCTCCACACTAAAATGAGATTAGTTAATCATGTTAAGTTTATTATAAACT

TCGGTTTTTATGAAGTCAAGTTTTTAGAGAGTTTTTAGACCATCTTTTACGATACCTTTT

GCTTTAACCTCTTTTATGGTATCATATTTTATATAAAGAAAAGGAGAAAAATATGTCCGC

CAAGAAAACTTTTTTTGCAAGTAATTTAAAGTACCTTAGATTAAAAAAGAACATGGAACA

ATTAGAACTTGCTAATCTCCTAGGACGAAAAAGCTCTTCTTCAATCAGTGAGTGGGAAAA

AGGAAAATATACTCCAAAGTCTGGTCTATTAAGTGATATCGCTGCTATCTTTGATGTTAG

TTTAACCGCCCTAATGGAAGAAGATTTAACCTTAGAAAGTAAGGGAGTCTCTTATCAGTT

GCCACAACGAGAGTTAGCGATGATTTCAGAACAATTATCCGAAAATAACTACACTAAGTG

GGTTGACTTCGCACAAATTCTTTTAGAACAACAAGCTAAAGAAGATCGTGAATTAGATAA

CCACTAAGTCGGACTAGTCTATGACAAAAATAGTCCATTTCTAGACTTATTCTGTCATTA

GCCAGACCCTTCCTACTAATCTATCTTATCCATAGCGACTTTCTTACTGATACAGGCTAG

TCTACTAACTGACATTGTTTTTATTGCCTCTTTTTATGTGTTATAATAGTTAGTATTAAA

TTAAGGAGAGTATAGTCTTATGATGAATATGCAAAACATGATGAAGCAGGCACAAAAGCT

TCAGAAACAAATGGAACAAAAACAAGCAGATTTGGCTGCTATGCAATTTACTGGAAAATC

AGCTCAAGACCTTGTCACTGCCACTTTCACGGGTGATAAAAAATTGGTTGGGATTGACTT

CAAAGAAGCTGTTGTAGATCCTGAAGATGTAGAAACGCTTCAAGACATGACCACTCAAGC

CATTAATGATGCCCTTACGCAAATTGATGAAACAACTAAGAAAACATTAGGCGCTTTTGC

TGGTAAATTACCATTCTAAAAAAACAAAAACTAGCTTGAGAAGGATCTCAGGCTAGTTTT

TTTATGAGTTAGTGCTTATCCTCTTGAGACAGTGTCCTCTTTTGCCAGTTCCAAGCAATA

CGATTTTTGATGGCAATGGGAGCAGGTTAACTTTCTGGTTCGTGGTGTATGGCCTGCAAT

GGAAAATCCCAAATCGCGCAGAAAGCAAATCATGCGTAATTGTTCTAAATCAGAATCCGT

ATAGAGTCGCCGTCCACCTGCTGTCAATGCTGTCGGAATCAAAATCCCACGTTGGTCATA

GTATTGGACAGTTCGAATAGAAACGCCAGCTAAGTTGGCCAATTCTCCAGTCGAATAGAA

TTGAGACATTTCCACACCTTCTTTCTACCATTTAGTCATCAAAAGCGACTCGTGTGCACC

CGACTCCCTCTTGATATGCCCCTAGCTTATCACATGACCTAGCGTTACAAGCAAGGGCTA

ACCCTAGAGGATTTTTTTGTTTTTTAAAAATAGCAGAGCCTTTAGTCGAAAAAGTCTCCC

AAAGCCGCGAAAGGGTTATCTGTGGTTACCTCTTCTTGCTGGCTGAGATATGCTTTGTTT

GTATCTGTTTCTAAAAAAGACTTATAGATTGCTGCTGTCATTCTGGCATCTTCTAAGCTA

TTATGACCTCGACCCTTAATGCCTAAAAAGGTTGCGACTGTTTGCAGACGAAGGTTAGCA

ATCCCATTTAAGTCTGCGCTACGTCTATCGTAAGCTTCATCAAAGAGGTCAATCTGGTAT

TGGTCCCTTAAATCTAAGCCATTTTCAGCTAGAATAGGCAGGTCAGATTTTTGAGCATTA

TAACCAATCAATGGTAACTCTCCCACAAAATTTTTAAACGCAGCCATAACCTCCTCTACT

TTTGGTGCCGCAGCAATCTTGTCAGATGTGATTCCTGTTAGGCCATTGATAAAGCTCTGT

AAGGGAGCATCTGTGTAAACATAGGTGTCAAAGCTGTCAACTTCTTTATGGTGGTCATAT

TTGACTGCTGATACTTGAATGACATGACTAACGTCATTGACAGTGTTAAATTCTAAATCA

AAGGCAATATAAGTATCTAAGTGTTGCATGTTTTCCTCCAAAAAAGACCCTGATAGGGCC

TTCGTCATTTTTTGTGATTATTTCGCAAACAAGCGGGTGAAAAAGCCTTTTTTAGGGCCA

ACTTCAGCCTGAACTTCTTCGACTTGTAAGCGAGCTTGGTTGGCTTCTGCTTTAGCTTCT

TCCAATTCTAGTTTTAGGGTTTCTTTATCAGCCATGGCTTTAGCAGTTAATTGTTGCTGC

TGATCCAATTGTTTATCTTTTTCAGCGATCTGCACGTCTTTAACACGCATTTGCTCATCT

TTTGAGGCAAGTTGAGCATCTTTGGCCTTGAGTTGCTCATAAAGGCGCGTGATTTCAGTG

TTCTTCTCATCCACAAGAATCTCTAAAAGCTCACGTTGTTTCGTTTCTTCACTGATAGGC

TCATCGTCAAAAATTGTTTTTTTATAGATCTCTTCCAGCTTAACAAGACCACTGCGCTTA

ACCACAGTAACGCCTTTTTCATTTTTGTCAAGATCTTCTTCGGGTAAAGACTTAACACGA

TTATTGACGGCCTGACGACTCACTCCCAAAATATCAGCTAGTTCACTAACTGTCTTTTCA

ATTCCCATATTTTCCTCTTTATCACTATCACTGAATATTCTTATTAAAAATAGTACCATA

ATAGGAGTATTCTGTCAATTTGACCTCATTTCAGTCGTGCTAAAGGCATCAATTATTTCT

TGAAAACATTGTTAGATGAATAGAAAACGGTATGCCAGGTTAGTTCATTTACCTGACTAT

TAAATCCCCCGCTTTTATGCTAAAATAAGGCTATGACTCAATACGATACGATTATTATTG

GAGGTGGCCCTGCTGGTATGATGGCCGCCATCTCCAGCAGCTACTATGGCTATAAAACTC

TCTTAATTGAAAAAAACCGCCGCTTAGGTAAAAAATTAGCGGGAACCGGTGGTGGACGCT

GTAATGTCACTAATAGTGGTAACCTTGATGACCTTATGGCAGGCATTCCTGGAAATGGCC

GCTTTTTATATAGTGTTTTTTCCCAGTTTGACAACCATGACATCATTGCCTTTTTCGAGG

AAAACGGCGTCAAACTCAAAGAAGAAGACCATGGACGGATGTTCCCCACAACAGATAAAT

CTAGGACAATCATTGATGCTCTGGAGAAAAAAATCAAGGCTCTTGGAGGCAAGGTACTAA

CCAACACTGAGGTGGTTTCCGTTAAAAAACAAGATGACCTCTTCTACCTCAAATCTGCAG

ACCAGACTTTTAGCTGTCATAAATTGATTGTGACCACAGGAGGCAAGGCCTATCCTTCAA

CGGGTTCAACAGGTTTTGGACATGATATTGCACGGCATTTTAAACTGACCGTGACTGATT

TAGAAGCTGCTGAGAGCCCTCTGTTAACTGATTTTCCTCACAAAGTCTTGCAAGGTATTT

CCCTAGACGACGTTACTCTTAGTTATGACAAACACGTCATCACACACGACTTACTCTTCA

CCCACTTTGGCCTTTCAGGACCTGCAGCTCTTCGTCTGTCCTCTTTTGTGAAAGGTGGGG

AAATTGCAGAGCTGGACTTTTTGCCACACTTATCAACAGATGACTTAACCGCTTATCTGA

GTGATCAACGTGATAAAAACATCAAAAATGCTCTTAAAGGCCTACTGCCTGAACGAGTAG

CTGATTTTTTATCAGAAAATTACCCCGAAAAGGTCAAACAACTCTCTCCAAAACAAGAAA

AGGAATTGCTTGATAAACTCAAACACCTCCAGATACCCATTACAGGAAAAATGTCTTTGG

CTAAATCATTTGTGACCAAAGGAGGCGTAGATTTAAAAGAAATCAACCCTAAAACCTTGG

AAAGCAAGAAAGTTCCTGGCCTGTATTTCGCTGGAGAGGTCTTAGATATTAATGCTCACA

CCGGAGGGTTTAATATTACCTCAGCTCTTTGTTCAGGATGGATCGCAGGGAAGTCTTCAT

AGCTAAAAAAGCCTATTATCACTAAGATAGTAGGCTTTTTAACTCCTATTACATGAGTTT

TTTGTTTTAGTCTCAGGCTGAATCGGTATGATTAAGTTAATAGCTATCAGTTTTTGTAGC

TTCGTTCTTAACAATCTTGATGATACGGCTAGTACCCAAGCGTGAAGCTCCTAAAGCAAT

AAATGTTTCAGCATCTTCCAATGATGAGATTCCACCTGCTGCCTTAATTTTAACACCTTC

GCCGACATATTTTGCCATCACTTCAACATCTTCAAATGTAGCACCTGCTGTCGAAAAACC

AGTAGAGGTTTTAATAAAGTCTGCACCTGAACGTGTGACAACTCCACAAAGTTCGATAAG

TTCTTCTTTAGTTAATTGACATGTCTCAACGATAACTTTTAAGATATGGTCTTGACATGC

GGCTTTGATTTGACGAATTTCTTCTTCAACAGTATCAAAATCCCCATTTTTAACGTCTGT

CAAATTAATGACCATGTCAATTTCATCAGCACCATTTTTAATAGCATCTTCACATTCAAA

AACCTTCGCTGCAGTTGTACTATAGCCATTTGGGAACCCAATAACAGTACAAATAGCTAA

TTTACCTGAAACGTATTCTGCTGCTTTTTTGACGTAAGAGGCTGGAATACATGCTGAAGC

TGTTTCATAAGCCATGGCATCATCTAAAATTGTTTGGATTTCTGGCCACGTTGCTGTTGT

TGCTAGCAAAGTATGGTCTACCGTTTTTAAAATATCTTTTACTTCCACGTTACTATCCTT

CTTTCTTTTGTCAACGTTTAGAATAGAGTTACAATACCGACGATAAAGGCGCTAAGCATA

CTTACAGCAATACCACCAATCATTGCACGGAAAACAAGTCGAGCTAGGGTGCTACGTTTC

TCCGGGCAAAGAACAGCAATACCTGAAACACAAATACCTAAACTTGATAAATTAGCAAAA

CCACAGAGTGAAATAGTTGCTACCAATGCTGTACGATAATCTAAAGATTTGATTAGGTGA

CCCAATTGTTGGAACGAAACAAACTCATTTAAAATCAACTTACTTCCAAGAAGGTTTCCT

TCTAGAAGAATGTTTTTGTGGTCAAATCCCATAAGAAAACCAAATGGAGCAAAAACATAT

GAGAAGATTTGTTCTAAGCGAATTCCCAATCCACTTAACATCATATTAATCAAAGAAACT

AAACCAACAAAGGCAATCAAACTAGCCCCAATTGAGAAAGCCATTTGTGCACCTGTGCTT

GCACCCTCAGCGATTGCATCAATCACATTGGCGTTATTACCTTTATTATCCATCTTAATG

TCATCAATTTTTTGAACAGGTTCTGTTTGAGGCAATAAGATTTTAGCAATGAGAATACTG

CCAATAGGAACCATTGTTGAAGCAATCAAGAGATATTCCATTGGAATGCCTAATGCAATA

TAGCCACCAAGAATAGAAACTGACATACTTCCCATACCTGATACCAACACAACCATTATC

TCACTATCAGTCATACGACCTAGGTATTTGCTAACCAAAATTGGACTGTCTGTTTGACCA

AGAAACATATTAGCTACGGCAACAAAACTCTCAACCTCTGAGGATTTCATAATTTTACCA

ACGCCCTTACCTATCCATTTTACTACAAATCCAAGGATTCCTACATAATAAAGTAGACTA

ACTAGGGCAGATAAGAAAACAATATTACCAAGCGTTTGAATAGCGAAAATAAAACCAGTT

TTTGCGCCACTATCTGCTAATGACCCAAACACAAAATTTAAACCAGCTTGACCACAGTTG

ATTACGCTAGTAACTCCAGTTGAAACAACACTAACAATTTGTTGGCCTAGTGGGATACGT

ACTAAGATTAGCGCAATAATGAATTGAACGATAAGAGCTTTTCCAATTAAACTTAGAGAA

ACACTCTTACGATTGAAAGAAATTGCATACACAATTCCTAATACCAATAAAATACCAATA

ATACTATAAATAAATTGCATGATGACTTCCAAAACCTCGCTTATTGTGATTTATCATTTT

CAATAAGTGTGCGGAGTGCTTCAACAGCAACTTGAATAGCTGCTTCTGTATCATGAGCCA

TTGGGTTATCCATACCCAAAGCGTTGCGTTCTTGATTACCAACAACAAGGAAATCTGATC

CACAACGAACTCCTAGATGACTTGCTGCTACAAAAAGAGCTGCAGATTCCATTTCAGACG

CTTTTGTTCCCAGACGTTTCCATGCTTCCCATTTATTTAATAATTCATAACTAACTGGCA

TACGTTCTGGCTCATGTTGCCCGTAAAATGCATCTTTACATTGAACTACTCCTGCGTGAC

TTGTATAACCAAGCTTTTTAGCTGCATTAACTAGAGCATTTGTCACTTCCAAGTCAGCTA

CAGCAGGAAATTCAATCGGAGCATATTCTTTACTGGTCCCTTCCATACGGATAGCTCCAG

TTGCAATAACGATGTCTCCGCCTTTAACATCAAGGTCAATACCACCACATGTTCCAACAC

GGATAAAGGTATCAGCGCCACAGAGTTTCAATTCTTCCATCGCAATAGACGCTGATGGAC

CACCAATACCAGTTGAAGTAACACTAACTTTTTCACCGTTTAATGTACCTGTATACGTAA

CATATTCACGGCTGTCTGCTACAAGAACTGCATTATCAAAATGTTCTGCAATTTTTGCAC

AACGTTTTGGATCACCTGGCATGATAACATAACGACCTACATCACCTGGACGAATCTGTA

GGTGATATTGCAATCCGACTTCACCTGAATAATTTTGCATGTTAAAAACCTCCTGTATTT

TACTTGTCTAATTTTTGATACACTTGTATAGTATCACACACACTCTATTTTGTAAACGCT

TTTTTAAGGTGTTTTTCATCTCTTTTTTGACAAAAAGTACCTGGGTGTGAGATACTAACA

CTATAGATGGGTACAATTCTAATCAAGGAATACTGTTAGTGCTAAAATAGTTTTTAGACA

AGTTAAACTCCACTAAAGATTATTAAATGAGGTAAGTGATATGTCTACTAACGACTTAAC

CAAAAAACTCAAAAAACTTAAACATGTGCAAGTATATAATACTATTTTTCAACTTATTCA

AGATGGTACATATAGTCCTGGCATGCAACTGCCATCAGAACCTGAACTTGCTAGACAGCT

CAATGTCAGTCGGATGACACTGCGTAAATCCCTAGCCCTCCTCCAAGAGGATCATCTCAT

AAAAAATATCAGAGGAAAGGGAAATTTTATACTCAAGACTCCTGAAACTAAATATCACCA

AGGTTTCGAATATCTTCAACACCCCATATATGCAAGCCTATCATCTGAAATCACAAAGGT

TGAATTGGAATATCGGATTGAAGTGCCCACTGTTGCCATTACAGCATCCCTAAAGCAAGA

AACTCCTGTTGTGATTATTGTTGATCGCTGGTATCATAGCCAAAATAAAGCTATTGCTTA

TAGTTTATCTTTTATCCCTATTGAGGTTATTTCCAAATATGCTATAAATCTCAATCAAGA

AGAGCCCCTTCTTACTTTCTTAGAAGAGAAAATCTATGAATCTGGTAAAGCTTCTCATTC

CTGCAACCAAATCGGCTATACCAAGACTGGCAATTACACAGCAACTAAGTATACTCTATC

AGAAAATAGTGCTTTTATTTTAATCCAAGAAACTCTCTACAATGGTAAAGACATCTTGGT

CTCAACCAAACACTACGTTCCTGCTGATTTATTTGACTTAAAAGTTCAATCTCAAAGTTG

CCAAAGCGACACTCATTAAACACCTTTAAACAGTTATTAATACCTGACTCCCACACTTAG

ATGTGGAGTTTTTTGATATTATGGCTAAAAAAGATAACTAGTTAATTTTTTTATTTGCTT

TTGCTTTTATTTATTGTATAATGATTATATATCCACTTAACCAGTTAAATACCAGTTAAG

TGGATTCAGAGCGCTGATAACGACTCTGAGGTTATTAAATATTAAGAAGGAGAAAGCAAT

GGCAAAAAAGTCAAAAATTGCTAAATACCAAAAGCAGCTCCAACTGATTGAACAGTACGC

TGACCTTCGTCGTGACTTAAAAGCGAAGGGCGATTATGAATCACTGCGCAAATTGCCTCG

TGATTCAAATCCAAACCGCTTGAAGAACCGCGACAAAATTGACGGCCGTCCTCATGCTTA

CATGCGTAAGTTTGGTGTTAGCCGTATCAATTTCCGTGACTTAGCTCACAAAGGACAGCT

TCCTGGCGTCACTAAAGCTAGCTGGTAAGCTTACTTCACCTTAGGCAGACAATTATTTTG

CAGGTAATTATCTGAATTATTAGTAACCATAGAAGCCAATTACTTATCATCATGACAACC

CGGTATGCCAAGATACCGGGTTGTTTTTTCAAAGGGAGATTCTTATCCATTATAAATCTT

ACTAACTAGCAAGTGTGACTAGTTTATTACAAAACTGTCTGGAAACTGGTCAAAAGCTAA

ACTAGGCTTCGCGTTTAGGCTCATATTAGCAAAATGCTGTTTGTCATACTCAATAGCTGC

TGCTAAAGCAATCATACCCGCGTTATCTCCACAGAGTCTTAGTTTAGGAATCACAACTTC

TATATGTGTAATTTCTTGTGCTAATCTATCTCGTAATCCTTGATTTGCCGCTACACCACC

AGCCACTACAAGCATCTTTGCAGGGTATCTGCTCAGAGCTTTTTTTGTTTTGGCCAATAA

AATATCTAGAACAGCTGCTTGAAAAGATGCACACAAATCTTCTAGAATCAGTTCATTGCC

TTTTTGCTTAGCATTATGATGAAGATTGATAAAAGCGGATTTGAGTCCTGAAAAAGAAAA

TTCTAAGTGGTCTTCTGTTATCATAGCACGAGGGAAATGATAAGTATCTTGACCTTTATG

AGCCAACTGATCAATTTCACGTCCTGCCGGATAGGTTAAGCCCATCACACGCCCAACTTT

ATCATAAGCCTCACCAACAGCGTCGTCACGCGTCTCGCCAATAATATGATAATCCCCTGG

CTCTGGGACATAAACCAATTCTGTGTGTCCTCCTGAAACCAATAGTGCTATTAAAGGATA

AACTAAAGGCTTTTGTTCACGCGCTGCCATTAAGTGACCTGCCATATGATTAACCGGAAT

CAAAGGTAAATGATTTGCCCAAGCAAAAGCCTTCGCAGCTGCTAATCCAACTAACAAGGC

TCCAACAAGTCCAGGGCCATAAGTTACCGCAACTGCTGATAAATCTGAAGCACTGATGCC

AGCTTCTTGCAAAGCATCCTCAAAGCAAGTCGTAATCACTTCGACATGGTGTCTACTAGC

TACTTCTGGCACCACACCACCAAAGCGTTTGTGGCTCTCAACCTGACTTGCAATGACATT

ACTTAAGAGGGTACTTTCGTTTTTTAAAATCGCTACACTGGTCTCATCACAAGAACTCTC

AACTGCTAAAATATACCTATCTGTCATGTTTTCCTTCCCGTTTCATCAATAAAGCTGTTT

CTATAGGATTTCTATAGTAGTCTGGTCTTTTGCCAATAAGTTTAAAACCAAACTTCTGAT

ATAAACCCTGTGCTCTATGATTAGATTCTCTAACCTCTAAAAAGATGTCACTTTCAATGC

TATCTAAATGCGTCATCAATTGAGACGCTAGCCCAAGTCCTTGATGACTAGGTAGTATAG

CAATTTGAGTCATTTCAACTTCACCAGCTAAATCTTGAACAGCTAAAAAACCTAGCAATT

TATCATGATCATATAGTAGGAAATAATCTGTTTGATCCCGCCGTATATCGATTAATACTT

GTTCTAAAGTCCAAGGAGACGTGCCATAAACCATTTCCAAAAGCTGATAAATATTTTTTG

CTTGTTCTTCAACTGTTTTCATATTAGATTCGCTTAACGTAGTGACTATCATCTTTTATC

TCATTATCTTTGAGCCAGTTTTCTTCAGCTTCCACTCTCTTGAGATATTGAGGGACAAAG

GCGTCTACATTTTCTGGTGCCAAACTTTGCCCCAAAAGACCACATTCGTAAGCAGAAGGA

AGGGTTGGAAGTAGTATCGCCTGAGGTAGTTTCTTTTGAATTTGCTCAGCAAAAGGAGTA

GTCTCCCCAACAAAAATCAGCTGTCCTTCTTCTACTAATTGTTCTATAATAACTTCTAGT

GAAGCATGGGCTTGTGGCATCACTGATTTTCCTTGCCGATAATAACCTACATACGCATTT

TGCCTTCTAGCATCAATCAATGGCACCACCAAAGTATTTGGATATTGTTTACAAGTAGAC

GCAGCCAAAGCATATAGACTGGAAATCCCGACCAATGCAATATTTAAACTGTACGCTAAC

GTTTTTGCAGTAGCAACTGCCACTCGTAAACCTGTGTAAGATCCAGGGCCTTTTGCAACC

ACTATTCTTTCTAAATCTTGAGGTTTAAGATCAGTACAAGTCATCAAAAAATCAATAGCA

GGCATAAGGCTAACACTATGATTTTTCTGAATGTTAAGGGTCATATCTGCTAGAAGTGTC

TCATCATCAAGTATAGCAAGGGACAAGGTTTTATTTGAGGTATCAAATGCAAGTGTCTTC

ATTTTATGCCTTTCTACTGCAAGTTCTAGTATCAATTATAACACAAATTAAGAATCATAA

ACAAAAAGGCTGACTCTTAATTTGATTTAGACTATACCATAAATGTACCTAAATTTTTTA

CTAAAAGCTAAGTTTATGGTATAATTATAATAATTGCACAGAATCAGGAATTAATCTACT

ACACAATAATGACAACATATAAAACCGTCAAAATGGAACACCTACCTTTTGACCTATTTA

TTATTGTTTGGATAATTGATTCATAACGAAAGGAATAAAAGACAAATGATTTATAAAGTT

TTCTATCAAGAAACAAAGGATCAAAGCCCACGTCGTGAAAGTACTAAAGCACTCTATCTT

AATATTGATGCTACTGATGAACTCGATGGCCGTATCAAGGCTCGTCGTCTCGTTGAAGAT

AACACCTATTATAACGTGGAATTTATCGAGCTACTTTCTGACAAACACCTCGATTACGAA

AAAGAGACCGGTGTTTTTGAATTAACGGAGTTCTAAGATGACAAATATCAGTTTAAAACC

TAATGAAGTTGGGGTGTTTGCCATTGGTGGTCTGGGAGAGATTGGGAAGAATACTTATGG

TATTGAGTATCAAGACGAGATCATTATCGTTGATGCCGGTATCAAATTCCCAGAAGATGA

TCTTTTGGGAATTGATTACGTTATTCCAGACTATTCTTACATTGTCGATAACTTAGACCG

GGTAAAAGCTCTTGTTATTACACATGGTCACGAAGACCACATTGGTGGTATTCCATTTCT

TCTCAAACAGGCTAATATCCCTATCTACGCAGGACCTTTAGCACTTGCTCTTATCCGTGG

TAAATTGGAAGAACATGGTCTTTGGCGTGAAGCAACTGTTTACGAAATCAATCACAACAC

CGAGTTAACTTTTAAAAACATGAGCGTCACTTTCTTCAAGACTACTCATTCCATTCCAGA

ACCGGTCGGTATTGTTATCCACACCCCTCAAGGTAAGATTATCTGTACTGGTGATTTTAA

ATTTGACTTCACGCCAGTAGGAGATCCTGCAGATTTACAACGTATGGCAGCGCTTGGTGA

AGAAGGTGTCCTTTGCTTATTATCAGACTCAACTAATGCTGAAATCCCAACCTTTACTAA

CTCTGAAAAAGTTGTTGGCCAATCCATTTTAAAAATTATCGAAGGTATTCATGGTCGTAT

TATTTTTGCTTCCTTCGCTTCAAACATCTACCGTTTGCAACAGGCTGCAGAAGCTGCTGT

TAAAACGGGACGTAAAATTGCCGTTTTTGGTCGTTCTATGGAAAAAGCAATTGTTAACGG

TATTGAACTTGGCTATATTAAAGTTCCAAAAGGTACCTTCATTGAGCCAAGCGAACTTAA

AAATCTTCATGCTAGTGAAGTTTTAATTATGTGTACAGGTAGTCAAGGTGAGTCTATGGC

AGCACTTGCTCGTATCGCTAATGGCACTCATCGTCAAGTAACTTTACAGCCTGGTGATAC

TGTGATTTTCTCATCAAGTCCAATCCCTGGTAATACCACAAGTGTCAATAAATTAATCAA

TACCATCCAAGAAGCTGGTGTTGATGTTATTCATGGAAAAGTCAATAATATTCATACTTC

TGGTCATGGAGGACAGCAAGAACAAAAGCTCATGCTTAGTTTGATTAAACCAAAATATTT

CATGCCAGTACATGGTGAGTATCGCATGCAAAAAGTTCATGCAGGACTGGCGATGGATAT

TGGTATTCCAAAAGAAAATATCTTTATTATGGAAAATGGAGACGTTCTAGCTTTAACCAG

TGACAGTGCGCGTATTGCTGGTCACTTTAATGCCCAAGACATCTACGTGGATGGTAATGG

CATCGGGGATATTGGAGCAGCCGTTTTACGGGACCGCCGTGATTTGTCAGAAGATGGTGT

TGTCTTAGCTGTTGCAACTGTTGATTTTAACACACAAATGATTTTAGCTGGACCAGATAT

TCTGAGCCGTGGTTTCATTTATATGCGTGAGTCAGGTGACTTGATCCGCGAAAGCCAACG

TGTGCTCTTTAACGCTATCCGTATTGCTTTAAAAAATAAGGATGCTAGCATCCAATCAGT

TAACGGTGCTATCGTCAATGCTCTTCGTCCATTCTTGTATGAAAAGACTGAGCGTGAACC

AATTATAATTCCGATGGTCTTAACACCTGATAAGCACTAAAATCAAATAATACTCATTTC

TTAATACTATCTGTCTATATCTTTAACATATCAGTTCTAGTTCAGATAATTATCAAACTA

AAAACCAGTCCTCATAGAACATGGACTGGTTTTTAGTTTTCTATAATGGAAAATCAATTG

GTGTCCGATCAGCTAATAATAGCTTGCTAGCTCAAATACAATAGCTCATCCAAAAGACCC

ACTCAACTTGAGCAGGTCCTGATAACTCTAATAGTTATGAATATAATGGTCAATTTCCCA

TTGAGAAACAAAAGTTGCATAGGAAGACCATTCAATTCGTTTTGCTTCTAAGAAATTAGT

GTAGATATGGTATCCTAGTGCCTTTTGTACCACATCATCTTTTTGAAGAGCTTTTAAGGC

ATTATGAAGCGTTGATGGCAAATCAATAATGCCTGCTTCATTTCGTTCTTCCATTGTCAT

GGTATAAATGTTAGCTTCAACGGGTTCTGGAGCTTCAATTTTGTTAATGATACCATCTAA

TCCAGCTTCCAAGAGAACAGCCAAGGCTAAATAAGGATTAGCTGTCGGATCAACCGAACG

TAACTCCAAACGCGTTCCCATACCACGTGATGCTGGAACACGGATAAGCGGTGAACGATT

ACTTCCAGCCCAAGCGACATAAACAGGTGCCTCATAACCCGGAACTAATCGTTTGTAAGA

ATTCACTGTAGGGTTAGTGATAGCAGTGTAGTTATAAGCATGCTTCATTAGTCCTCCCAA

GAAATAATAAGCATCTTCTGATAGCTGCATTCCTCGCTTATCAGTTTCATCATAAAAAGC

ATTATTACCTTGGTTATCAAACAAAGACATGTTACAGTGCATCCCTGATCCAGCTATTCC

AAATTTTGGTTTAGCCATAAAAGTAGCATAAAGTCCATGTTCACGGGCAATCGTTTTTAC

AACTAGCTTAAAAATTTGAATATTATCACAAGCTTTCAAAACATCTGCATATTTAAAATC

AATCTCATGTTGACCAACAGCCACTTCATGATGACTAGCTTCCACTTCAAAACCCATTTT

CGTTAAAATATTCACAATTTCACGGCGCGTGTTGTCTGCTAAGTCAATTGGCGCTAAATC

AAAATAACCACCATTATCGTTAACTTCAAGTGTCGGATTACCTTTATCATCCATCTTAAA

AAGGAAAAATTCTGGTTCTGGCCCAAGATTAAATGATTTGTAGCCGATCTCGTTCATGTG

TTTCAGGGCTCTTTTTAAATTTCCTCTAGGATCTCCTGCAAAAGGCTTTCCTTCTGCTGT

ATAAATATCACAAATTAAACCTGCAACTGCTCCATTTTCATCTCCCCAGGGAAAAACAAT

CCAAGTGTCTAAATCGGGGTAAAGGTACATATCTGACTCATTGATCCGTACAAAACCTTC

GATAGATGAACCATCAAACATAACCTTGTTAGACAATACTTTGTCTAACTGTTCTTTCGT

TGCAGGAATCTCCACATTTTTCATAACGCCCATGATATCAGTGAACATCAAGCGAAGAAA

CGTTACATTTTTTTCTTTGACTTCATGACGAATGTCAGCTACTGTGATTGCCATTAAATA

GGTCTCCTTTAAATTTTATACAATTTATATCTTAAATACGAAAATTACCAATATGCTGTG

AGGGAGTAGAAAAACCGCTTTGTGTCAGCATTTCATCATGTAAGATCCGTCTGACATCTG

CATCTGTTAGAGCTTTTTGTTTTTGCATTAACTTACCTTGACGCTCGACATATTCGCGTT

TAATAGCTGCAATATTTAATCCCTCAGACAAAAAATCTTTAATTTCCAAAAGTCGGTCCA

TGTCATTTAAGGAAAACATACGCCGATTCCCTTGCGTTCGTTCAGGCTTAATTAAACCTT

GATCTTCATAATAGCGAATTTGTCTCGCAGAGAGATCTGTTAATGTCATCACCGTTCCAA

TAGGAAAAACAGCCATTGAGCGTCTAAGTTCTTTTTCTTTCATGACAAACTCCTTTCATC

TTCCTTATTATAGGCTGAAAAAAATAACCTGTCAAGTATATATGTTATATTATCTAACAT

CACTGTGTTTTTTGTTAAACCATTTATTTTTAATAAGTTCAACATCAGTATTAACTAAAA

TAGCTACAAAGACGCCACAAAATGTTTCAAATACCCGCGAAGTCACATAAATAAACGTTT

GTCCTGTTGGAATAGACAGTGTAATAATCAAAAGAGCAGCCACTGCTCCAATAATACCTG

ATTTATTATTACATGCAACATTAACCATGATACATAACATGGTACAAATAGGAACAATTA

ATAAAGTAACCCAAAAAGCTTGATGAAATATCTCATTGAATGCAAAAAATACTAAGGAGA

GTAGGCCACCTATACTATTACCAATAATTCTAGAAAAGCCAAAGTGTACGCTTTTATCAA

AGTCTTCTCTTAAACTAAATACAGCAGTTAGAGCACCAATTTGGAGCCCTTTCCAACCAA

ATAAATGAAAAACCAATAAAACTAAAAAGACCGATAAGCCTGTTTTCAATGTTCTCATTC

CCAATTTAAACTTTTTAATATCAAACTTGTAATTAGATAATAATTTTCTTAACGTTTTCA

TACCTATCCCTTCACTATCTATTTTAACATATCAATCCATAAAACCCTTTTGGTAAACGT

TTTATTTATGATTAGGATATCTTTTATAAACCAATTGATATGAAATCGTCATAAACTTTT

GAAAATTGCAGTGTCAGCCAAATACCACTATATCTAAAAACTAGTAAATGATAAGTGAAG

TTAGGGAAAGTTCAATAACTGTACATGATCAACTAAAAAAAGACATGCATCAGCATGCCC

TTTACTATATATTATAATGACTATTATTTTTCAGTCAATGCTGCCAAACCTGGTAATACT

TTACCCTCGAGAAGTTCCATGCTAGCGCCTCCGCCGGTACTGATCCATGAGAATTTGTCA

GCACGGCCAAGGTTGATAGCAGCTGCTGCTGAATCACCACCACCGATGATTGATTTAACG

CCTGGTTGTTTAACGATAGCGTCCATGACACCGATTGTACCAGCTTGGAAGTCAGGGTTT

TCAAAGACACCCATAGGTCCGTTCCAAACAACTGTTTTAGCACCAGTAAGTGCTTCATCA

AATTTAGCGATTGATTTAGGACCGATGTCAAGACCAAGGAAGCCTTCTGAAACTGCTTCA

CCTTCAGTGTCGCGAACTTCAGTGTAACCAGCAAATGCGTTTGCTTCTTTCGAGTCAACT

GGCAAGATCAATTTACCATTTGATTTTTCAAGGAGGTCTTTAGCAACATCCAATTTGTCT

TCTTCTACAAGTGAGTTACCGATTTCGATACCTTGAGCTTTGTAGAATGTGTAAGTCATA

CCACCACCGATAAGAACTTTATCAGCTTTTTCAAGAAGATTTTCGATAACACCAATCTTA

TCAGAAACTTTTGAGCCACCAAGAATAGCTACGAATGGACGTTCTGGAGTTTCAACTGCT

TCTTGGATATAAGCAATTTCGTTTTCAAGAAGGAAACCAGCTACAGCTTTTTCAACGTTT

GCTGAAATACCTACGTTTGATGCGTGAGCACGGTGTGCTGTACCAAATGCATCGTTAACG

AAGATTCCATCTCCAAGTGAAGCCCAGTATTTACCAAGTTCTTCGTCATTCTTAGATTCT

TTCTTACCGTCAACATCTTCAAAACGAGTGTTTTCAACCAAAAGAACTTGTCCATCTTCC

AAAGCATTGATTGCTTCTTCTAATTTTGAACCACGAGTAACACCTGGGAATACAACATCT

TGACCAAGTTTAGCAGCTAAATCAGCAGCTACCGGTGCAAGTGATTTTCCTTCTTTGTCA

GCTTCTTCTTTAACACGTCCAAGGTGAGAGAAGAGGATAGCACGACCACCTTGTTCGATG

ATATACTTGATTGTTGGAAGAGCCGCAGTGATACGGTTGTCGTTAGTGATAACGCCGTCT

TTCAAAGGCACATTAAAGTCAACACGAACGAGGACTTTTTTACCTTTCAAATCAACGTCT

TTAACAGTCAATTTAGCCATGTGAATAGACTCCTTAATATTTTTTATACTCCTCCATTAT

ACCACAATTCCTAGCATAAAGATAAAATATGAAAATAATAAAATAATCATTTCACAAAAT

CAACATTTTTTTCAGTCATTAACCCTATTTTCAGAAAATAATAGTACTTAAAAAGAGGTT

ACATTTTCTTTTGCACCTCTTTTTCAATTACTTTATTTAAAGCTTTTTAAACTTTTACGG

CGTTCCTCTAGTTGCTTAAGCACATCCAGCTTTTCACCTTTATAAATGGCACCTTCCCAT

GAACCATACATAGGATTAGGGAAAATGATAAAGCGTCTTCCAAACTCTTCTTGCAAATCT

GATAATAAAGCTGTTCTATCTTCTTGAGATTTTTTTGAAAAATCAGCAAAATCTAGAAGA

TTATCACCAAATAGCATCGTTACATTAGTTGTTTCTTTGACCTTTTGACGACGACTCTCC

TTTGATTTTACGCCTTTTTCTAAGAATAGAAGATGATCACGACCTTGTACTGGAATACCT

TCTTTTTGGAGATTTTCCATTGTAGCATCTACTTGAGTAGTTGATCTGTCTGAAATGTAG

TAAATTTGAACACCATTTTGGTCTGCAAATTGCAAAAAGTCTTTAGCACCAGCAACCGGT

TTTGCTTCTTTCTTTTGTACCCAATAATCCCAGCTTTCAGGTGTAAATCCTGTTCCTTCC

AAAACATTTTTAGCTTGATAAGGGCTATTATCAAGAACTGTTTCGTCAATATCTAATACA

ATCGAATAAGGTTTATCCGTTGGTTTATTGAGTTGTTCTTTTAAGCGATCCGTTGCTAAC

TGATACCCCTGTAAATAAAGCGCTTGAGTTTCCGCAGCTCGCTGGTACCATAAAACAGAC

ATGGTATTTTCACGCGATCTTAATTGGTCATCACTATAGCTATTATAGGTTTGTTTAGTA

GCAGGCTTACGATTAACTGATTTGTTGTTATCAACTTTAGCACAACCTGTCACCAAAAAA

AGGGATAAGGTAAGTGATATAACACTAACAACTTTTTTGGATTTCATTGAAAATCTCCTT

TTTGCTTATTAAGTTGATTTTAAACTTATTTAATTTAAAAGTCAATACAACTTAACATTT

AATCAATACTATGTTGTTAATCATGAAAAAGGCTTTTTTTACTTTCATTTCTCCAATAAA

AGTAATGTATTCATTTCGTCGTTTGCCACTTCACAAGGTATAATAGCCTTCTGCTAAATC

CAGTATTGAGCAACTTTTTAGCCAAGCTTTTACACGTTATTTTGTTTTAAGGGCAGATAA

AACTTGTGTACGGATATCCTCTACCCCACTTGGTGTATTTGGTAGAAAAAGGGTTTGGTT

TCCTTTTGCCGCAAAGGTATTGAGGGTATCCAGGTATTGGTTGGTCAACAAAATGGACAT

GATTTGTTCTTCGTTTAAGGAAATATTCGCTTCTTTAAGCTCTTGAATAGACTCCGCCAA

ACCATCCACAATGGCCTTACGCTGTTGGGCAATCCCTACCCCGTGCAATCGATCTTTTTC

AGCCTCTGCTTCTGCTGCTGTCACAATTTTAATTTTATCTGCGTTTGCCAACTCTTGAGC

AGCTACACGTTTGCGTTGAGCCGCATTGATTTCATTCATTGACTGTTTGACTTCAGCATC

AGGCTCCACCTTGGTAATCAAGGTTTTGACAATAATATATCCATAGGTTGACATTTCTTC

TGCCACTTGATGTTGTACTTCCAAGGCAATTTCATCTTTTTTCTCAAACAATTCGTCTAA

GGTCAACTTCGGCACGGATGACCGCAAGGCATCTTCAATATAAGACTTGATTTGAGATTC

AGGTTTCATTAATTTATAGTAAGCATCCGTTACATTTTGCTCGTTAACACGGTACTGAGT

GGCAACATTCAAGGTCACAAACACATTGTCCTTGGTTTTGGTTTCAACGATGATTTCTGA

CTGTAAAAGACGCAGCTGCACACGCGCAGCAATTTTGTCAATCCCAAACGGAAGTCGGAT

ATGAATACCACTAGTGGCTGTTTTTTGGTACCTACCAAAACGTTCCACGATGGCAACAGA

TTGTTGGCGAACCACATAAAGGGTGCTGGCAACAATAGCTAGGATTATAATAACCCCAAA

AGCAATAAAGATAAATGGTTCTAACATAAAATCTCCTCCTTGTAATGTCTTAGAACTGAA

CCGTTTTCAGCTCTTGACTTATTGTTATAATACAATTATAATGTTTTTCTAGGTTTTGTC

AAATTAAATCGTAACCATTAGCTTTGTGATGGATTACCCTCATTTTTTAGGGGCTTACCA

TCACTAAAACCACTCATTACGACATCAATATAGACAAAAAGAGTCAGACCTTTGCCTAAC

TCCTCTTTTTTTATTGTATTCTATTATTCTACACTCATGAGATATGGGTAAACAGGCTGA

TCTCCTTGATGGATCTCAACTTCCACATCTTCGTAAGTCTCTCCTAAATACCCAGCAAGT

TCTTCTGCTAAGTCTTGGTCACCTTCTTCACCAACAAAGATAGTCACAATCTCGCTATCT

TCGTCAATCATCTTTTCAAAGGCAGCTTTTAGAGTTGCTTCCATGTCTGGGTTTGAAACG

ATGATTTTACCATCAACCATTCCCAAGAAATCATTTTCATGGATTTCTAAACCATCAATG

GTGGTGTCACGGACTGCCAAAGTAACGCTTCCACTGACCACGTCAGAAAGACTAGTTGAC

ATATCTGCTACATTGTCCTCTAAAGATTTTGAAGGATCAAAGGCCAACAAGCTGGTAAAT

CCTTGCGGAACAGTACGTGTTGCAACAACAGCAGCCGGAATATCCACCACTTCTGCAGCA

GATTGGGCAGCCATAAAGATATTTTTATTGTTTGGCAAGATAATGACTTGTTTGGCATTA

ACTGCTTCAATAGCTTTAACAATATCTTCTGTAGATGGATTCATGGTTTGACCACCAGAA

ATCACGTAATCAACACCTTGCGCCTTGAAAATTTCAGATAAACCTTCTCCAGCTACTACT

GCAATCAAGCCAAAGTCTTTTACTTCAGCCTTGTTTTTTTCGACATCAGTCTTTTGAACC

TGTGCTTCATGCTGGTTACGCATATTGTCCACTTTAATCTTGATGAGCGAACCATATTTA

AGACCTTCTTGCATCACAAGTCCGGGGTCTTCAGTATGGACGTGAACTTTAACAATCTCA

TCATCATTGACCACTAACAGCGAGTCACCAAGGCCACTAAGGTAGCCTTGAAACTCATCA

TAGTTGAATTCTTTCACATAGGTTGGGCCTTGTTTAAGGGCAACCATGATTTCAGTACAA

TAACCGTAGGTAATGTCTTCAGTAGCCACATGACCAACAACGGATTTGTGATGCTCAGCA

TTAATCATTTCAGACATGTTAGCAGGAGTCGCTTTAAAGTCTGCTGAAGTCACATAGTCG

CCATTCAAAGCAGATAGGAAACCTTCATAGATAAAGACAAGACCTTGACCGCCTGAATCA

ACCACACCAACTTCTTTAAGAACAGGAAGTAAATCAGGAGTCTTAGCAAGGGCACCTTTG

GCACCATCTAAAGCAGCTTGCATGACTTCCACGGCGTCGTCTGTAAGGTCTGCTTTTTTT

AAGGCAGCAGTCGCTGCACCCCGAGAAACAGTTAAAATTGTTCCTTCAACAGGTTTCATC

ACTGCTTTGTAGGCAACTTCTACACCCACTTGAAAGGCTTGTGCCAAATCTTTACCTGTC

AGCTCATCTTTTCCCTTAATGCTTTGACCAAAACCACGAAAAAGTTGTGAAGTAATAACT

CCAGAATTTCCACGCGCTCCCATCAAAAGACCTTTTGATAACATTTGTCCAACTTCTCCC

ACAGTTGACGCAGGTTTATCAGCAACTTCCTTGGCACCATTGTCCATAGTCATGCTCATG

TTTGTTCCTGTATCACCATCTGGAACAGGAAAAACATTTAAGGAATTAACATATTCTGCT

TGTTTGCCAAGGCGAGTCGCTGCCGCTTGGACCATTTCTTGGAATAAGCTTGTTGTAATA

TTTGACACTAATTTTCTCCTACAACCTTAATATTTTGCACGTAAACATTGACCATATCAG

CAGTGAGACCAAGCTGACTTTCAAGGTTAAATTTCACGCGTTCTTGGATATTTTTAGAGA

CTTCACTGATTTTCACACCATAACTCATCACAGTGTAGACATCAACAGAAATACCCAGAT

CTGTTGATTTTACTACAACACCCTTAGCGTAGTTTTCTTTGCGAAGTAGTGATTGAAAAT

TGTCTTTTATTGCACTTTTGCTGGCCATTCCAACAACACCAAAAATTTCCGTTGCTGATC

CTCCAACGACAGTTGCAATCACATCGTCGGATAGCTCGATTAGACCATCTTTTGTATTAA

TTTTTACAGTCATAGTTACTACCTCAAAAAGTTTTTATCATCCTATTTTATCATATTTTA

GAAGTAATGTAAAAGAGATAGAATAGTTAACGATAGCGTTTATTACCCTGACTAGCTTAT

GGATAGAAATAAGCTAATAAAAAAAGCCCTAAGGCTTATTTCTATTAGATGCGTTCTACT

TTACCAGATTTAAGCGCACGAGCAGAAGCCCAAACTTTTTTAGGTTTACCATCAACAAGG

ATAGTAACTTTTTGAAGGTTTGGTTTAACTGTACGTTTTGTTTGGTTCATCGCGTGTGAA

CGATTGTTTCCAGAAACGGTTTTACGTCCTGTAAAATAACATACTTTAGCCATTTTGATC

TGTTTCCTCCTACGTAGAATATGTTTACGGATGTGCTAGCACCACATACCTATCTATTCT

ATCATAGGGTGCTTAATTTGACAAGGTATTTCCCATTTTCTTTTTCAACAATTTTACGGA

ATTACTATTACAATAGTCTACGTACTCATTTTTCACTACATTTTTTAGCTAAAACATATT

AACATTACGCTTAACCTCTTTAACAATAACAAAAAGACTTACCATAGATAGCAGGGTCCA

GATTTTAATACTCTAAATTATGCTTTGTTTGTTCACAATCAAAGATTATTTCACAGAAAC

TGGTATCCATTTTAGGGTGTCAATTATATCTAAAAAAGCCCTCAAGAGGGCTTTAGGGTG

TTTAATACTCTAAATTATGCTTTGTTAGCTGATCCGAAAACATCGATACGTTCTTCAACA

GCACCTTGAACAGCTTTCATACCTGGAGCCAAGAATTTACGTGGGTCAAATAATTTTTTA

CCATCATATTCTGCTTCGTTAGCTTCGTAGTTACGAGCAAATTCACGAGTTGCGTTAGAG

AAAGCGATTTGACTTTCAGTGTTAACGTTAACTTTAGCAACACCAAGACGGATTGCTTCT

TTGATTTGATCGTCAGGAATACCAGAACCACCGTGCAATACGATTGGGAAACCTGGTACA

GCTGCTGTTAATTTTTCTAAGTGGTCAAGAGCAAGACCTTCCCAGTTTTCTGGGTATGGA

CCGTGGATGTTACCGATACCAGCTGCCAAGAAGTCAATTCCGGTTTCAACCATTGCTTTA

GCATCTTCGATTGGAGCGAGCTCACCTTTACCGATGATACCGTCTTCTTCACCACCGATT

GTACCAACTTCAGCTTCAACTGAAACGCCTTTAGCATGTGCAATTTTAACAACTTCTGCA

GTTTTAGCAAGGTTTTCTTCAACTGGAAGGTGTGAACCGTCAAACATGATTGAAGTGTAA

CCTACTTCGATACACTCAAGAGCATCTTCGTAGTGACCATGATCAAGGTGGATAGCAACA

GGAACAGTGATACCCATTGACTCAACAAGGTTAGTAATAAGAGATTGACATACTTTATAA

CCACCCATGTATTTTGCTGCACCCATTGATGTTTGGATAAGAACTGGAGCTTGTTTAGCT

TCTGCTGCACGCAATATTGCTTGAGTCCACTCTAAGTTGTTTGTGTTAAATCCACCAACA

GCATATCCGTTTTCACGAGCTGCTTGGACAAATTTTTCTGCTGAAACGATTGCCATTTGT

AAGGCCTCCTCTTATTTTTCGGGATAACTCCCCGTTTACATTGTTCATTCTAGCACAATT

TTCCATAAATTTCTAGTCTTTCACAAGCTTAAAGCGTTTTCCAAAAGCAGATTATCAAAA

GTTTTCTCCAGTAAATCTATGCCTTCTTATCCTTCATTTAACCAAAAAAAGAGACGAATC

AATGATTCACCTCTTACATTATGCGGAGAGTGGGACTTGAACCCACACGACCTAAAGCGG

TCACAGGATCCTTAGTCCTGCGCGTCTGCCAATTCCGCCATCCCCGCTACTCATCAGCAA

CATAAATTATTATAGCAACTGTGAGTTTAACTGTCAATACTTATTTCTCAACTTTTTTCA

AAAAAGCGGCAATTTTTTTCTGGTATTGTTCTGGGTTTGTTTCAAAGGATTTGGCGTGTT

TTGCCCCTTTAACAATCAAGATTTCCTTAGGACCTTTCGTGGCCTTATAATTGTCATAAA

CCATTTTTGTAGGAACAAAATCATCCTTATCACCATGGATAAATAAAGTTGGACGTTTAT

TTTTAGCCAGCTGTTTCACTGAGCTCGCTTCTCCGTAACTAAAACCTGCTCGAATCTTAG

ATAAGGCAGAGACTTCATAGAGTAAAGGAAAGGCAGGCAAGTTGTACATAGCCTTGGCCT

GAAACTTCAATTCGTCCCAAACACTGGTATAACCGCAATCTTCGATGAGGGAGGTGACTT

GCGCAGGCAATCGCTCACCACTTGCCATCATTACTGTTGCAGCACCCATAGATAAGCCAA

AGAGTGTGATTTGGCTTTCAGGGTTTTCCTTAATCAGTTGGTCTGTCCAAGCCATGACAT

TAAGGCGGTCATTCCAGCCATAACCAATCAAGTTCCCTTCACTTTCCCCATGGGCCTCAT

TGTCTGGCATTAAGACATTATAGCCCAAATCATGAAAAAGCATGGCATATGGCTTCATAT

CTTCTTTGTCATTCGTAAAACCATGAACAACAATAGCTGTCTTTTTTGTTTTTTGAGCAG

CTGGTAAGTACCACCCCACTTGTTTTAACCCACGATTTGTTAGTTGACGTTTTTCGTAAG

GTAAAGCGTCAAAAGACTGTTCAGCTGGGTATAATGGATTATTCGTACTCCGTTTCTTAT

TATTAATAAACGATTTCTCTTCTCTTATTTGTGCAACATGAAAGAAATAAAAACTAGCAC

CTACGCTAATAAGAGTTATCAAAAGGAATAGAATTCCTAAATACTTTGCTATTCGAATAG

TTTTCATACCAAGTATTTTACAAAAAAATGACTGAAAAAGAAAGTGCTAACCTAAAAATA

TCACGTTTTTCTTTTTCTAATGCTATTTTTATAGAAAAGATCCCTCTAATAGCTAGTAAT

CTTTATCAGTATCTGCTAGCCAAGCAGAATGCCACAAACTTACCCTTAAAACCACTAATT

GCTATTTTTAATAGCAGCTGTAACAAAAGCGGTGTATAGCTCCTCAGGACGATTTGGACG

GCTTTGCAATTCTGGGTGGTATTGAGCTGCTACAAAGAATTTTTTCTCTTTCAATTCTAC

AATTTCAACTAAGCGGTTGTCAGGTGATACCCCTGAGAAAACAAAACCAGCTGCTTCAAA

TTCTGAGCGGAATTTGTTGTTGAACTCATAACGGTGACGATGACGACGTTGCACCACCTC

TTGATTATTGTAAGCCATTGCTGCTTTTGAACCTGGTTTCAATTTACATGGGTACAATCC

AAGACGAAGCGTACCTCCCATATCTTCAATATCAATTTGATCACGCATGATATCAATGAT

GGGGTATTTAGTACTTGGCTCTAACTCAAAAGAGTTTGCACCTTCCATATTGAGGACATG

GCGGGCAAATTCAACACAAGTCAACTGCATGCCAAGACAGATACCAAGCATTGGCACATC

ATTTTCGCGGGCATAGCGAATCGCTTGGATCTTGCCTTCTGTACCACGTTGACCAAAACC

ACCTGGTACAATAATCCCATCCGCATCTCCCAACAAGTCAGCTGCATTATCCACCGTCAC

ATCGTTAGCATTGACCCATTTTAGGTCAATTGCGGTATCATTGGCATAACCAGAATGTTT

CAAGGCTTCCACAACAGATAAGTAGGCATCTGGCAATTCCACATATTTACCAACCAAGGC

AATTTTTGTTGTTTTTCTTAAATTCATCACCTTGTCAACCATAGCTGACCACTCGGTCAT

ATCAGCTTGTGGCGCATTCAATTTCAAATGATCACAAACAATCTGATCCATTGATTGAGC

TTGCAAGTTCAATGGAATTTGATACAAGTGTTCTACATCACGTGACTCGATAACAGCTTC

TGAATTCACATCACAGAACTGAGCCAATTTATTTTTAATCCCCTGCTCAACTGGCTCTTC

CGTACGAATAACCAACATATTCGGTTGAATCCCTAAACCACGCAATTCTTTAACGGAATG

TTGCGTTGGCTTAGTTTTCATTTCACCCGCTGCCTTTAAATATGGCAACAAGGTGGTGTG

AATATACATCACATTTTCAGAACCAACATCAGCTTTCATCTGACGAAGAGCTTCTAAGAA

CGGTAAACTTTCAATATCGCCAACAGTCCCCCCGACCTCAGTAATAATTACATCAGAGTC

CGTTGTTGAAGCTGCGCGTTTGATTTTTTCTTTAAGGGCATCTGTGATATGAGGAATCAC

CTGAACAGTCGCTCCAAGGTACTCACCTTTACGTTCTTTACGAAGGACTTCACTATAAAT

TTTACCTGTTGTAACATTGGAATATTTATTAAGATTGATATCAATAAAACGTTCATAGTG

ACCCAAATCCAAGTCGGTTTCAGCACCGTCATCTGTCACATATACTTCTCCATGCTGATA

GGGGCTCATCGTCCCTGGATCAATATTAATATAAGGATCAAATTTTTGAATCGTCACCTT

AAGTCCACGATTTTTTAACAATCGGCCAAGACTTGCCGCAACAATTCCTTTGCCAATTGA

TGATACAACACCACCAGTTACAAAAATGTATTTCGTCATAGTACTCCTTTATATAAGCTG

AATAAAAGCATTGCTTTCTTGTTAATTCCCTATTAGTTGGTTCCACATTGGTTAGGTATC

ACCTTAGTTTTCTAAATATAGAGGCAAACTGGCATTATTCTAGAAAACAAAAATAGCCCC

CTATTAACTAGAGAGCTTTTGCCTGACCTCTTAAAGAGGTGCCCGAATAATAATATAAAC

TATAATAGACAATTTGTCAAATGATAATTACATTTTTATTTCTGAACTAAAGACATTAGA

GAACTGGTTCTTCATCTTCTTCATCTTCTTCGTCTTCTTCATTGATATCAACTTCTTCAA

AATCATCTTCAGGGATAATTTCATTCAATTCTGAATCATATGACTCCACTTCTGATTTTT

CATCATCTGGATCTTCTTCATCATATTCAACCTCAGCTGATTCTTCTGTGAAATCTTCAT

CTTCTGGATCATCATCTCTATAATCAATAGCATCTTCATCTCCATCCATGAAAGCATTAA

CGCGTTTTTTCTTACGTTTTTGTGCGCCATCTTCATCTTCTTCTAGAGTAATAATTTCTT

CATCAATTTCATCAATAGCATACCACGAACGAAGTCCCCATTTGTTTTCACCTAGAGGAA

TAAATGAGCCATCCGTATTTAAGTCAGTATAGAAAAATGGTAATGCATGACGAATACCTG

CATCAGATTTTCCTAAGTAATTTTGAATCTCATTGACTAAATCGCTAAAGTACATCTCAT

TGTCGCGACCGCGTTCTTCCAAAATGGCACGCGCCACTTCAATCATGGAAAGCTCGCTTT

TTTCTTGTCCTGCAAATACGTCTAATTTCAAGGCAGTTCTCCTTATTTTATCATTCTCTA

CCATTTTACGCTAAAAAAAGTAATTCGTCAAAACTTTTATCAACTCATATAACTTCTTCT

TGACAAATCTTTAAAGCTAATTCATTAAAATAACGTCTAACTAATTCCTTATTATTTCTT

TAGAAAAAGAAAAACCAGACTAGAAAGACGTTCTAATCCGGCTTGTACGTTTTGTTCCTT

AATAAGATTACTTAACGCTTGCTGTGCTTGTAATCACTTCAACTGCTTTTTTCATTGCAA

TATCATGTTTCAACATATCTGCTGAAAGAAGAGAACGAACTTGGTCAGCTGGCATGTTAT

ATTCTGTTGCAAGGTCATTAATTTCTTGTTCAATTTCACTATCTGTTGCTTCAAAACCTT

CTGCTTTAGCAATTGCTTCAATAACAAGGTTTGTTTTAACACGTTTGTCAGCTTCAGCTG

AATATTGGTTATGTAAATCTTCTTGAGTTGTACCAGTCAATTGGAAGTACATTTCAGGTG

AGATTCCTTGACGTTGCATGTTGCCCATAAATTCATTCACTGAACGGTTGACTTCTTCAT

GAATCATTTCTTCGGGTAAATCAACAATTTCAGCATTTGCAACTGCTAATTCAATCGCAG

CTCCTTCAACAGCATCATCATAAGCAGTTTCTTGAGCTGCTTCAAGTTCTTTACGATATT

TTACTTTTAAGTCTTCAAGTGTGTCAACATCTTCATCAATATCTTTTGCAAGCTCATCAT

CAAGCTCTGGTACTTCTTTTGTTTTGACTTCGTGAATAGTTGTCATAAATTTAGCGGCTT

TACCTGCAAGATCTTCTGCTTGGTAAGATTCTGGGAATGTGACATTAACTTCTACTTCAT

CGCCAGCTTTAGCACCAACTAGTTGATCTTCAAAACCTGGGATAAATTGTCCTGAACCAA

GTTCAAGAGAGAAGTTATCTCCTTTACCGCCATCAAACTCAACACCATCAACTGAACCAA

CAAAGTCAATCACAACAGTGTCACCTTGAGCTGCTTCACCGTCTTTAATAATGAGTTCCG

CAAGGTTTTGACGTTCGCGCTCAATTTTAGCATCCACGTCTTCATCTGAAACTTCTTTTG

AAGCATCAACTTCTACAACTAGGTTTTTATAATCACCAAGTTTCACTTCAGGTTTTGTCA

CAACTTCAGCAGAAAGTGTCCACTCTTTCCCTTTTTCCATTGACACAACATCGATTTTTG

GTTGTGCAACCACATCAAGACCAAGTTCCGTCACAGCTGCTTCATAAGCTTCTGGCAATA

CAATATTCAAAGCATCTTCATAAAGAACTTCTTCACCAAATTTTTGGTTGAAGACTGGAC

GAGGCATGTGTCCTTTACGGAAACCTGGTGCATTTAAGTCTTTTTTGATTTTATTAAAAG

CTTTATCAAGAGCTGGTTTTATTTTATCTTGACTGATTGTAAATGTAATCACGCCACGAT

TTGTAGCTTTGTTTTCAAATGATGTAGACATTAAGTCATTTCTCCTTAAAATAATTTCGA

TACAGTTCATTTTATCATAATTTAAAGCGTTTTTAAAGCTTTTTATGCGTGAAATCAAAC

GCGCTATTAAGAGTATCTACCTGTCATTAGTCTACTAACAGTTATTTTCAATTTATAAAG

GTTAATGTTAGACTTTTTCGCTTACTAACTGACTATTTTCAAGCATTATCACAACGGATG

ACTATGGCATTCTTTATGATAAAACAATTATCATAACTTTGGTTAAAATATTAATTTATC

TCTTTTCAAGATTGGTTTTTTTTTGATAAACTATCAAAGAAAGGATTTCTCATGACATTT

ATATTACATTACCTAGAGCAGTCTCATATCGAAAATATTGGCTTAACGATTTTTAAAAAG

CTAGTGTCTCTCATTATTCTATTACTCTTTTTTGCTATTTTAAAACGTGTTACTAACTAT

CTTTTTGAAAAAACCATTAACAAATCGTTTGCTTATTCAAGACAAAGTGAAGCTCGCAAA

AAAACACTCTCTAAGCTCACTCACAACATTTTAAATTACTTATTGTATTTTCTTCTCATT

TATTGGATACTTAGCTTATTTGGCATACCTGTCTCTAGTCTTCTAGCAGGAGCTGGAATT

GCTGGAGTAGCAATCGGGTTAGGTGCACAGGGATTTTTATCTGACGTTGTCAATGGCTTT

TTTATCTTATTTGAGAATCAATTTGAAGTAGGAGATAACGTTACTATCTCTGACATAGAA

GGCAGTGTCTTTGGTGTTGGAATTAGAACTACTCAAATTCGTGGATTCGATGGAACACTA

CATTTCATCCCAAACCGTAGCATCACCGTTGTCAGCAACAAATCTCGTGGTAATATGCGT

GCCTTAATTGAAATTCCTCTTTATTCCACTGTTAATTTGAGCCAAGTCACTCGAATCATT

GATGAAGTGAATCAAAAGGAATTGCCAAACCATCCTCAAATCGTTGGCAAACCAAATATT

TTAGGGCCTCAAAATAACTCTAATGGACAGTTCACTTTTAGAATTGCTATTTTCACAGAA

AATGGAGAACAATTTAAGATTTACCATACTTTTTATAGACTTTATCAAGAAGCTTTACTC

AAAGAAGGCATTCAATTACCGACTGCAATCAGCTATTCTATCACCTCAAAATCTTAATAT

AATCTTAAAAAATCCTTTTTTCTAGAAAAAACACCAATTGTTTTATTCCAGAAAAAAGGG

TTTAGGATTTACGGATAATATCCATATTGTAATCAGCCCTAGCTCCACCAATTAATTTTG

GTCGACTAGCCAAAGCTGTCACATGAGCACCTCCTAGCTCTCGTTGACAAGGAATCAGAG

GGATCTGAACACGCTTAATATGCATTCCTATAGCAGTATCACCTATATCAAGTCCAGCGT

GTGCAATAACTTCTTCTACTTCGACAGGGTCTGACATAAGTTGAAAAGCAGCCATCTGAG

CACTGCCACCAGCATGAAGATTCGGAACCACATTGACAATCTCTAACTGCTTAGATTCTG

CCACATGCCTCTCTACTACCAAGGCACGATTGACGTGTTCACAACCTTGTACAGCCAAAT

GAACACCACGCTTATTTAACTCATCTAACACAACTTCAACAACTATTTGACCTACTTCTA

GACTAGATTGTTTTCCAATTCTACTACCCAAAATTTCGCTAGATGAAAGACCTAGAACAA

AAAGATTACCTGGTTGAATAGCAGAGCGTTCTACAACATCAATAACGATTTCCCTTGTTT

GTTTCTCTAAATTATTCAACAACTTCTCACTCTCCTTATCTCTTTTTTTGCCTTAATATG

GCCTTAAAGATAATAAAACCAACAAAAAGTCCTAATGTATTTTGCATGACGTTTCCCCAA

ATACCGGCTAAAGATCCCGAAAGGCCATAACCAAGCATAAGAGAGCCTAAAAAATACCAA

AAAATCATGATAAAAGAACCTATCACAACACCAAACCAACGCTTGCGTCCTCTCCATCCC

GCAAAATACCCTTGGACACTGTGAGCAATCAAACTATGAAACATCCACTGAGGGTATCCT

GCCACTAAATCGATTAAAAAACCTGATAACCCTCCCACAATAGCTCCTTGAGCTGATCCA

AAGGAAAAGCTGACAGCATAAATTCCTGCATCCAACAGCGTTAAAAAACCTGTTGGAGTA

GGTAGCATGACAAATCTTCCTAAAACAACAACCAATGCTGTCAATATCCCTGTTAAACTC

ATTTGTCGTATCTTACTATTTTGCATAACTTTGTTTTACTCCGTATCTATCAGATTGTAA

AATAGCTTGGTAGACTAACTCTTTTGAGTTTTTCACAGCTTCTAGTGGCGTTTTTTTCTT

TACTAACTGACTAGCGATACTAGATGCAAAAGTACAGCCTGCGCCAATATTATTTTTCTC

AAGTACAGGACATTCCAAGGTAACAATCTCTTTACCGTCATAAAAAAGATCAATAGCCTT

TTTCTGACTAAATCGATTACCACCCTTGATAACCACCTGTTTTGCCCCAAGTTGATAAAA

ATATTTAGCAGCTTCTTGCATATCTTTTAAAGAAACAATCTCTTTTTGAGATAATAGCTG

AGCCTCTACCAAATTTGGAGTAACAACAGTTACATAAGGCAATAATTGTAAAATCTCCTG

TCTTAGAGGAACTATTTTAACATCATCTATTTCTTTACATGCTAGAACAGGATCTAAAAC

CACTGGGATTCCCACATGCCCCTTTATAAAATCCAAAACTATTTCACACATTTCCGCATT

AGGTAACAAACCAATTTTAATAGCAGAAATTGGAGCGTTCGTAAAGCTGTTCAACTGATC

ACGGAAAATTTCTTTAGCTACCGGAAATAGACTAAAGCCCTCTTCAGATCTAGTAGTTAA

ACAAGTTACCGCTACAAAGGCCTGTAGGTCATACCTTATGTAAGTCGCTAAATCAGCATA

GAGGCCACCTCCACTTAAAATATCGTTCCCAGATATAGTCACGATATAATCAGTTTTCAT

AACAAATCTCCTTCAAGTAAAGCCCATTTCCAGATATCGTTGGTCCAGCTAGCTGACGAT

TTTTGGAGCTCAAAATCACTTTAACTTGTTCAACTGGCATTTGACCATTACCAATTTTCA

GCAAAGTTCCCACCATATTCCTCACCTGCTTATAGAGAAAACCATTTCCTGAAAAAGTAA

ACACCAAAAAATCAGTTTTTTCATCCCGAGATACCGTTGCTTTGGTAATCGTTCGCACCT

TATTTTGTACCGAAGTTCCTGCTGCTGTAAAACCAGTAAAATCGTGTGTGCCTACCAGAC

CGTTAATAGCTTCTTGCATAAGTTTAATATTAAGAGTATAAGGGTAATGAGTGGTATAGT

GTCGCATCATCGGATTTTTAGGTCGACCATTATCAACTAAAAATTCATAAGTTTTTAAAT

GCTTCTGATAACGACAATGAAAATCATCAGCCACTTTTTCTATATTAACAACATCAATAT

CTTCAGGAGTCTGTGTATCAAGAGCAAATCGTAATTTTTCAACTTCTTGTTCTTGAGGCA

AATCAAAATGGATCACCTGTCCATAAGCATGTACTCCTGCATCCGTACGTCCTGCACCAT

GAATTATAATTTTTGTACCGTTATTTAATTTATAGAGTGTTTTTTCAATCTCTTCTTGAA

CCGTACGTAAATGTCTTTGTCTTTGAAAACCCGAAAAAAGAGTCCCATCATAGGAAATCG

TTGCTTTGTATCTTACCATGTTACTATTTTAACATTTCTTAAACGTAAGAGAAACTATTT

TTAATCAAACTGTACCCAAACAAATGAAAGACGTATTGAAGCTAACTCTAAGCTACTAAC

GATTTAAAAGCTATAAGAAAGCTATTTCCTCCAAACTCTCTCTCCTTTTTTTATAGATTT

ACCCCCAATTCCT

>18S-48_L8_1_3

ACTTTATTATACCAAGTCCAACCAAATGTTAGACAATAAATCTAACTTAAGGAAGCTAGC

AGCATGAGAATCCAGATGGTCAAGAGTGCCAAACTTAAGCTGATGGGCACACCCAGAAGA

ATTTGTTTTTTGAAAGCAAGACTGCGTTCCTCTATATGGGGAAGGGAGAGTTGAATGAGG

GAACCAGCTGATGAAAAAGGAGAGATATTAGTAGATAGAGCGCCAATAATAGTGGCTGTT

GTGAGTAAGCGAATATCAATGTGAGGACTTTGAGCACTGATGGTAGCAATGATGGGAAAG

AGAGTTGGGGCTACAACGGATAGGGTGGAACTAAAGAGTGACATCACTCCGGCTATCACA

CAAAAGAACAGAGGTAACCAGAAATGAGGAATGGTTGTTGTTATGAGGTGCCCTATCAGT

GTGACTAAACCTGACTTGACCGCTAGAGACATTAGTAAGCTCATGCCGCAGAGCATGATA

ATTATAGCCCAGGGAACCTTAGCCAAAATGGCTTCTTGCTTCCCTAATTTGAGCCTTAAG

GCGAGGCAGACCATGAGTATTGAGACAAAGCCAATATCAAATGTTTGTCGATAAGTAGCT

ATCCATGCGATGTTTGGGAAAATGAGATGCAACAAGGGAAAAAGCCAAACCAAAACCATG

CTGCTGATCATGAGCAAGGTGGTTTGTCTTTGGACCTTGCTGAGGACTGGTGGTTGGTCA

ATAGTCAAGGATGAGTTTGTTCTTCCCTTACTGTAGCGGATGTAACAAGATAATAAAAGC

AAGACGATGAGTGGGTAGATAATGCTGACGATAAAGATATGATTGCCAAGTGAAAAAGCT

TGCTCTTCCCATCCCATTTGCTTAAACAGGCCTTGAAAGACAATGCCTGAGCCACTGGTT

ATCAAATTAGCCCCTCCTGAAGCTCCCCAATTGACGGCTTGAGCTCCAATCAAAGGGTGT

TTGTCCGCTTTTTGACAGAGGGTAATCGCTAGAGGACAGCAAACGGCCATAGTAGTGAAA

AATCCAGCACCTAAAGCAGACAAAAGGGTTGCTATCAGGTATAAAATCATGTAGATGGCG

TTAGGGTGGGTGCGTGTGCGGTAGAGAATGTGTTGAGCCAAAACATCAAGAGTGCCGTTA

GTTGTTGCAACGTTATAAAAGAGAGAGACGCTAAAAATGGTAAAAAAGAGTGAGGTTGGC

CAAAAATGAAGAAGTTCTTTGGGGCCTAATCCCATGAGAGTGGTTGCGATGAGGTAAGAA

AAAGCAATAGCCAGTAGGCCAATATTGATTTTGGTGCGGTAACCAATTCCAATGGCTAGA

GCAATGGCGCTAATTATTATTAAATGAATCATTGAATTGTCCTTTAGTTAGAATATGAAA

ATAGGTTGGCAATTTTGGCGCTCAAAAGACCACCTGTTTCCTATTGGTACTTGACTATTA

TACACAAAAAAATTACAATTGTCTTGACACTTGTTGGGATTTAAAATGAGGTATTCAGGT

TTGTGATTTCCTACGGAAGTTAATAGCAATTGCCTCTAGTGCTTACAAGTGATATTAAAA

ATAGAGGACTTAGTGATGTCAATCATTTCAACTGATTTAACCCCTTTTCAAATAGATGAT

ACATTGAAAGCAGCCTTGCGAGAAGATGTTCATTCCGAAGATTACAGTACCAATGCCATT

TTTGATCATCATGGCCAAGCCAAGGTGTCGCTTTTTGCCAAGGAAGCTGGTGTTTTAGCG

GGGCTAACCGTTTTTCAAAGGGTTTTTACCCTATTTGATGCCGAGGTGACCTTCCAGAAT

CCTCATCAATTTAAGGATGGGGATCGTTTGACTAGTGGCGATTTGGTTTTAGAAATCATA

GGCTCGGTGAGAAGCCTCTTAACATGTGAACGCGTTGCCTTGAATTTTTTACAACATTTA

TCAGGGATTGCTTCGATGACAGCTGCTTATGTAGAAGCCTTAGGCGATGATCGCATTAAG

GTATTTGATACTCGAAAAACCACTCCTAATTTACGTCTTTTTGAGAAATATGCCGTGAGA

GTTGGCGGTGGCTATAATCATCGCTTTAATTTATCAGATGCTATCATGCTAAAAGACAAT

CACATTGCGGCAGTAGGTAGTGTTCAAAAGGCGATTGCTCAAGCGCGTGCCTATGCCCCT

TTTGTGAAAATGGTCGAGGTGGAAGTGGAAAGCCTTGCTGCTGCCGAAGAAGCTGCGGCG

GCGGGTGTTGATATTATCATGTTGGATAATATGTCATTGGAACAGATTGAACAGGCCATT

ACTCTAATTGCAGGACGTTCTCGGATTGAATGTTCTGGAAATATTGATATGACCACTATT

AGCCGTTTTCGTGGTTTAGCGATTGATTACGTCTCCAGTGGTAGTTTAACCCATAGTGCT

AAGAGTCTCGATTTTTCCATGAAGGGTTTAACCTACCTTGATGTCTAAGTTGCAAAATAA

ACTAACTTTTTAAAGGATGTCTTTCCTCTAGAACGAGTTTTATGTCAGATAGTTTAAACG

CCTCTTCAAATACTTTCCCTGTTTTCACCTGATTCGTGTCCGCTTATTATACCAATTCCA

AGCATTCACCTGAAAAAACGATAGGGAGAGGTTAGTGTTTTTATTGTAAAAGTTCCACGC

CAAATCCATGAAGAAAATTTTGTTTTCAGAAATCACGATTCATCATAATAAAATCAATCA

AAGTCGTTAACTAGGAGTGTACTGACCCCAAAAAGTTGGACAAATAATTATTGAAAGGAT

TTAGTTCTGTATTGGACAGGACTAAATCCTTTTAGTTTTGCTTTGATTCGTTTGTTGTTG

TAGTAAAAAATGTAATCTGTAATAGCTTCTTCAAGCTTGTCAAGTGATTGATAAGTTGTC

TCGAGTCCATAAAACATTTCAGATTTGAGAATGCCAAAGAAGGAATCCATCATGCCGTTA

TCCGGGCTATTCCCTTTGCGGGACATGGATGGCAGAATACCTTTAGACTCTAAAAAATCA

TGGTAGGACTAGTGTTGGTATTGCCACCCCTGGTCACTATGAAGAATAGTTCCGCTGTAT

GAATCCGCTGGAAAAGTCTTCTCAAGCATGGTTTGAACCTGTTTCAAGTTAGGAGACCGA

GACAGAGTGAAATCAATAATCTCACTGTTATAGCCGTCAAGAACAGGTGATAAGAAGAGT

TTTCTTTCAGGTAAGGCCAACTCCGTCACATCAGTGTAACATTTTTCGTAAGGTTTAGAG

CCTTCGAAGTGACGTTTAATCAGATTATCAGCCTTCTTACCAACCTCACCTTTGTAAGAA

GAGTACTTGCGCTTACGACGAATGCGGGCTGCTAGGCCCATGACTTTCATGAGACGTTGA

ACTTTCTTATGATTGACCACAAATCCTCGGTTACGCAACTCCATATGAATACGACGATAA

CCGTAATTACCTTTATGCTCATCATAAATCTCTCGAATCACATGCTTTAGTTCTATATCC

TTATCTCCTTGAGCTAGTCGCTTCACTTGATAGTAATAGGTGGAGCGTGATAAATCAAGG

ATTTCAAGTAGCATCTCTAAGGGAAATTGATTCCTTAATGCTTGAATGATTTCTGTTGCT

CTTTGAGTTTTGCTTCGTCTCTCAAGCGGTATTCTCTCAGCTTTTTTAGCACAGCATTCT

CCGCTCTAAGATATTCTAATTCCTTCTGAAGACGTTCAACCTCTGTCATCTCTTCCAAAT

TTTTCTTACGTTTACGCCCCATCTTACTCGGTCTCCCTCTTGGTTTTTCAAGAATAGTAT

AACCGTTTTTCTTGTATTGCGCTATCCACCTTGAGAGCATACTAGAAGTTGGTAAAGCAT

AATCTAAGGATGTTTGTTTCTGAGATTGGCCGTCAATCAAGACTTTATTTATTATCTCTT

GCTTCAGTTCTGGAGAATAATAATTATTCTTACATTTTTGAACAATGGTTACCCCATACC

GATCAATCAAGCGAATCATATATTTGACATCGGATTCTGCCATATCAAACTTTTTTGATA

TGGATTTAATGGACTCTCCCATTTGCCGTAACTCATAAATCTTAACTTTCGTTTCTTGAT

TAAATTTCATAAAAAAATACCACCCCAATCGTTAGATTTTGTGTCTAACTTTTGGGGTGC

AGTTCAGTCCAGACGAGGAGGTTTTTATCTGTAACATTATTTTGCAAATGCTAATGTATC

ATATTCATATGAAATTCACAAGATACAATACTCAGTAATAGTCTACATAGCGTAAATATT

CATCTGATATTTGCCTAAAACGATCTTTCATGTTAAGATATAAGAAGAAAAATCAATAGA

TGAGGTATTTAACGTGGCATTTGGAGAAAATGGACCTCGCAAAAAAACAACCTTTGAAAA

AGTGACAATGGTCGTTGTTATCCTAATGGTTCTTGTAACTGTTGGCGGGCTTATTGCGAG

TGCCCTATCAGTATTAATGTAAGGAAACGCTGAAAGGCGTTTTTTTAAAGTAAAAGAAAG

TTAGAGAATAAAAAAAGTATGAGTATGTTTTTAGATACGGCAAAAATCAGTGTCCAAGCA

GGCCGTGGTGGCGATGGTATGGTAGCTTTTCGTCGTGAAAAGTACGTCCCAAATGGCGGT

CCTTGGGGAGGAGACGGCGGTAAAGGTGGCTCAGTTATCTTCCGAGTGGACGAAGGTTTG

CGTACCTTGATGGATTTCCGGTATAATCGTAAATTCAAGGCGAAATCTGGTGAAAAAGGT

ATGACCAAAGGCATGCACGGCCGTGGTGCAGAAGATTTGATTGTCTTTGTTCCTCAAGGG

ACAACGGTTCGTGATGCTGAAACTGGAAAAGTAATTACAGACTTAGTAGAACATGGTCAA

GAAGTGGTCATTGCTAAGGGTGGTCGTGGTGGCCGTGGGAATATTCGTTTTGCAACTCCT

CGTAATCCTGCGCCAGAGATTGCTGAAAATGGTGAGCCAGGTGAGGAACGTCAGTTAGAG

TTAGAACTCAAAATCTTAGCTGACGTTGGCTTGGTCGGATTCCCATCTGTTGGGAAATCA

ACTTTGTTAAGTGTTGTGTCATCAGCTAAACCGAAAATTGGTGCCTATCATTTTACCACT

ATTGTTCCTAATCTTGGAATGGTTCGTACAAAGTCAGGTGATAGCTTTGCTATGGCAGAT

CTGCCAGGTTTGATTGAAGGTGCTAGCCAAGGGATTGGTTTGGGAACTCAATTCCTCCGT

CATATCGAGCGGACACGGGTTATTCTTCATGTGATTGACATGTCTGCTAGTGAGGGTCGT

GATCCTTACGAAGATTATGTTTCCATTAATAACGAGCTGGAAACCTATAATTTGCGATTG

ATGGAGCGCCCACAGATTATTGTGGCTAATAAAATGGACATGCCAGAGGCTCAAGAAAAC

TTGAAAGCCTTCAAGAAAAAATTGGCTGCCCAATATGACGAGTTTGATGATTTGCCAATG

ATTTTCCCTATTTCAAGCCTAGCACATCAAGGCTTAGAGAACTTGTTAGAAGCAACGGCA

GAATTACTTGCTAAAACAGACGAATTCTTATTATATGATGAAGCTGATCTGGTGGATGAA

GAAGCTTACTATGGCTTTGCAGAAACTGAAAAAGACTTTGAGATTACCCGAGATGATGAT

GCTACTTGGGTCTTGTCTGGTGAGAAATTGGAACGTCTCTTTGTCATGACCAATATGGAA

CGTGACGAATCCATCATGAAATTTGCCCGTCAATTGCGCGGTATGGGGGTTGACGAAGCC

CTTCGTGAACGTGGAGCAAAAGACGGGGATCTTGTTCGTATTGGCAAATTTGAATTTGAA

TTTGTGGATTAATGTGGTCTTTAGAAATGGCATTGCCATAGCAACCTTGTGTTTTACCTT

TAAGATGGAGGTTTCTGATTATGGGTGATAAACCGATATCCTTTAAGGATAAGGATGGAA

ATTTTGTATCGGCAGCAGATGTTTGGAATGCTGAGAAATTAGAAGAATTGTTTAATCTTT

TAAATCCCAATAGACGTTTGCGCTTAGAACGTGAAAAATTGAAAAAGGATGAGGCTTAGG

CCCTCATCCTTTTTTGACCTTTTTTTCTTGGTAACGTTGGAGCATTAGCCAATTATAAAA

AGTGTAACTATCATTAATAAGAAAGGTGGCAAAGCAGATGACTACTGCATATTGGCTAGA

ATCTGTTTGAGCAGCATGCATCCATAAGAGAATGAGGACTAGATCATTGAGCCCGTATGC

TAAAGCAAAATAAGGGCTACGCTTATAACTGAGATAAACGGCTGAAAAAGAAGTGGCAAT

CGATAAAGTGGAGACAAGTAAATAGGCTGTTTGAATAGAAGCCAAGATGCCGTAGAAAAT

AAGAGTGACCAGTATAGTAAAAACGAAAAGACATCTTCGATCAGTGGGTGTTAATCGTGA

AATAGTCACTTGTGATTTTTTCCCTTCAAAAGGATGGTTTAACCAAGTAAAAAGTGAAAA

AATAGTCATAGGGAGGGTCATGAAAAGATAAGTCATTAACTCTCCATAGTAACTGTTTCG

TAATGAAAGATAGGCATAGATAATGGAAAAAATAATGACGAGCCCTTGACCAATAGGATT

AGCTTTAGCACTAAAAATGAGAGAGGTCACACCAATCAAAGAAGCTATTAAAGCTAAGGG

AGCTTGGTCACCAAATAAAAAGGCGCTAATTAAAATGGCCAAGACAGAGCTTAACCAAAG

TGCCCACTCGGTTTTTGTAAAGTAGTTTAAATAAGCCATACCCCTATTCAAACATAGGCT

AAGGCATCTGTCAATAGGGGCTTGGTTTTGATAAGAAGTAGCAGGGCAGGTTAGATAGGC

TCTTTAAGATAATAAAATATGTCAAGCTTTAAATTACTTATTTTCCGTGATGATAAGGAA

AAAACAGATTTTTTAAAGCGTTTACATTACCTGTTTTTCTTCTTATATATAGAAAGAGAA

GTGAGCAAAGAAAGGACAAAAGATATGAAACAAAGAAAACACCGTTACCAATTTCCAGAT

GGCTTTTTATGGGGAAGTTCCACATCTGGGCCTCAAAGCGAAGGAACGGTTCCTGGAGAT

GGCAAAGGACCTAGCAACTGGGATTACTGGTTTAGTATTGAGTCAGCTAAGTTTCATCAC

CAAATTGGTCCAGAAAAAACCTCAACCTTTTATGAAAACTATAAGGGTGACATTGCCCTT

TTGAAAGAAACAGGCCACACGATTTTTCGGACCTCAATCCAGTGGTCTCGATTGATTCCA

GAAGGAGTCGGGGAGGTTAATCCAAAGGCGGTGACATTTTACCGAGAAGTATTTCAAGAC

ATTATTGCTCAAGGTATTAAATTGATTGTTAACCTCTATCATTTTGATCTCCCCTATGCC

TTGCAAGAAAAAGGTGGCTGGGAAAACAAAGCTACTGTCTGGGCTTATGAAACCTACGCA

AAAACCTGTTTTGAGCTTTTTGGTGATTTAGTAAACACTTGGATTACCTTTAATGAGCCG

ATTGTTCCAGTCGAATGTGGCTATTTAGGTTATTATCACTACCCTTGTAAAGTAGATGCC

AAGGCAGCAGTTCAAGTGGCTTACAACACACAACTGGCCAGTTCACTGGCGGTCAAAGCT

TGTCATAAGCTTCATCCCGACCACAAGATTAGTATTGTGCTTAACATGACACCTGCTTAT

CCTCGGAGTAATGCTCCTGAAGATGTCAAGGCTGCTAGAATCGCAGAGCTTTTCCAAACC

AAATCTTTCCTAGATCCATCAGTTTTGGGTGTGTACCCAGCAGAGTTAGTTTCCATTTTA

GAGGAAGCTGATTTATTACCTCAATATAGTGCTGATGAATTAGAGATTATCAAAAACAAT

ACTGTTGATTTTTTGGGAGTTAATTATTATCAGCCTTTGCGGGTTCAGGCACCTAGTAAG

TCACAGCAAGAGGGGGACCCCCTTATCTTAGATATTTATTTTGAACCTTATGATATGCCT

GGTAAAAAAGTCAATCCTCACCGGGGTTGGGAAATTTATGAACCAGGACTTTATGATATT

GCCCTTGATTTAAAAGAACACTATGGTAACATCGAATGGCTAGTCACGGAAAATGGTATG

GGTGTCGAAGGTGAAGAAGCTTTTTTAGCAGATGGCCAGATACAAGATGACTACCGCATC

ACCTTTATCGAGGATCATCTTATCCAGCTTCATAAGGCTTTGGGAGAAGGAGCCAACTGC

AAAGGCTACCTCTTGTGGACTTTTATTGATTGCTGGTCTTGGTTAAATGCTTATAAAAAC

CGATATGGTTTGGTAGCTTTGGATTTAGAGAGTCAAAAGCGAACCATAAAAAAATCAGGT

TACTGGTTTAAGGCGTTGAGCGAGTCAAATGGTTTTGATAAATAGGAGGCAATATGGTTG

ATTTAGGATTTTCTCTATACCCTGAGCGGTATGATGTTACCAAGAGTAAAGCTTATATTG

ACTTATGTCATAGCTATGGAGCCAAGCGCTTGTTTATGAGTCTCTTACAGCTGGCACCAG

CTGATCACCAGATGTTTCATTGCTACGCAGAGCTCATTGCTTATGCAAATCAATTAGGCA

TTCGAGTGATTGCTGACGTTAGTCCAAGCTTTATTAGCCAAGCTGGTTGGTCAGATCAGC

TTATTGAACGAGCTCATGCCTTCGGGTTAGCGGGCTTACGCTTAGACGAGGCTCTCCCTC

TAGCAGAGATTGTAACTTTAACAAGAAATCCCTTTGGGCTTAAAATTGAACTTAATATGA

GCACTGATAAACAATTATTGATGTCATTGCTAGCGACAGATGCAGAACGGAGCAATATTA

TTGGTTGCCATAATTTTTATCCACATGAATTTACCGGTTTGTCATGGCAACATTTTAAGG

ATATGTCACGTTTTTATCATGAGCATGATATTGAAACGGCTGCTTTTATTACCGCTCAGT

CAGCATCTGAAGGCCCTTGGCTACTGGCAGAAGGCTTGCCGACAGTGGAAGACCATAGGC

ACTTGCCCATTGGCCTGCAAGTAGAATTAATGAAAGCGATAGGAACCATTGATAATATCC

TCATATCTAACCAATTCATTTCGGAAGAAGAATTGGCAGCATGTACTCAGGCTTTAGCAA

GACCAGTCACAACGATCAAGGTTAGGCCTATCATCGATTTGACAGAAGTTGAGGAGCAAA

TCATTGGCTATCCGCATTGTTATCGTGGCGATGTATCTGATTATGTGATTCGATCAACCA

TGCCACGTCTAGTCTATGCTCAGGAATAGATTGCTCCAAGAGACCAATCTAAGGAGGTCA

AACGAGGTAGCATTATTATTGACAATGACCGCTATCATCGATATAAGGGAGAACTGCAAA

TTGCTTTGAAAAATTTTACGATTAGTTCAAAAGCTAATGTGGTTGCTGAGGTCAGAGAGG

ACTATCTTAGTTTGTTAGATGATTTGCGACCTTGGCAGGAATTTTGTTTGGAGATTGACC

CATCATAGATTCTTTTCACAATCAATGTGGAAAATGATGGTCACCTTTTTCAGGAAATAT

TTGGTACACTAAAAGAGGGTAGAAAGCGTTTTCTAAAGTAGTAAGGAGGACTAATGTTAT

CAAAAAAAGAACTGGCCATCATAACATTTCTAATCCATCACAAAGAGCAGTTTGTCTCTA

GTGCAACTCTGGCTGAGGTTATTGGTATGAGTGACCGTACGGTTAGGAAATACCTCAAAG

AACTGATTAGTAGCCTTCCTGAACACGGAGCTCACATCATTTCTAAACAAGGTCGAGGTT

ACTGCTTAGAAATTGACCATTCTATGGCATTTGACATCTTTTGGCAAGAAAGTGTGACGT

CTAAAAAACGTTTGGCAGATGTGACTCAAGTGGAAGAATCGGTTGATCGAGAAGATTATG

TGCTTCATAAATTCTTTTTTGAAGATGCCGTTCAGGATTTTGAAGAGCTTTGTCAAGAAT

TGTATATCAGTCGGACAACTTTAAAACATGTTTTAGCAGGCATTAAGGAGCGCATAATAC

CTTACCAGTTAGAGCTTGAAATAACTCACCAACATATTCAAATAAGAGGTAAAGAAGAGG

ACATTAGGCATTTTATCATGGACTATTTTTTTGTTACTAGTTTTGATAATACCTTGTCCA

CCATGGTAGGTAATACCTTTCTTGAAGGTATTAATTTTGCAGAAATCATGATTATTGTCT

TGGATGAATGTCGGGATGCTAAGTTGAAACTTTCTGATTTTGTCATGAATAATCTAGTCT

TGCATATTGCCCTAATGGTTCAGCGTATTCGATCGGGGTGTCCATTAGAACTGTTTTCCA

TTCCAATAGCTATTCGTCAGTCTGATGAGTATCAAGTAGCTCTGCGCATTTTGTATCAAG

TGGAAGAAGTGATGGGGATACGTTTTCCTAAAGAGGAGGCTAATTATATTGCACTGCATC

TAAATGTCAAACATTCTGTTGGTAAGCATTGGCAAGATGATAATACCGATGAAAAGTTGC

AGGACCATTTAAAAGCATGTATTGCCAAGATTAGTCAATTGACAGGCATGACTTTAGAAA

CAGATACTAATTTATTTCAAGGTTTATTAGCCCACATGATGCCCTTGACCACACGTCTTG

AAAACCATATTCAGCTGACTAATCCTTTAACTGAGGAAATTAAAAGCCAGTATCCTGAAA

TATTTACCCTTACCAAGCAGACCTTTTCGGATTTGCTTGTATGTCAAAATAATGATGTCT

CAGATGATGAATGGGCTTATATTAGTTTGCATTTAATGGCGGCCATTGAGCGTTATTCAA

ACCGTCATAAATTGAGGGTTCTAGTAGTTTGTGCCACAGGTTATGGTAGTGCCATGATGT

TGAAAAATCGCTTGGAAAGGGAATTTGAAGGACGTTTGCGTATTGTCGATGTTATCAGTT

ACTATGAAATAACCGAAGAGCGACTGAAAACCGTTGATTTGATTATTTCCTCTATCAGCC

TTGCCAATCTGATGTTTTTAACACCAGTGATTACTGTCAGTGTTTTTTTGTCAAATCAGG

ATATTGAAACTATTCGACAATTTATTGGAGAACAAGAAGGTATCAAGAAAGAGGTCAGTT

TACCGTCACAGATGAGTTTAGCAAAAGCAGAGCAGCTTCTAACAGGTGTTTTTAGCCCTA

ATCGTTTTCTTTATTTAGACGAGAAAATCTCAAAGGAAGACTTGCTTTTACGGATGATTG

CTTGTCTTGACGAGGCGGGAACAGAAACGTTTGTAGAGGACTTCTATCATCAAATGGTAC

TGAGGGAAAATTACAGCCCAGTCATCTATGGAGAAGTGCTTGCCTTTCCGCATCCTGCTA

ATCCCATGACTTATTCTGAACAGGTAGTAGTGGCTATTTGCCGAGAGCCTCTTGAGTGGG

ACAAGGCGCATCAAGCTGTTCATTTTGTCTTTCTATTATCCCCGTCTAAAGGGCATAATC

ATAGGTTAAAATATGTATCACCAGGTTTGGCATCTTTTGTCAATCAGGTGGAGCTTCAGC

AAGCGTTACTAGAAGAACCAAATTATTCCAAATTCATGACAGTGTTTACCCCACTTATTC

ATGATTAAAACGATTAAAAGGAGAAACCTTATGAAATCTATTATGTTAGTTTGTAATGCC

GGTATGTCAACTAGTGTGTTGGTGACTAAGATGCAAAAAGCAGCCCAAGCGCGTGACCTT

GAAGTTTCTATTTGGGCAGTTCCTGTGAGTGAAGCTGACAACGAAGTGGCTGCTAATAGC

ATTGACTTTCTCTTGTTAGGTCCACAAGTCAAATTCTTACTAAAGGATTTTAAAGATAAG

TTTGAACCAGATATTAAAGTAGATGCCATTAACATGGCTGATTATGGTCTTATGAACGGT

GAAAAAGTCCTTGAAACAGCCCTAGCGATGATGGAGGAGTAGGCATGACGGAACCATCCA

ACTTAGAAGCTATTATGGGCTTAATCATGTATGGAGGCGAAGCCAAGGGCAAGGCCGTAG

AGGCTATTCATGCAGCACGAGATGGGCAGTTTGAGGAGGCTCAGCACTTACTTCAAGAAG

CGACAAACTCGTTAAATATTGCCCATAAATCGCAGACACATTTGCTCTCCCAAGAAGCAG

CTGGTGATGCGGTTGAATTGTCTTTATTGATGGTGCATGGGCAAGATCACATGATGACTG

CCTTAGCCTTTATTGATTTAGCCAAGGAATTGGTTACCGTTTACGACAAAATGTCAGCAA

TACAAAGAGGTGAGTCATGATTATTTCAGCAGAAGGAGATGTTTCCATTCATCATTGTTT

TGAAGCCATGCTGATATCGCTCCAAGAAGATTATCGACAAAATACCTTGAGACAGTTGTC

AAGAGATGATATCAAAGCGGGGATAAACTATGTTAAACATTATGGTAAATACAAAGAGCG

TACAGTGAGGGTGCTTATTACGGCCCTAGAAGCTCCGCAATTATATACTGCTTGTTTTAG

TTCCAATCATGGGATAAAGGAATTGGCTTATCGATTAGAGGCTCTGGGGGAGAATCGGAC

TAGAATTACCTATTCTTATGACTATCATCCTCTAGATATTTTTCAAAAAGCAAATCATTT

TATTGTTGGCAAACTGTTCAAAAAAAGCCTAGAACGCCAATCTCAAGCTCAGTTAGCAGC

CCTTATCCAATATGCTAAACAGTTGCAGCAACCATCGTCTTTGTGAAGATTATTCATAGA

CCTTGAATGAGAGAGAAGGGTTCATTTGGATAAGAGCCGATTACCGGTTATCTCTAAGCA

TCACGTTTAACAAGGATGATTTTGAGATAATTGTGTTAGGCAGACACCGTTGCTTTTAAG

GTTACCTTTGTTACATTAAAAAAATAAAAAGAGGAGAAGATTTATGTCGCATTTTTCTGA

TAAGTTTATGGAGATTTCAGGAAAAATTGGGTCACAACGGCATTTAGTAGCTATTCGTGA

TTCCTTTATTTCAATGATGCCTATTACCATGGCAGGTTCAGTTGCTGTCTTATTGAACGT

TTTTTTACGCGATATTCCAAACAATATGGGATGGACAGGGTTTGCAAAAGCTATGCAACC

TGTTATCGACATCAATGGCTATGTTTATTTTGGGACAATTGGTATCATGGCGCTCTTTTT

TGCCTTTGCTTTCGGTTACAATTTAGCTGTTATGCATAAGATTAATCCATTGGCGGTAGG

ATTGATTTCTTTTGGCTCATTTATTGCCACCCTACCTCAGACTTTAACCATTTCGACACC

ACTTGAAAATGCCTCTGCTAACCTAATTAGCAATTTAAAAGAAATGGGGCTTTCAGTCGT

TACTGCGGGTGGAGCTTCAAGTATTGAAACTAGTCAATGGGGAGCAATAGCTCTAAAGTA

TGTCGGTGCCACAGGCTTGTTCACAGCTTTAATTATTGGCTTTTTATCAAGTTTTGTGTA

TGCGGCTTTGACCAAACGTAATATTACCATTACCTTACCAGATAATGTACCACCTGCGGT

TAATAAAGCCTTTGCTGCCATTATTCCAGGAACAGTTGCGATTTACGCTTCGGCAATTTT

CGCCTACCTCATCTTCGCATTAACAGGTTCTTCCCTAAGTGATGTCATTTCAACCTATAT

CCAATTACCATTGCTAGGCTTATCACAGGGTATTGGTTCAGTTATCTTGTTAACCTTCTT

AGTTCAACTGCTCTGGTTCTTTGGCTTGCATGGTCATAATGTCTTAGCCCCAGTGATGGA

TGGTATTTATATGGTAGCTTTAACAGAAAATACAGCTGCTTACAATACAGCGCATAGCGC

TGCTAATTTACCATACCTTTGGACACGCGGGTCCTTTGATGCTTATGCTCAAATGGGTGG

TTCAGGAGTAACCTTGGCATTGATTATTGCTATCTTCATATTCTCAAAACGAGAAGAGCA

TAAAACCATTGCCAAATTGTCCGCACCAATGGGTGTTTTCAATATCAACGAACCAATTAC

ATTTGGGATGCCAATTGTTTTGAATCCAACCTTTGTGATTCCATGGCTAATTGTGCCACC

AATTTGTGCTAGCATAGCTTATTTTGCTACTGCAATAGGTTTGATTCCACCGATATTCTT

ATCAGTACCTTGGATTACCCCTGTTGGACTTTATGCTTACCTAGCTACTGGAGGTAATAT

TATGGCAGGTTTGGTTTCACTGTTTAACCTCTTTGTTGCCTTCCTTATCTGGGCACCATT

TGTCATTTTGGCCAATAAAGAAAAAGCGAGTGACCTTGCATGACAGAAAAAAATGGAGTA

ACTTTTTTAATGGCTGTTGGCTTTTTTCTCATTGCCATTAGTCCGATGGTTGGTAACAAA

TACTTACACATAGTGACGGGACTAGCTCTTACGGGATTAGGTTATTACCTCACTAAACAT

AAACGCCCATAAAAAAGCATAGGGGACACTCTTATGAAGGAGAGAGTCATAAGAGTATCC

CTATTTTGCTGGTTTGAATAATAACAGTAACCAAAAAAGGCAAGGAGATAGGTTTCCTTG

CCTTATTATTTTTTGGAAGTTAGTATGGTGTCTGCTCTATTAGACGTTTAAAACCTTGTC

TAAAAATTCAATGAGACGAGGGTGCTTAGGATGATCAAAAATCTCTTCAGGAGTACCATC

TTCCAAGAATTGACCACCGTCTGTAAAGATAACACGGTTAGCTACTTGGCGAGCAAATCC

CATTTCGTGAGTAACAATTAACATGGTCATGCCTTGTTCTGCCAAGTCTTTCATAACGTT

CAAAACGTCCCCAACCATTTCGGGGTCAAGAGCAGAAGTTGGTTCATCAAAGAGCATAAT

ATCTGGGTTCATTGCTAAACTTCTGGCGATGGCGACACGCTGTTTTTGCCCGCCGGATAA

ACTTCCTGGTAAGGCGTCGGCCTTATCAGAAAGTCCGACTTTTTCTAGAAGTGCCATGCC

ATGCTTTTTAGCAACTTCTTTAGACTCTTTACCTAATTCAACAGGAGCAAAGATAATATT

CTCAAGAACAGTCATGTGAGGGAAAAGGTTGAAGTGTTGGAATACCATCCCGATATTTTC

ACGTGCTTTGTCAATATTAGTCTTAGGATCTGATAATTCAAAACCGTCCACCATGACCTT

ACCACTGGTAATCGTCTCTAGAAGGTTGAGGGTACGTAGAAAGGTTGATTTCCCAGAACC

AGAAGGACCAATGATACAAACGACATCACCTTCGTAGAATTTGGCATCAATTCCTTTTAA

GACTTCATTTTGACCATAAGATTTGTGTAAATCTTGAACATCAATTTTTAATTCTGTCAT

TAGTTAAGCCTCTTTTCTAAACGTTTTGCAAGTCTTGTTAAGAGTGTAATCATGATAAGG

TAAATAATTGCTAAAATAGCATACATACGGAACGACTGGTAATTTCTAGCAATAATGATT

TTACCTGTTTGGAAGAGTTCCACTAAACCAATTGCTGAGACGATTGTTGTATCCTTCAAT

GAAATAACAAACTGGTTGATAAAGTTAGGTAACATTAGTTTCACAGCTTGTGGGAGAATT

ACTTTTCTCATCGTGGTTCCGTAAGACAAACCAAGACTTCGACTAGCTTCCATTTGCCCT

GCTGGAACAGCTTCGATACCACCGCGAACAATTTCAGCAATATAGGCTCCGCCATTAAGT

GACAGTGCAATTGTAGCAGCTAAGAAATCATTAATCGGTGACTGGTGGCCGGTCATACTC

TCGATAAGGTTTGGTACTCCCCAGAAAATGAAGGCAGCCACAATCATCAAAGGAATCCCT

CGAACAACGTCCACAAAGGCCGTTGAAATGAGTCGAAGTGATTTAGTTGGTGACACGGCC

ATCATCCCAAAGATGATCCCGATAATTATAGCAATAGCAAATGAAATAAGGGTTAAACTG

AGCGTGGTTCCAAGTCCTGCCAATAGTTGTTTGTAGTTATTTGATAATAGGCCTGAAATA

GTAGACTCATCAGCACCTTTTTCAGAAGGAGTTGCAGCTTTCTTAGAGTCAAGGTATTTA

TCTATAATGTCATCATACTGACCAGATTTTTTGAGAGCAGCTAAGCCATTGTTGAACATT

TCGATTAATTCTGGATTAGTTCCTTTCTTGACAGCAAAACCAACTTCACCAGTAGAAATG

CCCTCAAGAGGTGTTTCAAAGCGACGACCTTGAGAGATAGCGTATTTAAGAACCGCCTCA

TCATCCATGATAGCATCTACAGAACCTGAATTTAAGCTATCATACATGCTAGAACCATCA

TCAAATGCCTTGACATTATAACCATATTTAGGAGCGTTTTCTTTTAACCAAGAGTAAGAT

GAAGTGCCGTTTTTAGCACCTACTGTTTTTCTGTCTAAGTCTTCATAGTTCTTGATGTTT

TTTCCAGCTTTAACAGCTAAAATGATATTAGAAGTATAATAAGGATCAGAAAAATCAAAG

ATAGCTTTACGAGCGTCAGTAATAGTTGCACCAGCAATGACCCCATCTGCTTGGCTAGAT

TGCACAGCATTTAAGGCAGCATCGAAACCTGGATTAGCGATTTCAATTTTGAAACCTTGT

TGTTTAGCAATAGCTTTGATTAATTCTATGTCAATACCAACGTATTTGCCCTTACCATTT

TGAAATTCAAAAGGTGCAAAAGACGAATCAGAGACAATTTTATAACTGTCCTTTATAGGT

GTAGCTTTGGCAGATGGATTTCCCGTTGCCTGACTGGTGGTAGCTTTATCATCTGTGCCT

AACCACTTGGTCATGATAGCTTGGTAGGTACCATCAGCTTTCATAGCTTTAAGAGCTGTA

TTGAAATCATTAACTAGATAATCATATCCGCTTCCCTTTTTGACAGCAAACCCAAAGCTT

CCAATGGGCTCTCCTTTCATGTTAATAGCAATATCTTGGTTTTGGCTGATTGCGTATTGG

ATAACCGCCTCATCATCCATAACAGCGGCAATAGAACCAGCAGATAAACTATTATACATA

AGATCACCTGTGTCAAATGTTTTAACAGTATAATCATACTTTTTTTTATAGTTATTCAAA

AAGGCTTGAGCCGCTGTTCCATTTTTAACACCGACCGTTTTTCCTTTTAAGTCACTGTAT

TTTTTGATGGCATTGGCTTTACGTGTCGCAATGACAATTTTGGTATCGTAATATGGCTCT

GAGAAATGAAAGACTTTCTTACGAGCATTCGTAATGGTTGTACCGGCCATTAGAGCACTC

GCTTGACCAGATTGTACAGCATTTACAGCTGCATCAAAACCCGGGAAACTCATACTGAAA

TCCCAAGATTGACGTTTGGCTACTTCATTAATAATATCAACGTCAATTCCTTTGTAAATT

TGATCTGAGTCTTTAAATTCAAATGGGGCATAAGCTGTATCTGAAACAATAGCAATAGTT

TCAGCACTTGCAATATTGCATGTCAAAAAAATAGACATTATCGCAAGCAGCAATACTTTT

ATTTTGTGCGTCATACTAATCTCCTTATAAAACTAAAAAATATGGTTAATTATAACAAAA

TGTTGAACATTAAGCAAGAAATTTGTTATTTATATGTCATAAAACATAACATGTAAATGA

CATGTTTTTTACGCTTATTGGAGGTGATTCTGGCTTATCTATGTTAAAATAGTTTATAAG

AGATAAGACTATTAGAGGGTATAAAATGATTGATAAACGTGATGATAAACCATTTAAACT

AAAATCAAAGTATAAACCGTCTGGTGATCAGCCACAAGCTATTGAAAGCCTAGTTGATAA

TATCGAGGGCGGAGAAAAAGCTCAGATTCTCCTAGGTGCTACAGGAACGGGAAAAACATA

CACCATGAGCCAAGTAATTAGTAAAGTAAATAAACCTACCTTAGTGATAGCTCACAATAA

AACCTTAGCAGGACAATTATATGGTGAATTTAAAGAATTTTTTCCAGACAATGCTGTGGA

ATACTTTGTTTCCTATTATGACTACTACCAACCTGAAGCATATGTCCCATCAAGCGACAC

CTATATCGAAAAAGATAGTTCAGTCAATGATGAAATCGATAAACTTCGCCATTCAGCCAC

CTCTTCTTTACTTGAGCGTAACGATGTCATAGTGGTAGCTTCTGTATCTTGTATTTATGG

ACTAGGTTCGCCAAAAGAGTACGCAGATTCAGCAGTCAGCCTACGTCCAGGTCAGGAAAT

ATCACGTGACACTTTGCTAAATCAATTAGTAGATATTCAATTTGAGCGTAATGATATTGA

TTTTCAACGCGGCTGTTTTCGTGTACGTGGCGATGTTGTTGAGGTCTTTCCAGCATCCAG

GGATGAACATGCTTTTCGGGTGGAATTTTTCGGGGATGAGATTGACCGTATATGTGAAAT

CGAAAGTTTAACTGGGAAAACTATTGGAGAAGTTGACCATTTGGTTTTATTTCCAGCTAC

TCACTTTGTAACAAATGATGAGCATATGGAGCAATCAATAGCCAAAATTCAAGCAGAGTT

AGCAGAGCAGTTACAGTTATTTGAATCTGAAGGTAAACTACTAGAAGCACAGCGATTACG

CCAAAGAACGGAATATGATATTGAAATGTTACGTGAAATGGGCTATACTAGCGGAGTCGA

AAACTATTCCCGTCATATGGATGGCAGGTCACCAGGAGAACCTCCTTATACCTTGTTAGA

TTTCTTCCCAGAAGATTTTTTAATTATGATTGATGAAAGTCACATGACAATGGGACAAAT

CAAAGGCATGTATAATGGTGACCAAGCCAGAAAACAAATGCTAGTTGACTATGGTTTTCG

ATTGCCATCAGCTCTTGATAATCGTCCCTTAAGACGCGAGGAATTTGAAAGTCATGTGCA

CCAAATTGTCTATGTTTCAGCAACGCCTGGTGAATACGAAATGTCACAGACTAATACAAT

TATTGAGCAAATTATTCGTCCGACTGGGCTACTTGATCCTGAAATTGATGTAAGGTCAAG

TATGGGGCAAATGGATGATTTGTTGGGAGAAATTAACCAGCGCGTAGCGCGCGATGAACG

GACGTTCATCACAACCTTAACTAAAAAAATGGCAGAAGATTTGACTGATTATCTAAAAGA

GATGGGTGTCAAGGTCAAGTATATGCACAGTGATATTAAAACCTTAGAGCGGACAGAAAT

TATTCGTGATTTACGACTTGGTATTTTTGATGTCTTGATTGGAATCAATCTGCTCCGTGA

AGGGATTGATGTGCCAGAAGTTAGTCTTGTAGCCATTTTGGATGCTGATAAAGAGGGTTT

CCTCCGTAATGAACGCGGCTTGATTCAGACCATCGGTAGAGCAGCTCGAAACGTAGATGG

TCATGTCATTATGTATGCGGACAAGATGACAGATTCTATGCAGCGGGCTATTGACGAAAC

GGCTCGTCGTCGTGAGATTCAAATAGCATATAACAAAGCGCACGGCATCGTTCCACAAAC

TATAAAAAAAGACATCAGAGGCCTTATTAGTATAAGTAAAACAAGCCACAATGATATATC

AAAAGAAGAGATGGATTATGAAAGTATGTCAAGAGGAGAACGAAAAGAAGCGATCAATGC

TCTGCAAAAACAAATGCAAGAGGCAGCTGAATTACTTGATTTCGAATTGGCAGCTCAAAT

GCGAGACTTGATTTTAGAACTTAAATTAATGGACTAGGTTTTGAATAAAGCTTATATTAT

ACTTATTAACATATATAAGTTAAAGTCAAATAATTCTTTTTATTAGGACAATTTTAAGTT

AACTTTTTAAAGTAAAGATTAACTTAGTAAGTTTAGTAAAAAGGCATAAAATTTAACTGT

CTAAATAGTTAAGCTTTAAAAAAAATTTTCTTTTTAATAATATGACTTTTAGCTTGTCAA

GTTAGGTTTTTTCGAGTAGAATTTTTTAGGACATTATTTCACTAATACAAGAAGGTAAAG

TATGATAAAAAACAAATCAAGAATGAGAGGAATTGCTGTTTTTATTGGCAAGACAATTTT

ATTTTATCTGATTTTAATGTTGCTGGTCTATTTCTTTGGTTATCTAGGACATGGTCAAAG

TAACTTTATTTATAATGAATTTTAGGAGATTGACTGATGATTAAGGATATGATTGACAGT

ATTGAGCAGTTTGCTCAGACACAGGCTGATTTCCCAGTTTATGATTGTTTAGGGGAACGC

CGAACTTACGGACAACTCAAAAGAGATTCTGATAGCATTGCTGCATTTATAGATAGCTTA

GCTTTACTGGCAAAATCTCCAGTTTTGGTTTTTGGGGCGCAAACTTATGATATGTTAGCT

ACTTTTGTAGCTTTAACCAAATCTGGTCATGCCTATATCCCAGTAGATGTGCATTCAGCA

CCAGAACGTATTTTAGCGATTATTGAGATTGCAAAGCCCAGTTTAATCATTGCTATTGAG

GAATTCCCTCTTACTATTGAAGGGATTTCCCTTGTCTCACTATCAGAGATTGAGTCAGCA

AAATTAGCAGAAATGCCCTATGAGCGAACACATTCTGTTAAAGGAGATGATAATTATTAC
[truncated: 1,474,541 more chars]
