# Supplementary material for: GAS‐J, a User‐Friendly Browser Application for Genome Assembly, emm‐Typing, MLST Typing, and Virulence Factor Gene Detection of Streptococcus pyogenes
Source: Microbiol Immunol. 2025 Apr 20;69(7):384–8. doi: 10.1111/1348-0421.13223 (PMC12232109; doi:10.1111/1348-0421.13223)
Supplement: Supplementary file 2 — Supplemental_Dataset2_consensus_emm. [file MIM-69-384-s002.docx]

>Consensus_seq_for_emm_detection

CTAAATCTTTTTCAACCTGTTTCTTAGCTTCACGTGATGCGTCCAAGTCACGGCGAAGACCTTGACGGCTTGCGTCTGAGATTTGTTTTTCTTCTTTAACCTTATCAAGTTCAGCAGTCAAGTTTGCTAAATCTTTTTCAACCTGTTTCTTAGCTTCACGTGATGCGTCCAAGTCACGGCGAAGACCTTGACGGCTTGCGTCTGAGATTTGTTTTTCTTCTTTAAGTTTTTTCTGCTCTGCTTCTTTTTCTTTTCGCTCTGCTTCTTTTTCATTGATATAGCGAAGGGCACCGTCAAGATGTTCTTGCTTATCCGCTAATTTGTCTTCTAAAGCTTTTCGCTCTGCTTCTTTTTTGTCATGATCATGTCTCTCGTTTTTAAGTCGATTCAACTCAGCTTCTTCGTCATGATCATGTCTCTCGTTTTTAAGTCGTTCCAACTCAACTTCATCGTTAAGCTTCTCAACATCATCTTTAAGGTTTTTTAACTCTTGTTCCTTTTTCTGTAAATCAGTCTTAAGCTCTTCATTTTCCGATTTAAGTTTGTCATTTTCTTCTATTTGATGTTACCAAAGCTGAGCCGGTACAGTGTNCCCCTTTAGTACTTCATTTCTAGCATCGTCANCTCTAGGGTGATCAGCGCCTTAACTTCTGTCTGGCTTGCTAACCCTGCTCCTACGACTGTTAAAGCAACCGCTACTGAAGCAGTACCTTTTTTTAATTTTCTAAGCGAATACTGTCTATTCGTATCTTTTCTAACCAT
